# 33rd Annual Meeting & Pre-Conference Programs of the Society for Immunotherapy of Cancer (SITC 2018): Washington, D.C., USA. 7-11 November 2018

**DOI:** 10.1186/s40425-018-0422-y

**Published:** 2018-11-06

## Poster Presentations

### (ADCC/CDC)

#### P1 The identification of potent anti-tumor antibodies for ADC therapeutics from patients undergoing immunotherapy

##### Alexander Scholz, PhD^1^, Jerald Aurellano^1^, Michael Harbell, MS PhD^1^, Danhui Zhang, MD PhD^1^, Samantha O'Connor^1^, May Sumi, BS^1^, Beatriz Millare, BS^1^, Felix Chu, MS^1^, Sheila Fernandez^1^, Cathrin Czupalla^1^, Iraz Aydin, PhD^1^, Amy Manning-Bog, PhD^1^, Yvonne Leung, BS, PhD^1^, Kevin Williamson, BS PhD^1^, Chantia Carroll^1^, Dongkyoon Kim, BS PhD^1^, Xiaomu Chen, MS PhD^1^, Sean Carroll, BS, PhD^1^, Ish Dhawan, PhD^1^, Ngan Nguyen, BS PhD^1^, Shweta Thyagarajan^1^, Mark Whidden^1^, Gregg Espiritu Santo, BS PhD^1^, Nicole Haaser, MS^1^, Hibah Mahmood^1^, Guy Cavet, PhD^1^, Lawrence Steinman, MD^2^, Tito Serafini, PhD^1^, Wayne Volkmuth, BS PhD^1^, Jonathan Benjamin, MD, PhD^1^, William Robinson, MD^2^, Norman Greenberg, PhD^1^, Daniel Emerling, PhD^1^, Jell DeFalco^1^

###### ^1^Atreca Inc, Redwood City, CA, USA; ^2^Stanford University School of Medicine, Stanford, CA, USA

####### **Correspondence:** Daniel Emerling (d.emerling@atreca.com)

**Background**

Anti-tumor therapy with antibody-drug conjugates (ADCs) is predicated on the identification of antibodies that demonstrate suitable selectivity for tumor cells that are also internalized upon binding their cognate target. Remarkably, only a select number of such antibodies with the propensity to internalize have been identified, limiting the range and breadth of ADC therapeutics in the clinic. Here we show that Atreca’s Immune Repertoire Capture (IRC™) technology can identify potent anti-tumor antibodies with internalization activity applicable for ADC therapeutics from patients undergoing immunotherapy.

**Methods**

We analyzed blood plasmablasts from patients with non-progressing metastatic cancer using IRC™ technology. Briefly, plasmablasts were collected from patients and paired heavy and light chain antibody sequences were then obtained from individual cells. Antibody sequences representing expanded clonal families were subsequently expressed and analyzed for their ability to (i) bind to human tumor and non-tumor tissues and (ii) internalize into cancer cells when labeled with a pH-sensitive dye. Those antibodies with a high internalization rate were directly conjugated with a cytotoxic agent (auristatin MMAE) and tested in an in vitro ADC assay.

**Results**

Patient-derived antibodies from several cancer types bound to human tumor tissue but not adjacent normal tissue and also internalized into A549 lung tumor cells. These internalizing antibodies were able to induce target cell death in vitro when conjugated directly or indirectly to a cytotoxic agent across several human tumor cell lines.

**Conclusions**

In this study we demonstrate that patient-derived antibodies which bind to public tumor-selective antigens and internalize into cancer cells can be identified by our IRC™ technology. Furthermore, we demonstrate that these antibodies can deliver a cytotoxic payload to target tumor cells to induce cell death.

**Ethics Approval**

The study was approved by Sutter Health Institutional Review Board, approval #2016.148-1

#### P2 Intratumoral application of hu14.18-IL2 for treatment of GD2+ pediatric malignancies: A novel immunotherapeutic approach aiming at in-situ vaccination

##### Romana Gugenberger, PhD^1^, Zachary Morris, MD, PhD^2^, Oliver Mutschlechner^1^, Paul Sondel, MD, PhD^2^, Hans Loibner, PhD^1^

###### ^1^Apeiron Biologics AG, Vienna, Austria; ^2^University of Wisconsin, Madison, WI, USA

####### **Correspondence:** Hans Loibner (hans.loibner@apeiron-biologics.com)

**Background**

hu14.18-IL2 is an antibody-cytokine fusion protein that combines targeting and immune activation of a human IgG1 monoclonal antibody with the immune stimulatory function of IL2. The humanized antibody portion targets the GD2 ganglioside antigen expressed on a variety of tumors of neuroectodermal origin. Clinical efficacy of the immunocytokine by i.v. application has been shown already in several clinical trials in melanoma and neuroblastoma. Dose limiting toxicity relates to systemic IL2 toxicity. A novel approach was explored preclinically in murine tumor models to deliver hu14.18-IL2 locally by intratumoral (IT) injection aiming at induction of a systemic immune response (in-situ vaccination). We present here activity of the immunocytokine in vitro against various GD2 positive pediatric tumor cell lines. We also discuss a humanized mouse model based on patient-derived xenografts (PDX) by directly transplanting surgical material. Finally we will present the design of a clinical trial to explore safety and clinical activity of IT hu14.18-IL2 in patients with GD2+ pediatric malignancies.

**Methods**

Expression of the target antigen GD2 on human cell lines MG63 (osteosarcoma), TC-71 (Ewing’s sarcoma), RH41 (rhabdomyosarcoma) and Y79 (retinoblastoma) was analyzed by flow cytometry. Hu14.18-IL2 mediated ADCC and whole blood cytotoxicity (WBT) was determined by 51Cr release assays.

**Results**

We found expression of antigen GD2 on all cell lines derived from neuro-ectodermal pediatric malignancies. Hu14.18-IL2 was effective in mediating ADCC and WBT against all cell lines in vitro, and potency was found higher than that of the unconjugated chimeric anti-GD2 antibody ch14.18/CHO in osteosarcoma and retinoblastoma. The effects were antigen specific as addition of an anti-idiotypic antibody abrogated the cytolytic activity. A humanized mouse model (CD34+ cell engraftment and transplantation of patient derived GD2+ sarcoma tissue) with intra-tumoral application of the immunocytokine is presently set up.

**Conclusions**

Immunocytokine hu14.18-IL2 is effective in vitro against various GD2 positive pediatric malignancies by activation of both antibody and IL2 effector functions. Humanized mouse tumor models with GD2+ patient derived tumors may be useful to explore IT immunocytokine in vivo. A clinical phase I/II trial in several advanced pediatric GD2 positive tumors (mostly sarcomas; “basket study”) is in preparation with repeated IT administration of low doses of hu14.18-IL2 (in-situ vaccination).

#### P3 Evaluating antibody-mediated cellular cytotoxicity and potency of antibody-drug conjugates within three- dimensional tumor models

##### Chris Langsdorf, BS, Bhaskar Mandavilli, PhD, Yi-Zhen Hu, Aimei Chen, Bachelor of Science, Marcy Wickett

###### ThermoFisher Scientific, Eugene, OR, USA

####### **Correspondence:** Chris Langsdorf (chris.langsdorf@thermofisher.com)

**Background**

Three dimensional tumor spheroids provide biochemical conditions that closely resemble the tumor microenvironment in an intact organism. Noninvasive approaches such as fluorescence microscopy are highly advantageous as they allow for the study of these three dimensional systems. Here we investigate the penetration and potency of natural killer cells, cytotoxic T cells, and antibody-drug conjugates in three-dimensional models of breast and lung cancer.

**Methods**

Tumor spheroids were formed by incubating cancer cell lines overnight in Nunclon Sphera 96-well plates. Natural killer cells were isolated from human PBMCs using negative magnetic selection and expanded in culture for 16 days. Natural killer cells were added to SKBR3 breast cancer spheroids with or without trastuzumab. T cells were isolated from human PBMCs using negative magnetic selection and activated for 72 hours. Activated or resting T cells were added to lung cancer spheroids. Immune cell penetration and tumor cytotoxicity were evaluated using whole-spheroid imaging on a confocal high-content imaging system. Trastuzumab was site-specifically conjugated with monomethyl auristatin E (MMAE) and iFL pHrodo Red via SiteClick conjugation. Spheroids of HER2+ breast cancer cells were treated 48 hours with this antibody drug conjugate. ADC penetration and apoptosis were evaluated using confocal high-content imaging.

**Results**

Unstimulated T cells produced minimal cytotoxicity, similar to untreated spheroids. Activated T cells penetrated and produced significant cytotoxicity throughout cancer spheroids. SKBR3 breast cancer cells form a compact, viable spheroid. Addition of NK cells leads to moderate cytotoxicity, while addition of NK cells and trastuzumab results in substantial cytotoxicity and degradation of spheroid structure (Fig. 1). Trastuzumab labeled with iFL pHrodo Red becomes brightly fluorescent following specific endosomal internalization into breast cancer cells, but minimal toxicity is observed. Trastuzumab conjugated with both iFL pHrodo Red and MMAE internalizes into cells and results in cell killing (Fig. 2).

**Conclusions**

Fluorescence microscopy combined with novel cell and antibody labeling methods permits investigation of the penetration and potency of natural killer cells, cytotoxic T cells, and antibody-drug conjugates in three-dimensional solid tumor models.

**Fig. 1 (abstract P3).**
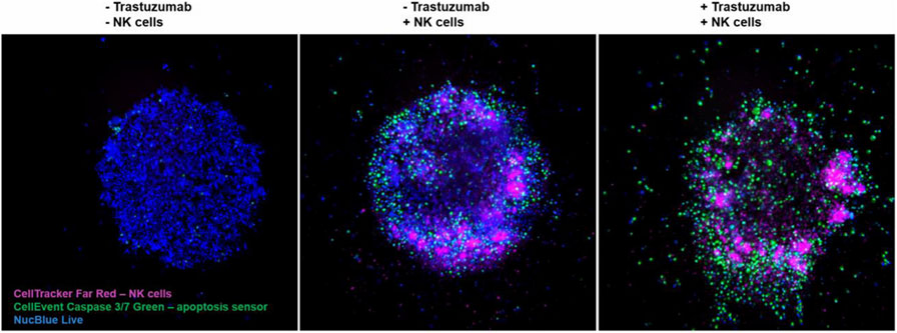
See text for description.

**Fig. 2 (abstract P3).**
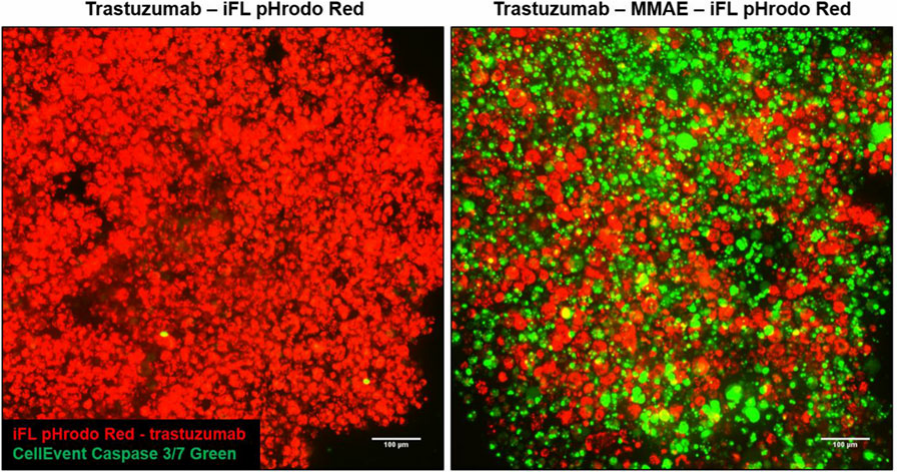
See text for description.

#### P4 PBD-based anti-MICA/B antibody drug conjugate with a dual mechanism of action: direct tumor cell killing and restoration of NKG2D-mediated immunosurveillance

##### Florence LHOSPICE, Pharm D^1^, Laurent Pouyet, PhD^2^, Ester Morgado^2^, Romain Remark, PhD^1^, Delphine Bregeon^1^, Adeline Montbel^1^, Nadia Anceriz^1^, Mathieu Blery, PhD^1^, Ariane Morel, PhD^1^, Manel Kraiem^1^, Kenneth Crook^1^, Eric Vivier^1^, Yannis Morel, PhD^1^

###### ^1^Innate Pharma, Marseille, France; ^2^MI-mAbs, Marseille, France

####### Eric Vivier (eric.vivier@innate-pharma.fr)

**Background**

MICA and MICB can be expressed at the surface of a wide variety of tumor cells upon stress, while having a very limited expression on healthy tissues. This makes MICA/B promising targets for the development of antibody drug conjugates (ADC). In addition, MICA and MICB serve as ligands for NKG2D, a potent activating receptor expressed on NK, CD8+T and γδ T cells. As a consequence, the expression of MICA and MICB promotes recognition and elimination of tumors by these lymphocytes through NKG2D engagement. However, in vitro and in vivo studies have reported that chronic engagement of NKG2D by its ligands induces NKG2D downregulation and lymphocyte dysfunction, leading to compromised immunity. We thus aimed to generate an ADC targeting MICA/B- expressing tumors with a dual function to achieve optimal therapeutic benefits: (i) killing of tumor cells and (ii) disrupting the interaction between MICA/B and NKG2D that induces impaired immunosurveillance [1] [2].

**Methods**

Antibodies were screened based on their binding affinity for the most frequent MICA/B alleles, as well as for their internalization, cytotoxicity and immunomodulatory properties. The MICA/MICB cross-reactive, pan-allele antibody with the highest affinity was conjugated to valine-alanine-pyrrolobenzodiazepine (PBD) dimers using bacterial transglutaminase-based site-specific conjugation to generate anti-MICA/B-PBD ADC

**Results**

Anti-MICA/B-PBD showed potent in vitro cytotoxicity against a variety of solid cancer cell lines as well as in vivo efficacy in both HCT116 human colon carcinoma and breast cancer patient-derived xenograft models. The immunomodulatory properties of anti-MICA/B-PBD were assessed in MICA-transgenic mice engrafted with MICA- expressing mouse melanoma B16-F10. Lastly, cell surface MICA/B expression and soluble form concentration were assessed in a large panel of samples from healthy donors and patients with various cancers in order to determine potential therapeutic indications for clinical development.

**Conclusions**

MICA/B molecules are attractive targets for an ADC approach based on their selective expression in a wide range of malignancies while showing restricted expression in healthy tissues along with manageable concentrations of their soluble form. The anti-MICA/B-PBD shows efficacy both in vitro and in vivo, paving the path for further evaluation towards clinical development.

**References**

1. Wiemann K, Mittrucker HW, Feger U, Welte SA, Yokoyama WM, Spies T, et al. Systemic NKG2D down-regulation impairs NK and CD8 T cell responses in vivo. J Immunol. 2005;175(2):720-9. PubMed PMID: 16002667.

2. Blery M, Vivier E. NKG2D-MICA Interaction: A Paradigm Shift in Innate Recognition. J Immunol. 2018;200(7):2229-30. Epub 2018/03/21. doi: 10.4049/jimmunol.1800176. PubMed PMID: 29555675.

#### P5 Anti-CD38 immunotherapy kills Treg (CD4+CD25+FoxP3+CD38hi) and Breg (CD19+CD24+CD38hi) cells and restores the anti-tumor T-cell repertoire in chronic lymphocytic leukemia (CLL)

##### Alak Manna, PhD, Sonikpreet Aulukh, MD, Laura Lewis-Tuffin, PhD, Taimur Sher, MD, Sikander Ailawadhi, MD, Rami Manuchakian, MD, Asher A. Chanan-Khan, Aneel Paulus, MD

###### Mayo Clinic, Jacksonville, FL, USA

####### **Correspondence:** Asher A. Chanan-Khan (Chanan-Khan.Asher@mayo.edu)

**Background**

CLL is the most common adult B-cell leukemia in western hemisphere. A subset of CLL cells immunophenotypically resembles B-regulatory cells (Bregs) and produce IL-10 and TGFβ that functionally imparts to them tumor-supportive properties. These cells are known to support and maintain T-regulatory (Treg) cells. Together, CLL Bregs and Tregs suppress CD8+ cytotoxic T-cell (cTLs) fostering an immunosuppressive and tumor promoting milieu that contribute to disease progression. We observed that a large proportion of CLL-Bregs and Tregs have a high CD38 receptor expression. This led us to hypothesize that eliminating them can potentially restore anti-tumor immune-effector response and is possible through anti-CD38 immunotherapy.

**Methods**

Blood peripheral mononuclear cells (PBMCs) were isolated from patients with a confirmed diagnosis of CLL (n=17) or healthy donors (n=6, control) under a protocol approved by the Mayo Clinic IRB. Characterization of CLL B-cells (CD19+CD5+), CLL-Bregs (CD19+CD24+CD38+IL10+), Tregs (CD4+CD25+CD127dimFoxP3+) was performed by flow-cytometry. Anti-CD38 immunotherapeutic, Daratumumab (Dara) was used in experiments. Intracellular IL-10 and FoxP3 were measured via fix/perm protocol followed by cytokine staining. Naïve T-cell to Treg transformation was determined using a trans-well co-culture assay. Extracellular IFNγ and IL-10 were measured via ELISA. Apoptosis was determined using annexin-V/PI staining. cTL proliferation was assessed via CFSE labeling of CD8+ sorted T-cells. For in vivo studies, a CLL patient-derived-xerograph (PDX) mouse model was established. (Figure 1)

**Results**

We noted that compared to healthy donors, CLL patients had a significantly higher % of Tregs (55.23±6.85%) and these Tregs had high CD38 expression (MFI=616.8±36.27). Consistent with our hypothesis, ex-vivo treatment of CLL patient PBMCs with Dara (1ug/mL) was highly lethal to CD38hi Bregs and Tregs. We also noted that CD38hi CLL-Bregs promoted transformation of naïve CD4+ T cells into Tregs in an IL-10/TGFβ dependent manner and neutralization of IL-10/TGFβ prevented this process. Notably, Dara treatment of naïve CD4+ T cells elicited same effect. Overall, Dara induced CLL cell death via ADCC, CDC, ADCP and mitochondrial/FcγR-mediated apoptosis. In Dara-treated CLL PBMC+T-cell co-cultures, ex vivo, we observed decreased IL-10 but increased IFN-y, Th17 and cTL counts. Similarly, in the PDX model, Dara-treated mice showed an increase in CD8+ and Th17 cells but a decrease in Bregs and Tregs.

**Conclusions**

Anti-CD38 immunotherapy is lethal to immunosuppressive CD38hi Breg/Treg cells and may improve anti-tumor T-cells function via modulating CLL immune-microenvironment. The results of these analyses have led to the approval of a phase-II clinical study that will be testing Dara in relapsed/refractory CLL patients.

**Acknowledgements**

Acknowledgement: Daniel Foundation of Alabama and Mayo Clinic Cancer Center (CA015083; A.A.C-K.), University of Iowa and Mayo Clinic Lymphoma SPORE Developmental Research Program (P50 CA097274; A.P.) and the Predolin Foundation (A.A.C-K).

**Ethics Approval**

Ethics Approval: The study was approved by Mayo Clinic Institutional Research Board (IRB# 14-009163)

**Fig. 1 (abstract P5).**
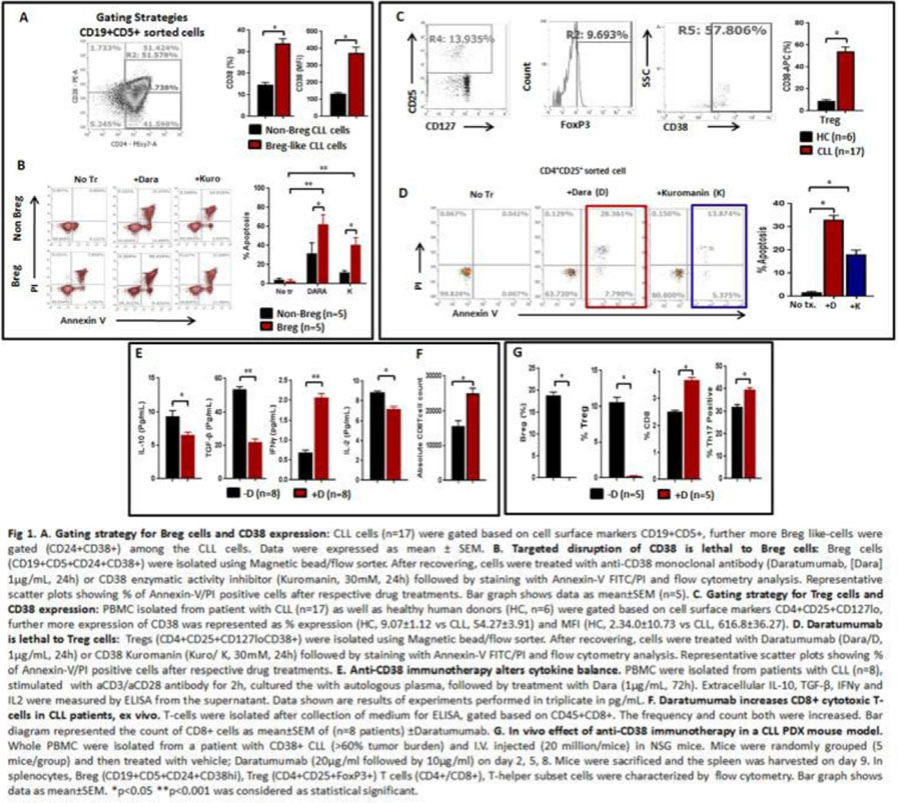
Anti-CD38 immunotherapy kills Treg and Breg

#### P6 Anti-CD38 immunotherapy kills Treg (CD4+CD25+FoxP3+CD38hi) and Breg (CD19+CD24+CD38hi) cells and restores the anti-tumor T-cell repertoire in chronic lymphocytic leukemia (CLL)

##### Alak Manna, PhD, Sonikpreet Aulakh, MD, Laura Lewis-Tuffin, PhD, Taimur Sher, MD, Sikander Ailawadhi, MD, Rami Manuchakian, MD, Asher A. Chanan-Khan, Aneel Paulus, MD

###### Mayo Clinic, Jacksonville, FL, USA

####### **Correspondence:** Asher A. Chanan-Khan (Chanan-Khan.Asher@mayo.edu)

**Background**

CLL is the most common adult B-cell leukemia in western hemisphere. A subset of CLL cells immunophenotypically resembles B-regulatory cells (Bregs) and produce IL-10 and TGFβ that functionally imparts to them tumor-supportive properties. These cells are known to support and maintain T-regulatory (Treg) cells. Together, CLL Bregs and Tregs suppress CD8+ cytotoxic T-cell (cTLs) fostering an immunosuppressive and tumor promoting milieu that contribute to disease progression. We observed that a large proportion of CLL-Bregs and Tregs have a high CD38 receptor expression. This led us to hypothesize that eliminating them can potentially restore anti-tumor immune-effector response and is possible through anti-CD38 immunotherapy.

**Methods**

Blood peripheral mononuclear cells (PBMCs) were isolated from patients with a confirmed diagnosis of CLL (n=17) or healthy donors (n=6, control) under a protocol approved by the Mayo Clinic IRB. Characterization of CLL B-cells (CD19+CD5+), CLL-Bregs (CD19+CD24+CD38+IL10+), Tregs (CD4+CD25+CD127dimFoxP3+) was performed by flow-cytometry. Anti-CD38 immunotherapeutic, Daratumumab (Dara) was used in experiments. Intracellular IL-10 and FoxP3 were measured via fix/perm protocol followed by cytokine staining. Naïve T-cell to Treg transformation was determined using a trans-well co-culture assay. Extracellular IFNγ and IL-10 were measured via ELISA. Apoptosis was determined using annexin-V/PI staining. cTL proliferation was assessed via CFSE labeling of CD8+ sorted T-cells. For in vivo studies, a CLL patient-derived-xerograph (PDX) mouse model was established.

**Results**

We noted that compared to healthy donors, CLL patients had a significantly higher % of Tregs (55.23±6.85%) and these Tregs had high CD38 expression (MFI=616.8±36.27). Consistent with our hypothesis, ex-vivo treatment of CLL patient PBMCs with Dara (1ug/mL) was highly lethal to CD38hi Bregs and Tregs (Figure 1). We also noted that CD38hi CLL-Bregs promoted transformation of naïve CD4+ T cells into Tregs in an IL-10/TGFβ dependent manner and neutralization of IL-10/TGFβ prevented this process. Notably, Dara treatment of naïve CD4+ T cells elicited same effect. Overall, Dara induced CLL cell death via ADCC, CDC, ADCP and mitochondrial/FcγR-mediated apoptosis. In Dara-treated CLL PBMC+T-cell co-cultures, ex vivo, we observed decreased IL-10 but increased IFN-y, Th17 and cTL counts. Similarly, in the PDX model, Dara-treated mice showed an increase in CD8+ and Th17 cells but a decrease in Bregs and Tregs.

**Conclusions**

Anti-CD38 immunotherapy is lethal to immunosuppressive CD38hi Breg/Treg cells and may improve anti-tumor T-cells function via modulating CLL immune-microenvironment. The results of these analyses have led to the approval of a phase-II clinical study that will be testing Dara in relapsed/refractory CLL patients.

**Acknowledgements**

Acknowledgement: Daniel Foundation of Alabama and Mayo Clinic Cancer Center (CA015083; A.A.C-K.), University of Iowa and Mayo Clinic Lymphoma SPORE Developmental Research Program (P50 CA097274; A.P.) and the Predolin Foundation (A.A.C-K).

**Ethics Approval**

Ethics Approval: The study was approved by Mayo Clinic Institutional Research Board (IRB# 14-009163)

**Fig. 1 (abstract P6).**
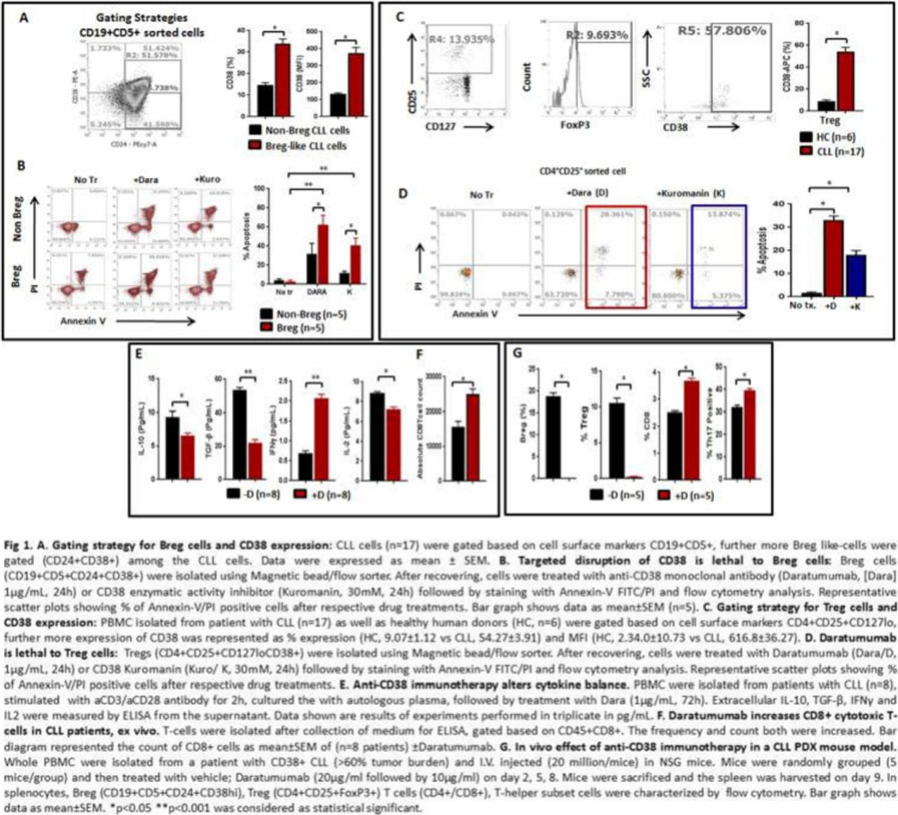
Anti-CD38 immunotherapy kills Treg and Breg

#### P7 Potent tumor-directed T cell activation and tumor inhibition induced by a 4-1BB x 5T4 ADAPTIR™ bispecific antibody

##### Michelle Nelson, PhD^1^, Gabriele Blahnik-Fagan^1^, Robert Bader^1^, Doreen Werchau, BS^2^, Anneli Nilsson^2^, Lill Ljung^2^, Jeannette Bannink, BS^1^, Danielle Mitchell^1^, Lynda Misher^1^, Catherine McMahan^1^, Maria Askmyr^2^, Anna Dahlman^3^, Peter Ellmark, PhD^2^, Gabriela Hernandez-Hoyos^1^, Sara Fritzell^2,4^

###### ^1^Aptevo Therapeutics Inc., Seattle, WA, USA; ^2^Alligator Bioscience AB, Lund, Sweden; ^3^Allligator Bioscience AB, Lund, Sweden; ^4^Alligator Bioscience, Lund, Sweden

####### **Correspondence:** Gabriela Hernandez-Hoyos (ghoyos@apvo.com)

**Background**

4-1BB (CD137) is an important activation-induced co-stimulatory receptor that regulates immune responses of activated CD8+ T and NK cells, by enhancing proliferation, survival, cytolytic activity and IFN-γ production. The ability to induce potent anti-tumor activity by stimulating 4-1BB on tumor-specific cytotoxic T cells makes 4-1BB an attractive target for designing novel therapeutics for immuno-oncology. However, clinical development of a monospecific 4-1BB agonistic antibody has been hampered by dose-limiting hepatic toxicities. To minimize systemic immune toxicities, we have developed a novel 4-1BB x 5T4 bispecific antibody designed to direct tumor- specific T cell responses to the tumor microenvironment by stimulating 4-1BB function only when co-engaged with 5T4, a tumor-associated antigen.

**Methods**

ALG.APV-527 was built based the ADAPTIR™ platform with binding domains to 4-1BB and 5T4 generated using the ALLIGATOR-GOLD® human scFv library and subsequently optimized to increase binding affinity, function, stability and manufacturability. To assess its agonistic function, ALG.APV-527 was tested in NF-κB luciferase reporter systems and assays using primary cells in the presence or absence of cells expressing 5T4. To stimulate primary cells, enriched CD8+ T cells or unseparated PBMC were sub-optimally cultured with anti-CD3 antibody. Secretion of IFN-γ was measured at 72 hrs using ELISA or Luminex-based assays. To measure proliferation, PBMC were labelled with Cell TraceTM and CD8+ T cells were gated using multicolor flow cytometry. For tumor inhibition studies, the human colon carcinoma HCT116 xenograft model expressing endogenous levels of 5T4 was used. 5T4 expression was evaluated in normal human tissues and a range of different human tumors by immunohistochemistry (IHC).

**Results**

In vitro, ALG.APV-527 triggers luciferase reporter activity in the presence of 5T4-expressing cells. Using enriched T cells or whole unseparated PBMC, ALG.APV-527 induces a concentration-dependent increase of IFN-γ production when co-cultured with 5T4-expressing cells. ALG.APV-527 enhances primary CD8+ T cell proliferation preferentially over CD4+ T cells. Of significance, ALG.APV-527 is capable of inhibiting tumor growth in a human colon carcinoma xenograft model. IHC staining confirms that 5T4 is overexpressed in a range of solid tumors but not in normal tissues, indicating that ALG.APV-527 may primarily localize to the tumor improving potential for a achieving concentrations that demonstrate efficacy in a solid tumor setting.

**Conclusions**

ALG.APV-527 induces potent CD8+ T cell co-stimulation but only in the presence of 5T4 antigen. Based on preclinical data, ALG.APV-527 is a promising anti-cancer therapeutic for the treatment of a variety of 5T4- expressing solid tumors.

#### P8 Single-cell proteomic analysis of T cells stimulated by Bi-specific T-cell Engagers (BiTEs) shows robust and unique polyfunctional secretion profile

##### Sean Mackay, MBA^1^, Patrick Paczkowski^1^, Brianna Flynn, MS^1^, Kevin Morse^1^, Tiffany Coupet, BS^2^, Claire Godbersen, BS^2^, Charles Sentman^2^, Jing Zhou, MD, PhD^1^

###### ^1^IsoPlexis, Branford, CT, USA; ^2^Geisel School of Medicine at Dartmouth, Lebanon, NH, USA

####### **Correspondence:** Jing Zhou (jing@isoplexis.com)

**Background**

T cell cytokines can drive anti-tumor activity and greater polyfunctionality (co-secretion of 2+ proteins per single cell) has been shown to be associated with improved clinical outcome in the study of CAR-T cell therapy and vaccine. We employed single-cell proteomics to fully evaluate the impact of BiTEs on polyfunctional T cells. BiTEs as cancer-targeting drugs link T cells with tumor by binding CD3 and a tumor antigen. Natural killer group 2, member D (NKG2D) ligand, such as MICA, expressed on more than 90% of human tumors but limited on normal tissues has emerged as appealing targets for BiTEs. This study has explored the polyfunctional profile of T cells by two BiTEs: B2-OKT3 (MICA x CD3) and hNKG2D-OKT3 (NKG2D ligands x CD3).

**Methods**

Blood T cells were negatively enriched from 3 healthy donors and incubated with K562 cells at a ratio of 1:2 in the presence of 250 ng/ml of B2-OKT3, hNKG2D-OKT3 or control Tz47-2C11. After 36 hours stimulation at 37°C, 5% CO2, CD4+ and CD8+ T cells were separated with anti-CD4 or anti-CD8 microbeads. Approximately 30,000 cells were loaded on the IsoCode single-cell chip (SCBC), pre-patterned with a 32-plex antibody ELISA array per cellular microchamber. Secreted proteins were captured from ~1500 single T cells after 16-hour-on-chip incubation at 37°C, 5% CO2. The T cell polyfunctional profile was evaluated across 5 functional groups: effector (Granzyme B, IFN-γ, MIP-1α, Perforin, TNF-α, TNF-β), stimulatory (GM-CSF, IL-2, IL-5, IL-7, IL-8, IL-9, IL-12, IL-15, IL-21), regulatory (IL-4, IL-10, IL-13, IL-22, TGF-β1, sCD40L, sCD137), inflammatory (IL-1β, IL-6, IL-17A, IL-17F, MCP-1, MCP-4), and chemoattractive (CCL-11, IP-10, MIP-1β, RANTES).

**Results**

Both B2-OKT3 and hNKG2D-OKT3 BiTEs enhanced single-cell polyfunctionality and polyfunctional strength index (PSI) of both CD4+ and CD8+ T cells when activated by K562 tumor cells compared to the Tz47-2C11 negative control (Figure 1). The polyfunctional response was mainly driven by effector cytokines, including Granzyme B, IFN-γ, and MIP-1α, chemoattractive MIP-1β, and regulatory sCD137. hNKG2D-OKT3 elicited more robust polyfunctional response of both CD4+ and CD8+ T cells to K562 cells stimulation than B2-OKT3. Detailed polyfunctional cell subsets with unique cytokine signatures induced by each BiTE are elucidated through high- dimensional single-cell visualizations of the data.

**Conclusions**

Single-cell proteomic analysis reveals a significantly upregulated polyfunctional profile of T cells induced by the BiTEs against tumor cells than the negative control, providing important insights into BiTE-triggered T cell activity as well as better evaluation and understanding of BiTE therapies.

**Fig. 1 (abstract P8).**
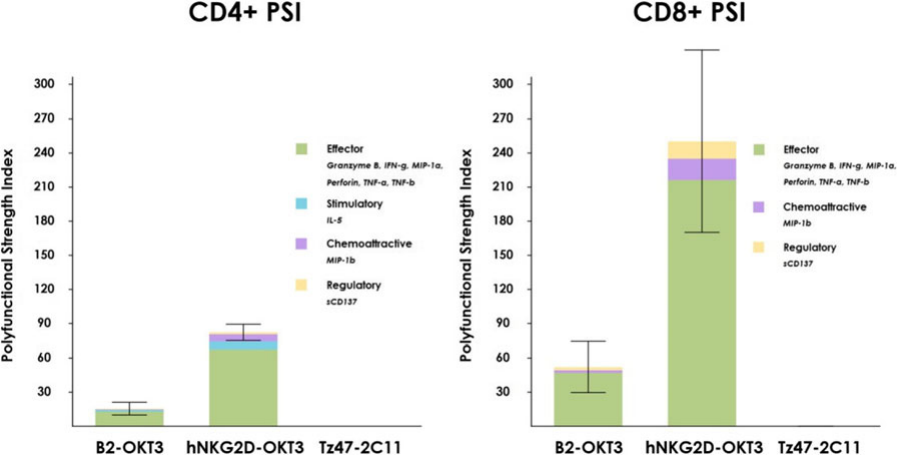
T Cell Polyfunctional Strength Enhanced by BiTEs

#### P9 Identification and functional profiling of PD-L1 targeted engineered toxin bodies for antigen seeding technology and redirection of T cell response to tumors

##### Brigitte Brieschke, BS, Sara LeMar, Garrett Robinson, Aimee Iberg, PhD, Shaoyou Chu, PhD, Jack Higgins, PhD, Erin Willert, PhD, Hilario Ramos, PhD

###### Molecular Templates, Austin, TX, USA

####### **Correspondence:** Hilario Ramos (hilario.ramos@mtem.com)

**Background**

Molecular Templates’ Engineered Toxin Body (ETB) platform comprises recombinant immunotoxins leveraging Shiga-like Toxin A subunit (SLTA) properties of self-internalization, predictable retrograde transport, and lethal ribosomal inactivation with antibody binding domains to create targeted biologics capable of potent and specific direct killing of cancerous cells (MOA-1). Antigen Seeding Technology (AST) addition to the ETB scaffold provides a novel approach for redirection of preexisting memory cytotoxic T lymphocytes (CTLs) to cancerous cells (MOA-2). Both MOAs are designed to be functional in patients previously treated with standard of care agents. Here we describe the development of PD-L1 targeted ETBs with AST functionality capable of promoting cytolytic activity by CTLs recognizing a common Cytomegalovirus (CMV) viral antigen (HLA:A02 restricted CMV-pp65- NLVPVMATV, A2-pp65) on targeted tumor cells. We further describe the characteristics that distinguish the complementary mechanisms of action.

**Methods**

ETBs comprising SLTA fused to PD-L1 scFvs were engineered with or without A2-pp65 peptide. Human tumor cell lines expressing or lacking PD-L1 and HLA:A2 were used as target cells for cytotoxicity assays. Antigen restricted CTLs were expanded from human donors and used in co-culture models as effector cells.

**Results**

ETBs with potent direct cell kill activity have been identified to bind PD-L1 outside of or overlapping critical contact residues for PD-1. These ETBs have checkpoint inhibitor activity in a PD-1/PD-L1 blockade assay, though significantly less than their corresponding monoclonal antibody. A2-pp65 peptide fusion to ETBs resulted in cell- surface presentation of A2-pp65 peptide in complex with MHC-I and triggered efficient lysis by A2-pp65 specific CTLs in a target and HLA-restricted fashion. SLTA mutations which cause ER retention or do not inactivate ribosomes have no direct cell kill activity (remove MOA-1) but retain the antigen presentation and CTL directed lysis (maintain MOA-2). Additionally, genetic engineering identified modifications that could enhance MOA-1 activity without limiting MOA-2 activity, thus identifying molecules with optimal activity for clinical development. The predictable routing of ETBs support both MOAs and indicates a reduced threshold and routing requirement for MOA-2 as compared to MOA-1, allowing for broader cytotoxicity.

**Conclusions**

We have developed ETBs which bind distinct epitopes on PD-L1 and provide two unique and complementary mechanisms of action. Coupling both mechanisms of cytotoxicity into one molecule allows for potential to increase target penetrance, expand a prolonged immune response, and overcome resistance. In vivo syngeneic and xenograft studies are ongoing in preparation for clinical development of PD-L1 targeted ETBs with AST functionality in 2019.

#### P10 Local radiation with intratumoral anti-disialoganglioside (anti-GD2) and interleukin-2 (IL2) induces significant tumor responses with immunologic memory in a syngeneic murine NXS2 neuroblastoma model

##### Julie Voeller, MD^1^, Amy Erbe, PhD1, Kayla Rasmussen, MS^1^, Jacob Slowinski^1^, Sabrina VandenHeuvel^1^, Ravi Patel, MD, PhD^1^, Hans Loibner, PhD^2^, Stephen Gillies, PhD^3^, Jacquelyn Hank, PhD^1^, Alexander Rakhmilevich, MD, PhD^1^, Zachary Morris, MD, PhD^1^, Paul Sondel, MD, PhD^1^

###### ^1^University of Wisconsin Madison, Madison, WI, USA; ^2^HL Bioscience Research GmbH, Vienna, Austria; ^3^Provenance Biopharmaceuticals, Carlisle, MA, USA

####### **Correspondence:** Julie Voeller (jvoeller@uwhealth.org)

**Background**

Neuroblastoma is the most common extracranial solid tumor in pediatrics. Standard therapy for patients with high-risk disease includes anti-disialoganglioside (anti-GD2) monoclonal antibody (mAb), GM-CSF (granulocyte- macrophage colony stimulating factor), and IL2 (interleukin-2)—an immunotherapeutic regimen that has significantly improved survival rates. Our lab has previously shown that local radiation therapy (RT) combined with intratumoral (IT) immunocytokine (IC; a fusion of hu14.18 anti-GD2 mAb and IL2) can cure mice with melanoma. We aimed to test and optimize this in situ vaccine approach in a murine neuroblastoma model.

**Methods**

Using the murine NXS2 neuroblastoma cell line, subcutaneous neuroblastoma tumors were established on the dorsal right flank of syngeneic A/J mice. Mice bearing 155mm3 tumors (engrafted about 2 weeks prior) received no RT or 12Gy RT to the tumor on treatment day 1, followed by daily intratumoral injections of either 50μg IC or PBS on treatment days 6-10. All mice with complete response were rechallenged on treatment day 90 by injecting NXS2 cells into the dorsal left flank.

**Results**

We observed improved tumor control (Figure A) and animal survival (Figure B; p<0.0001) when animals were treated with a combination of RT and IT-IC. Complete tumor regression was observed in 75% (9/12) of animals receiving RT and IT-IC, with 89% (8/9) of these rejecting rechallenge. Of all the other groups, only 9% (1/11) of animals receiving IT-IC alone and 33% (4/12) of animals receiving 12Gy and PBS had complete tumor regression.

**Conclusions**

Combined treatment with RT and intratumoral IC cures most mice bearing a single, 155mm3 NXS2 neuroblastoma tumor and induces immunologic memory. Our ongoing studies continue to investigate this immunotherapy regimen to test its effectiveness in more advanced disease as well as in other in vivo syngeneic murine neuroblastoma model systems.

**Fig. 1 (abstract P10).**
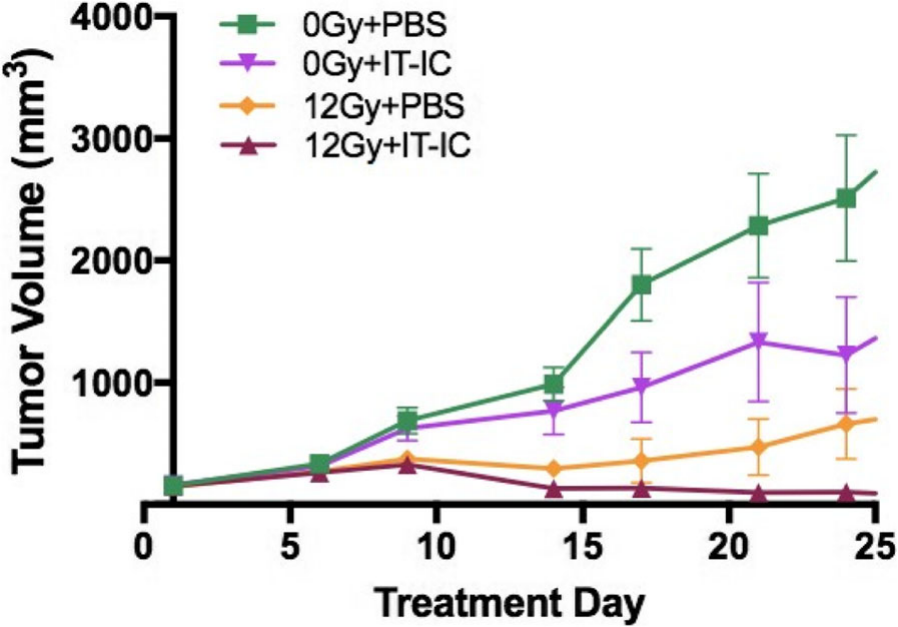
See text for description.

**Fig. 2 (abstract P10).**
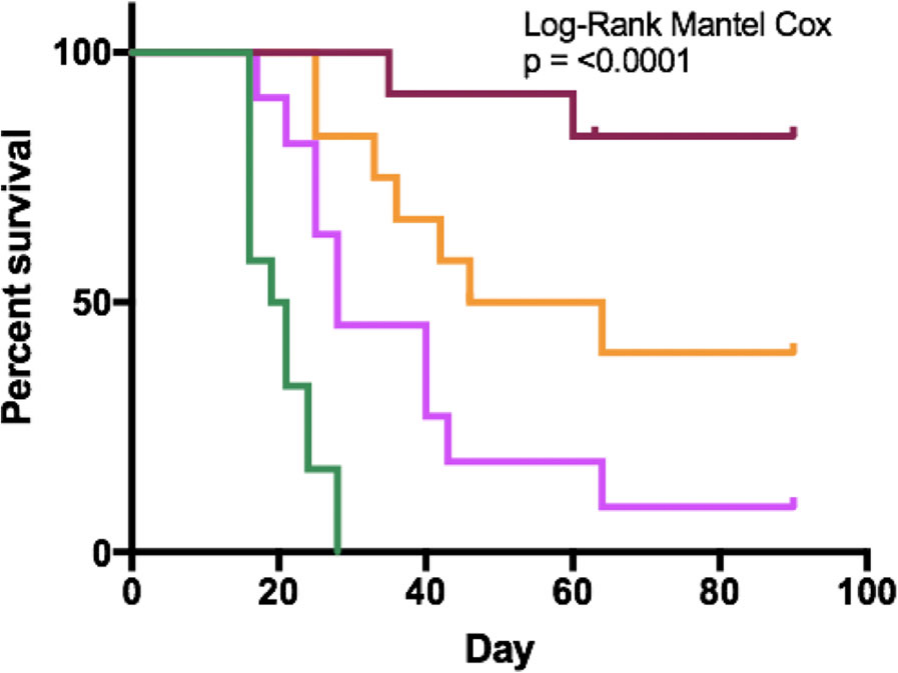
See text for description.

#### P11 A CD25-targeted pyrrolobenzodiazepine dimer-based antibody-drug conjugate shows potent anti-tumor activity in pre-clinical models of solid tumors either alone or in combination with a PD-1 inhibitor

##### Francesca Zammarchi, PhD^1^, Karin Havenith, PhD^1^, Francois Bertelli^2^, Balakumar Vijayakrishnan^2^, Patrick van Berkel, PhD^1^

###### ^1^ADC Therapeutics, London, UK; ^2^Spirogen/MedImmune, London, UK

####### **Correspondence:** Francesca Zammarchi (francesca.zammarchi@adctherapeutics.com)

**Background**

Regulatory T (Treg) cells infiltrate into various types of human cancers and contribute to the immunosuppressive tumor microenvironment [1]. The intratumoral balance between Tregs versus T effectors (Teffs) cells appears to impact the outcome of the immune system-mediated tumor eradication and numerous attempts are currently underway to reduce the CD25-expressing Tregs cells [2].

**Methods**

Sur301 is an antibody-drug conjugate (ADC) composed of PC61, a rat monoclonal antibody directed against mouse CD25, stochastically conjugated to a pyrrolobenzodiazepine (PBD) dimer via a protease-cleavable linker, with a drug-to-antibody ratio of 2.

**Results**

In vitro, sur301 demonstrated potent and specific cytotoxicity in a CD25-expressing mouse lymphoma cell line, while no specific cytotoxicity was observed in two CD25-negative murine colon cancer-derived cell lines, MC38 and CT26. All three cell lines were highly sensitive to SG3199, the PBD dimer toxin of sur301, irrespective of their CD25-status. In vivo, sur301 anti-tumor activity was investigated in the syngeneic MC38 and CT26 models, two immunogenic colon cancer models with tumor-infiltrating CD25-positive Treg cells [3]. Sur301 was administered either alone (0.1, 0.5 or 1 mg/kg, single dose) or in combination with an anti-PD1 antibody. A non-binding control ADC was used as negative control and tested as single dose at 1 mg/kg, either alone or in combination with an anti- PD1 antibody. Single doses of sur301 at 0.5 or 1 mg/kg induced strong and durable anti-tumor activity in both models. When tumor free survivor (TFS) animals were re-challenged with MC38 or CT26 cells, no mice developed new tumors, demonstrating tumor-specific protective immunity was generated by the single action of sur301. A single sub-optimal dose of sur301 at 0.1 mg/kg elicited limited anti-tumor activity when tested as single agent, but combination with an anti-PD1 antibody resulted in synergistic anti-tumor activity in both models. Combination of a single dose of sur301 at 0.5 or 1 mg/kg with the anti-PD1 antibody further increased the number of responders and none of the re-challenged animals developed new tumors. The non-binding control ADC showed no or significant reduced activity when tested at the same dose as sur301.

**Conclusions**

In conclusion, sur301 demonstrated potent in vivo activity against CD25-negative immunogenic solid tumors with infiltrating CD25-positive Treg cells. Sur301 in vivo activity was further enhanced by combination with anti-PD1 antibody. These data warrant further investigation of ADCT-301, a PBD-based ADC targeting human CD25, in patients with solid tumors, either alone or in combination with checkpoint inhibitors.

**References**

1. Sasidharan Nair V, Elkord E. Immune checkpoint inhibitors in cancer therapy: a focus on T-regulatory cells. Immunol Cell Biol, 2018. 96(1): p. 21-33.

2. Menetrier-Caux C, et al. Targeting regulatory T cells. Target Oncol, 2012. 7(1): p. 15-28.

3. Arce Vargas F, et al. Fc-optimized anti-CD25 depletes tumor-infiltrating regulatory T cells and synergizes with PD-1 blockade to eradicate established tumors. Immunity, 2017. 46(4): p. 577-586.

**Ethics Approval**

All in vivo study work was approved by the Institutional Animal Care and Use Committee at Charles River Laboratories, Morrisville, N.C..

### Best Practices for Improving Cancer Immunotherapy

#### P12 Response and toxicity with immune checkpoint inhibition in older patients with non-small-cell lung cancer

##### Keval Yerigeri, BS^1^, Kristen Marrone, MD^2^, Jiajia Zhang, MD, MPH^2^, Julie Brahmer, MD^2^, Patrick Forde, MD^2^, Christine Hann, MD, PhD^2^, David Ettinger, MD^2^, Ronan Kelly, MD MBA^2^, Josephine Feliciano, MD^2^, Sarah Sagorsky, PA-C^2^, Michelle Turner, NP^2^, Valerie Rowe, NP^2^, Jarushka Naidoo, MD^2^

###### ^1^Northeast Ohio Medical University, Copley, OH, USA; ^2^Johns Hopkins University School of Medicine, Sidney Kimmel Comprehensive Cancer Center, Baltimore, MD, USA

####### **Correspondence:** Kristen Marrone (kmarron1@jhmi.edu)

**Background**

Immune checkpoint inhibition (ICI) has rapidly become standard of care in advanced or metastatic non-small-cell lung cancer (NSCLC) treatment. Initial phase III clinical trials suggest ICI may have decreased efficacy in NSCLC patients ≥ 75 years old. The relationship between age-related immune system changes and ICI treatment is poorly understood.

**Methods**

The Johns Hopkins Upper Aerodigestive Diseases Immunotherapy Database was queried for all patients ≥ 75 years old treated with anti-PD-1/PD-L1 agents as part of a clinical trial or standard of care, from 2007 to 2018.

**Results**

Thirty-one patients ≥ 75 years old receiving anti-PD-1/PD-L1 agents for locally advanced or metastatic NSCLC were identified. Eleven patients were female, median age was 80.8 years (range: 75.1-90.6) with median ECOG PS=1 (range: 0-3). Twenty-seven patients received PD-1/PD-L1 monotherapy (nivolumab=16, pembrolizumab=10, atezolizumab=1) and 4 received combination (+chemotherapy=1, +ipilimumab=2, +additional ICI=1). Ten patients received ICI in the first-line setting (1L); 21 patients in the second-line or beyond (2L+). In 1L ICI monotherapy (n=8), median doses received was 5.5 (range: 2-19), median progression-free survival (mPFS) was 7.3m, and median overall survival (mOS) was 11.3m. In 2L+ patients, median dose administration was 4 (range: 1-24), mPFS was 7m and mOS was 7.6m. Across 1L and 2L+ ICI monotherapy patients, a rate of 81.5% all-grade toxicity was seen, of which 30% were high-grade (3+). All 1L and 2L+ ICI combination patients (n=4) experienced a toxicity, with 3 patients experiencing high-grade events. Across all patients, the most common low-grade toxicities were fatigue (n=8) and dyspnea (n=8). High-grade pneumonitis was seen in two 1L ICI monotherapy patients; 2L+ ICI monotherapy high-grade toxicities included dyspnea (n=4), hypoxia (n=1; Grade 5), pneumonitis (n=1), chest pain (n=1), delirium (n=1), aspiration (n=1), heart failure (n=1), lymphadenopathy (n=1) and pleural infection (n=1). Combination ICI high grade toxicities included pneumonitis (1L=1; 2L+=1) and rash (2L+=1). Across patients, reasons for treatment discontinuation included progressive disease (31%), with double the patients stopping for toxicity (62%) and treatment ongoing for 2 patients.

**Conclusions**

Our results indicate increased frequency and severity of toxicity in anti-PD1/PD-L1 treated older NSCLC patients, with decreased time to off treatment compared to landmark phase III studies. Survival data comparisons are limited in the setting of the current small sample size, but show interesting trends of decreased time on therapy and decreased overall survival. Further translational evaluation of senescent remodeling’s role in outcome and toxicity with ICI in older NSCLC patients is needed.

**Acknowledgements**

We would like to thank the patients and their families who agreed to participate in the Johns Hopkins Upper Aerodigestive Diseases Immunotherapy Database.

**Ethics Approval**

The study was approved by the Johns Hopkins School of Medicine’s Ethics Board, IRB00087582.

### Biomarkers and Immune Monitoring

#### P13 Molecular profiling of anti-PD-1 treated melanoma patients reveals importance of assessing neoantigen burden and tumor escape mechanisms for clinical treatment

##### Charles Abbott, PhD^1^, Sean Boyle, PhD^1^, Eric Levy, PhD^1^, Rena McClory^1^, Sekwon Jang, MD^2^, Richard Chen^1^

###### ^1^Personalis, Menlo Park, CA, USA; ^2^Inova, Fairfax, VA, USA

####### **Correspondence:** Charles Abbott (cabbo003@ucr.edu)

**Background**

Despite the remarkable response of some melanoma patients to checkpoint inhibitor therapy, significant numbers of patients do not achieve complete response. It is of great interest to identify biomarkers and mechanisms that influence immunotherapy effectiveness. Here we apply a comprehensive tumor immuno-genomics platform (ACE ImmunoID) to identify potential biomarkers of response to checkpoint blockade therapy.

**Methods**

We characterized the immuno-genomics of tumors from 31 stage III/IV melanoma patients who have received anti-PD-1 treatments to assess potential factors influencing response. Tumor responses to the therapy were evaluated using RECIST criteria with a median follow-up of 12 months. Immuno-genomic profiling was performed using Personalis’ ACE ImmunoID platform: an augmented exome/transcriptome platform and analysis pipeline. Analysis included assessment of tumor mutations, neoantigen characterization, HLA typing, gene expression quantification, and tumor micro-environment profiling. The molecular information of the tumors was then analyzed together with their corresponding clinical response.

**Results**

We observed a trend that higher neoantigen burden was associated with better progression free survival. Further investigation of patients with high neoantigen burden that failed to achieve complete response (3 PD, 1 PR) revealed potential resistant mechanisms to anti-PD-1 therapy. Specifically, we identified two of these patients with high expression of IDO1 or CTLA4, which may facilitate immune escape in a PD-1 independent manner. Additionally, we found two patients with mutations in their antigen presentation machinery (APM). The first patient had two independent HLA mutations in HLA-A and HLA-B (stop-gain mutation and splice site mutation, respectively), leading to the likely loss of surface expression of two classes of HLA-A and HLA-B proteins. In the second APM mutation patient we observed a frameshift deletion event detected in B2M at a very high frequency (80% AF) in their tumor. These APM mutations suggest reduced neoantigen presentation in these patients, likely underlying mechanisms for tumor escape.

**Conclusions**

While we observed the expected association between neoantigen burden and response to checkpoint blockade therapy, we also identified potential resistance mechanisms in patients that involve perturbations to antigen presenting machinery and high expression of non-targeted checkpoint genes. This highlights the potential importance of broad immuno-genomic profiling of patients that are candidates for receiving immunotherapy. We are continuing to increase our cohort size to identify additional mechanisms for immune evasion.

**Ethics Approval**

IRB file # 16-2427

#### P14 Durvalumab treatment-induced transcriptional changes in the tumor microenvironment associated with longer survival in patients with late stage Non-Small Cell Lung Cancer (NSCLC)

##### Ikbel Achour, PhD, Zachary Cooper, PhD, Sriram Sridhar, Maria Ascierto, PhD, Jixin Wang, Young Lee, Natasha Angra, Shaad Abdullah, MD, FACP, Rajiv Raja, PhD, Brandon Higgs, PhD, Maria Jure-Kunkel

###### Medimmune, Gaithersburg, MD, USA

####### **Correspondence:** Maria Jure-Kunkel (jure-kunkelm@medimmune.com)

**Background**

Baseline biomarkers including PD-L1, IFNg signature and tumor mutational burden (TMB) have demonstrated clinical utility in predicting overall survival in patients treated with anti-PD(L)1 therapies. However, changes in the tumor microenvironment on checkpoint therapy and their relationship to survival are poorly understood. Here, we systematically analyzed tumor microenvironment transcriptional profiles before and on durvalumab treatment and explored association with survival in patients with NSCLC.

**Methods**

CP1108/NCT01693562 is a nonrandomized phase 1/2 trial evaluating durvalumab (10 mg/kg, Q2W) in patients with solid tumors including advanced NSCLC (squamous and non-squamous). RNA sequencing was performed on 97 baseline tumors and 29 paired baseline and on-treatment (6 weeks) tumors. Gene and pathway level analyses were performed in relation to overall survival at baseline (prolonged OS >2yrs, n=23, compared to short OS <1yrs, n=61) and following durvalumab treatment (n=11 in both OS groups); clinical data cut off was 10/16/2017

**Results**

Among 763 genes differentially expressed at baseline between tumors from patients with prolonged compared to short survival (FC≥ 1.5 ; p≤0.05), gene signatures of CD8, Th1, T-agonist, T-effector, B, Natural Killer cells (NK), M1 macrophages, dendritic cells (DC), chemoattractant chemokines, and IFNg were expressed at higher levels (FC≥2 ; p≤0.04). Following durvalumab-treatment, in both OS groups, CD8 and T-effector gene signatures were significantly increased (FC≥3 ; p≤0.04), and IFNg,Th1, M1 and chemoattractant chemokine gene signatures were moderately induced (FC≥1.7 ; p≤0.1). Baseline levels of Th2, M2 macrophages and MDSC gene signatures did not significantly change on durvalumab. T-agonist, B, NK and CD1c+ DC cell gene signatures were further induced (FC≥2 ; p≤0.05) only in patients with OS>2 yrs. High expression levels of T-agonist, B cell, and CD1c+ DC signatures correlated with improved OS in early stage non-squamous NSCLC in TCGA. On-treatment reduction of at least 2-fold (p≤0.04) in genes involved in angiogenic, metabolic, cell-cell adhesion and cell cycle pathways including WNT7B, VEGFA, FASN, EVPL and CDKN2B were observed specifically in patients with prolonged OS compared to no change in patients with short OS.

**Conclusions**

Durvalumab treatment resulted in substantial changes in gene expression in the tumor microenvironment, with notable increases in B and dendritic cell signatures associated to prolonged survival. Our results provide new insights into the anti-tumor mechanism of PD-L1 blockade.

**Trial Registration**

NCT01693562

#### P15 Development of a robust, simplified method to measure receptor occupancy in peripheral blood from patients treated with a novel anti-PD-1 agent, AB122

##### Devika Ashok, PhD, Dana Piovesan, MSc, Sharon Zhao, Hema Singh, Steve Young, PhD, Matthew Walters, PhD, Lisa Seitz, MSc

###### Arcus Bio, Hayward, CA, USA

####### **Correspondence:** Devika Ashok (dashok@arcusbio.com)

**Background**

Exhausted T cells express high levels of immune checkpoint proteins, including programmed cell death-1 (PD-1) receptor. Preclinical and clinical data support the role of PD-1 and its ligand, programmed cell death ligand 1 (PD- L1), in promoting tumor evasion by curtailing immune responses. In a Phase 1 clinical trial of the anti-PD-1 monoclonal antibody AB122, we determined receptor occupancy (RO) in peripheral blood T cells using a directly conjugated competitive antibody method. We contrasted the data quality and derived RO values to previously established methodology described for nivolumab using biotinylated anti-human IgG4.

**Methods**

RO assays were developed using healthy donor peripheral blood mononuclear cells (PBMCs) spiked with AB122. We evaluated parameters including specimen stability, fresh vs. frozen samples, wash conditions, reagent concentrations and adapted the protocol for application to whole blood specimens to eliminate the need for PBMC isolation. Multi-color flow cytometry enabled determination of RO as well as proliferation status in individual T cell subsets using Ki67 as a functional readout of the effect of anti-PD-1 therapy. We developed the RO panel to work in conjunction with an intra-nuclear staining protocol for Ki67. This included identification of optimal clones for surface staining, blocking non-specific staining and selection of a clone for Ki67 identification. Finally, we deployed both RO determination protocols to evaluate an initial set of samples from cancer patients enrolled in the ongoing dose escalation Phase 1 study of AB122.

**Results**

Comparable RO data were obtained using both the AB122 competitive antibody and saturation methodologies using biotinylated anti-human IgG4 in PBMC samples from study subjects. Across all initial subjects tested, including different dose groups and time points, an average of ≥ 90% RO was observed using either method. In addition, a greater than 2-fold increase in Ki67+ T cell subsets was observed in approximately half of the patients.

**Conclusions**

Data from our Phase 1 dose-escalation cohorts demonstrates complete RO across a range of dosing regimens of AB122 and is consistent with data published for other anti-PD-1 antibodies. This optimized assay eliminates the need for multiple wash steps, decreases variability and enables testing with smaller numbers of cells. In addition, we have modified the direct competition method to enable direct assay of whole blood specimens allowing for a one- step staining process and preservation at a central lab to eliminate PBMC isolation.

#### P16 Better efficacy of PD-1 antibody predicted by immune-related adverse effects is impaired by high dose steroids

##### Xue Bai, MD^1^, Michelle Kim^2^, Gyulnara Kasumova^2^, Tatyana Sharova^3^, Justine Cohen, DO^4^, Donald Lawrence, MD^4^, Christine Freedman, RN^4^, Riley Fadden, NP^4^, Krista Rubin, MS, FNP-BC^4^, Ryan Sullivan, MD^4^, Keith Flaherty^4^, Genevieve M. Boland, MD, PhD^2^

###### ^1^Massachusetts General Hospital Cancer Center, Harvard Medical School; Department of Renal Cancer and Melanoma, Peking University Cancer Hospital and Institute, Boston, USA; ^2^Department of Surgical Oncology, Massachusetts General Hospital, Harvard Medical School; Geisel School of Medicine at Dartmouth, Hanover, USA; ^3^Department of Surgical Oncology, Massachusetts General Hospital, Harvard Medical School, Boston, MA, USA; ^4^Massachusetts General Hospital Cancer Center, Harvard Medical School, Boston, MA

####### **Correspondence:** Genevieve M. Boland (gmboland@partners.org)

**Background**

PD-1 antibody has greatly improved the prognosis of unresectable or metastatic melanoma, and is now the standard first line therapy. It also brings a spectrum of immune-related adverse effects (irAEs). However, the correlation between the presence and timing of irAEs and the efficacy of PD-1 antibody remains elusive.

**Methods**

We retrospectively collected clinical data of pembrolizumab or nivolumab monotherapy-treated patients in Massachusetts General Hospital from 2009 to 2017. Correlations between irAEs and clinical outcomes were statistically analyzed.

**Results**

Of total 147 enrolled patients, 81 (55.1%) had irAE(s) (median 1/patient), 33 (22.4%) had severe irAE(s) (grade 3,4). The presence of irAE(s) was correlated with better therapeutic efficacy, which was impaired but not entirely offset by the application of high dose steroids, i.e. irAE+/high-dose steroids(-) subgroup had the best clinical outcome (median PFS 132.1 weeks, median OS not reached), followed by irAE+/high-dose steroids(+) (median PFS 43.0 weeks, median OS 182.6 weeks), which was better than irAE- subgroup (median PFS 11.4 weeks, median OS 74.7 weeks) (P<0.001 as for both PFS and OS). In total 158 irAEs were reported, among which 37 (23.4%) affected skin, 32 (20.3%) endocrine system, 31 (19.6%) muscle and joints, 21 (13.3%) gastrointestinal, 12 (7.6%) pulmonary, 9 (5.7%) hepatic, 4 (2.5%) renal, 4 (2.5%) neural, 2 (1.3%) pancreas, and 6 (3.8%) others. Median onset time of irAEs affecting each system was significantly different (P=0.036), early onset irAEs included neuropathy (6.9 weeks), hepatitis (7.3 weeks), and late onset irAEs included musculoskeletal (32.7 weeks), and cutaneous (27.6 weeks). Within the subpopulation of patients with irAE(s), those with musculoskeletal irAEs had better therapeutic response (ORR 84.6% vs. 48.1%) (P=0.005), longer median PFS (119.6 vs. 43.4 weeks) (P=0.010), and longer median OS (not reached vs. 189.6 weeks) (P=0.013). While cutaneous irAEs in total did not correlate with outcomes, the subset of vitiligo patients (n=8) had longer median PFS (not reached vs. 60.4 weeks) (P=0.032), and the tendency towards better therapeutic response (ORR 87.5% vs. 56.9%) (P=0.186) and longer median OS (not reached vs. 229.3 weeks) (P=0.191). Interestingly, rare irAEs* requiring high dose steroids (median onset of 7.7 weeks), were correlated with shorter median PFS (28.1 vs. 90.0 weeks) (P=0.006) and shorter median OS (120.0 weeks vs. not reached) (P=0.037).

**Conclusions**

The presence of irAE(s) serves as a prognostic biomarker during PD-1 antibody monotherapy. Application of high dose steroids impairs PD-1 antibody efficacy, and may impact subtype-specific predictive values of different irAEs.

#### P17 Combined MAGE-A1,3/6,4, and 10 expression levels quantified in solid tumors by (BaseScope™) RNA in situ hybridization (ISH) identify targets for immunotherapy

##### Anshika Bajaj, PhD^1^, Helly Xiao Yan Pimentel^2^, Bingqing Zhang, PhD^2^, Ruby Hsu, PhD^2^, Peter Berglund, PhD^1^, Jan Ter Meulen, MD, PhD^1^

###### ^1^Immune Design, Seattle, WA, USA; ^2^Advanced Cell Diagnostics, Neward, CA, USA

####### **Correspondence:** Anshika Bajaj (anshika.bajaj@immunedesign.com)

**Background**

Ongoing clinical trials of cancer vaccines and adoptive cell therapies target members of the melanoma-associated antigen (MAGE-A) family, highly prevalent in tumors. However, no multiplexed diagnostic assay is available to quantify MAGE-A expression for ideal patient selection. The homologous nature with 50-80% sequence identity between the MAGE-A genes poses significant challenges to their specific detection in tumors. Due to antibody cross-reactivity there is limited capability of protein-specific assays to detect and distinguish between the various MAGE-A antigens. Here, an RNA ISH based assay was developed to assess and quantitate *MAGEA1*, *MAGEA3/6*, *MAGEA4*, *MAGEA10* expression in normal and tumor tissue.

**Methods**

*MAGEA* (*-A1*, *-A3*, -*A4*, and *-A10*) specific probes were designed targeting sequences with minimal inter-gene identity. Tissue samples that passed quality control were evaluated for *MAGEA* expression. BaseScope™ LS Red ISH assays were performed on Leica Bond RX using the BaseScope LS kit on cell pellet arrays, tumor, and normal tissues.

**Results**

4 assays were developed, each designed to specifically detect RNA encoding MAGE-A1, -A3/6, -A4, or -A10. Experiments done in control cell lines (1 negative and 4 cells lines each expressing 1 of the 4 *MAGEA* genes) demonstrated that the assays were highly specific and sensitive for their respective target genes. *MAGEA* expression was assessed in 10 melanoma, head and neck, lung, and esophageal cancer biopsies. Samples were assigned dot scores based on semi-quantitative visual scoring of the number of dots (RNA molecules)/cell. Moderate-high expression of all 4 *MAGEAs* (score of 2-3) was observed in biopsies from 1/1 melanoma patients, 2/3 lung cancer patients, and 1/4 head and neck cancer patients. High *MAGEA1* and *3* expression only (score of 3) was observed in 3/4 head and neck cancer patients. As expected, analysis of normal tissue samples except for testes revealed minimal signal.

**Conclusions**

Specific and sensitive BaseScope assays were developed for *MAGEA1*, *MAGEA3/6*, *MAGEA4*, and *MAGEA10*. The assays demonstrate inter-gene specificity, are amenable to multiplexing, and can potentially be used as a companion diagnostic in clinical trials targeting *MAGEA* antigens. Furthermore, these preliminary results demonstrate that these 4 MAGE-A antigens are highly prevalent in cancers such as head and neck, melanoma, and lung and support the development of an active immunotherapy based on Immune Design’s dendritic cell-targeting ZVex® vector platform.

#### P18 Preliminary evaluation of a novel whole slide multispectral assessment of seven markers: Potential to minimize bias in the characterization of the tumor immune environment

##### Carmen Ballesteros Merino, PhD^1^, Shawn Jensen, PhD^2^, Carla Coltharp, PhD^3^, Kristin Roman, MS^3^, Chichung Wang^3^, Nikhil Lonberg, HSDG^2^, Sebastian Marwitz^2^, Tarsem Moudgil, MS^2^, William Miller, BS^2^, William Redmond, PhD^2^, Yoshinobu Koguchi, MD, PhD^2^, Carlo Bifulco, MD^2^, Clifford Hoyt, MS^3^, Bernard A. Fox, PhD^2^

###### ^1^Robert W Franz Cancer Center, Earle A Chiles Research Insititute, Portland, OR, USA; ^2^Robert W Franz Cancer Center, Earle A Chiles Research Institute, Portland, OR, USA; ^3^Perkin Elmer, Hopkinton, MA, USA

####### **Correspondence:** Bernard A. Fox (foxb@foxlab.org)

**Background**

PD-L1 expression and tumor-mutational burden enrich for patients that respond to checkpoint blockade, but these evaluations are only a component of the entire story. Recently, our lab reported that evaluation of specific cell-cell relationships provided a powerful biomarker for overall survival in patients with HPV- head and neck cancer (HNSCC). However, the areas selected for analysis were operator selected “hot spots”. This approach introduces the potential for unconscious bias in the selection process. To address this, we have sought to perform whole slide evaluations of sections to compare with hot spot analysis. This study is a preliminary report applying a novel set of fluorophores and filters that allow the visualization of seven colors on a whole slide.

**Methods**

Tissue samples included pellets of cultured lymphocytes and tumor specimens. A sample of the cultured lymphocytes that were fixed and embedded were analyzed by flow cytometry for immune markers. Formalin-fixed paraffin embedded (FFPE) sections were stained with antibodies for CD8, CD68, FoxP3, PD-1, PD-L1, cytokeratin and DAPI. PerkinElmer Opal reagents were used to identify markers and included standard and a new set of fluorophores that included Opal 480, 520, 570, 620, 690, and 780. Slides were imaged using a new scanning approach on a Vectra Polaris (PerkinElmer, Inc, Waltham, MA).

**Results**

Preliminary comparison of cells that were used to produce FFPE blocks by flow cytometry and multiplex IHC provided similar results for some markers. Determining optimal staining, exposure times and thresholds for analysis for this new method needs work, but the potential exists for effective evaluation of a whole slide with 7 different markers.

**Conclusions**

Our preliminary results provide reason to be optimistic that this approach can assess 7 colors in a whole slide. Whole sections labelled with 7 colors and spectrally unmixed supports deeper analysis of immune-biology on multiple scales, including re-analysis of spatial metrics based on emerging hypotheses about how cellular and expression distributions relate to disease progression and response to therapy.

#### P19 Molecular determinants of response to PD-L1 blockade across tumor types

##### Romain Banchereau^1^, Ning Leng^1^, Edward Kadel, BS^1^, Dorothee Nickles, PhD^1^, Steve Lianoglou, BS, MSc, PhD^1^, Oliver Zill^1^, Sushit Jhunjhunwala^1^, Luciana Molinero, PhD^1^, Mahrukh Huseni^1^, Marcin Kowanetz, PhD^1^, Richard Bourgon, BS, PhD^1^, Craig Cummings, PhD^1^, Sanjeev Mariathasan, PhD^1^, Priti Hegde, PhD^1^, Thomas Powles, MBBS, MD, MRCP^2^

###### ^1^Genentech, South San Francisco, CA, USA; ^2^BART, London, UK

####### **Correspondence:** Romain Banchereau (banchereau.romain@gene.com)

**Background**

Immune checkpoint inhibitors targeting the PD-1/PD-L1 axis lead to durable clinical responses in subsets of cancer patients across multiple indications including non-small cell lung cancer (NSCLC), urothelial carcinoma (UC) and renal cell carcinoma (RCC). This work aims at determining whether unifying molecular profiles can predict response across these tumor types.

**Methods**

379 samples from three phase II trials were investigated. PD-L1 expression on tumor-infiltrating immune cells (IC), tumor mutation burden (TMB) and bulk transcriptome measurements were obtained before treatment with atezolizumab from 218 UC (IMvigor210), 83 NSCLC (POPLAR) and 78 RCC (IMmotion150) patients. Objective response was assessed by RECIST v1.1. Patients from a phase I atezolizumab monotherapy basket study (PCD4989g) were employed as an independent validation cohort. PD-L1 IC was assessed by immunohistochemistry (Ventana SP142: >1% of IC was defined as positive). TMB was assessed by whole exome sequencing. Bulk tumor transcriptomes were assessed by RNAseq.

**Results**

Initial analyses focused on responder prevalence in PD-L1 IC+ and/or TMBhigh individuals. They revealed variable results across tumor types with overall sensitivity/specificity of 76.4%/34.5% and 74.5%/55.4% for PD-L1 IC and TMB respectively. Importantly, no common TMB threshold predicted response across indications. Unsupervised analysis revealed that RCC tumors cluster away from UC and NSCLCs. Supervised analysis showed that PD-L1 IC expression correlated with myeloid and lymphoid signatures across tumor groups, while few immune genes associated with TMB. Modular transcriptional analysis failed to identify a unified tumor signature associated with response, although parallels were seen between NSCLC and UC, but not RCC. Using a linear model that accounted for genes associated with PD-L1 IC levels, the CDK4/6 inhibitor CDKN2A, which is frequently mutated in UC and NSCLC tumors, was identified as the most significant correlate of response to PD-L1 inhibition, highlighting the association of non-immune pathways to checkpoint blockade outcome. Tumor-related pathways including mismatch repair and senescence were enriched in responders with low tumor immune infiltrate. Finally, machine learning identified a 42-gene signature associated with outcome, which included both immune- and tumor-related components. This signature complemented TMB and PD-L1 IC to increase responder prevalence both in training and independent validation cohorts.

**Conclusions**

While no unifying gene signature correlated with response across tumor types, consistent overlaps were observed between UC and NSCLC, highlighting common mechanisms of response to PD-L1 inhibition between tumors from different origins. Machine learning can integrate high-dimensional datasets across indications to identify both immune- and tumor-related determinants of response to checkpoint blockade.

#### P20 A structured tumor-immune microenvironment in triple negative breast cancer revealed by multiplexed ion beam imaging

##### Leeat Keren^1^, Marc Bosse^1^, Robert West^2^, Sean Bendall, PhD^1^, Michael Angelo, MD, PhD^1^

###### ^1^Stanford University, Stanford, CA, USA; ^2^Stanford, Stanford, CA, USA

####### **Correspondence:** Michael Angelo (mangelo0@stanford.edu)

**Background**

Cancer progression is a complex process that depends on the interplay between cells in the tumor, the microenvironment, and the immune system, which can act both to promote and suppress growth and invasion [1]. Triple-negative breast cancer (TNBC) is an aggressive form of invasive breast cancer lacking appreciable expression of therapeutic targets: estrogen receptor, progesterone receptor, and Her2 [2]. In terms of TNBC immunotherapy, no single biomarker has been sufficient for adequate patient stratification [3]. Consequently, there is still much interest in its tumor immune landscape: which immune cell types are present, which immunoregulatory proteins are expressed, and how these vary between patients.

**Methods**

We leveraged a next-generation tissue pathology imaging platform we have developed, Multiplexed Ion Beam Imaging [4] coupled to Time of Flight (MIBI-TOF) mass analysis, to perform a retrospective study on TNBC patients from the Stanford Pathology archive. With this we simultaneously quantified in-situ expression of 36 proteins covering identity, function and immune regulation at sub-cellular resolution in 41 TNBC patients. This data enabled us to develop a multi-step analysis pipeline for standardized processing of this multiplexed imaging cohort, including deep-learning-based segmentation, cell type identification, and spatial enrichment analysis of the tumor immune microenvironment.

**Results**

While the composition of tumor-immune populations varied widely between individuals, this heterogeneity could be reconciled by the overall amount of immune infiltration, where there was enriched co-occurrence and ordering of specific immune populations conserved across the cohort. Monocytes were at all levels of infiltrate while B and NK cells only in patients with the greatest immune cell density. At the same time, distinct immune populations expressed checkpoint proteins (i.e. PD1, PD-L1, IDO, and LAG3) in different patients, and patients that express one immunosuppressive pathway were more likely to express another. Data-driven analysis of spatial organization revealed either immune compartmentalized or immune mixed tumors. Most interestingly, this histological organization was significantly correlated with expression of checkpoint molecules (particularly PD1, PD-L1, and IDO) in a cell-type- and location-specific manner. Here, ordered immune structures along the tumor-immune border served as a hallmark of tumor compartmentalization and were linked to overall survival with standard chemotherapy.

**Conclusions**

Together, these data demonstrate an organization in the tumor-immune microenvironment that is structured in cellular composition, spatial arrangement, and expression of regulatory proteins. We elucidate these organizational features creating a resource for TNBC and provide a framework to apply highly multiplexed subcellular imaging to complex immune oncology.

**Acknowledgements**

S.C.B. is supported by a gift from Christy and Bill Neidig, the Damon Runyon Cancer Research Foundation (DRG- 2017-09), the NIH 1DP2OD022550-01, 5U19AI116484-02, and U19AIP97

*Corresponding author email: paola.nistico@ifo.gov.it9. M.A is supported by 1-DP5-OD019822. S.C.B and M.A. are jointly supported by 1R01AG056287–01, 1R01AG057915-01, and 1U24CA224309-01 from the NIH. L.K. is a Damon Runyon Fellow supported by the Damon Runyon Cancer Research Foundation (DRG-2292-17)

**References**

1. Chen DS, Mellman I. Elements of cancer immunity and the cancer–immune set point. Nature. 2017;541:321–330.

2. Denkert C, Liedtke C, Tutt A, von Minckwitz G. Molecular alterations in triple negative breast cancer—the road to new treatment strategies. Lancet. 2017;389:2430–2442.

3. Maleki Vareki S, Garrigós C, Duran I Biomarkers of response to PD-1/PD-L1 inhibition. Crit. Rev. Oncol. Hematol. 2017;116:116–124.

4. Angelo M, et al. Multiplexed ion beam imaging of human breast tumors. Nat. Med. 2014;20:436–42.

#### P21 Deep learning-based PD-L1 tumor cell (TC) scoring improves survival prediction compared to pathologists on durvalumab-treated NSCLC patients

##### Nicolas Brieu, PhD^1^, Ansh Kapil^1^, Aleksandra Zuraw, Dr^1^, Abraham Silva, MD^1^, Marlon Rebelatto, DVM, PhD, DACVP^2^, Keith Steele, DVM, PhD^2^, Guenter Schmidt, PhD^1^

###### ^1^Definiens, Munich, Germany; ^2^MedImmune, Gaithersburg, MD, USA

**Background**

PD-L1 expression in non-small cell lung carcinoma (NSCLC) patients is commonly quantified by the tumor cell (TC) score estimated by pathologists. An accurate score is key to identify patients that could benefit from anti-PD- L1 check point inhibitor treatment, patients with high score being more likely to respond to such therapy [1]. Recent advances in deep learning algorithms for computer vision enable an accurate alternative to pathologist scoring via the identification of positive and negative tumor regions [2]. With statistical analysis of the clinical response, we show the predictive value of the automated scoring system and evaluate it against pathologist scoring.

**Methods**

The dataset consists of tissue sections of NSCLC patients from subsets of NCT01693562 [1] and NCT02000947 [3] clinical trials and stained with Ventana SP263 PD-L1 assay. Using two-fold cross validation, we train a deep semi- supervised convolutional neural network [2] for the automated segmentation of PD-L1 positive and PD-L1 negative tumor cell regions. Training is based on labeled patches generated from the manual annotation of positive and negative tumor cell regions by two pathologists on a subset of images (n=20) as well as on unlabeled patches generated from the remaining non-annotated images (n=305). The dataset for network application, TC score estimation and further statistical analysis consists of the non-annotated NSCLC samples of the durvalumab monotherapy clinical trial (NCT01693562) for which overall survival (OS), progression free survival (PFS) defined by RECIST criteria, and three pathologist scores are available (n=152). The automated score is estimated from the segmented regions as the relative area of the PD-L1 positive tumor cell region. Using leave-one-out cross-validation, we finally optimize the cutpoint between low and high scores regarding log-rank test associated with overall survival and progression free survival.

**Results**

The deep learning-based score is strongly correlated with the consolidated pathologist score obtained by majority voting (Pearson:0.80). It yields more significant OS and PFS stratifications in terms of Cox proportional hazards regression than the pathologist score for both the standard 25% cut-off [1] and the respective optimized cut-off (Figure.1 and Table.1).

**Conclusions**

Our results suggest that the proposed deep learning based system for PD-L1 TC scoring enables the retrospective stratification of durvalumab-treated NSCLC patients into predictive groups. Upon further improvement of the correlation to pathologists and confirmation of the presented results in a prospective trial, we envision that the proposed model could be used in a clinical routine setting to identify patients which may benefit from anti-PD-L1 therapy.

**References**

1. M. Rebelatto, J. Walker et al., Development of a programmed cell death ligand-1 immunohistochemical assay validated for analysis of non-small cell lung cancer and head and neck squamous cell carcinoma, Diagnostic Pathology (2016), 11:95, DOI 10.1186/s13000-016-0545-8

2. A. Kapil, N. Brieu et al., Deep Semi Supervised Generative Learning for Automated PD-L1 Tumor Cell Scoring on NSCLC Tissue Needle Biopsies, ArXiv (2018), https://arxiv.org/abs/1806.11036

3. S. Antonia, NA. Rizni et al., Safety and antitumour activity of durvalumab plus tremelimumab in non-small cell lung cancer: a multicentre, phase 1b study, Lancet Oncol (2016), 17(3):299-308, DOI 10.1016/S1470- 2045(15)00544-6

**Ethics Approval**

This works relies on data from NCT01693562 and NCT02000947 clinical trials (clinicaltrials.gov)

**Fig. 1 (abstract P21).**
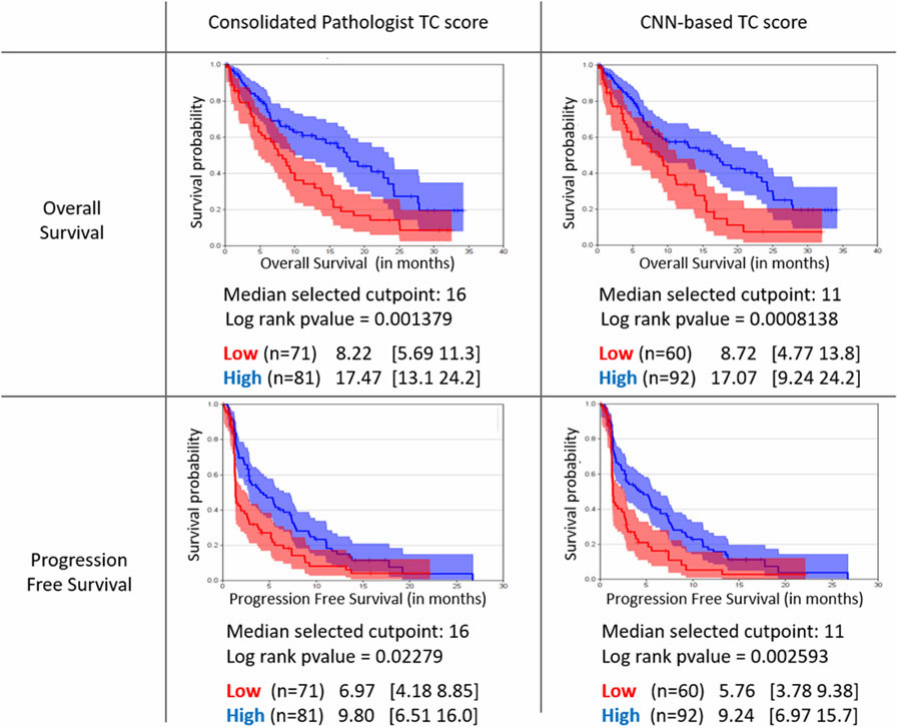
See text for description.

**Table 1 (abstract P21).**
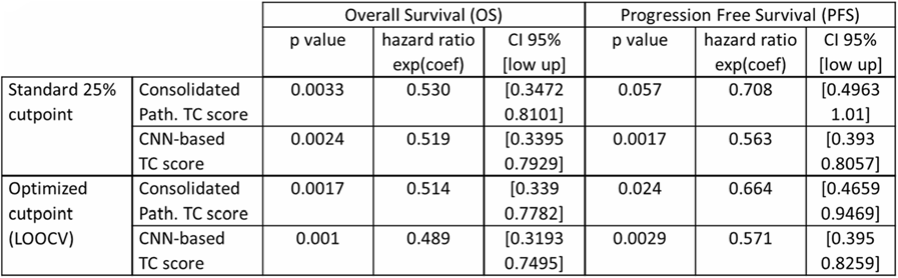
See text for description.

#### P22 Impact of tumor inherent interferons on immune reactivity and personalized therapy in triple negative breast cancer

##### Natasha Brockwell, BBiomed (Hons)^1^, Jai Rautela^3^, Tim Molloy, PhD^4^, Sandra O'Toole, MD, PhD^5^, Vinod Ganju, MBBS, FRACP^6^, Belinda Parker^1^

###### ^1^La Trobe Institute for Molecular Science, Bundoora, Australia; ^2^La Trobe Institute for Molecual Science, Bundoora, Australia; ^3^Walter and Eliza Hall Institute, Melbourne, Australia; ^4^St Vincents Centre for Medical Research, Sydney, Australia; ^5^Garvan Institute for Medical Research, Sydney, Australia; ^6^Hudson Institute for Medical Research, Frankston, Australia

####### **Correspondence:** Natasha Brockwell (n.brockwell@latrobe.edu.au)

**Background**

Triple negative breast cancer (TNBC) is known for its ability to rapidly metastasize within the first two years and its association with tumor infiltrating lymphocytes (TILs). As immune infiltrate has been associated with a good prognosis and therapeutic response in TNBC, immunotherapy is now being trialed. However, responses have been underwhelming to date and difficult to predict, leading to an inability to accurately weigh up the benefit-to-risk ratio for their implementation. Previous work done in our laboratory demonstrated that type I IFN signalling can increase the heat of the tumor, induce a tumor specific T cell responses and sensitize mice to checkpoint inhibitors [1]. This suggested that characterization of a tumors heat is imperative in deciding which patients are most likely to benefit from immunotherapy and the type of immunotherapy they should receive.

**Methods**

Multiplex immunohistochemistry using the OPAL method was utilized to characterize tumor heat through assessment of T cell subsets and effector status and novel IFN biomarkers. A TNBC cohort (n = 21) where sequential biopsies were taken pre, mid and post chemotherapy was used to assess the role of tumor heat in chemotherapeutic response and relapse. Two independent adjuvant TNBC cohorts (n = 398; n = 159) were used to validate findings. Murine TNBC cells were manipulated to have constitutive expression of the type I IFN pathway. Syngeneic mouse models were used to assess the role of inherent type I IFN signalling on chemotherapeutic response, survival and the immune landscape.

**Results**

We demonstrate the superior prognostic information that can be gathered from TIL characterization whereby T cell subsets and their effector function can be used to predict response to chemotherapy and relapse. Furthermore we identified a novel prognostic marker that indicates presence of an intact type I IFN signalling pathway. Patients with loss of this marker were up to eight times more likely to relapse with metastatic disease than those who retained the biomarker, this was prognostic in 3 independent TNBC cohorts. Overexpression of the type I IFN pathway in murine TNBC cells resulted in increased sensitivity to chemotherapy, decreased metastasis and promotion of a T cell inflamed tumor.

**Conclusions**

Our work suggests tumor inherent type I IFN signalling and TIL characterization predicts relapse. Immunotherapy aimed at increasing tumor heat may hold promise in those lacking immune activation or IFN signalling prior to chemotherapy or checkpoint inhibitors, supporting the notion of TME characterization pre-treatment to personalize therapy.

**References**

1. Brockwell N K, Owen K L, Zanker D, Spurling A, Rautela J, Duivenvoorden H M, Baschuk N, Caramia F, Loi S, Darcy P K, Lim E, and Parker B S, “Neoadjuvant Interferons: Critical for effective PD-1 based immunotherapy in TNBC,” Cancer Immunol. Res., Aug. 2017; Vol 5:871-884.

**Ethics Approval**

This study was approved by the latrobe animal ethics committee, approval number AEC15-62. This study was approved by the human resources ethics committee of the Royal Prince Alfred Hospital, approval number X15-0388 [SSA/16/RPAH/397].

#### P23 Centrifuge-less immunostaining of suspension cells for flow cytometry analysis by DA-Cell™ washer and plate for superior data and workflow

##### Namyong Kim, PhD, Melvin Lye, Namyong Kim, PhD

###### Curiox Biosystems

####### **Correspondence:** Melvin Lye (melvin@curiox.com)

**Background**

We describe the DA-Cell™ system, a novel wall-less plate and laminar-flow cell washer that enables the automated washing of suspension cells and retains more than 95% cells at a fraction of the time and with higher viability of cells.

**Methods**

The wall-less DropArray (DA) plate consists of an array of 96 hydrophilic spots separated and surrounded by a hydrophobic surface, which functions as a virtual wall. In a typical immunostaining assay, a 50 μl drop, containing cells and antibody mix, is dispensed on each spot of the DA plate. During incubation, cells settle on the surface of the spots. The plate then undergoes a laminar-flow washing process in DA-Cell washer by repeated cycles of aspiration and dispensing of buffer through two sets of nozzles. The controlled buffer flow minimizes turbulence and cell loss. The cell washing process only requires 3-4 minutes by eliminating the need of a centrifuge, which also reduces stress on the cells and possible cross-contamination of antibodies on cell membranes, leading to better segregation of cell populations in flow cytometry. Additional incubation time of 10-20 minutes improves cell retention to more than 99%. Most importantly, cell incubation and washing on a DA-Cell system minimizes operator variability as mixing and washing steps are mechanically controlled and mostly automated, significantly improving reproducibility and consistency of flow cytometry analysis.

**Results**

We show a series of immunostaining assays comparing the DA-Cell system with a conventional centrifugation. Based on the staining index, absolute cell counts and additional data, we demonstrate that the DA-Cell system produces superior data while simplifying and expediting cell preparation for flow cytometry analysis.

**Conclusions**

The DA-Cell system produces superior data while simplifying and expediting cell preparation for flow cytometry analysis.

**Acknowledgements**

Singapore Immunology Network

#### P24 Centrifuge-less red blood cell lysis and immunostaining of whole blood for flow cytometry using DA-Cell washer and plate

##### Namyong Kim, PhD, Melvin Lye, Namyong Kim, PhD

###### Curiox Biosystems, San Carlos, TX, USA

####### **Correspondence:** Melvin Lye (melvin@curiox.com)

**Background**

Blood cells are prime indicators of immuno-surveillance, and the ease of blood sampling makes blood analysis a key interest for clinical and research applications. While current flow cytometry methods are high-throughput and provide fine resolution in the segregation of white blood cell (WBC) populations, WBC enrichment involving red blood cell (RBC) lysis are laborious and typically performed manually, contributing to experimental variability especially as blood cells are sensitive to physical and chemical stress.

**Methods**

We describe RBC lysis and leukocyte immunostaining on a centrifuge-less platform DA-Cell™, using a novel wall-less plate and laminar flow washer. The DropArray (DA) plate consists of an array of 96 hydrophilic spots surrounded by hydrophobic surface, which functions as a virtual wall that separates each spot. The maximum volume of each spot can be increased to 300μL with an insert. During lysis, WBC settle to the surface of the spot, allowing the spent lysis buffer to be removed. After removal of the insert, the plate goes through a gentle 3-4min laminar-flow washing process in the DA-Cell washer, decreasing cell washing time by at least 50% while eliminating centrifugation that stresses cells and disrupts antibody binding.

**Results**

In studies comparing mouse whole blood lysis (1X RBC Lysis buffer, eBioscience) and antibody staining by conventional tube centrifuge and DA-Cell, DA-Cell achieved dramatically higher staining index and improved resolution of cell cluster by flow cytometry. CD45+ leukocyte recovery and viability was uncompromised compared to conventional tube centrifuge.

**Conclusions**

In summary, DA-Cell system provides gentle, fast and convenient blood lysis, while improving data quality with superior antibody staining.

#### P25 Consistent pharmacodynamics and immunological responses to the TLR9 agonist, SD-101, following intratumoral injection in multiple cancer types

##### Albert Candia, PhD^1^, Cristiana Guiducci, PhD^1^, Ezra Cohen, MD^2^, Ronald Levy, MD^3^, Mohammed Milhem, MBBS^4^, Antoni Ribas, MD, PhD^5^, Thomas Tuting, MD^6^, Erick Gamelin, MD PhD^1^, Robert Janssen, MD^1^, Robert Coffman, PhD^1^

###### ^1^Dynavax Technologies, Berkeley, CA, USA; ^2^Moores Cancer Center, La Jolla, CA, USA; ^3^Stanford University Hospital and Clinics, Stanford, CA; ^4^University of Iowa Health Care, Iowa City, IA, CA; ^5^University of California at Los Angeles, Los Angeles, CA; ^6^University Hospital Magdeburg, Magdeburg, Germany

####### **Correspondence:** Albert Candia (acandia@dynavax.com)

**Background**

SD-101 is a synthetic class C CpG oligonucleotide agonist of Toll-like receptor 9. SD-101 stimulates dendritic cells to release interferon-alpha and mature into antigen presenting cells that effectively activate T cell responses. SD-101 is administered intratumorally (IT) and has been evaluated in combination with radiation therapy for lymphoma, and is currently being evaluated with pembrolizumab for melanoma and HNSCC. Pharmacodynamic and biomarker assessments across these three different tumor types offer mechanistic insights into the anti-tumor activity observed in the clinic.

**Methods**

Peripheral blood collected before and 24 hours after dosing was analyzed with Nanostring or qPCR by a panel of IFN responsive genes as an indirect measure of target engagement. Biopsies from injected lesions were collected prior to treatment and at specific post-dose time points, and gene expression was analyzed by Nanostring to evaluate the immunophenotype of the tumor environment. Tumor responses were assessed using Cheson criteria for lymphoma and RECIST v1.1 for melanoma and HNSCC.

**Results**

Type 1 IFN production was demonstrated by the activation of IFN responsive genes in peripheral blood. The range of induction on an individual basis (2 to 29 fold) and maximal induction on an averaged cohort basis (approximately 10-fold) were comparable across tumor types indicating similar mechanism of dendritic cell activation. Analysis of gene expression in tumor biopsies before and after treatment across the three tumor types shows a consistent increase in immune functions and cell types expected to contribute to anti-tumor activity. These changes are also consistent with the known mechanisms of action for SD-101 and include increases in CD8+, Th1, and NK cells. Responding patients (PR/CR) had average increases of at least 2-fold and as high as 9-fold in these cell types and functions. These increases in immune functions occurred in patients with or without prior checkpoint inhibitor therapy, and in the largest data set from melanoma patients, the changes in these cell types and functions correlated significantly with reductions in size of target lesions.

**Conclusions**

Biomarker assessments across three different tumor types following IT administration of SD-101 demonstrate consistent pharmacodynamic and biomarker activities consistent with its mechanism of action. The results suggest that activation of the innate immune system may be a core component of combination therapies in orchestrating an anti-tumor immune response in a wide range of cancer types.

**Ethics Approval**

The studies described were approved at the Institutional Review Boards of the respective clinical sites.

#### P26 High-dimensional flow cytometry of circulating immune cells predicts clinical responses to combination Immune Checkpoint Blockade (ICB) and Radiotherapy (RT) in Gastroesophageal Cancer (GEC)

##### Joseph Chao, MD, Wanqiu Hou, Yi-Jen Chen, MD, PhD, Helen Chen, Michael Tajon, Marwan Fakih, MD, Peter P. Lee, MD

###### City of Hope Comprehensive Cancer Center, Duarte, CA, USA

####### **Correspondence:** Peter P. Lee (plee@coh.org)

**Background**

While ICB has been promising, the majority of GEC patients do not respond to single agent anti-PD-1 therapy. Combination strategies are being explored to augment immune responses including combining ICB with RT. We are currently conducting a prospective trial testing palliative RT with pembrolizumab in patients with metastatic GEC. To study immune correlates of ICB, we employed high-dimensional flow cytometry single cell analyses to characterize blood immune profiling as predictive biomarkers of clinical response.

**Methods**

In this single institutional trial, patients received standard palliative RT 30 Gy over 10 fractions to a single site of disease. Pembrolizumab 200 mg was given concurrently with RT with first dose concordant with the first fraction. Cycles repeated every 3 weeks for up to 35 cycles in the absence of disease progression or unacceptable toxicity. Peripheral blood was collected at baseline prior to first fraction of RT (C1D1 pembrolizumab) and ~21 days after completion of RT (~C2D15 pembrolizumab). Blood comprehensive immune profiling was interrogated using three 15-color flow cytometry panels. Abscopal responses were assessed using RECIST1.1 of lesions out of the field of RT.

**Results**

In this current analysis, 5 patients were included. RECIST responses included 2 confirmed partial responses (PRs), and 3 patients with progressive disease. The 2 PRs have been durable lasting >12 months and ongoing at data cut- off. On C2D15, most patients demonstrated a decrease in CD56hi NK cells (p=0.04), CD1c+ dendritic cells (p=0.02), plasmacytoid dendritic cells (p=0.01), CD33hi myeloid-derived suppressor cells (p=0.02), and PD- 1+KLRG1+ exhausted CD8 T cells (p=0.005). Comprehensive immune profiling demonstrated a strong correlation of RECIST responses with low levels of circulating follicular helper T cells (r=0.99, p=0.001), and PD-1+BTLA+ exhausted CD4 T cells (r=0.91, p=0.03) at baseline C1D1. Analysis of immune changes over time also demonstrated a strong correlation of RECIST responses with an increase in circulating T cells (r=0.94, p=0.02) and nonclassical monocytes (r=0.92, p=0.03) as well as a decrease in Th2 cells (r=0.89, p=0.04) at C2D15 vs. C1D1.

**Conclusions**

Palliative RT plus pembrolizumab demonstrated encouraging activity in our dataset. Durable RECIST responses correlated with changes at C2D15 in circulating innate immune (T cells and nonclassical monocytes) as well as adaptive immune signatures (follicular helper T cells, Th2, and exhausted CD4 T cells). The ability to identify blood biomarkers early in ICB therapy that may predict durable clinical benefit is of significant clinical utility in GEC and warrants study in larger prospective cohorts.

**Acknowledgements**

Joseph Chao and Wanqiu Hou contributed equally to this work.

**Trial Registration**

NCT02830594

**Ethics Approval**

The study was approved by the Institutional Review Board of the City of Hope, approval number 16099.

#### P27 Tumor mutational burden assessment on FFPE samples using a targeted next-generation sequencing assay

##### Ruchi Chaudhary, PhD^1^, Dinesh Cyanam, PhD^2^, Vinay Mittal^2^, Charles Scafe^2^, Warren Tom, PhD^2^, Janice Au- Young^2^, Seth Sadis^2^, Fiona Hyland^2^

###### ^1^Thermofisher Scientific, South San Francisco, CA; ^2^Thermo Fisher Scientific, South San Francisco, CA

####### **Correspondence:** Ruchi Chaudhary (ruchi.chaudhary@thermofisher.com)

**Background**

Recently, high Tumor Mutational Burden (TMB) was associated with significantly longer progression-free survival from immune checkpoint blockade combination therapy in NSCLC. Although TMB was originally determined by whole exome sequencing (WES) of matched tumor-normal samples, the high input requirement, complex bioinformatics, and long turn-around time makes this approach impractical for routine testing. Herein, we develop a targeted amplicon-based panel for computing TMB and detecting important variants from FFPE research samples.

**Methods**

A targeted panel was designed that included 409 key cancer genes covering 1.7 Mb of genomic region. Utilizing Ion AmpliSeq multiplex PCR chemistry, the workflow required only 20 ng of input DNA. The assay enabled a 2.5-day turn-around time from sample to report. The workflow enabled < 60 min of hands-on time for automated library preparation and templating on a batch of 4 samples. Sequencing was performed on Ion GeneStudio S5 System at sufficient coverage depth (~1200x) to support accurate variant detection with an analysis pipeline containing optimized variant calling parameters. A tumor only informatics workflow was developed that removed germline variants present in population databases. Two cell line samples and nine FFPE samples were analyzed by the tumor only workflow. Matched tumor-normal samples were analyzed by WES and the tumor samples were independently analyzed using the targeted TMB panel.

**Results**

An in-silico analysis using 10,000 exomes from the TCGA MC3 project demonstrated the panel could support high sensitivity (≥85%) and PPV (≥90%) necessary to stratify high and low mutation burden samples. TMB estimates on a normal diploid cell line (NA12878) was < 1 TMB for all 8 replicates. In a cancer cell line (HCC1143; expected TMB 8.33 mutations/Mb), the average TMB for 4 replicates was 6.11 (SD 0.43). TMB estimates obtained with the tumor only workflow using the targeted panel had high concordance (r2 = 0.87) with the TMB values obtained from the matched tumor/normal analysis using WES. For two samples with highest TMB by both assays, the assay detected loss of function mutations in MSH2 and TP53 genes. The informatics pipeline identified mutation signatures consistent with specific mechanisms such as UV and tobacco damage, and detected samples impacted by FFPE processing.

**Conclusions**

A simple workflow has been developed on the Ion Torrent sequencing platform to estimate TMB from FFPE and fresh frozen tumor research samples. This solution will advance research in immuno-oncology.

#### P28 Detection and validation of cancer immunotherapy biomarkers in blood and urine-based liquid biopsy

##### Simo Zhang^2^, Shidong Jia^2^, Amy Wang^2^, Chen Xie^2^

###### ^1^Predicine, Inc., Hayward, CA, USA; ^2^Predicine, Hayward, CA, USA

####### **Correspondence:** Amy Wang (amy.wang@predicine.com)

**Background**

Immunotherapy response varies widely, making it difficult for physicians to know whether immunotherapy will be effective for a given patient. Indeed, ~80% or more patients with cancer fail to respond to checkpoint inhibitor immunotherapy. In addition to PD-L1IHC staining, recent studies reported that patients with deleterious mutations in mismatch repair (MMR) genes, high tumor mutation burden (TMB) or microsatellite instability (MSI) are also associated with better clinical response. As tissue biopsy represents a practical challenge due to its insufficient quantity or lack of access, noninvasive molecular has emerged as an efficient complementary test and attracted increasing attention in clinical development of cancerimmunotherapy. With Predicine’s gene RARDAR technology, we developed a blood-based PredicinePLUS NGS panel to capture genomic alterations in 180 cancer genes including tumor mutation burden (TMB) and microsatellite instability (MSI). Technical validation was performed to evaluate assay sensitivity, specificity and accuracy using reference samples with known genetic profiling. The panel has been further tested using tissue biopsy and plasma samples from cancer patients. The development of PredicinePLUS panel offer a comprehensive solution to stratify and monitor cancer patients who may benefit from cancer immunotherapy.

**Methods**

Nucleic acids were extracted from plasma samples and tested for DNA based SNV, CNV and gene rearrangement by proprietary pipeline.

**Results**

Mutation detection at DNA level can go down to 0.1% AF. At 0.25% expected AF, 94.4% SNVs were detected; at 0.1% expected AF, 78.6% SNVs were detected.High linearity was observed from detected and expected copy number from spiked-in cell lines; clinical validation of HER2 amplification in breast cancerHigh correlation between panel-TMB and TMB from WES on 14 cell lines. Consistency of panel-TMB at AF =0.5% or above in a series of dilution of reference materialsHigh correlation of panel-TMB with WES in a public study (Rizvi et al. Science 2015). D. high panel-TMB showed favorable PD-1 response in the public study.

**Conclusions**

A non-invasive PredicinePLUS NGS test was developed to support cancer immunotherapy clinical studies. A cfRNA-based PD-L1 mRNA assay was developed to monitor PD-L1 mRNA gene expression in circulation.

#### P29 Ensemble computational intelligence reveals novel molecular signatures of cancer biology and pan-cancer survival

##### Richard Williams, Thomas Chittenden, Nicholas Cilfone, x, Jeffrey Gulcher

###### Wuxi NextCODE, Cambridge, MA, USA

####### **Correspondence:** Thomas Chittenden (tchittenden@wuxinextcode,com)

**Background**

Next-generation sequencing has significantly advanced our understanding of cancer biology by providing a unique genomic perspective of the molecular states of human disease. However, combining multiple ‘omics’ measurements into biologically-relevant statistical computing frameworks to define causal molecular underpinnings of disease remains a significant challenge.

**Methods**

To address these issues, we developed novel feature learning approaches that enhance quantitative assessment of annotated tissues from The Cancer Genome Atlas. Our a priori biological-knowledge and data-driven network-based approaches improve performance and interpretability of both deep learning and probabilistic programming strategies.

**Results**

Herein, we demonstrate the utility of collapsing molecular signals, from five different -omics platforms, into integrated metagenes that are highly informative across roughly 8,200 tumors, encompassing 22 cancer types. We identified multiple immune related genes and pathways comparing cancer sub-types (e.g. CCR1, IFNA4, CD34, IL25 – kidney renal clear cell carcinoma vs. kidney renal papillary cell carcinoma), between 22 diverse cancer types (e.g. IL-20 and tumor necrosis factor production nested in negative regulation of cellular metabolic process), and associated with overall patient survival (e.g. FCGR2A, IFNE, TGFB1, IL23A, CD80 and type I interferon signaling pathway nested in cell proliferation).

**Conclusions**

Our results demonstrate the potential of deep learning methodologies to help revolutionize the analysis and interpretation of multi-omics data, how to identify more complex disease etiology than previous methods, and how to hypothesize putative ‘network driver genes’ of disease state and progression. Taken together, these aspects allow researchers to generate better novel hypotheses of therapeutic targets or diagnostic biomarkers.

#### P30 Tracking the cancer immune response using neural network deep learning of serial inflammatory marker data for forecasting timing of therapy

##### Brendon Coventry, MD PhD, Mohsen Dorraki, MSc, BS, Anahita Fouladzadeh, BSc, Andrew Alison, Derek Abbott, PhD

###### University of Adelaide, Adelaide, SA, Australia

####### **Correspondence:** Brendon Coventry (brendon.coventry@adelaide.edu.au)

**Background**

The immune response in advanced cancer patients is not static but fluctuates under homeostatic control around a mean level of inflammation indicative of the anti-cancer response occurring in the patient. Immunotherapy given during immune activation (as opposed to immune inhibition) might be expected to better induce stronger anti-cancer responses, therefore timing is likely to be important. In multiple studies, the key inflammatory marker C-reactive Protein (CRP) has been widely associated with cancer survival; predicting cancer risk; a bio-maker for tumour recurrence; as a marker in oncology for prognosis; and as a reliable tool for making critical treatment decisions for several cancer types. Since CRP is biomarker of immune system activity, and CRP concentrations exhibit low values in healthy subjects, the ability to forecast CRP trends might potentially guide clinical decisions in cancer therapies based on the inflammatory state existing in the patient at the precise time of treatment.

**Methods**

We investigated time-series analyses of our previous data sets from advanced melanoma and other ovarian cancer patient data using (i) Periodogram analysis and (ii) Recurrent Neural Networks (RNNs) using Long Short-Term Memory (LSTM)-based, approaches to predict the future state in a C-reactive protein (CRP) time-series in cancer patients. Deep learning provided CRP time-series forecasting.

**Results**

Using Periodogram methods, the time-series used in [1,2] did not contain enough data points in the measured time period to conclude whether the CRP data was periodic or not, particularly for the previously hypothesised period of seven days. Moreover, the study [3] provided a prescription for the minimum data sampling rate required for improved testing of a periodic CRP (or other biomarker) signal hypothesis. We abandoned this method in favour of investigating RNN approaches. The distribution of CRP was highly skewed, so a log(.) representation that is more symmetric and less skewed is recommended for CRP estimation. We challenged our data interpretation [1] and other data [4] for periodicity in either serial daily CRP measurements in melanoma patients, or in gynaecological cancer patients [2] with less frequent measurements.

**Conclusions**

Deep learning and other of machine learning-based approaches for biomedical signal analysis can be used to predict trends in C-reactive protein time-series, with greater accuracy than periodogram approaches. Deep LSTM RNN with 200 layers achieves the lowest prediction error. These approaches offer useful avenues for bio-marker monitoring. Forecasting CRP trends can provide potentially valuable information for guiding clinical decision-making for more accurately timing of therapies, including immunotherapies.

**Acknowledgements**

Australian Melanoma Research Foundation & Cancer Council SA/ SA Government/ SAHMRI for supporting the Trial Data collection and CRP monitoring,

**References**

1. Coventry BJ, Ashdown ML. Quinn MA, Markovic SN, Yatomi-Clarke SL, Robinson AP. CRP identifies homeostatic immune oscillations in cancer patients: a potential treatment targeting tool? J Transl Med. 2002; vol. 7, no. 102.

2. Madondo MT, Tuyaerts S, Turnbull B, Vanderstraeten A, Kohrt H, Narasimhan B, Amant F, Quinn M, and Plebanski M. Variability in CRP, regulatory T cells and effector T cells over time in gynaecological cancer patients: a study of potential oscillatory behaviour and correlation. Journal of Translational Medicine. 2014; 12:179.

3. Dorraki M, Fouladzadeh A, Salamon SJ, Allison A, Coventry BJ, and Abbott D. On detection of periodicity in C- reactive protein (CRP) levels, Sci. Rep., in press.

4. Leontovich AA, Dronca RS, Suman VJ, Ashdown ML, Nevala WK, Thompson MA, Robinson A, Kottschade LA, Kaur JS, McWilliams RR, Ivanov LV, Croghan GA, Markovic SN. Fluctuation of systemic immunity in melanoma and implications for timing of therapy. Front Biosci (Elite Ed). 2012 Jan 1;4:958-75.

#### P31 Prevalence of high microsatellite instability in cancer patients in the real world

##### Razvan Cristescu, PhD^1^, Kai-Li Liaw, PhD^1^, Scott Pruitt, MD, PhD^1^, Mark Ayers, PhD^1^, Jianda Yuan, MD, PhD^1^, Thao Vo, MD^1^, Senaka Peter^1^, Andrew Joe, MD PhD^1^, Darcy Hille^1^, Sun Young Rha^2^, Torben Steiniche^3^, Andrey Loboda, PhD^1^

###### ^1^Merck & Co., Inc., Kenilworth, NJ, USA; ^2^Yonsei Cancer Center, Seoul, Korea, Republic of; ^3^Aarhus University Hospital, Aarhus, Denmark

####### **Correspondence:** Razvan Cristescu (razvan_cristescu@merck.com)

**Background**

High microsatellite instability (MSI-H) cancers are vulnerable to immunotherapies targeting the PD-1/PD-L1 pathway. PD-1–blocking mAb pembrolizumab was recently approved by the FDA for the treatment of MSI-H cancer, regardless of tumor histology or location. This analysis evaluated MSI-H prevalence by cancer type and stage using several real-world observational studies to examine the potential patient populations that might benefit from treatment.

**Methods**

Four data sources, including 1 database and 3 retrospective observational molecular epidemiology studies, were evaluated. Microarray analysis of loss of MLH1 gene expression was used as a surrogate for MSI-H status (<2.35 on log10 scale in quantile-normalized data) to assess prevalence of MSI-H in the Moffitt Cancer Center database of ~16,000 archived tumors. Results were compared with those of 3 epidemiologic studies that evaluated MSI-H status using a PCR-based assay on archival tissue of gastric, ovarian, endometrial, cervical, and 8 rare cancers, most (72%- 100%) from patients with stage III-IV disease.

**Results**

In the Moffitt database, for the following cancers, MSI-H prevalence by MLH1 expression loss was: endometrial (138/664, 20.8%), sarcoma (4/38, 10.5%), gastric (4/41, 9.8%), colorectal (194/2251, 8.6%), esophageal (2/49, 4.1%), kidney (2/60, 3.3%), cervical (2/72, 2.8%), melanoma (1/41, 2.4%), prostate (1/88, 1.1%), lung (22/2064, 1.1%.), ovarian (5/523, 1.0%). MSI-H prevalence was generally higher in stage I-II than in stage III-IV. In epidemiologic studies of advanced disease, MSI-H prevalence by PCR was assessed in Korean patients with gastric cancer (0/103, 0%) and in patients with ovarian (1/40, 2.5%), endometrial (7/49, 14.3%), cervical (1/44, 2.3%), and rare cancers (0/305, 0%) in Denmark.

**Conclusions**

Using MLH1 expression loss as a surrogate, a large database with comprehensive molecular data of cancer patients provided MSI-H prevalence estimates in many cancer types not usually tested for MSI-H. The results were generally comparable with those of epidemiologic studies that used PCR-based MSI testing. These real-world data found MSI-H tumors in multiple cancers and may help to identify patients who can potentially benefit from treatment with pembrolizumab.

**Ethics Approval**

The abstract is based upon 1 database and 3 retrospective observational molecular epidemiology studies; no ethics approval was required.

#### P32 Monitoring of M-MDSC vs. G-MDSC in clinical studies – which is more important? (monocytic versus granulocytic myeloid derived suppressor cells)

##### Henry Hepburne-Scott, PhD, Phoebe Bonner-Ferraby

###### Serametrix, Carlsbad, CA, USA

####### **Correspondence:** Phoebe Bonner-Ferraby (pbf@serametrix.com)

**Background**

It is now almost universal good practice to monitor MDSC during clinical trials for novel checkpoint inhibitors and combination therapies. This is because MDSC protect tumors from anti-PD-1 and other such drugs by infiltrating tumors and providing localized immunosuppression. MDSC monitoring is also increasingly important in combination studies to help assess the efficacy of putative anti-MDSC agents, such as HDAC inhibitors.However, MDSC is an elusive biomarker, a “catch-all” category of immature immune cells and immunologists rarely agree on a definitive phenotype. Even having navigated the minefield of markers there is still the question of whether monocytic or granulocytic MDSC are dominant in protecting tumors from checkpoint-mediated de-repression of cytotoxic T cells. Whilst a biomarker assay for M-MDSC is already well established and widely used, a reliable assay for G-MDSC has proved more challenging. Here we report the development and application of a novel assay for G-MDSC for use in I-O clinical studies.

**Methods**

A flow cytometry panel consisting of Lineage Cocktail, CD14, CD33, CD15 and HLA-DR was developed, validated and used to identify G-MDSC in peripheral whole blood samples drawn from cancer patients. To ensure operator independent gating of the continuous HLA-DR marker a computational algorithm was used to determine sample- specific thresholds for gating. Data for G-MDSC were compared with same-sample data for M-MDSC.

**Results**

The assay was successful in identifying G-MDSC in peripheral Whole Blood samples collected in 5mL Cyto-Chex BCT tubes. A single blood draw yielded sufficient material to measure both M-MDSC and G-MDSC from the same sample. However, during assay validation the stability of the G-MDSC was found to be significantly less than the stability of M-MDSC (logistical implications of this will be discussed). There was poor correlation between the two cell types: samples that were rich in M-MDSC did not always have high levels of G-MDSC and vice versa.

**Conclusions**

Whilst MDSC monitoring is a valuable part of some immune-oncology clinical development it may not be sufficient to rely on measuring the monocytic phenotype alone for all studies. The poor correlation between M-MDSC and G-MDSC observed in this study suggests that both types should be included if a biomarker program is to be truly effective. The use of a combo-MDSC assay will enable a fuller understanding of the mechanisms of resistance to checkpoint blockade.

#### P33 DNA damage detected by localized γH2AX is associated with elevated TILs and PD-L1 expression in human colorectal carcinomas

##### Shruti Desai, PhD, Parker Sulkowski, Aravind Kalathil, Ranjini Sundaram, Ila Datar, PHD, Charles Fuchs, MD, MPH, Patricia LoRusso, DO, Ranjit Bindra, Kurt A. Schalper, MD, PhD

###### Yale University School of Medicine, New Haven, CT, USA

####### **Correspondence:** Kurt A. Schalper (kurt.schalper@yale.edu)

**Background**

Cancer cells accumulate genomic aberrations due to the frequent combination of increased DNA damage and decreased DNA repair capacity. H2AX is a histone component of nucleosomes and its phosphorylation on Serine139 (γH2AX) is the first step in recruiting repair proteins upon DNA damage. DNA alterations can produce mutant neoantigens that are recognized as non-self and presented by the HLA system to trigger anti-tumor immune responses and mediate sensitivity to immune checkpoint blockers. To date, evidence for the association between active DNA damage in cancer cells and adaptive anti-tumor immune responses in intact tumors specimens remains elusive.

**Methods**

Using γirradiated cell line preparations and expression controls, we standardized a multiplexed quantitative immunofluorescence (mQIF) panel for simultaneous and localized measurement of DAPI (all cells), cytokeratin for tumor epithelial cells (AE1/AE3, DAKO), γH2AX to map active DNA damage (JBW301, Millipore), CD3 for T- lymphocytes (Rabbit polyclonal, DAKO) and PD-L1 (E1L3N, CST) in formalin-fixed paraffin-embedded (FFPE) tissue samples. We then used the assay to study 265 stage I-IV colorectal carcinomas (CRCs) from Yale represented in tissue microarray format. We analyzed the level of the targets, their association and correlation with major clinicopathologic variables and survival.

**Results**

The levels of γH2AX (but not total H2AX) were significantly higher in FFPE preparations of γirradiated HEK293 cells than in control/untreated cells or in morphologically normal human tissues. The radiation-induced γH2AX increase was abrogated in HAP1 cells with targeted deletion of the H2AX gene. Detectable γH2AX protein was found in 170 (64%) of CRCs with nuclear staining pattern and predominant expression in cytokeratin-positive tumor cells. Elevated tumor γH2AX was significantly associated with increased CD3+ tumor infiltrating lymphocytes (TILs) and PD-L1 protein expression (P<0.05). Elevated CD3, PD-L1 and γH2AX were associated with lower tumor stage and better overall survival in the cohort. No significant association was seen between the markers and age, gender or smoking status.

**Conclusions**

Tumor DNA damage as measured by nuclear γH2AX protein expression occurs in 64% of CRCs and is associated with increased anti-tumor immune responses and better prognosis. TILs and PD-L1 are prognostic in CRC. Our results support targeting DNA repair deficiency pathways in combination with immune stimulatory agents as therapeutic strategy in a proportion of CRCs.

**Acknowledgements**

Research supported by a Stand Up to Cancer Colorectal Cancer Dream Team Translational Research Grant (Grant Number SU2C-AACR-DT22-17)

#### P34 Clinical application of urinary cell free DNA as a marker for cancer

##### Jeffrey Ding, Debin Sun

###### Admera Medical Technology Corp., Suzhou, China

####### **Correspondence:** Jeffrey Ding (jeffrey.w.ding@gmail.com)

**Background**

The role of circulating cell free DNA holds great promise for individualized medicine for cancer. As it carries information on DNA from cells exfoliated in urine and from circulation, urinary cell free DNA (UcfDNA) is believed to have the potential of being a useful and ultra-noninvasive tool for cancer screening, diagnosis, prognosis, and monitoring of cancer progression and therapeutic effect [1]. However, compared with the widely studied cell- free DNA in blood, less is known about the role of UcfDNA. The presence of UcfDNA signals from tumor has remained controversial, possibly due to the lack of appropriate method and technology to robustly extract and detect the potentially highly degraded UcfDNA.

**Methods**

**Image 1 (abstract P34).**
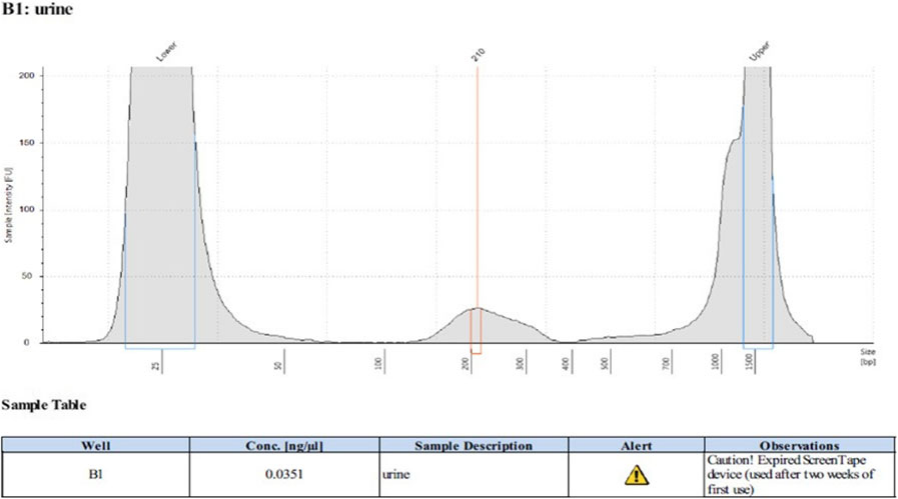
See text for description.

**Image 2 (abstract P34).**
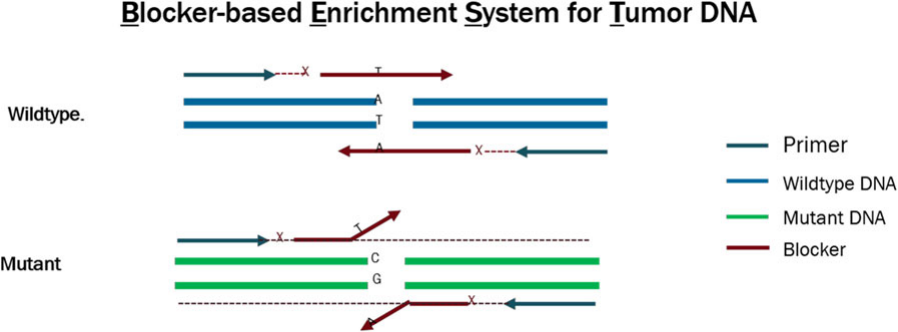
See text for description.

We developed and optimized UcfDNA extraction method from urine samples, and compared UcfDNA quantity and quality regarding the urine sampling time and the storage condition. As a proof of concept, we processed urine samples from pregnant women during their second trimester and used real-time quantitative PCR (qPCR) assays to successfully detect UcfDNA from fetal in maternal urine. We subsequently collected urine samples from lung cancer patients and applied the developed methods to extract UcfDNA. The qPCR assay with the blocker-based enrichment method was developed and used for EGFR mutation detection in UcfDNA (Image 1,2). We also explored the possible application of UcfDNA for the microsatellite instability(MSI) testing.

**Results**

**Image 3 (abstract P34).**
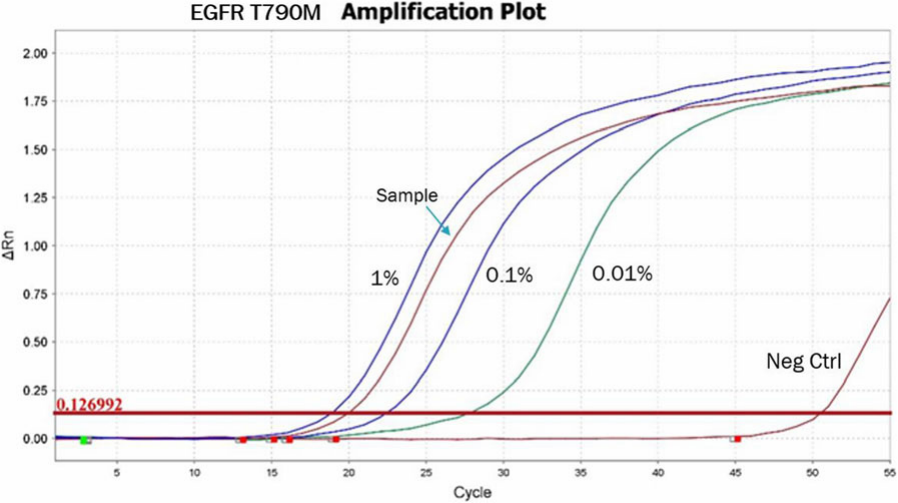
See text for description.

We successfully detected EGFR mutations in UcfDNA which also found in the corresponding tumor tissue samples, demonstrating the UcfDNA extraction and genotyping was possible from urine samples of cancer patients (Image 3).

**Conclusions**

We have confirmed the existence of UcfDNA from tumor, and have shown that UcfDNA could be used for noninvasive cancer genetic test and cancer research.

**References**

1. Lu T, Li J. Clinical applications of urinary cell-free DNA in cancer: current insights and promising future. Am J Cancer Res. 2017 Nov 1;7(11):2318-2332.

#### P35 Development of biomarkers to assess adenosine generation & activity in support of clinical trials conducted with the adenosine receptor antagonist AB928

##### Daniel DiRenzo, PhD, Joanne Tan, PhD, Devika Ashok, PhD, Amy Anderson, PhD, Jenna Jeffrey, PhD, Lisa Seitz, MSc, Manmohan Leleti, PhD, Steve Young, PhD, Jay Powers, PhD, Matthew J. Walters, PhD

###### Arcus Biosciences, Hayward, CA, USA

####### **Correspondence:** Matthew J. Walters (mwalters@arcusbio.com)

**Background**

The high levels of adenosine (ADO) found in the tumor microenvironment have been shown to inhibit immune responses through activation of the A2aR and A2bR receptors on immune cells. The extracellular enzymes ecto-5’- nucleotidase (CD73) and tissue non-specific alkaline phosphatase (TNAP) catalyze the extracellular conversion of adenosine monophosphate (AMP) into ADO. We have previously shown that AB928, a dual A2aR/ A2bR antagonist, blocks the immunosuppressive effects of ADO in human cell culture systems and in mouse syngeneic tumor models. Herein, we describe the development of assays to measure the expression and activity of adenosine- generating enzymes in human tumor samples and peripheral blood. These assays are being implemented in ongoing clinical trials with AB928, to identify tumor types and patients most sensitive to adenosine receptor antagonism.

**Methods**

Gene expression data were extracted from The Cancer Genome Atlas (TCGA). To correlate protein and gene expression levels, immunohistochemistry (IHC) and NanoString analyses were performed on serial sections of formalin fixed paraffin embedded (FFPE) tumor tissue. Circulating levels of CD73 were quantified with an in-house developed CD73 ELISA and total AMP-ase enzymatic activity in plasma was determined using an AMP-Glo assay.

**Results**

TCGA gene expression analysis identified non-small cell lung, renal clear cell, triple-negative breast, ovarian, colorectal, and gastro-esophageal cancers as tumors that express high levels of adenosine processing enzymes. Specifically, combined CD73 and TNAP levels were highest in lung adenocarcinoma (CD73 = 4.024; TNAP = 4.753) whereas colorectal (CD73 = 4.101; TNAP = 0.7493) and ovarian (CD73 = 2.173; TNAP = 5.288) cancers were heavily biased towards CD73 or TNAP, respectively. IHC on human FFPE tumor microarrays demonstrated that NSCLC had the highest CD73 protein levels with a stark contrast between adenocarcinoma (70.5 +/- 17.3 μM2) and squamous cell carcinoma (7.3 +/- 1.9 μM2). In contrast, prostate cancer had low CD73 gene expression (median TPM = 1.82) and protein levels (0.99 +/- 0.07 μM2). Overall, there was strong agreement between TCGA data and IHC (R2 = 0.793) suggesting that transcript levels of CD73 broadly predict local protein levels. In addition, we are in the process of determining the relationship between tumoral CD73 levels and, using different methodologies, peripheral CD73 protein levels and enzymatic activity.

**Conclusions**

Collectively, these assays provide a detailed picture of the capacity of individual human tumors to generate adenosine and should enable the correlation of this information with peripheral activity/levels of adenosine- generating enzymes and potentially the clinical benefit of AB928.

#### P36 Defining the expression of Programmed Death-Ligand 2 in high grade glioma tumor microenvironment

##### Gifty Dominah^1^, Victoria Sanchez, BS^1^, John Lynes, MD^1^, Nicholas Adamstein^2^, Arnold Obungu, BS, BA^3^, Xiang Wang, MS^1^, Nancy Edwards, BA^1^, Edjah K. Nduom, MD^1^

###### ^1^NINDS/NIH, Bethesda, MD, USA; ^2^Columbia University College of P&S, New York, NY, USA; ^3^Indiana University School of Medicine, Indianapolis, IN, USA

####### **Correspondence:** Edjah K. Nduom (edjah.nduom@nih.gov)

**Background**

Checkpoint blockade with anti-programmed death-1 (PD-1) therapy has been demonstrated as a promising treatment for many systemic cancers. Tumor programmed death-ligand 1 (PD-L1) expression has been shown to increase the response to anti-PD-1 immunotherapy in many tumor types. We have recently validated PD-L1 expression as a negative prognostic factor in high grade gliomas (HGG). However, PD-1 has two ligands, PD-L1 and PD-L2, and PD-L2 expression has not been characterized in HGG tissue. Accordingly, the potential prognostic and/or predictive value of PD-L2 has not yet been assessed. This study aims to establish reliable means to detect PD-L2 expression in HGG patient samples, validate this expression and define its clinical relevance.

**Methods**

The PD-L2 antibody clone 24F.10C12 was optimized for staining by immunofluorescence (IF) in PD-L2-plasmid transfected HEK293 cells. Further antibody validation was performed via immunohistochemistry (IHC), IF staining, and RNAscope in situ hybridization (ISH) using normal brain slides and human heart tissue as a positive control. After antibody validation, immunohistochemistry and RNAScope ISH was used to evaluate PD-L2 protein and mRNA expression in paraffin-embedded HGG slides.

**Results**

PD-L2 expression using clone 24F.10C12 was found in transfected HEK293 cells, but not in untransfected cells. By IHC, IF, and ISH, PD-L2 was expressed adjacent to blood vessels in normal brain and HGG slides. There was heterogeneity between HGG patient tumor samples as some tissues also had perinuclear expression of PD-L2. Moreover, HGG tissues that expressed low grades of PD-L1 showed high PD-L2 expression grades per IHC suggesting an inverse relationship between the two.

**Conclusions**

PD-L2 expression in HGG has a distinct cellular pattern and distribution, separate from the expression of PD-L1. PD-L2 expression should be further characterized in these tissues to determine the potential prognostic or predictive value of this marker for immune therapy of HGG patients.

**References**

1. Pratt D, Dominah G, Lobel G, Obungu A, Lynes J, Sanchez V, ... Maric D. Programmed death ligand 1 ss a negative prognostic marker in recurrent isocitrate dehydrogenase-wildtype glioblastoma. neurosurgery. 2018.

#### P37 Multiplexed biomarker quantification to assay and characterize T-cell activation

##### Shilan Dong, MS, Jason Cahoon, BS, Rachit Ohri, PhD

###### Enable Life Sciences LLC, Worcester, MA, USA

####### **Correspondence:** Rachit Ohri (rachit@enablelifesciences.com)

**Background**

Multiplexed biomarker based characterization has proven effective for disease diagnosis and therapeutics development [1]. We adapted a similar multiplexed biomarker strategy for characterizing T-cell activation, with the goal of developing a standardized cell-culture assay for immuno-oncology applications. The panel of chosen biomarkers expressed by T-cells included: [a] IL-2, important for downstream immune activation [b] TNF⍺, important in acute phase immune cell activation, differentiation and migration [c] IL-10, with the role of tumor-specific immune surveillance and mitigating pathologic inflammation, and [d] IFNγ, with the role of tumor- protection through immune cell activation including dendritic cells.

**Methods**

Activation of T-cells was induced by ionomycin and PMA (phorbol 12-myristate 13-acetate) [2] (Sigma-Aldrich, St. Louis). Jurkat cells [UMass, Worcester at 1X106 cells/ml] and primary CD3-positive pan T cells [iXCells Biotechnologies, San Diego at 8X105 cells/ml] were cultured in 24 well plates in RPMI 1640 media (10% FBS and 1% Penn-Strep). 24 h protein-level expression (in the cell supernatant) of IL-2, IFNγ, TNFα, and IL-10 was quantified in response to a wide concentration range for both activators (250 - 2000 ng/ml for ionomycin and 10 - 100 ng/ml for PMA). For the specific combination of 2000 ng/ml ionomycin and 50 ng/ml PMA, time-course expression levels were also determined over a 0h - 56h period. Biomarker quantification was pursued using Luminex methodology.

**Results**

Our multiplexed biomarker data indicates significant overall consistency of expression trends comparing one biomarker to another, though minor differences does occur (eg. the optimal time point for activation), even though comparative concentration levels varied. Additionally, consistency was also observed between the activation profiles of primary CD3+ pan T-cells and the Jurkat T-cell line for [a] the most synergistic combination of ionomycin and PMA for T-cell activation [i.e. 2000 ng/ml ionomycin and 50 ng/ml PMA] (IL-2 expression levels statistically higher i.e. p<0.05 or equal compared to any other combinations of PMA and ionomycin) (Figures 1A, 1B), as well as [b] the time-course of T-cell activation [i.e. biomarker plateauing or high expression most commonly in the 8h - 48h window] (Figures 2A-D). Despite overall consistency, nuanced differences were observed between primary T- cells and Jurkat cells (e.g. timing of the peaking or plateauing of individual biomarker expression), which may further vary between different sources of primary T-cells (to be evaluated).

**Conclusions**

In conclusion, our results provide the basis of a robust, standardized, sensitive and efficient assay for pan T-cell activation.

**Acknowledgements**

The authors acknowledge and thank MBI (Massachusetts Biomedical Initiatives), WPI (Worcester Polytechnic Institute), and University of Massachusetts Medical School for the use of their facilities.

**References**

1. Wagner JA. Strategic approach to fit-for-purpose biomarkers in drug development. Annu. Rev. Pharmacol. Toxicol. 2008; 48:631-651. 2. Manger B, Weiss A, Weyand C, Goronzy J, Stobo JD. T cell activation: differences in the signals required for IL 2 production by nonactivated and activated T cells. The Journal of Immunology. 1985; 135(6):3669-3673.

**Fig. 1 (abstract P37).**
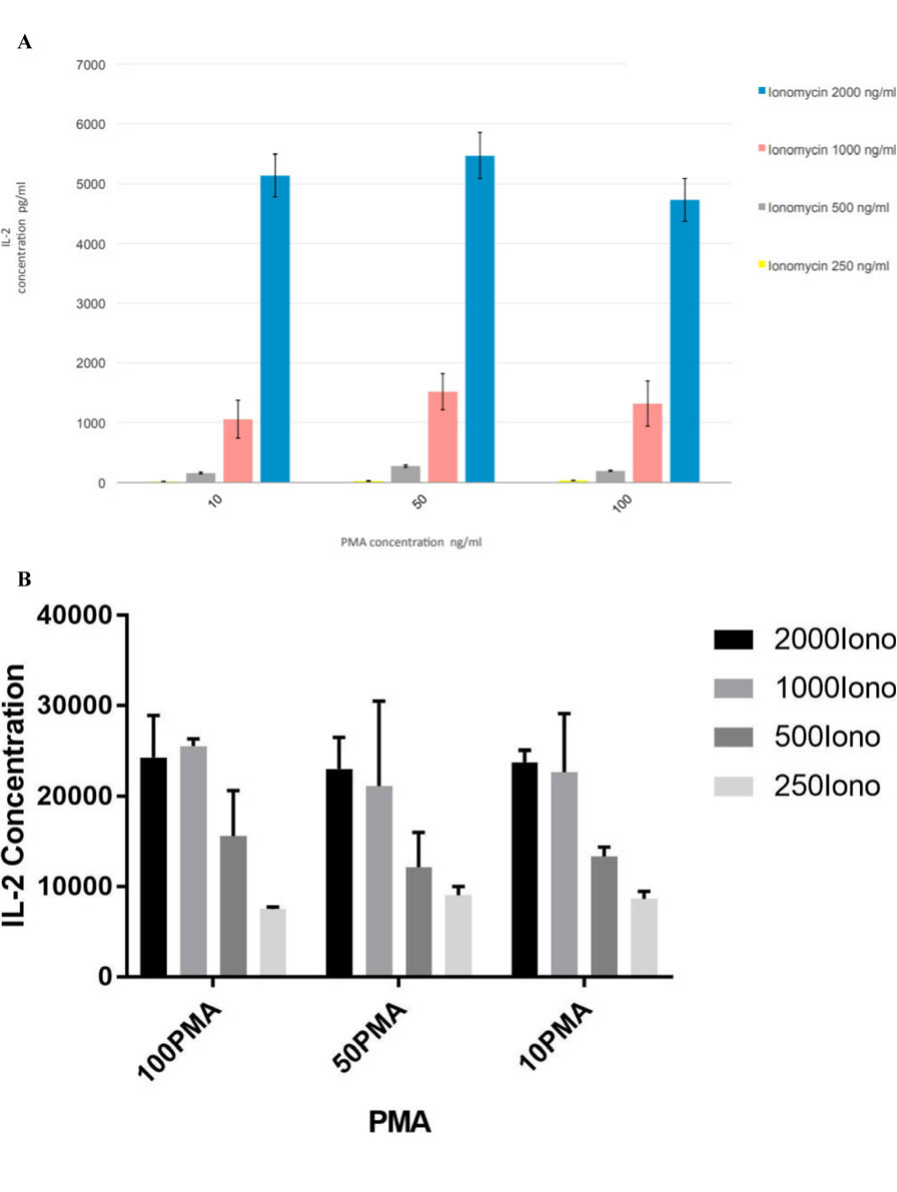
See text for description.

**Fig. 2 (abstract P37).**
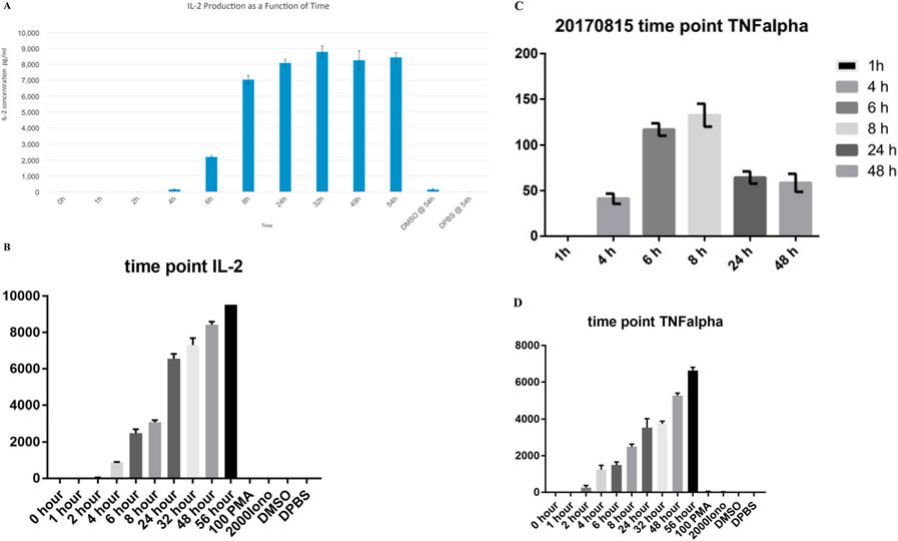
See text for description.

#### P38 Highly multiplex spatial immuno-profiling in FFPE tumor tissue with InSituPlex technology

##### Abdul Mohammed, PhD, Gourab Chatterjee, PhD, Kevin Hwang, PhD, Julie Xia, Amanda Bares, PhD, Michael Murphy, Eloise Wheeler, Armen Changelian, Katir Patel, PhD, Bonnie Phillips, PhD, Sean Downing, PhD, Mael Manesse, PhD

###### Ultivue, Cambridge, MA, USA

####### **Correspondence:** Sean Downing (sean.downing@ultivue.com)

**Background**

Innovative and future translational research tools are key to enabling the full impact of personalized medicine. Current pathology methods rely on chromogenic and H&E staining with low multiplexing capabilities, limiting the depth of information obtained from a single tissue sample. Fluorescence-based tissue staining and analysis enable quantification and higher multiplexing; however, current multiplex immunohistochemistry (mIHC) technologies not only compromise throughput and potentially damage the sample with each round of staining, but also require post- acquisition spectral unmixing. Using InSituPlex technology, a highly multiplexed assay (8-plex) can be carried out in a single work day, with maximum tissue preservation and no spectral unmixing.

**Methods**

InSituPlex technology was used to carry out high-multiplexed immuno-profiling on deidentified FFPE tissue sections. Samples included human tonsil as well as multiple tumor types in skin, lung, and colon. A panel of eight different markers was developed, including CD3, CD8, CD45RO, CD68, PD-1, PD-L1, FoxP3, as well as pan- cytokeratin and Sox10 as tumor markers. Staining of the eight markers was performed in a single run on individual slides, following a manual or automated protocol on the Leica BondRX. Images were acquired using commercially available fluorescence slide scanning platforms, including the Zeiss Axio Scan.Z1, without the need for linear unmixing. All images were analyzed using IndicaLabs HALO.

**Results**

Images from the eight-plex assay on tonsil tissue were compared to individually stained reference samples to confirm the specificity of the markers in the multiplex assay. In tumor samples, abundance of immune and tumor cells was characterized through cell counting. In addition, the different expression levels of PD-L1 in immune and tumor cell types were recorded over the entire section. Phenotyping and spatial distribution analysis was carried out to identify cytotoxic T-cells, memory T-cells, exhausted T-cells, regulatory T-cells, and macrophages on single sections. Reproducibility of the assay was also assessed using the data points from serial sections.

**Conclusions**

InSituPlex technology enables the staining of eight different immune and tumor markers on single FFPE sections, with a streamlined workflow and high reproducibility. Resulting images were then used to perform phenotyping of multiple subsets of T-cell population, macrophages, and tumor cells.

#### P39 PD-L1 expression on tumor versus antigen presenting cells investigated with multiplexed IHC using UltiMapper™ I/O assays

##### Amy Zhang, MS, Alexis Wong, PhD, Max Rubinstein, Laura Sciarra, PhD, Chakib Boussahmain, BS, Bonnie Phillips, PhD, Katir Patel, PhD, Sean Downing, PhD, Stephanie Hennek, PhD

###### Ultivue, Cambridge, MA, USA

####### **Correspondence:** Sean Downing (sean.downing@ultivue.com)

**Background**

In the field of immuno-oncology, there is great promise for improving outcomes by identifying meaningful biomarkers within the tumor microenvironment. The TME is highly heterogeneous, requiring characterization of what cell types are present and what cellular interactions are taking place. Multiplexed IHC is a promising approach to profile cell types and map interactions to identify useful biomarkers. Ultivue has developed UltiMapper assays that provide numerous advantages over other multiplexed IHC approaches including high multiplexing in situ, sample preservation, streamlined workflows, and versatile implementation. The checkpoint marker PD-L1 has become a common marker in immune-oncology, but the usefulness of this marker in insolation has been questioned. In this study, we investigate the expression of PD-L1 on both tumor cells and antigen presenting cells to better understand which cell phenotypes may be important biomarkers.

**Methods**

Multiplexed immunofluorescence was carried out using UltiMapper assays on multiple deidentified FFPE tissue samples, including lung, melanoma, colon, and breast. Each sample was stained and analyzed for the UltiMapper I/O PD-L1 and APC panel using serial sections. The UltiMapper I/O PD-L1 panel included the markers CD8, CD68, PD-L1, pan-cytokeratin, and Sox10. The UltiMapper I/O APC panel included markers CD11c, CD20, CD68, CD163, and MHCII. Staining was performed manually or using the Leica Bond Rx™ autostainer. Imaging was performed on various tissue scanners including the Zeiss Axio Scan.Z1, and image analysis was performed using HALO from Indica Labs.

**Results**

Cell phenotyping was carried out to measure the abundance of PD-L1 co-staining on macrophages, dendritic cells, B-cells, cytotoxic T-cells, and tumor cells. In tumor samples, PD-L1 expression was observed on both immune cells and tumor cells with a range of different expression levels and percent positive cells. Spatial analysis was employed to measure the distances between immune cells of differing phenotypes and tumor cells, which could be used to classify tumor samples hot or cold.

**Conclusions**

Multiplexed IHC is necessary to understand complex cancer biology and the mechanisms by which the immune system is activated or suppressed. The UltiMapper approach is efficient and easy to implement to achieve high quality multiplexed data on a range of tissue types. The UltiMapper I/O PD-L1 panel is complimented by the UltiMapper I/O APC panel to characterize PD-L1 expression on antigen presenting cells and tumor cells. The abundance and location of PD-L1 positive APCs may be a preferred biomarker over tumor cell PD-L1 expression.

#### P40 Intra-assay and inter-assay assessment of reproducibility and quantification of UltiMapperTM I/O PD-1 and PD-L1 immuno-oncology panels for tissue multiplexing

##### Bonnie Phillips, PhD, Katir Patel, PhD, Courtney Hebert, Jamie Buell, Sean Downing, PhD

###### Ultivue, Cambridge, MA, USA

####### **Correspondence:** Sean Downing (sean.downing@ultivue.com)

**Background**

The field of immuno-oncology has enthusiastically adopted multiplex IHC techniques to establish the spatial relationships between various immune cells in tumor biology in context. Multiplexing enables researchers to gain a deeper understanding and insight into the tumor microenvironment. Unfortunately, many of the multiplexing technologies currently utilized in the immuno-oncology field face a number of challenges, specifically in generating highly robust, reproducible, and easily quantifiable data sets. Ultivue’s UltiMapper I/O PD-1 and PD-L1 I/O kits that utilize InSituPlex (ISP) technology, a new method of multiplexed immunohistochemistry (IHC) that utilizes streamlined single antigen retrieval, staining, elongation, and detection steps allowing for the completion of the assay < 5hr. Here we assess these kits for intra-assay and inter-assay reproducibility and quantification.

**Methods**

Intra-assay reproducibility and quantification was accomplished by manually staining 5 serial sections from three different tissue types (tonsil, melanoma, NSCLC) with one set for each of the UltiMapper PD-1 (CD3, CD45RO, PD-1, CK/Sox10) and PD-L1 (CD8, CD68, PD-L1, CK/Sox10) I/O kits. Inter-assay assessment was determined by staining a single slide from a set of serial sections of each tissue type described above once a week for 5 consecutive weeks. Images were acquired using the Zeiss Axio Scan.Z1, without the need for linear unmixing allowing for direct whole slide imaging. Analysis was accomplished using IndicaLabs HALO software. Coefficient of variations (CV) were calculated based on resulting data.

**Results**

Analysis of intra-assay serial section images revealed that cell counts from section to section were within a CV of <10% across all markers, in all tissues, for both the UltiMapper PD-1 and PD-L1 kits. This included total cell counts, top 10% brightest cells, and all quartiles of cell counts based on fluorescence signal intensity. Similar results were seen for inter-assay comparisons over 5 weeks for both kits (<10% CV).

**Conclusions**

The results presented here indicate that InSituPlex technology is potentially much more reproducible than other tissue multiplexing techniques currently available, such as TSA. Histological standards for coefficient of variations in IHC based assays typically are <15%. Data presented here falls well within that standard indicating the potential for future translational applications. In conclusion, InSituPlex is a highly reproducible and quantifiable multiplexing staining technology across a variety of tissue types and markers, within a single run and over time.

#### P41 Characterization of circulating biomarkers in subjects with NSCLC using data independent acquisition mass spectrometry reveals host immune response mechanisms

##### Nicholas Dupuis, PhD, Jakob Vowinckel, PhD, Daniel Heinzmann, Claudia Escher

###### Biognosys, Schlieren, Switzerland

####### **Correspondence:** Nicholas Dupuis (nicholas.dupuis@biognosys.com)

**Background**

Identification of circulating biomarkers in cancer has proven utility in applications for early detection, differential diagnosis, predicting pre-treatment response to therapy, and treatment monitoring. More recently, circulating proteomic biomarkers have been evaluated as surrogate endpoints for early indication of benefit for immunotherapies. This last application is especially relevant during immunotherapy development where the optimal endpoint, overall survival (OS), can take longer to mature. Here, we present an unbiased survey of the circulating proteome of subjects with NSCLC to identify candidate biomarkers which may have utility in multiple stages of patient care.

**Methods**

Unbiased, data-independent acquisition (DIA) mass spectrometry was used to analyze plasma samples from subjects with Stage III-IV non-small cell lung cancer (NSCLC, n = 15) and age matched healthy donors (n = 15), enabling simultaneous sequencing and quantification of plasma proteins. Samples were prepared for mass spectrometry and spiked with a panel of standards covering 500 plasma proteins. All samples were analyzed using 1 hour gradients on a C18 column coupled to a Thermo Scientific Q Exactive HF mass spectrometer. Data was extracted using Spectronaut (Biognosys) with a sample specific spectral library and statistical analysis was conducted to identify disease associated biomarker candidates. Pathway analysis highlights dysregulated biological functions and predicts upstream regulatory pathways.

**Results**

A protein library was created containing 771 unique proteins. In DIA acquisition, 462 proteins were quantified across all samples. Univariate statistical testing identified 26 dysregulated proteins (20 up-regulated and 6 down- regulated; q-value > 0.05 and log2 fold change > 0.58). Multivariate (PLS-DA) analysis identified c-reactive protein (CRP) and serum amyloid a (SAA1/SAA2), complement C9, S100A8/S100A9, and leucine rich glycoprotein 1 (LRG1) as the most significantly changed proteins across sample groups. Significantly enriched pathways include acute phase response, complement system as well as IL-12 and IL-6 signaling. Similarly, upstream activated candidate pathways included STAT3, IL-6, and EZH2.

**Conclusions**

26 proteins were identified as candidate biomarkers and reflect the host immune response via acute phase response signaling, innate immune response (complement system), and other proinflammatory stimuli. Several of these markers have been linked to patient outcomes and poor prognosis. Accurate monitoring these proteins offers the possibility to define surrogate, molecular based, markers with multiple modes of utility.

#### P42 Expanding insights into the colorectal cancer tumor proteome; unbiased protein profiling reveals multiple proteomic-based tumor subtypes

##### Jan Muntel, Roland Bruderer, Nicholas Dupuis, PhD, Lukas Reiter

###### Biognosys, Zurich, Switzerland

####### **Correspondence:** Nicholas Dupuis (nicholas.dupuis@biognosys.com)

**Background**

Recent approvals of microsatellite instability (MSI) and PD-L1 testing, have expanded the tools available to identify tumor characteristics which predict patient responses to immunotherapies. However, even in MSI and PD-L1 positive subgroups, not all subjects achieve a durable response and work continues to identify tumor characteristics that will further predict the likelihood of patient response. To support and advance this area of research, new tools are being developed that provide deeper and unbiased views of the tumor proteome. Here, we characterize the protein expression profiles of 95 colorectal cancer tumors (CRC) using SWATH acquisition mass spectrometry (SWATH MS) to probe tumor phenotypic characteristics.

**Methods**

FFPE colon tissue samples (95 cancer, 10 healthy) from subjects with colorectal cancer across seven regions of the colon: cecum (16), ascending (17), right hepatic flexor (2), left splenic flexor (5), descending (12), sigmoid (21), nonspecific (22). Proteins were extracted from the tissue, processed to peptides, and injected on a Triart C18 column (YMC) coupled to a NanoLC 425 system (SCIEX). Eluted peptides were then analyzed with a TripleTOF® 6600 system (SCIEX) operated in SWATH mode. Data were analyzed in Spectronaut Pulsar X (Biognosys) with a project specific library.

**Results**

Across all samples, >4,500 protein groups were quantified (approximately 3,600 per sample). Data analysis revealed a large number of proteins (~1,000) were differentially expressed in the cancer cohort, including an elevation of proteins involved in translation which is consistent with increased tumor cell proliferation. Unsupervised clustering of the data separated the healthy and the cancer cohort and revealed three main proteomic subtypes within in the cancer cohort (A, B and C) which were largely distinguished by expression of cell adhesion proteins, including neuronal growth regulator 1 (NEGR1), a potential tumor suppressor. Interestingly, hepatocyte nuclear factor 4-alpha (HNF4A), a transcription factor which is known to be elevated in CRC, was most significantly overexpressed in subtype B, which correlates with protein signatures from MSI high samples from previous studies. Additional analysis of key protein networks related to CRC and MSI high status, as well as analysis of the mismatch repair proteins MSH2 and MSH6 expression, will be presented from this work.

**Conclusions**

High-throughput proteomic profiling of FFPE tissues using SWATH-MS enables the deepest phenotypic characterization of tumor tissue. Through global profiling, these analyses will help improve the functional understanding of the interplay between the expression of protein networks, tumor microenvironment, and response to immune-directed therapies.

#### P43 The presence of exhausted CD8+ T cells identifies a subset of immunogenic ER+ breast cancer patient tumors

##### Colt Egelston, PhD, Christian Avalos, Diana Simons, Min Hui Lim, Peter Lee, MD

###### Beckman Research Institute, City of Hope, Duarte, CA, USA

####### **Correspondence:** Colt Egelston (cegelston@coh.org)

**Background**

Estrogen receptor positive (ER+) breast cancers are generally thought to be less immunogenic and less immune infiltrated than triple negative breast cancer (TNBC). In TNBC the presence of tumor infiltrating lymphocytes (TILs) is predictive of response to chemotherapy and associates favorably with patient survival. However, in ER+ breast cancer the relationship between T cell infiltration and disease is less clear. Expression of both PD-1 and CD39 on exhausted CD8+ T cells has been described in murine models of chronic disease and recently human carcinomas. Here we profile human breast tumors for the frequency and phenotype of exhausted CD8+ T cells and their association with a unique immunogenic tumor microenvironment.

**Methods**

Fresh surgical tumor specimens were obtained from consented patients. Single cell suspensions were analyzed by flow cytometry for immune phenotyping and functional assessment of cytokine production by T cells. Single cell sorted T cells were subjected for whole transcriptome RNA sequencing and bulk sorted T cells were submitted for T cell receptor repertoire sequencing by Adaptive Biotechnologies. Formalin fixed tissues were then used for multispectral immunohistochemistry and RNA transcript analysis by Nanostring.

**Results**

Of 35 ER+ tumors assessed, 20% were found to have exhausted PD-1+ CD39+ CD8+ T cells at a significant frequency of total CD8+ TILs. On the contrary, 60% of TNBC tumors assessed contained exhausted PD-1+ CD39+ CD8+ T cells. These T cells showed a significant reduction of IFNγ, TNFα, and IL-2 production capacity. Further phenotyping of exhausted CD8+ T cells revealed a loss of expression of both the IL-7 receptor alpha (CD127) and KLRG1, suggesting terminal differentiation of these cells. Single cell and T cell receptor sequencing revealed distinct transcriptional and clonal signatures of PD-1+ CD39+ CD8+ T cells suggesting them to be a unique population in response to tumor antigen. Immunohistochemistry of tumor tissues showed that ER+ tumors with PD- 1+ CD39+ CD8+ T cells were significantly more infiltrated with T cells and showed characteristics of ‘hot’ tumors. Finally, Nanostring analysis of RNA transcripts from tumor tissues revealed that ER+ tumors containing PD-1+ CD39+ CD8+ T cells had a significantly higher expression of gene signatures of inflammation and IFNγ signaling.

**Conclusions**

This work suggests that a subset of ER+ breast cancer patients have inflamed, highly lymphocyte infiltrated tumors. These tumors are associated with the presence of exhausted CD8+ T cells, suggesting a tumor antigen driven response in these tumors and that these patients may benefit from immunotherapeutic interventions.

#### P44 Discovery and screening of protein biomarkers with the FirePlex Technology Platform

##### Timothy Erps, Amy Perea, PhD, Russell Neuner, PhD, Bianca Heinrich, PhD, Wayne Austin, Conor Rafferty, PhD, Matt Camilleri, Long To, James Murray, PhD, Daniel Pregibon, PhD, Elnaz Atabakhsh, PhD

###### Abcam, Inc, Cambridge, USA

####### **Correspondence:** Bianca Heinrich (bianca.heinrich@abcam.com)

**Background**

In patients and animal models, molecular biomarkers are used as indicators of normal and pathogenic processes. In drug discovery and screening pipelines, molecular biomarkers are used to assess the mechanism of action, efficacy, and toxicity of lead compounds. To address the need for rapid and sensitive quantitation of protein biomarkers, we have developed the FirePlex® Technology Platform. Utilizing patented hydrogel particles and a three-region encoding design, FirePlex Immunoassays allow for true, in-well multiplexing, providing flexible and customizable quantification of analytes.To facilitate biomarker discovery studies, we offer our standard-throughput FirePlex Immunoassays, which enable quantitation of up to 75 protein analytes per sample, from only 12.5 μl of input. These assays demonstrate 5 logs of dynamic range and sub-picogram/ml sensitivity, allowing for highly sensitive quantitation of analytes in serum, plasma, cell culture supernatant, urine, and saliva. Assays are run in 96well plate format, with readout on standard flow cytometers.

**Methods**

For drug discovery and screening studies, we offer our high-throughput FirePlex (FirePlex®-HT) Immunoassays for quantitation of up to 10 protein analytes per sample from only 6.25 μl biofluid input, in 384-well plate format. FirePlex-HT assays provide 3-4 logs dynamic range, demonstrate 1-100 pg/ml sensitivity, and have been validated in serum, plasma, and cell culture supernatant. The two-step workflow, no-wash assay format, and readout on high- content imagers limit hands-on time and are amenable to automation, thus making FirePlex-HT ideally suited for high-throughput screening studies.

**Results**

Here we present data from studies investigating cytokine profiling in human and rodent samples using the FirePlex immunoassays, and introduce the simplified workflow of the FirePlex-HT® immunoassays with data demonstrating the performance for quantifying key cytokines in multiplex, in biological samples.

**Conclusions**

Together, this novel combination of multiplexed, high-sensitivity assays and bioinformatics tools enables rapid quantitation of protein biomarker signatures in biofluid specimens.

#### P45 Tumor infiltrating T cells: complete workflows allow faster and improved analysis

##### Cesar Evaristo, PhD, Ramona Siemer, BTA, Philipp Steinbrueck, Zhongjie Yu, David Agorku, BS, Olaf Hardt, PhD, Christian Dose, PhD, Anne Richter, PhD

###### Miltenyi Biotec, Bergisch Gladbach, Germany

####### **Correspondence:** Cesar Evaristo (cesare@miltenyibiotec.de)

**Background**

Immunotherapies engaging T cells have proven clinical efficacy and tremendous potential. However, responses are often suboptimal. Further research is required to understand tumor-infiltrating leukocytes (TILs) biology and enhance outcomes. TIL analysis is technically challenging and labor intensive. Their number can be very low and small subpopulations might escape analysis as they get lost in the background noise. Importantly, tumor-infiltrating T cells are embedded in a highly immunomodulatory environment such that unbiased cell-intrinsic functional characterization is hindered. When working with large cohort sizes, even immunophenotyping TILs by flow cytometry is time consuming and data processing is laborious. Therefore, it is fundamental to use effective tools to streamline the workflow and to generate reliable data.

**Methods**

We established complete workflows combining tissue storage, dissociation, T cell isolation and phenotyping of mouse and human tumors. Tissues were processed immediately or stored in a solution that was shown to maintain cell viability and phenotype up to 48h after collection (Tissue Storage Solution™). Tumor dissociation was automated and optimized for epitope preservation using a tissue dissociator (gentleMACS™ Octo). We developed new T cell-specific enrichment reagents for magnetic cell sorting, incorporating novel technology enabling the removal of both superparamagnetic beads and antibody fragments (REAlease®). We labeled TILs using recombinant antibodies engineered to eliminate Fc receptor-mediated background (REAfinity™). Finally, flow cytometric analysis was performed using an automated analyzer (MACSQuant X™ and MACSQuant 16™).

**Results**

Optimal enzymatic dissociation was essential for analysis of critical tumor-specific sub-populations, such as PD1hiTim3+Lag3+CD39+CD8+ T cells present in tumors. Magnetic cell sorting resulted in enrichment of rare tumor infiltrating T cells by up to 500-fold, while maintaining activation status and phenotype. Isolation of T cells using REAlease technology allowed enrichment of subpopulations such as gamma delta T cells and Tregs with high purity. Use of REAfinity antibodies significantly diminished non-specific labeling of cells present in the tumor microenvironment. Use of MACSQuant analyzers decreased hands-on as well as total acquisition time by facilitating fast and fully automated sample processing, including sample mixing and absolute cell counting.

**Conclusions**

We optimized workflows that include standardized processing of tumor samples, newly developed tools for (semi-) automated magnetic isolation of tumor infiltrating T cells and automated flow cytometric analysis. These workflows greatly reduce experimental time and allow the performance of more complex experimental setups. We believe the use of these innovative tools and workflows can significantly increase the quality of the data obtained in immuno- oncology and immunotherapy research.

#### P46 Withdrawn

#### P47 Immune response in patients treated with autologous dendritic cells transduced with AdGMCA9 (DC- AdGMCAIX) in patients with mRCC from the phase I, open label, dose escalation and cohort expansion study

##### Izak Faiena, MD^1^, Nazy Zomorodian^1^, Beata Berent-Maoz^1^, Ankush Sachadeva^1^, Adrian Bot, MD, PhD^2^, Fairooz Kabinnavar^1^, Jonathan Said^1^, Gardenia Cheung-Lau^1^, Jia Pang^1^, Mignonette Macabali^1^, Tinle Chodon^1^, Xiaoyan Wang^1^, Paula Cabrera^1^, Paula Kaplan-Lezco^1^, Sandy Liu^1^, Begonya Comin-Anduix, PhD^1^, Allan Pantuck, MD^1^, Arie Belldegrun, MD, FACS^1^, Karim Chamie, MD^1^, Alexandra Drakaki, MD,PHD^1^

###### ^1^UCLA, Los Angeles, CA, USA; ^2^Kite Pharma, Santa Monica, CA, USA

####### **Correspondence:** Izak Faiena (ifaiena@mednet.ucla.edu)

**Background**

Patients with metastatic RCC were treated in a phase I trial with autologous dendritic cells transduced by a replication deficient adenovirus comprised of GM-CSF+CAIX. Nine patients in three dose escalation cohorts (5, 15, and 50 X 106 cells/administration) were injected based on a 3+3 design.

**Methods**

An enzyme-linked immunospot (ELISpot) assay was used to determine the frequency of CAIX-specific IFN-γ producing T cells in blood. 15-mer overlapping peptides from CAIX protein, AdV5-pepton, and controls (+/-) were plated in Elispot plates pre-coated with anti-IFN-γ antibody. Subsequent to assay development, the number of T- cells responding to CAIX was calculated as above the lower limit of detection (LLD) (7 spots). After subtracting the backgrounds, fold change was calculated with respect baseline. The criterion for positive immunological response was defined as the mean fold change plus two. Further assessment included immunohistochemistry (IHC) staining of tissue from patients #4 (with PD) and #8 (with SD) for CAIX, CD4/8, Ki67, GrZ8, PD1/L1. The samples were scored based on percent positivity and staining intensity (Table 1). Tissue was obtained from the primary tumor prior to vaccination, and the target tumor at the end of the study period (18 months).

**Results**

ELISpot showed consistently positive responses against CAIX upon vaccination with DC vaccine, more prominently in patients in cohort 3 (high dose) as well as in those with longer time to progression (Figure 1). None of the treated patients showed an objective response. However, patient #8 who achieved stable disease (SD) lasting 18 months had more than 2-fold change in immune response over baseline on day 35 and 60 after the first vaccination cycle. All nine patients showed different degrees of immunological reaction to AdV5 at baseline and elevation at the end of the study. IHC showed that both patients had high CAIX expression in primary tumor and on the target lesion post vaccination. Immune infiltrates were seen at baseline in both subjects, with predominant CD4/8 T-cells in patient #8 with a high PD-1 expression in infiltrating lymphocytes without PD-L1 expression in the tumor environment.

**Conclusions**

DC-AdGMCAIX vaccination may elicit robust immunologic response against CAIX in patients with ccRCC. The findings of high PD-1 expression in the patient with SD in both the primary tumor and target lesion warrants future efforts to explore how combination therapies with biological response modifiers may further enhance clinical responses.

**Acknowledgements**

Supported by NCI RAID Initiative NSC 740833 and Kite Pharma

**Trial Registration**

NCT01826877

**Ethics Approval**

The study was approved by UCLA institutional review board IRB#12-000577

**Fig. 1 (abstract P47).**
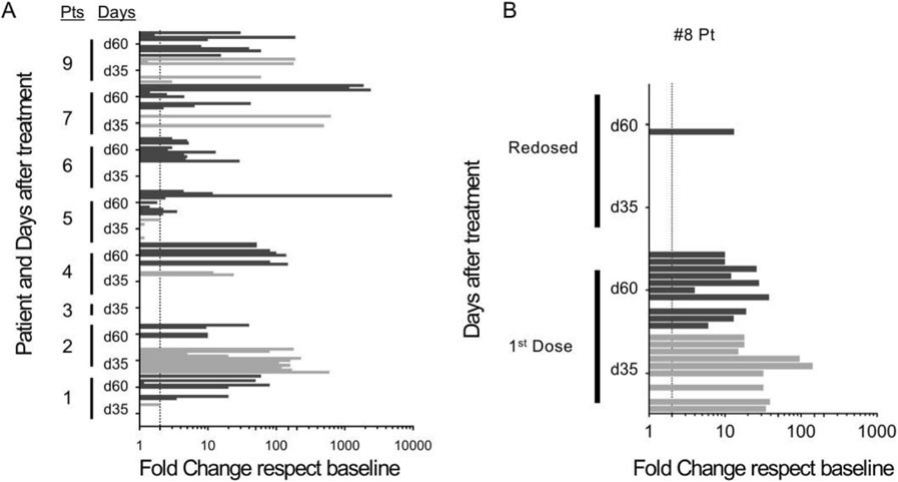
ELispot immune response in study subjects

**Table 1 (abstract P47).**
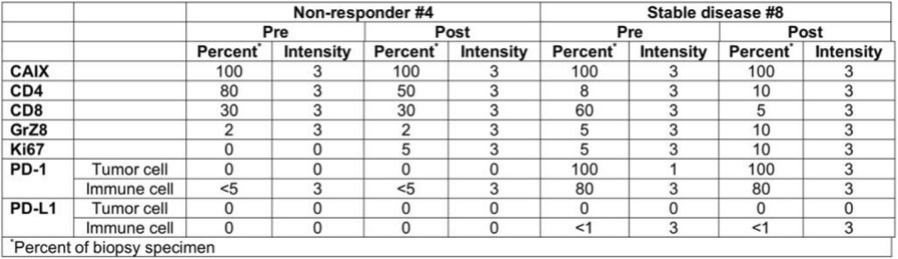
Immunohistochemistry staining

#### P48 A high baseline neutrophil-to-lymphocyte ratio in patients receiving anti-PD-1 therapy for head and neck cancer associates with poor prognosis, tumor hypoxia, and tumor M2 macrophage predominance

##### Corey Foster, MD^1^, Riyue Bao, PhD^2^, Sara Kochanny, BA^1^, Arun Khattri, PhD^1^, Rajesh Acharya, MS^1^, Allison Dekker, RN^1^, Yi-Hung Carol Tan, PhD^1^, Elaine Klema, BS^1^, Ryan Brisson, BS^3^, Vassiliki Saloura^1^, Alexander Pearson, MD, PhD^1^, Everett Vokes, MD^1^, Rom Leidner, MD^4^, Hisham Mehanna, PhD^5^, Tanguy Seiwert, MD^1^

###### ^1^The University of Chicago Medicine, Chicago, IL, USA; ^2^The University of Chicago, Chicago, IL, USA; ^3^Oakland University, Rochester, MI, USA; ^4^Providence Cancer Center, Portland, OR, USA; ^5^University of Birmingham, Birmingham, UK

####### **Correspondence:** Tanguy Seiwert (tseiwert@medicine.bsd.uchicago.edu)

**Background**

The associations among a high neutrophil-to-lymphocyte ratio (NLR), prognosis, and the tumor microenvironment for patients with head and neck (H&N) cancer receiving anti-programmed death receptor 1 (PD-1) therapy are not established.

**Methods**

One-hundred-fourteen patients with metastatic H&N cancer received anti-PD-1 therapy with high baseline NLR defined as >8.77 (highest quartile). Logistic regression analyzed the association with overall response rate (ORR), and Kaplan-Meier methods and Cox proportional hazards regression models were used to test the association between NLR group and progression-free survival (PFS) and overall survival (OS). Tissue was available from 60 patients for gene expression profiling and pathway analysis using RNA sequencing. Expression abundance was quantified by Kallisto and normalized using the trimmed mean of M-values normalization method. Genes differentially expressed in the high vs. low NLR group were detected using limma voom with precision weights.

Pathway enrichment and upstream regulator prediction was performed through the use of IPA (QIAGEN Inc.).

**Results**

Median follow-up was 14.3 months. ORR was 22.3% with a trend towards lower ORR for the high NLR group (p=0.20). Median PFS was 1.7 months (95% confidence interval [CI]: 1.0-3.6) for high NLR patients and 3.7 months (95% CI: 3.2-6.2) for low NLR patients (p=0.01). Median OS was 9.6 months shorter in the high vs. low NLR group (3.8 months vs. 13.4 months, p<0.0001), and a decreased NLR 6 weeks into therapy associated with improved OS (p=0.0008). High NLR remained associated with OS (p=0.01) on multivariate analysis and trended toward independent association with PFS (p=0.07). High NLR associated with higher expression of CD163 (p=0.006) and HIF1-alpha (p=0.009), which was further supported by predicted activation of HIF1-alpha-target molecules based on experimental evidence from the Ingenuity Knowledge Base (activation z-score = 2.834, p=1.88E-04).

**Conclusions**

High baseline NLR associates with poor prognosis for patients with metastatic H&N cancer receiving anti-PD-1 therapy and correlates with increased expression of genes related to M2 macrophage predominance and hypoxia within the tumor microenvironment.

**Ethics Approval**

This study was approved by The University of Chicago Institutional Review Board.

#### P49 Comprehensive immune and molecular analysis of a cohort of non-small cell lung cancer (NSCLC) patients treated with a personal neoantigen vaccine, NEO-PV-01, in combination with anti-PD1

##### Joel Greshock^1^, Ramaswamy Govindan, MD^2^, Riley Curran^1^, Rana Besada^1^, Samantha Gates^1^, Victoria Kohler^1^, Meghan Bushway^1^, Julian Scherer, PhD^1^, Ying Sonia Ting^1^, Yuting Huang, Master of Science^1^, Yvonne Ware, Masters^1^, April Lamb^1^, Lisa D.Cleary^1^, Melissa Moles^1^, Richard Gaynor, MD^1^, Matthew Goldstein, MD, PhD^1^, Lakshmi Srinivasan^1^

###### ^1^Neon Therapeutics, Inc; ^2^Washington University Medical School, Saint Louis, MO, USA

####### Joel Greshock (jgreshock@neontherapeutics.com)

**Background**

Neoantigens arise from DNA mutations and are important targets when presented on the surface of cancer cells for tumor-specific T cell responses. Vaccines targeting neoantigens have the potential to induce de novo and amplify pre-existing anti-tumor T cell responses. NEO-PV-01, a personal neoantigen vaccine, designed based on a patient’s tumor-specific mutations and are predicted to be presented by MHC molecules, is comprised of up to 20 long peptides and administered along with an immune adjuvant Poly-ICLC. Here we report comprehensive immune and molecular analysis observed in a cohort of metastatic non-small cell lung cancer (NSCLC) patients treated with NEO-PV-01 in combination with nivolumab (ClinicalTrials.gov: NCT02897765).

**Methods**

Tumor biopsies were scheduled for collection: i) prior to any treatment, ii) after 12 weeks of nivolumab monotherapy and iii) after completion of NEO-PV-01 vaccination. Tumor biopsies from each collection time point were analyzed for multiple immune and tumor markers by immunohistochemistry, gene expression and whole exome sequencing. Immune monitoring from peripheral blood samples was also evaluated at similar times for the presence of antigen-specific responses by IFNγ ELISPOT, intracellular cytokine staining, multi-parameter surface and functional phenotyping by FACS and the presence of cytolytic properties.

**Results**

IFNγ ELISPOT analysis with peripheral blood mononuclear cells (PBMCs) revealed neoantigen-specific CD4+ and CD8+ T cell responses that were only detected in the post-vaccination samples. Vaccine-induced immune responses were durable in one of the patients who reached the week 52 treatment timepoint. Neoantigen-specific T cells were of effector memory and central memory phenotype. Additionally, these cells were cytolytic and secreted IFNγ, TNFα and IL2. Assessment of serial tumor biopsies with repeat exome sequencing, gene expression, TCR repertoire analysis, immunohistochemistry and pathologic analysis will be presented.

**Conclusions**

NEO-PV-01 is immunogenic and leads to durable de novo neoantigen-specific immune responses in the peripheral blood of patients with metastatic NSCLC.

#### P50 Characterization of T cell receptor repertoire from FFPE extracted RNA from glioblastoma patients by anchored multiplex PCR and next-generation sequencing

##### Laura Johnson, PhD^1^, Josh Haimes^1^, Namitha Nair^1^, Angelo Porciuncula, PhD^2^, Kurt Schalper, MD, PhD^2^

###### ^1^ArcherDX, Boulder, CO, USA; ^2^Yale School of Medicine, New Haven, CT, USA

####### **Correspondence:** Laura Johnson (ljohnson@archerdx.com)

**Background**

The adaptive immune system is involved in various disease conditions including cancer, chronic infection, autoimmune disease and transplant rejection. Adaptive immunity is mediated by B and T lymphocytes, which are activated upon antigen binding to antigen receptors expressed on their surface. Therefore, the spectrum of these antigen receptors, or immune repertoire (IR), provides a means to monitor adaptive immune responses to disease, vaccination and therapeutic interventions. Next-generation sequencing (NGS) of antigen receptor genes is a valuable tool in the study of disease states and responses to various interventions. The Archer® Immunoverse™ TCR assay quantitatively assesses the T-cell content and clonal diversity of patient derived RNA. Here, we describe the use of an Anchored Multiplex PCR (AMP)-based NGS assay to analyze RNA extracted from FFPE samples from glioblastoma patients during an immunotherapeutic clinical trial.

**Methods**

In this study RNA was extracted both pre and post treatment, and the effects of the two therapeutic interventions were compared. Testing was performed by two independent labs and with both NGS and NanoString™ based assays.

**Results**

The results of the two different assays showed concordance with the T cell content increasing significantly post immunotherapeutic intervention as detected by the NanoString assay (p=0.003) while the increase was not significant post standard of care treatment (p=0.438). The trend was the same with the Archer® Immunoverse™ assay where a larger increase in T cell clones was detected post immunotherapeutic treatment compared to post standard of care treatment (p=0.037 vs. 0.5275 respectively). The results we present also show that the number of detected TCR clones and clonotypes depended on sample input amount, sequencing depth and RNA quality.

**Conclusions**

The results of this study support the utility of the Archer® Immunoverse™ assay in the study of FFPE derived RNA from patient samples.

#### P51 Objective quantitative measurements of PD-L1 expression in tumor tissue by Phosphor Integrated Dots staining in patients with non-small cell lung cancer

##### Kazuyuki Hamada, MD, PhD^1^, Ryotaro Ohkuma, MD^1^, Takehiro Takahashi, MD, PhD^1^, Takeshi Setogawa^2^, Masaru Takahashi^2^, Hisatake Okada^2^, Hiroo Ishida^1^, Yutaro Kubota, MD, PhD^1^, Hirotsugu Ariizumi, MD PhD^1^, Etsuko Satoh, MD, PhD^1^, Yuya Hirasawa, MD^1^, Yasutsuna Sasaki, MD, PhD^1^, Kiyoshi Yoshimura, MD, PhD^1^, Takuya Tsunoda, MD^1^, Satoshi Wada, MD, PhD^1^

###### ^1^Showa University, Tokyo, Japan; ^2^Konica Minolta, Inc., Tokyo, Japan

####### **Correspondence:** Satoshi Wada (st-wada@med.showa-u.ac.jp)

**Background**

The PD-1/PD-L1 signal suppresses activated T cells. Anti-PD-1 antibody treatment has an anti-tumor effect by blocking the PD-1/PD-L1 signaling, thereby reactivating exhausted T cells, especially tumor antigen-specific T cells. Several clinical studies were reported that the effect of anti-PD-1 antibody was associated with the number of CD8 T cells in tumor tissue. It was also reported that the effect of anti-PD-1 antibody was associated with the expression ratio (%) of PD-L1 in tumor tissue. However, the effect was also shown in cases with low PD-L1 expression ratio (%). Thus, the expression ratio (%) of PD-L1 in tumor tissue has not yet been a clearly predictive biomarker for anti-PD-1 antibody treatment. This may be a result of the pathologist individually measuring the percentage of PD-L1 positive cells in tumor tissue by immunohistochemistry (IHC).

**Methods**

Phosphor Integrated Dots (PID) staining is a highly accurate measurement method that detects the expression of specific molecules with high sensitivity and can be quantified as a particle number. In this study, we performed objective quantitative measurements of PD-L1 expression per unit area of tumor tissue by PID staining (PD-L1 PID score) in five patients with non-small cell lung cancer (three responders and two non-responders) treated with anti- PD-1 antibody. In addition, double staining with PD-L1 and CD8 T cells in tumor tissue was performed by PID and IHC, respectively.

**Results**

PD-L1 expression per unit area of tumor tissue was quantified by PID analysis. We also established double staining of IHC for CD8 T cells and PD-L1 with PID. It was proved that the PD-L1 PID score was significantly associated with the number of tumor-infiltrating CD8 T cells. Additionally, the effective cases of anti-PD-1 antibody tended to have a higher ratio of the PD-L1 PID score and the number of CD8 T cells.

**Conclusions**

PID staining is might be useful for PD-L1 measurement in tumor tissue. By conducting further case analysis and clarifying the relationship between the PD-L1 PID score and immune cells, characteristics of responders treated with anti-PD-1 antibody may be clarified.

**Ethics Approval**

The study was approved by Showa University School of Medicine‘s Ethics Board, approval number 2253.

#### P52 Emergence of an ICOShi CD4 T cell subset correlates with tumor reductions in subjects treated with the ICOS agonist antibody JTX-2011

##### Amanda Hanson^1^, Sean Lacey, MA Biostatistics^1^, Courtney Hart^1^, Ty McClure^1^, Ellen Hooper, MD^1^, Elizabeth Trehu, MD^1^, Deborah Law, DPhil^1^, Christopher Harvey, PhD^1^

###### ^1^Jounce Therapeutics, Cambridge, MA, USA; ^2^Jounce Therapeutics, Inc., Cambridge, MA, USA

####### **Correspondence:** Christopher Harvey (charvey@jouncetx.com)

**Background**

Inducible T cell Co-stimulator (ICOS) is a costimulatory molecule expressed primarily on T lymphocytes that is upregulated upon cell activation. ICOS was identified as a potential target of interest based on clinical data from studies with anti-CTLA-4. Sustained ICOS upregulation was associated with clinical benefit, with preclinical data confirming a role for ICOS signaling in optimal anti-tumor activity. JTX-2011 is a first-in-class ICOS agonist antibody that has been demonstrated preclinically to have a tumor-centric dual mechanism of action through stimulation of CD4 T effector cells and depletion of intratumoral T regulatory cells. Clinical and biological activity of JTX-2011 is currently being evaluated in the advanced solid tumor setting in the ongoing Phase I/II ICONIC trial (NCT02904226).

**Methods**

Relapsed/refractory cancer patients received escalating doses of JTX-2011 as a monotherapy or in combination with nivolumab (240mg) administered q3w. Serial collection of peripheral blood mononuclear cells (PBMCs) was performed to enable longitudinal assessment of biological activity through flow cytometry-based assays, including target engagement (TE), and immunophenotyping (IP) including a limited assessment of T cell exhaustion.

**Results**

At the RP2D, peripheral TE demonstrated sustained (>70%) engagement over the entire dose cycle, and IP data demonstrated no consistently significant changes in T cell populations following JTX-2011 treatment. The potential of sustained agonism to drive phenotypic T cell exhaustion was assessed, and no evidence of phenotypic T cell exhaustion was observed in subjects treated with the combination of JTX-2011 and nivolumab. No conclusions could be reached in monotherapy setting due to limited sample availability. Further analysis of peripheral T cell phenotype demonstrated the emergence of an ICOShi subset of CD4 T cells in select subjects. Interestingly, the emergence of this cell population correlated with tumor reductions in both JTX-2011 monotherapy and combination subjects. Of the evaluable subjects assessed (N=37), emergence of the ICOShi subset was detected in 7/7 subjects with a reduction of their target lesion >30%, but not in any subject with best overall response of progressive disease.

**Conclusions**

Analysis of longitudinal blood samples from subjects treated with JTX-2011 suggests that the emergence of a distinct ICOShi population of peripheral CD4 T cells correlates with a radiographic response to JTX-2011 treatment. The emergence of this population may serve as a surrogate biomarker of response and may be useful in guiding future clinical development.

#### P53 X4P-001, an orally bioavailable CXCR4 antagonist, increases immune cell infiltration and tumor inflammatory status in the microenvironment of melanoma

##### Timothy Henion^1^, Robert Andtbacka, MD, CM, FACS, FRCSC^2^, Robert Pierce, MD^3^, Jean Campbell, PhD^3^, Melinda Yushak, MD, MPH^4^, Mohammed Milhem, MBBS^5^, Merrick Ross, MD^6^, Katie Niland^7^, Lu Gan, MD, PhD^7^, Sudha Parasuraman, MD^7^, Yan Wang, PhD^7^

###### ^1^Acumen Medical Communications, Cambridge, MA, USA; ^2^Huntsman Cancer Institute, Salt Lake City, UT, USA; ^3^Fred Hutchison Cancer Research Center, San Diego, CA, USA; ^4^Emory University School of Medicine, Atlanta, GA, USA; ^5^University of Iowa, Iowa City, IA, USA; ^6^MD Anderson Cancer Center, Houston, TX, USA; ^7^X4 Pharmaceuticals, Cambridge, MA, USA

####### **Correspondence:** Robert Andtbacka (robert.andtbacka@gmail.com)

**Background**

The CXCR4/CXCL12 axis plays a central role in the trafficking of key immune cells in the tumor microenvironment (TME). Enhanced survival is reported in multiple syngeneic mouse models when a CXCR4 antagonist is combined with a check point inhibitor. X4P-001 is an oral, selective, allosteric inhibitor of CXCR4 that robustly inhibits the growth of murine B16-OVA melanoma. A biomarker-driven Phase 1b clinical study (NCT02823405) is on-going in melanoma patients to test the hypothesis that X4P-001 has the potential to modulate the TME in favor of an improved response to checkpoint inhibitors.

**Methods**

The primary objectives for this trial are to evaluate the safety and tolerability of X4P-001 alone and in combination with pembrolizumab in patients with metastatic melanoma and to characterize the effects of X4P-001 on tumor immune cell infiltrates. Serial biopsies of cutaneous or subcutaneous melanoma lesions, peripheral blood mononuclear cells, and serum samples were collected pre-dose, after three weeks of single-agent X4P-001, and after six weeks of combination treatment. Biopsies were assessed by immunohistochemistry and multiplex immunofluorescence for multiple protein markers, including CD8, FoxP3, and Granzyme B; and by NanoString analysis for changes in gene expression. Serum samples were assessed using the multi-analyte profile platform for key chemokines and cytokines.

**Results**

As of June 5th, 2018, 16 patients (median age 74.5 years, range 53-91) have been enrolled and all have completed treatment or are off study. X4P-001 alone increased infiltration of CD8+ T cells, granzyme B signal, antigen- processing and presentation machinery, such as HLA-DR, and both Tumor Inflammatory Signature (TIS) and IFN- gamma gene expression signature scores in the TME of select patients with paired evaluable biopsies. These biomarker responses were further enhanced when X4P-001 was combined with pembrolizumab. Adverse events related to either X4P-001 or pembrolizumab (greater than 10%) were diarrhea, fatigue, maculo-papular rash, dry eye, acute kidney injury, chills, decreased appetite, dry mouth, ocular hyperemia, oral candidiasis, and pruritus. These data, along with measurements of serum chemokines and cytokines, will be presented.

**Conclusions**

Treatment with single-agent X4P-001 consistently results in enhanced immune cell infiltration and activation in the TME leading to increases in TIS and IFN gamma gene expression signature scores. X4P-001 as a single-agent and in combination with pembrolizumab has an acceptable safety profile. These data support the use of X4P-001 to potentially improve outcomes for patients with tumors that are less responsive to checkpoint inhibitors.

**Trial Registration**

NCT02823405

**Ethics Approval**

Institutional Review Board approval was obtained from each participating center.

**Consent**

Each patient provided consent to participate in this clinical trial.

#### P54 Adenosine signature genes associate with tumor regression in renal cell carcinoma (RCC) patients treated with the adenosine A2A receptor (A2AR) antagonist, CPI-444

##### Andrew Hotson, PhD^1^, Stephen Willingham, PhD^1^, Lawrence Fong, MD^2^, John Powderly, MD, CPI^3^, Jason Luke, MD, FACP^4^, Mario Sznol, MD^5^, Saby George, MD, FACP^6^, Toni Choueiri^7^, Marios Giannakis, MD, PhD^7^, Brian Rini, MD^8^, Shivaani Kummar, MD^9^, Erik Evensen^10^, Ian McCaffery, PhD^1^, Chunyan Gu^1^, Long Kwei, PhD^1^, Ginna Laport^1^, Joseph Buggy^1^, Richard A. Miller, MD^1^

###### ^1^Corvus Pharmaceuticals, Burlingame, CA, USA; ^2^University of California, San Fransisco, San Francisco, CA, USA; ^3^Carolina BioOncology Institute, Huntersville, NC, USA; ^4^University of Chicago, Chicago, IL, USA; ^5^Yale University Cancer Center, New Haven, CT, USA; ^6^Roswell Park Cancer Institute, Buffalo, NY, USA ^7^Dana Farber Cancer Institute, Boston, MA, USA ^8^Cleveland Clinic Foundation, Cleveland, OH, USA; ^9^Stanford University School of Medicine, Stanford, CA, USA; ^10^Basis Bioscience, Foster City, CA, USA

####### **Correspondence:** Richard A. Miller (rmiller@corvuspharma.com)

**Background**

Adenosine in the tumor microenvironment is immunosuppressive. CPI-444, an adenosine A2A receptor (A2AR) antagonist, restores immune function and is active in preclinical tumor models. CPI-444 is being investigated as monotherapy and in combination with atezolizumab (Tecentriq®) in an ongoing Phase 1/1b trial in patients (pts) with treatment-refractory renal cell carcinoma (RCC). Biomarker objectives included identifying tumor gene expression pathways associated with tumor response.

**Methods**

Tumor biopsies obtained at trial screening from pts with RCC (n=30) were analyzed for gene expression profiles with the Nanostring PanCancer Immune Panel that included 770 markers of immune activity and inflammation. The gene expression correlation (Spearman) matrix was hierarchically clustered (Ward’s method) to identify modules of genes that were co-expressed across tumors. Gene cluster expression intensity was compared between pts with evaluable best change in tumor size ≤0 (n=8) vs >0 (n=15). The composite adenosine gene signature score was calculated as the average of the Log2 of expression values for seven genes (CXCL1, CXCL2, CXCL3, CXCL5, IL1B, IL8, SERPINB2) shown to be induced in vitro in normal peripheral blood mononuclear cells by adenosine [1].

**Results**

Analysis of baseline RCC tumors revealed a cluster of correlated genes in T cell activation, IFNg-signaling, and antigen presentation pathways; this cluster was not significantly associated with tumor response to CPI-444 monotherapy or combination. However, the gene cluster containing the adenosine gene signature was associated with tumor regression (p=0.02). This cluster included 18 other genes such as chemokines (e.g. CCL20), complement genes, and serum amyloid A1. An additional cluster contained components of the adenosine signature, CXCL3 and CXCL5, and also associated with regression (p=0.04). In contrast, a cluster enriched for growth factor response genes associated with tumor progression (p=0.01) and negatively correlated with the adenosine signature cluster.

Likewise, a cluster containing CX3CL1 and complement inhibition also associated with tumor progression (p=0.04) and inversely with the adenosine signature. These data support the co-regulation of adenosine and other biological processes within the tumor microenvironment, such as a negative relationship between adenosine and both CX3CL1 and growth factor signaling.

**Conclusions**

CPI-444 anti-tumor activity in RCC was associated with baseline expression of adenosine responsive genes, and is consistent with the mechanism of action of CPI-444. Adenosine-mediated immunosuppression and growth factor pathway activation may represent alternative oncogenic processes that define RCC pt subsets and could provide pathway specific biomarkers for prognosis and pt selection.

**Trial Registration**

NCT02655822

**References**

1. Willingham S, et al. Identification of adenosine pathway genes associated with response to therapy with the adenosine receptor antagonist CPI-444. European Society for Medical Oncology poster presentation. Munich, Germany, 2018.

**Ethics Approval**

The protocol was approved by the institutional review board or ethics committee at each participating center.

#### P55 Within the secretome: Immunomodulatory role of extracellular vesicles in breast cancers

##### Sheeba Irshad, MD, PhD^1^, Atousa khiabany, Mres^1^, Fabian Flores-Borja, PhD^1^, Ines García Carcedo^1^, Felix Wong^1^, Fabienne Beuron, PhD^2^, Jose vicencio^3^, Andrew Tutt^1^, Tony Ng^1^

###### ^1^Kings College London, London, UK; ^2^Institute of Cancer Research, London, UK; ^3^University College London, London, UK

####### **Correspondence:** Sheeba Irshad (sheeba.irshad@kcl.ac.uk)

**Background**

Extra-cellular vesicles (ECVs) are heterogeneous submicron-sized vesicles that vary in size, composition and surface biomarkers. Recently, evidence suggests ECVs can have dichotomic role in the regulation of the immune system, enhancing or suppressing an immune response depending on their cell of origin and functional state. We investigated the immunomodulatory functions of breast cancer cell line and patient-derived serum ECVs.

**Methods**

ECVs were isolated from cell line culture media and serum of patients diagnosed with early breast cancers (BC) using ultracentrifugation and gradient column methods, respectively. Isolated ECVs were characterized by nanoparticle flow, electron microscopy and dot blot analyses. ECVs were cultured with human PBMCs with or without ECVs for upto 5 days. Cell phenotypes, and their proliferation and activation states were evaluated by flow cytometry.

**Results**

Breast cancer cell-line ECVs significantly improved the survival of PBMCs in vitro (p=0.005). PBMCs cultured with ECVs had significantly higher CD4+T-cells (p=0.01) but reduced number of CD8+T-cells (p=0.001) compared to unstimulated PBMCs. No significant differences in the B-cell or NK-cell counts were observed. Assessment of the activation status demonstrated that BC cell line-derived ECV inhibited the activation of CD4+T-cells by anti-CD3/anti-CD28 activation beads (p=0.01) but no significant difference was observed in the activation status of CD8+T-cells. No differences in the proliferation rates of CD3+CD4+ or CD3+CD8+ T cells between ECV- stimulated versus no ECV stimulation was observed. Further analysis for the co-expression of CD45RO and CCR7 on CD4+ or CD8+ distinguished between naïve (CD45RO−CCR7+), central memory (CD45RO+CCR7+,Tcm), memory effector (CD45RO+CCR7−, TEM), and effector (CD45RO−CCR7−, Teff) T-cells. PBMC- ECV co- cultures significantly increased the percentage of CD4+ Tcm-cells (p=0.001) and CD8+ Tcm-cells (p=0.001), as compared to the decrease see in the frequency of the CD4+ Teff cells (p=0.001). Significant increase in T-regulatory cells populations in the PBMC-ECV co-culture conditions was observed (p=0.01). The effect of patient-derived serum ECVs on PBMCs in vitro are ongoing but have revealed differences in ECV secretion capacity within BC subtypes (TNBC, HER2+, ER+ ECVs). Functional validation of our preclinical results is awaited.

**Conclusions**

These results suggest that BC cells utilise ECVs to tame immune cells to promote an immunosuppressive microenvironment. The skewed maturation phenotype of T-cells following ECVs stimulation with increase in Tcm-cells suggest an accelerated maturation of naive T-cells. These reported differences in the immunomodulatory function of ECVs require further investigation.

#### P56 New Dextramer technology for T-cell epitope profiling, and single cell deep phenotyping using DNA barcodes, transcriptomic and genetic sequence analysis, on single cells

##### Kivin Jacobsen, PhD^1^, Liselotte Brix, phd^1^

###### ^1^Immudex, Copenhagen, Denmark; ^2^Immudex Aps, Copenhagen, Denmark

####### **Correspondence:** Kivin Jacobsen (kj@immudex.com)

**Background**

Identification of disease-specific T-cell epitopes is key to the development of many novel vaccines and immunotherapies. Profiling disease-specific T cells, emerging during a cellular immune response e.g. in tumor development or destruction, is an important aspect of personalized immunotherapy. The epitope diversity of the human population is large, and the technologies for identifying disease-specific epitopes have been inadequate.We have developed a process in which DNA barcoded Dextramer reagents are used to simultaneously screen for hundreds, or potentially thousands, of T-cell epitopes in a few milliliters of blood. Single cell deep phenotyping is quickly emerging, analyzing both cellular proteins, transcriptome and genetics of single cells by sequence analysis.DNA barcoded Dextramer reagents extend these technologies, to include simultaneous analysis of antigen specific T cells, by single cell sequencing.

**Methods**

We present a process in which DNA barcoded Dextramer reagents (dCODE Dextramer, can be used to simultaneously screen for hundreds, or potentially thousands, of T-cell epitopes in one small patient sample. Similar to previously reported academic methods, CITE-seq and REAP-seq, it is now possible to combine DNA barcoded MHC Dextramer technology, with single cell analysis allowing direct correlation between the specificity of the antigen specific T cell and its cognate T cell receptor protein sequence, which has not been possible with existing technologies.

**Results**

Profiling of antigen-specific T cells, by simultaneous detection of a large numbers of T-cell specificities in the same small cell sample, was performed using DNA barcode MHC Dextramer analysis, followed by mass sequencing. Pools of 50 MHC Dextramer reagents was screened on PBMC samples from human donors, and the analysis could identify relevant antigen-specific T-cell populations. The results were confirmed by Dextramer analysis using conventional fluorochrome based flow cytometry.

**Conclusions**

DNA Barcoded Dextramer reagents in large libraries, allows for, high-throughput screening of antigen-specific T cells in limited sample material.DNA Barcoded Dextramer, together with single cell sequencing technology, can be used for deep T cell phenotyping by barcode sequencing and transcriptomic or genetic sequence analysis, on the single cell level.

#### P57 A rapid multi-color fluorescence imaging on frozen tissues

##### Dinesh Jaishankar, PhD^1^, Cormac Cosgrove, PhD^2^, Caroline Le Poole^2^

###### ^1^Northwestern University, Chicago, IL, USA; ^2^Feinberg School of Medicine, Chicago, USA

####### **Correspondence:** Caroline Le Poole (caroline.lepoole@northwestern.edu)

**Background**

Immunofluorescence (IF) imaging is a commonly performed routine technique. To detect >four molecules that identify different cell types in a single image, multiplex imaging techniques now exist wherein the fluorophores are spectrally separated to avoid overlap [1]. Quantitative multispectral imaging on tissues can provide a wide range of information ranging from predicting response rates of immunotherapies to immune monitoring [2]. Current techniques to perform multispectral imaging are tedious, require expensive kits, and have been developed with paraffin-embedded sections in mind. Here, using readily available fluorophore-conjugated antibodies, and a rapid IF staining protocol, we imaged up to six different colors in a frozen mouse spleen section.

**Methods**

8μm sections of a naïve and frozen mouse spleen were cut, acetone fixed, and stored at -20oC until the staining process. An antibody cocktail comprising of primary-labeled antibodies to CD3, CD8, CD19, CD11b and CD206 was made. For the staining, sections were blocked using the Superblock, incubated with the antibody cocktail for 1hr at RT and coverslips were added with Prolong Diamond mounting medium. The DAPI stain was used to stain the nuclei. Single color and unstained slides were also processed the same way. Image acquisition, under 20x objectives, and the multispectral imaging analysis was performed on the Vectra 3.0 from Perkin Elmer.

**Results**

Using the spectral library generated from the single stained and unstained slides, the multispectral imaging analysis revealed the detection of six different colors on the frozen section.

**Conclusions**

For the first time, we show a rapid and easy method of detecting different markers on a single frozen tissue section.

This method has great potential for immune monitoring studies to measure the number and colocalization of immune and target cells.

**Acknowledgements**

We would like to thank Ryan Deaton, the imaging specialist at the University of Illinois at Chicago for helping with the multispectral imaging.

#### P58 The use of low-coverage sequencing of cell-free DNA for monitoring response to immune checkpoint inhibitors throughout treatment

##### Taylor Jensen, PhD^1^, Aaron Goodman^2^, Shumei Kato, MD^2^, Mina Nikanjam^2^, Christopher Ellison^1^, Gregory Daniels, MD, PhD^2^, Lisa Kim, MS^2^, Kimberly Kelly, MS^1^, Kerry Fitzgerald, PhD^1^, Erin McCarthy^1^, Prachi Nakashe, MS^1^, Amin Mazloom^1^, Graham McLennan, MS^1^, Daniel Grosu, MD, MBA^1^, Mathias Ehrich^1^, Razelle Kurzrock, MD^2^

###### 1Sequenom, a LabCorp company, San Diego, CA, USA; 2University of California San Diego, San Diego, CA, USA

####### **Correspondence:** Taylor Jensen (taylor.jensen@integratedgenetics.com)

**Background**

Immune checkpoint inhibitors continue to revolutionize the cancer treatment paradigm. It has been observed that even some patients with advanced, refractory malignancies achieve durable responses; however, only a minority of patients benefit, demonstrating the importance of developing new biomarkers to predict and/or monitor patient outcome. While markers including PD-1/PD-L1 expression, microsatellite instability, and tumor mutational burden have been shown to have varying degrees of predictive power, they are not the ideal markers to monitor and differentiate response during treatment. Interrogating cell-free DNA (cfDNA) isolated from plasma (liquid biopsy) provides a promising noninvasive method for monitoring response.

**Methods**

Whole blood was collected in Streck BCT tubes and plasma subsequently separated using centrifugation. cfDNA was isolated from 4 mL of plasma using an automated process and used to prepare sequencing libraries. The total amount of cfDNA isolated from each plasma sample was quantified using droplet digital PCR. All sequencing libraries were then subjected to low-coverage, genome-wide sequencing using illumina HiSeq2500 instruments, generating a median of 35.4 million reads (~0.3X genomic coverage). A newly developed metric – the Genome Instability Number (GIN) – was utilized to measure and quantify the cumulative abundance of copy number alterations (CNAs) present in the cfDNA and additional algorithms were applied to identify the genomic loci containing CNAs.

**Results**

A series of 477 plasma aliquots prospectively collected at various time points throughout the treatment of 98 cancer patients receiving immunotherapy was measured in this study. These data built on previous results to further describe how the GIN can be used to discriminate clinical response from progression, differentiate progression from pseudoprogression, and identify hyperprogressive disease, as early as 4-6 weeks after treatment initiation. In addition, the cfDNA profiles of a small cohort of melanoma patients were evaluated at frequent intervals shortly after the initiation of checkpoint inhibitor therapy to better identify response kinetics. Finally, CNAs across the genome were analyzed to determine whether specific genes or genomic regions were associated with patient response to checkpoint inhibitors.

**Conclusions**

These data suggest that low coverage, genome-wide sequencing of cfDNA may have utility for monitoring response to immunotherapy in cancer patients.

**Ethics Approval**

This study was performed and consents were obtained in accordance with UCSD Institutional Review Board guidelines for specimen collection and data analysis (NCT02478931) and for any investigational treatments.

#### P59 Assessment of consistency of multiplex fluorescent immunohistochemistry data across multiple users utilizing different quantitative analysis strategies

##### Shawn Jensen, PhD, Carmen Ballesteros Merino, PhD, Sebastian Marwitz, Nikhil Lonberg, HSDG, Bernard A. Fox, PhD

###### Robert W Franz Cancer Center, Earle A Chiles Research Institute, Portland Providence Medical Center, Portland, OR, USA

####### **Correspondence:** Bernard A. Fox (foxb@foxlab.org)

**Background**

Tools that facilitate examination of the tumor microenvironment in cancer patients who either respond or do not respond to treatment are informative to the future design of immunotherapeutic strategies. Multiplex fluorescent immunohistochemistry (mIHC) is a technique enabling examination of the number and location of cells within the tumor microenvironment. Recently, we described the Cumulative Suppression Index (CSI), which examines the number of CD8+ T cells in the invasive margin of tumor combined with the number of FoxP3+ or PD-L1+ cells within 30 um of CD8+ T cells[1]. This CSI correlated with overall survival in a cohort of 119 patients with HPV- oral squamous cell cancer. Future application of the CSI will require reliable analysis methods with minimal variation across studies and users to enable comparative analysis. In this current study, we systematically compared the reliability of three different methods of enumerating cellular phenotypes of mIHC images across multiple users.

**Methods**

Primary tumors obtained from oral squamous cell cancer patients were sectioned and stained with antibodies to CD3, CD8, FoxP3, CD163, and PD-L1. Nine representative images were collected from one patient and analyzed using either commercial phenotyping software based on machine-learning to phenotype cells (C), commercial phenotyping software coupled with a Thresholding method (C+T), or the Thresholding method alone (T). Multiple independent users analyzed the same nine images determining the number of CD3+ PD-L1-, CD3+PD-L1+, CD8+PD- L1-, CD8+PD-L1+, FoxP3+, CD163+PD-L1-, CD163+PD-L1+, or PD-L1+ cells using each of the three methods.

**Results**

Analysis of the variation within each user across the three different analysis methods demonstrated tight correlation for the principle phenotypes of the CSI, namely CD8+PD-L1-, CD8+PD-L1+, FoxP3+, and PD-L1+ cells (Spearman Rank Correlation p<0.05). CD163+PD-L1+/- cells and CD3PD-L1+/- cells showed a correlation in only a fraction of the users which was partially influenced by low cell counts in a portion of those cellular phenotypes. More importantly, examining the reproducibility of data between users using the intraclass correlation coefficient demonstrated consistency across all phenotypes for all users using either the C+T or T methods (p<0.05).

**Conclusions**

Comparative analysis of mIHC data between multiple users requires confidence in a reproducible and consistent method for data analysis. These data demonstrate that C+T or T methods of analyzing data minimize inter-user variation when using the CSI mIHC panel tested in this study.

**References**

1. Feng Z, et al. Multiparametric immune profiling in HPV- oral squamous cell cancer. *JCI Insight.* 2017;2(14):e93652.

#### P60 Circulating tumor DNA assessment in plasma samples collected in Atezolizumab versus docetaxel in subjects with previously treated non-small-cell lung cancer (OAK) study

##### Yuqiu Jiang, PhD^1^, Namrata Patil, PhD^2^, Johnny Wu^1^, Wei Zou^2^, Stephanie Yaung^1^, Aarthi Balasubramanyam^1^, Susan Flynn^2^, Maureen Peterson^2^, Eric Peters^2^, Priti Hegde, PhD^2^, Simonetta Mocci^2^, Marcin Kowanetz, PhD^2^, John Palma^1^

###### ^1^Roche Sequencing Solutions, Pleasanton, CA, USA; ^2^Genentech, South San Francisco, CA, USA

####### **Correspondence:** John Palma (john.palma@roche.com)

**Background**

Circulating tumor DNA (ctDNA) sequencing and analysis has the potential to transform clinical management of subjects with advanced NSCLC. It has been shown that mutation(s) or molecular tumor burden assessed in plasma using NGS could potentially serve as disease monitoring tool or therapy response predictions.

**Methods**

In this exploratory pilot study, longitudinal plasma samples have been analyzed for presence of ctDNA in NSCLC subjects from the OAK study. 108 subjects were selected based on their clinical response profiles of early and late responders, and early and late progressors. For this preliminary report, 102 baseline samples and subsequent plasma collected at C2D1, C3D1, and C4D1 were analyzed by NGS. The AVENIO ctDNA Surveillance kit** (Roche, Branchburg, NJ) were used for sequencing analysis. The Surveillance kit (200 kb size) contains 17 cancer driver genes and additional 180 frequently mutated genes in cancer. This kit is capable of detecting four mutation classes: SNVs, fusions, CNVs and InDels. Association of survivals with ctDNA level or change was interrogated with Cox regression model. Response to treatment was assessed using RECIST v1.1.

**Results**

102 of the 108 (94%) baseline plasma samples were successfully sequenced. All 102 (100%) samples had somatic variants detected. The most commonly mutated genes in tumors were TP53 (59/102 subjects), KRAS (21/102), APC (21/102), and NPAP1 (15/102). The median number of variants detected per subject was 7. Mutant molecules per milliliter (MMPM) was also assessed for each baseline samples. The median MMPM was 139, ranging from 1 to 1972 for these 102 samples. Survival and therapy response in relation to ctDNA level or change at different time points will be reported at the time of presentation.

**Conclusions**

ctDNA testing with molecular barcoded sequencing and digital background error suppression of a 197 gene panel offers high sensitivity for tumor variant detection. The study demonstrated that tumor variants can be detected in blood in pre-treatment samples using the AVENIO kit. Subsequent analysis will be performed with plasma samples collected at C2D1, C3D1, and C4D1, and reported at the time of submission.**For Research Use only; Not for diagnostic purposes.

**Acknowledgements**

We would like to acknowledge Dr. Dan Klass, Ph.D., Katrina Mayol, and Nasiema Wingate-Pearse for their insightful discussions.

#### P61 Development of an exosome / EV analysis pipeline for tumor and immune monitoring

##### Joshua Welsh, PhD^1^, Kevin Conlon, MD^1^, Milos Miljkovic, MD, MSc^1^, Julia Kepley, BS^1^, Jennifer Marte, BS MD^1^, Jason Savage, PhD^1^, Veronica Galli, PhD^1^, Katherine McKinnon, MS^1^, Katherine Calvo, MD, PhD^2^, Hong Zhang^1^, Cynthia Masison^1^, Fatima Karzai, MD^1^, Fatah Kashanchi^3^, Deborah Citrin^1^, Steve Jacobson^4^, James Gulley, MD, PhD, FACP^1^, Genoveffa Franchini, PhD^1^, Thomas Waldmann, MD^1^, Kevin Camphausen, MD, PhD^1^, Jennifer Jones, MD, PhD^1^

###### ^1^National Cancer Institute, Bethesda, MD, USA; ^2^NIH Clinical Center, Bethesda, MD; ^3^George Mason University, Fairfax, VA, USA; ^4^NINDS, NIH, Bethesda, MD, USA

####### **Correspondence:** Jennifer Jones (jennifer.jones2@nih.gov)

**Background**

Exosomes and other Extracellular Vesicles (EVs) carry surface receptors that are characteristic of their cells of origin. Therefore, Extracellular Vesicles (EVs) have tremendous potential as non-invasive biomarkers for immunotherapy. We have developed a first-in-class pipeline to characterize EV heterogeneity and provide high- sensitivity quantification of informative EVs in biofluids before, during, and after treatment. This pipeline combines multiplex assays with high-resolution single EV flow cytometric methods together into a Mutiplex-to-Single EV Analysis (Mt-SEA) pipeline. With this pipeline, we are able to characterize a broad range of relevant EV subsets, while also accurately measuring the concentration of specific EV populations. With clinical cases, we demonstrate the performance of Mt-SEA method by confirming strong correlations of liquid biopsy EV repertoires with tumor burden and responses to treatment, including an abscopal immune response following radiation. Furthermore, EV analysis with Mt-SEA may identify previously unrecognized prognostic epitopes or EVs subsets.

**Methods**

To evaluate the use of this pipeline in an exploratory clinical cohort, we evaluated EVs from plasma samples of Adult T Cell Leukemia/Lymphoma patients receiving palliative radiation. Plasma was obtained before and after treatment. Multiplex EV capture beads were used with additional detection antibodies to identify more than 40 major EV subsets. General exosome and EV detection epitopes included CD63, CD9, and CD81. Tumor-specific epitopes for each patient included CD4, CD5, and CD25, based on available histo-/cyto-pathology results. Next, high-resolution single EV analyses were performed with nanoFACS sorting and a prototype nanoFCM analyzer.

**Results**

ATLL-derived EVs were detected in each pre-treatment sample, with reduced specific ATLL-derived EV subsets concentrations at the end of treatment. Furthermore, ATLL-specific EVs from patients with progressive systemic disease prior to treatment were found to carry CD44 and other stemness-associated epitopes, consistent with increasing tumor aggressiveness. Responses to treatment that were clinically evident mirrored changes in the Mt- SEA EV profiles, and Mt-SEA identified new candidate prognostic EV profiles associated with clinical outcomes that would not have been predicted.

**Conclusions**

The use of EVs as clinical biomarkers requires a combination of methods to broadly characterize EVs and rigorously enumerate specific selected EV subsets. Therefore, we developed the Mt-SEA pipeline. Mt-SEA provides unexpected insights into tumor biology, and detection of tumor-associated and immune-associated EVs and detection of EV repertoire changes during treatment paves the way to future evaluation of the Mt-SEA pipeline for personalized, bio-adaptive therapies in a wider range of tumor types.

**Ethics Approval**

The study was approved by the NCI IRB, with NIH intramural protocol number 02-C-0064.

**Consent**

Written informed consent was obtained from the patient for publication of this abstract and any accompanying images. A copy of the written consent is available for review by the Editor of this journal.

#### P62 A Pan-cancer view of the immune landscape in the tumor microenvironment via RNA and their potential for biomarkers in clinical trials

##### Wendell Jones (wendell.jones@q2labsolutions.com)

###### EA Genomics / Q2 Solutions

**Background**

Immune-based biomarkers are now commonly available when measuring the tumor microenvironment (TME), although many are based on immuno-histochemistry methods which sometimes have drawbacks: specificity, quantitation, variety. In addition, next-gen RNA sequencing and focused RNA assays are now much more accessible (for example, processing formalin-fixated material has a much higher degree of success) and are commonly employed in clinical trials to measure tumor activity and immune cell content in the TME.

**Methods**

Based on previous research [1-3] and pan-cancer information from TCGA, we have derived gene signatures for immune activity encompassing a dozen distinct subcomponents of the immune system and its response to tumor evolution. We have applied these immune signatures in multiple independent oncology datasets from the same indication and in multiple solid tumor indications (> 20) from TCGA and other sources to explain variation in disease-free and overall survival (DFS and OS). Using Cox multivariable analysis, we further have clarified the potential impact of these various immune components and tumor mutational burden (TMB) relative to therapy response and overall survival in the presence of multiple clinical covariates many of which are specific to each indication but which typically include patient age, tumor stage, and residual disease status. These signatures are available from a variety of RNA measurement platforms, including those that specialize in measuring difficult input material.

**Results**

The results include recognizing that the immune status, reflected in the expression-based signatures, play a large role (based on Hazard Ratio estimates) in explaining variation in survival in many indications, often having a larger potential impact than other biological factors or interventions. In addition, we show that there is often an interplay between cytotoxic and immune modulating cell activity in the TME (for example, cytotoxic lymphocytes vs. M2TAM cells) that explains much more variation than either factor by itself. We also show that these results are reproducible across multiple independent datasets for the same indication, implying they are robust. Further we show that while some indications have much commonality regarding the potential impact of specific immune components, other indications (such as pancreatic and kidney cancer) show more complex and sometimes counter- intuitive results regarding immune status which need to be explored further.

**Conclusions**

The results suggest relevant biomarkers for immune status that can be gleaned and developed from RNA measurements of the TME which can then be applied to clinical trials or converted into companion diagnostics as needed.

**Acknowledgements**

Some of the results presented are in part based upon data generated by the TCGA Research Network: http://cancergenome.nih.gov/

**References**

1. Newman AM, Liu CL, Green MR, Gentles AJ, Feng W, Xu Y, Hoang CD, Diehn M, Alizadeh AA. Robust enumeration of cell subsets from tissue expression profiles. Nature methods. 2015 May 1;12(5):453-7.

2. Rőszer T. Understanding the mysterious M2 macrophage through activation markers and effector mechanisms. Mediators of inflammation. 2015;2015.

3. Bindea G, Mlecnik B, Tosolini M, Kirilovsky A, Waldner M, Obenauf AC, Angell H, Fredriksen T, Lafontaine L, Berger A, Bruneval P. Spatiotemporal dynamics of intratumoral immune cells reveal the immune landscape in human cancer. Immunity. 2013 Oct 17;39(4):782-95.

**Ethics Approval**

This study is a meta-analysis of data from other studies and thus did not require review by an institutional ethics board.

#### P63 Estimation of Microsatellite Instability (MSI) by next-generation sequencing using a novel MSI classification method

##### Asha Kamat, PhD^1^, Sameh El-Difrawy^1^, Annie Kraitcheva^2^, Alice Zheng^1^, Simon Crawley^1^

###### ^1^Thermofisher Scientific, South San Francisco, CA, USA; ^2^ThermoFisher.com, Carlsbad, CA, USA

####### **Correspondence:** Asha Kamat (asha.kamat@thermofisher.com)

**Background**

Cancer-associated instabilities at microsatellite locations throughout the genome have been shown to be predictive of response to immunotherapy treatment.Here, we describe an NGS-based method to assess Microsatellite Instability (MSI) status in tumor-only and tumor-normal samples utilizing Ion AmpliSeq™ HD technology and an Ion GeneStudio™ S5 next-generation sequencer.

**Methods**

We have identified optimal chemistry and developed a novel algorithm to assess MSI status of samples using a large number of markers on an Ion GeneStudio S5™ Series sequencer. The diverse marker set includes monomers that vary in length between 10 BP and 40 BP in addition to di- and tri-nucleotide STR markers. The algorithm works with tumor-only or tumor-normal samples. Each sample is assigned an MSI score based on features that measure MSI response of markers in the assay.

**Results**

Combining a groundbreaking AmpliSeq™ HD workflow and a novel analysis method, we developed an assay that utilizes a diverse set of MSI markers. We evaluated performance of the assay and algorithm with a set of 50 samples including CRC, Endometrial and Gastric carcinomas tumor in both MSI-High and MSS status. The resulting scores were in concordance with results from capillary electrophoresis studies.

**Conclusions**

A next-generation sequencing based assay using multiple markers was developed to assign MSI status to tumor samples with great precision. The accuracy of the assay was validated using an orthogonal test. MSI status can be assigned using tumor-only or tumor-normal samples.

#### P64 Evaluation of PD-L1 and IRF-1 expression on circulating tumor cells as a predictive biomarker of checkpoint inhibitor response

##### Laura Kennedy, MD PhD^1^, Lance U'Ren, DVM, PhD^2^, Yao Sun^2^, Petros Grivas, MD, PhD^1^, Laura Chow, MD^1^, Vijayakrishna Gadi, MD, PhD^1^

###### ^1^University of Washington, Seattle, WA, USA; ^2^Rarecyte, Inc., Seattle, WA, USA

####### **Correspondence:** Vijayakrishna Gadi (vkgadi@uw.edu)

**Background**

PD-1 CPI therapy can generate durable responses with fewer side effects compared to conventional cytotoxic chemotherapy. Unfortunately, CPI can induce an objective response in less than 15 - 20% of non-melanoma solid tumor patients [1-3]. Multiple biomarkers have been evaluated as potential factors predicting response, but none has shown reproducible clinical utility across tumor types. Higher PD-L1 expression in tumor tissue is associated with higher response rates, but a single tumor tissue sample may not reflect spatial and temporal variability in PD-L1 expression. Circulating tumor cells can be collected at multiple timepoints with minimal risk and may provide a more comprehensive and dynamic view of tumor heterogeneity. Higher IRF-1 expression in tumor tissue has been correlated with longer progression-free survival (PFS) in metastatic melanoma patients treated with CPIs [4]. We hypothesize that evaluating both PD-L1 and IRF-1 expression on CTCs may better predict patient response to PD-1 CPIs.

**Methods**

Patients with metastatic solid tumors receiving de novo CPI alone or in combination with other treatments are eligible. Patients undergo peripheral blood collection for CTC evaluation at 3 timepoints: prior to receiving the first dose of CPI, prior to the second dose of CPI, and 3-6 months after starting therapy or at the time of CPI discontinuation. PFS is defined as time between initiation of therapy and progression or death of any cause, with patients censored at the time of last follow up. To isolate CTCs from peripheral blood, we use the AccuCyte® kit to create slides and a Ventana Discovery Ultra autostainer to stain the slides with a pancytokeratin (CK)/EpCAM antibody cocktail, anti-CD45, and a nuclear dye. Using the CyteFinder® software, CTCs are identified by positive staining for CK/EpCAM and negative staining for CD45. PD-L1 and IRF-1 expression are determined using mean fluorescence intensity (MFI) in computationally estimated cell compartments.

**Results**

To optimize the assay, low and high PD-L1/IRF-1 were created by spiking A549 cells cultured with or without 10 ng/mL interferon-gamma into whole blood (Figure 1). We will present progression-free survival data and corresponding CTC data for the first set of patients enrolled. A number of these patients have tumor tissue samples with varying degrees of PD-L1 expression by IHC, which will allow correlation between tumor tissue and CTC PD- L1 expression.

**Conclusions**

CTC PD-L1 and IRF-1 expression may provide a more comprehensive predictive biomarker for patients starting PD-1/PD-L1 CPIs.

**Acknowledgements**

We would like to thank the patients and providers of the Head/Neck/Lung Oncology group, the Genitourinary Oncology group, the Gastrointestinal Oncology, and the Breast Oncology group at the Seattle Cancer Care Alliance for participating in this trial. We would like to thank Alisa Clein for assistance with clinical research coordination.

**References**

1. Borghaei H, et al. Nivolumab versus docetaxel in advanced nonsquamous non-small-cell lung cancer. New England Journal of Medicine. 2015;373:1627-1639.

2. Powles T, et al. Atezolizumab versus chemotherapy in patients with platinum-treated locally advanced or metastatic urothelial carcinoma (IMvigor211): A multicentre, open-label, phase 3 randomised controlled trial. Lancet. 2018;391:748-757.

3. El-Khouiery A, et al. Nivolumab in patients with advanced hepatocellular carcinoma (Checkmate 040): An open-label, non-comparative, phase 1/2 dose escalation and expansion trial. Lancet. 2017; 389:2492-2502.

4. Smithy J, et al. Nuclear IRF-1 expression as a mechanism to assess “capability” to express PD-L1 and response to PD-L1 therapy in metastatic melanoma. J Immunother Cancer. 2017;5:25.

**Ethics Approval**

This study was approved by the Fred Hutchinson Cancer Research Center Institutional Review Board, Committee D, Protocol #10031.

**Fig. 1 (abstract P64).**
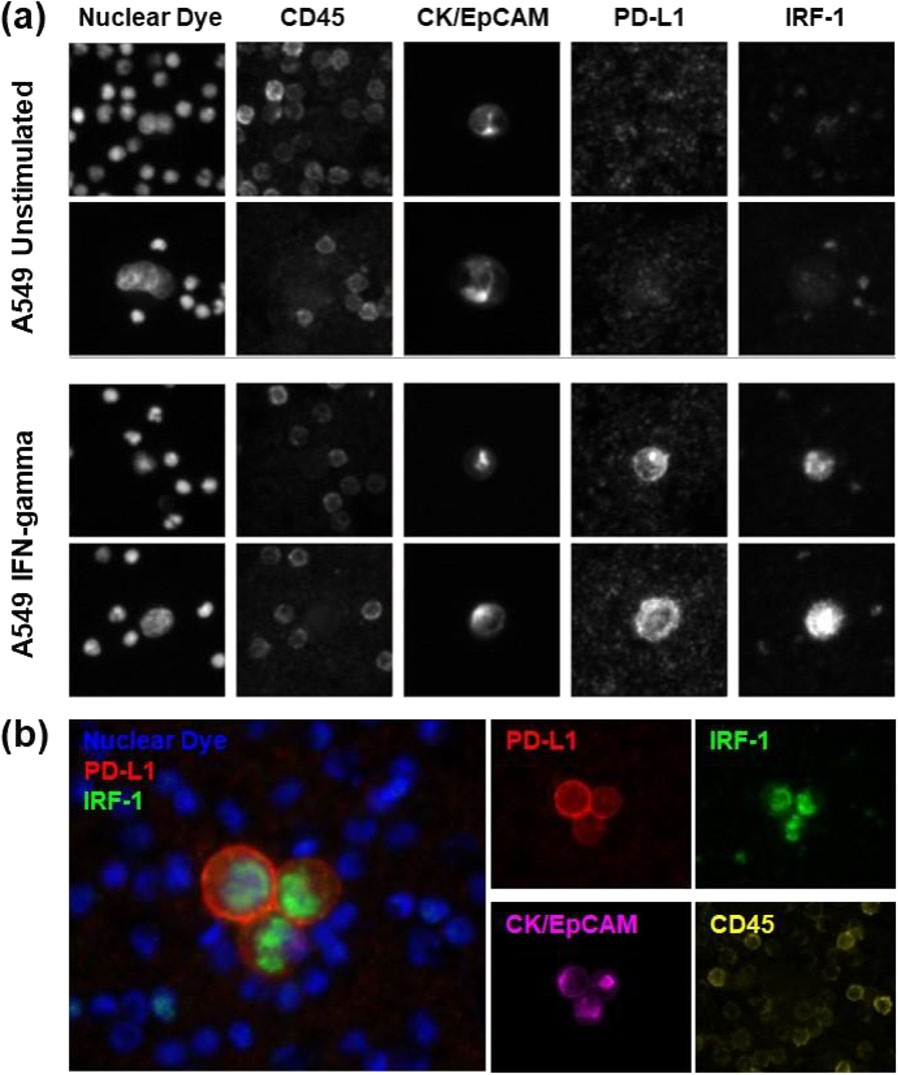
See text for description.

#### P65 IDO/HLA-DR expression and tumor mutational burden are complementary predictive biomarkers of anti- PD-1 immunotherapy in squamous cell carcinoma of the head and neck

##### Arun Khattri, PhD^1^, Ju Young Kim^2^, Riyue Bao, PhD^3^, Rajesh Acharya^3^, Yi-Hung Carol Tan, PhD^3^, Rom Leidner, MD^4^, Hisham Mehanna, PhD^5^, Nathan Roscoe^6^, Christine Vaupel, PhD^6^, Naveen Dakappagari^6^, Tanguy Seiwert, MD^3^, Sara Kochanny, BA^7^

###### ^1^Department of Medicine, The University of Chicago, Chicago, IL, USA; ^2^Navigate Biopharma Services, Inc., Carlsbad, CA, USA; ^3^The University of Chicago, Chicago, IL, USA; ^4^Providence, Portland, OR, USA; ^5^Institute of Cancer and Genomic Sciences, Birmingham, UK; ^6^Navigate Biopharma, Carlsbad, CA, USA; ^7^University of Chicago, Chicago, IL, USA

####### **Correspondence:** Tanguy Seiwert (tseiwert@medicine.bsd.uchicago.edu)

**Background**

Background: PD-1 checkpoint blockade is active in squamous cell carcinoma of the head and neck (SCCHN) (e.g. Seiwert et al, Lancet Oncol, 2016). Biomarkers such as PD-L1 immuno-histochemistry (IHC) and markers of the T- cell inflammation (e.g. Interferon-γ gene signature, INF-G) identify tumors more likely to benefit from treatment. However, both protein- and genomic markers have not been concurrently examined to identify an ideal combination of predictive markers in SCCHN.

**Methods**

Methods: Pretreatment formalin-fixed paraffin-embedded biopsies of 82 anti-PD-1 treated recurrent/metastatic SCCHN patients were studied for key immune resistance markers, namely, PD-L1 (tumor, stroma, PD-1/PD-L1 interaction), Myeloid suppressor cells (CD11b/IDO1/HLA-DR/ARG1) and regulatory T cells (CD4/CD8/CD25/FOXP3/Ki67) by combining multiplexed immunofluorescence (IF) and novel quantitative spatial imaging algorithms via AQUA® (Automated Quantitation Analysis) technology. Concurrently, INF-G gene signature was assessed by RNA-seq and tumor mutational burden (TMB) via exome sequencing (or if not available 1000-gene panel-sequencing (OncoPlus)). Correlations with overall survival (OS) and progression free survival (PFS) were performed using optimized cut points.

**Results**

Results: Stromal PD-L1 level was more predictive of survival than tumor PD-L1 level or PD-1/PD-L1 interaction score (OS, p=0.039; PFS p=0.019). However, tumor- IDO1/HLA-DR expression was most predictive of survival (OS, p=0.0005, HR = 0.4; PFS, p=0.0015, HR = 0.42), outperforming PD-L1 and INF-G signature. The markers remain significant after adjusting for HPV status in the multivariate model. TMB (using a previously established cutpoint of 175mt/exome=10mt/MB) was also associated with PFS in the overall population (OS, p=0.13; PFS, p=0.016), but TMB performed much better in HPV(-) SCCHN (OS, p=0.0083; PFS, p=0.043). Interestingly, IDO1/HLADR expression and TMB were only weakly correlated (r=0.20, P=0.078). Four distinct subgroups of tumors were identified using this combination with the combined IDO1/HLA-DRhi + TMBhi group having 60% OS plateau. HPV (+) patients had significantly higher levels of IDO1/HLADR expression in their tumors compared with HPV(-) patients (p<0.0001). Unique biologic characteristics of each of the four subgroups were also identified.

**Conclusions**

Conclusions: Our results show that IDO1/HLADR expression by tumor cells is highly predictive of outcome in SCCHN patients treated with anti-PD-1 therapy. TMB provides distinct and complementary predictive information and performs particularly well in HPV(-) tumors. The combined IDO/HLA-DR – TMB analysis identifies unique subgroups of patients, which are relevant for combination drug development as well as potential clinical care after further prospective validation.

**References**

1. Seiwert TY, Burtness B, Mehra R, Weiss J, Berger R, Eder JP, Heath K, McClanahan T, Lunceford J, Gause C, Cheng JD, Chow LQ. Safety and clinical activity of pembrolizumab for treatment of recurrent or metastatic squamous cell carcinoma of the head and neck (KEYNOTE-012): an open-label, multicentre, phase 1b trial. Lancet Oncol. 2016 Jul;17(7):956-965.

**Ethics Approval**

The study was approved by the IRB of the University of Chicago (IRB# 8980)

**Consent**

Written informed consent was obtained from the patient for publication of this abstract and any accompanying images. A copy of the written consent is available for review by the Editor of this journal

#### P66 Evaluation of Immune – related Markers in circulating proteome and their association with atezolizumab efficacy in patients with 2L+ NSCLC

##### Marcin Kowanetz, PhD^1^, Ning Leng^1^, Joanna Roder, PhD^2^, Carlos Oliveira, PhD^2^, Senait Asmellash, PhD^2^, Krista Meyer, PhD^2^, Heinrich Roder, DPhil^2^, Marcus Ballinger, PhD^1^, David Shames, PhD^1^

###### ^1^Genentech, South San Francisco, CA, USA; ^2^Biodesix, Steamboat Springs, CO, USA

####### **Correspondence:** Marcin Kowanetz (kowanetz.marcin@gene.com)

**Background**

Anti-PD-L1/PD-1 therapy has become a standard of care in NSCLC. However, understanding of the biological mechanisms of treatment efficacy and resistance is still incomplete. Here we examine the role of the circulating proteome in 2L+ NSCLC patients treated with atezolizumab (anti-PD-L1).

**Methods**

Using expression data for the circulating proteome from mass spectrometry of pre-treatment serum samples collected from 77 patients with NSCLC treated with atezolizumab (atezo) in a single arm Phase 1a study (NCT01375842) (development cohort) and machine learning methods, a test was developed to classify patients into “Good” and “Poor” prognosis groups. Protein set enrichment analysis (PSEA) was used to compare the underlying biology of classification of Good and Poor phenotypes. Blinded pre-treatment serum samples from 270 patients treated with atezo or docetaxel (doc) in the randomized Phase 2 study POPLAR (NCT01903993) were subsequently used for validation.

**Results**

The test stratified patients treated with atezo in the development cohort by overall survival (OS) (Good vs. Poor prognosis HR=0.23, p <0.001) and progression-free survival (PFS) (Good vs. Poor prognosis HR = 0.52, p = 0.012). PSEA identified trends in association of increased complement activation, acute inflammation and immune response type 2 in the Poor classification phenotype. In the blinded analysis of the validation cohort (POPLAR), 262 samples (97%) passed QC, and 134 (51%) were classified to the Good prognosis group. In the validation cohort, OS and PFS were associated with atezo efficacy compared to doc in the Good but not in the Poor prognosis group, with unadjusted classifier-treatment interaction p-value 0.005 for PFS and interaction p-value 0.001 for OS (Figure 1).

**Conclusions**

Patients characterized by complement activation, acute inflammation and immune response type 2 markers in their baseline serum appeared to derive less benefit from atezolizumab. The analysis of circulating-proteome-defined phenotypes may help to better understand the biological mechanisms beyond response and resistance to checkpoint inhibition in cancer patients.

**Trial Registration**

NCT01375842, NCT01903993

**Fig. 1 (abstract P66).**
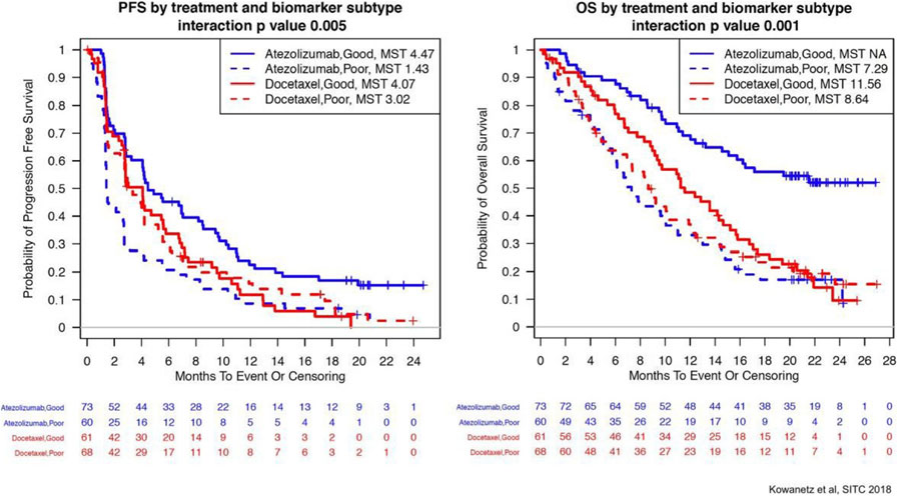
See text for description.

#### P67 Innovative combinatorial approach to characterize the immune landscape and analyze the tumor response after anti-PD-1 blockade in a 3D ex-vivo tumoroid system of non-small cell lung cancer

##### Melba Marie Page, PhD, Melanie Mediavilla-Varela, PhD, Jenny Kreahling, PhD, Soner Altiok, MD, PhD

###### Nilogen Oncosystems, Tampa, FL, USA

####### **Correspondence:** Melanie Mediavilla-Varela (melanie@nilogen.com)

**Background**

Cell lines and mouse models have provided valuable information in understanding the tumor microenvironment. The overall success in translating these results to the clinic is less than 10% and lacks the full complexity of the tumor microenvironment. Understanding these greater complexities is critical to the development of immuno-oncology therapies. Nilogen Oncosystems’ proprietary 3D-EXSM ex vivo drug screening platform analyzes fresh patient tumor tissue that remains embedded in its natural environment as a tumoroid. This approach includes a powerful combination of confocal image analysis, flow cytometry analysis, cytokine assays as well as gene expression analysis. With this model, we can visualize response, phenotype the tumor microenvironment and accurately determine the response to checkpoint inhibitors in non-small cell lung cancer (NSCLC) and correlates can be found among different platforms.

**Methods**

For the 3D-EX vivo platform, tumoroids were shaped from procured fresh tumor tissue from NSCLC cancer patients. They were then treated with Keytruda ex-vivo and treatment-mediated changes in TIL subpopulations were analyzed using confocal analysis, flow cytometric analysis, cytokine release by Bio-Rad’s 17-plex cytokine assay as well as gene expression by NanoString’s 770 gene Immune Panel.

**Results**

Ex vivo treatment of the 3D tumoroids with Keytruda, showed significant changes in T-cell activation and immune cell populations in 26% of NSCLC tumors. This was observed by the simultaneous increase in IFN-γ and TNF-α upon cytokine analysis. Furthermore, we found a differential expression of signature genes such as CD8, CXCL10, CXCL9, EOMES, Granzyme A/B, IFN-γ, related to T-cell subpopulations via Nanostring analysis, which was accompanied by an increase in Granzyme B via flow cytometry. Confocal imaging analysis allowed detection of immune cell mediated killing of tumor cells upon ex-vivo drug treatment.

**Conclusions**

The positive and negative associations between expression of immune function genes, TIL activation, and cytokine production by ex-vivo treatment shows that Nilogen Oncosystems’ 3D-EXSM platform can accurately recapitulate the tumor microenvironment. This comprehensive approach including the powerful visualization by confocal analysis provides profound technological advantages in analyzing the tumor immune microenvironment. With this combinatorial approach, the 3D-EXSM platform delivers a better understanding of the mechanism of action of immuno-oncology drugs that may aid in developing biomarkers that can be used for patient selection.

#### P68 Using high dimensional mass cytometry (CyTOF) and machine assisted analysis to detect biomarkers in the immunotherapy of cancer

##### Carsten Krieg, PhD^1^, Silvia Guglietta^1^, John Wrangle, MD^1^, Luis Cardenas, BS^1^, Mitchell Levesque^2^, Reinhard Dummer, MD^2^, Burkhard Becher^3^, Mark Rubinstein, PhD^1^, Mark Robinson^4^

###### ^1^Medical University of South Carolina (MUSC), Charleston, SC, USA; ^2^University Hospital Zurich, Zurich, Switzerland; ^3^University Zurich, Zurich, SC, Switzerland; ^4^University Zurich (IMLS), Zurich, Switzerland

####### **Correspondence:** Carsten Krieg (KriegC@musc.edu)

**Background**

Immunotherapy has created a lot of enthusiasm in oncology but, as not all patients respond, it becomes evident that the strategy “one drug fits all” is not applicable to all patients and all cancers. Therefor, selection of patients benefiting from mono-immunotherapy, combination therapy or advice on which drug to use on therapy, ideally by biomarker, is in high demand.

**Methods**

Here we approached the problem by designing a customized workflow by using high-dimensional single cell mass cytometry combined with machine-learning bioinformatics for the in-depth characterization of single immune cells in predicting and monitoring immune responses. The analysis is data driven, can be adapted to high throughput approaches and can model arbitrary trial designs such as batch effects and paired designs. We tested our workflow on two studies:

**Results**

a) Predict response to anti-PD-1 immunotherapy in melanoma. In our discovery cohort peripheral blood mononuclear cells (PBMCs) from 20 patients with stage IV melanoma before and 12 weeks on anti-PD-1 therapy was analyzed. We observed a clear T cell response on therapy. The most evident difference in responders before therapy was an enhanced frequency of CD14+ CD16+HLA-DRhi classical monocytes. We validated our results using conventional flow cytometry in an independent exploratory cohort of 31 patients before therapy. Finally, we correlated enhanced monocyte frequencies before therapy initiation with clinical response and could show association with lower hazard, extended progression-free and overall survival.b) Monitoring the immune response in non small cell lung cancer patients refractory to anti-PD-1 immunotherapy treated with a novel combination immunotherapy of anti-PD-1 and an IL-15 superagonist. 21 patients with non small cell lung cancer who got refractory to anti-PD-1 treatment received a novel combination therapy of IL-15 superagonist plus anti-PD-1. A response in the CD8+ T cell compartment was observed characterized, among other factors, by expansion of TCR variety. Unexpected we observed a strong expansion of effector NK cells starting around day 4 of therapy.

**Conclusions**

Taken together, high throughput mass cytometry together with an unbiased artificial intelligence driven analysis workflow might support patient selection prior to therapy, select the right drug combination and identify new drugable cell populations.

**Ethics Approval**

These studies have been approved by the University of Zurich and Medical University of South Carolina Ethics Boards.

#### P69 Simultaneous quantification of activated immune cells and PD-L1 expressing circulating tumor cells (CTCs) in peripheral blood of cancer patients receiving checkpoint inhibitor therapy

##### Rachel Krupa, BS, Robin Richardson, Priscilla Ontiveros, Joseph Schonhoft, Yipeng Wang, PhD, MD, Jiyun Byun, MS, David Lu, Aaron Oh, Sean Nisperos, Mark Landers, MS, Ryan Dittamore, MS

###### Epic Sciences, San Diego, CA, USA

####### **Correspondence:** Rachel Krupa (rachel.krupa@epicsciences.com)

**Background**

Expression of PD-L1 on tumor and tumor infiltrating lymphocytes has been associated with improved response to PD-1 and PD-L1 checkpoint inhibitors, however clinical utility is limited. Multimodal characterization of both the tumor and host immune system is an unmet medical need for the improved prediction of response to immunotherapy. Metastatic lesions are likely to be under-sampled by biopsy given tumor heterogeneity, clonal evolution, and temporal changes in the host immune system under therapeutic pressure. Therefore, we sought to expand the existing Epic Sciences’ non-invasive liquid biopsy platform to simultaneously examine expression of PD-L1 on circulating tumor cells (CTCs) as well as quantify changes in activated immune cell populations from a single sample in patients undergoing checkpoint inhibitor therapy. Examining dynamic biomarker changes both prior to and on therapy could yield novel diagnostic tools for response prediction and pharmacodynamics response for immunotherapy.

**Methods**

Blood samples from bladder, kidney, and prostate cancer patients undergoing checkpoint inhibitor therapy were collected at baseline and on-therapy (when available). Contrived samples were developed using cancer cell lines and healthy donor (HD) blood. Red blood cells were lysed and nucleated cells were plated onto glass slides and stained with DAPI and immune panels including pan-CK, CD45, PD-L1, CD4, CD8, and Ki-67. Approximately 3 million cells per slide were imaged through advanced digital pathology pipelines to detect and quantify changes in immune cell populations and to assess circulating tumor burden.

**Results**

An immuno-panel was developed to profile activated (CD8+Ki-67+ and CD4+Ki-67+) leukocyte subpopulations and PD-L1+ CTCs from a single blood sample. Feasibility was demonstrated in cell lines, HD, and patient blood. CTCs were detected in 76% (32/42) of samples tested. Of the 25 baseline samples tested, 12% (3/25) had PD-L1+ CTCs detected. No PD-L1+ CTCs were detected in the 13 on-therapy samples tested. Of 14 patients with matched samples, 50% (7/14) patients had an increase in activated CD4+ leukocytes and 14% (2/14) patients had an increase in activated CD8+ leukocytes in on-therapy samples compared to baseline. A decrease in activated CD4+ leukocytes was observed in 7% (1/14) patients and a decrease in activated CD8+ leukocytes was also observed in 7% (1/14) patients with therapy.

**Conclusions**

Development of a liquid biopsy based platform that can simultaneously measure immune biomarkers in CTCs and leukocytes will allow for real time assessment and monitoring of response to immune checkpoint inhibitors and may lead to novel diagnostic tools for response prediction.

#### P70 CD4+FOXP3+ regulatory T cells in the periphery of HNSCC patients demonstrate high phenotypic diversity depending on Treg subtype

##### Cornelius Kürten, MD^1^, Shanhong Lu^2^, Tullia Bruno, PhD^3^, Robert L. Ferris, MD, PhD^3^

###### ^1^University of Essen, Essen, PA, Germany; ^2^Centre South University, Pittsburgh, PA, USA; ^3^University of Pittsburgh, Pittsburgh, PA, USA

####### **Correspondence:** Robert L. Ferris (ferrisrl@upmc.edu)

**Background**

Regulatory T cells (Treg) promote immune escape and are a putative biomarker for response to immunotherapy.

Indeed, previous data from our group demonstrated that Ki67+ Treg were elevated in head and neck cancer (HNSCC) responders to nivolumab (CheckMate-141). Importantly, phenotypic markers for different subsets of Treg have been described: naïve Treg (nTreg, CD45RA+FOXP3low), effector Treg (eTreg, CD45RA-FOXP3high) and non-suppressive Treg (nsTreg, CD45RA-FOXP3low). While these populations have been described in peripheral blood lymphocytes (PBL) and tumor-infiltrating lymphocytes (TIL) of cancer patients, the presence of suppressive eTreg in the blood is a biomarker for disease progression in HNSCC patients. Thus, a more detailed characterization of this cell population is warranted.

**Methods**

Multi-parameter flow cytometry (CD8, CD4, CD45RA, FOXP3, Neuropilin-1, CD39, PD-1, Tim-3, LAG-3, CTLA-4, TIGIT, CD69, pAKT, Ki67, Bcl2) was performed on PBL from HNSCC patients (n = 50). T cell function was assessed using intracellular staining for cytokine production (IFNy, TNFa) after a 4 hour stimulation with PMA/Ionomycin. Findings were correlated with patient demographics and clinical outcome.

**Results**

A higher percentage of eTreg express inhibitory receptors (IRs) (two-way ANOVA: PD-1: mean diff. 22%, p <0.0001; Tim-3: mean diff. 15 %, p < 0.01; CTLA-4: mean diff. 13 %, p < 0.05; TIGIT: mean diff. 26 %, p < 0.0001; CD39: mean diff. 40 %, p < 0.0001) compared to naïve/non-suppressive Treg population. IR expression correlated highly on eTreg (Spearman correlation e.g. for PD-1 with Tim-3 r2 = 0.70, PD1 with TIGIT r2 = 0.72, PD-1 with CD39 r2 = 0.82, all p < 0.0001), but not on the non-effector Treg. Interestingly, proliferative eTreg (measured by Ki67 expression) were strongly associated with better survival (Hazard Ratio: 0.29, p < 0.05).

**Conclusions**

eTreg are a distinct cell subtype in HNSCC, as shown by their differential expression of IRs and proliferation/activation markers. The higher IR expression suggests that eTreg are an important target of currently available immunotherapy drugs among the whole Treg population. This also underscores the putative value of this cell subset as a biomarker of response to monitor longitudinally during anti-PD-1 immunotherapy.

#### P71 Characterization of tumor mutational burden (TMB) and homologous recombination repair (HRR) mutations to assess correlation with immune checkpoint inhibitors (ICIs) response in renal cell carcinoma

##### Matthew Labriola, MD^1^, Jason Zhu, MD^2^, Rajan Gupta^1^, Shannon McCall, MD^1^, Jennifer Jackson, PhD^3^, James White, PhD^3^, Elizabeth Weingartner^3^, Eric Kong^3^, Peter Simone, PhD^4^, Eniko Papp, PhD^3^, Kelly Gerding, PhD^3^, Eun-Hae Kim, PhD^3^, John Simmons, PhD^3^, Daniel George, MD^2^, Tian Zhang, MD^2^

###### ^1^Duke University Hospital, Durha, NC, USA; ^2^Duke Cancer Institute, Durham, NC, USA; ^3^Personal Genome Diagnostics, Baltimore, MD, USA; ^4^Pesronal Genome Diagnostics, Baltimore, MD, USA

####### **Correspondence:** Tian Zhang (tian.zhang2@duke.edu)

**Background**

The advent of immune checkpoint inhibitors (ICI) has revolutionized the treatment landscape for patients with metastatic renal cell carcinoma (mRCC) [1,2]. However, traditional biomarkers such as PD-L1 have not served as predictive markers of treatment response. Given the risk of toxicity and variable response rates, there is a need to develop more reliable predictive biomarkers to support precision immunotherapy. High tumor mutational burden (TMB) has been previously described as a robust biomarker for predicting ICI response in metastatic melanoma [3] and non-small cell lung cancer [4], but has not yet been fully explored in mRCC. Here, we describe the prediction of clinical outcomes for mRCC patients and ICI response using a solid tissue-based next-generation sequencing (NGS) assay to identify genetic correlates and tumor mutation burden.

**Methods**

34 patients with mRCC who had received ICI therapy at Duke Cancer Institute were identified. FFPE tumor samples from archival tissue banks were evaluated using Personal Genome Diagnostics elioTM tissue complete investigational NGS assay, screening for somatic variants across >500 genes, as well as TMB and microsatellite status. Clinical information was extracted from the medical record and tumor response was evaluated based on RECIST 1.1 criteria.

**Results**

16 of 34 patients displayed disease control (overall responses of: stable disease, partial response, or complete response) following ICI therapy. This patient cohort displayed a range of TMB scores from 0.4 to 12.2 mutations/Mb (predicted whole exome equivalent), with a mean TMB score of 2.8 mutations/Mb, exome equivalent. Overall, there was no significant difference in TMB scores between responders and non-responders, and no significant correlation between increased TMB score and response to ICI was found (Figure 1). Interestingly, genes related to DNA repair pathways, particularly homologous recombination (including FAM175A, RAD50, RECQL4, and SLX4), were more often found to be mutated in the ICI responder group compared to ICI non-responders. Of the 16 responders, 9 were found to harbor somatic mutations in at least one gene associated with DNA repair (Figure 2).

**Conclusions**

Overall, TMB did not appear to correlate to patient outcomes or ICI response in this mRCC patient cohort. However, NGS analysis showed an increase in somatic mutations in DNA repair genes in responders compared to non-responders. Recently, hereditary RCC syndromes have been mechanistically linked to defects in homologous recombination [5]. Our findings suggest that mutations in DNA repair pathway genes may correlate with ICI response and may have potential as a predictive biomarker for treatment success.

**Acknowledgements**

Study supported by research funds from Personal Genome Diagnostics (PGDx).

**References**

1. Motzer RJ, Escudier B, McDermott DF, et al. Nivolumab versus everolimus in advanced renal-cell carcinoma. New England Journal of Medicine. 2015; 373(19): 1803-13.

2. Motzer RJ, Tannir NM, McDermott DF, et al. Nivolumab plus ipilimumab versus sunitinib in advanced renal-cell carcinoma. New England Journal of Medicine. 2018; 378(14): 1277-90.

3. Goodman AM, Kato S, Bazhenova L, et al. Tumor mutational burden as an independent predictor of response to immunotherapy in diverse cancers. Mol Cancer Ther. 2017; 16(11): 2598-608.

4. Hellmann MD, Ciuleanu TE, Pluzanski A, et al. Nivolumab plus ipilimumab in lung cancer with a high tumor mutational burden. New England Journal of Medicine. 2018; 378(22): 2093-104.

5. Sulkowski PL, Sundaram RK, Oeck S, et al. Krebs-cycle-deficient hereditary cancer syndromes are defined by defects in homologous-recombination DNA repair. Nat Genet. 2018. Epub ahead of print.

**Ethics Approval**

This study was approved by Duke University’s Institutional Review Board, protocol number Pro00088779.

**Fig. 1 (abstract P71).**
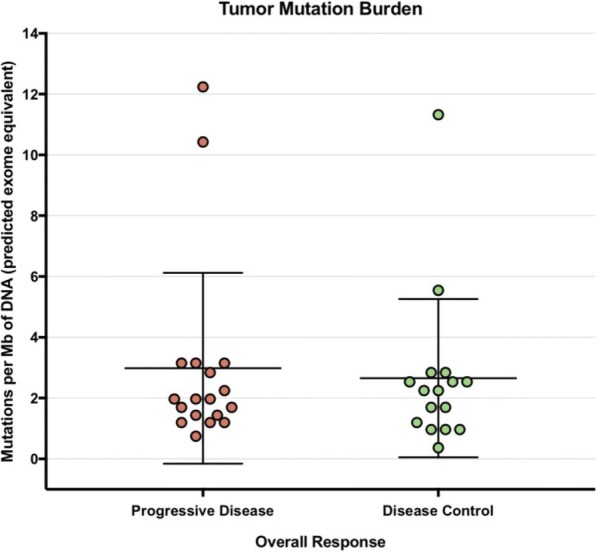
See text for description.

**Fig. 2 (abstract P71).**
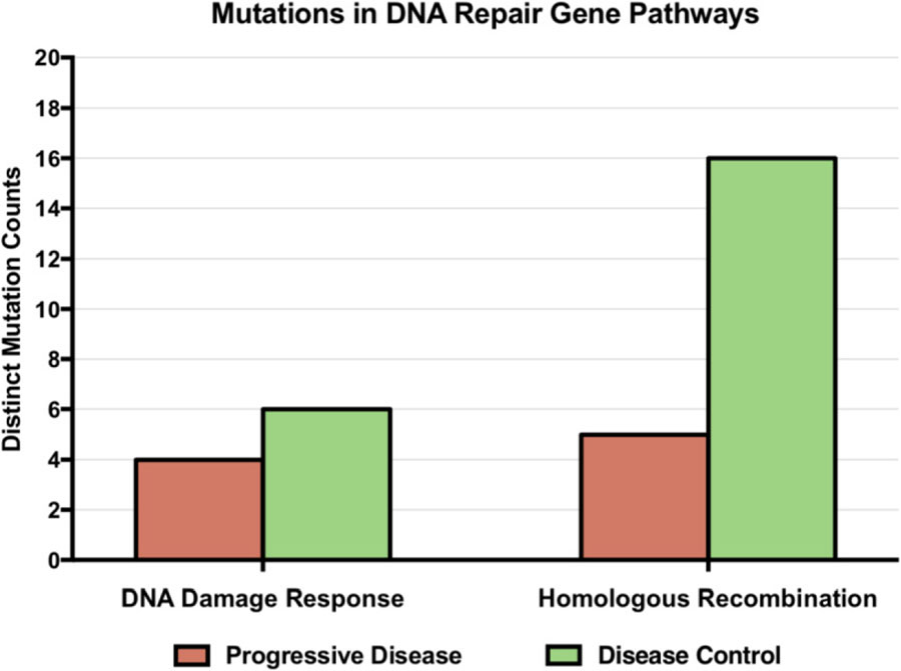
See text for description.

#### P72 Biomarker and preliminary pharmacodynamic evaluations of the PD-1 inhibitor ABBV-181 from an ongoing phase 1 clinical trial in patients with advanced solid tumors

##### Stacie Lambert, Gregory Vosganian, MD, Fiona Harding, PhD, James Sheridan, Stefan Englert, Dr, Sara Siggelkow, Apurvasena Parikh, PhD, Betty Wang, Kinjal Hew, Jaishree Bankoti, Marcia Stickler, Eileen Lee, Merriam Mcclellan, Daniel Afar, PhD, Anita Reddy, PhD, Stacie Lambert

###### AbbVie, Inc, Redwood City, CA, USA

####### **Correspondence:** Stacie Lambert (stacie.lambert@abbvie.com)

**Background**

ABBV-181 is a humanized, recombinant, modified IgG1 monoclonal antibody targeting programmed cell death 1 (PD-1). Here we present preliminary analyses of ABBV-181 pharmacodynamic (PD) data from an ongoing Phase 1 study in patients with solid tumors (NCT03000257).

**Methods**

Prior to initiation of clinical testing, ABBV-181 was characterized in vitro for PD-1 blocking ability and bioactivity and a PD-1 saturation assay was developed. In the ongoing Phase 1 study, patients with previously treated advanced solid tumors received ABBV-181 at 1, 3, or 10 mg/kg intravenous once every 2 weeks (Q2W) in dose escalation. Following dose finding, multi-histology, non-small cell lung cancer (NSCLC) and head and neck squamous cell cancer (HNSCC) cohorts were opened at 250 mg Q2W. PD-L1 expression was assessed on pre-treatment tumor samples (PD-L1 immunohistochemistry 28-8 pharmDx, 1% threshold). Whole blood samples were collected pre and post dosing to measure PD-1 receptor saturation and expression of Ki-67 and other biomarkers in circulating T cell populations by flow cytometry, while serum was collected pre and post dosing to measure cytokine biomarkers. Pharmacokinetic (PK) sample collections and PK-PD analyses were also conducted.

**Results**

In vitro characterization demonstrated that ABBV-181 blocked PD-1 binding to its ligands and increased IFNγ production in functional human and non-human primate peripheral blood mononuclear cell assays. T cells from non- human primates administered ABBV-181 showed PD-1 saturation. Preliminary clinical PD data were available for 72 patients treated with ABBV-181 (n=25 in dose escalation, n=47 in ongoing 250 mg Q2W dose expansion). PD- L1 was expressed on 32/60 (53%) available pretreatment tumor samples, with higher expression rates in the NSCLC and HNSCC cohorts compared to the multi-histology cohort. Rapid and sustained PD-1 saturation on circulating CD4 T central memory cells was observed at all ABBV-181 doses tested. A transient decrease in circulating T cell counts following dosing was also observed, consistent with prior reported clinical observations of PD-1 blocking agents. Increases in Ki-67+ CD8+ T cells within the first cycle were detected in approximately half of the tested patients. Significant increases in serum chemokines including IP-10 were also found post dosing with ABBV-181.

**Conclusions**

Preliminary results indicate that ABBV-181 demonstrates PD-1 receptor saturation and biological activity in peripheral blood at all clinical doses tested. PD data is consistent with the biological activities previously reported for other PD-1 blocking antibodies. Enrollment in these expansion cohorts continues at 250 mg Q2W and 500 mg once every 4 weeks as supported by ongoing PK-PD analyses.

**Acknowledgements**

AbbVie and the authors thank the patients participating in this clinical trial and all study investigators for their contributions.

**Trial Registration**

ClinicalTrials.gov, NCT03000257

**Ethics Approval**

This study (NCT03000257) was approved by each participating institution’s Ethics Board.

#### P73 Analysis of survival and mRNA expressivity in the tumor microenvironment of adenocarcinoma via K-means clustering algorithm

##### Sunyoung Lee, MD, PhD^1^, Andrew Baird, MD^3^, Jillian Dolan, BS^4^, Stuart Baird^5^, Fateeha Furqan, MD^6^, Shinyoung Park, MS^1^

###### ^1^Roswell Park Cancer Institute, Williamsville, NY, USA; ^2^National Institute for Mathematical Sciences, Seoul, Korea, Republic of; ^3^University of Pittsburgh Medical Center, Pittsburgh, PA, USA; ^4^University at Buffalo School of Medicine, Buffalo, NY, USA; ^5^St. Lawrence University, Canton, NY, USA; ^6^Rochester General Hospital, Rochester, NY, USA

####### **Correspondence:** Sunyoung Lee (syandsy@gmail.com)

**Background**

Stromal elements in tumor microenvironment (TME) impact the response to cytotoxic chemotherapy and immunotherapy. Advances in mRNA-sequencing have improved our understanding of TME expressivity. However, few models exist to analyze immune crosstalk between TME elements and mRNA expressivity in terms of patient survival.

**Methods**

mRNA-seq of 3,758 adenocarcinoma tumors and 314 non-tumor tissues (lung, breast, esophageal, gastric, colorectal, pancreatic, ovarian, endocervical, and prostate adenocarcinoma) were obtained from the Cancer Genome Atlas (TCGA) and analyzed based on mRNA expression. The mRNA expressivity of 195 genes enriched in stromal components were arranged into 26 gene groups (tables 1 and 2). Using mRNA expressivity via K-means algorithm (50 cycles of machine learning), patients were clustered into two groups (high and low mRNA expression) for each gene group. Kaplan-Meier and correlation analyses were performed to assess the significance of each gene group in survival.

**Results**

Genes associated with immune activation correlate with better survival until 60 (lung), 110 (breast), 50 (esophageal), and 20 months (pancreatic). There is negative correlation with survival in gastric and colorectal, no correlation in ovarian, and positive correlation in endocervical. Angiogenesis is negatively associated with survival in colorectal (p<0.1), lung and gastric (p<0.05). Genes related to desmoplasia and immunosuppressive chemokines have a significant association with poor survival in gastric, colorectal, and endocervical (p<0.05), as well as ovarian (p<0.1). Pancreatic has extensive expression of genes associated with desmoplasia and immunosuppressive chemokines, but degree of expressivity does not correlate with survival. Neutrophils are negatively associated with survival in gastric and endocervical (p<0.05), as well as esophageal and ovarian (p<0.1), but positively correlate in breast (p<0.1). Genes associated with cancer stem cells negatively correlate with survival in pancreatic. In prostate, expressivity of immune-related genes was low, and no correlation was found except for genes related to type II IFN, which positively correlate with survival. Patients with enhanced expression of genes associated with type I IFN and antigen presentation in tumor tissue show increased expression of these genes in their respective non-tumor tissue samples.

**Conclusions**

Analysis of large data was assisted by K-means (machine learning) algorithm, showing that stromal genes have varied impact on survival in adenocarcinoma. Genes associated with immune activation have temporal correlation with survival, which seems to be a result of tumor immune escape. Expressivity of type I IFN and antigen presentation in non-tumor tissues is conserved in tumor tissues. Future prospective studies in response to chemotherapy and immunotherapy are warranted.

**Table 1 (abstract P73).**
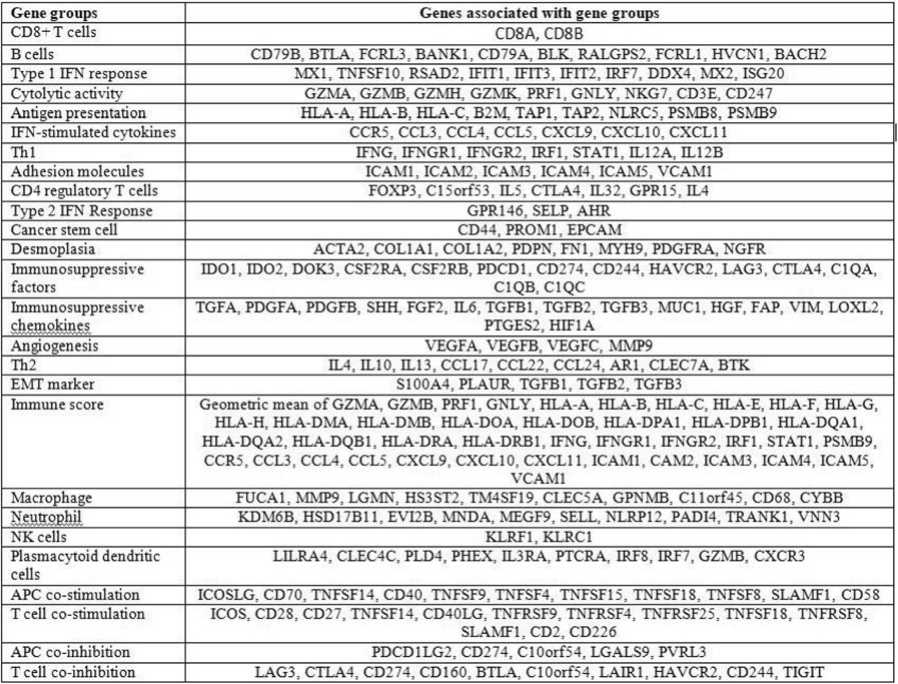
Gene associated with each gene group

**Table 2 (abstract P73).**
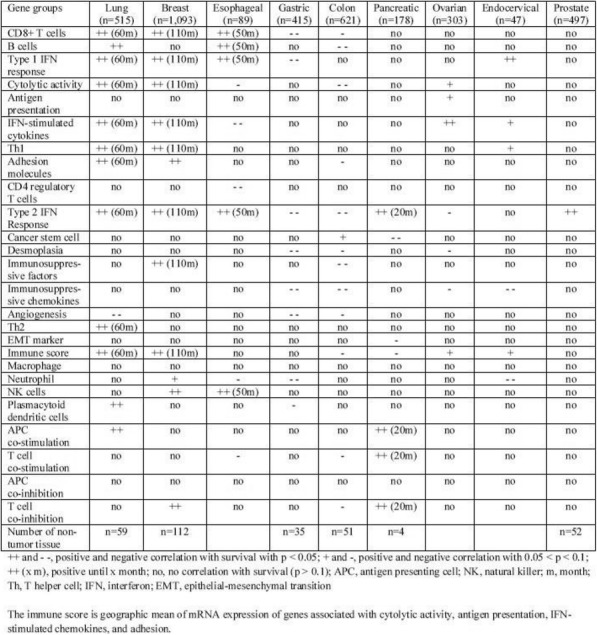
Gene groups and association with survival in adenocarcinoma, analyzed by Kaplan-Meir analysis

#### P74 Validation the immune contexture as prognostic biomarkers in high-grade serous ovarian cancer

##### Shin Wha Lee, MD^1^, Ju-hyun Kim^1^, Soo-Jung Kim^2^, Yong-Man Kim^1^

###### ^1^Ulsan University, ASAN Medical Center, Seoul, Korea; ^2^ASAN Institute for Life Science, Seoul, Korea, Republic of

####### **Correspondence:** Yong-Man Kim (ymkim@amc.seoul.kr)

**Background**

The analysis of single parameters alone may not provide sufficient insights about complex immune system–tumor interactions. This study is to validate the immune contexture as prognostic biomarkers in high-grade serous ovarian cancer (HGS-OC) and to find new era of immunoscore in HGS-OC.

**Methods**

We collected FFPE samples from 187 patients with HSOC and produced TMA samples. We accomplished the OPAL multiplex IHC assay for the quantitative analysis of immune markers, including CD4, CD8, CD20, FoxP3, PD-L1, and CK. Multiplex Biomarker Imaging and inForm® Image Analysis Software was used.

**Results**

FIGO stage III and IV patients were 84.5% (158/187). The optimal debulking surgery was done in 66.8% (125/187).

The 3-year disease-free survival and 5-year overall survival were 35.1% and 50.0%, respectively. Any single marker was not related to the survival including CD8, FoxP3, and PD-L1. However, high CD8:FoxP3 and CD8:PD-L1 ratios were correlated with the good survival. In cox regression model, the risk factors for HGS-OC survival were FIGO stage (HR 1.784, 95% CI: 1.295-2.457, p<0.001) and platinum resistance (HR 4.257, 95% CI: 2.753-6.582, p<0.001). Additionally, CD8:PD-L1 ratio was a favorable prognostic factor (HR 0.621, 95% CI: 0.042-0.917, p=0.017).

**Conclusions**

These findings indicate that, although any single immune marker is not related to the survival, CD8:FoxP3 and CD8:PD-L1 ratios provide the positive correlation with the prognosis in HGS-OC. Especially, CD8:PD-L1 ratio is prognostic biomarker which is comparable to clinical biomarkers. The next study for immunoscore is necessary to define immunoscore in ovarian cancer.

**Acknowledgements**

This study was supported by the R&D Project, Asan Institute for Life Sciences, Seoul, Korea (2016-588).

#### P75 Development of a next-generation sequencing-based microsatellite instability assay (MSI-NGS) for solid tumor testing

##### Sean Glenn, PhD, Sarabjot Pabla, MSc, PhD, BS, Jonathan Andreas, MS, Blake Burgher, BS, RN, Jacob Hagen, Jeffrey Conroy, BS, Mary Nesline, MS, Antonios Papanicolau-Sengos, MD, Vincent Giamo, BS, MS, Felicia Lenzo, Maochun Qin, MD, MS, Yirong Wang, MS, Mark Gardner, Carl D. Morrison, MD, DVM

###### OmniSeq, Inc., Buffalo, NY, USA

####### Carl D. Morrison (carl.morrison@omniseq.com)

**Background**

Microsatellite instability (MSI) is as a screening test for Lynch syndrome (HNPCC), is FDA-approved as a companion diagnostic for checkpoint inhibition, and has demonstrated high positive predictive value for response to anti-PD1 therapy for MSI-high/dMMR patients. Typically, MSI analysis involves comparison of allelic profiles of microsatellite markers generated by amplification of DNA from matching normal and tumor samples using a fluorescent PCR-based assay. The requirement of needing matched normal DNA has limited the number of patients that can have testing performed due to the difficulty in obtaining adjacent benign tissue in the tumor specimen received. Utilizing advances in NGS technologies and bioinformatics, we have developed a targeted, multi-plexed NGS assay (MSI NGS) which robustly and accurately determines MSI status without the requirement of a matched normal DNA.

**Methods**

Utilizing previously published data sets, 29 highly significant loci within the genome were determined for interrogation and integration into the MSI NGS targeted assay. For each loci, the number of peaks and average indel lengths were utilized in a custom algorithm on a gold standard training set, where MSI status was previously determined by MSI PCR methods, to define cluster centroid metrics which determine MSI and MSS status. By this process cluster 1 and cluster 2 were assigned as “MSI” and cluster 3 was assigned as “MSS” with 100% PPV and 96% NPV. The Euclidean distance of each experimental sample is then calculated across all 29 loci, and the cluster centroid closest to the sample determines the MSI status.

**Results**

Utilizing a 100 gold-standard sample set the overall concordance of the MSI NGS assay to MSI PCR is extremely high with only two false negative calls (98% concordance). The two false negative cases can be attributed to the fact that these cases are in cluster 1 which is closer (Euclidean distance) to the centroid of cluster 3 (MSS) than the centroid of cluster 2 (MSI). To this end, the reported sensitivity and specificity of the accuracy study are 96% and 100% respectively with a PPV of 100% and an NPV of 96%.

**Conclusions**

The development of a MSI NGS assay, which has recently been NYS-CLEP approved for all solid tumors, allows for robust and accurate testing of MSI status without the need of matched normal tissue, a major hurdle with conventional testing.

**Ethics Approval**

OmniSeq’s analysis utilized deidentified data that qualified as non-human subject research under IRB protocol (BDR #073166) approved by Roswell Park Comprehensive Cancer Center (Buffalo, NY).

#### P76 Analysis of the complex immune-cell milieu of tumors from patients treated with immunotherapy to better understand clinical response

##### Shumei Kato, MD^1^, Ryosuke Okamura^1^, Mina Nikanjam^1^, Ramez Eskander, MD^1^, Paul Fanta, MD^1^, Suzanna Lee^1^, Sean Glenn, PhD^2^, Devin Dressman, PhD^2^, Sarabjot Pabla, MSc, PhD, BS^2^, Jeffrey Conroy, BS^2^, Mary Nesline, MS^2^, Antonios Papanicolau-Sengos, MD^2^, Felicia Lenzo^2^, Mark Gardner^2^, Carl Morrison, MD, DVM^2^, Razelle Kurzrock, MD^1^

###### ^1^UC San Diego Moores Cancer Center, La Jolla, CA, USA; ^2^OmniSeq, Inc., Buffalo, NY, USA

####### **Correspondence:** Razelle Kurzrock (rkurzrock@ucsd.edu)

**Background**

Although immunotherapies, especially checkpoint inhibitors, have achieved salutary anti-cancer effect among patients with advanced cancers, most patients do not respond to immunotherapies. We comprehensively evaluated biomarkers associated with the “cancer-immunity cycle” among patients with diverse solid tumors to understand the immune landscape in metastatic cancers and the resistance mechanism for anti-PD1/PDL1 inhibitors.

**Methods**

Interrogation of key markers of the cancer-immunity cycle was carried out in patients (n=101) with diverse malignancies using a NYS Clinical Laboratory Evaluation Program (CLEP) approved targeted RNA sequencing assay (51 genes) developed in a Clinical Laboratory Improvement Amendments (CLIA) certified laboratory. Resultant gene expression data was QC filtered, normalized and ranked based on an assorted reference population of various tumor types. Gene signatures were determined using these ranked values with a rank value > 85th percentile considered high.

**Results**

The immune phenotypes of the patients’ tumor milieu demonstrated overexpression of multiple checkpoint blockade markers including PD-L1 (6.9%), PD-L2 (10.9%), CTLA4 (3%), LAG-3 (8.9%), TIM-3 (9.9%) and VISTA (15.8%). Overexpression of other cancer-immunity cycle markers were also observed including myeloid suppression markers (e.g. CCL2, CCR2 and CSF1R; 10-22%), metabolic immune escape markers (e.g. ADORA2A and IDO1; 9-16%) and T-cell primed markers (e.g. CD40, GITR, ICOS and OX40; 4-26%). Each patient had a unique cancer- immunity expression pattern that was distinctive from others in this cohort. Overexpression of TIM-3 and VISTA was associated with significantly shorter progression-free survival (PFS) from anti-PD1/PDL1 based therapies (P=0.007 and P=0.001 respectively).

**Conclusions**

Evaluation of the gene expression levels of biomarkers associated with the cancer-immunity cycle was feasible among diverse solid tumors using targeted RNA sequencing. Checkpoint blockade markers (TIM-3 and VISTA) were associated with shorter PFS with anti-PD1/PDL1 based therapies. All patients had unique immune related expression profiles suggesting more extensive molecular profiling beyond tumor mutation burden and PD-L1 status may be essential to personalize treatment options such as individualized combination immunotherapy.

**Ethics Approval**

All investigations followed the guidelines of the UCSD Institutional Review Board for data collection (Profile Related Evidence Determining Individualized Cancer Therapy, NCT02478931) and for any investigational therapies for which the patients consented. OmniSeq’s analysis utilized deidentified data that qualified as non-human subject research under IRB protocol (BDR #073166) approved by Roswell Park Comprehensive Cancer Center (Buffalo, NY).

#### P77 Identifying major immune-related subsets of GI tumors for clinical purposes

##### Amy Early, MD^1^, Sarabjot Pabla, MSc, PhD, BS^2^, Jeffrey Conroy, BS^2^, Mary Nesline, MS^2^, Sean Glenn, PhD^2^, Felicia Lenzo^2^, Antonios Papanicolau-Sengos, MD^2^, Blake Burgher, BS, RN^2^, Vincent Giamo, BS, MS^2^, Jonathan Andreas, MS^2^, Yirong Wang, MS^2^, Carl D. Morrison, MD, DVM^2^

###### ^1^Roswell Park Comprehensive Cancer Center, Williamsville, NY, USA; ^2^OmniSeq, Inc., Buffalo, NY, USA

####### **Correspondence:** Carl D. Morrison (carl.morrison@omniseq.com)

**Background**

Gastrointestinal (GI) tumors, both colorectal and non-colorectal, have a low response rate to immune checkpoint inhibitors (ICIs) outside of the setting of microsatellite (MSI) unstable. Currently there is uncertainty how to evaluate microsatellite stable GI tumors for evidence of checkpoint blockade as PD-L1 IHC has minimal applications in this setting. Furthermore understanding other mechanisms of immunosuppression in GI tumors, such as myeloid or metabolic suppression and the degree of CD8+ T-cell infiltration, can have profound influence on the selection of immunotherapies in this patient population.

**Methods**

131 MSI stable GI tumors (82 colorectal, 41 pancreatic, 8 small bowel) from multiple institutions were evaluated for PD-L1 expression by IHC, TMB (DNA-seq), and expression of 54 immune-related genes (RNA-seq) that are the target of multiple immunomodulatory immunotherapeutics or evaluate tumor infiltrating lymphocytes (TILs) in a CLIA setting.

**Results**

PD-L1 IHC was positive (TPS>=1%) in 32 (24%) of cases, but with only 3 (2%) of cases strongly positive (TPS>=50%). In PD-L1 IHC negative cases (n=99; TPS<1%) the corresponding PD-L1 RNA-seq value was low in 88 (89%) and moderately high to high in 11 cases (11%). A high macrophage content was identified in 50 (31%) cases indicative of strong myeloid suppression. All of these cases over expressed one or more myeloid-related immunotherapeutic targets including CCR2, CSF1R, or TGFB1. The majority of these 50 cases (n=37; 74%) also over expressed VISTA or TIM3, or both, indicating that over expression of these two checkpoint blockade receptors are strong indicators of myeloid suppression. In a similar fashion PD-L2 was frequently over expressed in these 50 cases (n=23; 46%). While a low number of CD8+ T-cells were common in cases with strong myeloid suppression (6/50;12%) it was more common in the remaining 81 cases at 47% (38/81). This latter group of cases with few exceptions can be aptly described as immune deserts.

**Conclusions**

Using a more sophisticated approach to evaluating the tumor microenvironment in GI tumors 3 major groups including PD-L1 positive, myeloid suppression, and immune deserts can be identified that has major implications for the application of precision immunotherapy for this patient population.

**Ethics Approval**

OmniSeq’s analysis utilized deidentified data that qualified as non-human subject research under IRB protocol (BDR #080316) approved by Roswell Park Comprehensive Cancer Center (Buffalo, NY).

#### P78 More sensitive identification of T-cell receptor beta rearrangements with an augmented transcriptome method

##### Eric Levy, PhD, Sean Boyle, PhD, Gabor Bartha, Pamela Milani, Robin Li, Shujun Luo, Rena McClory, John West, MBA, Richard Chen

###### Personalis, Inc., Menlo Park, CA, USA

####### **Correspondence:** Eric Levy (eric.levy@personalis.com)

**Background**

With the growth of new immunotherapies, there is an increasing need for comprehensive immuno-genomic profiling of tumors to identify new potential biomarkers. This includes neoantigen identification, HLA, immuno-modulators, tumor microenvironment, and T-cell receptor (TCR) repertoire. However, limited sample amount, formalin-fixed paraffin embedded tissue (FFPE) degradation, and cost of multiple sequencing assays pose a significant barrier to comprehensive immuno-genomics biomarker characterization in clinical trials.To address these challenges, we developed an augmented, immuno-oncology optimized exome/transcriptome platform (ACE ImmunoID) that can also identify abundant TCR clones, from limited FFPE tumor biopsy samples.

**Methods**

We designed the next generation of our ACE ImmunoID platform to augment RNA profiling of TCR and BCR, including TCR beta. We next characterized the performance of our platform at profiling TCR beta. First, we analyze the impact of sequencing depth and input material amount on the observed repertoires of diverse PBMC samples. We test LOD by diluting well-characterized clonal T-cell line samples into PBMCs. Finally, we analyze patient- derived FFPE and FF tumors to understand the profiles of tumor-infiltrating immune repertoires and effects of FFPE damage.

**Results**

We observe that at our specified 400ng of fragmented input RNA and 100M cluster sequencing depth, we detect approximately 26,000 clones, enough to get a picture of the clonality of a sample. Our platform has higher sensitivity to TCR clones compared to non-augmented transcriptome methods at a comparable depth of sequencing. Furthermore, in comparison to a commercially-available deep TCR kit, we identify 93.9% of the top 1000 clones, showing that we confidently identify high-abundance clones. We also are able to reliably identify clones down 0.0004% RNA by mass in the mixture. We further tested on FFPE samples, showing our platform works well with degraded RNA.

**Conclusions**

Our ACE ImmunoID platform has been designed to enable sensitive detection of major TCR repertoire clones in addition to comprehensive biomarkers from exome/transcriptome results. Here we demonstrate that our platform achieves both a higher sensitivity for TCR clones compared to non-augmented transcriptome approaches and a high concordance with the top abundance clones derived from targeted TCR methods. We show our method is feasible with FFPE samples, making it practical for clinical trial use. In summary, by combining exome/transcriptome with TCR characterization into a single assay, our ACE ImmunoID platform enables comprehensive immuno-genomics characterization of a tumor sample while reducing overall sample requirements and cost.

#### P79 Change in neutrophil to lymphocyte ratio during treatment with immune checkpoint inhibitors predicts survival in patients with advanced cancer

##### Mingjia Li, MD, Dan Spakowicz, PhD, MS, Jarred Burkart, Sandip Patel, Marium Husain, MD, MPH, Kai He, MD, PhD, Carolyn Presley, Erin Bertino, Peter Shields, David Carbone, MD, PhD, Claire Verschraegen, MD, Greg Otterson, Kari Kendra, Mingjia Li, MD, Dwight Owen

###### The Ohio State University, Columbus, OH, USA

####### **Correspondence:** Dwight Owen (Dwight.Owen@osumc.edu)

**Background**

Baseline neutrophil to lymphocyte ratio (NLR) is known to be prognostic for patients with many cancer types treated with immune checkpoint inhibitors (ICI), including non-small cell lung cancer (NSCLC). We evaluated NLR at baseline and during treatment for patients who received ICI to evaluate the prognostic value of the change in NLR over time.

**Methods**

A retrospective review of patients with advanced cancer who received ICIs from 2011 to 2017 at the Ohio State University was performed with IRB approval. NLR was calculated as ratio of absolute neutrophil/lymphocyte counts, and considered elevated if ≥ 5. Overall Survival (OS) was calculated from the initiation of ICI to death of date or last follow-up. Significance of Cox Proportional-Hazards models were evaluated by log-rank test at Alpha = 0.05. All calculations were performed using the survival and survminer packages in R.

**Results**

**Chart 1 (abstract P79).**
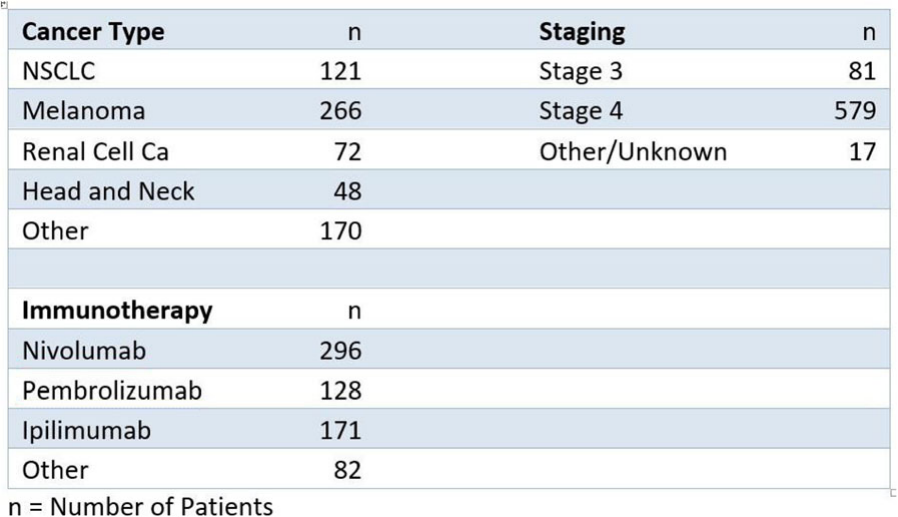
See text for description.

677 patients were included in the analysis. NLR was collected at the initiation of ICI and at least one time after (median 21, IQR 8 days, Chart 1).Patients with baseline NLR ≤5 had median OS 592 days (95% Cl: 497-681) compared to median OS 224 days (95% Cl: 158-269) for patients with baseline NLR >5, P<0.001 (Figure 1). Patients with on-treatment NLR ≤5 had median OS 616 days (95% CI: 532-878) compared to median OS 177 days (95% CI: 143-242, P< 0.001 for patients with on-treatment NLR >5 (Figure 2). Subgroup analysis of 121 NSCLC patients at baseline and on-treatment NLR showed similar prognostic value (Figure 3 and 4).For patients with baseline NLR >5 but where on-treatment NLR normalized to ≤5, there was improved median OS of 433 days (95% Cl: 304-NA) compared to median 150 days (95% Cl: 120-214) for patients when NLR remained high, P< 0.001 (Figure 5). Similar results were seen in NSCLC patient with baseline NLR >5 with on-treatment normalization of NLR (P=0.0018, Figure 6).

**Conclusions**

We confirmed the prognostic value of baseline NLR in patients with advanced cancer treated with ICI, including metastatic NSCLC. We demonstrated that change in NLR over time may identify patients with poor prognosis at baseline who nevertheless benefit from ICI. To our knowledge, the association between dynamic changes in NLR during treatment with ICI and survival have not previously been reported in NSCLC. This biomarker is especially attractive because NLR can be easily obtained from routine labs.

**Ethics Approval**

The study was approved by the Ohio State University Institutional Review Board, approval number # 2016C0070

**Fig. 1 (abstract P79).**
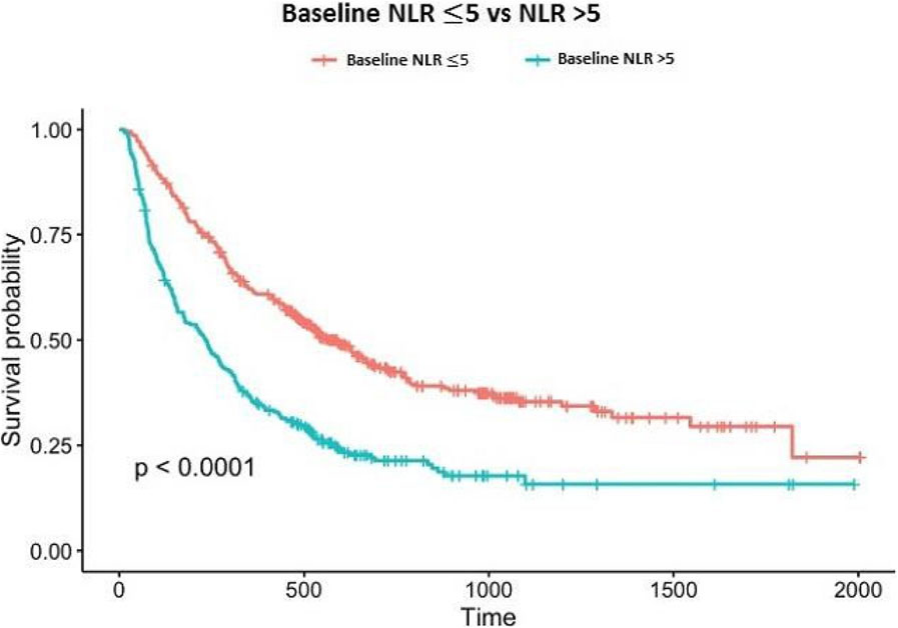
See text for description.

**Fig. 2 (abstract P79).**
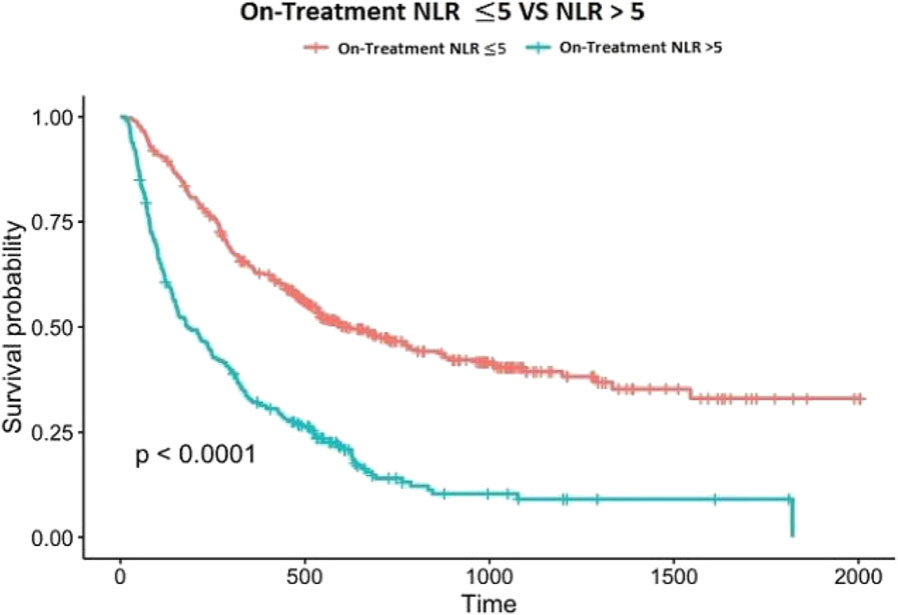
See text for description.

**Fig. 3 (abstract P79).**
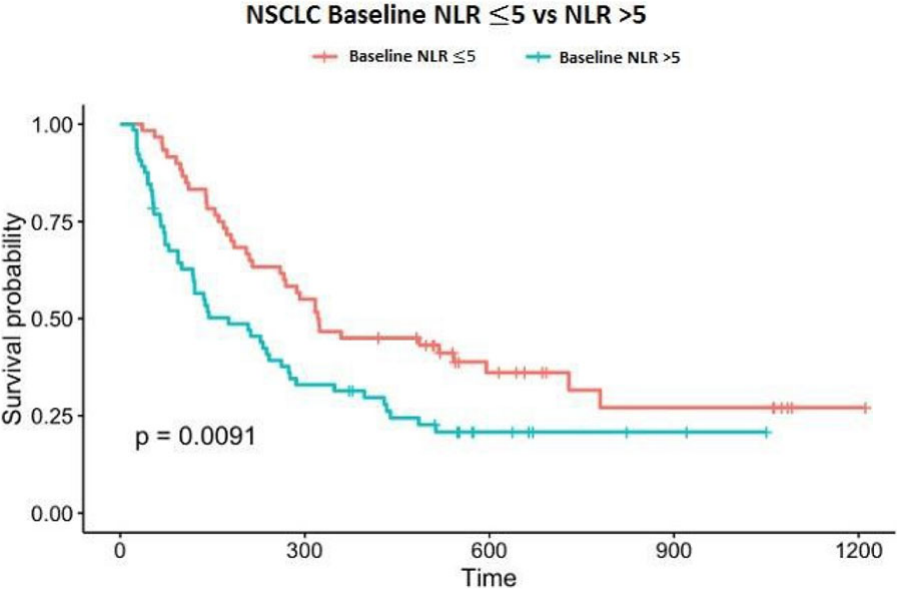
See text for description.

**Fig. 4 (abstract P79).**
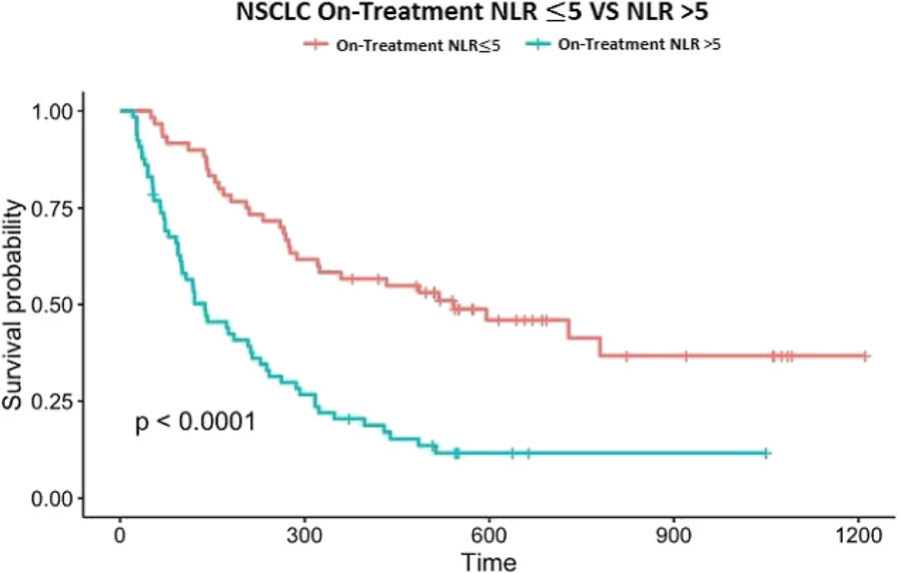
See text for description.

**Fig. 5 (abstract P79).**
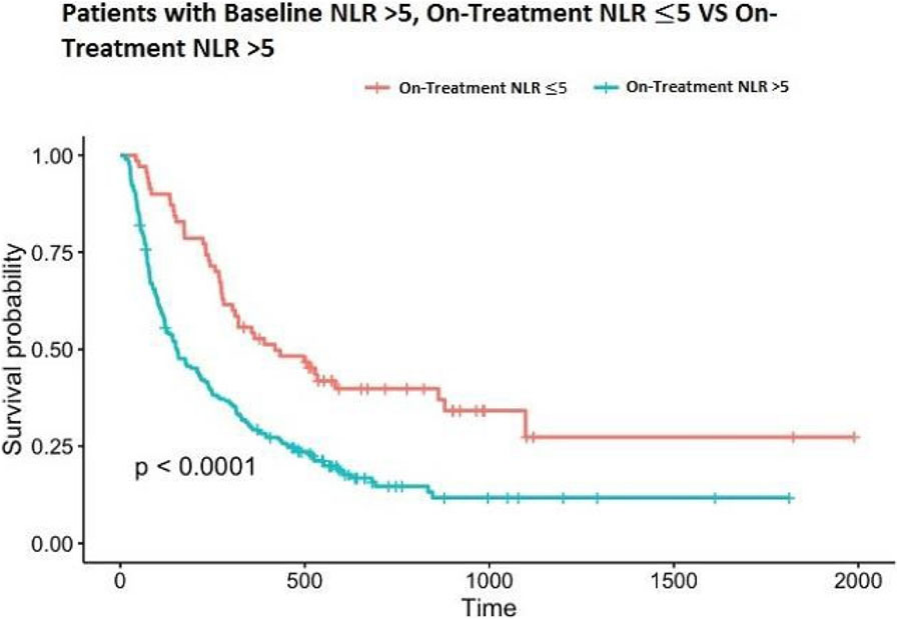
See text for description.

**Fig. 6 (abstract P79).**
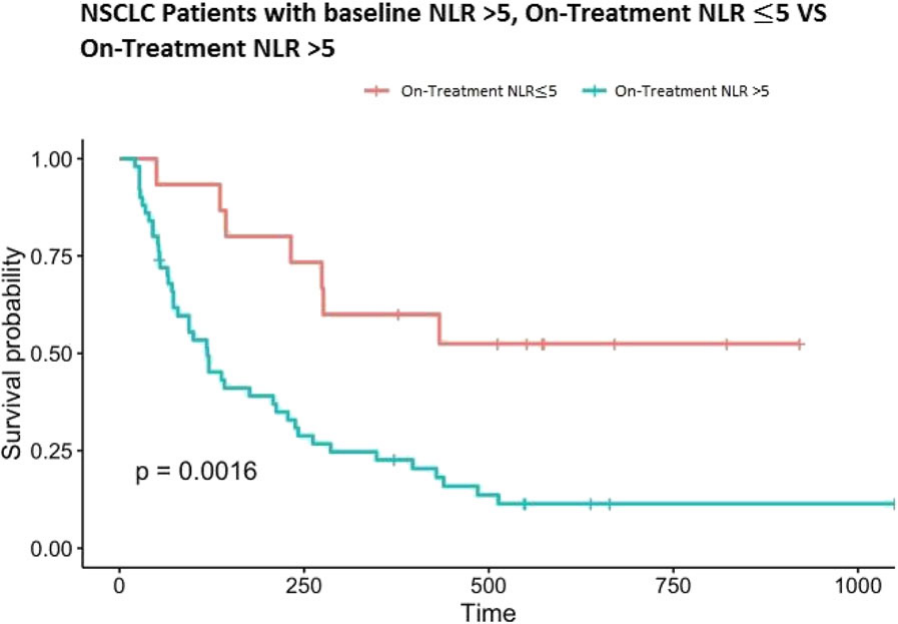
See text for description.

#### P80 Day-to-day profiling of T cell activation by measuring cellular markers and secreted cytokines in a rapid multiplexed cell/bead mixture assay

##### Zhaoping Liu, PhD (zhaopoing.liu@sartorius.com)

###### Sartorius, Albuquerque, NM, USA

**Background**

Optimized adoptive cell therapy protocols, and profiling drug candidates against immuno-oncology targets such as immune checkpoint proteins requires precise monitoring of ex vivo activation of human T lymphocytes. Here we describe the development of a large scale multiplexed assay using high throughput flow cytometry to daily profile T cell activation in human PBMCs treated with different activators (Figure 1).

**Methods**

On Day 0, PBMCs were stained with cell proliferation tracing dye before being plated into a 96-well plate. Cells were treated and cultured with this protocol: 3 different T cell modulators—CD3/CD28magnetic beads, phytohemagglutinin (PHA), and Staphylococcal enterotoxin B (SEB)—with 12 serial titrations and duplicate wells per dose. On culture Days 1, 3, and 6, cell and supernatant mixture samples were removed from the culture plate and evaluated without dilution using a multiplex cell/bead mixture assay that combined cell phenotype, T cell activation markers, cell proliferation, cell viability measurements and secreted cytokine analysis measured by bead-based ELISA. In each sample well of the assay plates, secreted levels of 3 cytokines (IL-4, IFNg and TNFa) were quantified by the standard curves generated from the standard wells in the same assay plate. In addition, over the course of a six-day activation, we generated multiple cellular readouts identifying the presence of different T cell subpopulations expressing early and late T cell action markers CD69, CD25 and HLA-DR. Cell proliferation, cell viability and cell number for different subpopulations were also evaluated. Each plate was read on the iQue Screener Plus system in approximately 20 minutes (Figure 2).

**Results**

In total, we measured 16 endpoints and generated 1152 data points per 96-well assay plate and 3456 data points from 3 assay plates ( days). We also acquired a total of 144 EC50s or IC50s (16-endpoint EC50/IC50s x 3 Days x 3 Activators), demonstrating the unique signature pattern of 3 different modulators on T cell activation (Figure 3).

**Conclusions**

These large-scale experiments with high throughput flow cytometry provide extensive T cell activation profiles with rapid assay turnaround time. The use of these assays can provide valuable information for optimizing immuno-oncology drug development such as checkpoint inhibitors and adoptive cell therapy development and manufacturing protocols.

**Fig. 1 (abstract P80).**
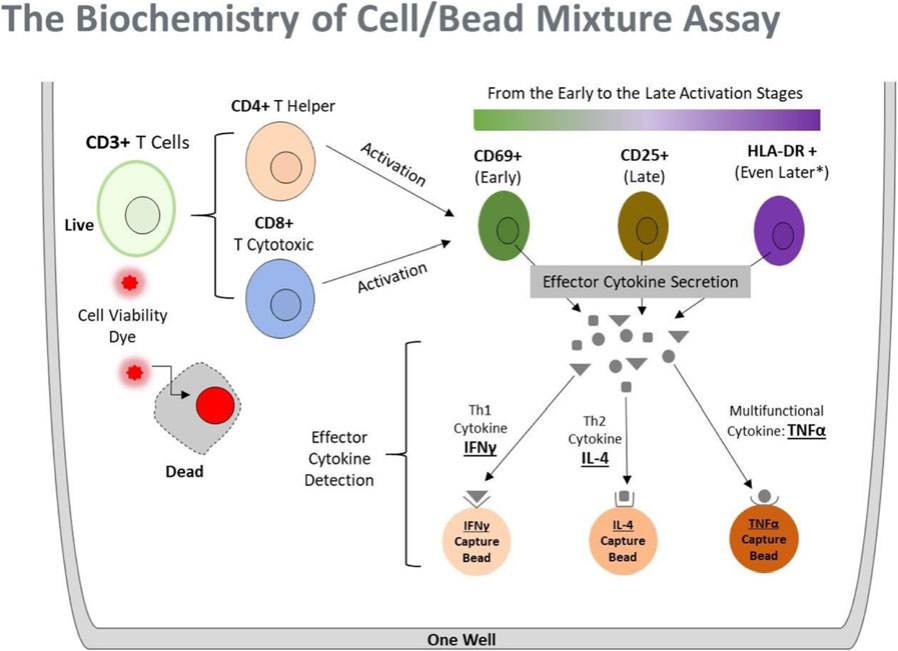
Assay biochemistry

**Fig. 2 (abstract P80).**
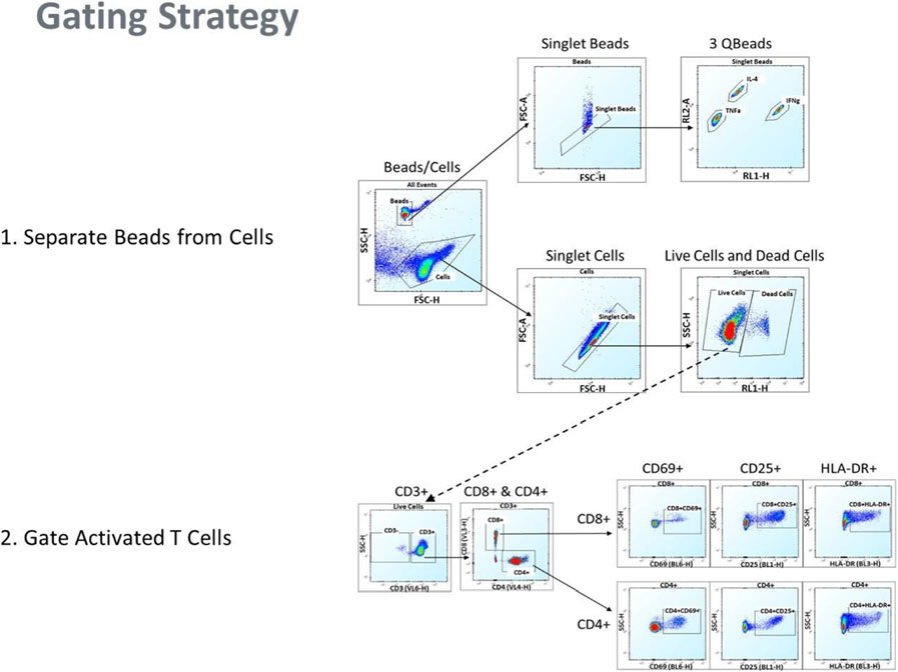
Gating strategy

**Fig. 3 (abstract P80).**
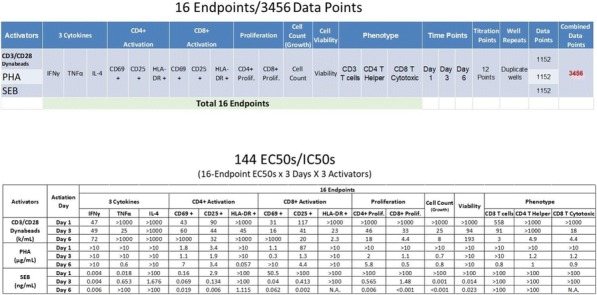
High content readout

#### P81 Germline encoded TRBV polymorphism predicts adverse events during checkpoint blockade immunotherapy

##### Timothy Looney, PhD, Geoffrey Lowman, PhD, Asha Kamat, PhD, Fiona Hyland

###### Thermo Fisher Scientific, South San Francisco, CA, USA

####### **Correspondence:** Timothy Looney (timothy.looney@thermofisher.com)

**Background**

Identifying predictive biomarkers for immune related adverse events (IRAEs) during immunotherapy is a key objective of current immuno-oncology research. Polymorphism within the TCRB variable gene (TRBV) has been implicated in autoimmune disease and may be mechanistically linked to IRAEs. Efforts to evaluate TRBV polymorphism by traditional approaches such as whole genome sequencing (WGS) have been hampered by the repetitive nature of the TCRB locus and incomplete genome assembly. Here we employed a novel long-amplicon TCRB repertoire sequencing approach to evaluate the link between TRBV polymorphism and adverse events in 54 Caucasians receiving checkpoint blockade immunotherapy for cancer.

**Methods**

To circumvent the challenge in measuring TRBV polymorphism by WGS, we instead employed next-generation sequencing of rearranged TCRB chains using RNA extracted from peripheral blood. Our strategy utilized multiplex PCR via framework 1 and constant gene primers to create ~330 bp amplicons spanning the three beta chain CDR regions including the germline-encoded framework and CDR1 and 2 regions of the receptor. Resultant amplicons were sequenced via the Ion Torrent, annotated by comparison to the gold-standard IMGT database, then mined to construct TRBV allele profiles for each individual including, where detected, novel alleles not found in the IMGT database. Finally, we correlated TRBV allele profiles with adverse events annotations to detect alleles or sets of alleles associated with severe (grade 3 or higher) adverse events following immunotherapy.

**Results**

Sequencing of TCRB libraries yielded on average 29k clonotypes per individual with mean evenness (normalized Shannon entropy) of .85. Principal component analysis and k-means clustering of TRBV allele profiles revealed the presence of 4 major sets of coincident variable gene alleles which we term haplotype groups. The incidence of severe adverse events varied markedly across haplotype groups: one group, comprising approximately one fourth of the sample set, appeared completely protected against grade 3 or higher adverse events (0% incidence), while up to 57% of individuals in other haplotype groups had severe adverse events (p = 2.4E-3, Fisher’s exact test).

**Conclusions**

These data suggest that germline-encoded TRBV polymorphism may play a mechanistic role in autoimmune toxicity during checkpoint blockade immunotherapy. We find that a subset of Caucasians appear to be at low risk of IRAEs and thus may be particularly well suited for immunotherapy regimens having elevated incidence of toxicity. Current and future studies will further explore the utility of TRBV polymorphism as a predictive biomarker for IRAEs.

#### P82 Peripheral blood TCRB repertoire convergence and clonal expansion predict response to anti-CTLA-4 monotherapy for cancer

##### Li Zhang, MD PhD^2^, Timothy Looney, PhD^1^, David Oh, MD, PhD^2^, Denise Topacio-Hall, BS, MA^1^, Lawrence Fong, MD^2^

###### ^1^Thermo Fisher Scientific, South San Francisco, CA, USA; ^2^University of California - San Francisco, San Francisco, CA, USA

####### **Correspondence:** Li Zhang (li.zhang@ucsf.edu)

**Background**

Tumor antigen-driven selection may expand T cells having T cell receptors (TCRs) of shared antigen specificity but different amino acid or nucleotide sequence in a process known as TCR convergence. Efforts to evaluate the biomarker utility of TCR convergence through TCRB repertoire sequencing have been hampered by the base substitution error rate of the Illumina platform, given that such errors may create artifacts resembling TCR convergence. Here we leverage the low base substitution error rate of the Ion Torrent platform to evaluate convergence as a predictive biomarker for response to anti-CTLA-4 monotherapy in a set of 22 individuals with cancer. For context, we compared convergence values obtained using this platform to those for the same samples interrogated with Illumina-based TCRB repertoire sequencing. Finally, we examined whether TCR convergence may be combined with measurements of clonal expansion to improve prediction of immunotherapy response.

**Methods**

Total RNA extracted from pretreatment peripheral blood leukocytes (PBL) from 22 recipients of Ipilimumab monotherapy. TCRB repertoire libraries were constructed by multiplex PCR via the Oncomine TCRB-LR assay, then sequenced using the Ion Torrent S5 to a target depth of 1.5M raw reads per library. To evaluate convergence within each repertoire we searched for instances where TCRB rearrangements were identical in amino acid space but had distinct nucleotide sequences within the CDR3. For a subset of samples, TCRB sequencing was performed in parallel via Illumina-based approaches.

**Results**

Sequencing of TCRB libraries yielded on average 31k clonotypes per individual with mean evenness (normalized Shannon entropy) of .82. TCR convergence was elevated in pretreatment PBL of responders compared to non- responders (mean frequency .022 vs .009; p=.03, Wilcoxon), and could discriminate responders from non- responders (AUROC = .77). Pretreatment evenness was reduced in responders vs non-responders and also predictive of response (AUROC = .74). A logistic regression model combining both features improved prediction of response (AUROC = .89).

**Conclusions**

These data suggest that PBL TCR convergence may serve as a predictive biomarker for response to anti-CTLA-4 monotherapy, potentially in combination with other immune repertoire features. Notably, measurements of TCR convergence appear to be sensitive to base substitution sequencing errors. These results highlight the impact of different sequencing approaches for assessing TCR repertoire.

#### P83 T cell receptor beta immune repertoire sequencing in several FFPE tissue types – Interrogation of the tumor microenvironment in archived tissue samples

##### Denise Topacio-Hall, BS, MA, Lauren Miller, BS, Elizabeth Linch, BS, Alice Zheng, PhD, Geoffrey M. Lowman, PhD, Timothy Looney, PhD, Mark Andersen, PhD

###### ThermoFisher Scientific, Carlsbad, CA, USA

####### **Correspondence:** Geoffrey M. Lowman (geoffrey.lowman@thermofisher.com)

**Background**

Immune repertoire sequencing is a valuable tool for studies of the tumor microenvironment and potential immune responses to cancer immunotherapy. Here we describe a T cell receptor beta (TCRβ) sequencing assay that leverages the low sample input requirements of AmpliSeq library preparation technology to extend the capability of targeted immune repertoire sequencing to include FFPE samples which can often be degraded and in short supply.

**Methods**

Evaluation of the highly diverse CDR3 region of TCRβ allows for T cell clone identification and frequency measurement. We demonstrate assay functionality with input of RNA or DNA samples, as well as flexibility in sequencing throughput and sample multiplexing capability. T cell repertoires were evaluated from as low as 10ng to 1ug of input material of varying repertoire diversity, such as sorted T cells, peripheral blood leukocytes, fresh-frozen tissue, and FFPE tissue from a variety of normal and cancerous tissues such as lung, colon, brain, spleen, lymph node, and thymus.

**Results**

Accuracy is demonstrated through the evaluation of samples comprised of known numbers of sorted T cells or spike-in experiments using well-studied lymphoma rearrangements. In order to test functionality of the assay with a range of degraded input material, RNA was controllably degraded with heat treatment at 90-95°C. In these systematically degraded samples we observe a strong correlation (r = 0.97) between the percentage of RNA molecules over 200bp in length and the amount of productive repertoire reads that the assay produces, while maintaining performance levels with samples with RIN values approaching 2. T cell richness and diversity in repertoires measured from FFPE tissue samples vary, as expected, depending on sample quality, disease state, and tissue of origin. To aid in sample input determination we present a complimentary qPCR assay, specific for T cell markers, which allows for sample T cell quantification and acts to guide optimal sample input ranges for library construction.

**Conclusions**

These data introduce a T cell immune repertoire sequencing solution for applications in a wide range of sample types including challenging FFPE preserved tissues. This assay is capable of profiling repertoire metrics from samples over a large range of input amounts from several tissue types. In addition, we demonstrate use of a qPCR assay for quantification of sample T cell content to guide sample input for TCRβ immune repertoire sequencing with samples with highly variable T cell content.

#### P84 Quantitative evaluation of tumor-infiltrating lymphocyte subsets and PD-L1 expression in lung cancer brain metastases

##### Benjamin Lu, MD, Richa Gupta, Hailey Wyatt, Matthew Ribeiro, Tyler Stewart, Veronica Chiang, MD, Joseph Contessa, Adebowale Adeniran, Harriet Kluger, MD, Lucia Jilaveanu, MD, Kurt Schalper, MD, PhD, Sarah B. Goldberg, MD, MPH

###### Yale Cancer Center, New Haven, CT, USA

####### **Correspondence:** Sarah B. Goldberg (sarah.goldberg@yale.edu)

**Background**

Lung cancer brain metastases (BrM) are associated with prominent morbidity and mortality. PD-1/PD-L1 inhibitors are safe and clinically active in patients with BrM in non-small cell lung cancer (NSCLC). While PD-L1 expression is associated with increased tumor infiltrating lymphocytes (TILs) and sensitivity to PD-1/PD-L1 inhibitors in extracranial tumors, the level and association between these markers in lung cancer BrM is unknown. Using spatially resolved/multiplexed tumor tissue analysis, we performed a comparative analysis of PD-L1 and major TIL subsets in primary lung cancers, BrM, and extracranial metastases (ECM).

**Methods**

We studied formalin-fixed paraffin-embedded tumor samples from a retrospective collection of 94 stage I-IV lung cancer patients from Yale between 2002-2013 represented in a tissue microarray. In total, 40 primary lung cancers, 63 BrM, and 15 ECM were included. Paired samples included primary-BrM from 11 patients and BrM-ECM from 12 patients. TIL density was determined by a semi-quantitative pathologist-based, scoring system using H&E preparations. Multiplexed quantitative immunofluorescence was used to evaluate PD-L1, CD4 for helper T-cells, CD8 for cytotoxic cells, and CD20 for B-lymphocytes. Signal for each marker was measured in marker-selected tissue compartments using the Automated Quantitative Analysis (AQUA) platform. We studied the association between markers and major clinicopathologic variables, including overall survival.

**Results**

Lung cancer histology included adenocarcinoma 62.5%, squamous cell carcinoma 11.5%, small cell 9.4%, and other NSCLC 16.7%. Only 8.5% of patients received immune checkpoint inhibitors. TIL density by pathologist read was significantly lower in BrM compared with primary lung tumors (p<0.0001). BrM had significantly lower levels of CD4+ T-cells (p=0.0416), CD8+ T-cells (p=0.0003), and CD20+ B-lymphocytes (p=0.0058) than primary lesions. Levels of tumor PD-L1 were comparable between BrM and primary lung tumors or ECMs (p>0.05). However, PD- L1:CD8 ratios were significantly higher in BrM compared with primary tumors (p=0.0024) or ECM (p=0.0322) without differences in PD-L1:CD4 ratios (p>0.05). Paired sample analyses demonstrated similar trends, though statistical significance was not achieved. There was no association observed between overall survival and TIL density, levels of TIL subsets, or PD-L1 expression.

**Conclusions**

Despite having lower levels of major TIL subsets, lung cancer BrM displayed similar PD-L1 expression compared with lung primary cancers and ECM. The latter indicates differences in the adaptive immune modulation of PD-L1 in BrM compared with extracranial tumors, suggesting alternative TIL-independent mechanisms sustaining PD-L1 expression in BrM. A better understanding of how the PD-1 axis differs in the brain microenvironment may help improve anti-PD-1/PD-L1 efficacy and reveal additional therapeutic targets

**Ethics Approval**

The study was approved by Yale University's Institutional Review Board, HIC# 1310012801.

#### P85 Correlation of clinical response and pathologic treatment effect after 4 weeks of preoperative PD- 1 blockade in primary head and neck squamous cell carcinoma (HNSCC)

##### Adam Luginbuhl, MD, Jennifer Johnson, MD, Madalina Tuluc, MD, Stacey Mardekian, MD, Chandala Chitguppi, MD, Larry Harshyne, PhD, Ralph Zinner, MD, Joseph Curry, MD, David Cognetti, MD, Ulrich Rodeck, MD PhD, Athanassios Argiris, MD, PhD, Adam Luginbuhl, MD

###### Thomas Jefferson University, Philadelphia, PA, USA

####### **Correspondence:** Jennifer Johnson (jennifer.m.johnson@jefferson.edu)

**Background**

Nivolumab, a PD-1 inhibitor, has been integrated into the clinical management of recurrent or metastatic HNSCC and is being evaluated in earlier stages of this disease. It is unclear whether imaging modalities, such CT and MRI, accurately reflect tumor response to immune checkpoint inhibition due to treatment-induced inflammatory changes at tumor sites. We sought to explore the relationship of imaging and pathology findings in the context of an ongoing neoadjuvant trial of preoperative nivolumab with or without tadalafil in resectable HNSCC.

**Methods**

Patients (n=17) with resectable primary HNSCC received nivolumab 240 mg IV Q 2 weeks for 2 doses and were randomized 1:1 to also receive tadalafil 10 mg PO for 28 days or not. Surgery was performed 4 weeks after the first nivolumab infusion. Tumor volumes were assessed pretreatment and on the day of surgery by CT scan. Resection specimens were graded histopathologically by two pathologists. Percent of treatment effect was determined by dividing the area of tumor showing changes consistent with treatment effect (fibrosis with chronic inflammation, foamy macrophage reaction and multinucleated giant cells) by total area containing treated and residual tumor. Radiographic effect was determined both by modified iRECIST and investigator assessment. Fischer exact test was performed to assess association between radiological and pathological tumor response. Percentage shrinkage of tumor was calculated in both radiological and pathological modalities and strength of association was calculated using Pearson correlation coefficient.

**Results**

Imaging results were used to stratify patients into response categories ranging from progression, stable disease and partial/complete response. Radiographic tumor shrinkage was observed in 11/17 (65%) patients with 16-100% volume reduction. A statistically significant relationship was noted between the radiological findings and treatment effects confirmed histopathologically (p = 0.009). A strong correlation was observed between these two groups (Pearson’s r = 0.7185; p = 0.001). All patients with stable disease or radiographic progression (6/17) at 4 weeks had no evidence of treatment effect in pathologic specimens.

**Conclusions**

In this treatment naive cohort, imaging approaches accurately captured treatment responses validated by histopathologic assessment of HNSCC surgical specimens obtained after two doses of nivolumab.

**Acknowledgements**

Sidney Kimmel Cancer Center at Thomas Jefferson UniversityBristol-Myers Squibb

**Trial Registration**

NCT03238365

**Ethics Approval**

The study was approved by Thomas Jefferson University Institutution‘s Ethics Board, approval number #17P.210

#### P86 T-cell receptor (TCR) repertoire features associated with disease-free survival following infusion with marrow-infiltrating lymphocytes (MILs)

##### Eric Lutz, PhD^1^, Alex Hopkins, PhD^2^, LAKSHMI RUDRARAJU, MS^1^, Elizabeth DeOlivera^1^, Ido Weiss, PhD^1^, Rachel Gittelman, PhD^3^, Erik Yusko, PhD^3^, Kathryn Boland, DVM, PhD^3^, Ivan Borrello, MD^2^, Kimberly A. Noonan, Ph.D^1^

###### ^1^WindMIL Therapeutics Inc., Baltimore, MD, USA; ^2^Johns Hopkins University, Baltimore, MD, USA; ^3^Adaptive Biotechnologies, Seattle, WA, USA

####### **Correspondence:** Kimberly A. Noonan (noonan@windmiltherapeutics.com)

**Background**

MILs are an autologous T-cell product expanded from bone marrow (BM) being developed as a novel cell therapy for both hematological and solid malignancies. In a Phase I trial evaluating MILs in patients with advanced multiple myeloma, 6 (27.3%) of 22 patients achieved a complete remission (CR). Immune analyses demonstrated that the establishment of persistent tumor antigen-specific T cells in BM correlated with improved clinical responses [1].Herein, we sought to identify the repertoire of T cell clonotypes within MILs, including the subset that specifically recognize tumor antigens; to track and compare their frequencies in blood and BM before and after infusion; and to compare T cell repertoire characteristics, such as clonality, between clinical responders and non- responders.

**Methods**

The TCRb CDR3 was sequenced using Adaptive Biotechnologies’ immunoSEQ Assay and used to identify and track MILs T cell clonotypes. The immunoSEQ assay was used on 11 specimens (unsorted MILs, IFNg-capture- sorted tumor antigen-specific CD4+ and CD8+ T cells, and blood and BM collected pre-treatment and 60, 180 and 360 days post-infusion) from 6 patients (3 clinical responders who achieved a CR and 3 non-responders whose disease progressed) from the Phase I study.

**Results**

When cumulative frequencies of MILs T-cell clonotypes were tracked in BM and blood, there were significant differences between responders and non-responders. Responders had a lower frequency of clonotypes at baseline but showed larger and more persistent increases in the frequency of clonotypes in both BM and blood. At day 360, fold- change from baseline in the frequency of MILs in both compartments segregated responders from non-responders. In general, T-cell repertoires in MILs were highly polyclonal and no specific TCRb variable genes were enriched in tumor antigen-specific T cells suggesting that multiple antigens are targeted. In all 6 patients, MILs were more polyclonal than pre-expanded BM. However, starting repertoires were more polyclonal in responders, and responders had larger and more persistent post-infusion increases in clonality. At day 360, all 3 responders maintained an increase in clonality whereas clonality returned to baseline or lower in all 3 non-responders.

**Conclusions**

These data provide a 1st look at the repertoire of T cell clonotypes in MILs and how the repertoire evolves after treatment. The data also demonstrate the highly polyclonal nature of tumor antigen-specific T cells within MILs, which could provide an advantage against heterogeneous tumors.

**References**

1. Noonan KA, Huff CA, Davis J, Lemas M. V, Fiorino S, Bitzan J, Ferguson A, Emerling A, Borrello I. Adoptive transfer of activated marrow-infiltrating lymphocytes induces measurable antitumor immunity in the bone marrow in multiple myeloma. Sci. Transl. Med. 2015; 7: 288ra78.

**Ethics Approval**

The study was approved by the Johns Hopkins University IRB.

#### P87 Preliminary evidence of intratumoral activation and immunomodulatory effect of CX-072, a Probody therapeutic antibody prodrug targeting PD-L1, in a phase 1/2a trial

##### Susan K. Lyman^1^, Judi Gordon^1^, Amy DuPage^1^, Preeti Pramanik^1^, Bruce Howng^1^, Michael B. Winter^1^, Irina K. Popova^1^, Olga Vasiljeva^1^, James Jones^1^, Kenneth Wong, MA^1^, Victoria Singson^1^, Jennifer Richardson, PhD^1^, Beiyao Zheng, PhD^1^, Mark Stroh, PhD^1^, Lori Carman, RN^1^, Vanessa Huels^1^, Karen Autio, MS, MD^2^, Valentina Boni^3^, Daniel Cho, MD^4^, Javier Garcia-Corbacho^5^, Iván Victoria Ruiz^5^, Omid Hamid, MD^6^, Nataliya Uboha^7^, Elisabeth de Vries^8^, Anthony El-Khoueiry, MD^9^, Alexander Spira, MD, PhD, FACP^10^, Rachel Sanborn, MD^11^, Fiona Thistlethwaite, MD, PhD^12^, Hendrik-Tobias Arkenau^13^, Johanna Bendell^14^, Patrick Ott, MD, PhD^15^, Naiyer Rizvi, MD^16^, Matthias Will, MD^1^, W. Michael Kavanaugh, MD^1^, Aung Naing, MD, FACP^17^, Luc R. Desnoyers, Ph D^1^

###### ^1^CytomX Therapeutics, Inc., South San Francisco, CA, USA; ^2^Memorial Sloan Kettering Cancer Center, New York, NY, USA; ^3^Hospital Universitario HM Sanchinarro, Madrid, Spain; ^4^Laura and Isaac Perlmutter Cancer Center, New York, NY, USA; ^5^Hospital Clinic de Barcelona, Barcelona, Spain; ^6^The Angeles Clinic and Research Institute, A Cedars-Sinai Affiliate, Los Angeles, CA, USA; ^7^University of Wisconsin, Madison, WI, USA; ^8^University Medical Center Groningen, the Netherlands, Groningen, Netherlands; ^9^USC Norris Comprehensive Cancer Center, Los Angeles, CA, USA; ^10^Virginia Cancer Specialists, Fairfax, VA, USA; ^11^Earle A. Chiles Research Institute, Providence Cancer Center, Portland, OR, USA; ^12^The Christie Hospital NHS Trust and University of Manchester, Manchester, UK; ^13^Sarah Cannon Research Institute-UK, London, UK; ^14^Tennessee Oncology, Nashville, TN, USA; ^15^Dana Farber Cancer Institute, Boston, MA, USA; ^16^Columbia University Medical Center, New York, NY, USA; ^17^The University of Texas, Houston, TX, USA

####### **Correspondence:** Luc R. Desnoyers (Luc@cytomx.com)

**Background**

CX-072 is a Probody™ therapeutic antibody prodrug directed against PD-L1. Probody therapeutics are masked antibodies designed to be selectively activated within the tumor microenvironment by tumor-associated proteases. CX-072 is designed to reduce systemic immune-related toxicities of anti-PD-L1 therapy, especially in combination with other drugs, while maintaining antitumor activity. PROCLAIM-CX-072 is a first-in-human, phase 1/2, open- label dose-finding trial investigating the safety and maximum tolerated dose of CX-072 as monotherapy and in combination with ipilimumab or vemurafenib. PROCLAIM-CX-072 patients have metastatic or recurrent solid tumors or lymphomas for which approved PD-1/-L1–based therapy is not available. Here we present the initial results of the tissue-based biomarker program intended to evaluate the mechanism of action of CX-072 in patients from PROCLAIM-CX-072.

**Methods**

Tumor biopsy and matched plasma samples were collected during the screening phase and after dosing of CX-072.

PD-L1 and CD8 expression were analyzed using immunohistochemistry. Relevant tumor-associated protease activity was measured by tissue zymography. Intratumoral CX-072 unmasking and activation were measured using capillary immunoelectrophoresis. Gene expression was profiled by NanoString.

**Results**

Results of the first 13 evaluable biopsies obtained are reported here. Nine of 12 (75%) predose biopsies had detectable levels of relevant protease activity. Two of 4 (50%) biopsies from patients treated with CX-072 at 3 mg/kg and 4 of 4 (100%) biopsies from patients treated at ≥10 mg/kg had detectable intratumoral activation of CX- 072, and the concentration of activated CX-072 in tumors increased with increasing dose. The preliminary calculated estimate of tumor receptor occupancy for patients receiving the 10 mg/kg dose was similar to that targeted for the PD-L1 inhibitor atezolizumab [1], and was consistent with quantitative systems pharmacology model predictions. Notably, the concentration of activated CX-072 measured in human tumor samples was similar to that associated with efficacy in a syngeneic preclinical tumor model. Consistent with the inhibition of the PD-L1 pathway by CX-072, we found an increase in CD8+ T cells and elevation of cytotoxic T-cell markers in the tumor of the one CX-072 monotherapy patient whose biopsy met evaluability criteria. These data support selection of the 10 mg/kg dose for clinical expansion cohorts.

**Conclusions**

These preliminary results show the presence of relevant protease activity, intratumoral Probody therapeutic activation, and biological effect of a Probody therapeutic in human subjects treated with CX-072. Taken together with previous data demonstrating stability of the masked Probody therapeutic in systemic circulation [2], these results support proof-of-mechanism for the Probody platform.

**Acknowledgements**

Editorial support was provided by ApotheCom (San Francisco, CA).

**Trial Registration**

ClinicalTrials.gov, NCT03013491

**References**

1. Stroh, M, Winter H, Marchand M, et al. Clinical pharmacokinetics and pharmacodynamics of atezolizumab in metastatic urothelial carcinoma. Clin Pharmacol Ther. 2017;102:305-312.

2. Autio K, Arkenau H-T, O’Neil B, et al. Preliminary results of the first-in-human, dose-finding PROCLAIM-CX- 072 trial of the PD-L1 Probody therapeutic CX-072 as monotherapy in patients (pts) with advanced solid tumors. J Clin Oncol. 2018;36(suppl):abstr 3071.

**Ethics Approval**

The animal study was performed in accordance with the Guide for the Care and Use of Laboratory Animals under a protocol (AP203) approved by the Institutional Animal Care and Use Committee at CytomX. The CX-072 clinical study was approved by all participating sites institutional review boards as well as the overseeing Ethics Board.

#### P88 Quantitative assessment and standardization of the programmed death 1 ligand 1 (PD-L1) immunohistochemistry companion diagnostic assays

##### Sandra Martinez-Morilla, PhD^1^, John McGuire^1^, Patricia Gaule, PhD^1^, Lauren Moore^1^, Balazs Acs^1^, Delphine Cougot^2^, David Rimm, MD, PhD^1^

###### ^1^Yale University, New Haven, CT, USA; ^2^Horizon Discovery, Cambridge, UK

####### **Correspondence:** Sandra Martinez-Morilla (sandra.martinez-morilla@yale.edu)

**Background**

Programmed Death 1 Ligand 1 (PD-L1) Immunohistochemistry (IHC) is the only FDA approved predictive marker to identify responders to anti-PD1 axis drugs. Multiple PD-L1 IHC assays with various antibodies and cut-points have been used in clinical trials across tumor types. Comparative performance characteristics of these assays have been extensively studied qualitatively, but not quantitatively. Since PD-L1 is a continuous marker, we propose the use of a standardized PD-L1 Index TMA to objectively evaluate concordance between antibody assays for PD-L1 using quantitative image analysis.

**Methods**

A panel of 10 isogenic cell lines expressing various amounts of PD-L1 was developed by Horizon Dx and constructed as an Index Tissue Microarray (TMA). Identical but independent batches of isogenic cells lines were cultured to create 3 TMA batches at 3 separate timepoints to control for any batch effect that may occur. The TMAs were validated using a previously published quantitative immunofluorescence protocol (QIF-AQUA). Comparing antibodies E1L3N, SP142 and SP263, reproducibility was assessed between batches. We then compared US Food and Drug Administration (FDA)-approved 22C3, 28-8, SP142 and SP263 assays and E1L3N lab developed test (LDT). Digital image analysis was used to quantify chromogenic PD-L1 assays using the open-source QuPath platform.

**Results**

There was very high reproducibility between blocks (R2=0.875-0.995) with the IF assay. The IF concordance between antibodies was also extremely high (R2=0.986-0.987). The 4 FDA approved assays and the E1L3N LDT were compared on the Index TMAs using quantitative chromogenic assessment with QuPath. The assays for 22C3- FDA, 28-8-FDA, SP263-FDA and E1L3N-LDT were essentially identical. The SP-142-FDA assay failed to detect low expressing cell lines detected by the other 4 assays. Levey-Jennings analysis was done to show the value of using the index array over time as a standardization tool in the CLIA lab setting.

**Conclusions**

We have built a standardized Index TMA that spans the dynamic range of PD-L1 expression that shows reproducibility by QIF across independent blocks. Quantitative assessment of the 4 FDA assays and the E1L3N LDT shows the assays recognize the entire dynamic range in a reproducible manner, except for the SP-142 assay that fails to detect low PD-L1 expressers. We propose this commercial TMA as a useful standardization mechanism to compare results between institutions and to identify abnormalities while running routine clinical samples. A multi-institutional comparison study with this Index TMA with different assays and platforms is currently underway.

#### P89 Withdrawn

#### P90 Using artificial intelligence to predict response to immunotherapy

##### Anthony Milici, PhD, Navi Mehra, Jospeh S. Krueger, BS, PhD, Karen Ryall, PhD, BS, Jenifer Caldara, BS, Will Paces, BS, Kelsey Weigel, PhD

###### Flagship Biosciences, Branford, CT, USA

####### **Correspondence:** Jospeh S. Krueger (jkrueger@flagshipbio.com)

**Background**

PD-L1/PD-1 checkpoint blockade is the backbone for the myriads of combination therapies being developed; and thus effective PD-L1 IHC testing remains critical for predicting patient response to these therapies. Pathological interpretations applied to PD-L1 immunohistochemistry (IHC) as a response biomarker are becoming more complex, going beyond simple Tumor Proportion Score (TPS) and requiring more complex diagnostic algorithms which evaluate the role of PD-L1 expression in 1) The tumor cells; 2) Immune cells in the Tumor Microenvironment (TME); and 3) Tumor infiltrating lymphocytes (TILs); all whose spatial relationships are critical for understanding the immune contexture. This complex matrix of several different biological cell types and spatial relationships can quickly become impossible for a pathologist to record and report successfully.

**Methods**

Recent advances in computer computational ability, machine learning algorithms, and data science now allow the application of Artificial Intelligence (AI) methods to create data-rich profiles from Whole Slide Images (WSI) of tissue that capture this key tissue context information about PD-L1. In this study, tissue image analysis was applied to this setting to increase objectivity and reproducibility of scoring, and AI interpretation of the results used to increase accuracy and sensitivity of the diagnostic performance of existing PD-L1 IHC testing methods. In this manner, existing FDA approved PD-L1 IHC tests can be re-evaluated by AI approaches to create this value in the clinical setting, without change to the IHC assay or significant disruption in the normal procedures performed in pathology labs.

**Results**

In this study, we demonstrate how our WSI AI platform captures the different attributes of PD-L1 stained slides to create a summary, singular score or output for decision making. Flagship’s cTA® records the PD-L1 staining, morphological, organizational, and spatial aspects of a tissue section to create a more sophisticated scoring system and better diagnostic cutpoint in a cohort of patient tissues measured against clinical response.

**Conclusions**

By applying Flagship’s cTA® Artificial Intelligence to existing PD-L1 IHC CDx, clinical labs can now go beyond using tissue image analysis for improving objectivity and reproducibility, and can also create entirely new scoring approaches from existing PD-L1 IHC CDx for improved clinical performance.

#### P91 It’s pan-o’clock: Tumor and circulating lymphocytic pan-pathology targets of the failed immune response

##### Anne Monette, PhD^1^, Antigoni Morou, PhD^2^, Nadia Al-banna^3^, Louise Rousseau, RT^4^, Jean-Baptiste Lattouf, MD, FRCSC^2^, Sara Rahmati^5^, Tomas Tokar^6^, Daniel Kaufmann, MD^2^, Jean-pierre Routy^7^, Igor Jurisica, PhD^8^, Rejean Lapointe, PhD^2^

###### ^1^University of Montreal / University of Montreal Hospital Research Centre / Lady Davis Institute for Medical Research / Jewish General Hospital, Montreal, Canada; ^2^University of Montreal / University of Montreal Hospital Research Centre, Montreal, QC, Canada; ^3^McGill University / University of Montreal Hospital Research Centre, Montreal, Canada; ^4^University of Montreal Hospital Research Centre, Montreal, QC, Canada; ^5^University of Toronto / Krembil Research Institute, Toronto, Canada; ^6^Krembil Research Institute, Toronto, Canada; ^7^McGill University Health Centre Chronic Viral Illnesses Service and Division of Hematology, Montreal, QUEBEC, Canada; ^8^University of Toronto / Krembil Research / Toronto Western Hospital / Techna Institute, Toronto, ON, Canada

####### **Correspondence:** Anne Monette (anne.monette@mail.mcgill.ca)

**Background**

Tumor infiltrating lymphocytes are widely associated with positive outcomes, yet carry key indicators of a systemic failed immune response against unresolved cancer. Cancer immunotherapies can reverse their tolerance phenotypes, while preserving tumor-reactivity and neoantigen-specificity shared with circulating immune cells.

**Methods**

We performed comprehensive transcriptomic analyses to identify gene signatures common to circulating and tumor infiltrating lymphocytes in the context of clear cell renal cell carcinoma. Modulated genes also associated with disease outcome were validated in several other cancer types. Using bioinformatics, we identified practical diagnostic markers and actionable targets of the failed immune response.

**Results**

On circulating lymphocytes, a minimal set of genes could efficiently stratify patients from healthy control donors.

From their associations with resistance to cancer immunotherapies and microbial infections, we have uncovered not only pan-cancer, but pan-pathology failed immune response profiles of effector and antigen presenting lymphocytes.

**Conclusions**

A prominent lymphocyte-specific cell migration pathway, is central to a panoply of diseases and tumor immunogenicity, correlates with multi-cancer recurrence in patients, and identifies a feasible, non-invasive approach to pan-pathology diagnoses.

**Ethics Approval**

The study was approved by the CHUM research ethics board Ethics Board, approval reference number SL07.053.

**Consent**

Written informed consent was obtained from the all study participants by the CHUM kidney biobank.

#### P92 Predictive plasma proteomic biomarkers of immunotherapy toxicity in patients (pts) with metastatic melanoma (MM)

##### Meghan Mooradian, MD^1^, Xuesong Gu^2^, Donald Lawrence, MD^1^, Justine Cohen, DO^1^, Tatyana Sharova^1^, Genevieve Boland, MD, PhD^1^, Towia Libermann^2^, Ryan J. Sullivan, MD^1^

###### ^1^Massachusetts General Hospital, Boston, MA, USA; ^2^Harvard University, Boston, MA, USA

####### **Correspondence:** Ryan J. Sullivan (rsullivan7@mgh.harvard.edu)

**Background**

Mechanisms underlying immune checkpoint inhibition (ICI) efficacy and toxicity have yet to be fully elucidated and, to date, there are no reliable biomarkers predictive of the development of immune-related adverse events (irAEs) or efficacy.

**Methods**

Pts with MM who developed select irAEs (colitis, hepatitis and arthritis) while receiving ICI (anti-PD-1, anti-CTLA-4 or combination anti-PD-1/CTLA-4) were identified. Using SOMAscan (SomaLogic; Boulder, CO), a proteomics platform that enables measurement of 1,305 proteins, quantitative plasma protein profiles were generated from banked plasma samples to identify candidate protein biomarkers predictive of irAEs and of progression-free survival (PFS). Comparator samples from pts without toxicity served as matched controls. Baseline protein expression was assessed and in evaluable pts, change in protein expression pre- and post-treatment was performed. PFS data was captured.

**Results**

36 pts were tested; 28 with confirmed irAEs and 8 controls. Baseline expression of IL-17, CXCL10 and TGFβ1 was associated with irAE development. Baseline IL-17 and CXCL10 expression was especially prominent in cases of ICI-induced arthritis, whereas TGFβ1 was linked to both ICI-induced arthritis and colitis. Independent ELISA validation studies of the candidate biomarkers are underway and will be presented. The one-year PFS of the toxicity cohort was 54% and 65% in those treated with PD-1 inhibition +/- anti-CTLA-4, respectively. Elevated TGFβ1, as well as LGALS3 and DLL4, were associated with poorer outcomes.

**Conclusions**

This study demonstrates the feasibility of SOMAscan to identify potential biomarkers of toxicity and outcome.

Previous literature has linked IL-17 and CXCL10 in autoimmunity, and TGFβ1 with a more pleiotropic role in immune regulation and melanoma pathogenesis. Our findings illustrate their possible role in the development of irAEs and outcome. If confirmed to be mechanistically involved in irAEs, targeted inhibitors of these proteins may serve as effective methods to abrogate or even prevent ICI-induced toxicity while minimizing effect on efficacy.

**Ethics Approval**

The study was performed with the approval of the MGH IRB, under research protocol 11-181

#### P93 Pre-analytical variables affect myeloid-derived suppressor cell quantitation by flow cytometry

##### Chihiro Morishima, MD, Amy Wright, Angela Riggins, Minjun Apodaca

###### University of Washington, Seattle, WA, USA

####### **Correspondence:** Chihiro Morishima (chihiro@uw.edu)

**Background**

Background: Myeloid-derived suppressor cells (MDSC) have been found to play an important role in limiting immune responses in many disease states including cancer. Higher circulating MDSC levels have been associated with greater tumor burden, poorer response to immunotherapy, and poorer survival. Optimal measurement of MDSC levels could provide clinicians with a useful management and/or prognostic tool.

**Methods**

Methods: Whole blood was obtained from healthy and diseased subjects through a University of Washington Institutional Review Board-approved study, #51834. A nine color flow cytometric assay included fluorescently- labeled antibodies against CD45, CD3, CD19, CD20, CD56, CD16, HLA-DR, CD33, CD11b, CD14 and CD15, and BD Trucount beads for quantitation. Samples were analyzed using a BD LSRFortessa and FlowJo software v9.9.5. Total MDSC were defined as CD45+CD3-CD19-CD20-CD56-CD16-HLA-DR-CD33+CD11b+ cells, while the monocytic (M-MDSC) and granulocytic (G-MDSC) subsets were defined as CD14+ or CD15+, respectively.

**Results**

Results: To confirm that our MDSC whole blood (WB) assay identified the same cells reported by other groups using PBMC as source material, we performed standard Ficoll density centrifugation of WB. The MDSC population identified by our assay in WB (0.32% of CD45+ cells) was found among cells from the Ficoll interface (0.28% of CD45+), but not the Ficoll pellet (0.02% of CD45+). Surprisingly, the yield of total and M-MDSC was higher with EDTA compared to heparin tubes (median 68% and 83% greater, respectively) among 5 donors with simultaneous blood collection in the two tube types, tested within 4 hours of blood draw. In addition, the duration of time that WB was kept at room temperature prior to cell labeling affected the yield of MDSC identified. For blood collected in EDTA tubes, total MDSC numbers decreased slightly by medians of 9% (N=7) and 11% (N=5) at 8 and 24 hours, respectively. M-MDSC numbers decreased somewhat more by medians of 14% (N=7) and 53% (N=5) at 8 and 24 hours, respectively. Finally, bilirubin levels as low as 1.6 mg/dL could impair the accurate identification of MDSCs. After controlling for these pre-analytical factors, significant differences in MDSC levels were still found among patients with hepatocellular carcinoma (N=35) compared to healthy controls (N=35, p=0.001).

**Conclusions**

Conclusions: MDSC are a heterogenous group of cells, and their quantitation in WB can be affected by a number of pre-analytical variables. Consideration of these factors, and measurement using a material type that has not been manipulated, such as WB, is likely to yield the most accurate results.

#### P94 Measurement of adenosine and other immunomodulators in the tumor microenvironment by in vivo microdialysis

##### Nadege Morisot, PhD, Julien Roeser, PhD, Holden Janssens, PhD, Arash Rassoulpour, PhD

###### Charles River Laboratories, South San Francisco, CA, USA

####### **Correspondence:** Nadege Morisot (nadege.morisot@crl.com)

**Background**

Understanding the tumor microenvironment is essential to gaining a better understanding of cancer biology and pathophysiology, and to date has proven to be challenging. The ability to determine the levels of adenosine and/or other molecules in the extracellular environment of tumors could provide unique insight into molecular targets for novel therapies in the treatment of cancers.

**Methods**

We implemented in vivo microdialysis to measure oncomodulator levels in the tumor microenvironment of freely moving mice. Microdialysis probes were implanted within 4T1 or CT26 syngeneic tumors and in the contralateral rump of the same animals. Probes were perfused with a buffered solution and dialysate samples were continuously collected for several hours. Levels of 12 metabolites (adenosine, cAMP, cGMP, arginine, ornithine, putrescine, tryptophan, kynurenine, kynurenic acid, 3-hydroxy-kynurenine, anthranilic acid, and xanthurenic acid) and lactate in the dialysates were quantified by liquid chromatography–mass spectrometry (LC-MS). Additionally, some animals received erythro-9-(2-hydroxy-3-nonyl)adenine (EHNA) through the microdialysis probe to examine the effects of adenosine deaminase inhibition on metabolite levels.

**Results**

We first confirmed that probe implantation did not affect the growth of 4T1 and CT26 allografts by comparing the volume of probe-implanted and -free tumor. Next, we found that adenosine levels were significantly elevated in both 4T1 and CT26 tumors, whilst the levels of cAMP and cGMP were reduced compared to non-tumor dialysis. Application of EHNA significantly increased the levels of Adenosine, cAMP, and cGMP in 4T1 tumors compared to basal levels. Additionally, we found that the levels of several kynurenine pathway metabolites were reduced within 4T1 tumors compared to the contralateral side. Interestingly, putrescine levels were elevated in CT26 but not 4T1 tumors compared to non-tumor dialysis. Finally, we observed high levels of lactate in both 4T1 and CT26 allografts.

**Conclusions**

Together, we have demonstrated the ability to detect levels of several signaling molecules that are believed to play a role in cancer biology. Additionally, we have been able to show that the reported levels are specific to the tumor microenvironment, and not a global circulating phenomenon within the same animal. In vivo microdialysis in murine tumor models may help in elucidating the mechanisms by which therapies such as chemotherapy and immune checkpoint inhibitors modulate the tumor microenvironment, helping identify the next-generation of therapies in oncology.

#### P95 Modulation of adenosine levels in the tumor microenvironment following treatment with anti-PD1 antibodies and oxaliplatin: an in vivo microdialysis study

##### Arash Rassoulpour, PhD, Nadege Morisot, PhD, Julien Roeser, PhD, Holden Janssens, PhD

###### Charles River Laboratories, South San Francisco, CA, USA

####### **Correspondence:** Nadege Morisot (nadege.morisot@crl.com)

**Background**

Elucidating the mechanisms by which cancer treatments modulate cellular signaling within the tumor microenvironment represents a major challenge in cancer research. In addition, the lack of reliable measurement of anti-cancer drug penetration in solid tumor may slow the development of effective systemic drug delivery strategies. We propose that microdialysis of the tumor microenvironment in syngeneic tumor models coupled to liquid chromatography–mass spectrometry may help filling in these gaps.

**Methods**

Mice bearing MC38 syngeneic tumors received control or anti-PD1 antibodies (5 mg/kg, i.p.) treatment on post-inoculation day 6, 9, 12 and 15. Next, animals were implanted with microdialysis probes within MC38 allografts. Dialysate samples from the tumor microenvironment were continuously collected in freely moving animal. In a separate cohort of MC38-bearing mice with microdialysis probes implanted in the tumor and contralateral subcutaneous (SC) space, baseline dialysate samples were collected, and mice received an acute systemic administration with oxaliplatin (10 mg/kg, i.p.). Dialysates were collected for two hours after treatment. Levels of adenosine, inosine and cAMP as well as the concentration of oxaliplatin in the tumoral and SC dialysates were quantified by liquid chromatography–mass spectrometry (LC-MS).

**Results**

We found that chronic treatment with an anti-PD1 antibody treatment reduced the levels of adenosine and inosine in MC38 allografts compared to control treated animals. Intriguingly, cAMP levels were higher in the tumor dialysates from anti PD-1 treated mice compared to controls. Moreover, oxaliplatin treatment produced a rapid and transient increase in adenosine and inosine levels in MC38 tumors. In contrast, changes in the concentration of these metabolites were much less pronounced in the SC space. From the same samples we were able to quantify the levels of oxaliplatin, and found that the levels in the core of the tumor were significantly less that in the SC of the same animal.

**Conclusions**

Here we bring proof-of-concept evidence that pharmacological and pharmacokinetic changes can be measured within tumor allografts in freely moving animals. Utilizing in vivo microdialysis we are able to simultaneously measure the levels of oncomodulators and anti-cancer drugs. Our experimental method may help the development of effective oncology therapeutic strategies.

#### P96 Assessment of RNA turbulence and PD-L1 expression on tumor-infiltrating lymphocytes in breast cancer

##### Apoorva Mylavarapu, BS^1^, Scott Morris^2^, Maulik Patel, PharmD, PhD^3^, Sandip Patel, MD^4^

###### ^1^University of California at Los Angeles, David Geffen School of Medicine, San Diego, CA, USA; ^2^Paradigm Diagnostics, Phoenix, AZ, USA; ^3^Abbvie Inc., Redwood City, CA, USA; ^4^University of California San Diego, Moores Cancer Center, La Jolla, CA, USA

####### **Correspondence:** Maulik Patel (mpatel@abbvie.com)

**Background**

Triple negative breast cancer (TNBC) as a subtype is generally associated with poorer outcomes compared to other breast cancer subtypes. In this study, we explore the utility of a novel tissue-based biomarker, RNA turbulence score, derived from a panel of 56 gene targets. We describe the RNA turbulence landscape and examine potential association of RNA turbulence score with PD-L1 positive tumor infiltrating lymphocytes (TILs) in breast cancer tissue samples.

**Methods**

Biomarkers were examined in breast cancer tissue samples (N=516). Expression of PD-L1 on tumor/tumor infiltrating lymphocytes (TILs) was assessed by immunohistochemistry with two different anti-PD-L1 clones (22C3 and E1L3N). PD-L1 positivity for this analysis was defined as staining intensity ≥ 1+ with tumor/immune cell staining of >1%. Molecular testing was conducted at Paradigm Diagnostics utilizing a 56-gene panel for RNA turbulence assessment. RNA turbulence score was calculated by assessing the number of genes with mRNA over- expression by at least 5-fold or greater with a significance of p<0.01.

**Results**

Average RNA turbulence score was higher (12.45 vs. 10.18, Mann-Whitney p<0.001) in patients whose tumor samples were positive for PD-L1 expression on TILs (19%, N=55) than in negative samples (N=233). A total of 40% (18/45) of TNBC samples were positive for TILs expressing PD-L1. In contrast, 11% (8/70) of estrogen receptor/progesterone receptor (ER/PR) positive tumors had evidence of PD-L1 positive TILs. The greater percentage of samples expressing PD-L1 on TILs in TNBC samples than in ER/PR-positive samples was statistically significant (p=0.0005; Fisher’s exact test). Average RNA turbulence score was also higher in TNBC samples than in ER/PR-positive samples (11.96 vs. 9.79, Mann-Whitney p<0.01). ER/PR-positive samples had *ESR1* exclusively over-expressed with *AREG* and *ERBB2* relatively higher in expression in comparison to TNBC. In contrast, the top three genes exclusively over-expressed in TNBC were *VEGFA*, *BAX*, and *LRP6*.

**Conclusions**

RNA turbulence score is greater in breast cancer patients whose tumor samples were also positive for PD-L1 expressing TILs. Additionally, we observe greater PD-L1 positive TILs in TNBC samples in comparison to ER/PR-positive samples. These findings suggest that RNA turbulence score may in part identify an increased immune active tumor microenvironment as assessed by PD-L1 positive TILs. Studies correlating RNA turbulence with response to anti-PD(L)-1 directed therapies in TNBC are warranted.

#### P97 Different functionality of CD8 PD-1-positive CD28-negative T cells in the periphery and in the tumor of lung cancer patients

##### Belinda Palermo^1^, Ornella Franzese^2^, Mariangela Panetta^1^, Giulia Campo^1^, Fabiana Cecere^1^, Virginia Ferraresi^1^, Gabriele Alessandrini^1^, Francesco Facciolo^1^, Gennaro Ciliberto, MD^1^, Paola Nistico', MD^1^

###### ^1^IRCCS - Regina Elena National Cancer Institute, Rome, Italy; ^2^School of Medicine, University of Tor Vergata, Rome, Italy

####### **Correspondence:** Paola Nistico' (paola.nistico@ifo.gov.it)

**Background**

The main objective of cancer immunotherapy is an efficacious control over tumor progression through the generation of a strong and persistent T-cell mediated immune response. CD8+ T cells are key players able to recognize and kill cancer cells, which experience phenotypic and functional changes due to the constant exposure to tumor-associated antigens, frequently in association with a dysfunctional state mediated by co-inhibitory receptors, including Programmed Death 1 (PD-1). Recently CD28 has been proven to be the main downstream target of PD-1- mediated signaling [1], and accordingly we have reported that a subset of Ag-specific CD8+ T-cell clones, characterized by PD-1 expression in the absence of CD28, show high proliferative capability and an AKT-dependent anti-tumor functionality sustained by ICOS [2-4]. Whereas, the co-expression of PD-1 and CD28 confers an exhausted phenotype and a defective anti-tumor functionality, reversible by PD-1 blockade. The role of these subsets in the tumor site is still not clarified, and there is a need to determine their presence and functionality to improve current therapies.

**Methods**

T cells were isolated from peripheral blood, tumoral (T) and adjacent non-tumoral (NT) tissue of lung cancer patients and analyzed by multiparametric flow cytometry for phenotypic characterization and polyfunctionality. Ag- specific CD8+ T cell clones (Melan-A and gp-100) were obtained as described [2-4].

**Results**

To elucidate the critical role of PD-1 in regulating CD8+ T-cell functionality, we have investigated the phenotypic and functional distribution of CD8+ T cells, with respect to PD-1 and CD28 expression, in the peripheral blood of patients with different solid tumors and at the tumor site of lung cancer patients. Preliminary results indicate that distinct PD-1+CD28- and PD-1+CD28+ CD8+ subsets could be found among T cells isolated from the periphery and both NT and T tissues. In the periphery we found that the differentiation and functional pattern of these T cells is similar to that identified in Ag-specific CD8+ T-cell clones [2-4], also in terms of GrzB, IFN-gamma and TNF- alpha production, ICOS and Ki-67 expression. Differently, when we compared periphery and tissue sites, we observed a heterogeneous phenotype and functionality of CD8+ T cells, also in terms of polyfunctionality and frequency of CD103+CD69+ T-resident as well as CD39+CD127- T-cell subsets, recently described as the major tumor-reactive T cells [5, 6].

**Conclusions**

These results highlight the critical role of PD-1 and CD28 molecules in regulating T-cell functionality and may help in the identification of biomarkers predicting the efficacy of anti-PD-1 therapy.

**Acknowledgements**

§ B.P. and O.F. contributed equally to this work. This work was supported by Associazione Italiana Ricerca sul Cancro (AIRC) IG n°15224

**References**

1. Hui E, Cheung J, Zhu J, et al. T cell costimulatory receptor CD28 is a primary target for PD-1-mediated inhibition. Science. 2017 Mar 31;355(6332):1428-1433.

2. Palermo B, Franzese O, et al. Antigen-specificity and DTIC before peptide-vaccination differently shape immune- checkpoint expression pattern, anti-tumor functionality and TCR repertoire in melanoma patients. OncoImmunology. In Press. https://doi.org/10.1080/2162402X.2018.1465163;

3. Franzese O, Palermo B, et al. Polyfunctional Melan-A-specific tumor-reactive CD8(+) T cells elicited by dacarbazine treatment before peptide-vaccination depends on AKT activation sustained by ICOS. OncoImmunology 2016. Feb 1;5(5):eP582

*Corresponding author email: rajesh.gottimukkala@thermofisher.com03;

4. Palermo B, et al. Dacarbazine treatment before peptide vaccination enlarges T-cell repertoire diversity of melan- a-specific, tumor-reactive CTL in melanoma patients. Cancer Res. 2010 Sep 15;70(18):7084-92.

5. Duhen T, Duhen R, Montler R et al. Co-expression of CD39 and CD103 identifies tumor-reactive CD8 T cells in human solid tumors. Nat Commun. 2018 Jul 13;9(1):2724.

6. Thommen DS, Koelzer VH, Herzig P et al. A transcriptionally and functionally distinct PD-1+ CD8+ T cell pool with predictive potential in non-small-cell lung cancer treated with PD-1 blockade. Nat Med. 2018 Jun 11.

**Ethics Approval**

The study was approved by Regina Elena National Cancer Institute's Ethics Board, approval number 1008/17

#### P98 High gene expression of estrogen and progesterone receptor is associated with increased T cell infiltration in patients with NSCLC

##### Michael Oh, MD, Jonathan Anker, MD, Young Kwang Chae, MD

###### Northwestern University Feinberg School of Medicine, Chicago, USA

####### **Correspondence:** Young Kwang Chae (young.chae@northwestern.edu)

**Background**

Recent reports have demonstrated that hormonal markers, including estrogen and progesterone receptor (ER and PR) status, may have prognostic and predictive relevance in non-small cell lung cancer (NSCLC). The precise impact of hormone signaling on clinical outcomes in NSCLC remains unclear, as initial studies have had diverging results. Here, we investigate the impact of hormone receptor status on tumor inflammation and survival in patients with NSCLC.

**Methods**

A dataset of NSCLC patients was obtained from The Cancer Genome Atlas (TCGA). RNA-Seq data was used to determined mRNA expression levels of ESR1 (ER-alpha), ESR2 (ER-beta), PGR (PR), and ARO (aromatase). Tumor infiltration by activated T cells was predicted using a previously-described method based on expression of immune metagenes [1]. Overall survival (OS) and progression-free survival (PFS) was assessed using Kaplan-Meier analyses with log-rank test.

**Results**

High levels of ESR1 was associated with significantly increased proportion of tumors infiltration by CD4+ (58% of tumors vs 19%, adjusted p < 0.001) and CD8+ activated T cells (55% vs 14%, adj. p < 0.001). Increased expression of PGR similar was associated with increased CD4+ (44% vs 18%, adj. p = 0.001) and CD8+ (48% vs 12%, adj p < 0.001) activated T cells. There were no significant differences based on ESR2 or ARO. These findings remained even after stratifying patients based on sex and histologic tumor type. In a multivariate logistic regression analysis, ESR1, PGR, and tumor mutation burden all were identified as independent factors predicting T cell infiltration.

However, greater expression of ESR1, PGR, or a combined measure of both genes did not confer greater overall or progression-free survival in this cohort.

**Conclusions**

Increased gene expression of estrogen receptor-α and progesterone receptor was associated with increased activated T cell infiltration in patients with NSCLC. The relevance of these findings will need be validated, potentially with clinical studies using immunotherapy based on hormone receptor status.

**References**

1. Angelova M, et al. Characterization of the immunophenotypes and antigenomes of colorectal cancers reveals distinct tumor escape mechanisms and novel targets for immunotherapy. Genome Biol. 2015;16:64.

#### P99 RRx-001 is a Phase 3-Ready small molecule dual inhibitor of CD47 and SIRPalpha

##### Pedro Cabrales, PhD^1^, Bryan Oronsky, MD, PhD^2^, Tony Reid, MD PhD^2^, Corey Carter, MD^2^

###### ^1^University of California San Diego, La Jolla, CA, USA; ^2^EpicentRx Inc., La Jolla, CA, USA

####### **Correspondence:** Bryan Oronsky (boronsky@epicentrx.com)

**Background**

CD47 binds to SIRPα on the surface of macrophages delivering a “do not eat” signal to suppress phagocytosis. To evade macrophage-mediated destruction, tumor cells frequently overexpress CD47. One area of intense interest is the targeting of CD47 with monoclonal antibodies (mAbs), three of which, Hu5F9-G4, CC-90002, and TTI-621, have proceeded to clinical trials [1]. However, these mAbs have been associated with severe hemolytic anemia and thrombocytopenia. RRx-001 is a minimally toxic small molecule that dually downregulates CD47 on tumor cells and SIRPα on macrophages and triggers tumor associated macrophage phagocytosis of tumor cells in vitro and in vivo. RRx-001 is entering Phase 3 trials for the treatment of multiple cancer indications.

**Methods**

The effect of RRx-001 on the expression of CD47 and SIRPα on macrophages was evaluated with Western blotting and flow cytometry. An in vitro phagocytotic assay was used to determine whether RRx-001 promoted engulfment of A549 tumor cells by macrophages. Transcriptional mRNA profiling in murine tumor associated macrophages (TAMs) was performed to analyze the cytokine profile of TAMs in the presence or absence of RRx-001. Finally, nude mice bearing A549 tumors were treated with RRx-001 in the presence or absence or absence of clodronate to determine the effect of macrophage depletion on RRx-001 anticancer activity.

**Results**

RRx-001 was shown to downregulate CD47 and SIRPα expression on tumor cells and macrophages, respectively, and to promote the phagocytosis of high-expressing CD47 A549 tumor cells. RRx-001 also stimulated the production of pro-inflammatory cytokines in TAMs. In tumor bearing mice, depletion of macrophages by clodronate reduced the antitumor effects of RRx-001.

**Conclusions**

RRx-001 is a Phase 3-ready small molecule innate immune checkpoint inhibitor, which triggers tumor associated macrophage phagocytosis of high-expressing CD47 tumor cells. Dual downregulation of CD47 and SIRPα by RRx- 001 results in TAM repolarization and phagocytosis of tumor cells. Depletion of macrophages by clodronate in tumor-bearing mice reduced the antitumor effects of RRx-001 and further suggests that the target of RRx-001 is the macrophage.

**References**

Liu X, Kwon H, Li Z, Fu Y. Is CD47 an innate immune checkpoint for tumor evasion? J Hematol Oncol. 2017;10:12. DOI: 10.1186/s13045-016-0381-z

**Ethics Approval**

All applicable international, national, and/or institutional guidelines for the care and use of animals were followed.

#### P100 Exploring dissociated human tissues as an alternative to fresh tissue for multiple downstream applications

##### Rebecca Parker^1^, Shawn Fahl, PhD^2^

###### ^1^Conversant Bio, Huntsville, AL, USA; ^2^Folio Conversant, Huntsville, AL, USA

####### **Correspondence:** Shawn Fahl (shawn.fahl@folioconversant.com)

**Background**

The foundation of the future of biomedical research requires access to highly-annotated primary human biospecimens. The logistical barriers of acquiring fresh tissue remains an impediment to advances in medicine, requiring the coordination of not only the tissue collection but also the downstream applications in the laboratory. Dissociation and cryopreservation of solid tissue provides a solution to this problem. These single cell suspensions remain viable following cryopreservation and ease the demands on large-scale experimental assays. Furthermore, these cells provide the ability to screen new biomarkers and therapeutic targets as they are uncovered without the need to source new fresh tissue.

**Methods**

Using this model, we have analyzed viably cryopreserved single cell suspensions generated from over 400 unique patients across 11 oncology indications by flow cytometry. Flow cytometry allows for the identification of cell surface marker expression on the single cell level and provides in-depth characterization of the cellular composition of the tumor microenvironment. This large-scale characterization revealed indication-specific trends to the tumor composition, which are vitally important considerations as the next-generation of therapeutic interventions are developed.

**Results**

N/A - Results pending late breaking submission requested.

**Conclusions**

Viably cryopreserved single cell suspensions from solid tissues provides numerous benefits to the logistical demands of sourcing fresh tissue. Using solid tumor indications as a model, we have demonstrated the utility of cryopreserved dissociated tumor cells to understand and screen the cellular composition of the tumor microenvironment. In particular, these results highlight the patient-specific heterogeneity of the tumor microenvironment, but also demonstrates indication-specific trends that are crucial when developing future immunotherapies.

#### P101 Evaluation of ctDNA mutations detected in plasma as potential correlate of immunotherapy efficacy in NSCLC patients

##### Namrata Patil, PhD^2^, Yuqiu Jiang, PhD^3^, Wei Zou^2^, Johnny Wu^3^, Stephanie Yaung^3^, Susan Flynn^2^, Maureen Peterson^2^, Eric Peters^2^, Priti Hegde, PhD^2^, John Palma^3^, Marcin Kowanetz, PhD^2^, David Shames, PhD^2^, Yuqiu Jiang, PhD^3^

###### ^1^Genentech/ Roche, South San Francisco, CA, USA; ^2^Genentech, South San Francisco, CA, USA; ^3^Roche Sequencing Solutions, Pleasanton, CA, USA

####### **Correspondence:** Namrata Patil (patil.namrata@gene.com)

**Background**

Multiple recent studies have shown that tumor mutation burden (TMB) may be a surrogate for the overall neoantigens likely to presented for an effective immune response. High TMB, including TMB measured in blood (Gandara et al. 2017), has been associated with clinical benefit with checkpoint inhibitors in several malignancies. The goal of this analysis was to evaluate whether the number of mutations detected in ctDNA by the AVENIO Surveillance panel (200kb) correlates with immunotherapy efficacy in NSCLC patients.

**Methods**

A subset of 375 baseline plasma samples from 2L+ NSCLC subjects enrolled in study OAK (NCT02008227) were analyzed using the AVENIO ctDNA Surveillance kit**, which covers 200 kb (Roche, Branchburg, NJ), from 375 pts. Data from 108 patient samples have been analyzed so far. The Surveillance kit contains 17 cancer driver genes and an additional 180 frequently mutated genes in cancer. This kit is capable of detecting four mutation classes: SNVs, fusions, CNVs and Indels. Tumor tissue samples from these patients were also analyzed for tumor mutation burden (tTMB) using the FoundationOne assay.

**Results**

102 samples of the 108 (94%) baseline plasma samples that passed QC metrics were successfully sequenced. All 102 samples had somatic variants detected. The median number of variants detected per patient was 7. Mutant molecules per milliliter (MMPM) was also assessed for each of the baseline samples. The median MMPM was 139, ranging from 1 to 1,972 for these 102 samples. Preliminary analysis of the number of variants detected with a limited filters algorithm was positively correlated (r=0.56) with tissue Tumor Mutation Burden (tTMB).

**Conclusions**

ctDNA testing with molecular barcoded sequencing and digital background error suppression of a small 197 gene panel offers high sensitivity for tumor variant detection (all 102 samples with variants detected). This study demonstrated that tumor variants can be detected in blood in pre-treatment samples using the AVENIO Surveillance kit. Preliminary analysis suggests that the number of mutations detected correlate with immunotherapy efficacy, specifically PFS. Analysis of all 375 pts as well as association with clinical benefit to atezolizumab will be presented.

**Trial Registration**

NCT02008227

**References**

1. Gandara DR, Pawel JV, Sullivan RN, Helland A, Han J, Aix SP, Rittmeyer A, Barlesi F, Kubo T, Park K, Goldschmidt JH, Gandhi M, Yun C, Yu W, Matheny C, He P, Sandler A, Ballinger M, Fehrenbacher L. Journal of Clinical Oncology. 2017; 35:15_suppl, 9001-9001.

#### P102 Localized measurement and clinical significance of OX40 and OX40L expression in human non-small cell lung cancer (NSCLC)

##### Angelo Porciuncula, PhD^1^, Micaela Morgado^1^, Sima Zacharek, PhD^2^, Maria Toki, MD, MSc^1^, Kostas Syrigos^3^, Vamsidhar Velcheti, MD FACP^4^, Joshua Frederick, PhD^2^, Roy Herbst, MD, PhD^1^, Kurt A. Schalper, MD, PhD^1^

###### ^1^Yale University, New Haven, CT, USA; ^2^Moderna Therapeutics, Cambridge, MA, USA; ^3^Athens School of Medicine, Athens, Greece; ^4^Cleveland Clinic, Pepper Pike, OH, USA

####### **Correspondence:** Kurt A. Schalper (kurt.schalper@yale.edu)

**Background**

Immunostimulatory therapies targeting co-inhibitory T-cell checkpoint pathways such as PD-1 and CTLA-4 produce lasting anti-tumor responses in a proportion of patients with diverse malignancies, including NSCLC. However, the majority of patients show primary resistance to treatment and a fraction of those initially responding have subsequent disease progression. Activation of co-stimulatory signals such as the OX40/OX40L pathway can favor anti-tumor immune responses and clinical trials modulating this pathway are currently ongoing. The expression, tissue distribution, biological associations and clinical significance of OX40/OX40L protein expression in human tumors remain largely unexplored.

**Methods**

Using formalin-fixed paraffin-embedded (FFPE) preparations from cell line transfectants and human tissue controls, we validated and standardized a multiplexed quantitative immunofluorescence (mQIF) assay for simultaneous and localized measurement of DAPI (all cells), cytokeratin for epithelial tumor cells (clone-AE1/AE2), OX40 (clone- SP195) and OX40L (clone-D6K7R). We used this panel to interrogate 619 stage I-IV NSCLCs from 3 retrospective cohorts represented in tissue microarray format (cohort#1 [Yale, n=280], cohort#2 [Greece, n=207]). In addition, we analyzed a collection of lung adenocarcinomas with molecular testing for major oncogenic drivers (cohort#3 [Yale, n=132]). The targets were measured in all cells of the preparation using fluorescence co-localization with DAPI and specifically in tumor (CK-positive) and stromal (CK-negative) cells. Associations between the markers and with major clinicopathological variables, driver mutations and survival were studied.

**Results**

Expression of OX40 protein was seen in 90% of NSCLCs in cohort#1 and 87% of cases in cohort#2. OX40 staining was predominantly located in stromal cells with membranous staining pattern. Detectable OX40L signal was identified in 9% of cases in the first cohort and 14% of the second collection with membranous/perinuclear staining pattern and predominant expression in CK-positive tumor cells. There was no clear association between OX40 and OX40L expression in the cohorts. The levels of OX40 and OX40L were not consistently associated with major clinicopathogical variables, level of T-cell infiltration or with the presence of oncogenic mutations in EGFR/KRAS in lung adenocarcinomas (cohort#3). Elevated levels of OX40L protein (but not OX40) were significantly associated with longer 5-year overall survival in both NSCLC cohorts (log-rank P=0.03 and P=0.04, respectively).

**Conclusions**

Our preliminary data show that OX40 protein is expressed in the majority of NSCLCs with predominant location in stromal/immune cells and does not segregate with a specific clinicopathological variant or molecular subtype. Elevated OX40L, observed in ~15% of NSCLCs, is unrelated with OX40 levels and has favorable prognostic value.

#### P103 Bulk and single-cell TCR and transcriptome profiling reveals significant phenotypic, spatial and temporal heterogeneity in the TIL repertoire of pancreatic cancer and melanoma patients

##### Chin Leng Tan^1^, Ignacio Heras-Murillo, MSc^1^, Katharina Lindner^1^, Aaron Rodriguez Ehrenfried^1^, Lena Appel^1^, Anna-Katharina Koenig^2^, Markus Buechler^2^, Ugur Sahin, MD^3^, Jessica Hassel^2^, Oliver Strobel^2^, Michael Flossdorf^4^, Rienk Offringa, PhD^1^, Isabel Poschke, PhD^1^

###### ^1^German Cancer Research Center, Heidelberg, Germany; ^2^Heidelberg University Hospital, Heidelberg, Germany; ^3^Johannes Gutenberg University Mainz, Mainz, Germany; ^4^Technical University of Munich, Germany, Heidelberg, Germany

####### **Correspondence:** Isabel Poschke (i.poschke@dkfz.de)

**Background**

Presence of tumor-infiltrating lymphocytes (TIL) is associated with good survival in many cancers, and harnessing of the T-cell response through checkpoint inhibition or infusion of ex vivo expanded TIL can result in tumor regression. While pancreatic ductal adenocarcinoma (PDA) has been considered a poorly-immunogenic tumor, we detect TIL infiltrates - and aggregates - in the majority of patients with primary resectable disease. PDA TIL phenotype and expansion capacity resemble that of melanoma TIL - our benchmark of an immunogenic tumor, and 80% of in vitro expanded PDA TIL cultures exhibit reactivity against autologous tumors.

**Methods**

To gain a better understanding of TIL reactivity, we performed repertoire profiling of freshly isolated TIL by T-cell receptor (TCR) deep- and high-throughput single-cell sequencing and transcriptomics, as well as phenotypic and functional analysis of bulk and single-cell expansion cultures.

**Results**

Intra-tumoral and intra-patient heterogeneity is significant and highly individual: TIL repertoires from multiple regions of the same tumor show an overlap between 8.4-100%. Lesions within the same patient share between 0-70% of TCRs and tend to overlap less if biopsies are not acquired concurrently, indicating a continuous turn-over or reshaping of TIL composition. Notably, repertoire-sharing is most prominent among the largest TIL clones, possibly explained by efficient migration/re-circulation of some clones, or their maintenance by ubiquitously expressed (tumor-)antigens. Enrichment of highly frequent CDR3 sequences within the TIL repertoire suggests in situ proliferation in response to tumor-derived antigens. Droplet-based TCR- and transcriptome-analysis of >6000 PDA TIL cells revealed that dominant T-cell clones exhibit multiple distinct phenotypes, enriched for markers associated with activation, proliferation and exhaustion. Importantly, TIL in vitro expansion - with protocols established for therapeutic TIL application- induces dramatic shifts in repertoire composition, resulting in loss of dominant clones and enrichment of bystander clones with high proliferative potential.

**Conclusions**

Our findings call for careful sampling and optimized culture conditions for TIL infusion products and illustrate the need to probe T-cell reactivity directly ex vivo. The heterogeneous TIL response implies that therapeutic efficacy of TCR gene therapy using tumor-dominant TCRs could be more consistent than that of TIL therapy. Emerging high- throughput single cell technologies and resulting data will facilitate rapid identification of relevant TIL populations.

**Ethics Approval**

The study was approved by the local ethics committee at the medical faculty of Heidelberg university and conducted in accordance with the declaration of Helsinki.

#### P104 Radiomic features that can predict response to PD-1 inhibitors in late-stage Non-Small Cell Lung Cancer are also associated with tissue-derived measures of immune response

##### Prateek Prasanna, PhD^1^, Mohammadhadi Khorrami, PhD^1^, Pradnya Patil^2^, Kaustav Bera, MBBS^1^, Vamsidhar Velcheti, MD FACP^2^, Anant Madabhushi, PhD^1^

###### ^1^Case Western Reserve University, Cleveland, OH, USA; ^2^Cleveland Clinic, Cleveland, OH, USA

####### **Correspondence:** Prateek Prasanna (pxp238@case.edu)

**Background**

Nivolumab is a FDA approved immune checkpoint inhibitor (ICI) for treatment of patients with chemotherapy refractory advanced NSCLC. The current standard for identifying candidates who would benefit from ICIs is sub- optimal. First, the role of computer-extracted textural descriptors (radiomic features) [1] on baseline CT in predicting response to ICIs, is investigated. Secondly, since the degree of immune response is reflective of a cancer’s ability to respond to ICIs, understanding the distribution of lymphocytic infiltration will help provide a morphological basis for the observed radiographic phenotypes. Towards that end, the predictive radiomic features are correlated with lymphocytic arrangement to understand their morphologic underpinning.

**Methods**

Non-contrast CT scans, before ICI treatment, were retrospectively acquired from 73 NSCLC patients. Patients with an objective response (complete/partial response) per RECIST after two cycles of nivolumab were defined as “responders” and patients with progressive disease were defined as “non-responders”. 454 intra-tumoral texture, 24 shape features and 7426 features from annular rings outside the expert-annotated nodules, capturing different measures of phenotypic heterogeneity, were extracted from the baseline scans. A linear discriminant analysis (LDA) classifier was trained using the most predictive features identified on the discovery set (n=29) and validated on the test set (n=44). Digitized H&E histology scans of baseline biopsies were available for 56 cases. The nuclei were first segmented [2] and then classified into either lymphocytes or non-lymphocytes using texture, shape, and color features. 76 features quantifying density or compactness of tumor infiltrating lymphocyte (TIL) clusters were subsequently extracted. A pairwise Spearman correlation analysis was performed between the TIL compactness measures and the top discriminating radiomic features.

**Results**

A combination of 2 intra-tumoral, 6 peri-tumoral and 1 shape delta radiomic feature yielded an area under the receiver operating characteristic curve (AUC) of 0.85 ± 0.05 within the discovery set and an AUC=0.81 within the validation set. TIL density was found to be statistically significantly (correlation coefficient=-0.5, p<0.05) correlated with a peritumoral Gabor [3] feature, as illustrated in Figure 1.

**Conclusions**

Radiomic features extracted from baseline CT scans were predictive of objective response to ICIs. The TIL compactness features model relationships between lymphocytes and their surrounding cells. Presence of an immune infiltration is more likely to manifest via unique textural patterns in the tumor environment. The radiomic features could therefore be capturing the degree of immune response, which in turn is known to be correlated with the likelihood that the tumor will have a favorable response.

**Acknowledgements**

Research reported in this publication was supported by the National Cancer Institute of the National Institutes of Health under award numbers 1U24CA199374-01, R01CA202752-01A1, R01CA208236-01A1, R01 CA216579-01A1, R01 CA220581-01A1, National Center for Research Resources under award number1 C06 RR12463-01, the DOD Prostate Cancer Idea Development Award; the DOD Lung Cancer Idea Development Award; the DOD Peer Reviewed Cancer Research Program W81XWH-16-1-0329, the Ohio Third Frontier Technology Validation Fund, the Wallace H. Coulter Foundation Program in the Department of Biomedical Engineering and the Clinical and Translational Science Award Program (CTSA) at Case Western Reserve University. The content is solely the responsibility of the authors and does not necessarily represent the official views of the National Institutes of Health.

**References**

1. Prasanna P, Patel J, Partovi S, Madabhushi A, Tiwari P.. Radiomic features from the peritumoral brain parenchyma on treatment-naive multi-parametric MR imaging predict long versus short-term survival in glioblastoma multiforme: preliminary findings. European radiology. 2017; 27(10): 4188-4197.

2. Veta M, Van Diest PJ, Kornegoor R, Huisman A, Viergever MA, Pluim JP.. Automatic nuclei segmentation in H&E stained breast cancer histopathology images. PloS one. 2013 8(7): e70221.

3. Tuceryan M, Jain AK. Texture analysis. In Handbook of pattern recognition and computer vision (pp. 235-276).

**Ethics Approval**

The study protocol was approved under University Hospitals (UH) IRB 02-13-42C

**Fig. 1 (abstract P104).**
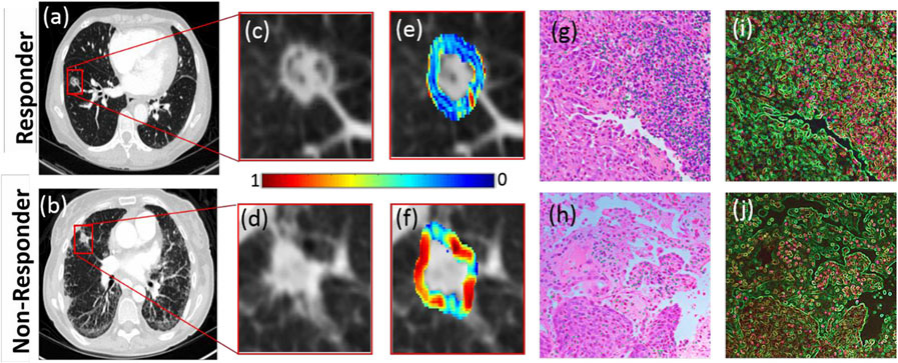
Radiomic expression and TIL density patterns

#### P105 Response to immunotherapy in hepatocellular carcinoma, a single-institutional analysis

##### Petra Prins^1^, Bhavana Singh, MD^2^, AIwu He^2^

###### ^1^Georgeotwn University, Washington, DC, USA; ^2^Medstar Georgetown University Hospital, Washington, DC, USA

####### **Correspondence:** Petra Prins (pap48@georgetown.edu)

**Background**

Despite advances in our understanding of the molecular pathways involved in hepatocellular carcinoma (HCC), therapeutic options remain limited and patient (pt) survival is dismal. Immunotherapy (IO) is one of the newer and more promising options for the treatment of HCC, with nivolumab being at the forefront. In the CheckMate 040 non-randomized, open-label, phase-1/2 study of nivolumab in pts with advanced liver cancer, a median overall survival (OS) of 15 months (m), an overall response rate of 15%, and a median duration of response of 16.6m was observed

**Methods**

In this single-institutional retrospective analysis, 30 pts with HCC received one of five different IO regimens. Thirteen pts had received sorafenib prior to initiation of IO. Patients received either atezolizumab plus bevacizumab, cemiplimab, pembrolizumab, nivolumab, or nivolumab plus ipilimumab until disease progression (PD) or unacceptable toxicity. Responses were assessed using RECIST v 1.1 criteria for stable disease (SD), partial response (PR) and PD, and blood biomarker levels, and were correlated with clinical outcomes like best response and progression free survival (PFS).

**Results**

Demographics for our cohort (n=30) were as follows: 73% were male and 27% female; 33% were African American, 30% Caucasian, 23% Asian, and 13% of other ethnicities. Of our cohort 33% had HBV and 30% had HCV as part of their disease etiology. A positive treatment response was observed in 90% of the pts with 67% having SD and 23% having a PR. Median OS was 20m (95%CI, 7.0-32.0) from the start of IO. A positive response to therapy significantly improved OS (20m SD versus 5m PD, p=0.005). A positive treatment response also showed clear PFS benefits: 20m (95%CI, 0-40.0) for PR, 7m (95%CI, 2.4-11.6) for SD and 3m (95%CI, 0-6.2) for PD (p=0.016). Data for circulating blood biomarkers and image biomarkers are currently being analyzed.

**Conclusions**

Overall, treatment with immunotherapy resulted in durable responses and favorable PFS and OS in pts with advanced HCC within our institution, making immunotherapy a valid option for the treatment of HCC. Additional correlation of IO treatment response with blood biomarkers might improve our understanding of treatment response.

#### P106 Multiplexed ion beam imaging (MIBI) for characterization of the tumor microenvironment (TME) across tumor types

##### Jason Ptacek, PhD, Rachel Finck, Murat Aksoy, Jay Tarolli, Jessica Finn

###### Ionpath, Inc, Menlo Park, CA, USA

####### **Correspondence:** Jessica Finn (jessica.finn@ionpath.com)

**Background**

Cancer arises from tumor cells taking advantage of complex relationships between stromal, vascular, and immune cell subsets. To date the ability to characterize the cellular composition and spatial organization of the TME has been limited by the techniques available to image the necessary number of biomarkers for broad phenotyping at a subcellular resolution. Here we show the applicability of MIBI for cell phenotype identification and their spatial relationships across multiple tumor types.

**Methods**

FFPE samples from tumor biopsies were imaged for their cellular composition and architecture using multiplexed ion beam imaging. Samples were stained with a panel of 15 antibodies, each labeled with a specific metal isotope. The panel was validated by comparing to single-plex IHC and included antibodies for tumor and immune cell subsets in addition to immunotherapy targets (PD-1, PD-L1). In MIBI the stained section is scanned via secondary ion mass spectrometry to image the tissue for expression of the antibody targets. Multi-step processing, including machine-learning-based segmentation, was used to produce images of the TME and determine both the frequency of cell subsets and the distance between immune cells and tumor cells.

**Results**

A total of 25 tumor specimens from 8 tumor types, plus control samples, were characterized for their immune profile, spatial organization of tumor and immune cells and their expression of PD-1 and PD-L1. Tumor-associated macrophages (TAMs) and tumor infiltrating lymphocytes (TILs) were observed in breast, gastric, lung, ovarian, and head and neck cancers. Nearest-neighbor immune:tumor distances revealed the level of mixing between tumor and immune cells (Figure 1). For example, ovarian serous carcinoma samples showed large numbers of infiltrating cytotoxic T cells and macrophages amongst tumor cells. However, the TMEs differed with one showing mixing of the populations and the second showing a compartmentalized organization. In contrast, an ovarian endometrioid carcinoma specimen showed much less robust immune cell infiltrate. Similar results of mixing or compartmentalization were observed for lung and gastric adenocarcinomas. Interestingly, PD-L1 expression was detectable in all gastric adenocarcinomas with the strongest expression observed in the tumor showing the most compartmentalization.

**Conclusions**

The function and phenotypes of cells can only be determined through the co-expression of multiple proteins.

Multiplexed imaging by MIBI reveals the complex tumor immune landscape by enabling the characterization of the spatial relationship of immune and tumor cells and the expression of immunoregulatory proteins. This work demonstrates the possibilities of MIBI for future patient stratification through characterization of the TME.

**Fig. 1 (abstract P106).**
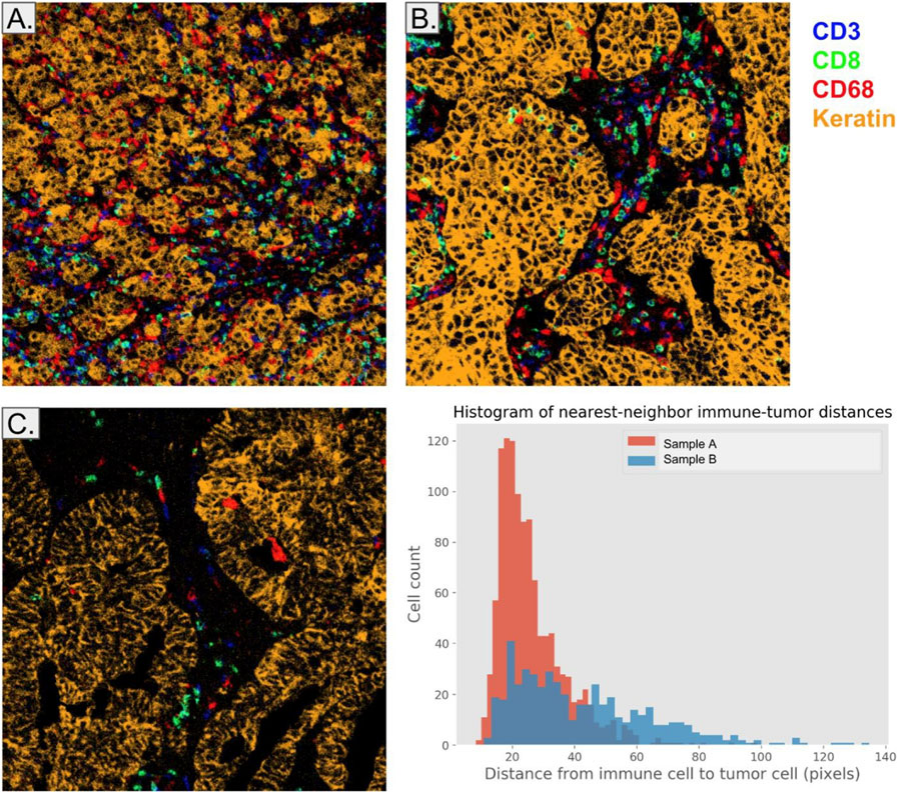
Spatial distribution of cells within tumor samples

#### P107 A comparative study of the PD-L1 IHC 22C3 and 28-8 assays in melanoma samples

##### Gabriel Krigsfeld, PhD^1^, Kim Zerba^1^, James Novotny Jr^1^, Shuntae Williams^2^, Michael Matthews, MS^3^, Hytham Al- Masri, PhD^2^, David Gold^1^, James White^1^

###### ^1^Bristol-Myers Squibb, Princeton, NJ, USA; ^2^Hematogenix Laboratory Services, LLC, Tinley Park, IL, USA; ^3^Acupath Laboratories, Inc, Plainview, NY, USA

####### **Correspondence:** James White (James.White@bms.com)

**Background**

Nivolumab is a programmed death-1 (PD-1) receptor blocking antibody approved as monotherapy or in combination with ipilimumab for patients with unresectable or metastatic melanoma. The Dako programmed death ligand 1 (PD- L1) immunohistochemistry (IHC) 28-8 pharmDx is an FDA-approved PD-L1 complementary diagnostic for melanoma. While the Dako PD-L1 IHC 22C3 pharmDx is an FDA-approved companion diagnostic for several tumor types, it is not approved for melanoma. To date, studies have compared the 2 assays in non-small cell lung cancer and urothelial carcinoma. We report a comparison of the 28-8 and 22C3 assays on real-world melanoma samples, which provide new data to address the potential interchangeable use of these assays in clinical practice.

**Methods**

Formalin-fixed, paraffin-embedded melanoma samples were obtained from Acupath Laboratories, Inc (Plainview, NY) with basic demographic information. Staining and scoring of slides were performed at Hematogenix (Tinley Park, IL). The 28-8 and 22C3 PD-L1 assays were used per manufacturer’s protocols and evaluated using the 28-8 pharmDx tumor cell scoring algorithm [1]. Slides were randomized and pathologists were blinded to the assay at scoring, with paired 28-8 and 22C3 assay results read by the same pathologist. Samples that were heterogeneous (determined by bracket evaluation with H&E staining), had high melanin content, or failed to meet minimum criteria (≥100 tumor cells per 28-8 assay) were excluded from the final analysis.For the primary analysis, overall, positive, and negative percentage agreement (OPA, PPA, and NPA) at the ≥1% PD-L1 expression level were evaluated. Secondary analyses included agreement rates at the ≥5% expression level, Passing–Bablok regression, and Bland– Altman plots with summary statistics describing the differences between assays across the dynamic range.

**Results**

Of 265 samples analyzed, 202 were confirmed to contain melanoma tissue with quantifiable PD-L1 expression.

Average patient age was 66.4 years. Average sample age was <2 years (657 days [range 637–678]), and samples ranged from stage I to IV melanoma. High analytical concordance was observed between the 28-8 and 22C3 assays across paired melanoma samples. OPA, PPA, and NPA for all paired samples at the ≥1% PD-L1 expression level were 93.1%, 82.1%, and 97.3%, respectively. Identical PD-L1 expression values for the 28-8 and 22C3 assays were reported for 82.7% of samples, with a ≤10% difference in tumor cell membrane staining for 98.0% of samples.

**Conclusions**

These data support the potential interchangeability of the PD-L1 IHC 28-8 and 22C3 pharmDx assays for assessing tumor cell membrane PD-L1 expression on melanoma samples.

**Acknowledgements**

Medical writing assistance was provided by Bernard Kerr, PGDipSci, CMPP™, of Spark Medica Inc. (US), funded by Bristol-Myers Squibb. This study was funded by Bristol-Myers Squibb.

**References**

1. Dako. PD-L1 IHC 28-8 pharmDx SK005. Package Insert. 2017. [https://www.agilent.com/cs/library/packageinsert/public/128371004.PDF] Accessed July 30, 2018.

#### P108 HLA class I subtypes are associated with immune-related adverse events in patients with melanoma treated with ipilimumab

##### Anton Safonov, MD^1^, John Pluta^1^, Lu Qian^2^, Ravi Amaravadi^1^, Allison Applegate^3^, Elizabeth Buchbinder, MD^4^, Justine Cohen, DO^4^, Claire Friedman, MD^5^, Ruth Halaban, PhD^6^, F. Stephen Hodi, MD^4^, Christine Horak, PhD^7^, Douglas Johnson, MD, MSCI^8^, John Kirkwood, MD^9^, Tara Mitchell^1^, William Robinson, MD, PhD^3^, Lynn Schuchter, MD^1^, Jeffrey Sosman, MD^10^, Mario Sznol, MD^6^, Megan Wind-Rotolo, PhD^7^, Jedd Wolchok, MD, PhD^5^, Mingyao Li^1^, Peter Kanetsky^2^, Katherine L. Nathanson^1^

###### ^1^Perelman School of Medicine, University of Pennsylvania, Philadelphia, PA, USA; ^2^Moffitt Cancer Center, Tampa, FL, USA; ^3^University of Colorado, Denver, CO, USA; ^4^Dana Farber Cancer Institute, Boston, MA, USA; ^5^Memorial Sloan Kettering Cancer Center, New York, NY, USA; ^6^Yale University, New Haven, CT, USA; ^7^Bristol-Myers Squibb, Lawrenceville, NJ, USA; ^8^Vanderbilt Cancer Center, Nashville, TN, USA; ^9^University of Pittsburgh Medical Center, Pittsburgh, PA, USA; ^10^Feinburg School of Medicine, Northwestern University, Chicago, IL, USA

####### **Correspondence:** Katherine L. Nathanson (knathans@upenn.edu)

**Background**

Checkpoint immunotherapies have demonstrated substantial benefit in patients with melanoma. However, there are currently no established biomarkers to predict immune-related adverse events (irAEs). Because genetic variation at the human leukocyte antigen (HLA) locus is associated with increased risk of autoimmune disorders, we evaluated associations between irAEs and HLA subtypes.

**Methods**

We utilized clinical and genomic data from 269 chemotherapy and immunotherapy-naïve melanoma patients treated with ipilimumab monotherapy on the Bristol-Meyers-Squibb clinical trial CA184-169 (NCT01515189). DNA samples were genotyped on Affymetrix 6.0 and standard sample- and SNP-level quality control pipelines were conducted; only samples with high levels of European genetic ancestry (i.e. Caucasian) were further analyzed. Genotype imputation was completed using the Haplotype Reference Consortium Backbone. We used SNP2HLA to establish estimated allele dosages for each 4-digit HLA allele (n = 172), and subsequently collapsed these calls into one of nine established class I supertypes. We catalogued occurrences of immune-related adverse events by CTCAE grade and affected organ system. Logistic regression was performed to evaluate associations of class I HLA supertype allele dosage with irAE outcomes (grade < 2 vs grade ≥ 2) adjusting for ECOG score, dosage, and number of doses.

**Results**

HLA-A24 was significantly associated with hepatitis (Grade > 2), with meta-analysis odds ratio 3.69 [95% CI 1.75 to 7.51, p = 0.0005]. Odds ratio was 3.54 [95% CI 1.51 to 8.31, p = 0.0036] in the BMS dataset (remaining significant after correction for multiple comparisons), and OR 3.85 [95% CI 0.96 to 15.5, p = 0.056] from the consortium data set. The frequency of HLA-A24 in our population was 9.6%.

**Conclusions**

Our results demonstrate that a specific HLA subtype, HLA-A24, is associated with checkpoint-inhibitor related hepatitis. HLA-A24 has been explored in its dose-dependent response to hepatitis B and C vaccination. Our results potentially demonstrate unmasking of germline-mediated immune sensitivity by checkpoint inhibitors. This data warrants further study into HLA subtypes as predictive biomarkers for immune-related adverse effects. These findings should be confirmed in additional study populations, potentially with sequence-based HLA typing.

**Trial Registration**

NCT00135408, NCT00261365, NCT01515189

**Ethics Approval**

The protocol and all amendments were approved by the institutional review board or independent ethics committee for each study center

#### P109 Clinical efficacy of immune checkpoint inhibitors in patients with small cell lung cancer is associated with high tumor mutational burden and development of immune-related adverse events

##### Biagio Ricciuti, MD^1^, Suzanne Dahlberg^1^, Sasha Kravets^1^, Safiya Subegdjo^1^, Renato Umeton^1^, Adem Albayrak, MS^1^, Lynette Sholl^2^, Mark M. Awad^1^

###### ^1^Dana Farber Cancer Institute, Boston, MA, USA; ^2^Brigham and Women's Hospital, Boston, MA, USA

####### **Correspondence:** Mark M. Awad (Mark_Awad@dfci.harvard.edu)

**Background**

Immune-checkpoint inhibitors (ICIs) have shown promising activity in only a fraction of patients with small cell lung cancer (SCLC), and factors associated with clinical benefit are not well characterized. High tumor mutational burden (TMB), quantified by whole exome sequencing, has been shown to predict response to ICIs in SCLC. However, whether targeted next generation sequencing (NGS) can be used to identify SCLC patients with high TMB who might benefit from treatment with immunotherapy is currently unknown. The relationship between the development of immune-related adverse events (irAEs) and immunotherapy response in SCLC is also unknown.

**Methods**

Patients with SCLC at the Dana-Farber Cancer Institute (DFCI) who received treatment with immunotherapy and/or had successful NGS were included in this study. TMB was determined using the DFCI NGS OncoPanel platform of >450 genes. The relationships between TMB, the development of irAEs, and clinical outcomes were determined among patients with SCLC treated with PD-1 inhibitors alone or in combination with a CTLA-4 inhibitor.

**Results**

Out of a total of 145 patients, 125 (86.2%) had successful NGS with TMB assessment and 64 (44.1%) were treated with ICIs. The median (range) TMB was 9.29 (1.21-33.89) mutations/megabase. Among 44 TMB-evaluable patients treated with ICIs, the median progression-free survival (mPFS) was significantly longer in the 21 patients with a TMB above median (“TMB-high”) compared to the 23 patients below median (“TMB-low”) (4.1 vs. 1.4 months, HR: 0.39 [95%CI: 0.19-0.77], P < 0.01) (Figure 1A). The median overall survival (mOS) was significantly prolonged in the TMB-high group compared to the TMB-low group (10.5 vs. 2.7 months, HR: 0.43 [95%CI: 0.20- 0.89], P = 0.02) (Figure 1B). Among 64 patients with SCLC treated with ICIs (44 TMB-evaluable), the 21 (32.8%) patients who developed at least one irAE had significantly longer mPFS (4.1 vs. 1.3 months, HR: 0.33 [95%CI: 0.19-0.56], P < 0.001) and mOS (12.5 vs. 3.2 months, HR: 0.35 [95%CI: 0.20-0.62], P < 0.01) compared to the 43 (67.2%) patients who did not develop irAEs (Figure 2 A,B). Patients with irAEs had a significantly higher mean TMB than those who did not experience any irAEs (13.09 vs. 9.41, P = 0.02).

**Conclusions**

Median PFS and OS on immunotherapy were significantly longer among patients with SCLC and high TMB and among those who developed at least one irAE. TMB determination by NGS may be a helpful biomarker for identifying patients who are likely to benefit from treatment with ICIs in SCLC.

**Fig. 1 (abstract P109).**
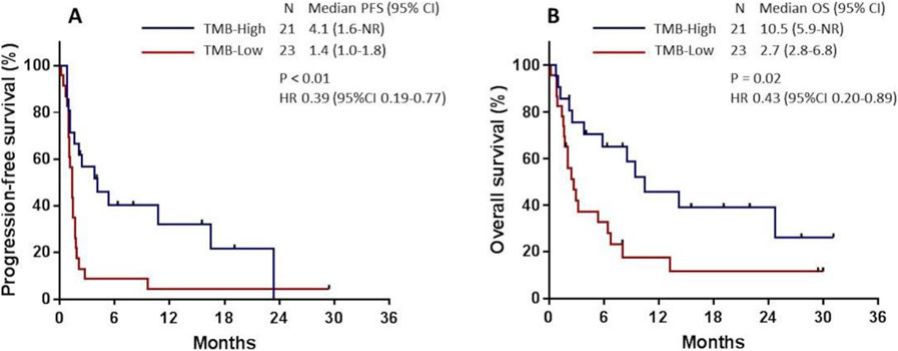
See text for description.

**Fig. 2 (abstract P109).**
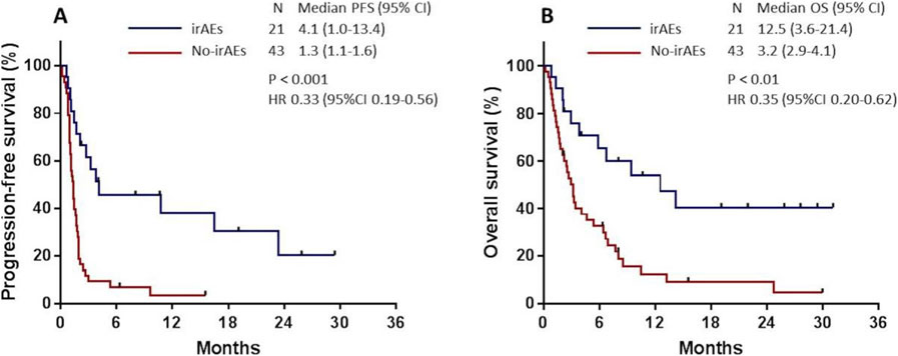
See text for description.

#### P110 Modular repertoire analysis of blood transcriptomic immune response in metastatic renal cell carcinoma patients treated with Pazopanib

##### Darawan Rinchai, PhD^1^, Elena Verzoni^2^, Agata Cova^2^, Paola Squarcina^2^, Loris Cecco^2^, Paolo Grassi^2^, Raffaele Ratta^2^, Veronica Huber^2^, Matteo Dugo^2^, Monica Rodolfo^2^, Jessica Roelands, Master^1^, Damien Chaussabel^1^, Giuseppe Procopio^2^. Davide Bedognetti, MD, PhD^1^, Licia Rivoltini, MD^2^

###### ^1^Sidra Medicine, Doha, Qatar; ^2^Fondazione IRCCS Istituto Nazionale Tumori, Milan, Italy

####### **Correspondence:** Davide Bedognetti (dbedognetti@sidra.org)

**Background**

Transcriptional modular repertoire analysis has been developed and successfully used as a basis for the selection of biomarkers and development of multivariate transcriptional indicator of disease progression in patients with systemic lupus erythematosus or infectious diseases. In the context of cancer immunotherapy, understanding the molecular mechanisms modulated by a given drug is critical to implement more efficient therapeutic approaches. Here, we employed a modular repertoire approach to investigate immune response in metastatic renal cell carcinoma patient treated with Pazopanib (Tyrosine kinase inhibitors).

**Methods**

Peripheral blood mononuclear cells collected from 8 metastatic renal cell carcinoma patients (mRCC) receiving first-line Pazopanib were prospectively analyzed at baseline, 3 and 6 months for whole-genome transcriptomic perturbations using Illumina HT12v4BeadChip. As for the best response, partial response was observed in 3 patients and stable disease in 5 patients. A set of 260 transcriptional modules was used for the analysis of this dataset using a pre-defined framework (1–3). A module is considered to be “responsive” to the treatment when significant changes in abundance are observed for a proportion of its constitutive transcripts that is greater that what could be expected by chance.

**Results**

We first assessed changes in transcript abundance at the modular level. The percentage of responsive transcripts constitutive of a given module was determined for each time point (group comparison) or individual comparison.

The group comparison analysis showed that module perturbations peaked at 3 months and decreased at 6 months. These perturbations include enrichment of modules M3.6 & M8.46 (Cytotoxic/NK), M4.11(Plasma cells), and M8.89 (Immune response) (Figure 1). In addition, individual-level analysis showed that Pazopanib administration was associated with the decreased of the immunosuppressive module M9.34 in 5 patients. Such module was up- regulated only in one patient who did not respond to treatment. Interestingly, a rapid increased of Interferon (IFN) modules (M1.2 & M3.4) was observed exclusively in responding patients (Figure 2).

**Conclusions**

These results suggest that Pazopanib has a strong immune modulatory effect and might reshape anti-tumor immunity by reducing immunosuppression and triggering cytotoxic mechanisms and IFN pathways. The peak of this immune modulation is observed 3 months after treatment. These data provide a strong rationale for exploring combinatorial immune-targeted therapy based on kinase inhibitors such as a Pazopanib and for implementing transcriptomic analysis of peripheral blood in the context of cancer immunotherapy.

**References**

1. Chaussabel D, Quinn C, Shen J, Patel P, Glaser C, Baldwin N, et al. A modular analysis framework for blood genomics studies: application to systemic lupus erythematosus. Immunity. 2008 Jul 18;29(1):150–64.

2. Obermoser G, Presnell S, Domico K, Xu H, Wang Y, Anguiano E, et al. Systems Scale Interactive Exploration Reveals Quantitative and Qualitative Differences in Response to Influenza and Pneumococcal Vaccines. Immunity. 2013 Apr 18;38(4):831–44.

3. Chaussabel D, Baldwin N. Democratizing Systems Immunology with Modular Transcriptional Repertoires Analyses. Nat Rev Immunol. 2014 Apr;14(4):271–80.

**Fig. 1 (abstract P110).**
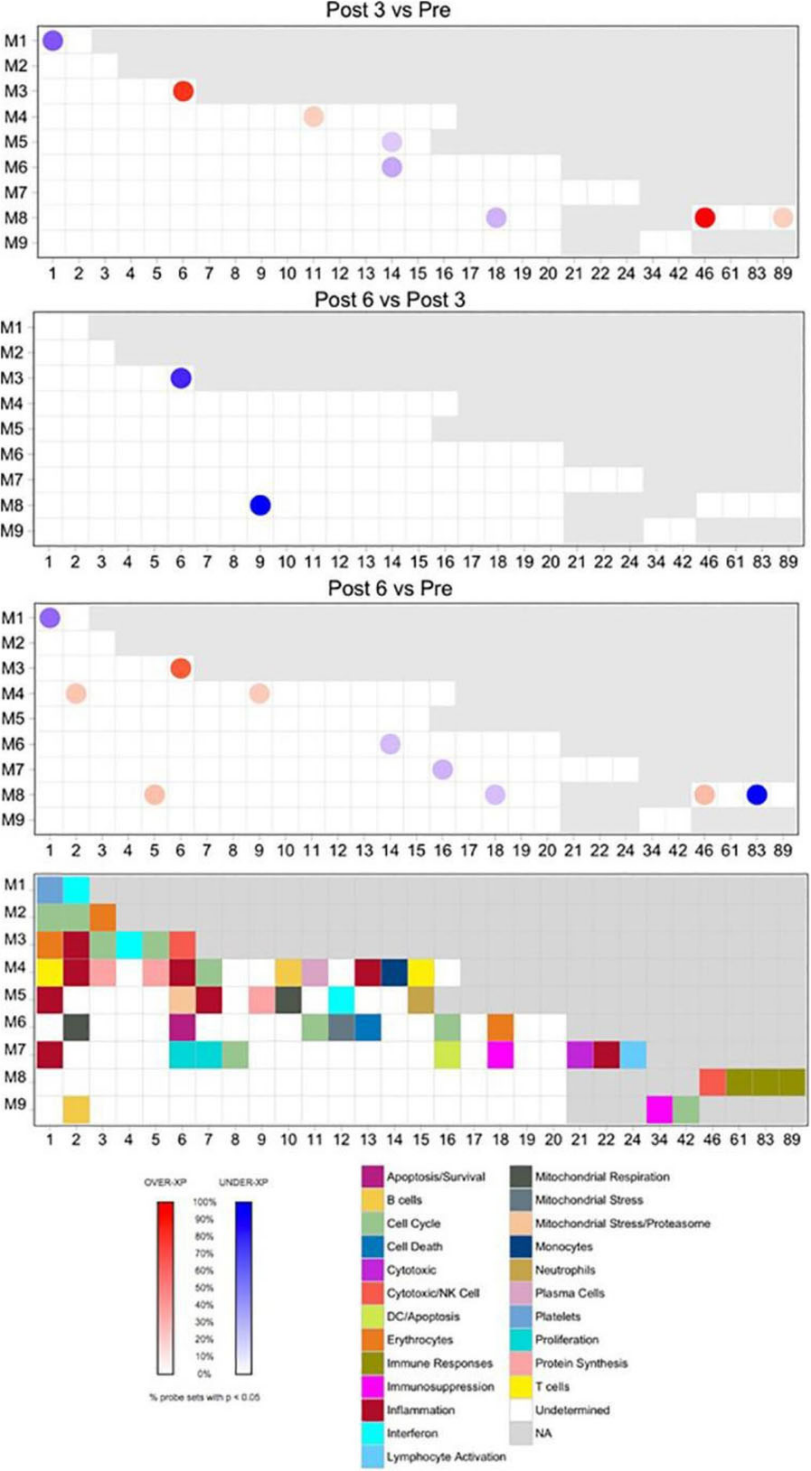
See text for description.

**Fig. 2 (abstract P110).**
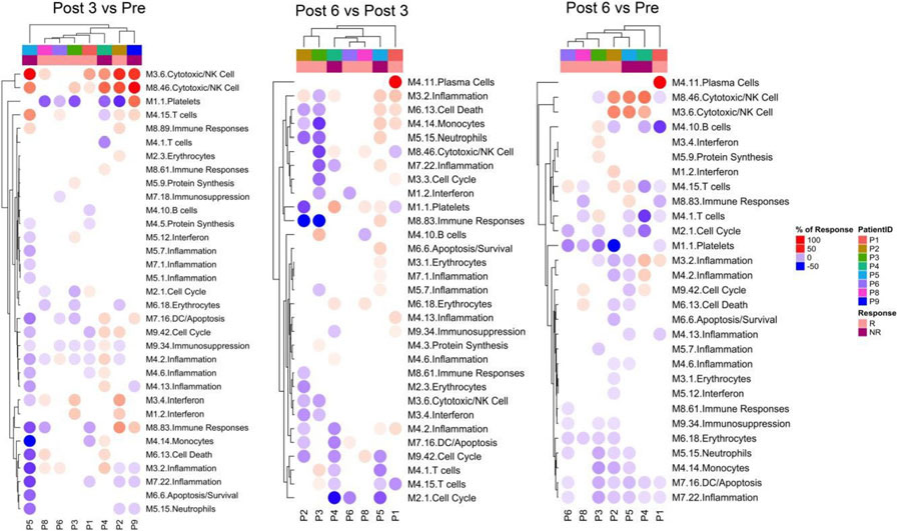
See text for description.

#### P111 Streamlined human immune monitoring with mass cytometry: 29 markers in a single tube with automated data analysis

##### Christina Loh, PhD^1^, Thiru Selvanantham, PhD^1^, Leslie Fung^1^, Michelle Poulin^1^, C. Bruce Bagwell^2^, Margaret Inokuma^2^, Clare Rogers, MS^1^, Greg Stelzer, PhD^1^, Steven Lott, PhD^1^

###### ^1^Fluidigm, South San Francisco, CA, USA; ^2^Verity Software House, Topsham, ME, USA

####### **Correspondence:** Clare Rogers (clare.rogers@fluidigm.com)

**Background**

Immune monitoring is an essential method for quantifying changes in immune cell populations in health and disease.

The extreme heterogeneity of immune cells demands a high-parameter approach to more fully and efficiently quantify these changes. Mass cytometry is an ideal solution, enabling simultaneous detection of over 40 phenotypic and functional markers in a single tube of sample.

**Methods**

We developed a single-tube, 29-marker panel for mass cytometry based on the Human ImmunoPhenotyping Consortium (HIPC) consensus panel [1], expanded to allow identification of additional leukocyte subsets, particularly T cells. Automated data analysis with Verity Software House GemStone™ has been developed specifically for data collected with the panel, providing users with results in minutes, reducing time-to-answer and variability inherent in manual gating.

**Results**

Repeatability was tested with a single PBMC sample stained by a single technician in two technical replicates and acquired in triplicate on two Helios™ mass cytometers. SDs for percent of parent were 1% or less for 16 identified populations. Reproducibility was tested by determining the variability in measurements of five PBMC lots stained by five technicians and collected on two Helios instruments. CVs on mean percent of all measured populations were under 20%. R2 values for agreement of percent parent populations using the full 29 marker panel compared to smaller 10-to-12-marker panels for T, B and myeloid cell populations were 0.94 or higher. In addition, comparison of cell population frequencies determined using GemStone analysis with manual gating in WinList demonstrated a high degree of concordance.

**Conclusions**

We conclude that this panel kit can provide consistent immune population identification and enumeration for any given lot of PBMC, and that data generated by the kit is amenable to either manual or automated data analysis.

**References**

1. Maecker HT, McCoy JP, Nussenblatt R, Standardizing immunophenotyping for the Human Immunology Project. Nature Reviews Immunology 2012; 12: 191-200.

#### P112 Proteomic profiling of biomarkers for response to checkpoint immunotherapy in melanoma patients

##### Marijana Rucevic, PhD (m.rucevic@olink.com)

###### Olink Proteomics, Watertown, MA, USA

**Background**

Checkpoint immunotherapy has greatly improved clinical outcomes in patients with several malignancies including melanoma. However, only a subset of advanced melanoma patients generates durable responses to such immunotherapy. To date, there are no reliable methods to predict responders which urges a need to develop serological biomarkers of melanoma response to immune checkpoint therapy.

**Methods**

We applied an innovative and highly sensitive Proximity Extension Assay (PEA) for comprehensive profiling of ~1000 proteins in the plasma of 55 metastatic melanoma patients that received anti-PD-1 treatment whereas, 18 patients were previously treated with anti-CTLA-4 while 37 received anti-PD-1 monotherapy. Of the 55 patients, 42 were classified as having benefit from immunotherapy (‘responders’) and 13 had no benefit (‘non-responders’). Our study included analyses of samples collected at baseline, on-treatment (~67 days from baseline) and post-treatment from these patients.

**Results**

Proteome wide analysis identified changes in ~100 immune response proteins over the course of treatment with different characteristic behavior across the treatment period. The dynamic response proteins included current and new exploratory checkpoint targets (PD-1, PDL-1, LAG-3, Gal-9, CD27, TNFR2) as well as, many proteins associated with the events that are hallmarks of immune response to cancer, such as cytokines/chemokines, angiogenesis, vascular cell adhesion and oncogenic signaling proteins. Importantly, 7 proteins including HER3, SAA4, NID1, ST6GAL1, OPN, TIMP1 and CXCL13 were found distinctive between responders and non- responders at the first on-treatment time point and demonstrated potential to classify responders and non-responders Thus, they may represent a protein signature that can predict treatment response or serve as surrogate markers for efficacy to immune checkpoint therapy in melanoma.

**Conclusions**

Our study might provide better understanding of immune system interaction with checkpoint inhibitors and demonstrates potential to improve melanoma patient’s management that may have a significant impact to the field of immunotherapy.

#### P113 Digital spatial profiling of bone-marrow infiltrating immune cells in acute myeloid leukemia

##### Sergio Rutella, MD, PhD, FRCPath^1^, Jayakumar Vadakekolathu, PhD^1^, Sarah Church^2^, Heidi Altmann^3^, Elena Viboch, MS^2^, Jorn Meinel, MD^3^, Yan Liang, MD PhD^2^, Joseph Beechem, PhD^2^, Alessandra Cesano, MD, PhD^2^, Sarah Warren, PhD^2^, Gerhard Enhinger, MD^4^, Marc Schmitz, MD^4^, Martin Bornhauser, MD^3^

###### ^1^Nottingham Trent University, Nottingham, UK; ^2^NanoString Technologies, Inc., Seattle, WA, USA; ^3^University Hospital Carl Gustav Carus, Dresden, Germany; ^4^Technische Universität Dresden, Dresden, Germany; ^5^The John van Geest Cancer Research Centre, Nottingham, UK

####### **Correspondence:** Sergio Rutella (sergio.rutella@ntu.ac.uk)

**Background**

The therapeutic approach in patients with acute myeloid leukemia (AML) has not changed substantially in >30 years. The discovery of new treatment strategies, including immunotherapy, remains a priority [1-3]. Herein, we employed Digital Spatial Profiling (NanoString Technologies, Seattle) to characterize the expression of 31 immuno- oncology (IO) proteins in 10 bone marrow (BM) samples from adult patients with newly diagnosed AML.

**Methods**

FFPE BM slides were incubated with fluorescent markers (CD3 to identify T cells, CD123 to identify AML blasts, and Syto83 to stain nuclei) to establish the overall tissue morphology, followed by a cocktail of antibodies conjugated with oligo tags via a photo-cleavable linker. We then identified regions of interest (ROIs: CD3-rich and CD3-poor) with visible light-based imaging and selected them for high-resolution multiplex profiling. Oligo tags from the selected ROIs released upon exposure to UV light, were collected via a micro-capillary tube, hybridized to 6-spot optical barcodes, and digitally counted using the NanoString nCounter® platform. In parallel, we measured the expression of 770 immune-related mRNAs (including 14 molecules covered by the DSP IO panel) using the Pan-Cancer Immune Profiling Panel™ (NanoString Technologies, Seattle).

**Results**

We selected 24 geometric ROIs per BM sample using fluorescent anti-CD3 and anti-CD123 antibodies. Overall, T cell abundance across the 10 BM biopsies was highly variable and median CD3 barcode counts were used to classify samples into CD3-rich and CD3-poor. In the context of the six CD3-rich biopsies (Fig. 1A), ROIs were assigned to either CD3low, CD3int and CD3high categories using the 25th and 75th percentile of the area-normalized CD3 barcode counts. The expression levels of PD1, B7-H3, CD45RO, FoxP3, CD4 and CD8 were significantly higher in CD3high compared with CD3int and CD3low ROIs. The density of CD8+ T cells with an activated/exhausted CD45RO+PD1+ phenotype correlated with PD-L1 expression, consistent with the establishment of adaptive immune resistance (Fig. 1B). A similarity matrix of IO proteins allowed us to identify co-expression patterns of monocyte/macrophage markers (CD14/CD68) and negative immune checkpoints (B7-H3 [CD276] and VISTA), as well as a correlation between PD-L1, Bcl-2 and PTEN within the selected ROIs (Fig. 1C). Finally, in situ expression of mRNA and barcode counts for CD19, CD14, CD68, CD56 and Bcl-2 were also significantly correlated.

**Conclusions**

This proof-of-concept study provides evidence for heterogeneous immune profiles and advances our understanding of the immuno-biology of AML. DSP could support the implementation of future immunotherapy clinical trials.

**Acknowledgements**

Grant support: Qatar National Research Fund (#NPRP8-2297-3-494), Roger Counter Foundation (Dorset, UK) and John and Lucille van Geest Foundation to S.R.

**Ethics Approval**

Bio-banked tissue biopsies were collected at Technische Universität Dresden, Germany, under approval by the SAL AML Biobank's Ethics Committee.

**Fig. 1 (abstract P113).**
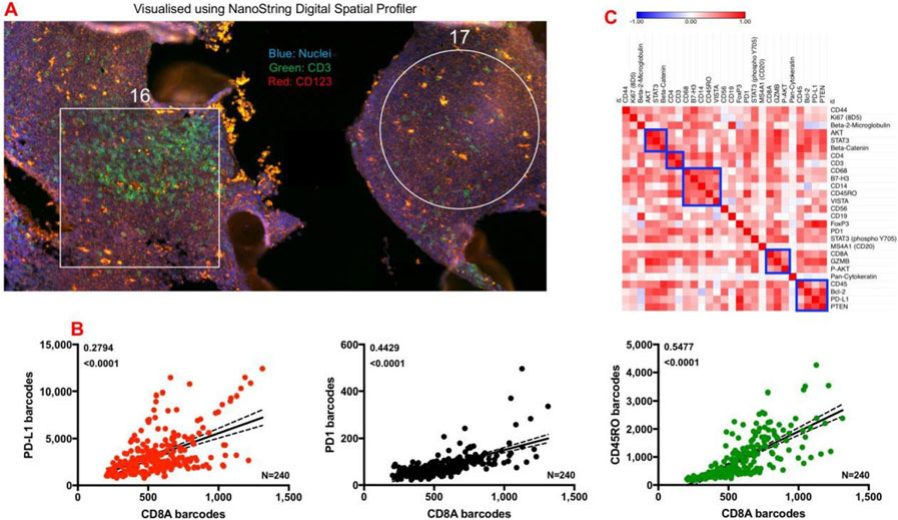
Digital spatial profiling of AML

#### P114 ImmunoINTEL, a flow cytometry based platform that identifies and quantifies the most critical cell subsets and related functional potential in dissociated solid tumors

##### Konstantin Salojin, PhD^1^, Christine Hauther^1^, Dai Liu^1^, Santosh Putta^2^, Norman Purvis, PhD^1^, Matt Westfall, PhD^1^

###### ^1^Pierian Biosciences, Franklin, TN, USA; ^2^Qognit,Inc., Franklin, TN, USA

####### **Correspondence:** Konstantin Salojin (csalojin@pierianbio.com)

**Background**

Standard immunohistochemistry (IHC) approaches to tumor-infiltrating leukocyte (TIL) phenotyping yield limited information, as they utilize antibody (Ab) panels not broad enough to identify and functionally characterize the complexity of TIL subsets. A flow cytometry platform was developed that delivers quantitative, clinically relevant information to support the following: 1) Phenotypic and functional analysis of TILs to characterize the immune status within the tumor microenvironment (TME), and 2) Quantification of TIL and tumor cell (co-)expression profiles of targetable immunomodulatory receptors (IMR) and ligands (IMR-L).

**Methods**

Standardized flow cytometry set-up, QC and sample processing procedure were established. Automated instrumentation and sample processing procedures were implemented in a barcode-based workflow utilizing Hamilton STARlet automated liquid handlers fully integrated with an SMS/LIMS and multi-dimensional flow cytometry data analysis software. Six Ab panels, ranging from 6-12 colors were designed, optimized, and validated to profile PBMC and TIL subsets.

**Results**

Phenotypic composition of the following cell subsets in the TME was delineated and quantified using qualified antibody panels for: (1) T, B, and NK cells; (2) T regulatory cells; (3) myeloid cell subsets (classical/nonclassical monocytes, M1/M2-macrophages); (4) myeloid cells with suppressive phenotypes (M- and G-MDSC); (5) myeloid/plasmacytoid DCs; (6) epithelial and mesenchymal cells. The relative quantities of the TIL subsets were tabulated based on tumor type. The ImmunoINTEL panels were complemented with drop-in IMR and IMR-L markers (PD1/PDL1, TIGIT/CD112-CD155, TIM-3/Galectin-9, SIRPa/CD47, LAG-3/HLADR) to profile the functional status of TILs, and to study the cytotoxic potential of tumor TILs. Reliable separation of IMR/IMR-L positive TILs from negative TILs and IMR/IMR-L positive tumor cells from negative tumor cells was observed, with notable heterogeneity, across cell and tumor types. This included the elevated expression of several IMR-Ls on TILs and IMRs on epithelial and stromal cells, suggesting tumor- and TIL-intrinsic mechanisms modulating checkpoint interactions. Results demonstrate a highly reproducible data set with minimal variability in PBMC reference/control specimens (CVaverage < 5%; n = 30) and accurate quantitation of lymphoid, myeloid, epithelial, and mesenchymal cell subsets in tumor samples.

**Conclusions**

Stringent flow cytometry QC processes were developed and implemented to ensure quality and precision of phenotypic and functional analyses of dissociated TILs and tumor cells, capturing the most critical metrics of intra- tumor immune responses and providing quantitative characterization of IMR and/or IMR-L interactions for more refined selection of potential responders to immune checkpoint inhibitors.

**Ethics Approval**

This study was reviewed and approved by BioIVT’s and iSpecimen’s Ethics/Regulatory Committees, approval numbers: 0944, 0984, 0985, 01034, 01045, 01050, 01071, 01084, 01095, & 01096. All of the samples collected under the above listed approvals are in full compliance with applicable Good Clinical Practices as defined by U.S. Food and Drug Administration (FDA) and U.S. Department of Health and Human Services (HHS) regulations as well as the International Conference on Harmonization (ICH) guidelines.

#### P115 Predictive and pharmacodynamic biomarkers associated with treatment with the oral selective AXL Inhibitor bemcentinib in combination with pembrolizumab in patients with advanced NSCLC and Melanoma

##### Robert Holt, PhD^1^, David Micklem, PhD^1^, Anthony Brown^1^, Cornelia Schuster^2^, Oddbjørn Straume^2^, James Lorens, PhD^1^

###### ^1^BerGenBio ASA, Bergen, Norway; ^2^Haukeland University Hospital, Bergen, Norway

####### **Correspondence:** James Lorens (james.lorens@uib.no)

**Background**

Bemcentinib (BGB324) is a first-in-class, oral, potent and highly selective inhibitor of the AXL tyrosine kinase currently in phase II clinical development across several cancer types. AXL over-expression has been observed in patients failing PD-1 therapy in several cancers whereas AXL inhibition via bemcentinib has shown synergistic effect with checkpoint blockade in pre-clinical models. Selective blockade of Axl by bemcentinib in combination with pembrolizumab in NSCLC and melanoma is currently being explored in two Phase 2 trials BGBC008 (NCT03184571) and BGBIL006 (NCT02872259). Here we report results of the biomarker research programmes designed to identify predictive and pharmacodynamic biomarkers associated with bemcentinib/pembrolizumab treatment.

**Methods**

Fresh pre-treatment tumour biopsies were mandatory for PD-L1 and AXL analysis by IHC. Plasma protein biomarker levels were measured using the DiscoveryMap v3.3 panel (Myriad RBM) at pre-dose and C2D1 to identify predictive and pharmacodynamic biomarkers associated with bemcentinib/pembrolizumab treatment. The co-localisation of AXL and PD-L1 in tumour infiltrating immune cells was determined using NeoGenomics MultiOmyx.

**Results**

One cycle of treatment with bemcentinib significantly altered soluble Axl protein levels in a subset of patients including those who had benefited from treatment. This observation was consistent across multiple disease indications and treatment regimes. Protein biomarkers predictive of patient benefit following bemcentinib treatment have been identified - correlations with AXL and PD-L1 IHC will also be presented. AXL is expressed in a subset of tumour infiltrating immune cells, primarily macrophages. Inaddition, AXL and PD-L1 were found to be co- expressed.

**Conclusions**

Predictive biomarker candidates were identified supporting potential companion diagnostics development for bemcentinib/pembrolizumab treatment. Pharmacodynamic biomarkers indicate that bemcentinib is selective and on target. AXL is expressed on macrophages and is co-expressed with PD-L1.

**Trial Registration**

NCT03184571 and NCT02872259

**Ethics Approval**

All relevant ethical and regulatory approvals were obtained.

#### P116 Quantitative measurement of CD8, CD68 and PD-L1 expression in a novel multiplex assay and associations of overall survival in non-small cell lung cancer (NSCLC) patients treated with anti-PD-1 Therapy

##### Fahad Shabbir, MD^1^, Jon Zugazagoitia, MD^1^, Yuting Liu, PhD candidate^1^, Katir Patel, PhD^2^, Brian Henick, MD^3^, Scott Gettinger, MD^1^, Roy Herbst, MD, PhD^1^, Kurt Schalper, MD, PhD^1^, David Rimm, MD, PhD^1^

###### ^1^Yale School of Medicine, New Haven, CT, USA; ^2^Ultivue Inc, Cambridge, MA, USA; ^3^Columbia University Irving MedicalCenter, New York, NY, USA

####### **Correspondence:** Fahad Shabbir (fahad.ahmed@yale.edu)

**Background**

While PD-1 axis therapies have dramatically changed outcomes in some lung cancer patients, many patients don’t benefit from these immunotherapies. Quantitative immunofluorescence (QIF) may provide a method for selection of those that benefit. This approach has been limited by the fact that traditional fluorescent multiplexing using tyramide amplification and unique species antibodies result in complexity that would be challenging in the CLIA lab setting. Here we test a novel, single mix, multiplex approach to simultaneously assess CD8, CD68 and PD-L1 in immmuno- therapy treated NSCLC patients

**Methods**

A tissue microarray with 81 spots in two-fold redundancy was derived from Yale patients treated with immunotherapy between 2011and 2017. Both a traditional tyramide and species unique multiplex (DAPI, CD68, PDL1 and Cytokeratin) and a DNA-based kit (UltiMapper Kit 1: CD8, CD68, PD-L1 & Cytokeratin/SOX10 with a nuclear dye) were assessed using the PM2000 microscope and AQUA software. The UltiMapper antibody premix is a single step, antibody mix which decreases overall staining time in anticipation of CLIA lab usage. We validate this new assay by regression with conventional QIF and overall survival (OS).

**Results**

Reproducibility was measured using two slides from 2 different blocks on two different days and showed that they were significantly correlated for CD8 (R2 = 0.58), CD68 (R2 = 0.67) and PD-L1 (R2 = 0.71). Comparative regressions between the two different protocols for the expression of PD-L1 in both tumor and stromal compartments were excellent (R2 = 0.93, and 0.83). From a total of 81 patients, we excluded those whose samples were taken after therapy and those who received more than one immunotherapy resulting in 62 patients for outcome analysis using only the data from the Ultimapper assay. Assessment by OS showed significant relationships for high PD-L1 in tumor (21m vs 8m median OS, p=0.036) and high PD-L1 in CD68 positive macrophages (20m vs 10m median OS, p=0.012). Also, on the same slide, high CD8 was associated with better OS (22m vs. 11m median OS, p=0.011).

**Conclusions**

Expression levels of PD-L1 in tumor, but also in macrophages and the presence of CD8 in tumor show OS benefit in patients treated with immune checkpoint blockade in NSCLC. Future studies are required to evaluate this approach within a CLIA certified laboratory setting.

**Acknowledgements**

Sponsored research agreement between Yale and Ultivue (D. Rimm - PI)

**Ethics Approval**

Yale Human Investigation Committee protocol #9505008219.

#### P117 Host immune response in undifferentiated pleomorphic sarcoma – 10-year retrospective analysis

##### Joseph Sheridan, Andrew Horvai, Ross Okimoto, Rosanna Wustrack

###### University of California, San Francisco, San Francisco, CA, USA

####### **Correspondence:** Rosanna Wustrack (rosanna.wustrack@ucsf.edu)

**Background**

Undifferentiated pleomorphic sarcoma (UPS) is an aggressive soft-tissue sarcoma (STS) characterized by high rates of local and metastatic recurrence. Due to the paucity of therapeutic options, advanced disease remains lethal in a large majority of patients. An improved understanding of how the tumor microenvironment modulates UPS progression may enhance our ability to predict therapeutic responses and improve outcomes.

**Methods**

Thirty-six clinically annotated UPS patients collected over 10 years at a single institution with minimum five-year follow-up and available tumor specimens were included in this retrospective study. Using primary tumor specimens, we performed a targeted immunohistochemical analysis of the UPS microenvironment. We quantified expression of lymphocyte markers (CD8, CD20, CD68) and immune checkpoint protein (PD-L1) in all 36 UPS tumors using automated image analysis. The median percentage of positive cells for each subpopulation was used to define high expression vs. low expression. The Kaplan-Meier method was used to analyze OS and DFS; the association of specific TILs with OS and DFS was analyzed using the Log Rank Test.

**Results**

Factors that correlated with improved overall survival in our UPS cohort included localized disease (p=0.015), and use of intraoperative radiation therapy (IORT) or adjuvant radiation therapy (p=0.01). Our immunohistochemical analysis revealed the presence of TILs (CD8, CD20, CD68) and expression of immune checkpoint protein (PD-L1) in UPS tumors. Patients with a greater population of CD8+ TILs had a 5-year OS of 66% compared to those with lower levels of 28% (p=0.003, Figure 1). CD8+ T-cell expression in UPS tumors inversely correlated with local recurrence (p=0.04), suggesting CD8+ T-cell mediated immune surveillance. Interestingly, we also observed an increase in metastatic events in patients whose tumors harbored low CD8 expression compared to high CD8

expression (59% vs. 41%).

**Conclusions**

Through our quantitative immunohistochemical (IHC) analysis of immune cell subsets in UPS tumors, we identified improved survival in patients with increased infiltration of CD8+ T-Cells. Our study demonstrates that patients with low levels of CD8+ TILs are at increased risk of local (and potentially metastatic) recurrence. These findings underscore the importance of immune mediated tumor surveillance in UPS. Recent advancements in systemic immunotherapy further highlight the immunogenicity of UPS tumors and demonstrate the clinical impact of targeting the tumor microenvironment to improve outcomes for UPS patients.

**Ethics Approval**

Approval from the Institutional Review Board was obtained before beginning this study.

**Fig. 1 (abstract P117).**
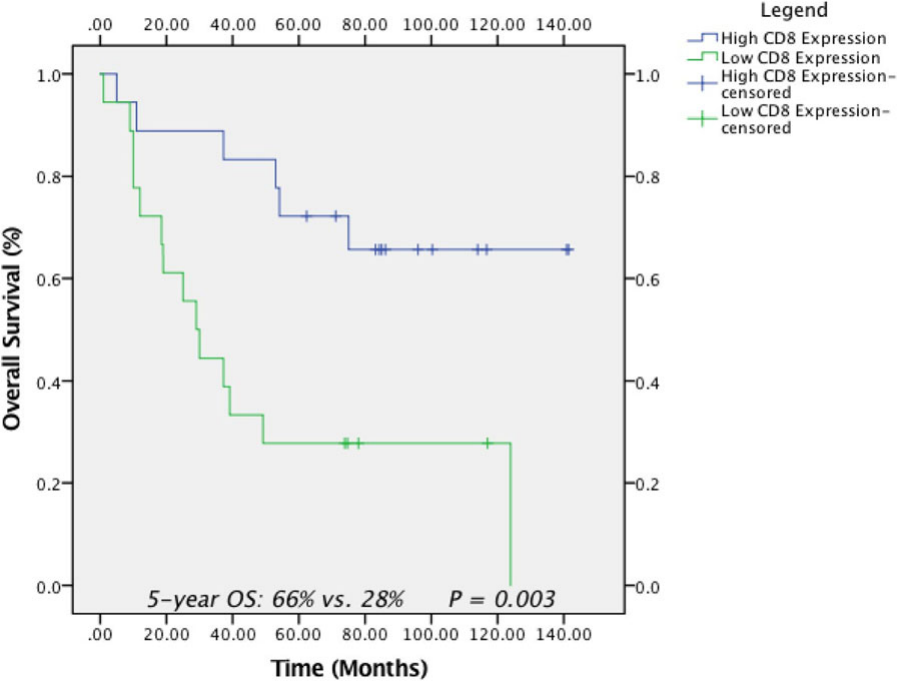
See text for description.

#### P118 Immunosuppression involving MDSC and IL-17 is associated with decreased levels of serum rapid turnover protein and shorter survival in patients with gastrointestinal cancer

##### Masahiko Shibata, MD, Kenji Gonda, MD, PhD, Takahiro Nakajima, Tatsuo Shimura, Koji Kono, Seiichi Takenoshita

###### Fukushima Medical University, Fukushima, Japan

####### **Correspondence:** Koji Kono (kojikono@fmu.ac.jp)

**Background**

Although a causal relationship of inflammation and immune function of cancer patients is more widely accepted today, the precise cell mechanisms mediating this relationship have not been elucidated. Accumulating evidence suggests that myeloid-derived suppressor cells (MDSC) may contribute to the negative regulation of immune responses during cancer and inflammation. IL-17 is a pro-inflammatory cytokine that is primarily secreted by T helper (Th)17 cells and has been reported to be associated with immunosuppressive conditions in patients with cancer. We have reported that systemic inflammation is closely associated with immunosuppression, malnutrition, and poor prognosis in several types of cancer. Cancer cachexia is a multifactorial condition characterized by hypoproteinemia, and systemic inflammation is a major cause of cachexia.

**Methods**

Rapid turnover proteins such as prealbumin (PA), retinol binding protein (RBP) and transferrin (TF), were measured and analyzed in correlation with prognosis in 288 patients with esophageal cancer (Study 1). In order to characterize the inflammation, the production of IL-17 was measured and MDSC (CD11b+CD14-CD33+) in peripheral blood were detected by flow cytometry in 106 patients including 43 with gastric and 63 with colorectal cancer. Blastogenic response of lymphocytes with PHA (SI: stimulation index) was used as a marker of cell-mediated immunity (Study 2).

**Results**

(Study 1) The patients were divided according to their average levels of PA, RBP and TF. The survival of the patients with higher PA, RBP or TF levels were significantly longer than of those with lower PA, RBP or TF levels. The levels of PA, RBP and TF were significantly inversely correlated with SI. (Study 2) The IL-17 production and MDSC levels were both increased along with disease advancement, and significantly correlated with each other. Further, they both were correlated with neutrophil/lymphocyte ratio (NLR), and CRP, inflammatory markers, and significantly inversely correlated with the levels of PA, RBP, and TF. The overall survival of the patients with higher IL-17 production or higher MDSC was shorter than of those with lower IL-17 production or lower MDSC.

**Conclusions**

IL-17-mediated inflammation may associate with immunosuppression involving MDSC, malnutrition and shorter survival and IL-17-targeting therapy may be effective for cancer treatment.

#### P119 Fab-selective proteolysis coupled with liquid chromatography-mass spectrometry for monitoring therapeutic antibodies in circulation

##### Takashi Shimada, PhD, Noriko Iwamoto, PhD

###### Shimadzu Scientific Instruments, Bothell, WA, USA

####### **Correspondence:** Takashi Shimada (tashimada@shimadzu.com)

**Background**

Individual cancer treatment according to drug efficacy indicators is very important matter. The genetic background, drug surrogate biomarker, and immune cell monitoring will be potentially incorporated into the future medicine as well as the expression level of target molecule or pharmacokinetic information. For the analysis of low-MW drugs, liquid chromatography-mass spectrometry (LC-MS) are often used. This is optimal approach for the structure identification and quantitation with sequential and comprehensive manner. However, for the high-MW biopharmaceuticals, pharmacokinetic parameters are often analyzed by ligand binding assays (LBA). The LBA may have some significant limitation. Therefore, analytical innovation should be indispensable independent of a variety of antibodies. For LC-MS, it should be developed the integrated and overall optimized approaches from complex biological samples because of the issue in separation and ionization suppression. We have focused on the two features: antibody structure-indicated analysis, and complementarity-determining region (CDR)-targeting quantitation. The ideal antibody analysis is possible using the selective quantitation of somatic mutated region CDRs.

**Methods**

IgGs were immobilized in resin pore (100 nm) via Fc, so that Fab was oriented to reaction solution. And proteolysis was performed by immobilized trypsin on the surface of FG nanoparticles (200 nm). Owing to these diameter difference, limited proteolysis on Fab was successful with maintaining the antibody specificity while minimizing the complexity or protease contamination. We named this method nano-surface and molecular-orientation limited (nSMOL) proteolysis.[1] And the generated signature peptides from each antibody were quantified using triple quadrupole LC-MS/multiple reaction monitoring (MRM) analysis.

**Results**

We have performed the assay validation development for Trastuzumab, Bevacizumab, Nivolumab, and more than 25 items according to the guideline on bioanalysis method validation.[2] The quantitation range in human plasma was obtained from about 0.2 to 300 μg/ml, which is enough to cover the clinical levels. And the verification of clinical samples has also been successfully with high-reproducibility.

**Conclusions**

The feature of nSMOL is the breakthrough solution for the accuracy, reproducibility, cost, general-purpose, QC and stability. We have some study design for the elucidation of overall mechanism of antibody drugs to discuss the relationship between drug level and efficacy or anti-drug antibody assay. And for practical use, we have another activity into the expanded clinical application, comparative assay of original/biosimilar, and therapeutic drug monitoring. Furthermore, the antibody profiles and distribution mechanism[3] in tumor tissues based on nSMOL may be expected to aid the acceleration of the biopharmaceuticals.

**Acknowledgements**

This study was partly collaboration results with Dr. Hamada A of National Cancer Center and Dr. Yonezawa A of Kyoto University.

**References**

1. Iwamoto N, Shimada T, Umino Y, Aoki C, Aoki Y, Sato TA, Hamada A, Nakagama H. Selective detection of complementarity-determining regions of monoclonal antibody by limiting protease access to the substrate: nano- surface and molecular-orientation limited proteolysis. Analyst. 2014;139(3):576-580.

2. Iwamoto N, Shimada T, Terakado H, Hamada A. Validated LC-MS/MS analysis of immune checkpoint inhibitor Nivolumab in human plasma using a Fab peptide-selective quantitation method: nano-surface and molecular- orientation limited (nSMOL) proteolysis. J Chromatogr B Analyt Technol Biomed Life Sci. 2016;1023-1024:9-16.

3. Terrell-Hall TB, Nounou MI, El-Amrawy F, Griffith JIG, Lockman PR. Trastuzumab distribution in an in-vivo and in-vitro model of brain metastases of breast cancer. Oncotarget. 2017 Jul 26;8(48):83734-83744.

#### P120 Validated next generation sequencing assay for the characterization of the T-cell repertoire from RNA

##### Jennifer Sims, Martin Buchkovich, PhD, Victor Weigman, Jason Powers, John Pufky, Jennifer Mason, PhD, Patrick Hurban, PhD

###### Q2 Solutions, Morrisville, NC, USA

####### **Correspondence:** Patrick Hurban (patrick.hurban@q2labsolutions.com)

**Background**

Activation of T-cells during cell-mediated immunity is initiated by the stimulation of the T- cell receptor (TCR) by major histocompatibility complex-antigen complexes. While the entire TCR chain is diverse, most of the diversity is concentrated in a hypervariable complementarity-determining region 3 (CDR3) loop, the center of the antigen- binding site for the TCR. The frequency of a specific CDR3 sequence within the T-cell repertoire is a surrogate for the abundance of its corresponding T-cell clone. Deep sequencing of the TCR CDR3 region assists with resolving T- cell diversity, and, in oncology, detects specific clones or changes in clonality associated with anti-tumor immune responses. Here, we have validated an RNA based TCRβ/γ next-generation sequencing assay for use with whole blood, peripheral blood mononuclear cells (PBMCs) and formalin-fixed paraffin-embedded (FFPE) tissues.

**Methods**

TCR analysis was performed using RNA or total nucleic acids derived from whole blood, PBMCs or tumor FFPE specimens. The TCRβ/γ sequencing assay entailed gene specific cDNA synthesis, preparation of sequencing libraries, TCR gene specific amplification, sample barcoding and sequencing. A 2x150 bp sequencing was performed and ≥2 million paired-end reads for each sample were obtained. Data analysis was performed using Archer Analysis software.

**Results**

Rarefaction analysis was used to characterize the analytical sensitivity of the assay at varying sequence depths and input amounts, using RNA from whole blood and PBMCs from healthy donors. The total number of unique TCRβ RNA fragments observed using 20-1,200 ng of RNA ranged from 2,890 to 94,969 for whole blood, and 8,433 to 174,350 for PBMCs. The assay detected 1 TCRβ RNA fragment in the background of up to 174,350 TCRβ molecules, yielding a sensitivity of 5.7 *10-6. 100% accuracy was observed for both TCRβ and TCRγ using pre- characterized T-cell derived cell lines. FFPE tumor specimens from patients with lung and breast adenocarcinoma were used to determine TCR repertoire and its diversity. Each tumor was classified as having either high (≥15%), medium (7-14%), or low (≤7%) TILs in FFPE sections using H&E analysis. Using 400ng RNA, the number of unique TCR fragments detected in FFPE samples ranged between 53-1,632. FFPE samples were either polyclonal or highly clonal, however TCR clonality did not always correlate with TIL count.

**Conclusions**

We have validated an RNA-based NGS assay for the analysis of TCRβ/γ in whole blood, PBMC and FFPE specimens. The assay can support both TCR repertoire analysis and minimal residual disease monitoring in T-cell malignancies.

#### P121 Interaction of immune checkpoints in tumor-stromal microenvironment of primary and chemoreduced retinoblastoma

##### Lata Singh, PhD^1^, Mithalesh Singh^2^, Seema Kashyap^2^, Seema Sen, MD^2^, Moshahid A. Rizvi, PhD^1^

###### ^1^Jamia Millia Islamia, New Delhi, India; ^2^All India Institute of Medical Sciences, New Delhi, India

####### **Correspondence:** Moshahid A. Rizvi (mrizvi@jmi.ac.in)

**Background**

Interactions between malignant and non-malignant cells create the tumor microenvironment (TME). The non-malignant cells of the TME have a dynamic and tumor-promoting function at all stages of carcinogenesis. Cytotoxic T lymphocyte-associated antigen 4 (CTLA4), programmed death-1 (PD-1) and programmed death-ligand 1 (PD-L1) are key components of the immune checkpoint pathway. They play a crucial role in the regulation of T-cell activation and their expression in TME constitutes a predictive biomarker in cancers. It has recently been shown that chemotherapeutic agents could modify tumor microenvironment. Therefore, we investigated the expression of PD-1, PD-L1 and CTLA-4 in primary and chemoreduced retinoblastoma to define their significance in the tumor microenvironment with patient prognosis.

**Methods**

Expression of immune markers (PD-1, PD-L1 and CTLA-4 protein) was evaluated in 75 prospective cases of primary (Group I) and 25 cases of chemoreduced (Group II) enucleated retinoblastoma specimens by immunohistochemistry. mRNA expression of genes of interest were investigated by quantitative real time PCR (qPCR) and results were finally correlated with clinicopathological parameters and patient outcome by statistical analysis.

**Results**

Differential expression pattern of PD-1, PD-L1 and CTLA-4 proteins was found in both group I (primary retinoblastoma) and group II (chemoreduced retinoblastoma) cases. Immunohistochemistry showed cytoplasmic/membranous staining of these immune markers using their respective antibodies. Increased expression of PD-1, PD-L1 and CTLA-4 were found in stromal/immune cells of group II as compared to Group I. Expression of these immune markers showed significant correlation with poor tumor differentiation, tumor invasion and patient outcome (p<0.05).

**Conclusions**

This is the first of its kind study investigating the role of immune markers in primary retinoblastoma and their alteration in expression after chemotherapy. Tumor microenvironment of retinoblastoma showed expression of PD- L1 in primary patients and increased expression in PD-L1, CTLA-4 and PD-1 after chemotherapy. This paves the way for development of new strategies for treatment of chemoreduced retinoblastoma.

**Acknowledgements**

Dr. Lata Singh was supported by Department for Science and Technology (DST), Govt. of India, funding agency for providing National Post-Doctoral fellowship (N-PDF) and conducting this research.

**Ethics Approval**

This study was approved by Institute’s Ethical Committee, AIIMS (Ref. No. IEC-424/RP-6/2016)

**Consent**

Written consent was obtained from all the patient's guardian

#### P122 Validated next generation sequencing RNA-based assay of the IGHV mutation status and repertoire diversity

##### Jeran Stratford, Patrick Hurban, PhD, Victor Weigman

###### Q2 Solutions, Morrisville, NC, USA

####### **Correspondence:** Jeran Stratford (jeran.stratford@q2labsolutions.com)

**Background**

High-throughput sequencing of immune repertoire is increasingly used for clinical diagnosis, monitoring of residual disease, and development of cancer immunotherapies. The load of somatic hypermutations in the rearranged immunoglobulin heavy-chain variable region gene (IGHV) is a powerful prognostic biomarkers in CLL. CLL arising from unmutated IGHV cells (≥98% identity to closest germline rearrangement) are more aggressive and associate with poor prognosis, while CLL with mutated IGHV (<98% identity) have favorable outcomes and a higher rate of durable remissions after treatment with chemoimmunotherapy combinations. Historically, IGHV mutation status has been performed using Sanger sequencing looking at a single dominant clone. However, next- generation sequencing can reveal the sample's IGHV diversity. We have developed an NGS based IGHV assay with comparable results to Sanger sequencing. The assay is certified by the European Research Initiative on CLL (ERIC) [1].

**Methods**

Purified RNA is used as template to produce PCR amplicons of the VH1, VH3, VH4 and combined VH2/VH5/VH6/VH3-21 subfamilies. Productive PCR reactions are analyzed, sequencing libraries are prepared and sequenced using 2x300 paired-end Illumina MiSeq method. Paired reads within each sequenced amplicons are synthetically joined and aligned using all V-region sequences of the ImMuno GeneTics reference. Following filtering, the proportion of remaining reads corresponding to each IGHV gene is determined. The NCBI’s IgBlast software is used to assign a mutation status (<98% is mutated and ≥98% is unmutated) for each sequenced amplicons with ≥50% of reads aligned to a single gene. Sequenced amplicons with <50% of reads assigned to a single gene are classified “polyclonal”. A sample level mutation status is then determined by reconciling the mutation status for each sequenced amplicon. If all sequenced amplicons for a sample are polyclonal, the sample is classified as “polyclonal”. If the sample has any amplicons labeled as mutated and an absence of any unmutated labeled amplicons then the sample will be classified as mutated. If the sample has any amplicons with an unmutated status, the sample is classified as unmutated.

**Results**

For each sample we report the mutation status, percent identity to germline IGHV clonotypes, V/D/J assignment, and re-arrangement productivity. We also identify stereotype subset #2 and indicate its prognostic value.

**Conclusions**

Our assay has met ERIC guidelines [2] and was awarded IGHV assay certification. With its ability to characterize IGVH diversity, the utility of a certified NGS IGHV assay is critical in determining care for CLL patients now and providing a springboard for future biomarker development.

**References**

1. http://www.ericll.org/ighv-gene-mutational-analysis-certification/

2. Rosenquist R, Ghia P, Hadzidimitriou A, Sutton L-A, Agathangelidis A, Baliakas P, Darzentas N, Giudicelli V, Lefranc M-P, Langerak A, Belessi C, Davi F, Stamatopoulos K. Immunoglobulin gene sequence analysis in chronic lymphocytic leukemia: updated ERIC recommendations. Leukemia. 2017; 31:1477-1481.

#### P123 Peripheral blood lymphocyte responses in patients with renal cell carcinoma treated with high-dose interleukin-2

##### Rupal Bhatt, MD, PhD^1^, Lei Sun, Ph.D^2^, William Slichenmyer, MD^3^, Sean Rossi^2^, Juan Alvarez, PhD^4^, Wenxin Xu, MD^1^, Heather Losey, PhD^2^

###### ^1^Beth Israel Deaconess Medical Center, Boston, MA; ^2^Alkermes, Inc., Waltham, MA, USA; ^3^Alacrita Consulting, Waltham, MA, USA; ^4^Merck & Co, Boston, MA, USA

####### **Correspondence:** Lei Sun (Lei.Sun@alkermes.com)

**Background**

High-dose interleukin-2 (HD IL-2) activates the expansion of immunosuppressive regulatory T cells (Tregs), cytotoxic CD8+ T cells and natural killer (NK) cells. Previous data show that immunosuppressive ICOS+ Tregs are significantly expanded after treatment with HD IL-2 [1], but no data are readily available that specifically quantify and compare the levels of expansion of cytotoxic effectors such as CD8+ T cells and NK cells relative to Tregs. This study was conducted with the primary goal to assess the pharmacodynamic effects of HD IL-2 on numbers of circulating CD8+ T cells, NK cells, and Tregs.

**Methods**

Whole blood samples were collected prior to the first dose and after the last dose of treatment cycles 1 and 2 from a cohort of renal cell carcinoma patients receiving treatment with HD IL-2. CD8+ T cells, NK cells, and Tregs were quantified by flow cytometry. Safety and antitumor activity were monitored throughout the study period. Response was assessed according to RECIST, and best response was recorded.

**Results**

Ten patients with renal cell carcinoma were enrolled: median age 55 (range 39-62), male/female 6/4, ECOG PS of 0=9/1=1, and median number of prior therapies 2 (range 1-3). All treatment emergent adverse events seen were consistent with the known adverse event profile of HD IL-2 [2]. Capillary leak syndrome was reported in 5 patients. Five of the 10 patients achieved best response of partial response, and 1 patient had mixed response. Administration of HD IL-2 resulted in robust expansion of circulating Tregs with a mean maximum expansion of ~4-fold as compared to ~2-fold expansion of circulating CD8+ T cells and NK cells.

**Conclusions**

The safety profile and clinical response observed in this small cohort of patients were similar to previous published data [2]. A more robust expansion of Tregs over CD8+ T cells and NK cells was observed in patients treated with HD IL-2, consistent with the known biological activities of IL-2.

**Acknowledgements**

This study was funded by Alkermes, Inc. The authors gratefully acknowledge the patients and their families who participated in this study.

**References**

1. Sim GC, et al, IL-2 therapy promotes suppressive ICOS+ Treg expansion in melanoma patients. J. Clin. Invest. 2014;124(1): 99–110.

2. Marabondo S and Kaufman HL. High-dose interleukin-2 (IL-2) for the treatment of melanoma: safety considerations and future direction. Expert Opin. Drug Saf. 2017;16(12):1347-1357

**Ethics Approval**

This study was approved by the Beth Israel Deaconess Medical Center IRB, protocol #06-105.

#### P124 Quantitative multichannel immunofluorescence imaging to assess the immune composition of the T cell- inflamed tumor microenvironment in bladder cancer

##### Randy Sweis, MD, Ken Hatogai, Danny Kim, Yuanyuan Zha, PhD, Alexander Pearson, MD, PhD, Gary Steinberg, MD, Thomas Gajewski, MD, PhD

###### University of Chicago, Chicago, IL, USA

####### **Correspondence:** Randy Sweis (rsweis@uchicago.edu)

**Background**

A T cell inflamed tumor microenvironment is linked to improved prognosis and response to immunotherapy in bladder cancer. This immune phenotype can be measured by the presence of tumor-infiltrating T-cells with various gene expression signatures reflective of the immune response. Tumor-infiltrating BATF3+ dendritic cells have been shown in murine models to be critical for both priming an immune response and for recruitment of effector CD8+ T cells to the tumor microenvironment. In human bladder cancer specimens, the presence and distribution of tumor- infiltrating BATF3-dendritic cells has not been previously evaluated.

**Methods**

We performed quantitative multiplex immunofluorescence imaging on 64 bladder cancer specimens from patients to investigate the population of immune-infiltrating cells present in the tumor such as BATF3 cells and CD8+ T cells (Figure 1). We used a previously described immune gene expression signature to determine the presence of absence of the T cell-inflamed tumor microenvironment based on RNA sequencing for a subset of these samples. Immune cells proportions were calculated relative to the tumor cell counts in 5 randomly selected regions of interest for each specimen.

**Results**

The proportion of BATF3+ cells per 1000 tumor cells ranged from 0 to 6.32 with an average of 0.68. There were no BATF3+ cells in 23 out of 64 specimens (36%). The proportion of CD8+ cells per 1000 tumor cells ranged from 0 to 135 with an average of 10.4. There were no CD8+ cells in 8 out of 64 specimens (13%). In a subset of 18 samples for which RNA sequencing data were available, we found a 39-fold higher proportion of BATF3+ cells in tumors that were T cell-inflamed by gene expression profiling compared with those that were non-T cell-inflamed (P=0.02). T-cell inflamed tumors also had a 2.4-fold higher CD8+ cell proportion compared with non-T-cell inflamed tumors, but this difference did not reach significance (P=0.30).

**Conclusions**

The presence of tumor-infiltrating BATF3+ dendritic cells correlates strongly with the presence of a T cell-inflamed tumor microenvironment by gene expression profiling. Further analyses are ongoing to assess the impact of spatial relationships of immune cells and their association with immunotherapy response and outcomes.

**Ethics Approval**

Specimens used in this study were obtained through a protocol approved by the University of Chicago Institutional Review Board (IRB 15550B)

**Fig. 1 (abstract P124).**
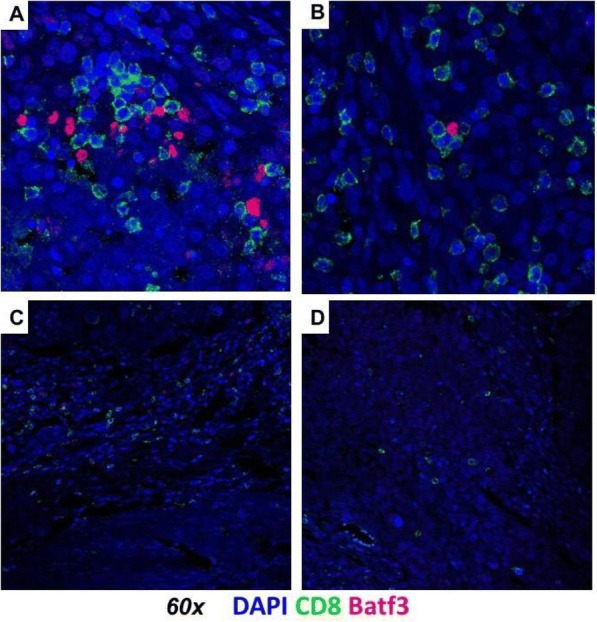
Multichannel immunofluorescence for CD8 and Batf-3

#### P125 DNA methylation biomarkers for noninvassive detection of hepatocellular carcinoma

##### David Taggart, PhD^1^, Dhruvajyoti Roy, PhD^1^, Gen Li^1^, Dan Liu^1^, Lianghong Zheng^1^, Kang Zhang^2^

###### ^1^The Laboratory for Advanced Medicine, Inc., West Lafyette, IN, USA; ^2^University of California San Diego, San Diego, CA, USA

####### **Correspondence:** David Taggart (david.taggart@lamoncogroup.com)

**Background**

The epigenetic inactivation of tumor suppressor genes by promoter hypermethylation is an important aspect of tumorigenesis. Indeed, aberrant methylation of CpG sites within genomic DNA isolated from cancer cells has been shown to correlate with clinically relevant information and has the potential to be used for cancer diagnosis and identification of the cancer tissue of origin. Malignant cells shed genomic DNA into circulation through both cell death and active release by viable cells. Therefore, investigating the methylation of cell-free DNA allows for the noninvasive detection and early diagnosis of cancers, such as hepatocellular carcinoma. Here, we identified and validated hepatocellular carcinoma-specific methylation markers for diagnosis of the disease with high sensitivity and specificity.

**Methods**

Banked samples were obtained for 130 subjects, including: 60 subjects diagnosed with hepatocellular carcinoma (Stage I to IV), 30 subjects without liver disease, 10 subjects diagnosed with benign liver disease and 30 subjects diagnosed with breast, colorectal or lung cancer. Samples were provided to the laboratory blinded for analysis. Cell- free DNA was then extracted from the samples, bisulfite converted, and DNA methylation was quantified by using the IvyGene® Platform. After data collection and analysis of all samples was complete, the samples were unblinded to calculate test performance.

**Results**

A total of 57 of the 60 samples drawn from subjects with hepatocellular carcinoma were correctly identified for an overall calculated sensitivity of 95%, with little difference between the sensitivity of detecting Stage I to Stage IV hepatocellular carcinoma (range 89% to 100%). Additionally, 29 of 30 samples drawn from subjects without liver disease and 9 of 10 samples drawn from subjects diagnosed with benign liver disease were correctly identified as non-cancer for a combined calculated specificity of 97.5%. Of the samples drawn from subjects with cancer other than liver cancer, 90% of breast cancer samples, 80% of colorectal cancer samples and 90% of lung cancer samples were correctly identified as not liver cancer, for a total calculated analytical specificity of 87%.

**Conclusions**

These data demonstrate the high diagnostic potential of cfDNA methylation markers in the blood for the detection of hepatocellular carcinoma. Indeed, quantification of cfDNA methylation may be a more sensitive and specific method for the detection of hepatocellular carcinoma than ultrasound, which is the current recommended imaging method for surveillance of high-risk populations.

**Ethics Approval**

This project was approved by the Institutional Review Boards (IRBs) of Sun Yat-sen University Cancer Center, Xijing Hospital, and West China Hospital.

#### P126 Peripheral immune monitoring identifies biomarkers of response and toxicity after neoadjuvant combination immunotherapy with ipilimumab (ipi) and high dose IFNα2b (HDI) in patients with melanoma

##### Ahmad Tarhini, MD, PhD^1^, Ghanashyam Sarikonda^2^, Arjun Khunger, MD^3^, Jack El-Sawada, MD^4^, Christian Laing^2^, Christine Vaupel, PhD^2^, Naveen Dakappagari^2^

###### ^1^Cleveland Clinic and Case Comprehensive Cancer Center, Cleveland, OH, USA; ^2^Navigate BioPharma Services, Inc., A Novartis Subsidiary, Carlsbad, CA, USA; ^3^Cleveland Clinic, Cleveland, OH, USA; ^4^University of Pittsburgh Medical Center, Pittsburgh, PA, USA

####### **Correspondence:** Ahmad Tarhini (tarhina1@ccf.org)

**Background**

Neoadjuvant ipi-HDI given over 6 weeks for locally/regionally advanced melanoma showed preoperative radiologic response of 36% and pathologic complete response (pCR) of 32% [1]. Mechanistic studies may illuminate the underlying mechanisms of immune susceptibility and resistance and identify biomarkers of response and toxicity.

**Methods**

Peripheral blood mononuclear cells (PBMC) from treated patients (N=28) on this trial were tested at baseline (before initiating ipi-HDI), 6-weeks, 3-months and 12-months following neoadjuvant ipi-HDI). High complexity (14-color) flow cytometry designed to detect key immunological biomarkers such as MDSCs, regulatory T cells, PD-1 and TIM3 expression on T-cells, and differentiation of T-cells into Th1, Th2 or Th17 phenotype were used to evaluate the effects of immunological biomarkers on safety and efficacy. Statistical significance was determined using R-package employing Kruskal’s test.

**Results**

Higher levels of Th1 cells (defined as CD45RA- CCR6- CXCR3+ CCR4-) correlated with preoperative radiological response (p=0.0070) while higher Th2 cells (defined as CD45RA- CCR6- CXCR3- CCR4+) was associated with progressive disease (0.0092). In evaluating pathologic response, a higher multimarker score consisting of Th1 cells and CD8+ central memory T-cells was associated with pCR (p=0.0406), in contrast, higher TIM3 expression on T- cells correlated with gross viable tumor (p=0.0472). Higher levels of phenotypically naive and effector memory CD8+ T-cells (p=0.0140) or lower levels of Th2 cells were associated with lower toxicity as well (p=0.0243). Finally, a multimarker score consisting of higher CD19+ and CD8+ cells was associated with lower toxicity (p=0.0013) and vice versa

**Conclusions**

Peripheral immune monitoring may identify predictive biomarkers of response and toxicity following combination neoadjuvant immunotherapy. Validation and long term immune monitoring studies are ongoing and will be presented.

**Trial Registration**

https://clinicaltrials.gov/ct2/show/NCT01608594NCT01608594

**References**

1. Tarhini AA, Rahman Z, Lin Y, Vallabhaneni P, Tawbi HA-A, Gnan A, et al. Neoadjuvant combination immunotherapy with ipilimumab (3 mg/kg or 10mg/kg) and high dose IFN-a2b in locally/regionally advanced melanoma. Journal of Clinical Oncology. 2016;34(15_suppl):9585-.

**Ethics Approval**

The study was initiated after approval from the institutional review board (IRB) and was conducted in accordance with the Declaration of Helsinki

**Consent**

A University of Pittsburgh IRB approved written informed consent (IRB# PRO12020161) was obtained from all patients.

#### P127 Characterization of changes in tumor immune microenvironment after treatment with neoadjuvant combination immunotherapy with ipilimumab (ipi) and high dose IFNα2b (HDI) in patients with melanoma

##### Arjun Khunger, MD^1^, Jennifer Bordeaux, PhD^2^, Ju Young Kim^2^, Christine Vaupel, PhD^2^, Naveen Dakappagari^2^, Ahmad A. Tarhini, MD, PhD^3^

###### ^1^Cleveland Clinic, Cleveland, OH, USA; ^2^Navigate BioPharma Services, Inc., A Nov, Carlsbad, CA, USA; ^3^Cleveland Clinic and Case Comprehensive Cancer Center, Cleveland, OH, USA

####### **Correspondence:** Ahmad A. Tarhini (tarhina1@ccf.org)

**Background**

Neoadjuvant ipi-HDI given over 6 weeks for locally/regionally advanced melanoma showed preoperative radiologic response of 36% and pathologic complete response (pCR) of 32% (Tarhini, et al. ASCO 2016). Mechanistic studies may reflect the underlying dynamics of tumor immune microenvironment (TME) in response to neoadjuvant treatment and offer important insights into immune mechanisms of response and resistance.

**Methods**

Tumor biopsy specimens of 28 patients with locally/regionally advanced melanoma, who were treated with neoadjuvant ipi-HDI were obtained at baseline and post-treatment assessment. Primary (archival) tumor samples collected at the time of diagnosis were also available. Multiplexed fluorescence immunohistochemistry combined with unique AQUA (Automated Quantitative Analysis) algorithms specifically designed to classify regulatory T cells (Tregs), Myeloid derived suppressor cells (MDSCs), CD3+ T cells, PD-1 expression, PD-L1 expression and IDO1/HLA-DR co-expression was used to assess tumor immune modulation in response to neoadjuvant immunotherapy. Statistical significance was determined using the paired t-test.

**Results**

Our analysis revealed that there was a significant decrease in the proportion of Tregs between pre-treatment primary and post-treatment biopsy samples (p=0.007). Also, there was a significant increase in the percentage of CD3+ T cells between pre-treatment primary and post-treatment biopsy samples (p= 0.005), supporting the hypothesis of increased generation of effector T-cells post-treatment. In correlation with pathologic response, there was a consistent trend towards a decrease in IDO1/HLA-DR co-expressing cells post-treatment as compared to pre- treatment primary (p= 0.067) and baseline (p=0.08) biopsy samples. No significant changes in expression of PD-1 or PD-L1 were seen between pre-treatment baseline and primary samples and post-treatment samples. In addition, no significant changes were detected in the immune profiles of pre-treatment baseline versus primary tumor biopsy.

**Conclusions**

A significant increase in CD3+ T cells and a significant decrease in Tregs was observed following treatment with neoadjuvant immunotherapy in our study. Consistent trends towards a decrease in IDO1/HLA-DR co-expression in responding patients support a role for IDO1 in immune resistance. Comparable TME profiles between preprimary and pre-treatment baseline tumor biopsies may be useful in guiding future studies.

**Trial Registration**

https://clinicaltrials.gov/ct2/show/NCT01608594NCT01608594

**References**

1. Tarhini AA, Rahman Z, Lin Y, Vallabhaneni P, Tawbi HA-A, Gnan A, et al. Neoadjuvant combination immunotherapy with ipilimumab (3 mg/kg or 10mg/kg) and high dose IFN-a2b in locally/regionally advanced melanoma. Journal of Clinical Oncology. 2016;34(15_suppl):9585-.

**Ethics Approval**

The study was initiated after approval from the institutional review board (IRB) and was conducted in accordance with the Declaration of Helsinki.

**Consent**

A University of Pittsburgh IRB approved written informed consent (IRB# PRO12020161) was obtained from all patients.

#### P128 Multiplexed immunofluorescent assay development for study of the PD-1/PD-L1 checkpoint in the tumor immune microenvironment (TIME)

##### Sneha Berry, MS, Sneha Berry, MS, Sneha Berry, MS, Nicolas Giraldo, MD PhD, Peter Nguyen, MS, Benjamin Green, BS, Haiying Xu, Aleksandra Ogurtsova, Abha Soni, DO, Farah Succaria, MD, Daphne Wang, MS, Charles Roberts, Julie Stein, MD, Elizabeth Engle, MSc, Drew Pardoll, MD, PhD, Robert Anders, MD, PhD, Tricia Cottrell, MD, PhD

###### Johns Hopkins University School of Medicine, Baltimore, MD, USA

####### **Correspondence:** Sneha Berry (jtaube1@jhmi.edu)

**Background**

Multispectral immunofluorescent (mIF) staining of formalin-fixed paraffin-embedded (FFPE) tissue allows spatially-resolved quantitative analysis of cell position and protein expression. The design and validation of mIF panels is a challenge. Our goal was to develop a 7-plex assay for characterizing PD-1 and PD-L1 expression, with high sensitivity for multiple markers and minimal bleed-through between fluorescent channels, while avoiding steric hindrance among markers occupying the same cellular compartment.

**Methods**

Single IF slides were stained for PD-1, PD-L1, CD8, FoxP3, CD163, and a tumor marker (e.g. Sox10/S100 for melanoma) using primary antibodies at manufacturer’s recommended concentrations and visualized with an Opal kit (PerkinElmer). Positive signal was compared to chromogenic IHC (n=3 tonsil specimens). In some instances, the kit’s HRP-polymer was substituted for one that provided greater amplification. Primary antibody titrations were performed, and the concentration with comparable signal to chromogenic IHC that showed the highest IF signal to noise ratio was selected. Using the selected primary antibody concentration, TSA dilution series were performed on n=5 tumor specimens to minimize bleed-through. Finally, the optimized single IF stains were combined into multiplex format, which was again validated to ensure no positivity loss. Images were scanned with the Vectra 3.0 and processed using inForm (Ver 2.3).

**Results**

The percent positive pixels for CD163, CD8, and tumor marker expression by IF were comparable to chromogenic IHC with manufacturer’s recommended protocols (p>0.05). However, PD-1, PD-L1, and FoxP3 showed ~50% loss of signal (p<0.05), which was recovered by replacing the Opal kit’s secondary HRP polymer with PowerVision (Leica). Unbalanced fluorescence intensities between 540 to 570 Opal dyes resulted in significant bleed-thorough and led to false positive pixels. This error was minimized >2 fold (2.5% to 1.1%) by concentrating the 570 dye and ensuring that this dye pair was used to study markers in different cellular compartments (nuclear FoxP3 vs. membrane CD8), so any residual bleed-through could be discounted during image analysis. Using the optimized panel, we are able to reliably identify cell types contributing PD-L1 and PD-1 to the TIME, and even resolve populations of PD-1high vs. PD-1low lymphocytes.

**Conclusions**

We demonstrate successful optimization of a 7-color multiplex panel characterizing the PD-1/PD-L1 axis to provide high quality data sets for whole slide or regional analysis of the TIME. With the use of multiparametric assays such as this, we hope to guide improved approaches to patient selection and potentially identify additional tumor types likely to respond to anti-PD-(L)1 immunotherapy.

**Ethics Approval**

The study was approved by Johns Hopkins University Institutional Review Board.

#### P129 Simultaneous single cell analysis of multiple analytes resolves T cell populations at high resolution

##### Sarah Taylor, PhD^1^, Katherine Pfeiffer^1^, Michael Stubbington, PhD^1^, Josephine Lee^1^, Jerald Sapida^1^, Liselotte Brix, phd^2^, Kivin Jacobsen, PhD^2^, Bertrand Yeung^3^, Xinfang Zhao^3^, Tarjei Mikkelsen^1^, Deanna Church, PhD^1^

###### ^1^10x Genomics, Pleasanton, CA, US; ^2^Immudex, Copenhagen, Denmark; ^3^BioLegend, San Diego, CA, USA

####### **Correspondence:** Sarah Taylor (sarah.taylor@10xgenomics.com)

**Background**

Characterization of lymphocyte types and understanding their antigen binding specificities are key to the development of effective therapeutics. Recent technological advancements have enabled the integration of simultaneous cell-surface protein, transcriptome, immune repertoire and antigen specificity measurements at single cell resolution, providing comprehensive, high-throughput characterization of immune cells.

**Methods**

Using the 10x Genomics Single Cell Immune Profiling Solution with Feature Barcoding technology in conjunction with TotalSeq™-C oligo-conjugated antibodies (BioLegend) and DNA barcoded MHC Dextramer® reagents (Immudex), we performed multi-omic characterization of PBMCs from cytomegalovirus (CMV) seropositive and seronegative donors. Next generation sequencing libraries were made following the 10x Genomics workflow, where gene expression and immune repertoire libraries are generated alongside libraries from DNA barcodes conjugated to antibodies or MHC Dextramer reagents, allowing quantification of cell surface proteins and identification of TCR specificities. Analysis was performed using the latest version of Cell Ranger (v3.0). The TCR-dist algorithm was used to identify clusters of related TCR sequences and enriched CDR3 motifs.

**Results**

Combining single cell TCR assembly with barcoding of MHC Dextramer reagents allowed the identification of full length, paired alpha and beta TCR sequences with specificity for known CMV antigens. Cells were also labelled with barcoded antibody reagents to allow cell type characterization based on surface protein expression to augment the transcriptomic information provided by the gene expression portion of the assay. The combination of these single cell assays allowed the identification of CD8+ cell populations with specificity for CMV/MHC. Multiple TCRs that bound CMV/MHC were observed and we identified enriched amino acid motifs within the TCR sequences. We analyzed the distribution of CMV-binding TCRs with similar sequences shared across individual donors and compared the observed CMV-binding TCR sequences with those previously reported in TCR-antigen databases.

**Conclusions**

The analytical approaches outlined here provide a systematic and scalable method for deciphering TCR-peptide MHC specificity, with clear implications in understanding the complexity of the tumor microenvironment. The multi-omic combination of gene expression, paired adaptive immune receptor repertoire, antibody-based detection of cell surface proteins and Dextramer-based analysis of antigen binding specificity for the same single lymphocytes allows the comprehensive characterization of immune cell populations at unprecedented resolution and throughput. Identifying discrete cellular phenotypes that underlie immune receptor specificity and antigen binding capabilities is critical for developing a better understanding of the adaptive immune response to cancer; leveraging this understanding will be key in the development of successful cellular and transgenic immunotherapies.

#### P130 A measurable immune response in the tumors and lymph nodes of patients with lymph node positive and lymph node negative breast cancer

##### Archana Thakur, PhD^1^, Dana Schalk^1^, Tayson Lin^2^, Johnson Ung^1^, Griffin Calme, BS^2^, Johnson Ung^1^, Lawrence G. Lum, MD, DSc^1^, Lydia Choi^3^

###### ^1^University of Virginia Cancer Center, Charlottesville, VA, USA; ^2^Wayne State University, Detroit, MI, USA; ^3^Karmanos Cancer Institute, Detroit, MI, USA

####### **Correspondence:** Lawrence G. Lum (lgl4f@hscmail.mcc.virginia.edu)

**Background**

The functional significance of various immune cell subsets may provide important information for maximizing the prognostic value of immunoscoring. In early stage breast cancer patients with no detectable lymph node invasion relapse rate is about 20-30% while one-third of lymph node positive breast cancer patients remain free of distant metastasis. The prognostic gap in this group of patients remains and the vast majority of the patients in both groups had no prognostic marker to assist in driving clinical management based on risk factors. We hypothesize that immune cell population and its T cell activation status (Th1 type) in the resected breast tumors and sentinel lymph nodes may serve as immunological biomarkers of tumor aggressiveness and offer useful prognostic information to facilitate specific therapy related clinical decision as well as response to therapeutic options. Since the nodal immune environment is influenced in part by tumor-derived factors, we tested a small cohort for cytoplasmic expression of IFN-gamma and IL-10 to explore the association between Th1 and Th2 status of the TILs along with the triple staining for the CD3+ T cells, CD20+ B cells and CD68+ macrophages in tumors and lymph nodes biopsies with clinical outcomes of breast cancer patients.

**Methods**

Biopsies from four groups of patients were examined for T cell activation status, 1) no recurrence until present and node negative; 2) no recurrence until present and node positive; 3) recurrence in last 5 years and node negative; 4) recurrence in last 5 years and node positive. Tissue slides from sentinel node biopsies and tumor resections from thirty women who underwent sentinel node biopsy with invasive breast cancer resection were dual stained for CD3/IL-10, CD3/IFN-gamma or triple stained for CD3/CD20/CD68. Slides were imaged using a Digital Scanner, the density and intensity of each cell type and cytokine was recorded as the number of positive cells per unit tissue surface area.

**Results**

Our data show that patients who had no evidence of disease after 5 years had weak to moderate expression of IFN-gamma by T cells while patients who progressed rapidly had no expression of IFN-gamma by T cells in the tumors or in the lymph nodes.

**Conclusions**

Understanding of the immune cell environment within the tumors and lymph nodes may have implications for improving clinical outcome of cancer patients.

**Ethics Approval**

The study was approved by Wayne State University's IRB, approval number 634183

#### P131 High-plex predictive marker discovery for melanoma immunotherapy treated patients using NanoString® Digital Spatial Profiling

##### Maria Toki, MD, MSc^1^, Pok Fai Wong, MD, MPhil^1^, James Smithy, MD, MHS^2^, Harriet Kluger, MD^1^, Chris Merritt, PhD^3^, Giang Ong, MS^3^, Sarah Warren, PhD^3^, Joseph Beechem, PhD^3^, David Rimm, MD, PhD^1^

###### ^1^Yale University School of Medicine, New Haven, CT, USA; ^2^Brigham and Women's Hospital Department, Boston, MA, USA; ^3^NanoString Technologies, Seattle, WA, USA

####### **Correspondence:** David Rimm (david.rimm@yale.edu)

**Background**

NanoString Digital Spatial Profiling (DSP)*, a technology previously validated to quantitative immunofluorescence, offers the capacity of highly multiplexed immune marker quantitative measurements with spatial resolution within specific regions of interest (ROI) on formalin-fixed, paraffin-embedded (FFPE) tissue. Here, we used NanoString DSP to explore the predictive value of a 44plex panel of immune markers measured in multiple compartments in a melanoma immunotherapy (ITx) treated cohort.

**Methods**

NanoString DSP technology uses a cocktail of primary antibodies conjugated to indexing DNA oligos. ROI on the tissue are selected with fluorescently labeled antibodies, and oligos from that region are UV cleaved, sipped up, and quantified on the nCounter® platform. Here, we used a tissue microarray (TMA) cohort which includes 60 Itx treated melanoma patients to identify potential predictive markers of survival and response using a 44plex panel of immune markers collected sequentially and quantified in three different compartments: in macrophage, leukocyte and the melanocyte ROI, defined by CD68+, CD45+ and S100+HMB45+ respectively. Each patient was represented by two cores and the counts for each marker were averaged.

**Results**

Biomarker counts were highly concordant across unique TMA cores from the same patient tumor. High concordance between DSP and quantitative fluorescence was also seen as a validation for the DSP method. Out of the 44plex panel measured in three different ROIs, 13 and 16 immune markers were found to be associated with prolonged progression free survival (PFS) and overall survival (OS). Perhaps the most striking finding was that, using a CD68 specific compartment marker measurement, we found that PD-L1 expression in macrophages and not in tumor was a predictive marker for PFS, OS and response. Other notable compartment-specific biomarkers found to be associated with outcome included beta 2-microglobulin, HLADR, CD8 and IDO1.

**Conclusions**

NanoString DSP capacity for highly multiplexed immune marker measurements on selected compartments allowed the discovery of multiple predictive markers in a single TMA section of a melanoma ITx cohort. This tool represents the highest multiplexed spatially informed method for discovery without the liabilities of cycling methods. These findings can be used for the construction of predictive signatures involving multiple compartments reflecting the complexity of the tumor microenvironment. Future studies on the DSP platform are underway with multiplexing capacity to nearly 1000 biomarkers on the same tissue section.*FOR RESEARCH USE ONLY. Not for use in diagnostic procedures.

**Ethics Approval**

The study was approved by the Yale Human Investigation Committee protocol #9505008219 and conducted in accordance with the Declaration of Helsinki.

#### P132 RNA sequencing of rare antigen-specific T cells and tissue micro-regions using the RareCyte platform

##### Lance U'Ren, DVM, PhD, Rebecca Podyminogin, Nolan Ericson, Eric Kaldjian, MD, Tad George, PhD

###### RareCyte Inc., Seattle, WA, USA

####### **Correspondence:** Lance U'Ren (luren@rarecyte.com)

**Background**

The immune system provides antigen-specific protection against pathogens as well as malignancies, both of which evolve strategies to evade immune surveillance and containment. Effective immune response often depends on activation of rare immune cell sub-types, whose function are influenced by the tissue micro-environment, the pathogen or cancer, and other factors. The RareCyte platform provides integrated multi-parameter imaging and retrieval capabilities that allow phenotypic identification and isolation of rare cells and microscopic regions of interest for sequence and transcript level analyses in order to study the complexity of host defense.

**Methods**

We utilized RareCyte’s CyteFinder platform to identify rare antigen-specific T cells by using tetramers against influenza-specific T cell receptors (TCR) in both unstimulated and influenza peptide stimulated samples. Additionally, we isolated 40 μm regions of interest (ROIs) from B and T cell regions of human tonsil tissue. Single cells and tissue ROIs were isolated with the integrated CytePicker module. SMART-seq2 whole transcriptome amplification was carried out on single retrieved cells and ROIs, followed by Illumina Nextera XT library preparation and sequencing on an Illumina MiSeq. Expression analysis was carried out with DESeq2 and TCR sequences were retrieved from the antigen-specific T cell RNAseq datasets utilizing the TraCeR software package. For 6-color imaging, tonsil and melanoma tissue sections were immunofluorescent stained with panels containing BV421, Alexa488, Alexa647, sytox orange, qDot-625, and qDot-800 fluorophores and scanned with the CyteFinder system.

**Results**

We validated T cell activation by gene expression analysis revealing upregulation of transcripts in pathways such as TCR signaling and inflammatory response/cytokine signaling and were able to match paired alpha/beta TCR sequences with previously published influenza antigen-specific T cell databases. Expression analysis referencing retrieved tonsil T cell zone ROIs against B cell zone ROIs resulted in an expected differential analysis, such as upregulation of CD8a, CCL19, and CCL21 and downregulation of CD38, CR2, and CXCL13.

**Conclusions**

We were able to confirm that anti-influenza T cells isolated with the RareCyte were specific for expected TCRs and that peptide stimulated cells had an activated phenotype through RNA sequencing. Additionally, we validate use of the instrument for the picking of tissue micro-regions with confirmation of the cell type and tissue micro- environment via RNA sequencing. We also demonstrate that the system can be used for 6-color tissue imaging of melanoma cells and tumor-associated immune cells.

**Ethics Approval**

Human samples in this study were procured from commercial vendors who collected them according to their established ethics policies

#### P133 Spatially resolved and multiplexed immunoprofiling of NSCLC using imaging mass cytometry reveals distinct functional profile of CD4 and CD8 TILs associated with response to immune checkpoint blockers

##### Franz Villarroel-Espindola, PhD^1^, Miguel Sanmamed, MD, PhD^2^, Jonathan Patsenker^1^, Ya-Wei Lin^3^, Brian Henick, MD^4^, Jovian Yu^1^, Mark Verburg^5^, Tayf Badri^1^, Jon Zugazagoitia, MD^1^, Daniel Carvajal-Hausdorf, MD^6^, Ruth Montgomery, PhD^1^, Roy Herbst, MD, PhD^1^, Lieping Chen, MD, PhD^1^, David Rimm, MD, PhD^1^, Tal Shnitzer, PhD^3^, Ronen Talmon, PhD^3^, Yuval Kluger, PhD^1^

###### ^1^Yale School of Medicine, NEW HAVEN, CT, USA; ^2^Clínica Universidad de Navarra, New Haven, CT, USA; ^3^Israel Institute of Technology, Haifa, Israel; ^4^Columbia University, New York, NY, USA; ^5^Stony Brook University, New York, NY, USA; ^6^Clinica Alemana Universidad Desarrollo, New Haven, CT, USA

####### **Correspondence:** Franz Villarroel-Espindola (kurt.schalper@yale.edu)

**Background**

Reinvigoration of anti-tumor immunity by blocking co-inhibitory checkpoints has revolutionized the treatment of numerous human malignancies including non-small cell lung cancer (NSCLC). Understanding the mechanisms mediating anti-tumor effect and determinants of sensitivity/resistance to treatment remain major challenges. Efforts to comprehensively analyze intact tumor specimens have been limited by the number of targets that can be measured with preserved tissue architecture and in native cell conditions. We used a 29-marker IMC panel to study the immune composition of NSCLC and association with benefit to immune checkpoint blockers.

**Methods**

The IMC panel was established by validation of individual metal-conjugated primary antibodies in formalin-fixed/paraffin-embedded (FFPE) samples using cell-line controls, human tissue specimens and comparison with multiplexed immunofluorescence. Markers included structural tissue components: GAPDH/Histone- 3/LipoR/DNAintercalators; cell-phenotype markers: Vimentin/Cytokeratin/CD3/CD4/CD8/CD20/CD68/FOXP3/CD45RO; functional indicators: TBET/PD-1/LAG- 3/TIM-3/CD25/B2M/KI-67/GZB; and candidate immunomodulatory targets: PD-L1/PD-L2/B7-H3/B7-H4/PD- 1H/VISTA/IDO-1/CD47. We studied 12 NSCLC/normal-lung tissue pairs and 30 baseline tumor specimens from patients receiving PD-1 axis blockers represented in tissue microarrays. Treated cases included 12 with durable clinical benefit (DCB) and 18 without benefit (NDB). We analyzed the overall levels of the markers and profiles of individual T-cell subpopulations using MCDviewer software. We performed integrated and spatially-resolved analysis in marker-selected patches using mathematical integration of signal and training of a support vector machine classifier to distinguish patients with response/resistance to treatment.

**Results**

The IMC panel showed specificity for individual markers, reproducibility across experimental runs and concordance with immunofluorescence. NSCLCs showed increased CD4+/CD8+/CD20+ TILs with higher expression of functional markers than case-matched non-tumor lung tissue. In cases treated with immune checkpoint blockade we identified prominent differences in the T-cell profile between patients with benefit/DCB compared with those without response to treatment/NDB characterized by higher levels of effector memory CD8+/CD45RO+ TILs and lower levels of T-cell immune inhibitory receptors. Cases with primary resistance to treatment were associated with CD4+ or CD8+ TILs containing increased levels of both activation (CD25/TBET/GZB/Ki-67) and immune suppression/dysfunction markers (PD-1/LAG-3/FOXP3). The spatially resolved machine learning classifier stratified DCB/NDB cases with 90% of concordance.

**Conclusions**

We have standardized a 29-marker immuno-oncology multiplexed IMC panel for simultaneous, quantitative and spatially resolved analysis of multiple protein targets in FFPE specimens. Our results show higher adaptive immune response in NSCLC relative to patient-matched non-tumor lung and identify a distinct activated/dysfunctional profile of T-cells in patients with primary resistance to immune checkpoint blockers. Our work supports further exploration of machine learning-based classifiers to objectively integrate markers and use as biomarkers.

**Acknowledgements**

Thanks to Yale CyToF core facility and Fluidigm(R).Funding: Department of Defense Lung Cancer Research Program Career Development Award #LC150383; Yale SPORE in Lung Cancer P50CA196530; Stand Up To Cancer - American Cancer Society Lung Cancer Dream Team Translational Research Grant SU2C-AACR-DT17- 15; Lung Cancer Research Foundation grant.

**Ethics Approval**

The study was approved by Yale Human Investigation Committee protocol #1608018220

#### P134 Prognostic and predictive value of baseline biomarkers in advanced non-small cell lung cancer (NSCLC)

##### Huifen Wang, PhD, Robert McEwen, Ketan Patel, PhD, Binbing Yu, J Carl Barrett, Kris Sachsenmeier, PhD

###### AstraZeneca, Gaithersburg, MD, USA

####### **Correspondence:** Binbing Yu (yub@MedImmune.com)

**Background**

Expression levels of biomarkers measured prior to treatment may indicate high tumor burden and be useful to inform or optimize treatment options or be predictive of response.

**Methods**

We studied baseline status (post hoc) for several biomarkers that are commonly used in the “real world” clinical oncology setting, lactate dehydrogenase (LDH), neutrophil to lymphocyte ratio (NLR), carcinoembryonic antigen (CEA), and prostate-specific antigen (PSA). We used the FlatIron Health database to correlate baseline biomarker data with respect to overall survival (OS) to predict immune oncology (IO)/chemo/targeted therapy response in NSCLC. The study included patients diagnosed with stage IIIB/IV NSCLC, aged ≥18 years at diagnosis, who had a non-missing value of blood-based baseline biomarkers of interest, received ≥1 IO/chemo/targeted therapy, and who were not participating in clinical trials. Pre-specified values along with median values of each biomarker served as cutoffs to define patients with high and low level of each biomarker. Time from NSCLC diagnosis to first line (1 L) treatment start, and from 1 L treatment start to end of follow-up/death were used to define baseline and OS, respectively. Covariates of practice type, age at diagnosis, gender, histology, stage at diagnosis, smoking status, performance status, number of treatment lines, and 2 L treatment type (IO vs chemo/targeted therapy) were studied and included in the multivariate model to assess biomarkers’ prognostic value, and used for propensity score matching between the IO and chemo/targeted therapies.

**Results**

Of 10,678 patients included, most received 1 L (57.6%) and 2 L (26.8%) therapies. Of 716 patients on 1 L IO therapy, 18% remained on IO therapy; while 16.5% of patients on 1 L chemo/targeted therapy switched to IO therapy in 2 L. Baseline LDH, CEA, and NLR showed significant prognostic benefit for all treatments. A potential predictive value of LDH and CEA in response to IO therapy was observed: Patients on IO therapy with a low baseline biomarker level had improved OS vs patients on IO therapy with a high baseline biomarker level and chemo/targeted therapy-treated patients.

**Conclusions**

These data support the hypothesis that in NSCLC, patients with high tumor burden and/or specific leukocyte profiles are generally non-responsive to IO therapies.

#### P135 Integrative genomic and proteomic analysis identifies cancer subtypes and signaling networks associated with aberrant tumor expression of VISTA

##### Duncan Whitney, PhD^1^, Anna Ma, MS^1^, Ramachandra Katabathula, PhD^2^, Timothy Wyant^1^, Jefferson Parker^1^, Salendra Singh, MS^2^, David Tuck^1^, Vinay Varadan, PhD^2^

###### ^1^Curis Inc, MA; ^2^Case Western Reserve University, Cleveland, OH, USA

####### **Correspondence:** Duncan Whitney (dwhitney@curis.com)

**Background**

VISTA is a negative regulator of T-cell and myeloid cell function and is gaining importance as a target for cancer immunotherapy [1-3]. VISTA is highly expressed in tumor-infiltrating leucocytes, particularly within the myeloid lineage. Recent evidence suggests that tumor cells themselves also express VISTA, exacerbating the immunosuppressive milieu within the tumor microenvironment [4]. Determining tumor subtypes that overexpress VISTA can inform the indication selection for VISTA-targeting agents and design of clinical trials in specific patient populations.

**Methods**

Genome-scale data was obtained from the TCGA Pan-Cancer Atlas dataset spanning 33 cancer types and >11,000 tumor samples [5]. DNA Methylation profiles were utilized to model leukocyte infiltration in individual tumor samples. The immune-cell component of VISTA expression was modeled using a nonlinear non-parametric regression model (LOESS). Non-immune compartment tumors with high VISTA expression, independent of leukocyte infiltration scores, were selected as likely over-expressing VISTA. VISTA-High tumor types and subtypes were determined and signaling network activities on a per-sample basis were derived using the InFlo systems framework. Pathways associated with aberrant VISTA expression were identified using a multivariate Gaussian model of gene expression and immune infiltration. Quantitative immunofluorescence (QIF) of VISTA protein expression was run on tissue microarrays (TMA): breast (n=602), lung (n=271), colorectal (n=2017), and ovarian (n=250) tumors. VISTA expression in stromal and tumor compartments was differentiated using labeled anti- cytokeratin and anti-VISTA antibodies.

**Results**

We identified 703 cancer samples (6.4%) exhibiting likely aberrant tumor cell expression of VISTA on tumor cells.

VISTA-high tumors were enriched (P<< 0.01) for Low Grade Glioma (LGG), Glioblastoma Multiforme (GBM), Kidney Renal Clear Cell Carcinoma (KIRC), Head and Neck Squamous Cell Carcinoma (HNSC), Sarcoma (SARC) and Mesothelioma (MESO). The CpG Island Methylator Phenotype (CIMP)-high of LGG and GBM, as well as the Atypical and Classical subtypes of HNSC were markedly enriched within the VISTA-High tumor population. We also identified subpopulations of lung (5.2%), breast (4.5%), colorectal (3.9%), and ovarian (4.1%) cancers to exhibit VISTA over-expression in the tumor compartment. Signaling networks identified as significantly associated with VISTA over-expression (P < 0.05) were identified. VISTA protein was expressed primarily in the stroma in nearly all (90-100%) of the TMA histospots, whereas between 5-10% showed VISTA expression in the tumor compartment, consistent with findings from the TCGA analysis.

**Conclusions**

Overexpression of VISTA on tumor cells was consistent between RNA analysis of TCGA and QIF- analyses of proteins in TMAs. This study represents the most comprehensive analysis of VISTA expression to date.

**References**

1. Lines JL, Sempere LF, Broughton T., Wang L, Noelle R. VISTA is a novel broad-spectrum negative checkpoint regulator for cancer immunotherapy. Cancer Immunol Res. 2014; (2): 510-7.

2. Nowak EC, Lines JL, Varn FS, Deng J, Sarde A, Mabaera R, Kuta A, Le Mercier I, Cheng C, Noelle RJ. Immunoregulatory functions of VISTA. Immunol Rev. 2017. 276: 66-79.

3. Gao J, Ward JF, Pettaway CA, Shi LZ, Subudhi SK, Vence LM, Zhao H, Chen J, Chen H, Efstathiou E, Troncoso P. VISTA is an inhibitory immune checkpoint that is increased after ipilimumab therapy in patients with prostate cancer. Nature medicine. 2017; 23(5): 551.

4. Boger C, Behrens HM, Kruger S, Rocken C. The novel negative checkpoint regulator VISTA is expressed in gastric carcinoma and associated with PD-L1/PD-1: A future perspective for a combined gastric cancer therapy? Oncoimmunology. 2017.

5. Thorsson V, Gibbs DL, Brown SD, Wolf D, Bortone DS, Ou Yang TH, Porta-Pardo E, Gao GF, Plaisier CL, Eddy JA, Ziv E, Culhane AC, Paull EO, Sivakumar IKA, Gentles AJ, Malhotra R, Farshidfar F, Colaprico A, Parker JS, Mose LE, Vo NS, Liu J, Liu Y, Rader J, Dhankani V, Reynolds SM, Bowlby R, Califano A, Cherniack AD, Anastassiou D, Bedognetti D, Rao A, Chen K, Krasnitz A, Hu H, Malta TM, Noushmehr H, Pedamallu CS, Bullman S, Ojesina AI, Lamb A, Zhou W, Shen H, Choueiri TK, Weinstein JN, Guinney J, Saltz J, R. A. Holt RA, Rabkin CE, Cancer Genome Atlas Research Network, Lazar AJ, Serody JS, Demicco EG, Disis ML, Vincent BG, Shmulevich L. The immune landscape of cancer. Immunity. 2018; 48: 812-30

#### P136 Genetic immunosignatures associate with progression-free survival in advanced soft tissue sarcoma patients treated on a Phase 2 trial of the VEGF receptor inhibitor axitinib plus pembrolizumab

##### Breelyn Wilky, MD^1^, SuFey Ong^2^, Sarah Warren, PhD^2^, Alessandra Cesano, MD, PhD^2^, Despina Kolonias^1^, Eric Wieder, PhD^1^, Deukwoo Kwon, PhD^1^, Andrew Rosenberg, MD^1^, Jonathan Trent, MD, PhD^1^, Krishna Komanduri, MD^1^

###### ^1^University of Miami - SCCC, Miami, FL, USA; ^2^NanoString Technologies, Seattle, WA, USA

####### **Correspondence:** Breelyn Wilky (b.wilky@med.miami.edu)

**Background**

Vascular endothelial growth factor (VEGF) maintains the immunosuppressive tumor microenvironment by limiting T cell infiltration and promoting suppressive immune cell phenotypes. Accordingly, simultaneous blockade of VEGF with checkpoint inhibitors has led to improved immune cell infiltration and tumor responses in melanoma and renal cell carcinoma. In soft tissue sarcomas (STS), response rates to PD-1 monotherapy or dual CTLA4/PD-1 blockade are modest at 16-19% [1,2]. We hypothesized that addition of VEGF receptor inhibitor axitinib plus anti- PD1 checkpoint inhibitor pembrolizumab would improve responses in STS. In a Phase II study of axitinib/pembrolizumab in 30 patients with advanced STS (NCT02636725), we observed 4 month progression-free survival (PFS) of 47.7%. We obtained tumor biopsies from study patients and evaluated expression of immune- related gene signatures in respect to clinical outcomes.

**Methods**

Formalin-fixed, paraffin embedded core needle tumor biopsies were obtained from study patients at baseline and after 12 weeks on study treatment. RNA was extracted from unstained slides and hybridized with NanoString® IO360 beta gene expression panel (for research use only) prior to analysis on the nCounter® platform. After normalization with housekeeping genes and technical controls, expression of immune subset signatures was analyzed using research algorithms developed by NanoString.

**Results**

28 baseline and 14 on-treatment biopsies contained sufficient tumor for analysis after pathologist review. STS histologies included alveolar soft part sarcoma (ASPS, n=9), leiomyosarcomas (LMS, n=6), undifferentiated pleomorphic sarcoma (UPS, n=5), and other (n=9). Higher baseline expression of antigen presentation machinery, type 1 interferons, immunoproteosome, and interferon downstream signaling signatures was significantly associated with PFS > 4 months, whereas B cell, NK cell, and NK CD56dim signatures were negatively associated with PFS. Higher glycolysis gene expression in on-treatment samples relative to baseline was associated with PFS > 4 months. UPS significantly differed from other subtypes with higher baseline expression of proliferation, stroma, myeloid inflammation, PD-L2, B7-H3, and TGF-beta signatures. UPS immunoprofiles were significantly altered with treatment relative to other subtypes, with higher expression of myeloid inflammation, inflammatory chemokine, B cell, dendritic cell, and TGF-beta signatures. ASPS demonstrated higher baseline expression of endothelial cell, antigen presentation machinery, and apoptosis signatures, with lower proliferation relative to other subtypes.

**Conclusions**

This is the first analysis in STS patients treated with immunotherapy to correlate genetic expression signatures with clinical outcomes. Pathways identified by the NanoString IO360 beta panel will require prospective validation, but may ultimately serve as predictive biomarkers, and suggest alternative targets to further enhance efficacy of immunotherapy in STS.

**Trial Registration**

Clinicaltrials.gov: NCT02636725

**References**

1. Tawbi HA, Burgess M, Bolejack V, Van Tine BA, Schuetze SM, Hu J, D'Angelo S, Attia S, Riedel RF, Priebat DA, Movva S, Davis LE, Okuno SH, Reed DR, Crowley J, Butterfield LH, Salazar R, Rodriguez-Canales J, Lazar AJ, Wistuba II, Baker LH, Maki RG, Reinke D, Patel S. Pembrolizumab in advanced soft-tissue sarcoma and bone sarcoma (SARC028): a multicentre, two-cohort, single-arm, open-label, phase 2 trial. Lancet Oncol. 2017; 18(11):1493-1501.

2. D'Angelo SP, Mahoney MR, Van Tine BA, Atkins J, Milhem MM, Jahagirdar BN, Antonescu CR, Horvath E, Tap WD, Schwartz GK, Streicher H. Nivolumab with or without ipilimumab treatment for metastatic sarcoma (Alliance A091401): two open-label, non-comparative, randomised, phase 2 trials. Lancet Oncol. 2018; 19(3):416- 426.

**Ethics Approval**

The study was approved by the University of Miami Institutional Review Board, approval number 20150932.

#### P137 A systematic literature review (SLR) of tumor mutational burden (TMB) and efficacy with immunotherapy (IO) in lung cancer

##### Connor Willis, PharmD^1^, Michelle Fiander^1^, Dao Tran, PharmD^2^, Beata Korytowsky^3^, John-michael Thomas, PharmD^3^, Signe Fransen^3^, Florencio Calderon^3^, Teresa Zyczynski^3^, Lisa Siegartel^3^, Diana Brixner^1^, David Stenehjem^2^

###### ^1^University of Utah, Salt Lake City, UT, USA; ^2^University of Minnesota, Duluth, MN, USA; ^3^Bristol-Myers Squibb, Plainsboro, NJ, USA

####### **Correspondence:** Diana Brixner (diana.brixner@utah.edu)

**Background**

TMB is an emerging biomarker that may predict response to IO. This SLR evaluates published evidence on TMB as a biomarker for efficacy of IO in lung cancers.

**Methods**

Cochrane SLR methodology was followed [1]. Searches were conducted through April 2018 using: Medline; EMBASE; EMCARE; and SCOPUS. Two clinical trial registries (clinicaltrials.gov; ICTRP) and published conference abstracts were also searched. Studies of any design assessing clinical efficacy (objective response rate [ORR], progression-free survival [PFS], overall survival [OS]) in lung cancer (NSCLC/SCLC) by TMB or the association of TMB with other biomarkers or enrichment factors were included.

**Results**

Database searches identified 3662 unique references, full text screening of 809 articles was conducted, and 81 studies met all inclusion criteria. TMB was reported primarily as total mutation count (n=32) or by mutations/megabase (mut/Mb) (n=40). TMB was categorized as low, intermediate, or high in 32 of 81 studies. Methods used to categorize TMB were predetermined thresholds (n=21), the 50th percentile (n=9), or other percentiles (n=2). All studies showed improved PFS (n=8) [2-9] and ORR (n=8) [2-4, 8-12] of IO in TMB high vs. low/intermediate. Significant efficacy results (p<0.05) for IO in TMB high were observed in 6 of 8 studies for PFS and 4 of 8 studies for ORR. Improved OS was observed in 7 of 9 studies with 2 of 9 studies showing significant results [2-4, 10, 12-16]. Results on OS are presented (Table 1) and highlight the small sample size of included studies. TP53 and EGFR mutations were positively and negatively associated with high TMB, respectively [17-19] [19-21]. The literature showed a mixed association of PD-L1 and TMB [2, 7, 22-29]. High TMB was associated with smoking history [7, 19, 30-40], squamous cell carcinoma [18, 33, 41, 42], and male gender [19, 39, 40].

**Conclusions**

This is the first SLR to assess the association of TMB and efficacy in lung cancer. Robust, adequately powered observational and prospective clinical studies should continue to assess TMB and other biomarkers to IO with clinical outcomes. This will validate ongoing data sets and further support precision treatment planning.

**References**

1. Cochrane handbook for systematic reviews of interventions, J.P.T. Higgins and S. Green, Editors. 2011.

2. Carbone DP, et al. First-line nivolumab in stage iv or recurrent non-small-cell lung cancer. N Engl J Med, 2017; 376(25): 2415-2426.

3. Goodman AM, et al. Tumor mutational burden as an independent predictor of response to immunotherapy in diverse cancers. Mol Cancer Ther, 2017; 16(11): 2598-2608.

4. Griesinger F, et al. Tumor mutation burden (TMB) is associated with improved efficacy of atezolizumab in 1l and 2l+ NSCLC patients. Oncology Research and Treatment, 2017; 40((Hellmann M.D.) Memorial Sloan Kettering Cancer Center, New York, USA): 220-221.

5. Hellmann MD, et al. Molecular determinants of response and resistance to anti-pd-(l)1 blockade in patients with NSCLC profiled with targeted next-generation sequencing (ngs). Journal of Clinical Oncology, 2017. 35(15).

6. Hellmann MD, et al., Nivolumab plus ipilimumab in lung cancer with a high tumor mutational burden. N Engl J Med, 2018. 378(22): 2093-2104.7.

7. Mahadevan, N., et al., Non-synonymous mutation burden in lung carcinoma is associated with durable clinical response to immune checkpoint blockade. Journal of Thoracic Oncology, 2017. 12(1): p. S428-S429.

8. Rizvi NA, et al., Cancer immunology. Mutational landscape determines sensitivity to PD-1 blockade in non-small cell lung cancer. Science, 2015. 348(6230): p. 124-8.

9. Roszik J, et al. Novel algorithmic approach predicts tumor mutation load and correlates with immunotherapy clinical outcomes using a defined gene mutation set. BMC Medicine, 2016; 14(1): 168.

10. Gettinger S, et al. Predictive value of measuring somatic mutations and tumor infiltrating lymphocytes for PD-1 axis therapy in non-small cell lung cancer (NSCLC). Journal of Thoracic Oncology, 2017; 12(1): S430-S431.

11. Haratani K, et al. Tumor immune microenvironment and nivolumab efficacy in EGFR mutation-positive non- small-cell lung cancer based on t790m status after disease progression during EGFR-tki treatment. Annals of Oncology. 2017; 28(7): 1532-1539.

12. Singal G, et al. Analyzing biomarkers of cancer immunotherapy (cit) response using a real-world clinico- genomic database. Annals of Oncology. 2017; 28((Abernethy A.) Medical Group, Flatiron Health, Inc., New York, USA): p. v404-v405.

13. Owada Y, et al. Correlation between mutation burden of tumor and immunological/clinical parameters in considering biomarkers of immune checkpoint inhibitors for non-small cell lung cancer (NSCLC). Journal of Clinical Oncology. 2017; 35(15).

14. Rothberg B G, et al. The prognostic impact of EGFR, KRAS and TP53 somatic mutations in curatively resected early-stage lung adenocarcinomas. Journal of Thoracic Oncology. 2017; 12(1): S623.

15. Schrock A B, et al. Pulmonary sarcomatoid carcinomas commonly harbor either potentially targetable genomic alterations or high tumor mutational burden as observed by comprehensive genomic profiling. J Thorac Oncol. 2017; 12(6): 932-942.

16. Chen M. et al. The potential clinical application of comprehensive genomic profiling in targeted therapy and immunotherapy of lung cancer. Journal of Thoracic Oncology. 2017;12(11):S2010.

17. Dong, Z.Y., et al., EGFR mutation correlates with uninflamed phenotype and weak immunogenicity, causing impaired response to PD-1 blockade in non-small cell lung cancer. Oncoimmunology, 2017. 6(11): p. e1356145.

18. Choi, M., et al., Mutation profiles in early-stage lung squamous cell carcinoma with clinical follow-up and correlation with markers of immune function. Annals of Oncology, 2017. 28(1): p. 83-89.

19. Goldberg, M.E., et al., The interaction of PD-L1, TMB, and genomic alterations in NSCLC. Cancer Research, 2017. 77(13).

20. Liu, S.Y., et al., Dual positive PD-L1 and cd8+ til represents a predominant subtype in NSCLC and correlates with augmented immunogenicity. Journal of Thoracic Oncology, 2017. 12(1): p. S237.

21. Liu, X., et al., Molecular biomarker study of programmed death receptor ligand 1 (PD-L1) in korean patients with lung adenocarcinoma [#4213], in AACR 2018 Proceedings: Abstracts 3028-5930. 2018, CTI Meeting Technology: Chicago.

22. Nakagomi, T., et al., New therapeutic targets for pulmonary sarcomatoid carcinomas based on their genomic and phylogenetic profiles. Oncotarget, 2018. 9(12): p. 10635-10649.

23. Schabath, M., et al., Molecular epidemiology of programmed cell death 1-ligand 1 (PD-L1) protein expression in non-small cell lung cancer. Journal of Thoracic Oncology, 2017. 12(1): p. S475-S476.

24. Senarathne, W., et al., Composition of the immune microenvironment differs between carcinomas metastatic to the lungs and primary lung carcinomas. Annals of Diagnostic Pathology, 2018. 33: p. 62-68.

25. Ross, J., et al., Immune checkpoint inhibitor (ICPI) efficacy and resistance detected by comprehensive genomic profiling (CGP) in non-small cell lung cancer (NSCLC) [#1138] Annals of Oncology, 2017. 28(Suppl 5).

26. He, L., et al., Evaluating genomic signatures predicting veliparib sensitivity in non-small cell lung cancer (NSCLC). Journal of Thoracic Oncology, 2017. 12(1): p. S267-S268.

27. Kadara, H., et al., Whole-exome sequencing and immune profiling of early-stage lung adenocarcinoma with fully annotated clinical follow-up. Annals of Oncology, 2017. 28(1): p. 75-82.

28. Kim, H.S. and J.Y. Han, Association of PD-L1 expression with tumor infiltrating immune cells and mutation burden in the high grade neuroendocrine carcinoma of the lung. Journal of Clinical Oncology, 2017. 35(15): p. Suppl 1.

29. Ono, A., et al., Clinical factors associated with mutation burden in non-small cell lung cancer. Annals of Oncology, 2017. 28((Yamaguchi K.) Hospital and Research Institute, Shizuoka Cancer Center, Shizuoka, Japan): p. v578-v579.

30. Quek, K., et al., Mutational landscape of non-small cell lung adjacent normal [#5363], in AACR 2018 Proceedings: Abstracts 3028-5930. 2018, CTI Meeting Technology: Chicago.

31. Reck, M., et al., Smoking history predicts sensitivity to parp inhibitor veliparib in patients with advanced non- small cell lung cancer. J Thorac Oncol, 2017. 12(7): p. 1098-1108.

32. Schrock, A.B., et al., Characterization of 298 patients with lung cancer harboring MET exon 14 skipping alterations. J Thorac Oncol, 2016. 11(9): p. 1493-502.

33. Shim, H.S., et al., Unique genetic and survival characteristics of invasive mucinous adenocarcinoma of the lung. J Thorac Oncol, 2015. 10(8): p. 1156-62.

34. Wang, C., et al., The correlation between mutation burden and disease free survival in patients with lung adenocarcinomas. Journal of Clinical Oncology, 2017. 35(15).

35. Xiao, D., et al., Analysis of ultra-deep targeted sequencing reveals mutation burden is associated with gender and clinical outcome in lung adenocarcinoma. Oncotarget, 2016. 7(16): p. 22857-64.

36. Xiao, D., et al., Integrative analysis of genomic sequencing data reveals higher prevalence of lrp1b mutations in lung adenocarcinoma patients with copd. Sci Rep, 2017. 7(1): p. 2121.

37. Isaka, M., et al., Integrated genomic analysis to assess the molecular signature of Japanese patients with non- small cell lung cancer. Journal of Thoracic Oncology, 2017. 12(11): p. S2292.

38. Kojima, H., et al., Genomic analysis to assess a molecular signature in Japanese patients with pulmonary high grade neuroendocrine carcinoma. Journal of Thoracic Oncology, 2017. 12(11): p. S2186.

39. Davis, A.A., et al., Association of tumor mutational burden (TMB) with DNA repair mutations and response to anti-PD-1/PD-L1 therapy in non-small cell lung cancer (NSCLC). Cancer Research, 2017. 77(13).

40. Park, W., et al., Correlating isend and tumor mutation burden (TMB) with clinical outcomes of advanced non- small cell lung cancer (ansclc) patients on nivolumab. Journal of Thoracic Oncology, 2017. 12(11): p. S2005-S2006.

41. Patel, K., et al., In silico analysis of non-synonymous snps (nsSNPs) and outcomes in non-small cell lung cancer (NSCLC) patients (pts) treated with immunotherapy (it). Journal of Clinical Oncology, 2017; 35(15).

**Table 1 (abstract P137).**
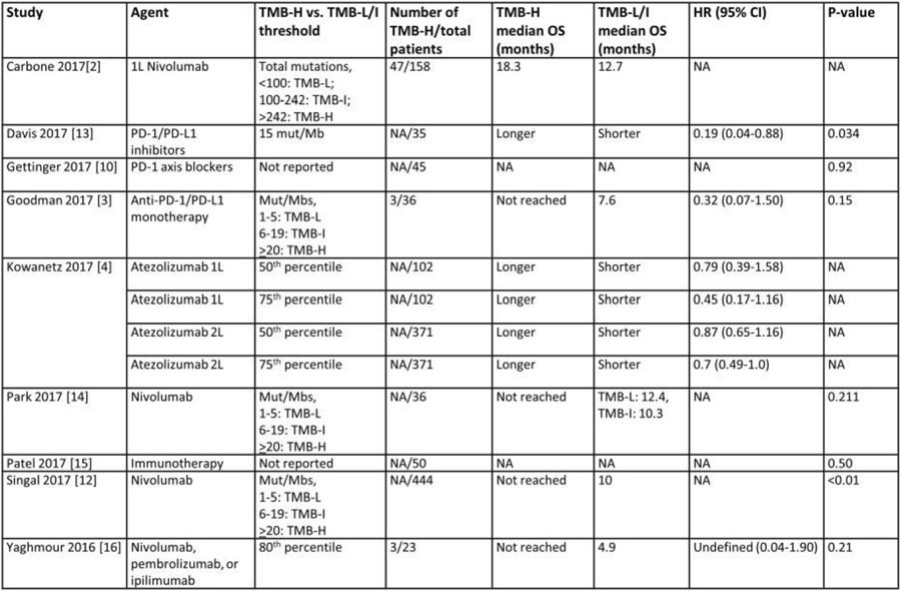
Overall survival with IO by TMB

#### P138 Quantitative assessment of cancer-associated fibroblasts and immunotherapy outcome in metastatic melanoma

##### Pok Fai Wong, MD, MPhil^1^, Wei Wei, MD, PhD^2^, Swati Gupta, PhD^1^, James Smithy, MD, MHS^3^, Harriet Kluger, MD^1^, David L. Rimm, MD, PhD^1^

###### ^1^Yale School of Medicine, New Haven, CT, USA; ^2^Yale School of Public Health, New Haven, CT, USA; ^3^Brigham and Women's Hospital, Boston, MA, USA

####### **Correspondence:** David L. Rimm (david.rimm@yale.edu)

**Background**

Clinical benefit from programmed cell death 1 (PD-1) immune checkpoint blockade is limited to a subset of metastatic melanoma patients, so there is a need for predictive biomarkers. Because the cancer-associated fibroblast (CAF) population is the predominant stromal cell type within the tumor immune microenvironment, we hypothesized that pretreatment CAF profiles could be associated with immunotherapy outcome.

**Methods**

Pretreatment whole tissue sections from 117 melanoma patients treated with anti-PD-1 therapy (pembrolizumab, nivolumab, or ipilimumab plus nivolumab) from 2011–17 were collected from Yale Pathology archives. Multiplex immunofluorescence for CAF profiling was achieved by simultaneous detection of nuclei (DAPI), melanoma cells (S100, HMB45), and the CAF markers, Thy1 (7E1B11, Abcam), smooth muscle actin (SMA; 1A4, Dako), and fibroblast activation protein (FAP; EPR20021, Abcam). Cell phenotyping and counting were performed using inForm software (PerkinElmer) and protein expression was measured by the AQUA method of quantitative immunofluorescence (QIF). CAF parameters by both methodologies were correlated with best overall response as defined by Response Evaluation Criteria in Solid Tumors (RECIST) 1.1, progression-free survival (PFS), overall survival (OS), and immune markers previously measured in this cohort.

**Results**

Pretreatment CAF parameters, by cell counts or QIF, were not associated with RECIST tumor burden classifications for best overall response, objective response rate or disease control rate. In contrast, PFS (all *P* < 0.05) and OS (all *P* < 0.003) had significant positive associations with Thy1 and FAP cell counts, and negative associations with SMA cell count, which were specific to anti-PD-1 treated patients. Similar associations were not observed in a historical untreated melanoma cohort. In the absence of therapy, FAP was instead a negative prognostic biomarker (*P* = 0.01). The specific association of FAP with anti-PD-1 survival advantage suggests mechanistic involvement and warrants further study. Multivariable analyses also revealed statistically significant PFS and OS associations with the CAF parameters, particularly for FAP, independent of age, sex, mutation, stage, treatment, and prior immune checkpoint blockade. The QIF data showed similar trends. There was no correlation between the CAF parameters and CD8 or PD-L1 by either method of assessment.

**Conclusions**

Pretreatment CAF parameters, by cell counts or QIF, are associated with immunotherapy outcome in metastatic melanoma patients. Multiplex analysis of the tumor microenvironment has potential to be used as a companion diagnostic for precision immunotherapy and may be complementary to existing markers (CD8 and PD-L1).

**Ethics Approval**

The study was approved by the Yale Human Investigation Committee protocol #9505008219.

#### P139 Pharmacodynamic effects of CA170, a first-in-class small molecule oral immune checkpoint inhibitor (ICI) dually targeting V-domain Ig suppressor of T-cell activation (VISTA) and PD-L1

##### Timothy Wyant, PhD^1^, Funda Meric-Bernstam, MD^2^, David Tuck^3^, Yung-Jue Bang, MD PhD^4^, Anna Ma, MS^3^, Jeffrey Sosman, MD^5^, Adil Daud, MBBS MD^6^, John Powderly, MD, CPI^7^, Javier Garcia-Corbacho^8^, Manish Patel, MD^9^, James Lee, MD, PhD^10^, Kyu-Pyo Kim^11^, Joshua Brody, MD^12^, Sun Young Rha^13^, Erika Hamilton, MD^14^, Marta Gil Martin^15^, Santiago Ponce Aix, MD^16^, Radhakrishnan Ramchandren, MD^17^, Myung-Ju Ahn^18^, James Spicer, MD, PhD^19^, Simon Pacey^20^, Gerald Falchook, MD^21^, Funda Meric-Bernstam, MD^2^

###### ^1^Curis Inc, Lexington, MA, USA; ^2^MDACC, Houston, TX, USA; ^3^Curis, Lexington, MA, USA; ^4^Seoul National University Hospital, Seoul, Korea; ^5^Northwstern, Chicago, IL, USA; ^6^UCSF, San Francisco, CA, USA; ^7^Carolina BioOncology Institute, Huntersville, NC, USA; ^8^Hospital Clinic Barcelona, Barcelona, Spain; ^9^FLorida Cancer Specialists SCRI, Sarasota, FL, USA; ^10^UPMC, Pittsburgh, PA, USA; ^11^AMC, Seoul, Korea, Republic of; ^12^Mt.Sinai, New York, NY, USA; ^13^Yonsei University Health, Seoul, Korea, Republic of; ^14^Tenn Oncology SCRI, Nashville, TN, USA; ^15^Catalan Institute of Oncology, Catalon, Spain; ^16^Hosptial Univesitario, Madrid, Spain; ^17^Karmanos, Detroit, MI, USA; ^18^Samsung Medical center, Seoul, Korea, Republic of; ^19^King's College Guy's Hospital, London, UK; ^20^University of Cambridge, Cambridge, UK; ^21^SCRI Healthone, Denver, CO, USA

####### **Correspondence:** Simon Pacey (hwwang.2004@gmail.com); Gerald Falchook

**Background**

VISTA was shown to independently suppress T cell responses and is expressed on both immune and tumor cells. VISTA expression is upregulated in tumors as a potential resistance mechanism after ICI therapy. As such, it has been considered a target for cancer immunotherapy. Pre-clinical studies have demonstrated dual blockade of PD-1 and VISTA can be synergistic. CA-170, novel oral dual inhibitor of VISTA and PD-L1/L2 is in phase 1 study with exploratory pharmacodynamic endpoints.

**Methods**

Longitudinal blood samples were collected for PBMC phenotyping. Archival tumor tissue was acquired on all patients and paired tumor biopsies (baseline and C2) were collected when feasible. IHC using select markers of interest and Nanostring immune transcriptome analyses were conducted on paired tumor samples.

**Results**

At the time of this analysis 41 patients had been treated across 6 dose levels (50 – 800 mg QD) with 39 having blood samples available for PBMC phenotyping, 19 patients had sufficient paired tumor biopsies for IHC, and 9 for Nanostring-based analysis. In the 39 patients with available blood samples, within 24 hours after the initial CA-170 dose, 28% showed an increase in the percentage of peripheral blood CD8 T cells that expressed CD69 (median +3.2 fold) and 21% showed an increase in CD8 T cells that expressed CD134+ (median + 2.8 fold). IHC staining analysis of paired tumor biopsies showed increases of CD8+ T cell population in 39% (1.5 fold to 13.8 fold) and CD11b+ myeloid population in 36% of samples (1.5 fold to 5 fold) at cycle 2. Additionally there tended to be increased expression of VISTA in 38% of evaluated samples (1.5 fold to 14.5 fold) post-treatment. Nanostring immune panel transcriptome analysis showed a trend for increased expression of signatures of T-helper (3/9 pairs), myeloid (9/9 pairs), and T-reg cells (3/9 pairs). Activation of IFN-γ gene signature and certain induced genes (CXCL9, CXCL10, CXCL11) showed an increase of at least one transcript ranging from 1.97 to 7.6 fold (7/9 pairs).

**Conclusions**

These data suggest that CA-170 treatment results in modulation of peripheral and intra-tumoral immune profile in treated patients. CA-170 treatment was associated with altered myeloid cell as well as T helper cell populations more frequently than CD8+ T cells. Increased expression of VISTA in tumor biopsies were noted post CA-170 treatment. Correlation between the PD effects and tumor response to CA-170 treatment is being explored.

**Trial Registration**

NCT02812875

**Ethics Approval**

The following institutions ethics boards have approved this study:MD Anderson Cancer Center, Seoul National University Hospital, Northwestern, UCSF, Carolina BioOncology Institute, Hospital Clinic de Barcelona, Florida Cancer Specialists/Sarah Cannon Research Institute, UPMC, Asian Medical Center, Mt. Sinai, Yonsei University Health System - Severance Hospital, Tennessee Oncology/Sarah Cannon Research Institute, Catalan Institute of Oncology, Hospital Universitario 12 de Octubre, Karmanos, Samsung Medical Center, King's College London, Guy's Hospital, University of Cambridge,Sarah Cannon Research Institute at HealthONE

**Consent**

Written informed consent was obtained from the patient for publication of this abstract and any accompanying images. A copy of the written consent is available for review by the Editor of this journal

#### P140 Deep profiling of Asian NSCLC to identify the tumor antigen-specific T cells and the predictive potential of the patients treated with PD-1/PD-L1 blockade

##### Joe Yeong, MBBS, PhD^1^, Lisda Suteja^2^, Yannick Simoni^3^, Kah Weng Lau^4^, Sherlly Lim^4^, Jie Hua, Josh Loh^4^, Angela Takano^4^, Eng Huat Tan, MD^2^, Kiat Hon, Tony Lim^4^, S. W. Daniel Tan^2^, Evan W. Newell, PhD^3^

###### ^1^Singapore Immunology Network/ Singapore General Hospital, Singapore, Singapore; ^2^National Cancer Center Singapore, Singapore, Singapore; ^3^Singapore Immunology Network, Singapore, Singapore; ^4^Singapore General Hospital, Singapore, Singapore

####### **Correspondence:** Tony Lim (lim.kiat.hon@singhealth.com.sg)

**Background**

Although PD-L1 tumor proportion score (TPS)(1), microsatellite instability status (MSI)(2) and interferon gamma (IFN-γ)(3) gene signature have been widely recognized as biomarkers to predict the responsiveness of PD-1/PD-L1 blockade treatment, more clinically robust and practical markers are needed to more precisely identify potential responders and also to warn of potential toxicity especially when combination immunotherapies are used. Asian Non-Small Cell Lung Cancer (NSCLC) are differ from their western counterparts in terms of etiology with low prevalence of smoking and high incidence of EGFR mutant positive adenocarcinoma(4). Following up on our recent study (Nature 2018)(5) that profiled and implicated CD39+CD8+ T cells as tumor antigen-specific T cell populations in both Asian NSCLC and colorectal cancer tumors, here we investigate the tissue localization and predictive potential of CD39+CD8+ T cells in the context of NSCLC. Because CD39 expression by CD8+ T cells in these tumors appears to be associated with tumor-reactivity, we hypothesize that patients with higher frequencies or densities of these cells should respond better to checkpoint blockade immunotherapy.

**Methods**

Quantifying these cells in-situ using formalin-fixed paraffin-embedded (FFPE) archival tissue is difficult due to the often-abundant expression of CD39 by tumor cells or other infiltrating immune cells, which confounds accurate quantification of densities/frequencies of CD39+CD8+ T cells by standard immunohistochemistry. To overcome this challenge, we developed a fully automated multiplex Immunohistochemistry (m-IHC) protocol with clinical autostainer to visualize and quantitate tumor infiltrating lymphocytes (TILs) on FFPE samples (Pathology 2018)(6). In addition to manual scoring by two pathologists, CD39+CD8+ T cells frequencies/densities, TILs and tumor cells expression were assessed using pathological image analysis software.

**Results**

Based on this approach, we found that the quantification of CD39+CD8+ T cells by CyTOF and m-IHC yield decent correlation which lays the foundation to translate this to clinical practice in near future. We also identified that the abundances of such subsets are associated with clinicopathological parameters such as EGFR mutation status. We then applied such quantification method on the pre-treatment FFPE biopsies samples of a pilot retrospective cohort of NSCLC treated with PD-1/PD-L1 blockade (N=20) and found that the abundance of these CD39+CD8+ T cells population are of predictive value. The prediction is independent to the abundance of CD8+ T cells as well as other TILs and CD39 expression on tumor cells.

**Conclusions**

Further study is ongoing to expand the cohort and explore the predictive potential of this biomarker compared to the TPS, MSI and FN-γ gene signature.

**References**

1. Reck M, Rodríguez-Abreu D, Robinson AG, Hui R, Csőszi T, Fülöp A, et al. Pembrolizumab versus Chemotherapy for PD-L1–Positive Non–Small-Cell Lung Cancer. New Engl J Med. 2016;375(19):1823-33.

2. Le DT, Uram JN, Wang H, Bartlett BR, Kemberling H, Eyring AD, et al. PD-1 Blockade in Tumors with Mismatch-Repair Deficiency. New Engl J Med. 2015;372(26):2509-20.

3. Ayers M, Lunceford J, Nebozhyn M, Murphy E, Loboda A, Kaufman DR, et al. IFN-γ–related mRNA profile predicts clinical response to PD-1 blockade. The Journal of Clinical Investigation. 2017;127(8):2930-40.

4. Cheng T-YD, Cramb SM, Baade PD, Youlden DR, Nwogu C, Reid ME. The International Epidemiology of Lung Cancer: Latest Trends, Disparities, and Tumor Characteristics. Journal of Thoracic Oncology. 2016;11(10):1653-71.

5. Simoni Y, Becht E, Fehlings M, Loh CY, Koo S-L, Teng KWW, et al. Bystander CD8+ T cells are abundant and phenotypically distinct in human tumour infiltrates. Nature. 2018;557(7706):575-9.

6. Lim JCT, Yeong JPS, Lim CJ, Ong CCH, Wong SC, Chew VSP, et al. An automated staining protocol for seven- colour immunofluorescence of human tissue sections for diagnostic and prognostic use. Pathology. 2018;50(3):333- 41.

**Ethics Approval**

The Centralized Institutional Review Board of SingHealth provided ethical approval for the use of patient materials in this study (CIRB ref: 2011/411/B)

**Consent**

Written informed consent was obtained from the patient for publication of this abstract and any accompanying images. A copy of the written consent is available for review by the Editor of this journal.

#### P141 Circulating tumor DNA ( ctDNA) a novel biomarker for immunotherapy response in advanced lung cancer

##### Meera Yogarajah, MD^1^, Ebenezer Appah, MD^1^, Katherine Neblett^2^, Clive Morris^2^, Greg Jones^2^, Vincent Plagnol^2^, Paul Walker, MD^1^

###### ^1^East Carolina University, Greenville, NC, USA; ^2^Inivata, Research Triangle Park, NC, USA

####### **Correspondence:** Meera Yogarajah (meera.bhradeev@gmail.com)

**Background**

Circulating tumor DNA (ctDNA) can be predicative of outcomes in lung cancer. Moreover the ctDNA levels can correlate with changes in tumor burden in response to therapy. We assessed the utility of (ctDNA) levels as an early indicator of response to immune-therapy.

**Methods**

Twenty-nine patients with advanced non-small cell lung cancer initiated on immune therapy with anti-PD1/PDL-1 therapy either alone or in combination with platinum-based chemotherapy were enrolled in this prospective trial. Patients had baseline plasma samples collected prior to therapy and serially with the initial 4 cycles of immuno- or chemo-immunotherapy. ctDNA was assessed in plasma by InVisionFirst™ (Inivata) ctDNA NGS assay for detection and quantification of genomic alterations in 36 genes commonly mutated in NSCLC. The early trends of the ctDNA allele fractions were correlated with imaging responses post 4 cycles of therapy and subsequently with interval imaging.

**Results**

Patients included 31% male, 62 years median age, 21% squamous, 72% adeno, 7% others, median cycles 4. Of the 28 patients, 7 were not evaluable at the time of analysis as they only had baseline values or did not have response assessment imaging. Twenty two patients had evaluable imaging assessments which was done on completion of C4 and later at intervals determined by the treating oncologist. Clinical benefit was demonstrated in 16 (55%) patients (complete response (CR, n =4), partial response (PR, n = 5) or stable disease (SD, n = 7)); 6 patients had progressive disease (PD). In patients achieving CR/PR there was no detectable ctDNA at baseline (n=4) or there was complete clearance of ctDNA after completion of 3-4 cycles of immune therapy (n=4), except for 1 patient who demonstrated persistent ctDNA. Follow up imaging demonstrated continued beneficial responses, with most patients who had CR continuing to be in CR and patients who had PR persisting as PR or improving to CR. Patients with stable disease had varying levels of ctDNA with mildly increasing levels in some and stable low levels in others. All 6 patients with PD had detectable ctDNA at progression and some showed increasing levels.

**Conclusions**

Early decrease or clearance of ctDNA during immune therapy was correlated with positive clinical responses.

Absence of detectable ctDNA was indicative of overall a good response and prognosis. Increasing or newly detectable ctDNA was indicative of progressive disease or poor overall outcome. These results suggest a value for validation in an expanded patient cohort.

#### P142 The immunogenomic impact of indoximod on the tumor microenvironment of melanoma patients

##### Jiayi Yu, PhD^1^, Gabriela R. Rossi, PhD^1^, Ravindra Kohle, MD, PhD^2^, David Munn, MD^2^, Yousef Zakharia, MD^3^, Nicholas Vahanian, MD^1^, Eugene Kennedy, MD, FACS^1^, Charles Link, MD^1^

###### ^1^NewLink Genetics, Ames, IA, USA; ^2^Medical College of Georgia, Augusta Univ, Augusta, GA, USA; ^3^University of Iowa, Iowa City, IA, USA

####### **Correspondence:** Gabriela R. Rossi (gabyrossi@icloud.com)

**Background**

The indoleamine 2,3-dioxygenase (IDO) pathway mediates immunosuppressive effects through the metabolism of tryptophan (Trp) to kynurenine (Kyn). This metabolic pathway triggers downstream signaling through the Trp sensors (GCN2 and mTOR) and the Kyn sensor (aryl hydrocarbon receptor, AhR) [1-4]. Indoximod is an orally administered, small-molecule IDO pathway inhibitor that reverses the immunosuppressive effects of low Trp and high Kyn that result from IDO activity. Preclinical studies demonstrate that indoximod has immunostimulatory effects involving three main cell types: CD8+ T cells, Tregs, and DCs. Indoximod increases proliferation of effector T cells, reprograms Tregs into helper T cells, and downregulates IDO expression in DCs. These effects are observed in both the presence and absence of IDO activity [5].

**Methods**

Patients with newly diagnosed unresectable locally advanced or metastatic melanoma in Phase 2 trial (NCT02073123) underwent pre-treatment tumor biopsy followed by a repeat biopsy after cycle 3 of pembrolizumab and indoximod. Fourteen pairs of tumor specimens (6 patients with objective response, and 8 non-responders) underwent RNA sequencing analysis and multiplex immunofluorescence staining to assess the phenotype and functional status of multiple immune populations in the tumor microenvironment (TME), define changes in the tumor genomic profile and gene expression. Baseline samples from the trial were used for predictive biomarker assessment (n= 38).

**Results**

Expression profiling identified up-regulation of multiple immune regulation pathways previously reported following pembrolizumab treatment. Importantly, comparison against published studies suggested immunologic and metabolic changes contributed exclusively by indoximod, including genes suggestive of increasing cytotoxicity and innate immune cell infiltration and activation (CD14, CD33, CD86, GZMM, CD11c, IRF8 among others). Melanoma- related genes were markedly decreased in responding patients compared to non-responding patients. Pro- inflammatory immunologic changes were observed only in the clinical-responder patients, while the non-responder patients showed minimal immunologic response. Consistent with the hypothesized mechanism of action for indoximod [5], IDO1 expression within the TME was downregulated upon treatment, especially in Ki67neg population. Considered as a predictive biomarker, patients with high IDO expression showed higher probability of response to treatment and longer progression-free survival (PFS). This result was independent of the expression levels of PD-L1.

**Conclusions**

The combination of indoximod and pembrolizumab induced multiple immunologic and metabolic changes in the TME. Comparison analysis indicates that some of these changes appear to be contributed exclusively by indoximod. High IDO1 expression at baseline shows correlation with clinical response to treatment.

**Trial Registration**

ClinicalTrials.gov Identifier NCT02073123

**References**

1. McGaha TL, et al. Immunol Rev. 2012;249(1):135-157.

2. Munn DH, et al. Immunity. 2005;22(5):633-642.

3. Metz R, et al. OncoImmunology. 2012;1(9):1460-1468. 4. Opitz CA, et al. Nature. 2011;478(7368):197-203.

5. Brincks EL, et al. Presented at: Annual Meeting of the American Association for Cancer Research (AACR); April 14-18, 2018; Chicago, IL. Abstract 3753.

#### P143 Rational combination of GITR agonism with PD-1 blockade in cancer patients

##### Roberta Zappasodi, PhD^2^, Cynthia Sirard, MD^3^, Yanyun Li, PhD MD^2^, Sadna Budhu, PhD^2^, Moshen Abu-Akeel^2^, Cailian Liu, MD^2^, Xia Yang^2^, Hong Zhong, BS^2^, Walter Newman, PhD^3^, Jingjing Qi^2^, Phillip Wong, PhD^2^, David Schaer^2^, Henry Koon, MD^4^, Vamsidhar Velcheti, MD FACP^5^, Michael Postow, MD^2^, Margaret K. Callahan, MD, PhD^2^, Jedd Wolchok, MD, PhD^2^, Taha Merghoub, PhD^2^

###### ^1^Memorial Sloan Kettering Institute, New York, NY, USA; ^2^MSKCC, New York, NY, USA; ^3^Leap Therapeutics, Walpole, MA, USA; ^4^Case Western Reserve University, Cleveland, OH, USA; ^5^Cleveland Clinic, Pepper Pike, OH, USA

####### **Correspondence:** Taha Merghoub (merghout@mskcc.org)

**Background**

Despite the clinical successes of checkpoint blockade, many patients are refractory to these therapies, highlighting the need of more effective combination programs targeting alternative immune pathways. Engaging the T-cell co- stimulatory receptor glucocorticoid-induced TNFR-related protein (GITR) with agonist antibodies has shown promising activity in preclinical mouse models. Based on this rationale, we initiated the first in-human phase-I trial of GITR stimulation with the agonist antibody TRX518 in advanced solid cancer patients (NCT01239134). Treated patients showed frequent reductions in circulating regulatory T cells (Tregs) that correlated with intra-tumor Treg reductions. However, this was not sufficient to achieve a clinical benefit. Here, we investigate the mechanisms underlying resistance of advanced tumors to GITR agonism and provide the rationale to combine anti-GITR with checkpoint blockade in clinical trials.

**Methods**

Mice were implanted with B16F10 murine melanoma and treated with the anti-GITR DTA-1 alone or in combination with the anti-PD-1 RMP1-14. Composition, phenotype and function of tumor-infiltrating T cells were analyzed by flow cytometry, in vitro killing assays and molecular profiling.

**Results**

We modeled tumor sensitivity and refractoriness to anti-GITR therapy by treating B16F10-bearing mice on day 4 (responsive tumors) or day 7 (refractory tumors) after tumor implantation respectively. We found that intra-tumor Tregs were significantly reduced and effector-T-cell:Treg ratios increased in both responding and refractory tumors. However, time course analyses revealed complete lack of Treg accumulation in day-4-treated responding tumors, suggesting that Tregs may limit T-cell functionality during tumor development. Accordingly, in refractory compared to responding tumors, CD8+ T cells down-regulated activation/memory T-cell markers and up-regulated exhaustion markers. To overcome resistance to anti-GITR, we co-administered anti-PD-1 with the day-7 anti-GITR suboptimal treatment. This combination controlled tumor growth similar to the day-4 curative anti-GITR monotherapy and achieved 50% long-lasting complete responses. These effects were associated with intra-tumor infiltration of highly activated and cytolytic CD8+ T cells. Based on these results and considering the biologic activity and safety profile of TRX518, we have initiated the clinical investigation of TRX518 in combination with anti-PD-1 in patients with advanced solid tumors (NCT02628574). Noteworthy, 3 of the first patients enrolled in this study have manifested clinical responses.

**Conclusions**

These findings indicate that Treg elimination from advanced tumors is not sufficient to activate cytotoxic CD8+ T-cell responses unless the T-cell exhaustion process is concurrently blocked, underscoring the need to combine Treg- inhibiting/depleting immunotherapies with strategies that counteract exhaustion to regress advanced tumors. This study highlights the importance of developing rational, evidence-based combination immunotherapies.

**Ethics Approval**

All mouse procedures were performed in accordance with institutional protocol guidelines at MSKCC. All procedures involving human subjects, human material, or human data, or involving animals were in compliance with the ethical regulations.

#### P144 A fully-automated multiplex fluorescence IHC assay with whole slide multispectral imaging on mouse tissue: phenoptics™ quantitative pathology solutions translational workflow

##### Yi Zheng, PhD, Carla Coltharp, PhD, Ryan Dilworth, PhD, Rachel Schaefer, Linying Liu, Victoria Duckworth, William Kennedy, Darryn Unfricht, PhD, Peter Miller, MS, Milind Rajopadhye, PhD

###### PerkinElmer Inc., Hopkinton, MA, USA

####### **Correspondence:** Milind Rajopadhye (milind.rajopadhye@perkinelmer.com)

**Background**

Developing biomarker research strategies for clinically relevant therapies in immuno-oncology is predicated on the ability to execute fully translational research studies. Phenoptics™ quantitative pathology solutions (QPS) is a comprehensive, end-to-end solution consisting of multiplex fluorescence immunohistochemistry staining along with multispectral imaging and tissue analysis. It provides an effective and quantitative method to reveal multiple biomarkers or cell types and their interaction within tissue on a per-cell and per-tissue-context basis. It enables a deeper understanding of the biology of the tumor microenvironment. Recently, the applications for Phenoptics have been expanded from regional areas of interest in human tissue Immuno-oncology research into whole slide IO tissue research, including animal studies, creating a truly translational platform. Here we describe a robust, fully- automated 7-color Opal staining procedure on Formalin-fixed paraffin-embedded (FFPE) section of mouse breast cancer followed by a multispectral whole slide scan and image analysis.

**Methods**

FFPE samples from mouse breast cancer were immunostained using Opal Polaris™ 7-color Automation IHC Kit on the Leica BOND RX™ automated stainer. Multispectral fluorescence imagery was acquired on a Vectra Polaris® automated imaging system and analyzed with inForm® and MATLAB® software.

**Results**

We’ve applied the fully-automated Opal staining procedure to a mouse breast cancer sample by using the new Opal Polaris 7-Color Whole Slide reagents plus the newly developed Opal Polymer anti-Rabbit HRP Secondary Antibody. Opal multiplexed fluorescence IHC in conjunction with tissue and cell segmentation and phenotyping using inForm image analysis software allowed us to reliably interrogate subsets of T cells, macrophages, tumor cells and proliferating cells. With the ability to spectrally unmix the whole slide image, we are able to analyze and examine spatial relationships between specific phenotypes in a much larger scale, across tissue types for fully translational studies.

**Conclusions**

The fully-automated Opal multiplex fluorescence staining assay and whole slide multispectral imaging that we developed for FFPE tissue research is illustrated here with a mouse breast cancer model, expanding the Phenoptics workflow into animal studies. It allows for unique tumor microenvironment assessment in the whole tissue section that is translational across models. With a comprehensive immune profiling of tumors and better understanding of cellular relationships across the whole slide, this approach provides us with a deeper understanding of the complex interactions of systems biology inherent in multiple models and tissue types in situ in solid tumors.

#### P145 Computational assessment of IDO1 feedback modulation

##### Jain Zhu, PhD^1^, Rebecca Zhu^2^

###### ^1^OmicsHealth LLC, Bethesda, MD, USA; ^2^Winston Churchill High School, Potomac, MD, USA

####### **Correspondence:** Jain Zhu (jian.zhu@omicshealth.com)

**Background**

IDO1 is a cancer-related immunosuppressive gene. With recent unfavorable results in clinical studies of IDO1 inhibitors, it is imperative to address questions such as its interactions with validated oncology targets, its involvement in drug resistance and feasible combination strategies for its inhibitors. With a wide range of experimental data, efficient computational tools are indispensable for managing and exploring the current knowledge space of IDO1.

**Methods**

As of July 2018, there were ~1,500 publications on IDO1 and cancer in PubMed. Abstracts of these articles were downloaded and systemically analyzed. Information on molecular/cellular interactions together with results from preclinical and clinical studies was extracted, standardized and integrated into a graph-based database. A network- oriented querying and visualization system was established to model the interactions between cellular components, pathways and drugs.

**Results**

As many oncology targets, IDO1 is regulated by feedback loops (FBL). Positive (+)FBLs sustain IDO1 expression and activity when it is induced. Negative (-)FBLs restrict IDO1 activity upon its own induction, but rescue it upon inhibition. The computationally generated IDO1 feedback network (Figure 1) comprises 100 nodes (protein, miRNA, pathway, immune cell etc.), 227 interactions (induction, suppression, activation, inhibition etc.) with 1981 possible FBLs (969 positive vs 1012 negative). Genes involved in (+)FBLs include AKT, NF-kappaB, PD-L1, IL- 10, HIF1A, IL-6, AhR, TIM-3 and STAT3. Modulation of these genes by IDO1 further enhances IDO1 activity. Kynurenine, produced by IDO1 and a ligand for AhR, is implicated in the (+)FBLs. Immune cells including MDSCs, TILs, Tregs and macrophages are also identified with (+)FBLs. On the other hand, IFN-gamma, TP53, p21, STAT1, along with effectorT, CD8+T and NK cells are predominantly involved in (-)FBLs. Mechanistically, IDO1 suppresses activities of effectorT, NK and CD8+T cells with reduction in IFN-gamma leading to a decrease in IDO1 expression. Some gene products are bi-functional, associated with both types of feedbacks. For example, IL6, being up-regulated by IDO1, induces IDO1 transcription through activation of STAT3 (IDO1->IL-6->STAT3->IDO1). Paradoxically, IL-6 up-regulates SOCS3 which promotes degradation of phosphorylated IDO1 (IDO1->IL- 6->SOCS3-|IDO1). The feedback network is further refined with key factors identified (Figure 2).

**Conclusions**

The cellular components involved in the FBLs are potential targets of drug combination with IDO1 inhibitors and biomarkers for treatment outcome. However, the roles of bi-functional genes need to be carefully evaluated. To provide a more complete picture of IDO1 in immune regulation, the feedback models need be constantly refined with newer data and expanded by incorporating data of other drug targets.

**Fig. 1 (abstract P145).**
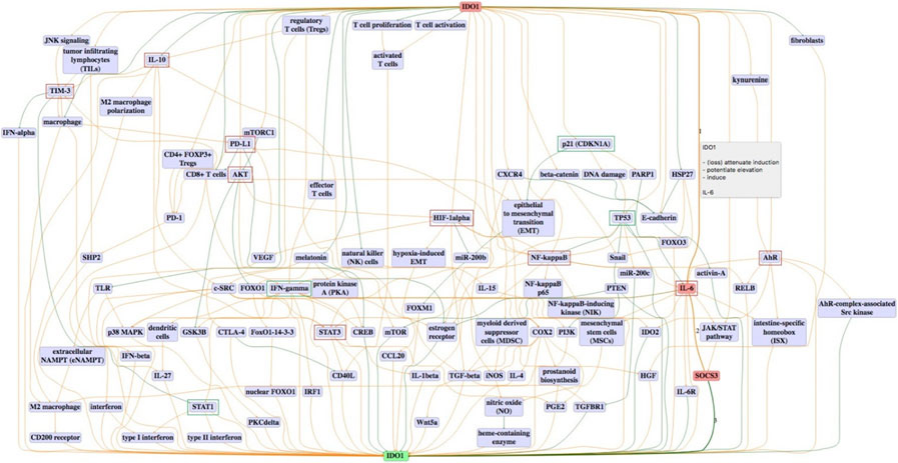
IDO1 feedback regulation network

**Fig. 2 (abstract P145).**
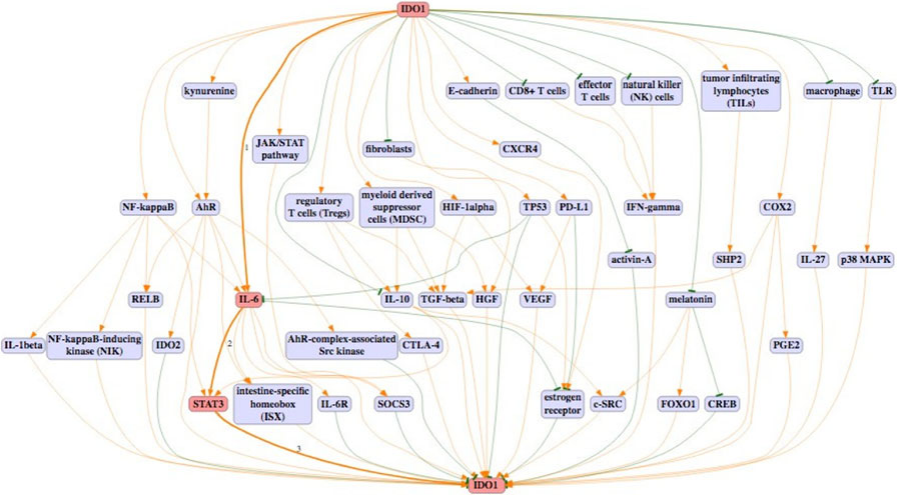
Key factors in IDO1 feedback regulation

#### P146 Cell proliferation improves prediction for immune checkpoint inhibitors (ICIs) response in PD-L1 positive TMB high non-small cell lung cancer (NSCLC)

##### Jason Zhu, MD^1^, Matthew Labriola, MD^1^, Daniele Marin, MD^1^, Shannon McCall, MD^1^, Edwin Yau, MD, PhD^2^, Grace Dy^2^, Sarabjot Pabla, MSc, PhD, BS^3^, Sean Glenn, PhD^3^, Carl Morrison, MD, DVM^3^, Daniel George, MD^1^, Tian Zhang, MD^1^, Jeffrey Clarke, MD^1^

###### ^1^Duke University, Durham, NC, USA; ^2^University at Buffalo, Buffalo, NY, USA; ^3^OmniSeq Inc., Buffalo, NY, USA

####### **Correspondence:** Tian Zhang (tian.zhang2@duke.edu)

**Background**

Treatment for metastatic NSCLC is now highly dependent upon ICIs. Although some patients have durable treatment responses, the majority of patients will have either primary resistance or acquired resistance to treatment. While PD-L1 and tumor mutational burden (TMB) status has demonstrated predictive value for multiple ICIs, both are imperfect biomarkers. More accurate biomarkers are necessary to predict treatment response and resistance in metastatic NSCLC. Here, we describe the use of cell proliferation to evaluate response in PD-L1+ TMB high NSCLC.

**Methods**

113 formalin-fixed, paraffin-embedded (FFPE) tumor samples of metastatic NSCLC were evaluated by RNA-seq to measure transcript levels of genes related to cell proliferation, DNA-seq of 409 genes for tumor mutational burden (TMB), and PD-L1 status (Dako 22C3 antibody assay). Tumors were defined as PD-L1+ with a tumor proportion score >50% and as TMB high with 10 or greater mutations per megabase of DNA. Cell proliferation, defined as the mRNA expression of either 10 genes (BUB1, CCNB2, CDK1, CDKN3, FOXM1, KIAA0101, MAD2L1, MELK, MKI67, TOP2A) was evaluated for association with PD-L1 IHC expression, TMB, and response to ICIs by RECIST 1.1 criteria.

**Results**

Among the cohort of 113 cases, 14 (12%) were PD-L1 positive and TMB high, with 7 responders and 7 non-responders (50% objective response rate [ORR]). Of these 14 cases, 7 were proliferative and 7 were non- proliferative. The ORR for proliferative tumors was 29% (2/7) and ORR for non-proliferative tumors was 71% (5/7). Tumor proliferation status therefore improves upon clinical prediction for response to ICIs, even within the PD-L1 positive/TMB high population.

**Conclusions**

The 50% ORR in PD-L1+ TMB high NSCLC (14 cases) was within the expected range. Using proliferation status, non-proliferative tumors had ORR of 71%. This evidence would support that cell proliferation can be used for additional stratification of PD-L1+ TMB high NSCLC.

**Ethics Approval**

OmniSeq’s analysis utilized deidentified data that qualified as non-human subject research under IRB-approved protocols, approved by both Roswell Park Comprehensive Cancer Center (Buffalo, NY, BDR #080316) and Duke Cancer Institute (Durham, NC, PRO00088762).

#### P147 Quantitative assessment of co-expression of PD-L1 and CMTM6 in the tumor microenvironment in non- small cell lung cancer (NSCLC) patients treated with PD-1 pathway blockade

##### Jon Zugazagoitia, MD, PhD^2^, Fahad Shabbir, MD^2^, Yuting Liu, PhD candidate^2^, Brian Henick, MD^2^, Scott Gettinger, MD^2^, Roy Herbst, MD, PhD^2^, Kurt Schalper, MD, PhD^2^, David l. Rimm, MD, PhD^1^

###### ^1^Yale School o Medicine, New Haven, USA; ^2^Yale School of Medicine, New Haven, USA

####### **Correspondence:** David l. Rimm (david.rimm@yale.edu)

**Background**

The importance of tumor versus immune cell PD-L1 expression in predicting benefit to PD-1 pathway blockade in NSCLC, and the role of PD-L1 regulators as potential contributors to these outcomes are poorly understood.

Mechanistic studies have suggested that CMTM6 is a main regulator of PD-L1 expression by preventing its lysosomal degradation. Here we assess CMTM6 co-localized with PD-L1 in tumor and immune cells as a predictive biomarker.

**Methods**

We used multiplexed immunofluorescence (IF) to quantify the expression of PD-L1 and CMTM6 in 73 pre-treatment NSCLC cases represented in tissue microarrays, 56 of whom received only monotherapy. We performed target measurement with a Tyramide-based IF panel (PD-L1/CMTM6/CD68/Cytokeratin[CK]) and analyzed the data using the PM2000 microscope and AQUA software. Targets were measured in CK+ tumor cells, CD68+ macrophages and the non-CK stromal compartment, then split by the median.

**Results**

PD-L1 and CMTM6 showed a modest association with each other, particularly in the stroma (R2 = 0.46) and CD68 (R2 = 0.40) compartments. In the monotherapy group, median OS was numerically longer, but not statistically significant, for patients with high PD-L1 in tumor cell, stroma or CD68 as compared to low PD-L1 tumors. However, OS was significantly longer for patients with concurrent high PD-L1 and high CMTM6 expression as compared to the remaining cases in stroma (23m vs. 6m, p = 0.02) and CD68 (22m vs. 6m, p = 0.03), but NOT in the tumor cell compartment (22m vs. 12m, p = 0.15). In contrast, OS was significantly shorter for patients with high PD-L1 but low CMTM6 expression in stroma (3m vs. 14m, p = 0.02) or CD68 (6m vs. 15m, p = 0.02), but not in the tumor cell compartment (21m vs. 12m, p = 0.80). Patients with low PD-L1 tumors derived no significant benefits in survival, regardless of CMTM6 status.

**Conclusions**

This works supports the hypothesized role for CMTM6 in stabilization of PD-L1 in patient tumors where co-expression in macrophages appears to be associated with benefit from PD-1 pathway blockade. Larger validation datasets are required to confirm this observation.

**Ethics Approval**

The study was approved by the Yale Human Investigation Committee protocol #9505008219.

### Cancer Vaccines, Personal Vaccines and Tech

#### P148 Viral based vaccine for personalized neoantigen-directed cancer therapies

##### Christian Ottensmeier, MD PhD FRCP^1^, Natalia Savelyeva^1^, Katy McKann^1^, Chuan Wang^1^, Jason Greenbaum^2^, Finn Nielsen^3^, Chantal Hoffmann^4^, Huguette Schultz^4^, Nathalie Silvestre^4^, Jean-Baptiste Marchand, pHD^4^, Maud Brandely-Talbot, MD, PhD^4^, Eric Quemeneur, PharmD, PhD^4^, Kaidre Bendjama^4^

###### ^1^University of Southampton, Southampton, UK; ^2^La Jolla Institute for allergy and immun, La Jolla, CA, USA; ^3^Rigshospitalet, Copenhagen, UK; ^4^Transgene, Illkirch Graffenstaden, France

####### **Correspondence:** Kaidre Bendjama (bendjama@transgene.fr)

**Background**

Anarchic cellular proliferation and deficient DNA repair mechanisms result in accumulation of mutations in cancer cells potentially leading to expression of tumor specific neoantigens (TSNA). Given their ad hoc onset in the tumor, TSNA are not subject central tolerance and may constitute ideal targets for therapeutic cancer vaccines. However, clinical implementation of TSNA directed vaccination requires a potent immunizing vaccine formulation allowing the reproducible generation of a bespoke vaccine for every patient within acceptable time and cost. Herein, we propose a workflow to meet both requirements using a modified vaccinia Ankara (MVA) based vaccine. Technical feasibility was shown, and immunogenicity of the vaccine demonstrated in HLA-A02 expressing mice.

**Methods**

Blood and Tumor tissue from a patient with adenocarcinoma of the lung was sequenced and compared to reference genome to identify 2218 variants. Variants were filtered for their expression at transcriptomic level in tumor tissue but not in normal tissue (blood) to select 18 variants (17 missenses and 1 frameshift variant) in 18 different genes. Consequently, 18 mutated sequences covering the mutations and flanking sequences were cloned in an MVA virus. This viral vaccine was generated using a process of less than 8 weeks and compatible with GMP requirements. The said viral vaccine was used to immunize HHD mice and CD4 and CD8 cellular responses against the target neoantigens were assessed by ELISPOT.

**Results**

Responses were observed in all vaccinated animals for 4 mutations while 3 other mutations led to sporadic responses. Stimulation with wild type (non-mutated) human peptides did not evidence any cross reactivity with the wild type proteins. The responses appeared to be of both CD4 and CD8 T-cell nature. Interestingly, 2 out of the 4 strongest responses observed in mice were detected in PBMC from the donor patient without immunization suggesting that these mutations are relevant neoantigens and that vaccination would boost these responses.

**Conclusions**

We propose here a new approach for patient specific neoantigenic vaccination based on a platform with a remarkable safety track record in clinical setting. We have previously shown that successful antitumor immunization using an MVA viral vaccine against a shared antigen was related to an improved clinical outcome. Targeting neoantigens lacking self-tolerance is therefore a very promising approach, we are intending to translate to the clinic as early as 2019. Plans for clinical translation and study designs will be presented.

#### P149 A phase I study of the safety and immunogenicity of a multi-peptide personalized genomic vaccine in the adjuvant treatment of solid tumors and hematological malignancies

##### Ana Blazquez, PhD, Alexander Rubinsteyn, PhD, Julia Kodysh, MSc, John Finnigan, Thomas Marron, MD PhD, Rachel Sabado, PhD, Marcia Meseck, MS, JD, Timothy O'Donnell, Jeffrey Hammerbacher, BA, Michael Donovan, John Holt, Millind Mahajan, John Mandeli, PhD, Krysztof Misiukiewicz, Eric Genden, Brett Miles, DDS MD, Hooman Khorasani, Peter Dottino, Hanna Irie, Amy Tiersten, MD, Elisa Port, Andrea Wolf, MD, Hearn Cho, MD, PhD, Ashutosh Tewari, MD, Samir Parekh, MD, Sujit Nair, Matthew Galsky, MD, William Oh, MD, Sacha Gnjatic, PhD, Eric Schadt, Philip Friedlander, MD PhD, Nina Bhardwaj, MD, PhD

###### Mount Sinai, New York, NY, USA

####### **Correspondence:** Nina Bhardwaj (nina.bhardwaj@mssm.edu)

**Background**

Mutation-derived tumor antigens (MTAs) arise as a direct result of somatic variations that occur during carcinogenesis and can be characterized via genetic sequencing. We developed a platform for a fully-personalized MTA-based vaccine in the adjuvant treatment of solid and hematological malignancies.

**Methods**

This is a single-arm, open label, proof-of-concept phase I study designed to test the safety and immunogenicity of Personalized Genomic Vaccine 001 (PGV001) that targets up to 10 predicted personal tumor neoantigens based on patient’s HLA profile (ClinicalTrials.gov: NCT02721043).

**Results**

Five subjects have been enrolled. PGV001_002 (head and neck squamous cell cancer), who has completed vaccination, received 10 doses of vaccine comprising 10 long peptides (LP) combined with poly-ICLC (toll-like receptor-3 agonist) intracutaneously. Vaccine-induced T-cell responses were determined, at weeks 0 and 27 (before and after treatment, respectively), ex vivo by interferon (IFN)-g enzyme-linked immunospot assay and after expansion by intracellular cytokine staining. Overlapping 15-mer peptides (OLPs) spanning the entirety of each LP and 9-10-mer peptides corresponding to each predicted class I epitope (Min) were pooled. Ex vivo responses to these peptide pools were undetectable at week 0 but were evident at week 27 against 2 OLPs out of 10 (20%) and in 5 Min out of 10 (50%). After in vitro expansion, neoantigen-specific CD4+ and CD8+ T-cell responses were found in 5 out of 10 pooled peptides (50%). 7 out of 10 (70%) epitopes elicited polyfunctional T-cell responses (secretion of INF-γ, TNF-α, and/or IL-2) from either CD4+ or CD8+ T cells.Deconvolution studies showed ex vivo IFN-γ production detection in 1 (15-mer) peptide out of 15 (6.7%) and in 4 (9-mer) peptides out of 22 (18.2%). After expansion, of 22 peptides tested, CD8+ T-cells were reactive against 13 peptides (59%), while CD4+ responses were found in 11 of 15 peptides tested. Both CD4+ and CD8+ T-cell responses were polyfunctional.

**Conclusions**

The PGV001 vaccine in our first patient showed both safety and immunogenicity, eliciting CD4+ and CD8+ responses to the vaccine peptides. As we enroll additional patients in this clinical trial, and perform deeper phenotyping of their tumor-reactive T cells, we will learn the determinants necessary for successful induction of immunity, while informing future immunotherapeutic approaches and rational combinations

**Trial Registration**

NCT02721043

**Ethics Approval**

The study was approved by the Icahn School of Medicine at Mount Sinai’s Institutional Review Board, Approval Number HSM 15-00841

#### P150 A Phase II open labeled, randomized study of poly-ICLC matured dendritic cells for NY-ESO-1 and Mean-A peptide vaccination compared to Montanide, in melanoma patients in complete clinical remission

##### Anna Pavlick, MD, MBA^1^, Ana Blazquez, PhD^2^, Marcia Meseck, MS, JD^2^, Michael Donovan^2^, Mireia Castillo- Martin^2^, Tin Htwe Thin, PhD^2^, Rachel Sabado, PhD^2^, John Mandeli, PhD^2^, Sacha Gnjatic, PhD^2^, Philip Friedlander, MD PhD^2^, Nina Bhardwaj, MD, PhD^2^

###### ^1^NYU University, New York, NY, USA; ^2^Mount Sinai, New York, NY, USA

####### **Correspondence:** Nina Bhardwaj (nina.bhardwaj@mssm.edu)

**Background**

Dendritic cells (DC) play a critical role in tumor immune-surveillance. Combination therapies by utilizing check point inhibitors may revert tumor-induced-T cell exhaustion; however, DCs are necessary to prime/activate T cells to target tumor cells. Montanide is a mineral oil-based adjuvant that enhances the immune response to vaccination. In this study, we compared the immunogenicity of Montanide and poly-ICLC-matured DCs.

**Methods**

This is a Phase II open label, randomized two arm study to compare Poly-ICLC matured DC with systemic administration of Poly-ICLC on days 1 and 2 (ARM A) to Montanide ISA-51 and Poly-ICLC as adjuvants for NY- ESO-1 and Melan-A/MART-1 peptide vaccination with systemic administration of Poly-ICLC on day 2 (ARM B) in study subjects with melanoma in complete clinical remission but at high risk of disease recurrence (NCT02334735). Evaluation of primary tumor expression of NY-ESO-1 and Melan-A tumor was determined by immunohistochemistry (IHC). Humoral responses were assessed by Seromics (ELISA) and T-cell responses were performed ex-vivo by interferon (IFN)-g enzyme-linked immunospot assay (ELISPOT) and after expansion by intracellular cytokine staining (ICS).

**Results**

Twenty-nine patients have been enrolled in this study. IHC studies demonstrated tumor expression of NY-ESO-1 and Melan-A in 78% and 81% of the patients, respectively. 100% of patients within arm B became seropositive for NY-ESO-1 peptide by cycle 2 day 8 (C2D8). 80% of patients within arm A also seroconverted to this antigen but titers were significantly lower. Melan-A-specific antibody responses were also found in arm B patients, but to a lesser degree. However, arm A patients failed to develop seroreactivity to Melan-A. Cellular responses are under analysis. Preliminary data show that subjects in both arms develop T cell responses to both antigens.

**Conclusions**

This vaccine trial reached the primary endpoint of safety and tolerability. Patients vaccinated with either DC or Montanide had demonstrable antibody titers to immunizing antigens, although the latter reproducibly induced higher titers. Evaluation of cellular responses is ongoing.

**Trial Registration**

NCT02334735

**Ethics Approval**

The study was approved by the Icahn School of Medicine at Mount Sinai’s Institutional Review Board, Approval Number HSM 14-00821

#### P151 Improving neoantigen identification for therapeutic and diagnostic use in immuno-oncology using mass spectrometry and machine learning

##### Sean Boyle, PhD, Eric Levy, PhD, Gabor Bartha, Jason Harris, Rena McClory, John S. West, MBA, Richard Chen

###### Personalis, Menlo Park, CA, USA

####### **Correspondence:** Sean Boyle (Sean.boyle@personalis.com)

**Background**

Neoantigens are increasingly critical in immuno-oncology as therapeutic targets for neoantigen-based personalized cancer vaccines (PCVs) and as potential biomarkers for immunotherapy response. However, the methods for identifying which neoepitopes are more likely to provoke an immune response remains an important challenge for improving both the effectiveness of PCVs and enabling the potential use of neoantigens as a biomarker in immunotherapy. Current MHC binding prediction algorithms are trained using in vitro MHC binding data. One shortcoming of this approach is data generated using this methodology does not consider important upstream processing and shuttling components which occur for natural neoantigen presentation. Recent advances in immuno-affinity purification and mass spec technology makes it possible to identify processed cell surface MHC bound peptides in an in vivo setting, providing the opportunity for development of improved neoantigen prediction pipelines.

**Methods**

We sought to enhance the prediction of neoepitope binding \by improving both the training data and predictive algorithms. Mono-allelic HLA class I cell lines were generated by transfecting individual class I HLA alleles into the HLA class I null cell line K562, prioritizing HLA class I alleles that were of high abundance in different populations. Cell surface bound MHC class I peptides were then identified through immuno-affinity purification followed by mass spectrometry. We then developed and trained neural networks to predict MHC class I presentation for each assayed HLA class I allele. The predictive algorithms were incorporated into our overall neoantigen pipeline (NeoantigenID), which integrates DNA and RNA based somatic variant calling, somatic HLA mutation calling, peptide phasing, identification of indel and fusion derived neoepitopes, and comprehensive immuno- genomics biomarker reporting.

**Results**

We applied paired transfection and mass spec in an allele specific manner to identify thousands of MHC bound peptides bound to dozens of HLA class I alleles. Our neoantigen prediction algorithm, trained on our own in vivo peptide data, consistently achieves a higher overall sensitivity and specificity than other tools based primarily on in vitro MHC binding data, when tested on the same HLA class I alleles. Our MHC class I-epitope binding prediction algorithm demonstrated an aggregative precision value of 0.88 across HLA alleles, as opposed to 0.50 for other widely used tools.

**Conclusions**

Effective neoantigen identification requires an accurate variant detection and characterization pipeline built upon a comprehensive exome and transcriptome platform. We have developed an accurate solution for detection of neoantigens with emphasis on improved MHC presentation prediction from a broad genomic footprint.

#### P152 Nanofluidic drug-eluting seed for sustained intratumoral immunotherapy for cancer treatment

##### Ying Xuan Chua, PhD^1^, Antonia Susnjar^1^, Jeremy Ho^1^, Jessica Rhudy^1^, Priya Jain, BS^1^, Marco Folci^1^, Andrea Ballerini^1^, Shailbala Singh^2^, Carly Filgueira, PhD^1^, Cassian Yee, MD^2^, Brian Butler^1^, Alessandro Grattoni^1^

###### ^1^Houston Methodist Research Institute, Houston, TX, USA; ^2^UT MD Anderson Cancer Center, Houston, TX, USA

####### **Correspondence:** Ying Xuan Chua; Alessandro Grattoni (agrattoni@houstonmethodist.org)

**Background**

While the clinical breakthrough of immune checkpoint inhibitors such as anti-programmed death-1 (PD-1) or cytotoxic T-lymphocyte-associated protein 4 (CTLA-4) in certain cancers has fueled optimism in immunotherapy, the efficacy is limited to a small subset of patients. In addition to insufficient anti-tumor immune response, systemic immunotherapy administration causes adverse side effects, whereby up to 91% of treated patients experienced clinically significant immune-related adverse events (irAEs). Intratumoral immunotherapy delivery has demonstrated anti-tumor efficacy in preclinical and clinical studies with minimal systemic toxicity. However despite encouraging results, the feasibility of intratumoral injections is restricted to accessible solid tumors whereas inaccessible tumors require technically challenging image-guided injections.

**Methods**

To fulfill the unmet need for sustained local drug delivery and to avoid repeated intratumoral injections, we developed the nanofluidic drug-eluting seed (NDES), a device the size of a grain of rice for intratumoral drug delivery. The NDES is inserted intratumorally using a minimally invasive trocar method similar to brachytherapy seed insertion and offers a clinical advantage of drug elution. Drug release from the NDES is driven by a difference in concentration, allowing for sustained immunotherapy delivery without the need for injections, actuation or clinician intervention. In this study, the NDES was used to deliver immunotherapeutics intratumorally in the 4T1 orthotopic murine mammary carcinoma model, which recapitulates triple negative breast cancer (Figure 1A).

**Results**

We demonstrated that NDES-mediated intratumoral release of agonist monoclonal antibodies, OX40 and CD40, resulted in potentiation of local and systemic anti-tumor immune response and inhibition of tumor growth compared to control. Further, mice treated with NDES-CD40 showed minimal systemic drug exposure and liver damage compared to systemically treated mice. As CD40 monotherapy was insufficient to eliminate tumor burden, we speculated that therapeutic synergy could be maximally achieved by combination treatment with radiation to induce immunogenic cell death. In line with this, NDES-CD40 in combination with localized radiation was more effective at reducing tumor burden than monotherapy (Figure 1B).

**Conclusions**

Overall we present the NDES as an effective platform for sustained intratumoral immunotherapy delivery with tremendous potential for clinical translation given that the NDES is applicable to a broad spectrum of drugs and solid tumors.

**Acknowledgements**

We thank Carlos Favela and Dr. Kemi Cui from the advanced cellular and tissue microscopy core, Dr. Jianhua (James) Gu from the electron microscopy core, Dr. Andreana L. Rivera, Dr. Yulan Ren, and Sandra Steptoe from the research pathology core of Houston Methodist Research Institute. This work was supported by Golfers Against Cancer, Nancy Owens Breast Cancer Foundation and start-up funds from Houston Methodist Research Institute (AG). Nanofluidic membranes were provided by NanoMedical Systems, Inc.

**Ethics Approval**

All animal experiments conducted were approved by the Institutional Animal Care and Use Committee (IACUC) of Houston Methodist Research Institute and performed in accordance with the National Institute of Health Guide for the Care and Use of Laboratory Animals and the Animal Welfare Act.

#### P153 Vaccination with HSP70iQ435 DNA elicits a broad, functional antibody response that may contribute to tumor control of melanoma

##### Cormac Cosgrove, PhD, Caroline Le Poole, Emilia Dellacecca, Steven Henning

###### Northwestern University, Chicago, USA

####### **Correspondence:** Caroline Le Poole (caroline.lepoole@northwestern.edu)

**Background**

On target, off tumor side effects during cancer immunotherapy remain a major concern. Vitiligo is an autoimmune disease resulting in severe skin depigmentation and often occurs in melanoma patients receiving immunotherapy. Importantly, cytotoxic T cells recognize the same antigens in both conditions, drive depigmentation in vitiligo and are the target of immunotherapies in melanoma treatment. Inducible HSP70 activates dendritic cells (DCs), mediates autoimmune reactivity in vitiligo and is overexpressed in melanomas. However, vaccination with a mutated form of HSP70i (HSP70iQ435A), developed in our lab, tolerizes DCs, prevents depigmentation and elicits tumor control in mouse models, pointing to a separation of tumor immunity and autoimmune responses, testable in Sinclair swine with melanoma and vitiligo. Experiments in CD8 knockout mice and B cell adoptive transfer experiments suggest that that beyond T cells, there is a key role for B cell or antibody-mediated tumor control in HSP70iQ435A DNA vaccinated mice.

**Methods**

C57BL/6 mice were vaccinated abdominally 5Q6days with either HSP70iQ435A or empty vector DNA using the Helios Gene Gun platform (Biorad). Serum was collected by cardiac bleed at euthanasia. ELISA was used to determine antibody isotype/subclass titers in serum and anti-HSP70-mediated ADCC was measured in an ELISA-based NK cell degranulation assay. Sinclair swine received 4 weekly doses of HSP70iQ435A plasmid DNA by jet injection directly to the perilesional border. Changes in lesion and melanoma size were tracked over 6 months.

**Results**

Significant anti-HSP70i IgG and IgM titers were present in HSP70iQ435A versus empty vector DNA-vaccinated mice. Antibodies titers were observed for IgG1, 2a, 2b, 2c, 3, and 4 subclasses and there was significant degranulation of NK cells in response to anti-HSP70 serum antibodies. In Sinclair swine, repigmentation of lesions was observed in HSP70iQ435A DNA-treated but not PBS-treated animals. Further, while the presence of vitiligo was associated with tumor shrinkage, melanomas in HSP70iQ435A DNA-treated sites showed a trend towards accelerated shrinkage compared to vitiligo alone.

**Conclusions**

Vaccination with HSP70iQ435A DNA elicits a broad antibody isotype and IgG subclass response, capable of activating NK cells to degranulate. Coupled with the overexpression of HSP70i by melanomas, antibodies therefore may contribute to tumor control through ADCC. Furthermore, experiments in Sinclair swine suggest that local vaccination can also reverse vitiligo without impacting tumor control. Thus, anti-HSP70i antibodies may support tumor control during repigmentation in response to HSP70iQ435A DNA vaccination. These data suggest a potentially important role for active anti-HSP70i vaccination to support tumor control.

**Ethics Approval**

The Ethics Boards at Loyola University, Chicago, and Northwestern University at Chicago approved the study.

#### P154 Empiric profiling of neoantigen-specific T cell responses in NSCLC patients with ATLAS™ reveals unexpected neoantigen and inhibitory antigen profiles

##### Kyle Ferber, PhD, BS^1^, Michael O'Keeffe^1^, Crystal Cabral, BS, MSc^1^, Christopher Warren^1^, Erick Donis^1^, Mariya Croll, MS^1^, James Loizeaux^1^, Melissa W. Hayes, PhD^1^, James Perry^1^, Wendy Broom, PhD^1^, Pamela Carroll^1^, Jessica Flechtner, PhD^2^, Jason Dobson, PhD, BS^1^

###### ^1^Genocea Biosciences, Cambridge, MA, USA; ^2^Genocea Biosciences, Inc., Cambridge, MA, USA

####### **Correspondence:** Jessica Flechtner (jessica.flechtner@genocea.com)

**Background**

Immunotherapy of non-small cell lung cancer has resulted in unprecedented, but sometimes short-lived efficacy in first- and second-line settings. The importance of T cells recognizing patient-specific mutations, or neoantigens, in successful immune checkpoint blockade (ICB) treatment is well established. Given the role of neoantigens in successful ICB treatment, coupled with the aspiration to improve response rates to ICB in the absence of added toxicity, efforts are underway to develop neoantigen vaccines to enhance specific T cell responses. The challenge is identifying which mutation, out of the tens to thousands present in a patient’s tumor, to include in their vaccine. Here we comprehensively identify neoantigen-specific T cell responses in the peripheral blood of NSCLC patients to identify their characteristics.

**Methods**

The ex vivo ATLAS™ technology enables comprehensive screening of a subject’s tumor mutanome by using subject-specific T cells and monocyte-derived dendritic cells (MDDCs). PBMC were isolated from nine consented NSCLC patients and viably frozen. Whole exome sequencing of paired tumor and saliva samples from the same subjects was performed. CD8 and CD4 libraries of putative antigens were constructed by cloning and expressing regions spanning each somatic mutation in E. coli ± a co- cytoplasmic variant of listeriolysin O. Unique libraries were pulsed in an ordered array onto MDDCs from each patient, and then their CD8+ and/or CD4+ T cells added and incubated overnight. Recall responses were measured by detection of cytokines in the supernatants.

**Results**

Subject tumors had medians of 700 somatic mutations with 100 unique sequences. More than 1,000 mutations were screened and both stimulatory and inhibitory CD4+ and CD8+ T cell responses to neoantigens, which were rarely shared between subsets, were identified. Inhibitory neoantigens made up a greater proportion overall. Up to 66% of neoantigens were not expressed (by RNAseq). Most of the identified neoantigens were not algorithm-predicted epitopes, nor could they be classified based on expression level or mutation type. T cell responses to driver mutations were infrequent. No obvious associations between neoantigen frequency and tumor mutational burden were identified. The impact of ICB on neoantigen response profiles will be discussed.

**Conclusions**

The ex vivo ATLAS platform can be used to identify and characterize neoantigens in the peripheral blood of oncology patients. Identified neoantigens are unexpected relative to epitope prediction algorithms and common neoantigen prioritization criteria. A Phase 1/2a clinical trial of a targeted personalized cancer vaccine, GEN-009, using ATLAS-identified antigens is ongoing.

**Acknowledgements**

US Oncology

**Ethics Approval**

This study was approved by US Oncology, Inc., Institutional Review Board; Committee #1- Dallas

#### P155 A DNA-based CSPG4 cancer vaccine designed by SynCon® technology is highly immunogenic and extends survival in a mouse model of melanoma

##### Bradley Garman, MS^1^, Jewell N. Walters, BS PhD^1^, Elizabeth K. Duperret, BS, PhD^2^, Aspen Trautz^2^, Jaemi Chu^2^, Jian Yan, BS PhD^1^, Laurent Humeau, BS, MS, PhD^1^, David B. Weiner BS, MS, PhD^2^

###### ^1^Inovio Pharmaceuticals, Inc., Plymouth Meeting, PA, USA; ^2^The Wistar Institute, Philadelphia, PA, USA

####### **Correspondence:** David B. Weiner (dweiner@wistar.org)

**Background**

Chondroitin sulfate proteoglycan 4 (CSPG4), a transmembrane glycoprotein with functional roles in tumor migration, invasion, angiogenesis, and metastasis, has emerged as a promising tumor antigen target due to its overexpression in several solid cancer types and limited expression in normal tissue. In this study, we used Inovio’s proprietary SynCon® technology to design a synthetic consensus DNA vaccine targeting CSPG4 (SynCon® CSPG4) and assessed its immunogenicity and preclinical efficacy in a tumor-bearing mouse model of melanoma.

**Methods**

C57BL/6 mice (n=5 per group) were immunized with 25 μg of SynCon® CSPG4 or vector control. Four immunizations were administered, two weeks apart, with electroporation following each immunization. Cellular immune responses were measured by mouse IFN-γ ELISpot and flow cytometry. The breadth and magnitude of antigen-specific T-cell epitopes was further assessed by epitope mapping using consensus matched peptides. Vaccine-induced anti-tumor immunity was assessed in C57BL/6 mice transplanted with 5x10^4 YUMM1.7 murine melanoma cells (n=10). One week following implantation, these mice were immunized with 25 μg of SynCon® CSPG4. At the same time, naïve mice (n=10) were challenged with 5x10^4 YUMM1.7 tumor cells as a control.

**Results**

SynCon® CSPG4 generated strong cellular immune responses in mice, as assessed by IFN-γ ELISpot and flow

cytometry. An average of over 800 SFU/10^6 splenocytes were elicited in mice vaccinated with SynCon® CSPG4. The percentage of CD8+IFN-γ+ cells in mice immunized with SynCon® CSPG4 (0.552%) was significantly higher than that of the control mice (0.09%) (p<0.0442). Additionally, the SynCon® CSPG4-vaccinated mice showed a significant increase in the percentage of CD8+CD107a+ T cells, an indicator of cytotoxic potential, compared to control mice (1.07% vs. 0.034%, respectively (p<0.01)). Epitope mapping revealed that SynCon® CSPG4 could elicit broad T-cell immune responses. Notably, SynCon® CSPG4 generated cellular responses against three peptide sequences identical to native mouse CSPG4 sequences, suggesting SynCon® CSPG4 is capable of breaking tolerance. Importantly, SynCon® CSPG4 significantly slowed tumor growth and increased survival in the YUMM1.7 mouse model of melanoma.

**Conclusions**

Herein, we demonstrate that a DNA-based, synthetic consensus CSPG4 immmunogen induced robust anti-tumor immunity. DNA immunogens designed by SynCon® technology have the potential to break tolerance and induce anti-tumor immunity in cancer patients. Clinical investigation of SynCon® immunogens is warranted, both alone and in combination with checkpoint inhibitors.

**Ethics Approval**

The study was approved by the University of Pennsylvania Institutional Animal Care and Use Committee.

#### P156 Developing a cross-reactive heteroclitic peptide cancer vaccine targetting poorly immunogenic mutant calreticulin in myeloproliferative neoplasms

##### Mathieu Gigoux, PhD^1^, Shannon Elf, PhD^2^, Michele Ciboddo^2^, Roberta Zappasodi, PhD^1^, Stephane Pourpe, MSc^1^, Nouran Abdelfattah, MSc^2^, Timothy Chan, MD, PhD^1^, Omar Abdel-Wahab, MD^1^, Ann Mullally, MD^2^, Taha Merghoub, PhD^1^, Jedd Wolchok, MD, PhD^1^

###### ^1^Memorial Sloan Kettering Cancer Center, New York, NY, USA; ^2^Harvard, Boston, MA, USA

####### **Correspondence:** Mathieu Gigoux (gigouxm@mskcc.org)

**Background**

Disease-initiating mutations in calreticulin (CALR), an endoplasmic reticulum (ER) chaperone protein, are present in approximately 40% of myeloproliferative neoplasms (MPN). In recent work we demonstrated that expression of mutant CALR alone is sufficient to engender MPN in mice. We further showed that the thrombopoietin receptor, MPL is required for mutant CALR-driven transformation and that the oncogenicity of mutant CALR is dependent on the C-terminus of mutant CALR, which is necessary for physical interaction between mutant CALR and MPL. Although more than thirty CALR mutations have been identified, all result in the generation of a common, 36-amino acid novel C-terminal CALR peptide, representing a tumor-specific neo-antigen. We predicted that the tumor- specific neo-epitopes generated by the mutant CALR C-terminus induce immune responses in MPN patients.

**Methods**

Unfortunately, HLA-A*02:01, the MHC-I allele most frequently found in the Caucasian population, is unable to bind to any neo-antigens generated by the CALR mutations, based on the predictive algorithm and confirmed by in vitro assay. As expected, none of these peptides could generate T cell reactivity, as measured by the presence of IFNγ and TNFα-expressing T-cells after 10 days. We therefore hypothesized that T cell reactivity against the CALR mutants could be activated using heteroclitic peptides, which are peptides with a single amino-acid mutation that increases MHC-I binding 10 to 1000-fold, and have the potential to generate cross-reactivity against the native CALR mutant peptides. To predict high-quality heteroclitic peptides, we used the NetMHC-Epitoptimizatron (NEOn) algorithm developed by our group, which generates candidate heteroclitic peptides for any MHC-I allele in mice and humans. To test this strategy, we took advantage of the fact H2-Kb and H2-Db, MHC-I alleles expressed in C57BL/6J mice, also do not bind efficiently to CALR mutant peptides.

**Results**

We are currently in the process of evaluating the response in mice immunized with H2-Kb and H2-Db heteroclitic CALR mutant peptides. In addition, we are also testing the relevance of this findings in PBMCs from HLA-A*02:01 healthy donors with HLA-A*02:01 heteroclitic peptides in vitro. Once hetercolclitic peptides are validated they will be tested in MPN derived PBMCs.

**Conclusions**

The design of a heteroclitic vaccine based apporach in MPN patients provides an attractive stragey to elicit antitumor immunity in MPN patients.

**Ethics Approval**

The study was approved by the MSKCC ethics board. IRB# 06-107 A(15) and IACUC# 96-04-017

#### P157 ISA101 and nivolumab for HPV-16+ Cancer: Updated clinical efficacy and immune correlates of response

##### Bonnie Glisson, MD^1^, Erminia Massarelli, MD, PhD, MS^3^, William William, MD^4^, Faye M. Johnson, MD, PhD^4^, Renata Ferrarotto, MD^4^, Ming Guo^4^, Lei Feng^5^, J Jack Lee^6^, Hai Tran^6^, Jaime Rodriguez-Canales, MD^7^, Tomas Zecchini Barrese^6^, Ignacio Wistuba, MD^6^, Jing Wang^5^, Sjoerd van der Burg, PhD^8^, Kimal Rajapakshe, PhD^9^, Cornelis Melief, MD, PhD^10^, Michael Curran, PhD^6^

###### ^1^MD Anderson Cancer Center, Houston, TX, USA; ^2^U T M D Anderson Cancer Center, Houston, TX, USA; ^3^City of Hope, Duarte, CA, USA; ^4^M. D. Anderson Cancer Center, Houston, TX, USA; ^5^M D. Anderson Cancer Center, Houston, USA; ^6^M D Anderson Cancer Center, Houston, USA; ^7^Medimmune, Gaithersburg, MD, USA; ^8^Leiden University, Leiden, Netherlands; ^9^Baylor College of Medicine, Houston, USA; ^10^ISA Pharmaceuticals, Leiden, Netherlands

####### **Correspondence:** Bonnie Glisson (bglisson@mdanderson.org)

**Background**

Therapy with ISA101, an HPV16 peptide vaccine, and nivolumab demonstrated a promising overall response rate (ORR) of 33% (8/24) in pts with incurable HPV16+ cancer (1). We now present updated clinical efficacy and immune correlative analyses.

**Methods**

Patients with HPV16 tumors and ECOG PS 0-1, and <2 prior regimens for recurrence were eligible. ISA101 100 mcgs/peptide D1, 22, 50 and Nivolumab 3 mg/kg iv q 2 wks starting D8, were given for up to 1yr. Baseline tumor samples (FFPE) were stained with multiplex immunofluorescence (mIF) for PD-L1, PD-1, CD3, CD8, CD68, and pancytokeratin in a single panel, scanned with the Vectra 3.0™ multispectral microscope, and analyzed using inForm™ 2.3.1 software. Whole transcriptome analysis of baseline tumors was performed on Affymetrix Clariom™ D arrays. Differential gene expression analysis was performed on responders (PR + CR) vs. non-responders (SD+PD). P-values were corrected for multiple hypothesis testing

**Results**

All responding patients had oropharynx primaries. Of the 8 responders, 4 remain in response > 1 yr (13.6-18.7 months) with a median duration of 10.3 months (3.8-18.7+) . Median OS is 17.5 months (95% CI: 13.8 – inestimable) with median f/u of 17 months in censored patients. The scores for activated T cells [(CD3+PD-1+) + (CD3+CD8+PD-1+)], activated cytotoxic T cells (CD3+CD8+PD-1+), and total macrophages [(CD68+PD-L1-)+ (CD68+PD-L1+)] in tumor tissue were directly correlated with clinical response (p values all < 0.05, Wilcoxon), and depth of response with the two CR pts having the highest degree of CD8+ T cell infiltration. Gene expression analysis revealed differential regulation of 225 genes (1.25 fold or >) in responders vs non-responders (p = 0.05). High expression of interferon response genes and low expression of genes reflecting oxidative phosphorylation were correlated with clinical response. Enrichment in gene sets associated with interferon response and immune infiltration strongly predicted response to therapy, whereas elevated expression of genes associated with oxidative phosphorylation predicted progression.”

**Conclusions**

Efficacy of ISA101 and nivolumab remains promising in long-term f/u. A randomized trial is ongoing to test this strategy. Similar to data with anti-PD-1 alone, increased infiltration by PD-1+ T cells (CD3+ and CD3+CD8+) was predictive of response. The correlation between macrophages and clinical response fits with data from preclinical models and possibly involves IFNγ. Enrichment in gene sets associated with interferon response and immune infiltration strongly predicted response to therapy, whereas elevated expression of genes associated with oxidative phosphorylation predicted progression.”

**Trial Registration**

clinicaltrials.gov NCT0246892

**References**

1. Massarelli E, William WN, Johnson F et al Combining Immune Checkpoint Blockade and Tumor-specific Vaccine: Phase II Trial of Nivolumab and ISA101 in Patients with Incurable Human Papillomavirus 16-Related Cancers JAMA Oncology In Press

**Ethics Approval**

The study was approved by U. T. M. D. Anderson Cancer Center's IRB March 24, 2015.

#### P158 IFN-α and 5′-Aza-2′-deoxycytidine enhance the anti-tumor efficacy of a dendritic-cell targeting MIP3α- Gp100-Trp2 DNA vaccine by affecting T-cell recruitment and tumor microenvironment gene expression

##### James Gordy, PhD^1^, Richard Markham, BS, MD^2^

###### ^1^Johns Hopkins Bloomberg School of Public, Baltimore, MD, USA; ^2^Johns Hopkins BSPH, Baltimore, MD, USA

####### **Correspondence:** Richard Markham (rmarkha1@jhu.edu)

**Background**

The chemokine MIP-3α (CCL20) binds to CCR6 found on immature dendritic cells. DNA vaccines fusing MIP-3α to melanoma-associated antigens Gp100 and Trp2 have been shown to be effective in therapeutically reducing melanoma tumor burden in mouse models. To further enhance the therapy, our laboratory has added agents designed to overcome immunoregulatory mechanisms of the tumor microenvironment. Here, we report that the combination of type-I interferon therapy (IFN) with 5’-Aza-2’-deoxycitidine (Aza) profoundly enhanced the therapeutic anti- melanoma efficacy of a MIP-3α-Gp100-Trp2 DNA vaccine.

**Methods**

The current studies utilize the B16F10 mouse melanoma model. Vaccinations are administered intramuscularly followed by electroporation. Vaccinations are given thrice at one-week intervals, beginning day 5 post challenge. Two days post vaccination, CpG adjuvant is administered into the vaccinated muscle. Aza is given i.p.at 1mg/kg on the first two vaccination days. IFN is given in a series of one high dose followed by three low doses, beginning on the first two vaccination days. Tumor sizes, growth, and survival were assessed by Anova, linear regression, and log-rank survival respectively. Tumor microenvironment gene expression levels were explored by RT-PCR, with dCt values tested by Anova. Tumor-infiltrating lymphocytes (TILs) were assessed by measuring vaccine-stimulated TILs by intracellular cytokine staining flow cytometry, tested by Anova. All experiments have been repeated with sample sizes of 3-8 mice per group.

**Results**

We demonstrate that the addition of IFN and Aza significantly enhances the anti-tumor efficacy of a MIP-3α-Gp100-Trp2 vaccine. At day 19 post challenge, the triple treatment has tumors 69% smaller than vaccine alone. Mouse survival was also significantly increased, with the median survival increasing compared to vaccine by 39% and to negative control by 86%. Importantly, this increase in efficacy was dependent on the presence of all three components: vaccine, IFN, and Aza. All permutations of one or two treatments provided inferior efficacy. Aza and IFN additions to the vaccine increase T-cell tumor infiltration and alter the proportion of CD8+T-cells. Finally, we show that Aza and IFN induce durable changes in IFN-stimulated gene transcription that remain long after administration.

**Conclusions**

Efficient targeting of antigen to immature dendritic cells with a chemokine-fusion vaccine offers a potential alternative approach to ex vivo dendritic cell antigen-loading protocols currently undergoing clinical investigation. Combining this approach with IFN and Aza therapy significantly improved vaccine efficacy. This enhancement is correlated with changes in TILs and IFN-stimulated gene expression. This line of investigation has great potential to become a novel melanoma therapy.

**Ethics Approval**

This study was approved by the IACUC of Johns Hopkins University, protocol MO16H147

#### P159 Correlation between response and HLA type in a randomized phase IIb trial of NeuVax + trastuzumab in HER2 low-expressing breast cancer patients to prevent recurrence

##### Annelies Hickerson, MD^1^, Guy T. Clifton, MD, FACS^1^, Tommy Brown, MD^1^, John Myers, III, MD^1^, Jessica Campf, MD^1^, Kaitlin Peace, MD^1^, Jennifer K. Litton^2^, Rashmi Murthy^2^, Timothy J. Vreeland, BS MD^2^, Diane Hale, MD^1^, Garth Herbert, MD^1^, Jason Lukas, MD/PhD^3^, Nicholas J. Sarlis^4^, Jarrod P. Holmes^5^, Elizabeth A. Mittendorf^2^, George E. Peoples, MD, FACS^6^

###### ^1^Brooke Army Medical Center, Houston, TX, USA; ^2^The University of Texas, Houston, TX, USA; ^3^Providence Regional Cancer Partnership, Everett, WA, USA; ^4^Sellas Life Sciences Group, Inc., New York, NY, USA; ^5^Redwood Regional Medical Group, Santa Rosa, CA, USA; ^6^Cancer Vaccine Development Program, San Antonio, TX, USA

####### **Correspondence:** Annelies Hickerson (annelies.hickerson@gmail.com)

**Background**

MHC class I (MHCI) peptide vaccines are HLA-restricted but may bind to multiple HLA-types. HLA types have been associated with response to multiple immunotherapies to include checkpoint inhibitors. The relationships between HLA-type, predicted peptide binding potential, and clinical response have implications for the design and development of active immunotherapy. In a planned interim analysis of a randomized phase IIb trial of the MHCI peptide, E75 (HER2 369-377), + GM-CSF (NeuVax) + trastuzumab versus GM-CSF + trastuzumab to prevent recurrences in node positive (NP) and/or triple negative (TNBC), HER2 low-expressing breast cancer patients we demonstrated a significant disease free survival (DFS) benefit specifically in triple negative (TNBC) patients to NeuVax + trastuzumab. This analysis examines the effect of HLA-type on trial outcomes.

**Methods**

Clinically disease-free HER2 low-expressing (IHC1+/2+, FISH nonamplified) NP (AJCC N1, N2, or N3) and/or TNBC patients after standard therapy were tested for the presence of the A2, A3, A24, and A26 alleles by flow cytometry. HLA-A2, A3, A24, and/or A26+ patients were randomized to receive trastuzumab + NeuVax (vaccine group; VG) or trastuzumab + GM-CSF (control group; CG). All patients received 1 year of trastuzumab per label. NeuVax or GM-CSF was given every three weeks x 6 starting with the third trastuzumab dose, and then boosted every six months x 4. The pre-specified interim analysis was triggered six months after last patient enrollment. The primary endpoint was DFS evaluated by log rank. The MHCI binding predictions were made using the IEDB Analysis Resource Consensus tool.

**Results**

275 patients were randomized in the study (VG n=136, CG n=139). 146 were HLA-A2+ (VG=71, CG=75), 133 HLA-A24+ (VG=71, CG=61), 88 HLA-A3+ (VG=44, CG=44), and 19 HLA-A26+ (VG=10, CG=9). Median follow up was 18.8 months. There were no significant clinicopathologic difference between the VG and CG as a whole or within HLA-allele subgroups, except that fewer HLA-A24+ VG patients received radiation therapy (p=0.02). In TNBC patients, active treatment benefited all HLA-types (Figure 1) especially HLA-A24+ patients (Figure 2, p=0.02). HLA-A24+ VG patients also showed a trend toward improved DFS study-wide (Figure 3, p=0.07). HLA- A24+ has the lowest predicted binding affinity of the four HLA alleles.

**Conclusions**

HLA-A24+ TNBC patients had a significant improvement in DFS despite the lowest predicted binding potential between E75 and this HLA-type. This suggests that lower-affinity peptides may generate a favorable immunologic response possibly due to decreased exposure and tolerance to epitopes.

**Trial Registration**

Combination Immunotherapy With Herceptin and the HER2 Vaccine E75 in Low and Intermediate HER2- expressing Breast Cancer Patients to Prevent Recurrence, NCT01570036

**Ethics Approval**

The study was approved by Western Institutional Review Board, approval number 20130058.

**Fig. 1 (abstract P159).**
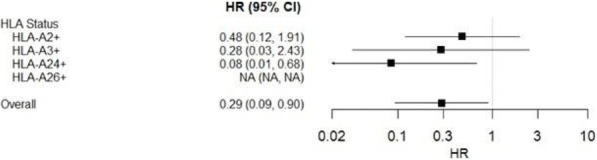
See text for description.

**Fig. 2 (abstract P159).**
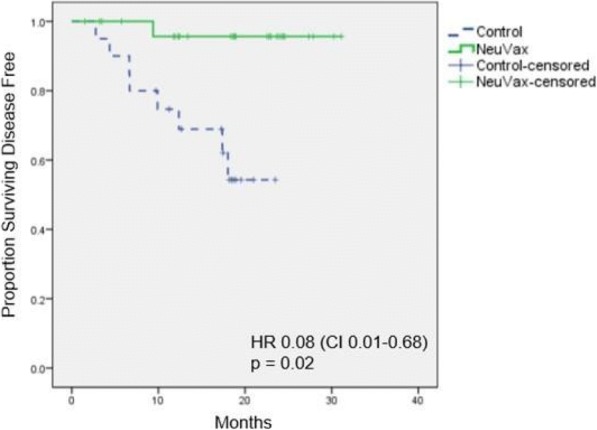
See text for description.

**Fig. 3 (abstract P159).**
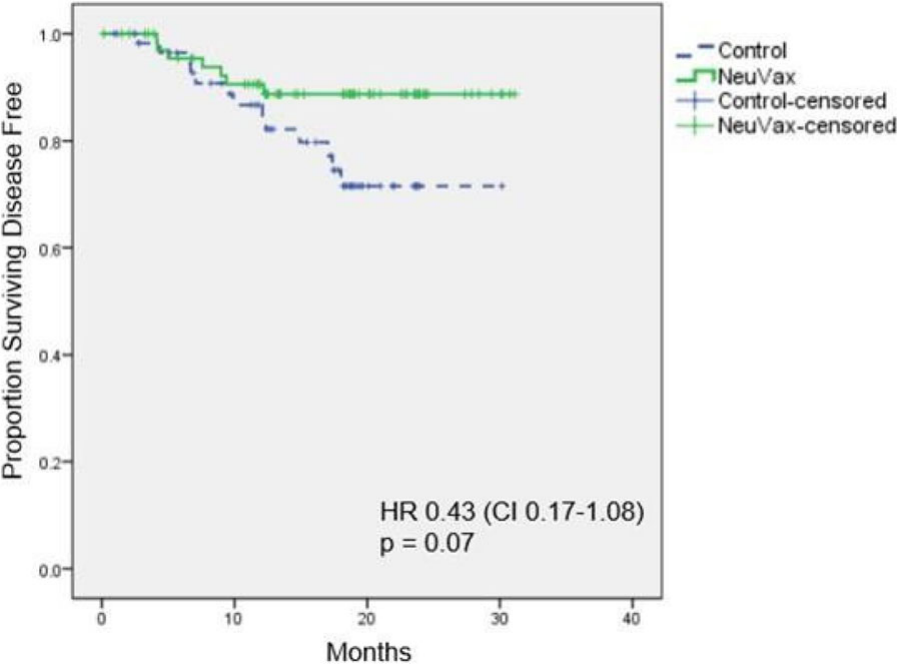
See text for description.

#### P160 Engineering a T cell stimulating extra-cellular matrix for immunotherapy

##### John Hickey, BS, Yi Dong, Hai-Quan Mao, PhD, Jonathon P. Schneck, MD, PhD, John Hickey, BS

###### Johns Hopkins University, Baltimore, MD, USA

####### **Correspondence:** Jonathon P. Schneck (jschnec1@jhmi.edu)

**Background**

T cells are the target of many immunotherapies; many, such as adoptive T cell therapy, require expansion of T cells to several thousand-fold. Expansion often comes at the cost of losing cytotoxic functionality and ability to persist as a memory cell which limits the success of these therapies. In tissue engineering, much effort has been taken to control environmental cues for differentiation, phenotype skewing, and proliferation of stem cells. We hypothesized that by controlling factors surrounding the microenvironment we could further activate T cells which would retain functionality to give improved immunotherapy.

**Methods**

We engineer an extracellular matrix out of hyaluronic acid and polyethylene glycol diacrylate. We can control stiffness and easily attach additional cell-attachment and stimulatory cues such as anti-CD3 or peptide-loaded MHC (major histocompatibility complex) and anti-CD28.

**Results**

First, we demonstrate that the extracellular matrix environment provides a “signal 3” to T cells being stimulated.

This is mediated by CD44 receptors on the surface of T cells and involves a crosstalk between the mTOR and Ras- Erk pathways with an ultimate upregulation of IL7 and IL15 receptors. Second, we engineered the extracellular matrix to present T cell stimulatory molecules to eliminate additional sources of stimulation. Here we found important biophysical properties of stiffness and extracellular matrix proteins influenced both the proliferation and phenotype of resultant T cells. Third, we stimulated rare antigen-specific T cells and adoptively transferred into a murine melanoma model and showed significant reduction of tumor burden compared to conventionally expanded T cells in plastic culture dishes.

**Conclusions**

Our studies demonstrate the importance of the environment, specifically the extracellular matrix in T cell stimulations. Additional engineering of the extracellular matrix enabled enhanced in vitro and in vivo functionality within an adoptive T cell therapy model. Further study and engineering of these extracellular environments could lead to substantial improvements in cellular therapies.

**Acknowledgements**

JWH is supported by NSF and ARCS Graduate Research Fellowships.

#### P161 The effects of IGF-1R antisense on cells of the glioma tumor microenvironment promotes immunostimulatory antigen release

##### D. Craig Hooper, Samantha Garcia, PhD, Rhonda Kean, MS, Aurore LeBrun, PhD, Emily Bongiorno, David Andrews, MD

###### Thomas Jefferson University, Philadelphia, PA, USA

####### **Correspondence:** D. Craig Hooper (douglasc.hooper@jefferson.edu)

**Background**

Glioblastoma (GBM) is the most lethal form of primary brain cancer. A highly anti-inflammatory tumor microenvironment (TME), in part due to the presence of tumor associated macrophages (TAMs) with M2-like properties, presents a significant obstacle to immunotherapy. While excised GBM tissues can be used as the basis of a personalized vaccine, this requires a strategy to generate antigens in an immunogenic format. Targeting the IGF- 1R expressed in GBM tissues, which sensitizes the cells to radiation [1] and modulates the function of other IGF- 1R-expressing cells in the TME may have utility in this regard. We show here that immune-stimulating antigens are released by tumor tissue treated ex-vivo in biodiffusion chambers with immunomodulatory IGF-1R antisense oligodeoxynucleotide and radiation.

**Methods**

GL261 cells were implanted stereotactically in the CNS of C57BL/6 mice and tumor tissue harvested at the appearance of clinical signs. Tumor tissues were dispersed, and aliquots subjected to different treatments prior to encapsulation in biodiffusion chambers. Fully-formulated chambers contained tumor tissues that had been cultured overnight with the IGF1-R antisense and then encapsulated with additional antisense and irradiated. Chambers were incubated in PBS or implanted in the flanks of naïve C57BL/6 mice overnight, then contents were retrieved and used to pulse naïve bone marrow dendritic cells (BMDCs). CD4+ T cells recovered from GL261-immune C57BL/6 mice were used to detect tumor antigenic determinants presented by the BMDCs with the readout being the number of cells producing IFNγ.

**Results**

The strongest IFNγ response was elicited by antigens recovered from fully-formulated chambers cultured in PBS overnight. If the overnight incubation with antisense was omitted a similarly immunogenic antigen preparation was generated only if the amount of antisense added to the chamber was increased. The contents of fully-formulated chambers implanted in mice overnight failed to stimulate immune CD4+ T cells in vitro suggesting that the antigen had been lost in the animals. However, these animals had been immunized and were protected against the growth of GL261 cells subsequently implanted in their CNS. Comparable results have been obtained in similar studies in other mouse tumor models.

**Conclusions**

Biodiffusion chambers containing irradiated glioma cells, prepared from excised tumor tissues, and an immunostimulatory IGF-1R antisense selectively produce glioma antigens in an immunogenic format appropriate for vaccination. The approach is currently under investigation in a clinical trial for patients with GBM (ClinicalTrials.gov, NCT01550523) and there is support from other mouse tumor models for its utility in diverse cancers.

**References**

1. Turner BC, et. al. Insulin-like growth factor-I receptor overexpression mediates cellular radioresistance and local breast cancer recurrence after lumpectomy and radiation. Cancer Res. 1997; 57:3079-83.

**Ethics Approval**

The study was approved by Thomas Jefferson University's Animal Care and Use Committee AWA A3085-10, AUP00902.

#### P162 Single cell sequencing to identify TCRs that recognize autologous tumor cells after vaccination with allogeinic DRibble vaccine

##### Hong-Ming Hu, PhD^1^, Christopher C. Paustian, PhD^2^, Zhifa Wen^3^, Tarsem L. Moudgil, MS^3^, Traci L. Hilton, PhD^2^, Sam Bookhardt, BA^2^, Guangjie Yu^3^, Eric Tran, PhD^3^, Venkatesh Rajamanickam^3^, Walter Urba, MD, PhD^3^, Rachel E. Sanborn, MD^3^, Bernard A. Fox, PhD^1^

###### ^1^Providence Portland Medical Center, UbiVac, Portland, OR, USA; ^2^UbiVac, Portland, OR, USA; ^3^Providence Portland Medical Center, Portland, Oregon, OR, USA

####### **Correspondence:** Hong-Ming Hu (hhu@providence.org)

**Background**

Adoptive immunotherapy with tumor-specific TCR gene-modified T cells has the potential to eradicate bulky disease. Traditional methods of TCR identification require lengthy in vitro culture to generate clonal T-cell populations, which adds time and complexity to this promising therapy. Here we described a simplified and reliable method to identify TCRs by single cell TCR sequencing of cells sorted with antibodies against T-cell surface markers that are up-regulated only when they are stimulated with specific tumor cell antigens.

**Methods**

A tumor-infiltrating lymphocyte (TIL) culture with T cells reactive against autologous tumor was generated from a brain metastasis of a patients with NSCLC. A panel of antibodies against T-cell surface antigens was screened to identify markers that are specifically up-regulated after stimulation with autologous tumors but not with related allogeneic tumor cells. Tumor-specific T cells were sorted from TIL with three suitable antibodies and expanded by a rapid expansion protocol. Expanded T cells were examined for their tumor-specificity and subjected to single cell TCR sequencing using the 10X genomic system. The top 10 TCRs were identified by bio-informatics approach and the corresponding alpha and beta chains were synthesized and cloned into a retroviral vector based on MSG backbone. PBMC from healthy donors were transduced with the retrovirus supernatant after activation. Tumor- reactivity of transduced T cells was determined after expansion in media supplemented with IL-2, IL-7, and IL-15. To identify tumor-specific TCRs in PBMC from the same patient after vaccination with allogeneic DRibbles, we also developed a protocol to expand tumor-specific T cells from PBMC with in vitro stimulation with DRibble- loaded PBMC.

**Results**

We identity CD94, CD137(4-1BB), CD355 (CRTAM) as specific markers for antigen-specific activation of T-cells by autologous tumor cells, whereas other “check point” markers such as CTLA-4, PD-1, Tim3, CD39, CD103 were up-regulated by stimulation with unrelated tumor cells. These antibodies were successfully used to sort and enrich tumor-specific T cells. The top 10 TCRs from each sorting were different but with overlapping clones. Five TCR clones were tumor-specific and capable to recognize the autologous tumor cells when they were expressed on T- cells from health donors. Additionally, ex-vivo culture of vaccine stimulated PBMC from a post-vaccine timepoint generated T cells enriched for activity against autologous tumor.

**Conclusions**

We developed a simplified work flow to identify tumor-specific TCRs. This flow will be further improved with antibody with DNA bar codes and used to identify tumor-reactive TCRs in a streamlined fashion.

**Acknowledgements**

The study was supported by Providence Portland Medical Foundation and NCI SBIR grant R44 CA121612.

**Ethics Approval**

The study was approved by EACRI Institutution‘s Ethics Board, approval number IRB: PDX06-108

#### P163 Humoral immune responses elicited by Globo H vaccine and the impact of Globo H expression levels on clinical response to Globo H vaccine in metastatic breast cancer

##### Jung-Tung Hung^1^, I-Ju Chen^2^, Wei-Chien Tang^2^, Shir-Hwa Ueng^3^, Tsai-Hsien Hung^1^, Fei-Yun Lo^1^, Hope Rugo, MD^4^, Chiun-Sheng Huang^5^, Alice Yu, MD, PhD^1^

###### ^1^Institute of Stem Cell & Translational Cancer Research; ^2^OBI Pharma, Inc., Taipei, Taiwan, Province of China; ^3^Chang Gung Memorial Hospital, Taoyuan, Taiwan, Province of China; ^4^University of California San Francisco Comprehensive Cancer Center, San Francisco, USA; ^5^National Taiwan University Hospital, Taipei, Taiwan, Province of China

####### **Correspondence:** Alice Yu (a1yu@ucsd.edu)

**Background**

An international randomized phase II trial of Globo H (GH) vaccine Adagloxad simolenin (OBI-822/821) in 349 patients with metastatic breast cancer (MBC) showed longer PFS in vaccinated patients who developed anti-Globo H (anti-GH) antibodies versus those who did not. The impact of immune responses to OBI-822/821 and GH expression on clinical outcome was further evaluated.

**Methods**

Anti-GH and anti-KLH antibodies were determined by ELISA. The binding ability and antibody-dependent cellular cytotoxicity (ADCC)/complement dependent cytotoxicity (CDC) of antisera were examined by flow cytometry and cytotoxicity assay, respectively. GH expression on tumors was detected by immunohistochemistry (IHC).

**Results**

OBI-822/821 elicited anti-GH IgM responses reached a maximum of ≥1:80 between week 4 and 12 (after 3 to 5 injections) and then declined. The mean anti-GH IgG titer peaked at week 40 (after 9 injections) and decreased once vaccination was complete. In the OBI-822/821 group, both GH responders (anti-GH IgG titer ≥1:160 at any time, N=64) and GH non-responders (anti-GH IgG titer <1:160, N=31) developed similar levels of anti-KLH IgG titers at week 40 (p=0.94, median titer=128,000, t-test on log transformed data). The sera of 40 patients with high anti-GH titers were assessed for their biological activities. By flow cytometry, 77.5% (31/40) and 17.5% (7/40) of their post- immune IgM and IgG, respectively, showed > 10% increase in binding to MCF7 cells. A 1.5-fold increase in CDC activity of post-immune sera over pre-immune sera was noted in 62.5% (25/40) of patients. ADCC responses were observed in 50% of the 40 patient samples tested.Among patients with higher GH expression by IHC (H score ≥ 80), there was a trend toward better PFS for the vaccinated (N=42) vs placebo group (N=23) (Hazard Ratio=0.59; 95% CI: 0.32, 1.10, P-value=0.10). For those with H score < 80, there was no difference in PFS between vaccinated and placebo groups (N=83 and 44 respectively. HR=1.22; 95% CI: 0.79-1.89, P-value=0.36).

**Conclusions**

Adagloxad simolenin induced antibodies against GH and KLH antigens in MBC. The anti-GH elicited by the vaccine was biologically active in binding to GH-positive cancer cells and mediating CDC/ADCC. Notably, in the vaccinated group, immune non-responders produced equivalent levels of anti-KLH antibody as immune responders, suggesting a similar capacity to respond to foreign antigens. A trend for better PFS in subjects with higher GH expression treated with OBI-822/821 versus placebo suggests the need for pre-screening GH expression in future trials of OBI-822/821.

**Trial Registration**

NCT03562637

**Ethics Approval**

The study was approved by Chang Gung Medical Foundation Institututional Review Board, approval number 102-3670A3.

#### P164 Personalized cancer vaccines based on peptide-TLR-7/8a conjugates induce CD8 T cell responses to neoantigens in primates that are dependent on TLR-7/8a potency

##### Andrew Ishizuka, DPhil^1^, Andrei Ramirez-Valdez^2^, Hide Yamane, PhD^2^, Yaling Zhu, PhD^1^, Vincent Coble^1^, Faezzah Baharom^2^, Kennedy Tobin^2^, Brennan Decker^1^, Geoffrey Lynn^1^, Robert A. Seder, MD^2^

###### ^1^Avidea Technologies, Baltimore, MD, USA; ^2^VRC, NIAID, NIH, Bethesda, MD, USA

####### **Correspondence:** Robert A. Seder (rseder@mail.nih.gov)

**Background**

Personalized cancer vaccines (PCVs) aim to enhance neoantigen-specific T cell responses. We developed a PCV platform based on peptide-TLR-7/8 agonist conjugates that self-assemble into nanoparticles (termed SNP-7/8a). SNP-7/8a ensures co-delivery of neoantigens with TLR-7/8a, and significantly expands the breadth of neoantigen- specific CD8 T cell responses in mice. Here, we determined how the potency of the co-delivered TLR-7/8a affected the magnitude and quality of neoantigen-specific CD8 T cells in mice and primates.

**Methods**

Mice were vaccinated with SNP-7/8a co-delivering an MC38 neoantigen (Adpgk) and either a high potency (EC50 = ~20 nM) imidazoquinoline TLR-7/8a (termed ‘2BXy’) or moderate potency (EC50 = ~ 600 nM) TLR-7/8a (termed ‘2B’). CD8 T cell responses were evaluated by tetramer staining. Studies were subsequently conducted in rhesus macaques (RM) as TLR expression in RM and humans is highly similar. RM with the MHC-I allele Mamu- A*01 were vaccinated subcutaneously with SNP-7/8a co-delivering mock neoantigens predicted to bind Mamu- A*01 and either 2BXy or 2B. CD8 T cell responses were measured directly ex vivo by peptide stimulation and intracellular cytokine staining for IFN-g.

**Results**

After the first immunization, the CD8 T cell response was higher in mice vaccinated with 2BXy-based SNP-7/8a.

However, there was no difference in the magnitude of the Adpgk-specific T cell responses after subsequent vaccinations. Interestingly, 2BXy-vaccinated mice had a significantly higher proportion of Adpgk-specific cells that had differentiated into KLRG1+CD127– short-lived effector cells (SLECs), whereas 2B-vaccinated mice had a higher proportion of KLRG1–CD127+ memory-precursor effector cells (MPECs). In primates, neoantigen-specific CD8 and CD4 T cells were identified directly ex vivo after a single vaccination with SNP-7/8a. While 2BXy- vaccinated RM trended towards a higher magnitude neoantigen-specific CD8 and CD4 T cell response after the first vaccination, the responses underwent a greater expansion in 2B-vaccinated animals compared to 2BXy-vaccinated animals. Moreover, 2BXy-vaccinated RM showed a trend towards having a higher proportion of IFN-g+ neoantigen-specific CD8 T cells that had terminally differentiated into CCR7–CD45RA+ (TEMRA) cells compared to 2B-vaccinated RM, where the response favored less differentiated CCR7+CD45RA– (Tcm).

**Conclusions**

SNP-7/8a induced neoantigen-specific CD8 and CD4 T cell responses in primates. Interestingly, the moderate potency TLR-7/8a led to T cell responses that were balanced between effectors and memory cells, which may be optimal for tumor vaccines. Efforts to define the innate mechanisms controlling CD8 T cell differentiation following SNP-7/8a vaccination are underway.

**Ethics Approval**

These studies were approved by the Animal Care and Use Committee of the VRC, NIAID, NIH.

#### P165 Withdrawn

#### P166 Ex vivo ATLAS-identified inhibitory neoantigens promote mouse melanoma tumor progression

##### Hanna Starobinets, PhD^1^, Kyle Ferber, PhD, BS^1^, Jason R. Dobson, PhD, BS^1^, Michael O'Keeffe^1^, Crystal Cabral, BS, MSc^1^, Matthew Lanchantin, BS^1^, Erick Donis^1^, Peri Matatia^1^, Erik Carter^1^, Adrienne Li, PhD^1^, James Loizeaux^1^, Jamie Foti, PhD^1^, Wendy Broom, PhD^1^, Pamela Carroll^1^, Paul Kirschmeier^2^, Jessica B. Flechtner, PhD^1^, Hubert Lam, PhD^1^

###### ^1^Genocea Biosciences, Cambridge, MA, USA; ^2^Dana-Farber Cancer Institute, Boston, USA

####### **Correspondence:** Jessica B. Flechtner (jessica.flechtner@genocea.com)

**Background**

Neoantigens are attractive targets for personalized cancer immunotherapy due to their recognition as foreign antigens not subject to central tolerance. Personalized cancer vaccines leverage neoantigens to direct the immune system to specifically recognize tumor cells for their coordinated attack and destruction. Although not well understood, published data also suggest that some immunotherapies result in hyperprogression. One hypothesis for this phenomenon is antigen-specific immune modulation by T cells. ATLAS™ is a T cell profiling platform whereby putative antigens can be screened ex vivo using autologous antigen presenting cells (APCs) and T cells.

Antigens are differentially characterized as stimulatory or inhibitory by significant up- or downregulation of T cell cytokine secretion relative to control responses; thus, the ATLAS assay allows for identification and characterization of desired as well as potentially unwanted antigen-specific T cell responses

**Methods**

A melanoma model was employed to identify murine neoantigens and inhibitory antigens using ATLAS. Mice were implanted subcutaneously with B16F10 tumors, which were subsequently resected for whole exome sequencing and assessed for non-synonymous mutations. ATLAS libraries individually expressing each mutation were constructed and used to screen splenic T cells from tumor bearing mice to identify stimulatory or inhibitory neoantigens. Candidate neoantigens were manufactured as synthetic long peptides and delivered subcutaneously to C57BL/6 mice with or without adjuvant to elucidate the ability of stimulatory or inhibitory vaccines to impact tumor growth.

**Results**

Non-synonymous mutations (>1600) were identified and incorporated into the ATLAS library. After T cell screening, multiple neoantigens were identified that differentially modulated the secretion of inflammatory cytokines. ATLAS-identified neoantigens were not enriched for NetMHCpan-predicted epitopes, known oncogenes, or tumor suppressor genes. In addition, there was no preferential enrichment of frame-shift mutations, insertions or deletions. After vaccination with ATLAS-identified antigens, significant T cell responses to stimulatory vaccine neoantigens were observed as well as modest anti-tumor efficacy against B16F10 tumor challenge. Strikingly, therapeutic immunization with inhibitory neoantigen peptides led to a marked and significant increase in tumor growth kinetics. Studies to further investigate the immunological mechanisms will be reported.

**Conclusions**

These studies demonstrate proof of concept in mice for therapeutic efficacy using the ex vivo ATLAS platform to prioritize neoantigens included in personalized vaccines. In addition, they demonstrate the existence and biological importance of vaccine antigens that lead to hyperprogression. A Phase 1/2a clinical trial of a targeted personalized cancer vaccine, GEN-009, filtered for inclusion of ATLAS-identified stimulatory antigens and exclusion of inhibitory antigens, is ongoing.

**Acknowledgements**

We would like to thank Catarina Nogueira for early exploratory work.

#### P167 Characterization of antigen specific immune responses from a first-in-human study evaluating the anti-ASPH cancer vaccine SNS-301 in biochemically relapsed prostate cancer patients

##### Michael Lebowitz, PhD^1^, Kanam Malhotra^1^, M.S. Walker^1^, Hong Dai, MD, PhD^1^, Michael S. Lebowitz, PhD^1^, Steven Fuller, PhD^1^, Amir Shahlaee, MD^1^, James J. Elist, MD^2^, Neal Shore, MD^3^, Luke Nordquist, MD, FACP^4^, Hossein Ghanbari, PhD^1^

###### ^1^Sensei Biotherapeutics, Gaithersburg, MD, USA; ^2^James J. Elist, MD, Beverly Hills, CA, USA; ^3^Carolina Urologic Research Center, Myrtle Beach, SC, USA; ^4^GU Res. Network/Urology Cancer Center, Omaha, NE, USA

####### **Correspondence:** Michael Lebowitz (mlebowitz@senseibio.com)

**Background**

SNS-301 (formerly PAN-301) is a first-in-class immunotherapeutic cancer vaccine candidate targeting human aspartyl (asparaginyl) β-hydroxylase (ASPH). ASPH is an oncofetal antigen expressed prevalently in multiple human cancers but not in healthy adult tissue and that has not been targeted in any prior clinical studies. As a biologic modifier of the NOTCH pathway, ASPH is associated with tumor cell growth, motility and invasiveness. SNS-301 is a vaccine which delivers over 400 copies of an extracellular domain of the ASPH protein displayed on the coat of a bacteriophage vector. Previously presented pre-clinical data demonstrated the vaccine’s ability to overcome tumor self-tolerance and to provide immune-mediated anti-tumor effects [1,2]. The safety and tolerability of SNS-301 have also been presented previously [3].

**Methods**

SNS-301 was administered intradermally every 21 days in a 3+3 dose-escalation trial evaluating 3 dose levels to patients with biochemically (rising PSA) relapsed prostate cancer with evidence of ASPH over-expression as detected in serum. Secondary immunologic endpoints included the calculation of geometric mean titers of anti- ASPH antibodies; stimulation of innate immune responses; production and persistence of antigen-specific B cell and T cell responses; and the correlation of anti-ASPH titers and serum ASPH levels.

**Results**

Twelve patients with measurable serum levels of ASPH received 6-18 doses of SNS-301 (median = 10 doses). All patients experienced dose-dependent ASPH-specific immune responses including B-cell, T-cell and antibody responses. Specifically, increases in activated, IFN-γ releasing T-cells were demonstrated, including activated patient-derived CD4+ helper T cells as demonstrated by cytokine release subsequent to in vitro stimulation with either SNS-301 or recombinant ASPH. Anti-ASPH antibody titers also increased in a dose-dependent fashion over the first 4-6 cycles (80-120 days) of vaccination. This increase correlated with concomitant increases in the percentages of ASPH-specific B-cells as measured by flow cytometry. Immune responses occurred faster and were more robust at the two higher doses vs. the lower dose. Immunologic efficacy generally correlated with biochemical responses in these patients.

**Conclusions**

In this phase I setting, the SNS-301 vaccine induced vibrant and durable antigen-specific immune responses, which generally correlated with biochemical responses. Based on these cumulative results, a multi-site phase 2 efficacy clinical trial will commence enrollment in the 2nd half of 2018.

**Trial Registration**

ClinicalTrials.gov Identifier: NCT03120832

**References**

1. Tomimaru Y, Mishra S, Safran H, Charpentier KP, Martin W, De Groot AS, Gregory SH, Wands JR. Aspartate-β-hydroxylase induces epitope-specific T cell responses in hepatocellular carcinoma. Vaccine. 2015; 33:1256-66.

2. Iwagami Y, Casulli S, Nagaoka K, Kim M, Carlson RI, Ogawa K, Lebowitz MS, Fuller S, Biswas B, Stewart S, Dong X, Ghanbari H, Wands JR. Lambda phage-based vaccine induces antitumor immunity in hepatocellular carcinoma. Heliyon. 2017; 3:e00407.

3. Nordquist LT, Shore ND, Elist JJ, Oliver JC, Gannon W, Shahlaee AH, Fuller SA, Ghanbari HA. Phase 1 open- label trial to evaluate the safety and immunogenicity of PAN-301-1, a novel nanoparticle therapeutic vaccine, in patients with biochemically relapsed prostate cancer. J Clin Oncol. 2018;36:suppl; abstr e15166.

**Ethics Approval**

The study was approved by Schulman IRB, approval number 201606469.

#### P168 Induction of chemokine receptor and suppression of inhibitory receptors of CD8 T cell controlled effectively cervicovaginal tumor in mouse

##### Daewoo Lee, MD^1^, Daeun Nam^1^, Sungjong Lee, PhD, MD^2^

###### ^1^The Catholic University of Korea, Bucheon, Korea, Republic of; ^2^St. Vincent's Hospital, Suwon, Korea, Republic of

####### **Correspondence:** Sungjong Lee (orlando@catholic.ac.kr)

**Background**

Activation of exhausted CD8 T cell and migration of immune cells into tumor site is an important for overcoming resistance to cancer therapy. We evaluated the role of suppression of inhibitory receptors and chemokine axis in cervicovaginal tumor bearing mouse.

**Methods**

C57BL/6 mice were categorized into four groups according to treatment modality. Mice were challenged with 1×105 TC-1 cells on cervix and vagina. HPV DNA therapeutic vaccine was injected intramuscularly and intratumoral injection of GMCSF was performed. The mice were harvested on day 21 and immune cells were investigated by flow cytometry. We checked the expression of inhibitory receptors of CD8 T cells, including PD1, TIM3 and LAG3. Chemokine axis such as CXCL9, CXCL10, and CXCR3 were evaluated to know migration mechanism.

**Results**

Combination of HPV DNA vaccine and GMCSF resulted in significantly lower expression of TIM3 inhibitory receptors of CD8+ T cells in tumor (p<0.05) (Fig 1). However, expression level of PD1 and LAG3 was not changed after combination therapy. They significantly induced accumulation of tumor specific CD8 T cell in tumor site and increased expression of CXCR3 on tumor infiltration CD8 T cell (p<0.05). CXCL9, chemokine, was overexpressed in cervicovaginal tumor after combination therapy (p<0.05) (Fig 2). However, expression level of CXCL10 was not changed after combination therapy. Finally, mice treated with combination therapy survived significantly longer than other groups with single therapy (p<0.05).

**Conclusions**

In conclusion, we overcame T cell exhaustion and identified chemokine axis during migration of CD8 T cell into cervicovaginal tumor using HPV DNA vaccine and GMCSF. This mechanism can be ideal target for future immunotherapy.

**Fig. 1 (abstract P168).**
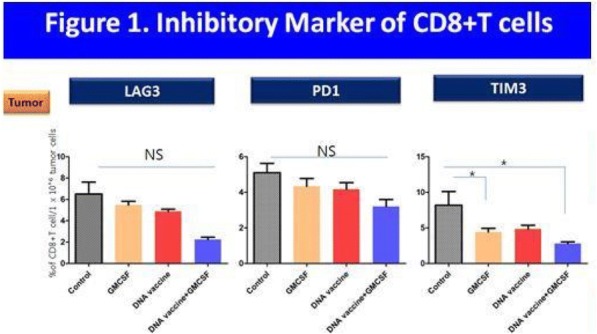
Inhibitory marker and chemokine

**Fig. 2 (abstract P168).**
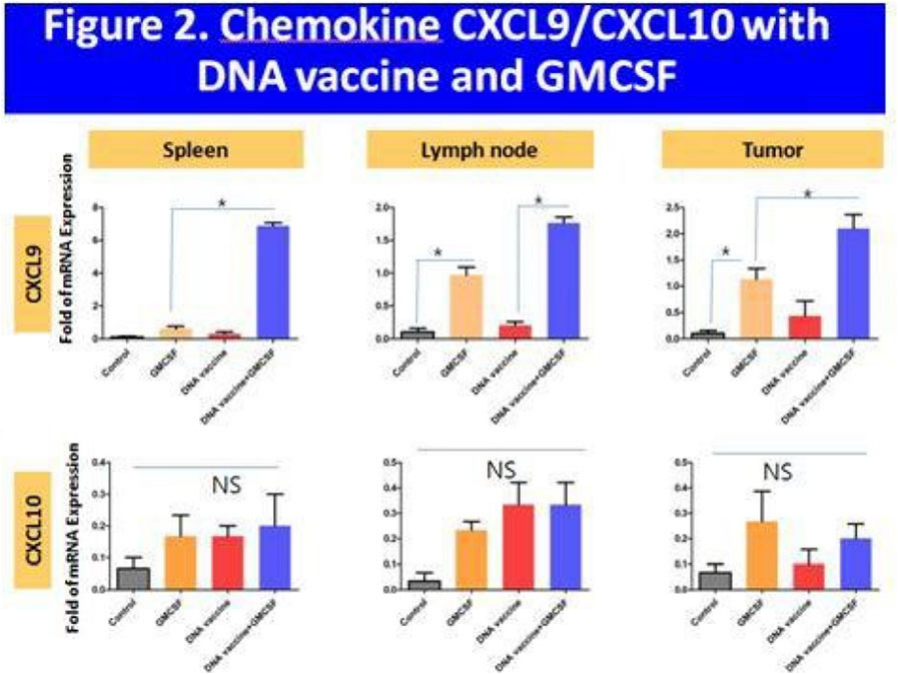
Inhibitory marker and chemokine

#### P169 In-Depth Characterization of Immune Responses Induced Against Patient-Specific Neoantigens using NEO- STIM™

##### Divya Lenkala, Brian McCarthy, Michael Nelson, Rachel Debarge, Janani Sridar, Yusuf Nasrullah, Master's in Biological Research, UK, Jessica Kohler, PhD, Matthew Goldstein, MD, PhD, Richard Gaynor, MD, Marit M. Van Buuren, PhD

###### Neon Therapeutics, Cambridge, MA, USA

####### **Correspondence:** Marit M. Van Buuren (mvanbuuren@neontherapeutics.com)

**Background**

Neoantigens are tumor-specific antigens important in eliciting and directing effective anti-tumor immune responses.

These antigens are not subject to central immune tolerance and are therefore potentially more immunogenic than tumor-associated antigens (1,2). A better understanding of the rules governing neoantigen immunogenicity will improve our ability to select high-quality targets for T cell responses with the potential to treat patients.

**Methods**

Patient-specific neoantigens were predicted using our bioinformatics pipeline, RECON®, and were used as immunogens in our proprietary ex-vivo stimulation protocol NEO-STIM to assess immunogenicity. Peptides were loaded into antigen presenting cells (APCs) and utilized to induce memory and de novo T cell reactivity from autologous peripheral blood mononuclear cells (PBMCs). In-depth analysis of the induced responses was performed through combinatorial coding analysis using peptide MHC (pMHC) multimers (3); functional analyses with multiplexed, multiparameter flow cytometry and cytotoxicity assays. Data from these analyses were used to characterize the specificity, functionality, sensitivity, phenotype and killing capacity of the induced T cells.

**Results**

Here we show exemplary data from one patient (ClinicalTrials.gov: NCT02897765) in which we induced multiple CD8+ and CD4+ T cell responses, both memory and de novo, towards predicted patient-specific neoantigens. These induced responses were reactive to the mutated neoantigens and not to the corresponding wild type antigen. These mutant-specific responses exhibited polyfunctionality in response to peptide stimulation along with capability to kill antigen expressing tumor cell lines. In conclusion, patient-specific neoantigens predicted by RECON were confirmed to be immunogenic by NEO-STIM induction of neoantigen-specific T cell responses.

**Conclusions**

NA

**References**

1. Yarchoan M, Johnson III BA, Lutz ER, et al. Targeting neoantigens to augment antitumor immunity. Nature Reviews Cancer. 2017; 17:209–222.

2. Martin SD, Coukos G, Holt RA, et al. Targeting the undruggable: immunotherapy meets personalized oncology in the genomic era. Annals of Oncology. 2015; 26:2367–2374.

3. Andersen RS, Kvistborg P, Frøsig TM, et al. Parallel detection of antigen-specific T cell responses by combinatorial encoding of MHC multimers. Nature Protocols. 2012; 7:891–902.

#### P170 Identification of breast cancer neoantigens exposed by radiation therapy

##### Claire Lhuillier, PhD, Nils-Petter Rudqvist, PhD, Takahiro Yamazaki, PhD, Tuo Zhang, PhD, Lorenzo Galluzzi, PhD, Sandra Demaria, MD

###### Weill Cornell Medical College, New-York, NY, USA

####### **Correspondence:** Claire Lhuillier (cfl2002@med.cornell.edu)

**Background**

Recent studies have highlighted the key role of mutation-generated neoantigens in tumor response to immunotherapy [1]. We have previously shown in the BALB/c-derived 4T1 mouse model of immune-checkpoint blockade (ICB)-resistant metastatic breast cancer that tumor-targeted radiation therapy (RT) combined with CTLA-4 blockade induces the CD8+ T cell-mediated regression of irradiated tumors and inhibits lung metastases [2]. Analysis of the T-cell receptor repertoire indicated that unique clonotypes expand in treated tumors, suggesting that tumor rejection involves T cells reactive to a set of tumor antigens that are made available to the immune system by RT [3]. Therefore, we hypothesize that RT increases the expression of genes containing immunogenic mutations and hence promote priming of neoantigen-specific T cells.

**Methods**

We performed whole-exome sequencing and RNA sequencing of untreated and irradiated (8GyX3) 4T1 cells *in vitro* to identify tumor-specific neoantigens and determine which ones are upregulated by RT. In addition, these mutations were documented *in vivo*, in 4T1 tumors harvested before and after treatment (8GyX3 + anti-CTLA-4). Several algorithms were used to predict MHC-I and MHC-II-binding epitopes from these mutated genes. Peptides with a predicted affinity <500 nM were synthesized and tested *in vitro* for binding to H2-Ld or H2-Kd in a MHC stabilization assay using RMA-S cells. Peptides showing stable binding in this assay were used to vaccinate BALB/c mice, followed by challenge with 4T1 cells to test for the induction of protective anti-tumor immunity.

**Results**

Out of 309 total mutations initially identified in 4T1 cancer cells, 22 predicted MHC-I-binding epitopes were tested *in vitro* and 6 of them were confirmed to bind to H2Ld or H2Kd. For MHC-II, we identified two I-Ad-predicted binders, which were tested in vaccination experiments. Results showed that 2 MHC-I and 1 MHC-II neoepitopes were immunogenic, as assessed by IFNγ/TNFα response after T cells re-stimulation. Two of the three neoepitopes were encoded by genes upregulated by RT. Although a vaccine based on these three neoepitopes is not sufficient to inhibit tumor growth, a significant tumor growth delay was seen when vaccination was combined with tumor- targeted RT and an OX40 agonist.

**Conclusions**

Further analyses are ongoing to understand the regulation of RT-exposed neoantigens *in vivo* and their contribution to T cell-mediated tumor rejection. In conclusion, these data provide initial proof-of-principle evidence that RT can expose existing neoantigens to the immune system.

**References**

1. Schumacher TN, Schreiber RD. Neoantigens in cancer immunotherapy. Science. 2015; 348:69-74.

2. Demaria S, Kawashima N, Yang AM, Devitt ML, Babb JS, Allison JP, Formenti SC. Immune-mediated inhibition of metastases after treatment with local radiation and CTLA-4 blockade in a mouse model of breast cancer. Clin Cancer Res. 2005; 11:728-734.

3. Rudqvist NP, Pilones KA, Lhuillier C, Wennerberg E, Sidhom JW, Emerson RO, Robins HS, Schneck J, Formenti SC, Demaria S. Radiotherapy and CTLA-4 Blockade Shape the TCR Repertoire of Tumor-Infiltrating T Cells. Cancer Immunol Res. 2018; 6:139-150.

#### P171 Safety and immunogenicity of a DNA vaccine encoding PSA and PSMA in patients with biochemically recurrent prostate cancer

##### Li Liu, PhD^1^, Neal Shore, MD^2^, Elisabeth Heath^3^, Matthew Morrow, PhD^4^, Kimberly Kraynyak, BS PhD^4^, Kamal Bhatt, LCEH MS^4^, Trevor McMullan^4^, Jessica Lee, MS, MPH^4^, Brian Sacchetta^4^, Samantha Rosencranz^4^, Ildiko Csiki, BS MD, PhD^4^, Mark Bagarazzi, MD^1^

###### ^1^Inovio Pharmaceuticals Inc, Plymouth Meeting, PA, USA; ^2^Carolina Urologic Research Center, Myrtle Beach, SC, USA; ^3^Wayne State University, Detroit, MI, USA; ^4^Inovio Pharmacetuicals, Plymouth Meeting, PA, USA

####### **Correspondence:** Mark Bagarazzi (mbagarazzi@inovio.com)

**Background**

After definitive therapy, approximately one third of prostate cancer (PCa) patients (pts) will experience rising prostate specific antigen (PSA) levels, a condition known as biochemical recurrence. Active immunotherapies, or antitumor vaccines, are appealing as potential treatments to eradicate micrometastatic disease. The choice of vaccine antigens in prostate cancer has largely been guided by the presence of proteins whose expression is known to be essentially restricted to the prostate, including PSA and prostate-specific membrane antigen (PSMA). Various prostate cancer vaccine platforms targeting these antigens have been reported. In the current study, we evaluated the immunogenicity and safety of INO-5150, a DNA vaccine encoding PSA and PSMA, with or without INO-9012 (encoding IL-12 immune adjuvant), in men with biochemically relapsed prostate cancer.

**Methods**

62 post-prostatectomy/radiation therapy pts with rising PSA were divided into 4 cohorts in a phase I, open-label, multi-center study and received INO-5150 ± INO-9012 (A, 2mg INO-5150; B, 8.5 mg INO-5150; C, 2mg INO- 5150+1mg INO-9012; D, 8.5mg INO-5150+1mg INO-9012). INO-5150±INO-9012 was administered via intramuscular (IM) injection followed by electroporation with the CELLECTRA® device on day 0, weeks 3, 12 and 24 then followed through study week 72. Safety, immunogenicity and efficacy were assessed for all evaluable pts.

**Results**

50/62 (81%) pts have completed all visits. 90% pts had Grade (Gr) 1-3 AEs, primarily injection site reactions, which were Gr1. 48/61(79%) evaluable subjects demonstrated immune responses to PSA/PSMA antigens. The response rate in ELISpot and ELISA assays were 70% (40/57) and 23% (14/61) respectively. In addition, analysis of antigen specific CD8+ T cells with lytic potential was conducted using flow cytometry. CD8+ T cells co-expressing CD38 and perforin were induced in 23/50 (46%) pts, who also experienced attenuated PSA increase (p=0.05 for PSA attenuation). In pts with stable disease (SD, N=20), treatment with INO-5150±INO-9012 significantly increased PSMA-specific CD8+ T cells with cytolytic potential as evidenced by the co-expression of the activation markers CD38 and CD69, expression of PD1 and upregulation of granulysin, granzyme A, granzyme B, and perforin compared to pts with progressive disease (PD) (p=0.024, N=30). 75% (27/36) of the patients with baseline (D0) PSADT≤12 months became stabilized with a significantly prolonged PSADT at WK72 (p<0.0001, N=27).

**Conclusions**

INO-5150 +/- INO-9012 was safe and immunogenic. A therapeutic effect was demonstrated in patients with D0 PSADT of≤ 12 months, as manifested by their dampening % rise in PSA and prolonged PSADT up to 72 weeks follow up.

#### P172 Personalized cancer vaccines based on self-assembling nanoparticles co-delivering peptide antigens and TLR- 7/8 agonists (SNP-7/8a) enhance the breadth and magnitude of anticancer CD8 T cell immunity

##### Geoffrey Lynn^1^, Faezzah Baharom^2^, Yaling Zhu, PhD^1^, Ramiro A. Ramirez-Valdez, BA Cantab^2^, Tobin Kennedy^2^, Vincent Coble^1^, Brennan Decker^1^, Hide Yamane, PhD^2^, Andrew S. Ishizuka, DPhil^1^, Robert A. Seder, MD^2^

###### ^1^Avidea Technologies, Inc., Baltimore, MD, USA; ^2^National Institutes of Health

####### **Correspondence:** Robert A. Seder (rseder@mail.nih.gov)

**Background**

Advances in genomic sequencing and in silico prediction algorithms have enabled the development of personalized cancer vaccines (PCVs) targeting tumor-specific neoantigens. With current peptide- and RNA-based PCVs, only ~10% of predicted neoantigens induce de novo CD8 T cell responses. Here, we show that the efficiency for inducing CD8 T cells to peptide antigens can be significantly improved and is highly dependent on the physical form of the peptide. Accordingly, particulate peptide antigens, unlike soluble peptide antigens, are taken up efficiently by dendritic cells in lymphoid tissue and promote CD8 T cell expansion through prolonged antigen presentation. Thus, a major challenge for peptide-based PCVs is ensuring consistent particle formulations despite the broad range of neoantigen physicochemical properties.

**Methods**

We developed a novel PCV platform based on charge-modified peptide-TLR-7/8 agonist conjugates that are chemically programmed to self-assemble into nanoparticles (“SNP-7/8a”) of a defined size (20–50 nm) irrespective of the underlying neoantigen composition. Several hundred unique SNP-7/8a compositions were synthesized to systematically screen how different parameters of SNP-7/8a (size, charge, peptide antigen length, etc.) impact formulation characteristics and in vivo immunogenicity.

**Results**

Bioinformatics analysis of the characteristics of 72 million human genome-derived neoantigens was used to demonstrate that SNP-7/8a ensures consistent nanoparticle formation with neoantigens at extremes of charge (-6 to +6) and hydropathy (GRAVY, -2 to +2). Vaccination of mice with SNP-7/8a using predicted neoantigens (n = 179) from three tumor models induced CD8 T cells against ~50% of those with high predicted MHC-I binding affinity (IEDB consensus score < 0.5). Importantly, SNP-7/8a induced CD8 T cells that inhibited tumor growth against several neoantigens that were reported non-immunogenic using conventional peptide- or RNA-based vaccines.

**Conclusions**

SNP-7/8a based on charge modified conjugate vaccines represents a universal approach for co-delivering peptide antigens and adjuvants in nanoparticles to increase the magnitude and breadth of anticancer T cell immunity.

#### P173 Early results of a phase I clinical trial of a HER2 dendritic cell cancer vaccine

##### Hoyoung Maeng, MD^2^, Lauren V. Wood, MD^2^, Lee England, PA-C^2^, Brenda Roberson, BSN, RN, OCN^3^, Santhana Webb^2^, Brittni Moore^2^, David F. Stroncek, MD^3^, John C. Morris, MD^4^, Jay A. Berzofsky, MD, PhD^2^

###### ^1^NCI, Bethesda, MD, USA; ^2^National Cancer Institute, Bethesda, MD, USA; ^3^National Institute of Health, Bethesda, MD, USA; ^4^University of Cincinnati, Cincinnati, OH, USA

####### **Correspondence:** Hoyoung Maeng (hoyoung.maeng@nih.gov)

**Background**

We have developed a therapeutic cancer vaccine targeting HER2 based on autologous dendritic cells (DCs) transduced with an adenovirus (AdHER2) expressing the non-signaling extracellular and transmembrane domains of HER2, a driver oncogene in many cancers that is often associated with worse outcome. In mice, the homologous vaccine cured virtually all mice with established tumors up to 2 cm or with established macroscopic lung metastases. The protection was dependent on antibodies against HER2 that inhibited HER2 phosphorylation, but was FcR (ADCC) independent, unlike the mechanism of trastuzumab, an FDA approved anti-HER2 antibody. We translated this discovery to a clinical trial in the NIH Clinical Center.

**Methods**

This is an open-label, non-randomized phase I clinical trial (NCT01730118) in patients with advanced metastatic cancers who have progressed after a minimum of one standard therapy, whose tumor is HER2 immunohistochemistry (IHC) score 1+, 2+ or 3+ or HER2/CEP17 ratio ≥ 1.8 by FISH. Part 1 of the study enrolled cancer patients naïve to any HER2-directed therapies to assess the safety and immunologic response.

**Results**

In Part 1, excluding the lowest dose level (5 million viable DCs) where no responses were seen (n=6), at the second and third dose level (10 and 20 million viable DCs, n=6 each), 45% (5/11 evaluable patients) had clinical benefit, including one CR lasting 89 weeks (BRCA WT high grade serous ovarian cancer), one PR lasting 24 weeks (gastric adenocarcinoma), and 3 cases of stable disease (1 ovarian carcinosarcoma and 2 colon cancers). The patient with a CR (HER2 IHC score 3+ and FISH HER2/CEP17 ratio 1.3) recurred with HER2 IHC score 0 and FISH HER2/CEP17 ratio 1.2 metastasis, and the patient with ovarian carcinosarcoma (HER2 IHC score 1+ and FISH HER2/CEP17 ratio 1.0) who had initial tumor shrinkage (-24.8%) progressed with new lesions showing HER2 IHC score 0 and FISH HER2/CEP17 ratio 1.2, suggesting immune escape. Adverse reactions were mainly Grade 1 injection-site reactions. Serial echocardiograms showed preserved cardiac function. Currently, dose-expansion cohorts (40 million viable DCs) in patients who are anti-HER2 therapy naïve (Part 1) or who progressed after standard-of-care anti-HER2 therapy (Part 2) are ongoing.

**Conclusions**

We have translated a cancer vaccine from mice to human clinical trials with promising early results, and intend to combine this vaccine with checkpoint inhibitors, as vaccines can induce T cell responses, turning “cold” tumors into “hot” ones. This combination strategy can potentially improve the response rate and effectiveness.

**Ethics Approval**

The study was approved by IRB, National Cancer Institute, National Institute of Health, project number P12990, protocol 13C0016

#### P174 A phase 1/2a study to evaluate the safety, tolerability, immunogenicity, and anti-tumor activity of GEN-009 adjuvanted neoantigen vaccine in adult patients with selected solid tumors

##### Roger Cohen^1^, Michael S.Gordon, MD^2^, Wendy Hill^3^, Lisa K McNeil, PhD^3^, Tingting Ge, PhD^3^, Arthur P. DeCillis, MD^3^, Melissa L. Johnson, MD^4^, Mark Awad, MD PhD^5^

###### ^1^University of Pennsylvania, Philadelphia, PA, USA; ^2^Honor Health, Scottsdale, AZ, USA; ^3^Genocea, Cambridge, MA, USA; ^4^The Sarah Cannon Research Institute, Nashville, TN, USA; ^5^Dana-Farber Cancer Institute, Boston, MA, USA

####### **Correspondence:** Arthur P. DeCillis (arthur.decillis@icloud.com)

**Background**

GEN-009 is a personalized adjuvanted neoantigen vaccine for the treatment of patients with solid tumors. Genocea’s proprietary Antigen Lead Acquisition System (ATLAS™) is used to prioritize tumor neoantigens that will be synthesized into peptides for inclusion in GEN-009. ATLAS uses a patient’s peripheral blood T cells and antigen presenting cells to screen for every patient-specific tumor mutation. The ATLAS neoantigen selection is based on a cytokine read-out. Neoantigens prioritized by ATLAS contain an epitope that is recognized by the patient’s T cells and should elicit patient-specific stimulatory CD4 or CD8 T cell responses. Unlike *in silico* models, ATLAS is also able to identify inhibitory neoantigens. Inhibitory neoantigens will be excluded from each patient’s vaccine.

**Methods**

GEN-009-001 is a first-in-human study conducted in three parts. For each patient, neoantigens will be selected by ATLAS, manufactured as synthetic long peptides, and co-administered with poly-ICLC adjuvant on 5 treatment days. The primary objectives in all parts are safety and immunogenicity. T cell responses before and after vaccination will be assessed in peripheral blood mononuclear cells by interferon-gamma/granzyme B FluoroSpot assay. Part A (n~9) is enrolling patients with melanoma, NSCLC, SCCHN or urothelial carcinoma who have completed treatment with curative intent and are without disease. Part B will enroll patients in 5 disease-specific cohorts (15 patients in each cohort including the tumor types noted above and renal cell carcinoma). Patients will initiate treatment with nivolumab monotherapy according to the FDA-approved label. Following ATLAS screening and vaccine production, GEN-009 will be added to the patient’s nivolumab treatment. Antitumor activity will be defined by response improvement with the addition of GEN-009 to nivolumab compared with nivolumab monotherapy as described in (Table 1).Part C will treat patients who have the above-noted tumor types and have previously received standard therapy that included a PD-1 or PD-L1 inhibitor with GEN-009 as a monotherapy. An interim analysis based on response using RECIST 1.1 will be conducted after 15 patients have been analyzed. An additional 25 patients may be enrolled.

**Conclusions**

GEN-009-101 Part A is currently enrolling patients.

**Ethics Approval**

The study was approved by Western Institutional Review Board (WIRB), approval number 1-P210

*Corresponding author email: cecile.chartier@iovance.com61-1.

**Table 1 (abstract P174).**
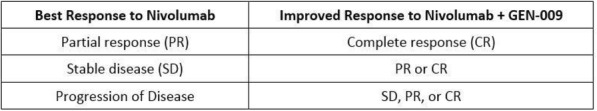
See text for description.

#### P175 Targeting arginase in tumor microenvironment

##### Evelina Martinenaite, MSc, Shamaila Munir Ahmad, Simone Kloch Bendtsen, MSc, Mia Aaboe Jørgensen, Stine Emilie Weis-Banke, Inge Marie Svane, MD, Mads H. Andersen, PhD

###### Center for Cancer Immunotherapy, Herlev, Denmark

####### **Correspondence:** Mads H. Andersen (mads.hald.andersen@regionh.dk)

**Background**

Cancer progression is associated with an increased immune suppression at the tumor site. Arginase-1 is an enzyme expressed by immune inhibitory cells, such as myeloid derived suppressor cells (MDSCs), that reduces arginine availability to the tumor infiltrating immune cells and thus reduces T cell functionality in the tumor milieu. To target arginase-mediated immune inhibition we aimed to identify and characterize T cell responses against arginase-1 derived peptides.

**Methods**

We characterized spontaneous immune responses against optimized 38-mere arginase-1-derived peptide epitope in cancer patients and healthy donors using ex vivo and in vitro IFNγ ELISPOT and intracellular staining for IFNγ and TNFα. T cell responses were further characterized by combining the magnetic bead sorting of CD4+ and CD8+ memory T cells and IFNγ ELISPOT. The natural mechanism of arginase-1 specific T cell activation through upregulation of arginase-1 expression was investigated by IL-4 stimulation.

**Results**

We have previously identified arginase-1 hotspot region that contains multiple peptide epitopes commonly recognized by T cells from cancer patients and healthy donors. We have shown that arginase-specific T cells can be isolated, expanded and are able to recognize arginase-1 expressing immune cells. In this study, we were able to demonstrate that T cells recognizing an optimized 38-mere arginase-1 peptide epitope are a natural part of the T cell repertoire. Strong ex vivo responses against arginase peptide were detected in IFNγ ELISPOT and arginase-1 specific CD4+ and CD8+ memory T cells were found in both healthy donors and cancer patients. We have also shown that arginase-1 specific T cells could be activated by the IL-4-induced upregulation of arginase-1 expression, which suggests the potential role of arginase-specific T cells in the immune regulation.

**Conclusions**

Our study shows that arginase-1 specific CD4+ and CD8+ T cells are a natural part of the immune system, which makes vaccination using arginase-1 derived peptides a promising approach to effectively target arginase producing tumors and inhibitory immune cells such as M2 macrophages and MDSCs in the cancer microenvironment.

#### P176 Identification and analysis of critical immune molecules and signaling pathways to improve the immunogenicity of dendritic cell vaccines

##### Deena Maurer, MS^1^, Deena Maurer, MS^1^, Deena Maurer, MS^1^, Patricia Santos, PhD^2^, John Kirkwood, MD^2^, Ahmad Tarhini, MD, PhD^3^, Hussein Tawbi, MD, PhD^4^, David Stroncek, MD^5^, Ping Jin, PhD^5^, Lisa H. Butterfield, PhD^2^

###### ^1^University of Pittsburgh, Pittsburgh, PA, USA; ^2^UPMC Hillman Cancer Center, Pittsburgh, PA, USA; ^3^Case Western Reserve University, Cleveland, OH, USA; ^4^MD Anderson Cancer Center, Houston, TX, USA; ^5^National Institutes of Health, Bethesda, MD, USA

####### **Correspondence:** Deena Maurer; Lisa H. Butterfield (butterfieldl@upmc.edu)

**Background**

Melanoma is the leading cause of skin cancer-related death in the USA. Clinical trials have revealed important successes with checkpoint blockade treatments. However, many patients do not respond, and those who do often have previously mounted an immune response.

**Methods**

To elicit a strong antitumor immune response, we created a dendritic cell (DC) vaccine loaded with three full-length tumor-associated antigens: Tyrosinase, MART-1, and MAGE-A6 (TMM2). DC were generated from patient monocytes, matured with IFNγ + LPS, and transduced with a recombinant adenovirus encoding these antigens. Patients received three intradermal injections of the vaccine. Human genome RNA microarrays were used to analyze the gene expression profiles of the DC vaccines at immature (day 5 iDC), mature (day 6 mDC), and antigen loaded (day 7 previously matured AdVTMM2 DC) stages.

**Results**

To understand the mechanisms of the DC based vaccine induced immune and clinical responses, microarray gene expression profiles from patients are being investigated. We are examining costimulatory and immune checkpoint molecules and their downstream signaling pathways that are induced upon maturation and/or transduction.Preliminary protein data on 32 patients reveal a decrease in the surface expression of the costimulatory molecule, ICOSL, post maturation and viral transduction, compared to healthy donors (HD). ICOSL protein expression positively correlates with antitumor T cell responses specific to TMM2. ICOSL is reported to be regulated by NFĸB [1]. Analysis of the microarray data revealed that NFĸB signaling is predicted to be inactivated in patient mDC. mRNA expression of the reported NFKB inhibitor, NLRP2, negatively correlates with clinical outcome.Microarray data on a similar HD dataset showed that several metabolic pathways (oxidative phosphorylation and fatty acid beta-oxidation) are inactivated in HD mDC [2]. Inactivation of these pathways at the mDC stage are associated with an immune stimulatory phenotype. Patient data does not show these pathways are being inactivated in mDC. The mRNA expression of genes involved in these pathways are decreased post maturation, but not significantly, and correlate with clinical response.

**Conclusions**

The decrease in ICOSL protein expression observed after maturation and/or transduction may influence the antitumor immune response. In addition, our microarray data suggests that melanoma patient DC may not be metabolically “fit” to generate an optimal antitumor immune response. By understanding the mechanisms in DC, we will be able to generate superior DC-based vaccines and combination therapies, that may lead to improved outcomes for advanced melanoma patients.

**Trial Registration**

This study is related to the Clinical Trial, Multiple Antigen-Engineered DC Vaccine for Melanoma, identifier number NCT01622933.

**References**

1. u H, Wu X, Jin W, Chang M, Cheng X, Sun SC. Noncanonical NF-kappaB regulates inducible costimulator (ICOS) ligand expression and T follicular helper cell development. Proc Natl Acad Sci U S A. 2011; 108:12827– 12832.

2. Jin P, et al. Molecular signatures of maturing dendritic cells: implications for testing the quality of dendritic cell therapies. J. Transl. Med. 2010;8:4. doi: 10.1186/1479-5876-8-4.

**Ethics Approval**

This study was approved by the University of Pittsburgh Institution's Ethics Board, approval numbers 0403105 (UPCI #09-021) and 12010416 (UPCI #04-001).

#### P177 Boosting T cell immunity against IDO1: Proof of concept for a novel cancer vaccine approach

##### Souvik Dey, PhD^1^, Souvik Dey, PhD^1^, Souvik Dey, PhD^1^, Erika Sutanto-Ward, BS^1^, Katharina Kopp, BS^2^, Mai- Britt Zocca, PhD^2^, Ayako Pedersen, PhD^2^, Mads Andersen, PhD^3^, Alexander J. Muller, PhD^1^

###### ^1^Lankenau Institute for Medical Research, Wynnewood, PA, USA; ^2^IO Biotech ApS, Copenhagen, Denmark; ^3^University of Copenhagen, Herlev, Denmark

####### **Correspondence:** Alexander J. Muller (mullera@mlhs.org)

**Background**

IDO1 (indoleamine 2,3-dioxygenase 1) is a tryptophan-catabolizing enzyme that fosters a tumor-promoting inflammatory microenvironment. IDO1 acts to subvert T cell immunity at multiple levels, including suppressing effector T cells and inducing/activating regulatory T cells. In this context, a particularly intriguing finding has been the observation that humans exhibit T cell reactivity against IDO1-expressing cells. IDO1-reactive T cells are found not only in cancer patients, where pathophysiological elevation of IDO1 has been frequently noted, but even in healthy individuals. This finding implies the existence of a T cell-mediated, counter-regulatory mechanism directed against IDO1. Initial clinical studies employing a peptide-based vaccine approach to harness this anti-IDO1 response for the benefit of cancer patients have thus far been encouraging. However, there is a pressing need to establish preclinical models for investigating the underlying basis of the anti-IDO1 response.

**Methods**

See Figure Legends.

**Results**

The CT26 colon carcinoma model was chosen for these studies based on high levels of IDO1 expression and responsiveness to IDO1 inhibition reported for these tumors. Predicted H2d MHC class I and II-restricted IDO1 peptide sequences, identified by computer algorithms, were selected so as to be compatible with the Balb/c strain- based CT26 model. Prophylactic vaccination identified a subset of these peptides that caused tumor growth suppression, including a class II-directed as well as class I-directed peptides. Therapeutic treatment of established CT26 tumors with a combination of anti-PD-1 antibody and class I, but not class II, peptide vaccine produced a combinatorial anti-tumor response beyond what was achieved with either agent alone. Interestingly, the combination of class I and class II peptides likewise elicited an enhanced combinatorial response, suggesting distinct mechanisms of action. Consistent with this interpretation, adoptive transfer of isolated CD8 and CD4 T cells from vaccinated responder mice reciprocally delayed growth of established tumors, such that CD8 (but not CD4) T cells were effective from a class I-vaccinated mouse while CD4 (but not CD8) T cells were effective from a class II-vaccinated mouse. Studies into the underlying nature of the IDO1-directed, anti-tumor response are currently ongoing.

**Conclusions**

As noted in humans, our results demonstrate that IDO1 is immunogenic in mice confirming that T cell responses against this endogenous protein are not fully tolerized. Initial findings that IDO1 peptide-derived vaccines can elicit effective anti-tumor responses demonstrate the utility of mouse models for further exploration and refinement of this novel approach to IDO1-directed cancer therapy.

**Ethics Approval**

This study was approved by the Lankenau Institute for Medical Research IACUC; protocol number A04-2097.

#### P178 Identification of shared phosphopeptide tumor targets in colorectal carcinoma for novel off-the-shelf vaccine development

##### Paisley T. Myers, PhD^1^, Erin D. Jeffery, PhD^1^, Benjamin Morin, PhD^1^, Christine Brittsan, MA^2^, Bishnu Joshi^1^, Antoine Tanne, PhD^1^, Karen Smith, BS^1^, Albert Hurwitz^1^, Kara L. Cummings, PhD^3^, Amanda M. Lulu^3^, Daniel L. Levey, PhD^1^, Linh Mach^1^, Mark A. Findeis, PhD^1^, Gayle C. Hunt, PhD^3^, Jeffrey Shabanowitz, PhD^4^, Donald F. Hunt, PhD^4^, Arthur A. Hurwitz^5^, Robert N. Stein, MD PhD^1^, Alex Duncan, PhD^1^, Dennis Underwood, PhD^1^

###### ^1^Agenus Inc., Charlottesville, VA, USA; ^2^Agenus Inc, Lexington, MA, USA; ^3^University of Virginia School of Medicine, Charlottesville, VA, USA; ^4^University of Virginia, Charlottesville, VA, USA; ^5^AgenTus Therapeutics, Inc., Lexington, MA, USA

####### **Correspondence:** Paisley T. Myers (Paisley.Myers@agenusbio.com)

**Background**

The high mortality rate of colorectal cancer (CRC) reflects limitations of current treatment modalities aimed at targeting the disease. Immunotherapy is a promising alternative to traditional chemotherapy, yet its benefit has been limited to patients with high mutational burden. Here, we describe a novel class of post-translationally modified neoantigens – phosphopeptide tumor targets (PTTs) – for use in a CRC vaccine. PTTs arise from dysregulated cellular signaling, are presented by HLA class I molecules, and are recognized as antigenic by circulating cytotoxic T cells. PTTs are highly prevalent among CRC patients, illustrating their role as excellent targets for the development of novel immunotherapeutics.

**Methods**

HLA:peptide complexes were immunopurified from CRC tissues using pan-HLA class I antibodies. Associated peptides were acid-eluted, concentrated, and enriched via immobilized metal affinity chromatography. Enriched phosphopeptides were analyzed on an Orbitrap Fusion Lumos mass spectrometer using complementary fragmentation methods and sequenced via Byonic™ software. Immunogenicity of PTTs was assessed by in vitro simulation of T cells derived from healthy donors.

**Results**

We applied our workflow to 26 CRC patient tissues. The patient population represents primary and metastatic tumors, microsatellite instable and stable classifications, and numerous HLA alleles, allowing for a broad assessment of PTT neoantigens associated with CRC. We identified over 500 unique PTTs, ranging from less than 1 to 100 copies per cell, with assignments to over 20 HLA alleles, highlighting the robust and sensitive nature of our ligandomic approach. Stringent selection criteria were employed to select strong candidates for inclusion in a CRC PTT vaccine aimed at targeting large patient populations. Thirty PTTs were selected based on 1) assignment to frequent HLA alleles, 2) derivation from proteins involved in dysregulated signaling pathways associated with malignancy, and 3) observation of memory T cell responses in healthy individuals. Based on frequencies of expression of prioritized HLA alleles, 70% of US/EU CRC patients would be eligible for vaccination and the majority of eligible patients’ tumors will contain multiple PTTs included in the vaccine.

**Conclusions**

The development of a novel PTT-based vaccine for CRC may improve survival outcomes compared to standard-of-care treatments. The inclusion of highly-prevalent, multiple allele-associated epitopes from diverse source proteins expands the eligible patient population and increases the likelihood of stimulating an effective, anti-tumor immune response. Furthermore, the shared frequency observed in the PTT repertoire allows for advancement towards the development of an off-the-shelf cancer vaccine with broad therapeutic potential within CRC regardless of tumor mutational burden.

#### P179 Use of Jurkat T cells for high throughput screening of chimeric antigen receptor constructs

##### Tina Nguyen, PhD, Scott McComb, PhD, Risini Weeratna, PhD, Tina Nguyen, PhD

###### National Research Council of Canada, Ottawa, Canada

####### **Correspondence:** Scott McComb (Scott.McComb@nrc-cnrc.gc.ca)

**Background**

Developing successful CAR T therapy requires identification of tumor targeting moieties that can recognize tumors with high degree of specificity with none to minimal auto activation of T cells. Easy, accurate, and rapid high throughput in vitro screening assays are necessary to help select lead candidates for further development of chimeric antigen receptor (CAR) T therapy.

**Methods**

Our model simplifies the workflow of testing by utilizing transient transfection of widely available Jurkat cells.

Using electroporation, rather than relying on lentiviral transduction of CAR expression plasmids, we have developed a high throughput flow cytometry based assay wherein Jurkat CAR cells are exposed to human tumour cell lines with and without cognate target antigens. CAR activation can be readily observed for of via CD69 expression within 6 hours and remains stable for up to 24 hours post activation. We provide data from a number of validation assays, including testing with varying target cells lines, CAR constructs carrying varying signaling domains, and CAR constructs consisting of ScFv and single domain antibodies targeting different tumor specific and tumor-associated antigens.

**Results**

Using our screening method, we were able to identify lead candidate CARs targeting EGFRvIII, HER2, and EGFRWT with strong potential for pre-clinical development. Testing with CAR constructs carrying various signalling domains (41BB, CD28, 41BB/CD28) indicates that while providing information as to the autoactivation and specificity of the extracellular domain of CARs this model does not necessarily provide insight as to the strength or quality of activation signalling.

**Conclusions**

Thus, we developed a tool made for enabling the discovery of lead candidate tumour target binding moieties suitable for CAR-T application.

#### P180 From infectious disease to personalized cancer vaccines: Integrated CD4, CD8, and regulatory T cell neo- epitope screening platform for the design of safe and customized vaccines

##### Guilhem Richard, PhD^1^, Randy F. Sweis, MD^3^, Leonard Moise, PhD^2^, Matthew Ardito, BA^2^, William Martin, BA MD^2^, Gad Berdugo, MSc, MBA^4^, Gary D. Steinberg, MD^3^, Anne S. de Groot, MD^1^

###### ^1^EpiVax Inc., Providence, RI, USA; ^2^EpiVax, Inc., Providence, RI, USA; ^3^University of Chicago, Chicago, IL, USA; ^4^EpiVax Oncology, Inc., Providence, RI, USA

####### **Correspondence:** Anne S. de Groot (annied@epivax.com)

**Background**

Precision cancer immunotherapy has proven to effectively control the tumor of patients in multiple clinical trials.

However, the selection of immunogenic T cell neo-epitopes using traditional methodologies remains a challenging exercise. Poor vaccine performance may partially be due to inclusion of mutated epitopes cross-conserved with self- epitopes recognized by regulatory (Treg), anergic, or deleted T cells. In addition, most cancer vaccine studies focus on the selection of CD8 T cell neo-epitopes while overlooking CD4 T cell neo-epitopes.

**Methods**

We have developed Ancer, an integrated and streamlined neo-epitope selection pipeline, that accelerates the selection of both CD4 and CD8 T cell neo-epitopes. Ancer leverages EpiMatrix and JanusMatrix, predictive algorithms that have been extensively validated in prospective vaccine studies for infectious diseases [1]. Distinctive features of Ancer are its ability to accurately predict Class II HLA ligands and to identify tolerated or Treg epitopes.

**Results**

Ancer was evaluated on data from the BLCA bladder cancer cohort hosted at The Cancer Genome Atlas (TCGA) database. An initial analysis was carried out on a representative set of eleven patients to derive the best vaccine candidate sequences from high-quality missense mutations. A median number of 24 [interquartile range: 15-64] candidate sequences were generated for each patient under study. This initial analysis demonstrates the capacity of Ancer to define a sufficient number of candidate sequences for vaccinating bladder cancer patients in a precision immunotherapy setting.We also assessed Ancer’s ability to predict patient outcomes on a larger subset of 58 individuals. While the disease-free status of the BLCA patients could not be explained by their tumor mutational burden (AUC=0.55, p-value=0.13), nor by their load of missense mutations (AUC=0.54, p-value=0.17), the number of neo-epitopes highly different from self significantly segregated disease-free patients from patients who recurred or progressed (AUC=0.68, p-value=0.02). These results suggest that defining the number of true neo-epitopes using Ancer may represent a novel biomarker for more robust anti-tumor immune response and higher likelihood of disease-free survival.

**Conclusions**

Our preliminary analysis of the BLCA cohort from the TCGA database showcases the value of Ancer in clinical settings. Our next step will be to investigate whether Ancer-defined neo-epitope load will serve as a biomarker for prognosis and response to therapy in the full BLCA cohort.

**References**

1. Moise L, Gutierrez A, Kibria F, et al. iVAX: An integrated toolkit for the selection and optimization of antigens and the design of epitope-driven vaccines. Hum. Vaccines Immunother. 2015;11:2312–2321.

#### P181 Pseudocowpox virus (PCPV), a potent tumor antigen-independent viral vector for cancer immunotherapy

##### Karola Rittner, PhD, Caroline Tosch, engineer, Thioudellet Christine, Christelle Remy-Ziller, Engineer, Marie- Christine Claudepierre, Ms, Benoit Sansas, Johann Foloppe, Philippe Erbs, Kaïdre Bendjama, Eric Quemeneur, PharmD, PhD

###### Transgene, Illkirch-Graffenstaden, France

####### **Correspondence:** Karola Rittner (rittner@transgene.fr)

**Background**

Viral vectors expressing tumor antigens and/or cytokines have proven to be clinically promising approaches to stimulate anti-tumor immunity and to modulate the tumor microenvironment. However, novel viral strains with improved immunogenic properties are sought. By screening a variety of wild-type poxviridae for their immunostimulant effects, we identified PCPV as a strong inducer of IFN-alpha expression in human PBMCs, with secretion rates up to 1000-fold higher than observed for Modified Vaccinia virus Ankara (MVA) or oncolytic Vaccinia virus (VV). PCPV was also superior to MVA in terms of activation of APCs, and increased CD86 expression in human M2-macrophages, suggesting a shift toward a less suppressive, antigen-presenting phenotype [1]. A recombinant PCPV encoding for the HPV E7 antigen was generated to assess the anti-tumor activity, and immunogenicity in syngeneic murine tumor models TC1, and MC38.

**Methods**

TC1 or MC38 tumors were implanted subcutaneously (sc) in C57BL/6 mice. PCPV-E7 (107 pfu) was injected either intratumorally or intravenously. Immune cells were analyzed both from spleen, or lung, as previously reported [2]. Cytokines and chemokines were analyzed by Luminex after dissociation of either skin or tumor. Tumor infiltration was analyzed after enzymatic dissociation, and TILs enrichment using CD45+ magnetic beads. Subpopulations were identified by flow cytometry.

**Results**

Like MVA-E7, PCPV-E7 induced a strong cellular response, as demonstrated by ELISPOT on splenocytes, and frequency analysis of antigen-specific short-lived effector cells. Noteworthy, PCPV displayed a distinct cytokine/chemokine profile at the site of injection, with increased levels of pro-immune cytokines (IP-10, IFN- gamma, GM-CSF, IL-18, MIP-1alpha, MIP-1beta, IL-12, and IL-6). PCPV-E7 and MVA-E7 displayed similar effect on large TC1 tumors. Interestingly, intratumoral injection of PCPV-E7 into fast-growing E7-negative MC38 tumors led to tumor growth control, and increased survival rates that were never observed with MVA or VV vectors. Unlike VV, PCPV displayed no oncolytic activity on MC38 cells in vitro, thus the effect did not result from direct tumor cell lysis after infection. Analysis of tumor infiltrates showed that PCPV treatment decreased the frequency of Ly6C-positive cell populations, and increased that of Ly6G-positive neutrophils. This observation was associated with an increase of G-CSF concentration in the treated tumors. Depletion experiments are underway to monitor the contribution of either neutrophils, CD4+, or CD8+ T cells, and MC38-specific T cell responses in survivors.

**Conclusions**

Our recent data demonstrate that PCPV represents a promising agent for anti-tumor vaccination, in particular for its intrinsic ability to control tumor growth in a tumor antigen-independent manner.

**References**

1. Rittner et al. AACR 2018, poster LB-287; manuscript in preparation. 2. Remy-Ziller et al. Human Vaccines & Immunotherapeutics 2018, 14(1):140-145.

#### P182 Identification of prevalent immunogenic tumor antigens in microsatellite unstable patients with uterine corpus endometrial carcinoma

##### Vladimir Roudko, PhD^1^, Cansu Cimen Bozkus, PhD^2^, Orfanelli Theofano, MD^2^, Stephanie Blank, MD^2^, Benjamin Greenbaum, PhD^2^, Nina Bhardwaj, MD, PhD^2^

###### ^1^Icahn School of Medicine at Mount Sinai Hospital, New York, NY, USA; ^2^Icahn School of Medicine at Mount Sinai, New York, NY, USA

####### **Correspondence:** Vladimir Roudko (vladimir.roudko@mssm.edu)

**Background**

Personalized vaccines using “neoantigens” that arise from tumor-specific genomic alterations are gaining momentum as a new approach of immunotherapy and are currently in evaluation in multiple clinical trials. However, these tumor antigens are highly personalized and require patient specific sequencing approaches for their identification. Here we report on a new computational pipeline, called UniVac (Universal Vaccine), that identifies highly frequent shared tumor epitopes. We applied our approach to microsatellite unstable (MSI-H) patient cohorts from The Cancer Genome Atlas (TCGA) as a proof-of-concept to determine if such shared antigens could be identified and be immunogenic.

**Methods**

A computational analysis of MSI-H patients from TCGA cohort, comprising 332 subjects was undertaken. The set of custom scripts were written in R and python, suitable for execution under UNIX environment. Immunogenicity of selected neopeptides were validated by standard immunological experiments, in which T cells were stimulated in vitro by overlapping long peptides spanning each neopeptide. T cell responses were determined by enzyme-linked immunospot assay (ELISPOT) and intracellular staining.

**Results**

We identified highly frequent, prevalent peptides, encoding MHC-I epitopes that originated from frameshift mutations in the MSI-H cohort of endometrial, colorectal and stomach tumors: 9, 34 and 33 peptides, respectively. In particular, we determined that the epitopes derived from 9 shared endometrial peptides are predicted to bind to the most frequent HLA alleles, thus targeting ~80% of all endometrial patient HLAome. The average frequency of the original 9 frameshifts in each tumor is estimated at ~40% rate, indicating a wide presence of those mutations in patients’ tumors. Moreover, the frameshift load does not affect gene expression, as revealed by transcriptome analysis. We validated common 9 frameshift peptides from endometrial patients in an array of immunological experiments. First, we tested them in T cell stimulation assays using peripheral blood mononuclear cells (PBMCs) isolated from healthy donors. Majority of peptides induced CD8+ T cell responses, as determined by TNF-alpha and IFN-gamma production. Next, we tested whether we could elicit neopeptide-specific T responses in MSI-H endometrial cancer patients. T cell responses were also observed in some patients. Selected endometrial shared frameshift peptides appear to be clonal and prevalently found in MSI-H patients, thus laying a foundation for a shared antigen cancer vaccine design.

**Conclusions**

We developed a computational pipeline to predict the most frequent and shared peptides across patients with MSI-H tumors.

#### P183 Immunogenicity and specificity of neoantigens derived from tumor-specific mutations in gastric cancer

##### Tetsuro Sasada, MD, PhD, Junya Ohtake, Susumu Iizumi, Taku Kouro, MD, PhD, Yuka Igarashi, Mamoru Kawahara, Erika Yada

###### Kanagawa Cancer Center, Yokohama, Japan

####### **Correspondence:** Tetsuro Sasada (tsasada@kcch.jp)

**Background**

Neoantigens derived from tumor-specific genetic mutations can be recognized as foreign by the host immune system, and might be suitable target for cancer immunotherapy possibly due to their higher immunogenicity. In this study, to know the immunogenicity and specificity of tumor-specific neoantigens, we comprehensively examined T cell responses against neoantigens derived from genetic mutations in gastric cancer.

**Methods**

Missense mutations were identified in tumor cells from two gastric cancer patients by using next-generation sequencing. Amino acid sequences, which were derived from the identified mutations and predicted to bind to HLA-class I (A*0201, A*0206, or A*2402), were selected by an epitope prediction server, IEBD. Long peptides (27- mer), in which the mutated sequences were located in the center, were synthesized. Peripheral blood mononuclear cells (PBMC) from healthy donors were cultured in the presence of the synthetic peptides and antigen-specific T cell responses were evaluated by cytokine production assay.

**Results**

Using next-generation sequencing, 156 missense mutations were identified in two gastric cancer patients. From them, 30 potentially immunogenic peptide sequences derived from the identified mutations were selected. In the analysis with PBMC from 18 healthy donors, 27/30 (90%) synthetic peptides showed an ability to induce antigen- specific T cell responses in at least one donor, assessed by cytokine production assay. Among them, 15 peptides were immunogenic in more than one donor. The antigen-specific responses in CD4+ T cells (70%) were observed more frequently than those in CD8+ T cells (43%). Most of the mutated peptides were shown to induce much higher antigen-specific T cell responses than the corresponding wild type peptides. The specificity of T cell responses to mutated sequences, but not to the corresponding wild type sequences, were confirmed in 5 of 8 (62%) peptides examined.

**Conclusions**

Our findings demonstrated high immunogenicity and specificity of neoantigens derived from tumor-specific genetic mutations. In addition, PBMC from healthy donors were suggested to be useful for assessing the immunogenicity of neoantigens derived from cancer patients. Further studies would be recommended to develop a novel immunotherapeutic approach, “personalized cancer vaccination”, targeting mutation-derived neoantigens in gastric cancer.

**Ethics Approval**

This study was approved by the Institutional Review Board of Kanagawa Cancer Center. Informed consents for the study were obtained from all participants.

#### P184 High tumour burden mandates a cancer vaccine targeting many neoantigens combined with check point inhibition

##### Elisa Scarselli, MD^1^, Anna Morena D'Alise^1^, Guido Leoni^1^, Gabriella Cotugno^1^, Fulvia Troise, Dr^1^, Francesca Langone^1^, Imma Fichera, Maria De Lucia^1^, Rosa Vitale^1^, Adriano Leuzzi^1^, Veronica Bignone, Elena Di Matteo^1^, Fabio Tucci^1^, Lidia Avalle^2^, Valeria Poli, Professor^2^, Armin Lahm^1^, Maria Teresa Catanese^1^, Antonella Folgori^1^, Stefano Colloca, Mr^1^, Alfredo Nicosia^1^

###### ^1^Nouscom srl, Rome, Italy; ^2^University of Turin, Turin, Italy

####### **Correspondence:** Elisa Scarselli (e.scarselli@nouscom.com)

**Background**

Neoantigens (nAgs) are a promising class of tumor antigens for cancer vaccination. with the potential of inducing robust and selective anti-tumor T cell responses. Adenoviruses derived from non-human Great Apes (GAds) represent novel genetic vaccines inducing potent cell-mediated immunity. Here, we developed a novel neoantigens vaccine approach based on the use of viral vectors, Great Apes Adenovirus (GAd), encoding multiple cancer neo- epitopes in tandem.

**Methods**

Balb/c mice were implanted either subcutaneously or intravenously with CT26 colon carcinoma cells. In early therapeutic vaccination, mice were vaccinated with a single intramuscular injection of GAd encoding CT26 neoantigens 3 days after cell inoculum. In advanced therapeutic setting, combined treatment of GAd and anti-PD1 started on established subcutaneous tumors (70-100 mm3). Tumor growth was monitored after vaccination and neo- antigen specific T cells were measured both in the periphery and in the tumors. DNA and RNA was extracted from tumors under treatment for NGS analysis of exome and transcriptome.

**Results**

Prophylactic or early therapeutic vaccination with GAds encoding nAgs in mice induced CD8+ and CD4+ T cells and efficiently controlled tumor growth, irrespective of the number of encoded nAgs. In presence of high tumor burden, GAd has no anti-tumor effect unless combined with anti-PD1 treatment. Moreover, in the presence of high tumor burden effectiveness of vaccination required a vaccine encoding many neoantigens. An increased breadth of T cell immune response was measured by IFN-γ ELISpot analysis in mice cured by the combined treatment. Finally, transcriptome analysis of unique TCR beta CDR3 sequences, revealed presence of an increased number of clonotypes in tumor biopsies of combo treated animals compared to anti-PD-1.

**Conclusions**

Vaccination with Great Apes Adenovirus (GAd) encoding neoantigens is very effective to strengthen and broaden T cells against tumor neoantigens. Growth of vaccine-induced T cells in established tumours can be successfully achieved with a vaccine encoding many neoantigens combined with the administration of anti-PD-1. The data presented here warrant further testing of cancer vaccines based on GAd into the clinic supporting their use as stand- alone treatment in tumor prevention or in minimal residual disease/adjuvant clinical setting. Conversely, in the presence of established tumors, combination with check-point inhibition is required.

#### P185 CUE-101, a novel Fc fusion protein comprised of HLA-A*0201-bound HPV16 E7 peptide and IL-2, for selective targeting and expansion of anti-tumor T cells for treatment of HPV-driven malignancies

##### Mary C. Simcox, PhD^1^, Steven Quayle^1^, Dharma R. Thapa, PhD^1^, Sandrine Hulot, PhD^1^, Alyssa Nelson, BS^1^, Lauren Kraemer, BS^1^, Zohra Merazga, MS^1^, Robert Ruidera, MSc^1^, Dominic Beal, PhD^1^, Gurpanna Saggu, PhD^1^, Maria Hackett, MSc^1^, Mark Haydock, BS^1^, Jonathan Soriano, MSc^1^, Joey Lee^1^, Luke Witt, BS^1^, Kelly Malone^1^, Jessica Ryabin, BS^1^, Simon Low, MSc^1^, Natasha Girgis, PhD^1^, Emily Spaulding, PhD^1^, John Ross, PhD^1^, Anish Suri, PhD^1^, Rodolfo Chaparro, PhD^1^, Ronald Seidel, PhD^1^, Kenneth Pienta, MD^2^

###### ^1^Cue Biopharma, Cambridge, MA, USA; ^2^Johns Hopkins School of Medicine, Baltimore, MD, USA

####### **Correspondence:** Mary C. Simcox (mesimcox@cuebio.com)

**Background**

Human papilloma virus (HPV) is responsible for 72% of oropharyngeal,70% of cervical, 90% of anal and 71% of vulvar, vaginal, or penile cancers, causing significant morbidity and mortality worldwide [1,2].CUE-101, a novel fusion protein designed to activate tumor antigen-specific T cells to treat HPV16-driven cancers, is comprised of a human leukocyte antigen (HLA) complex, HLA-A*0201, a peptide epitope derived from the human HPV16 E7 protein (amino acid residues 11-20), a reduced affinity human interleukin-2 (IL-2) variant, and an effector attenuated human immunoglobulin G (IgG1) Fc domain.

**Methods**

CUE-101 cellular binding, specificity, TCR- and IL-2 receptor (IL-2R)-induced signaling, and induction of activation and cytotoxic T lymphocyte markers were measured using flow cytometry with human E7-specific T cells (Astarte Biologics, Bothell, WA). Enzyme-Linked ImmunoSpot (ELISPOT) assays were performed to measure peptide-specific secretion of interferon gamma (IFNg). Anti-tumor efficacy with a murine surrogate molecule was assessed in the TC-1 model [3], and tumor antigen-specific T cell expansion in vivo was assessed via tetramer staining.

**Results**

CUE-101 binds to HPV16-E7–specific CD8 T cells (EC50 = 10-20 nM) and activates signal transduction downstream of TCR and IL-2R, whereas no activation of TCR signaling was observed upon CUE-101 treatment of bulk CD8 T cells or with a CUE-101 analog presenting a CMV peptide. Upon binding and receptor engagement, CUE-101 induces potent and dose-dependent secretion of IFNg (EC50 = 0.69 nM). Effector cytokine secretion is dependent on the peptide specificity of the HLA complex, and an analog lacking the IL-2 moieties exhibited >100- fold reduction in potency. Pharmacokinetic (PK) data in rats and monkeys suggest that exposures sufficient to promote antigen-specific T cell activation and expansion will be achievable in humans. In vivo, a murine surrogate of CUE-101 demonstrates anti-tumor activity both as a monotherapy and in combination with an anti-PD-1 antibody in the TC-1 E6/E7+ tumor model. Efficacy in this model was associated with expansion of HPV16 E7 reactive T cells, and establishment of immunologic memory was demonstrated via tumor rejection upon rechallenge with TC-1 cells in absence of any additional administration of the murine surrogate.

**Conclusions**

CUE-101 demonstrates selective binding, activation, and expansion of HPV16 E7-specific CD8+ T cells in vitro and a favorable PK profile in animals. A murine surrogate of CUE-101 exhibits anti-tumor efficacy both as a monotherapy and in combination with anti-PD-1. These data support the potential for CUE-101 to enhance anti-tumor immunity in patients with HPV16-driven malignancies.

**References**

1. de Sanjosé S, Diaz M, Castellsagué X, et al. Worldwide prevalence and genotype distribution of cervical human papillomavirus DNA in women with normal cytology: a meta-analysis. Lancet Infect Dis. 2007;7:453-459. 2. Frazer IH. Prevention of cervical cancer through papillomavirus vaccination. Nat Rev Immunol. 2004;4(1):46-54 3. Lin K, Guarnieri F, Staveley-O'Carroll K, Levitsky H, August J, Pardoll D, Wu T. Treatment of established tumors with a novel vaccine that enhances major histocompatibility class II presentation of tumor antigen. Cancer Res. 1996;56:21-26.

**Ethics Approval**

All animal activities and procedures were performed in accordance with the protocols approved by the Institutional Animal Care and Use Committee (IACUC) for ethical review of animal care and use.

#### P186 A multi-center study of hTERT immunotherapy in adults with solid tumors at high risk of relapse post- standard therapy: updated results from complete patient set

##### Anthony Shields, MD PhD^1^, Autumn J. McRee, MD^2^, Jennifer Johnson, MD^3^, Weijing Sun, MD, FACP^4^, Ashish Chintakuntlawar, MBBS, PhD^5^, Naseem Prostak, BS MS^6^, Kimberly Kraynyak, BS, PhD^6^, Matthew P. Morrow, PhD^6^, Jeffrey M. Skolnik, MD^6^, Robert H. Vonderheide, MD, DPhil^8^

###### ^1^Karmanos Cancer Center, Detroit, MI, USA; ^2^UNC Chapel Hill, Chapel Hill, NC, USA; ^3^Thomas Jefferson University, Philadelphia, PA, USA; ^4^University of Pittsburgh, Pittsburgh, PA, USA; ^5^Mayo Clinic, Rochester, MN, USA; ^6^Inovio, Plymouth Meeting, PA, USA; ^7^Inovio Pharmaceuticals Inc, Plymouth Meeting, PA, USA; ^8^University of Pennsylvania, Phildelphia, PA, USA

####### **Correspondence:** Jeffrey M. Skolnik (jeffrey.skolnik@inovio.com)

**Background**

Human telomerase (hTERT) is a reverse transcriptase that recognizes and elongates telomeric DNA ends. hTERT is expressed in up to 90% of cancers, and can be recognized by cytotoxic T cells. As a result, hTERT is an excellent target for T cell-enabling therapy. The administration of optimized full-length DNA sequences followed by electroporation (EP) in vivo has generated potent CD4+ and CD8+ T cell responses against hTERT in preclinical studies. In this phase 1 dose-escalation study, synthetic optimized DNA plasmids that target hTERT (INO-1400, or mutant hTERT; and INO-1401, or SynCon® TERT) were delivered intramuscularly followed by EP with the CELLECTRA® device, to assess safety, tolerability and immune effects of immunotherapy with INO-1400 or INO- 1401, alone or co-administered with a plasma encoding for human IL-12 (INO-9012) in adult patients with cancer.

**Methods**

Following standard of care neoadjuvant or adjuvant chemoradiotherapy and/or surgery, patients with one of 9 solid tumors at high risk of relapse, but without evidence of residual disease, were enrolled to one of 10 arms and received four doses, four weeks apart, of either INO-1400 or INO-1401 alone or in combination with INO-9012 by IM injection, followed by EP. Patients were followed for tolerability, immunogenicity and clinical response.

**Results**

As of 18 July, 2018, the study has completed enrollment and dosing of 93 patients. Doses of 2 or 8 mg of INO-1400/01, and of 0.5 or 2 mg INO-9012 were well-tolerated, with a majority of reported adverse events (AE) being low-grade and related to IM+EP administration. Two related SAEs have been previously reported (breast cellulitis; abdominal pain/elevated lipase), and one dose-limiting toxicity (DLT) has been previously reported (rash). No other reported SAEs or DLTs have been reported for this study. Immunogenicity by ELISpot for antigen-specific interferon-gamma (IFN-g) secreting T cells suggests that patients can generate hTERT-specific T cells across multiple doses, with or without IL-12, and with both INO-1400 and INO-1401. The majority of patients continue on study, with several patients having completed two years of study follow- up.

**Conclusions**

INO-1400/01 with or without INO-9012 given IM with EP is well-tolerated in adults with solid tumors, and can generate active hTERT-specific T cells, suggesting an ability to break immune tolerance. Dosing is complete, and follow-up is ongoing.

**Ethics Approval**

The study was approved by each participating Institutution‘s Ethics or Institutional Review Board(s).

#### P187 Preclinical evidence for the potency and tumor selective activation of a novel EpCAM-CD3 Protease- Triggered Immune Activator (ProTIA) T-cell bispecific therapeutic

##### Tillman Pearce, MD, Bee-Cheng Sim, Desiree Thayer, Fan Yang, Tillman Pearce, MD, Zach Lange, Ulrich Ernst, PhD, Volker Schellenberger

###### Amunix, Mountain View, CA, USA

####### **Correspondence:** Volker Schellenberger (vschellenberger@amunix.com)

**Background**

The regulatory approval of blinatumomab (Blincyto) provided proof-of-concept that a bi-specific T cell engager could redirect human T cells (via a CD3 binding site) to act against a hematologic malignancy (via a CD19 binding site). Extending this general strategy to solid tumors is intriguing, especially in immunologically cold tumors; however, the potential for on-target, off-tumor toxicity may limit the feasibility of this approach.Amunix is developing a novel class of molecules called Protease-Triggered Immune Activators, or ProTIA, as a strategy for selective activation of T cells within the tumor microenvironment (Figure 1). ProTIA molecules include single chain Fv fragments targeting both a cell surface antigen overexpressed on tumor cells and the CD3 epsilon molecule on T cells. This core bi-specific molecule is then modified by addition of proprietary recombinant polymers of defined length and sequence but of undefined structure called XTEN polymers. XTEN polymers are linked to the scFvs by a protease-cleavable site. ProTIA provides tumor selectivity by the antigen specificity of the ScFv, reduced penetration into normal tissue via the enhanced permeability and retention effect, and selective activation by tumor associated proteases.

**Methods**

An EpCAM-CD3 bispecific ProTIA was constructed with or without a tumor protease cleavable sequence (ProTIA-X vs. ProTIA-B), along with a pre-activated form without XTEN (active ProTIA, or ProTIA-A). In vitro cytotoxicity assays were performed using several cancer target cells and human PBMC effector cells. In vivo efficacy experiments in NOG mouse models were performed by implanting colorectal HCT-116 cells, then injecting human PBMC and test article when tumor was established. A mouse surrogate EpCAM-CD3 ProTIA was also constructed and assessed in vivo for on-target, off-tumor toxicity.

**Results**

In vivo, both ProTIA-A and ProTIA-X treatment, but not ProTIA-B treatment, resulted in tumor regression (Figure 2) with no adverse effect on body weight (effect on weight not shown). The mouse surrogate EpCAM-CD3 ProTIA- X had a 10-fold improved tolerability in C57BL/6 mice compared with the non-protected bispecific (ProTIA-A) (Figure 3).

**Conclusions**

T-cell redirecting therapies have great therapeutic potential for immunologically cold solid tumors or checkpoint inhibitor resistant tumors which have lost the capacity for cell surface neoantigen presentation, but pose a significant risk for on-target, off-tumor toxicity. These experiments confirmed that a novel ProTIA EpCAM-CD3 has similar potency as an unprotected bispecific (ProTIA-A) but with significantly reduced normal tissue toxicity.

**Fig. 1 (abstract P187).**
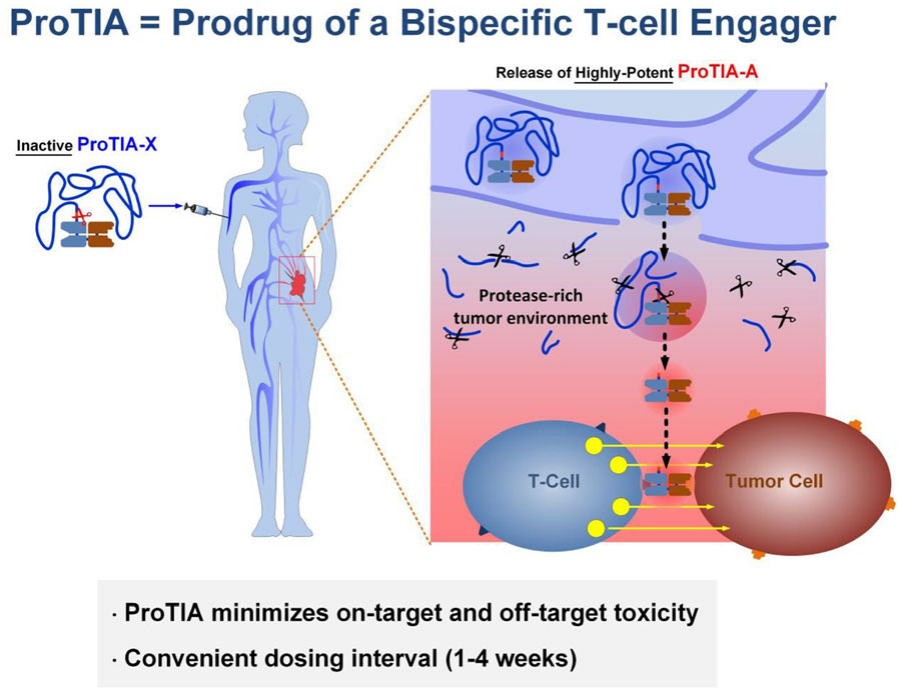
See text for description.

**Fig. 2 (abstract P187).**
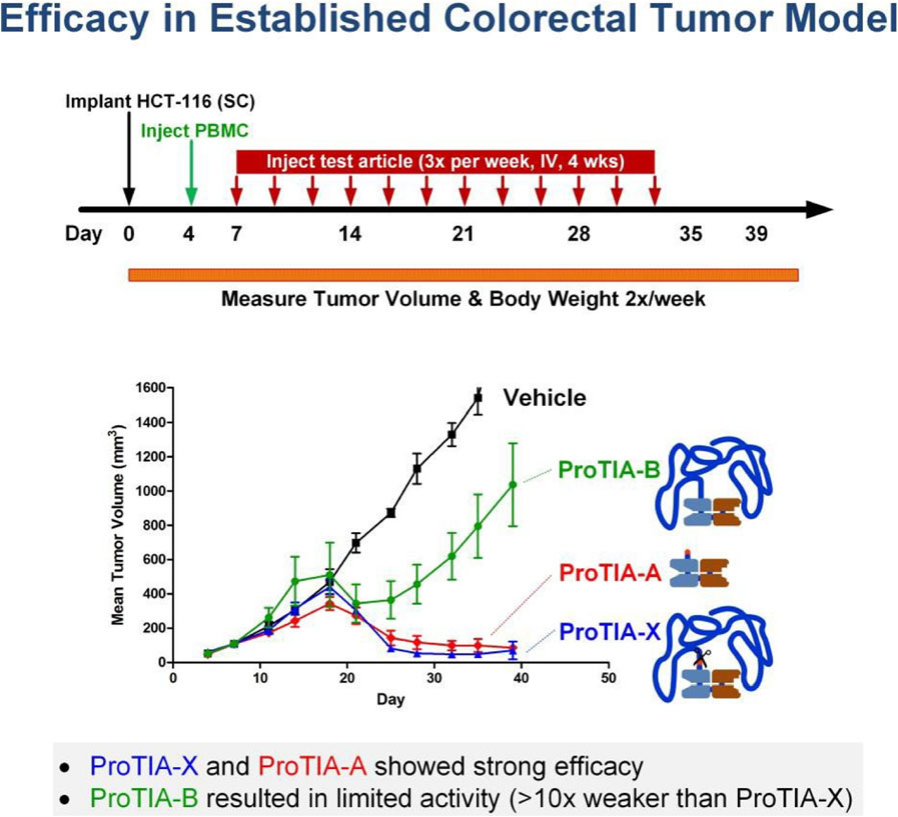
See text for description.

**Fig. 3 (abstract P187).**
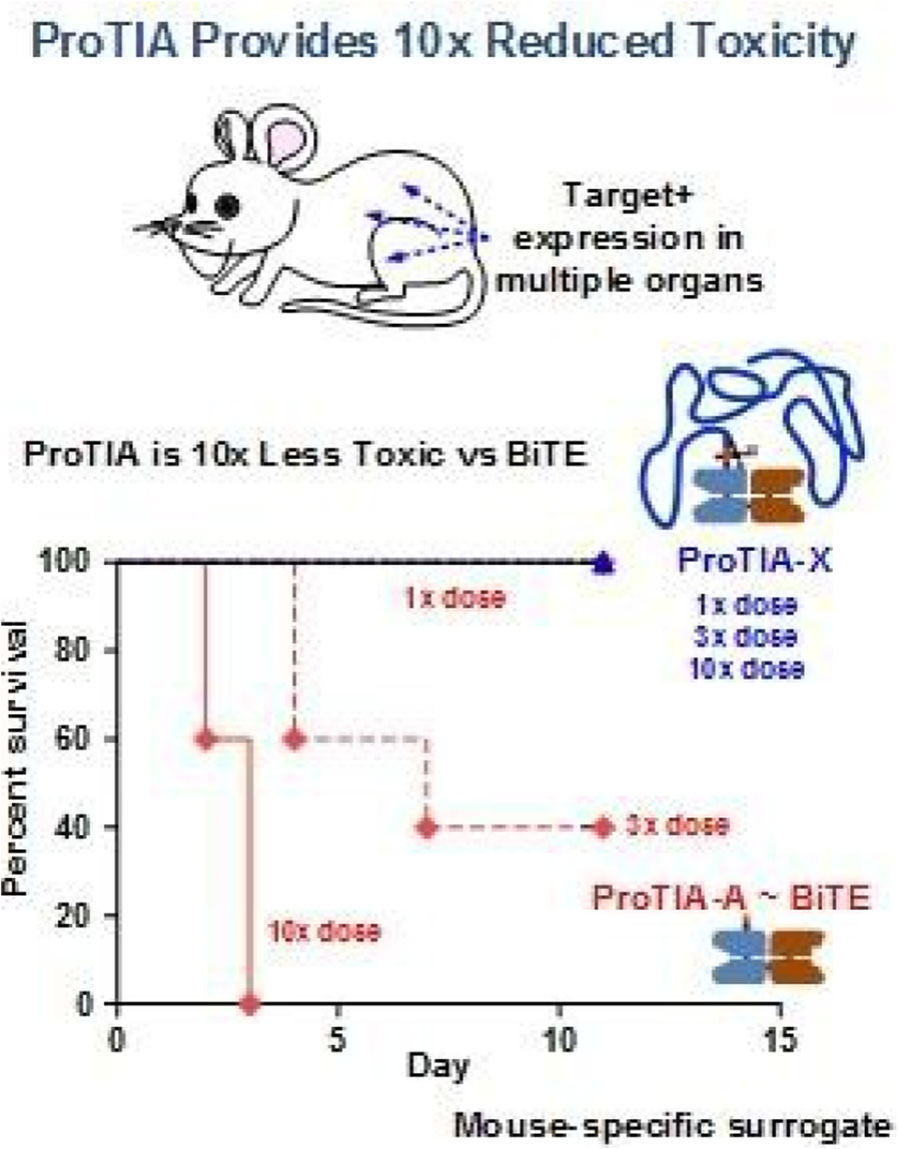
See text for description.

#### P188 Patient’s autologous T cells recognize tumor specific neoantigens in PDX models of ovarian cancer

##### Muzamil Want, PhD^2^, Muzamil Want, PhD^1^, Muzamil Want, PhD^2^, Anna Konstorum, PhD^3^, Ruea-Yea Huang, PhD^1^, Richard Koya, MD, PhD^1^, Sebastian Battaglia, PhD^1^

###### ^1^Roswell Park Comprehensive Cancer Center, Buffalo, NY, USA; ^2^Roswell ParkComprehensive Cancer Center, Buffalo, NY, USA; ^3^UConn Health, Farmington, CT, USA

####### **Correspondence:** Muzamil Want; Sebastian Battaglia (sebastiano.battaglia@roswellpark.org)

**Background**

Ovarian cancer (OC) is the fifth leading cause of cancer death in the USA and has the highest mortality rate of all gynecologic cancers. Since OC patients are often diagnosed at late stage with local and distal metastases, this offers a clinical challenge, with 70% of the patients developing chemoresistant disease. Private cancer neoantigens are derived from somatic mutations and represent an attractive target for ovarian malignancies, allowing for a fully personalized therapeutic approach.

**Methods**

We established patient-derived xenografts (PDX) model from one primary tumor of a Roswell Park ovarian cancer patient by injecting subcutaneously NSG mice with 2x106 cells. Tumor mutational landscape was interrogated in the primary tumor and two passages (P0, P1) via whole exome sequencing (WES) using the patient’s PBMCs as germline control. NetMHC was used to predict affinity to the patient’s HLAs and neoantigens were ranked based on differential binding of the wild type (WT) versus mutated (MUT) peptide. Potential neoantigens T cell activation by neoantigens was tested in vitro via ELISA and flow cytometry and in vivo by using neoantigen activated T cells for adoptive T cell therapy (ACT) infusing 5 x 106 cells intravenously in PDX mice (P1) 12 days post tumor implantation. TCRSeq was performed with 10XGenomics and analyzed with custom R scripts.

**Results**

We identified 184 non-synonymous mutations leading to 30 potential neoantigens with high affinity for the patient’s HLAs. Interferon-gamma production and upregulation of CD137 identified a core set of 6 neoantigens specifically recognized by patient’s autologous CD8+ T cells (Figure 1A-B), 4/6 neoantigens were common between PDX and primary tumor. Patient’s T cells were activated in vitro by PDX tumor lysate (Figure 1C). In vivo ACT studies showed that mice injected with neoantigen-stimulated PBMCs (ACT_MUT) have reduced tumor growth when compared to mice injected with unstimulated patient’s PBMCs (ACT_NP) (Figure 1D). Furthermore, ACT_MUT mice have higher levels of circulating T cells 15 days post-ACT and higher intratumoral T cells at end point than ACT_NP (Figure 1E-F). We then sought to identify the TCR moieties that determine T cell response. TCRSeq analyses on the two strongest neoantigens identified multiple TCR activated by a single cancer neoantigen (Figure 1G), suggesting oligoclonal T cell activation.

**Conclusions**

PDX models reflect the tumor mutational landscape of OC patient and presence of neoantigen reactive T cells in the blood of OC patient can be used to develop a personalized immunotherapeutic approach.

**Acknowledgements**

This work is supported by RPCI-UPCI ovarian cancer SPORE CEP grant. NGS services were provided by Genomics shared resources supported by NCI P30CA16056 and for Cytometry services were provided by the Flow and Image Cytometry Core facility at the Roswell Park Comprehensive Cancer Center which is supported in part by the NCI Cancer Center Support Grant 5P30 CA016056.

**Fig. 1 (abstract P188).**
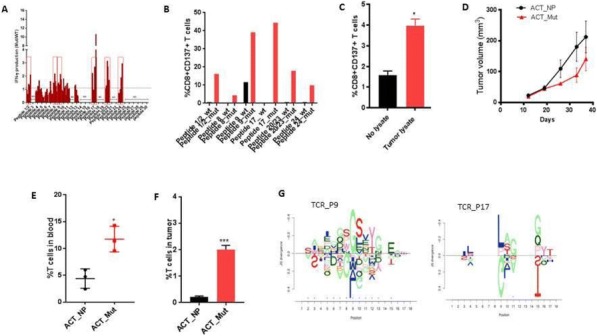
See text for description.

#### P189 A phase 1 study of safety and tolerability of AutoSynVax™ vaccine in patients with advanced cancer

##### Robert Wesolowski, MD^1^, Breelyn M. Wilky, MD^2^, Allison O'Neill^3^, Ana M. Gonzalez, MD PhD^3^, Elise E. Drouin, PhD^3^, Edward Dow^3^, Mohamed Uduman^3^, Antoine J. Tanne^3^, Maria Agarwal, PhD^3^, Bishnu Joshi^3^, Benjamin Morin, PhD^3^, Mark A. Findeis, PhD^3^, Sandra Craig^3^, Igor Proscurshim, MD^3^, Ricardo Soto^3^, Jennifer S. Buell, PhD^3^, Robert Stein, MD PhD^3^, John C. Castle^3^, Daniel L. Levey, PhD^3^

###### ^1^The Ohio State University, Columbus, OH, USA; ^2^Miami University, Miami, FL, USA; ^3^Agenus, Lexington, MA, USA

####### **Correspondence:** Robert Wesolowski (robert.wesolowski@osumc.edu)

**Background**

Agenus AutoSynVax™ (ASV™), AGEN2003, is an individualized, fully synthetic neoantigen vaccine comprised of computationally defined peptide immunogens complexed to recombinant heat shock protein (HSP) and administered with QS-21 Stimulon® adjuvant. In animal models and clinical trials, vaccines employing HSP-peptide complexes mixed with QS-21 elicit antigen-specific CD8+ and CD4+ T-cell responses.

**Methods**

After signing an informed consent form, patients (pts) with advanced cancer, considered incurable/without approved therapies were treated with AGEN2003. The vaccine was administered subcutaneously at 240μg with 50μg QS-21 bi-weekly. Study primary objective was safety and tolerability. Other objectives included objective response rate (ORR), overall survival (OS) and tumor specific immune response. Given the small cohort, data was summarized using descriptive statistics. An Institutional Review Board at each participating site has approved the study.

**Results**

Nine pts were enrolled on the Phase I study, 3 received treatment with vaccine derived from their tumor. Of six enrolled but not treated; 5 progressed prior to treatment. An additional 2 pts (<18 yr) received AGEN2003 through compassionate access (IND 16962). Among the treated pts, no Serious Adverse or grade (G) ≥3 toxicities attributed to vaccine were reported. The most common AE’s (G 1-2) were nausea and myalgia at injection site. One compassionate use pt with metastatic hepatocellular carcinoma, refractory to PD-1 inhibition, has been treated intermittently with AGEN2003 alone or in combination with pembrolizumab for 13 months. Of 3 study pts: 2 (1 with uterine leiomyosarcoma, 1 with inflammatory breast cancer) received 8 doses each; a third pt with pleiomorphic leiomyosarcoma received 3 doses. Neoantigen-immunogenicity was positively associated with survival and as of July 2018, 2 patients (both with a quantifiable immune response) remain alive.

**Conclusions**

AGEN2003 was well tolerated with no serious AE’s attributable to vaccine. We plan to combine future versions of ASV with immunomodulatory antibodies.

**Trial Registration**

NCT02992977

**Ethics Approval**

The study was approved by institutional review board at each participating institution (IRB study # 1174684)

**Consent**

Not applicable as no identifiable information is disclosed.

#### P190 Identification of antigenic epitopes of EGFR for lung cancer vaccine development

##### Juhua Zhou, PhD^1^, Ge Guo^1^, Jianzhong Zhang^2^, Shaoyan Huang^2^, Yanmin Li^1^

###### ^1^Ludong University, Yantai, China; ^2^Yantaishan Hospital, Yantai, China

####### **Correspondence:** Yanmin Li (84562765@qq.com)

**Background**

It has been documented that approximately 85% of the patients with lung cancer are non-small cell lung cancer (NSCLC). The mutation rate of human epidermal growth factor receptor (EGFR) gene is about >60% in NSCLC patients. The mutation loci of EGFR are mostly concentrated in the regions of exons 18-21. In addition, it has been reported that EGFR exon 19 T790M mutation is responsible for drug resistance of more than 90% of NSCLC patients to tyrosine kinase inhibitors. Thus, EGFR is a good candidate for developing non-small cell lung cancer vaccines.

**Methods**

In the current study, 40 peptides with 10 amino acids from EGFR protein were designed and synthesized using Immune Epitope Database (IEDB) online software for the prediction of T cell antigenic epitopes. Reverse immunology method was employed to screen T cell antigenic epitopes from 40 EGFR peptides, which was used to stimulate the expansion and interferon gamma release of peripheral blood mononuclear cells from 30 patients with NSCLC.

**Results**

Three unique peptides of EGFR were identified to be T cell antigenic epitopes due to their ability in the stimulation of cell expansion and cytokine release. Further studies in vivo showed that three unique peptides of EGFR could stimulate anti-lung cancer immune responses in both wild-type mice and NUDE mouse tumor model. The clinical trials of three unique peptides of EGFR as NSCLC cancer vaccines are under way.

**Conclusions**

Three unique peptides of EGFR may be used as cancer vaccines in the treatment of patients with NSCLC.

**Trial Registration**

This work was supported by “Taishan Scholar” special fund (No. tshw20120718) from Shandong Government, China

**Ethics Approval**

The study was approved by Ludong University's Institutional Review Board (IRB), approval number LDU- IRB2015002.

### Case Studies

#### P191 Combination of Sorafenib and anti-PD-1 for advanced hepatocellular carcinoma- real world experience

##### San-chi Chen, MD, Muh-Hwa Yang, Yee Chao

###### Taipei Veterans General Hospital, Taipei, Taiwan, Province of China

####### **Correspondence:** San-chi Chen (ychao@vghtpe.gov.tw)

**Background**

Advanced hepatocellular carcinoma (HCC) portends dismal prognosis. Recently, two anti-PD-1 monoclonal antibodies have demonstrated favorable responses in HCC. Nivolumab was proved for second line treatment based on CheckMate-040, in which nivolumab demonstrated the objective response rate (ORR) of 15% in sorafenib- experienced HCC. Similarly, pembrolizumab also achieved ORR of 16% in Keynote-224. However, there is still an unmet need to achieve a better response. Sorafenib that has been proved for the treatment of HCC for a decade is found to have immune modulation effect. Researches showed that sorafenib inhibited regulated T cells and promoted effector T cells, especially in low dose. These data support the rationale for sorafenib to combine with immunotherapy.

**Methods**

We retrospectively analyzed 43 HCC patients who received the combination therapy of sorafenib and anti-PD-1.

**Results**

Among 43 patients in this cohort, mean age was 63 (+/-12) year-old, 79% were male, 49% were HBV(+) and 21% were HCV(+). The Child-Pugh Score of A, B and C were 51%, 26% and 23%, respectively. Best responses were CR in 3 patients (7%), PR in 9 (21%), SD in 7 (16%) and PD in 15 (35%); the response rate was 28% and disease control rate (DCR) 44%. Patients had no significant difference of response in etiologies, Child-Pugh Score and initiate AFP levels. Eighteen patients (42%) developed grade 1/2 toxicities and 9 (21%) grade 3/4 toxicities including 4 immune hepatitis and three skin rash. Patients who developed grade 1/2 toxicities has ORR of 50% and grade 3/4 toxicities has ORR of 11%. Median PFS in responders and non-responders were 10.5 and 2.0 months, and median OS were 12.7 and 2.0 months, respectively. In univariate analysis, grade 1/2 toxicity (HR 0.35) and grade 3/4 toxicity (HR 5.11) were risk factors for disease progression; Child-Push Score C and BCLC stage D were risk factors for survival.

**Conclusions**

Combination therapy of sorafenib and anti-PD-1 has a favorable response in patients with HCC, event in those who have been previously treated with sorafenib. The adequate dose, synergistic effect and safety of this combination need a large-scale of clinical trial to confirm.

**Ethics Approval**

The study was approved by Taipei Veterans General Hospital‘s Ethics Board, approval number 2017-10-005BC.

#### P192 A case of nivolumab-induced gastrointestinal toxicity treated with vedolizumab in the context of metastatic non-small cell lung cancer

##### Cynthia N. Tran, MD^1^, Yinghong Wang, MD, PhD^2^, Wenyi Luo, MD^2^

###### ^1^Baylor College of Medicine, Houston, TX, USA; ^2^MD Anderson Cancer Center, Houston, TX, USA

####### **Correspondence:** Yinghong Wang (ywang59@mdanderson.org)

**Background**

Immune checkpoint inhibitors have emerged as a novel therapeutic class for a wide variety of malignancies through their action on the immune system. This action promotes significant anti-tumor effect but, simultaneously, can also result in immune-related adverse events (irAEs) that may limit their use [1]. Multiple case reports and case series of lower gastrointestinal irAEs have been reported; however, the data on upper gastrointestinal tract is very sparse [2]. We herein describe a case with severe and steroid-dependent upper gastrointestinal toxicity with nivolumab treatment who eventually achieved clinical and histological remissions with vedolizumab treatment.

**Methods**

N/A

**Results**

A 65 year old male patient with lung cancer initially diagnosed in 2013, previously treated with pemetrexed and carboplatin regimen, was started on Nivolumab since January 2017 for progressive disease involving the left upper lung, retroperitoneal lymphadenopathy, possible liver metastases, and several small brain lesions. Following sixteen cycles of nivolumab, he developed multiple episodes of severe nausea, vomiting, and abdominal cramps requiring five hospitalizations total for dehydration and poor oral intake with associated weightloss. His initial EGD showed active inflammation in both the stomach and duodenum (Figure 1A, B). Due to this severe GI irAE, nivolumab was stopped even though his underlying cancer had demonstrated good response to nivolumab maintenance treatment. He was treated with multiple courses of steroid (intravenous methylprednisolone to oral prednisone) at each hospitalization but always developed symptomatic recurrence on prednisone taper dose at 20 mg/day. Additionally, the patient developed oral thrush and Clostridium difficile infection while on steroids that required antibiotic treatment. Two trials of budesonide were attempted but unsuccessful. After the fifth hospitalization, he was initiated on vedolizumab infusion and achieved clinical remission within two weeks without further requirement of hospitalization or steroids for the following six months. His most recent EGD and biopsy after five doses of vedolizumab demonstrated complete resolution of active inflammation on histologic evaluation (Figure 2A, B). His lung cancer has since relapsed and the treatment plan is to resume nivolumab with concurrent use of vedolizumab.

**Conclusions**

Immune checkpoint inhibitors, such as nivolumab, have emerged as treatment for a variety of malignancies [1].

Their use can be associated with various immune-related adverse events (irAEs) involving the upper gastrointestinal tract which is not commonly seen [2]. This case scenario showed that vedolizumab can provide steroid-sparing therapeutic effect to achieve clinical and histological remission even in cases that failed multiple steroid courses with good safety profile.

**References**

1. Postow M, Callahan M, Wolchock J. Immune checkpoint blockade in cancer therapy. J Clin Oncol. 2015; 33:1974-1982.

2. Abdel-Rahman O, El Halawani H, Fouad M. Risk of gastrointestinal complications in cancer patients treated with immune checkpoint inhibitors: A meta-analysis. Immunotherapy, 2015; 7:1213-1217.

**Ethics Approval**

Institutional Review Board protocol is exempted at the University of Texas MD Anderson Cancer Center for single case report.

**Consent**

This study was granted waiver for consent.

**Fig. 1 (abstract P192).**
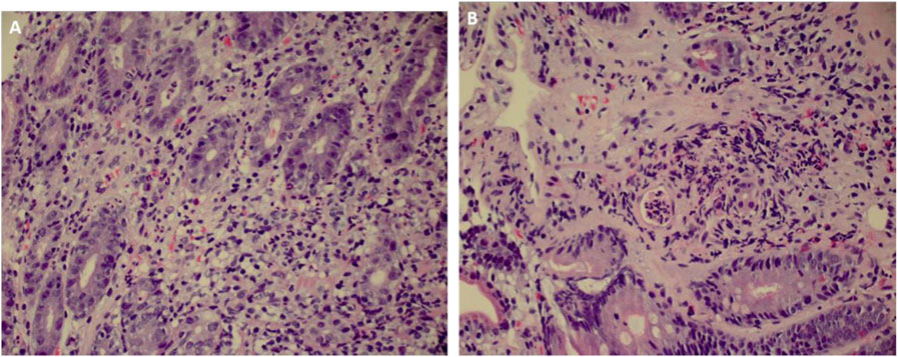
Pathology of stomach and duodenum pre vedolizumab

**Fig. 2 (abstract P192).**
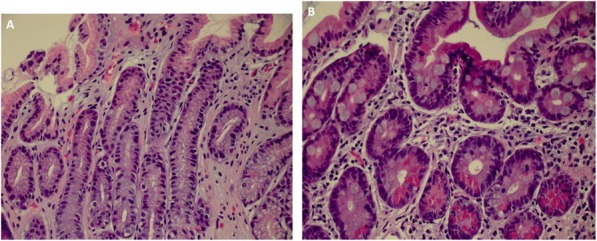
Pathology of stomach and duodenum post vedolizumab

#### P193 Exceptional response of MSH6-null castration-resistant prostate cancer patient with myelophthisic pancytopenia to pembrolizumab

##### Panagiotis Vlachostergios, MD, PhD, Julia T. Geyer, MD, Ana M. Molina, MD, David M. Nanus, MD, Himisha Beltran, MD, Scott T. Tagawa, MD, Panagiotis J. Vlachostergios, MD, PhD

###### Weill Cornell Medicine, New York, NY, USA

####### **Correspondence:** Scott T. Tagawa (stt2007@med.cornell.edu)

**Background**

Metastatic castration-resistant prostate cancer (mCRPC) remains lethal despite recent advances in AR-directed therapies. Myelophthisis is not rare in advanced PC and often limits chemotherapeutic options. The tissue-agnostic activity of the anti-programmed cell death-1 (PD-1) inhibitor pembrolizumab in tumors harboring mismatch-repair (MMR) defects may offer an important therapeutic option in such patients.

**Methods**

We describe a MMR-deficient mCRPC patient with widespread metastases involving the bone marrow causing myelophthisis, who responded to immune checkpoint inhibition with pembrolizumab.

**Results**

A 74 year old man initially presented in 1999 with early prostate cancer, Gleason 4+3 = 7 underwent prostatectomy and salvage radiation but had PSA recurrence 9 years later. He was started on combined androgen blockade with leuprolide and bicalutamide to which he responded (PSA nadir <0.1 ng/ml) for 7 months, but then experienced PSA and radiologic progression with osseous metastases. He received consecutively sipuleucel-T, abiraterone, enzalutamide, 177Lu-J591, Radium-223 and docetaxel for his mCRPC. However he had further disease progression with diffuse osseous, lymph node and visceral (bilateral lung, solitary liver, bilateral adrenal) metastases and a rising PSA level at 2,048 ng/ml. Due to persistent pancytopenia (WBC: 3.2 x103/uL, Hb: 7.5 g/dl, PLT: 30 x103/uL) a bone marrow biopsy was performed. Hematoxylin and eosin staining revealed extensive metastatic PC (PSMA+, synaptophysin+, ERG–). Additionally, whole exome tumor sequencing was performed and disclosed somatic loss of MSH2 and MSH6. Germline mutation testing (father with multiple myeloma) showed a pathogenic germline MSH6 mutation [c.2731C>T (p.Arg911*)]. The patient started pembrolizumab which he tolerated well. After 14 months on treatment, he has demonstrated complete PSA response (0.1 ng/ml) and complete resolution of soft-tissue (visceral and lymph node) metastases and CBC recovery (3.5 x103/uL, Hb: 11.8 g/dl, PLT: 132 x103/uL).

**Conclusions**

This is the first report of very good and durable response to immune checkpoint inhibition in a mCRPC patient with biallelic MSH6 inactivation who had extensive disease and myelophthisis. Personalized therapy of advanced PC with pembrolizumab in the context of MMR deficiency can be effective in the bone marrow.

**Ethics Approval**

The patient consented to participate in the Precision Medicine protocol at Weill-Cornell Medicine (WCM). The study was approved by our Institutional Review Board and Ethics Committee (WCM / New York-Presbyterian IRB protocol #: 1305013903).

**Consent**

Consent was received.

#### P194 Fecal microbiota transplantation for refractory immune checkpoint inhibitor-associated colitis

##### Yinghong Wang, MD, PhD^1^, Hamzah Abu-Sbeih, MD^1^, Diana Wiesnoski^1^, Beth Helmink, MD PhD^1^, Vancheswaran Gopalakrishnan, MPH, PhD^1^, Kati Choi, MD^2^, Hebert DuPont, MD^3^, Zhi-Dong Jiang^3^, Michael T. Tetzlaff, MD PhD^1^, Jennifer A. Wargo, MD, MMSc^1^, Robert Jenq, MD^1^

###### ^1^Univ of Texas MD Anderson Cancer center, Houston, TX, USA; ^2^Baylor College of Medicine, Houston, TX, USA; ^3^Univ of Texas School of Public Health, Houston, TX, USA

####### **Correspondence:** Yinghong Wang (ywang59@mdanderson.org)

**Background**

Background: Immune checkpoint inhibitors (ICIs) can lead to a severe immune-mediated colitis (IMC), which is treated with immunosuppressive therapy that is associated with significant morbidity. Pre-clinical models demonstrated that patients who develop IMC have differential bacterial signatures in their gut microbiome and that targeting specific bacterial-taxa may abrogate such toxicities.[1-4] Herein, we report the first reported case series of two-patients with refractory IMC successfully treated with FMT.

**Methods**

Methods: Included two-patients had a refractory IMC that was treated with FMT (50 grams). Clinical courses and immunohistochemical analysis of the colonic mucosa are detailed in Figures 1-7. Stool microbiome prior to and post-FMT was assessed via 16S sequencing (Figures 8-9).

**Results**

Results: Patient-1: a 50-year-old female with metastatic urothelial carcinoma received combined CTLA-4 and PD-1 blockade. Two-weeks after treatment initiation, she developed CTCAE grade ≥2 IMC. Infectious workup was negative. Colonoscopy demonstrated severe ulceration. She received corticosteroids, infliximab and vedolizumab for 3 months, but her symptoms and ulcers persisted. She then received a single-FMT via colonoscopy with complete resolution of symptoms and healed ulcers within 2 weeks. Prior to FMT, analysis in the colonic mucosa demonstrated high-density of CD8+ and low-density of CD4+ FoxP3+ T-cells. Post-FMT, CD8+ T-cell decreased and CD4+ FoxP3+ increased. Patient-2: a 78-year-old male with prostate cancer received ipilimumab. Three-months after treatment initiation, he developed grade ≥2 IMC. Infectious etiologies were excluded. Colonoscopy confirmed IMC. His symptoms and mucosal ulcerations persisted despite corticosteroids, infliximab and vedolizumab for 5 months. Then he received the first-FMT with partial response, however, after the second-FMT he had complete resolution of symptoms and ulcers. The density of all T-cell subtypes decreased post-FMT with persistence of CD4+ FoxP3+ cells. Principal coordinates analyses demonstrated the similarity to the donor microbiome most noticeably immediately post-FMT, but later deviated away, still, distinct from pre-FMT. At time of IMC diagnosis, patient-1 had a predominance of Clostridia and absence of Bacteroidia and Verrucomicrobiae and patient-2 had a predominance of Gammaproteobacteria (predominantly Escherichia). Immediately post-FMT, donor FMT-derived bacteria colonized the intestines of patient-1 (~75%) with abundance of Akkermansia that later decreased with expansion of Bifidobacterium. In patient-2, there was a notable increase in Blautia, Bacteroides and Bifidobacterium species post-FMT and a decrease in Escherichia.

**Conclusions**

Conclusion: These cases provide provocative and novel evidence that refractory IMC could be treated successfully with FMT, which reconstitutes the gut microbiome with relative increase in the proportion of regulatory T cells within the colonic mucosa.

**References**

Dubin, K., et al., Intestinal microbiome analyses identify melanoma patients at risk for checkpoint-blockade-induced colitis. Nat Commun, 2016. 7: p. P341

*Corresponding author email: Ulrike.Gnad-Vogt@curevac.com.2. Vetizou, M., et al., Anticancer immunotherapy by CTLA-4 blockade relies on the gut microbiota. Science, 2015. 350(6264): p. 1079-84.3. Chaput, N., et al., Baseline gut microbiota predicts clinical response and colitis in metastatic melanoma patients treated with ipilimumab. Ann Oncol, 2017. 28(6): p. 1368-1379.4. Wang, F., et al., Bifidobacterium can mitigate intestinal immunopathology in the context of CTLA-4 blockade. Proc Natl Acad Sci U S A, 2018. 115(1): p. 157-161.5. Borody, T.J. and A. Khoruts, Fecal microbiota transplantation and emerging applications. Nat Rev Gastroenterol Hepatol, 2011. 9(2): p. 88-96.

**Ethics Approval**

This study was approved by the Institutional Review Board at The University of Texas MD Anderson Cancer Center (PA18-0372)

**Consent**

Enrolled patients signed consent for the treatment protocol (CIND17-0036, CIND17-0058).

**Fig. 1 (abstract P194).**
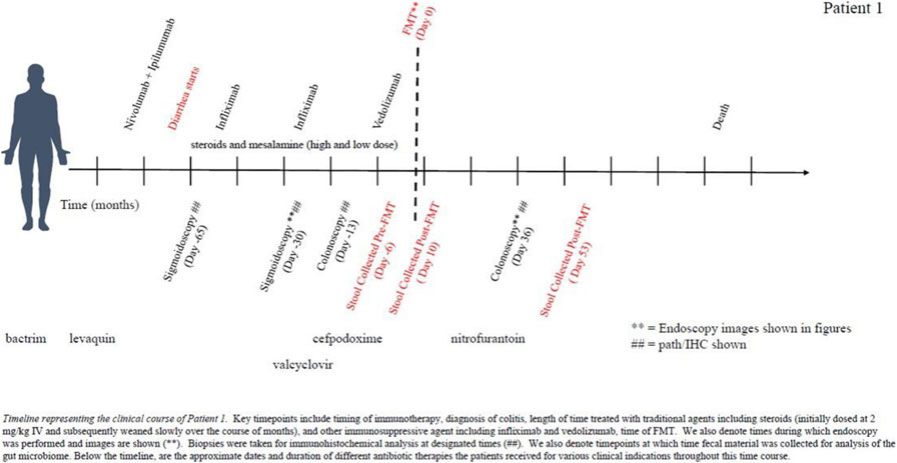
See text for description.

**Fig. 2 (abstract P194).**
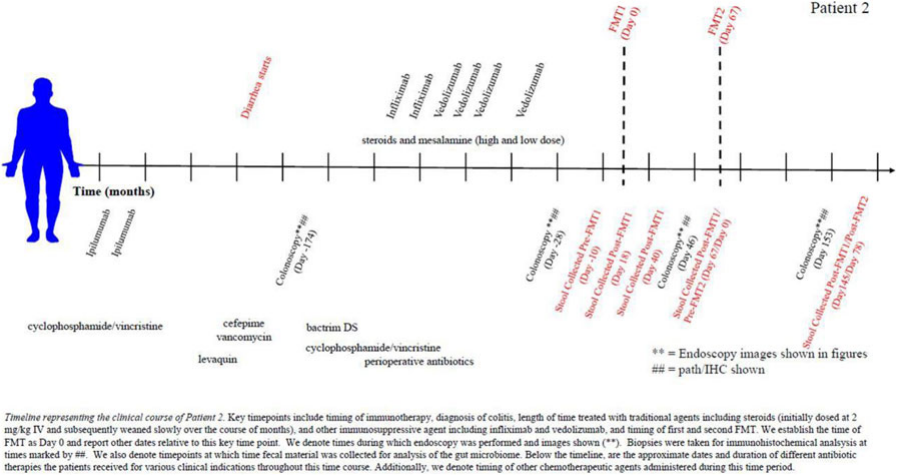
See text for description.

**Fig. 3 (abstract P194).**
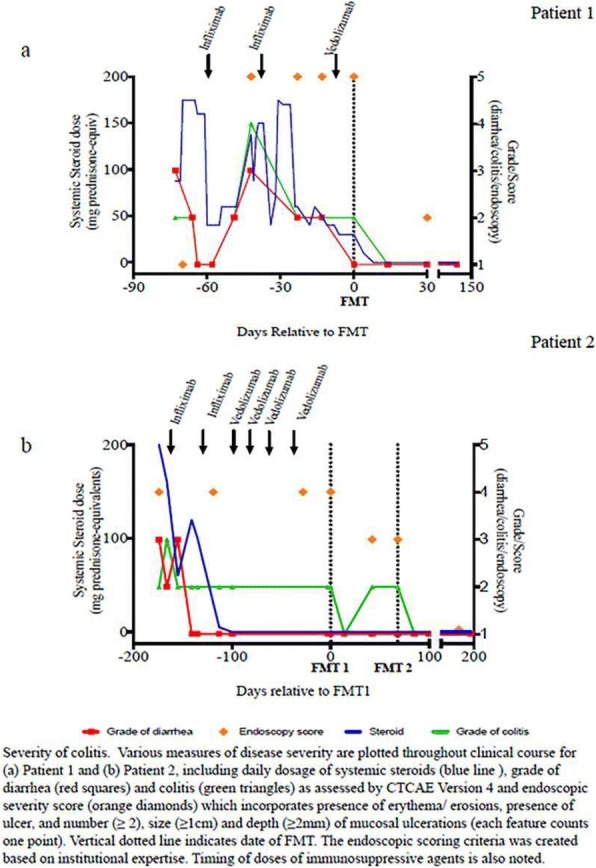
See text for description.

**Fig. 4 (abstract P194).**
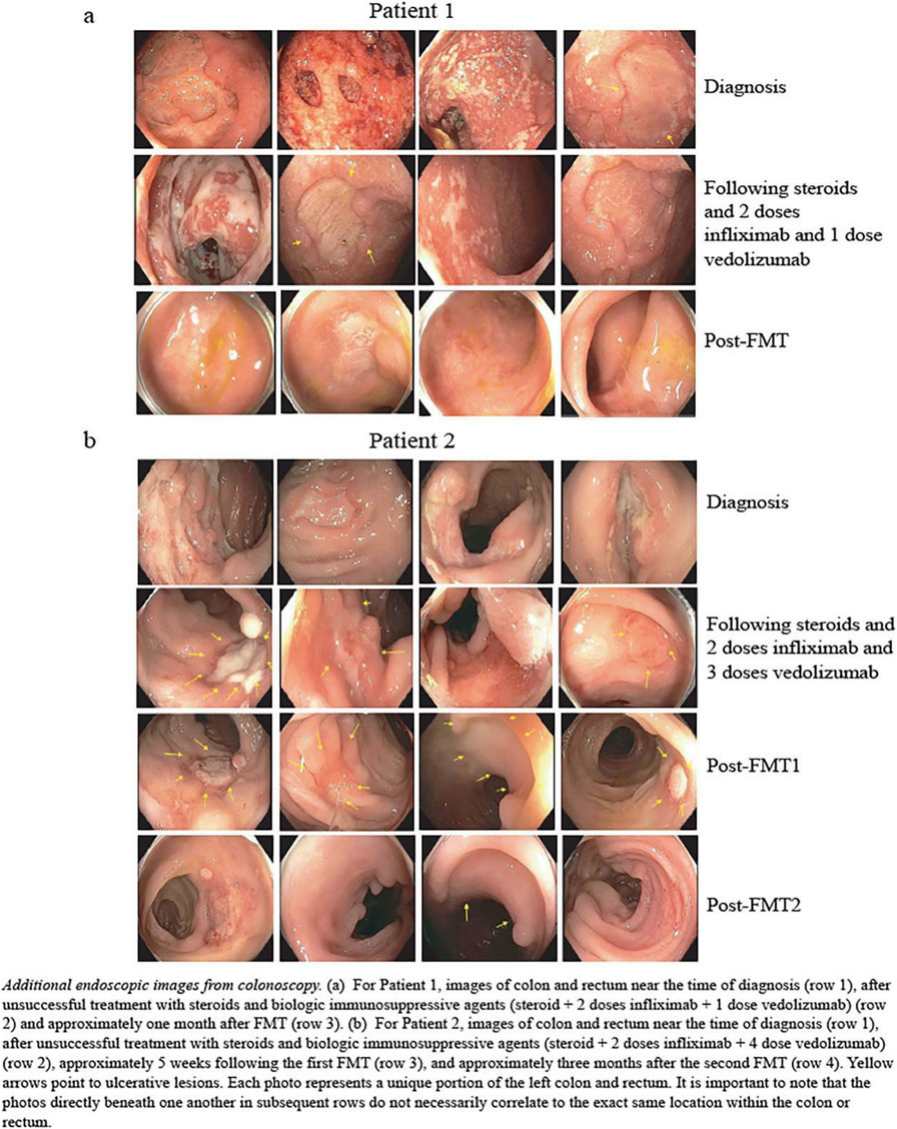
See text for description.

**Fig. 5 (abstract P194).**
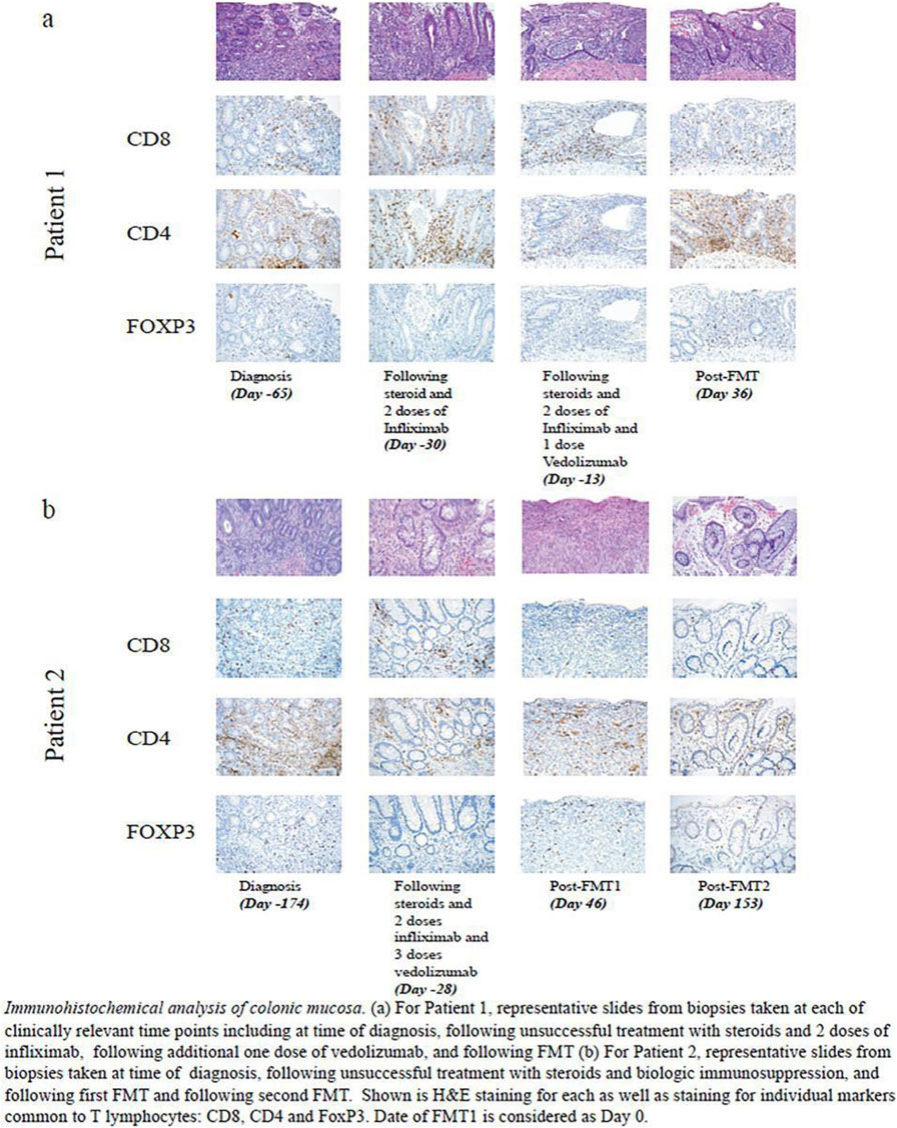
See text for description.

**Fig. 6 (abstract P194).**
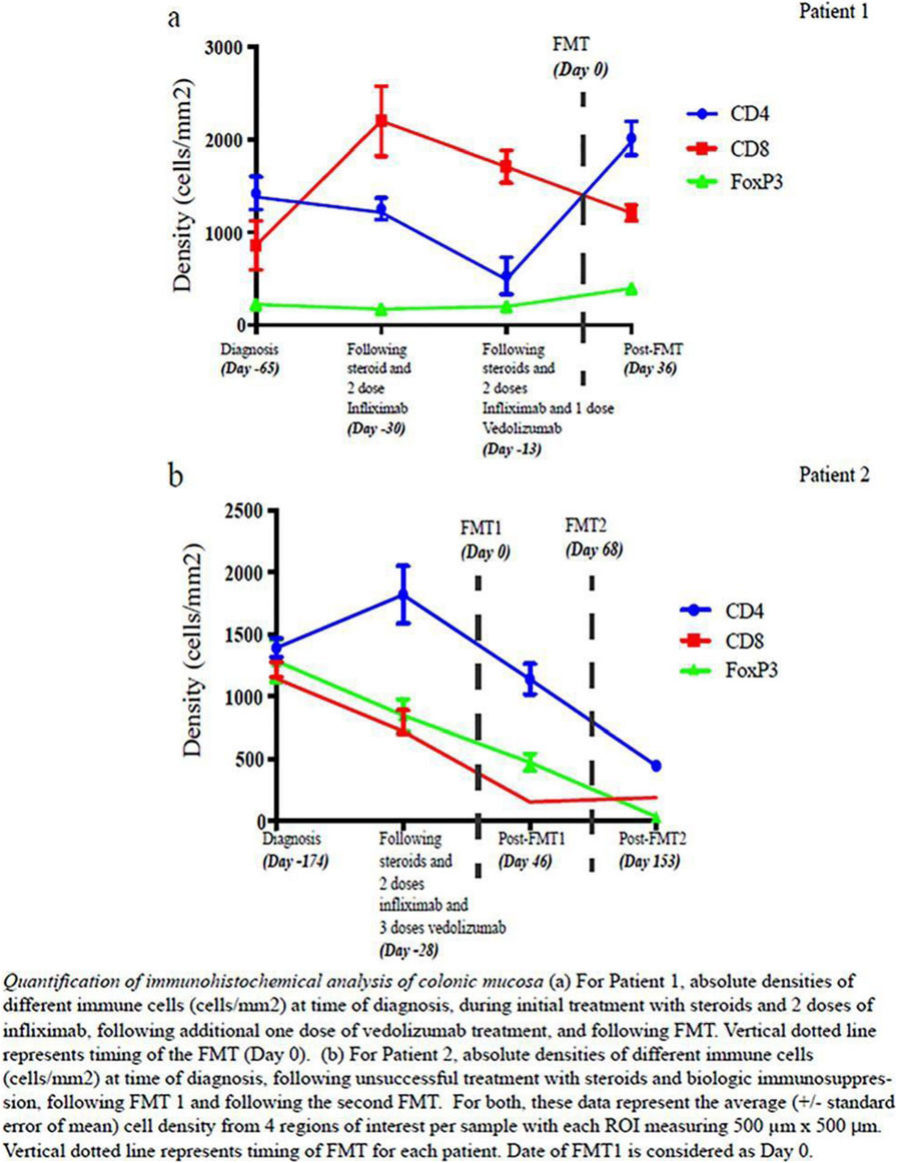
See text for description.

**Fig. 7 (abstract P194).**
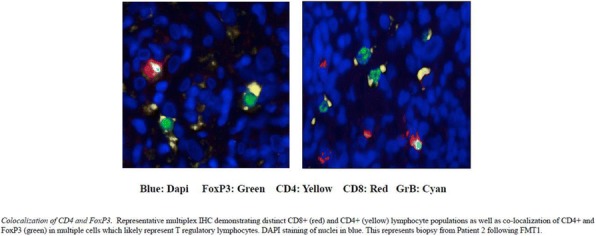
See text for description.

**Fig. 8 (abstract P194).**
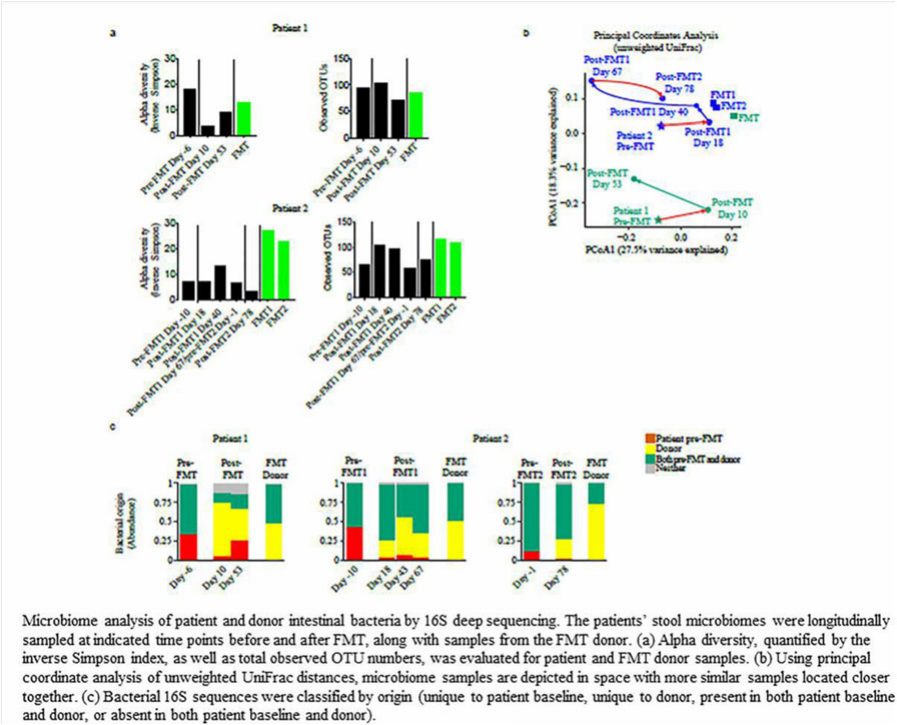
See text for description.

**Fig. 9 (abstract P194).**
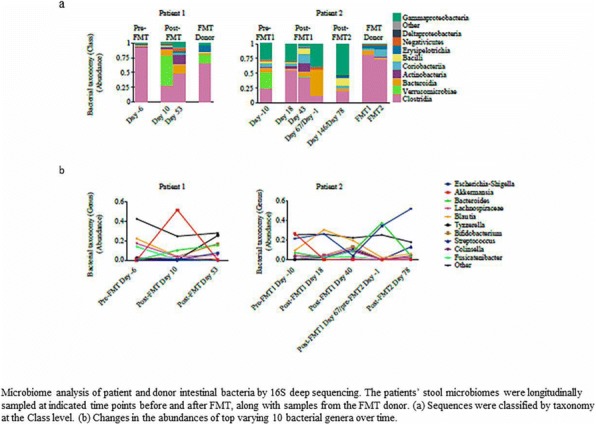
See text for description.

### Cellular Metabolism and Antitumor Immunity

#### P195 Transcriptomic profiling of specific populations of T-cells in Acute Myeloid Leukemia

##### Milad Abolhalaj, MSc^1^, Milad Abolhalaj, MSc^1^, Milad Abolhalaj, MSc^1^, Henrik Lilljebjörn^1^, Carl Sandén^1^, Karin Hägerbrand^2^, Peter Ellmark, PhD^2^, Carl Borrebaeck^1^, Thoas Fioretos^1^

###### ^1^Lund University, Lund, Sweden; ^2^Alligator Bioscience AB, Lund, Sweden

####### **Correspondence:** Milad Abolhalaj (kristina.lundberg@immun.lth.se)

**Background**

Acute myeloid leukemia (AML) has a 5-year survival of <5% among patients over 65 years. Checkpoint inhibitors have shown extraordinary effects in certain cancer patient groups and have been approved for different cancers.

However, the results have been modest in AML and alternative strategies are thus warranted. Our study focuses on the transcriptome of specific T cell populations in AML patients to pinpoint novel targets for T cell-based interventions.

**Methods**

CD8+ T-cells (CTL), CD4+ T cells (Th), and regulatory T-cell (Tregs) populations were sorted from peripheral blood of AML patients (n=5), harbouring TP53-mutations, and controls (n=3) using flow cytometry. RNA was extracted and sequenced and data was analysed using Qlucore Omics Explorer software. Differentially expressed genes in each T-cell population from AML patients, as compared to its counterpart in controls, were identified (fold change>2, one-tailed t-test, FDR<0.1). Additionally, using ANOVA (FDR<0.1), transcriptomes of the three T-cell populations were compared in AML and then similarly in controls. Furthermore, genes expressed at a higher level in CTLs from AML as compared to Tregs in AML were identified (fold change>2, one-tailed t-tests, FDR<0.1) and those associated with plasma membrane were extracted. Additionally, plasma membrane-associated genes expressed higher in Th as compared to Tregs, and higher in Tregs as compared to both Th cells and CTL, were identified.

**Results**

ANOVA of the T-cell populations in AML identified 161 differentially expressed genes and the same analysis on control subjects resulted in 338 (38 overlapping). Furthermore, 577/498 genes were found to be expressed at higher/lower level by CTLs from AML compared to CTLs in control subjects. The corresponding numbers for Th cells and Tregs were 181/141 and 390/387, respectively. Interestingly, 10 plasma membrane-associated genes were expressed at higher levels by CTLs in AML patients compared to Tregs in AML. Furthermore, 5 membrane- associated genes were expressed higher in AML Th cells than AML Tregs. Finally, 1 membrane-associated gene was expressed higher by AML Tregs compared to both AML Th and CTLs, clearly supporting our strategy to identify novel targets.

**Conclusions**

The gene expression profiles of T-cells from AML differ from T-cells of control subjects and the differentially expressed genes give mechanistic insights into the impact of AML. Additionally, the plasma membrane-associated genes identified as selectively expressed by different T-cell populations in AML are potential targeting candidates for specific therapeutic interventions.

**Ethics Approval**

Study was performed in accordance with guidelines/regulations, approved by the Regional Ethics Committee.

**Consent**

Informed consent obtained from all donors.

#### P196 Comparison of culture media for in citro T cell assays and expansion

##### Ponni Anandakumar, Anne Lodge, PhD, Anne Lodge, PhD, Benjamin Tjoa, Ph D

###### Astarte Biologics, Inc, Bothell, WA, USA

####### **Correspondence:** Ponni Anandakumar (panand@astartebio.com); Anne Lodge

**Background**

Identification of a reliable culture media to support in vitro T cell studies has become an important link in the chain of various Immuno-Oncology strategies. While many labs have chosen one favorite media for their T cell culture needs, it may be prudent to identify alternatives that can perform suitably, whether one works in the development of cell-based assays to screen potential drug candidates or generates and expands antigen-specific T cells. To address this issue, we have conducted a series of studies comparing the performance of several culture media.

**Methods**

A list of culture media (including several classic media + supplements as well as several new media) was compared to several commercially available T cell media in the generation of primary MLR (mixed-lymphocyte reaction), antigen-recall assay (e.g., CMV, tetanus), antigen-specific T cell proliferation assay, as well as in anti-CD3/CD28 driven T cell expansion culture.

**Results**

Classical media supplemented with several defined components can support primary in vitro responses as measured by cytokine production. Sustained T cell proliferation demanded additional supplementation and revealed greater differences between media. One representative data from these studies is included in this abstract. This experiment demonstrates the effect of human AB serum (HS) or fetal bovine serum (FBS) added to the culture medium X- VIVO 15 (Lonza, Walkersville, MD). At low peptide concentrations (3 and 10 ng/mL), the presence of HS and FBS inhibits T cell proliferation compared to X-VIVO 15 alone.

**Conclusions**

Media selection affects both T cell proliferation and function and therefore is critical to the success of adoptive T cell therapies. The strengths and shortcomings of several media are revealed in these data.

**Fig. 1 (abstract P196).**
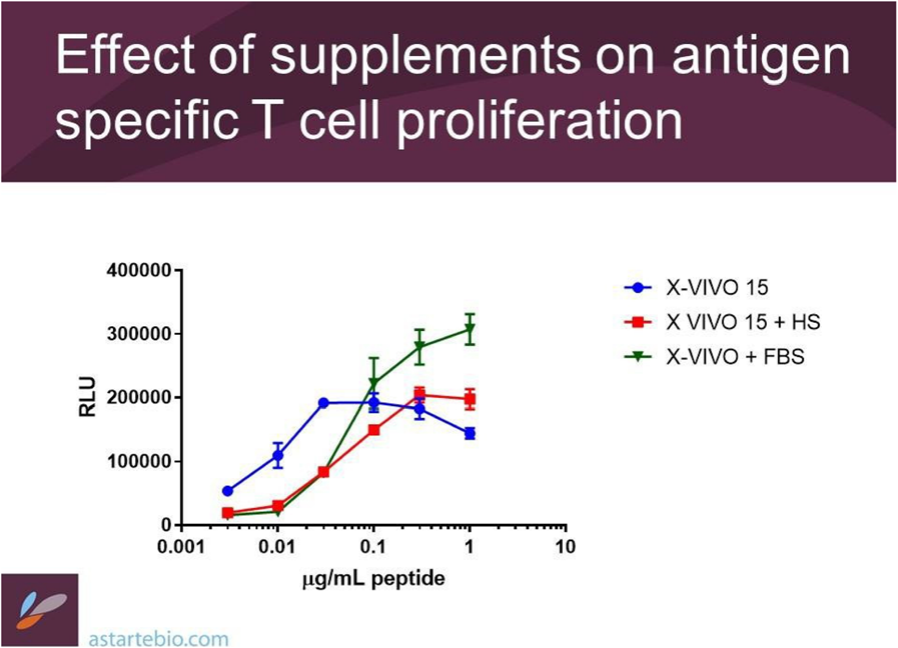
Effect of supplements on antigen specific T cells

#### P197 Modulating hexokinase 2 as a novel approach for simultaneous targeting of the tumor and its immunosuppressive microenvironment

##### Vered Behar, PhD, Reut Yosef, PhD, Eyal Dor-On, PhD, Nofar Amsalem, MSc, Yuval Ariel, MSc, Oren Becker, PhD

###### Vidac Pharma, Jerusalem, Israel

####### **Correspondence:** Vered Behar (vbehar@vidacpharma.com)

**Background**

During malignant cell transformation and thereafter, many cancer types undergo metabolic reprograming to aerobic glycolysis to support their growth and proliferation, a phenomenon known as the Warburg effect. Elevated aerobic glycolysis results in excess lactate secretion to the tumor microenvironment, contributing to its immunosuppressive qualities. Hexokinase 2 (HK2), which catalyzes the first step of glucose metabolism and is key to aerobic glycolysis, is also a key gateway for apoptosis. HK2 becomes overexpressed in many cancer types, either in conjunction or instead of the normal HK1 isozyme, where it binds to the outer mitochondrial membrane via the VDAC1 channel. This VDAC1/HK2 association entails multiple advantages to cancer cells: a) blocks pro-apoptotic signals; b) fuels glycolysis with a continuous flux of mitochondrial ATP; and c) reduces sensitivity to feedback inhibition by HK2 product, glucose-6-phosphate. Temporal high HK2 expression, and binding to VDAC, is also found in a variety of activated immune cells to support their changing metabolic needs. Detachment of HK2 from VDAC1 in activated immune cells leads to various responses ranging from glycolysis inhibition, NLRP3-mediated inflammasome activation, and metabolic reprogramming to activate immunity.

**Methods**

VDA-1102 is a novel small molecule that detaches HK2 from the mitochondria (it is not an inhibitor). We present here in vitro and in vivo studies demonstrating its mechanism of action, both in cancer cells and in immune cells, resulting in immune-mediated anti-tumor response.

**Results**

In vitro studies demonstrated that VDA-1102 selectively detaches HK2, but not HK1, from VDAC1 leading to cancer cell apoptosis as well as glycolysis inhibition and reduction in lactate secretion, culminating in proliferation inhibition. Effects on macrophages (activating the M1 phenotype) and T cells were also demonstrated. In vivo efficacy studies in various syngeneic mouse models demonstrated significant tumor growth delay, prolongation of survival, and metastasis prevention. Analysis of tumor-associated immune cells in vivo indicated a favorable treatment-induced change in these cells, especially in macrophages and T cells.

**Conclusions**

This data supports the notion that VDA-1102’s multiple effects target both cancer and the immune system. In cancer cells it induces apoptosis and prevents secretion of lactate to the tumor microenvironment, whereas in macrophages it stimulates an anti-tumor immune response. Our findings support further development of VDA-1102 to evaluate its potential as an anti-cancer therapy, either as a monotherapy or in combination with immunotherapies in high HK2- expressing solid tumors.

**Ethics Approval**

All mouse studies were performed in compliance with “The Israel Animal Welfare Act” and following “The Israel Board for Animal Experiments” approval No IL-17-7-244.

#### P198 IDH1-R132H, a rate determining mutation in the timeline of anaplastic astrocytoma

##### Jonathan Fraser, MD, Mikhail Prosniac, PhD, Larry Harshyne, David Andrews, MD, D. Craig Hooper, PhD

###### Thomas Jefferson University, Philadelphia, PA, USA

####### **Correspondence:** D. Craig Hooper (douglasc.hooper@jefferson.edu)

**Background**

Until recently, the WHO classified the differences between grade III anaplastic astrocytomas (AA) and grade IV astrocytomas, or glioblastomas (GBM) by histological findings including neovascularization and necrosis. However, it is now recognized that isocitrate dehydrogenase (IDH1/2) mutation status differentiates 2 subsets of AA. Standard of care survival for AA patients with tumors expressing IDH1 with the R132H mutation (IDH1R132H) is commonly 5-7 years. In comparison, AA tumors with wild-type IDH1 (IDH1wt), like the majority of GBM, shares the poor prognosis of GBM, with median survival of 15-17 months. These tumors differ in their content of CD163+ tumor associated macrophage (TAM) that resemble M2 monocytes. The objective of the current is to determine if there is an association between the expression of IDH1 isoforms and TAM infiltration, and whether or not this is reflected in features of peripheral immunity.

**Methods**

Patients were diagnosed at surgery with AA by WHO histologic criteria. Pre-operative MRIs were assessed for extent of enhancement. Peripheral blood obtained prior to surgery was examined for immune cell distribution by flow cytometry, cytokine expression by RT-PCR, and local cytokine levels by Luminex. Tumor tissue obtained at surgery was assessed for IDH1-R132H expression and CD163+ TAM infiltration by immunohistochemistry.

**Results**

The majority of IDH1wt tumors enhanced in MRI while most IDH1R132H did not. However several tumors from both IDH1 subsets exhibited the opposite MRI characteristics. CD163+ TAM were abundant in tumors that enhanced but rare in those that did not. Circulating CD163+ monocytes were also elevated in patients with enhancing tumors. Differences in the expression by PBMC of a variety of genes encoding M2 phenotype markers as well as immune factors and their receptors were also detected; these also discriminate between subjects with enhancing versus non-enhancing tumors. MRI enhancement was more closely associated than IDH1wt with evidence of type 2 immune properties in PBMC and sera.

**Conclusions**

The data indicates that there is a closer association between the loss of tumor vascular integrity than IDH1 mutational status in the appearance of CD163+ TAM. Differences in the tumor microenvironment evidenced by TAM content are correlated with changes in measures of peripheral immune function suggestive of a type 2 immune bias. IDH1wt and IDH1R132H AA evidently progress through similar stages, albeit at different rates.

**Acknowledgements**

Funded by the Albert Stevens Foundation

**Ethics Approval**

the study was performed with de-identified specimens obtained through Thomas Jefferson University under IRB approval number:16D.424

#### P199 Myo-inositol up-regulates PD-L1 in non-small cell lung cancer

##### Eileen Fung, PhD, Jane Yanagawa, Jessica Kim

###### UCLA, Los Angeles, CA, USA

####### **Correspondence:** Jane Yanagawa (jyanagawa@mednet.ucla.edu)

**Background**

Myo-inositol, an isomer of glucose, has shown to have preclinical promise for chemoprevention in multiple cancer models including lung cancer. However, results are conflicting in small randomized trials whether there is clinical benefit. While the molecular mechanisms driving chemoprevention are unclear, myo-inositol has been reported to affect the Akt1 signaling transduction pathway. Activation of Akt1 signaling pathway is associated with progression of non-small cell lung cancer (NSCLC) and enhanced expression of programmed death ligand-1 (PD-L1), an immunoregulatory protein. Binding of PD-L1 with its receptor, programmed death 1 (PD-1), results in T-cell exhaustion and, ultimately, immune evasion. We hypothesize that myo-inositol may impact signaling pathways that regulate PD-L1 expression.

**Methods**

A549, H441, H460, H1299, H1437, and H2291 lung cancer cell lines were treated with increasing doses of myo-inositol (0, 0.1, 1, 10, 100, and 200mM) for 72 hours in culture. PD-L1 cell surface expression was quantified using flow cytometry. Akt1 activation was determined by western blot analysis.

**Results**

Heterogeneous levels of basal PD-L1 expression were exhibited in A549, H441, H460, H1299, H1437, and H2291.

Exposure of 200mM myo-inositol for 72 hours, cell surface PD-L1 expression were increased (1.1~2.25 fold, flow cytometry) in all 6 NSCLC cell lines. Western blot analysis showed increased phosphorylation of Akt1 (1.1~2.2 fold, western blot) in all NSCLC cell lines following myo-inositol treatment.

**Conclusions**

Upregulation of PD-L1 expression is exhibited following treatment with myo-inositol in NSCLC cell lines. This is the first report linking myo-inositol to regulation of PD-L1 expression through the AKT pathway.

#### P200 Cell competition and flower code regulates competitive interactions between tumor and its microenvironment

##### Rajan Gogna, PhD, MS, MBA^1^, Esha Madan, PhD, MS, MBA^1^, Masaki Nagane^2^, Christopher Pelham^3^, Taylor Parker^4^, Antonio Beltran^5^, Carlos Carvalho, MD^5^

###### ^1^Champalimaud Center For Unknown, Lisbon, Portugal; ^2^Azabu University, Tokyo, Japan; ^3^St. Louis College of Pharmacy, Lisbon, Portugal; ^4^Simon Cancer Center, IU, Libon, Portugal; ^5^Champalimaud Centre for the Unknown, Lisbon, Portugal

####### **Correspondence:** Rajan Gogna (rajan.gogna@research.fchampalimaud.org)

**Background**

In living tissues, cells continuously interact, compete and compare their relative fitness levels[1, 2]. Cell competition explains oncogenic growth as an overarching phenomenon, it is the process where unfit cells, when confronted with fit cells, are recognized and progressively eliminated[3-5]. Cancerous cells cheat this mechanisms and project themselves as super-fit to neighboring cells[1, 6-10]. These cells instead of being eliminated, induce apoptosis in surrounding normal cells. This promotes oncogenic growth by creating space and availability of nutrients (Fig-1a). Recently a novel molecular mechanism termed “Flower-code” which helps in recognition of unfit cells via cell surface marks called “Fitness fingerprints” was elucidated[8, 12]. In humans these fingerprints are encoded by 4 different isoforms of the transmembrane protein Flower (C9ORF7). The isoforms that indicate reduced fitness are called Lose and are expressed in viable cells marked to be eliminated, by fitter cells which express Win isoforms.

**Methods**

We have used techniques including CRISPR-assisted genetic engineering, a molecular cell competition assay in human cells (Fig-2a), live-cell imaging, gene expression analysis via FISH, qPCR in human cancer cells and FFPE patient cancer samples of multiple origins and development of humanized anti-Flower antibodies, via phage display.

**Results**

Novel results demonstrate the functional properties of Flower. Isoforms 2 and 4, with N-terminus internalized, are termed “Win” isoforms as they provide competitive advantage over cells expressing Flower “Lose” isoforms 1 and 3, with N-terminus externalized (Fig-1b). Importantly, Lose-expressing cells do not undergo apoptosis if neighboring cells have similar levels of Lose, therefore acting as canonical fitness comparison markers. Flower-/- MCF-7 cells ectopically expressing Lose isoforms, are eliminated by Flower-/- cells expressing Win isoforms (Fig- 2b). FISH and qPCR show very poor expression of flower in normal tissue, but significantly high expression of Win isoforms within breast, colon, lung, SCC, liver and prostate cancers (n=95). Interestingly high expression of Lose isoforms in observed in the stromal cells 400-600μm from the edge of the tumor (Fig-2c).

**Conclusions**

First-time evidence is presented that Win and Lose isoforms of Flower, a fitness fingerprint protein, are expressed in cancer and stromal tissue, respectively. Win isoforms provides competitive advantages to cancer cells and expression of Lose isoforms on stromal cells marks them for progressive elimination, thereby supporting oncogenic growth. Flower is poorly expressed in normal tissues, thus is a high potential target for immune-therapy. We are presenting data highlighting the potential of monoclonal anti-Flower antibody in treatment of triple negative breast cancer PDX in abstract-P237

**Acknowledgements**

We acknowledge Champalimaud Research Foundation for supporting and funding this research.

**References**

1. Levayer R, Moreno E. Mechanisms of cell competition: themes and variations. J Cell Biol. 2013;200(6):689-98. Epub 2013/03/20. doi: 10.1083/jcb.201301051. PubMed PMID: 23509066; PubMed Central PMCID: PMCPMC3601360.

2. Amoyel M, Bach EA. Cell competition: how to eliminate your neighbours. Development. 2014;141(5):988-1000. Epub 2014/02/20. doi: 10.1242/dev.079129. PubMed PMID: 24550108; PubMed Central PMCID: PMCPMC3929405.

3. Merino MM, Rhiner C, Lopez-Gay JM, Buechel D, Hauert B, Moreno E. Elimination of unfit cells maintains tissue health and prolongs lifespan. Cell. 2015;160(3):461-76. Epub 2015/01/21. doi: 10.1016/j.cell.2014.12.017. PubMed PMID: 25601460; PubMed Central PMCID: PMCPMC4313366.

4. Morata G, Ripoll P. Minutes: mutants of drosophila autonomously affecting cell division rate. Dev Biol. 1975;42(2):211-21. Epub 1975/02/01. PubMed PMID: 1116643.

5. Moreno E, Basler K, Morata G. Cells compete for decapentaplegic survival factor to prevent apoptosis in Drosophila wing development. Nature. 2002;416(6882):755-9. Epub 2002/04/19. doi: 10.1038/416755a. PubMed PMID: 11961558.

6. Di Gregorio A, Bowling S, Rodriguez TA. Cell Competition and Its Role in the Regulation of Cell Fitness from Development to Cancer. Dev Cell. 2016;38(6):621-34. Epub 2016/09/28. doi: 10.1016/j.devcel.2016.08.012. PubMed PMID: 27676435.

7. Moreno E. Is cell competition relevant to cancer? Nat Rev Cancer. 2008;8(2):141-7. Epub 2008/01/11. doi: 10.1038/nrc2252. PubMed PMID: 18185517.

8. Rhiner C, Lopez-Gay JM, Soldini D, Casas-Tinto S, Martin FA, Lombardia L, et al. Flower forms an extracellular code that reveals the fitness of a cell to its neighbors in Drosophila. Dev Cell. 2010;18(6):985-98. Epub 2010/07/16. doi: 10.1016/j.devcel.2010.05.010. PubMed PMID: 20627080.

9. Levayer R, Hauert B, Moreno E. Cell mixing induced by myc is required for competitive tissue invasion and destruction. Nature. 2015;524(7566):476-80. Epub 2015/08/20. doi: 10.1038/nature14684. PubMed PMID: 26287461.

10. Moreno E, Rhiner C. Darwin's multicellularity: from neurotrophic theories and cell competition to fitness fingerprints. Curr Opin Cell Biol. 2014;31:16-22. Epub 2014/07/16. doi: 10.1016/j.ceb.2014.06.011. PubMed PMID: 25022356; PubMed Central PMCID: PMCPMC4238900.

11. Merino MM, Rhiner C, Portela M, Moreno E. “Fitness fingerprints” mediate physiological culling of unwanted neurons in Drosophila. Curr Biol. 2013;23(14):1300-9. Epub 2013/07/03. doi: 10.1016/j.cub.2013.05.053. PubMed PMID: 23810538.

12. Gogna R, Shee K, Moreno E. Cell Competition During Growth and Regeneration. Annu Rev Genet. 2015;49:697-718. Epub 2015/12/04. doi: 10.1146/annurev-genet-112414-055214. PubMed PMID: 26631518.

**Ethics Approval**

All animal studies performed in this research are approved by Champalimaud Institutional Review Board and the Portuguese Gov Ethical board (DGAV), the approval number is 0421/000/000.

**Fig. 1 (abstract P200).**
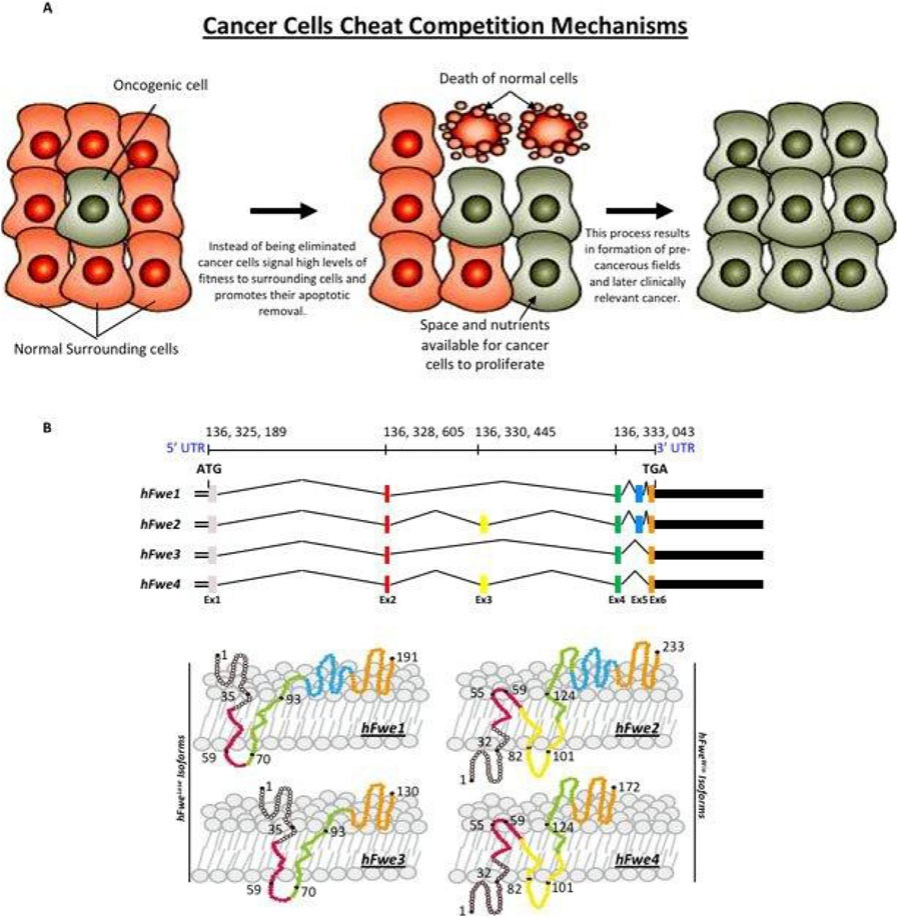
See text for description.

**Fig. 2 (abstract P200).**
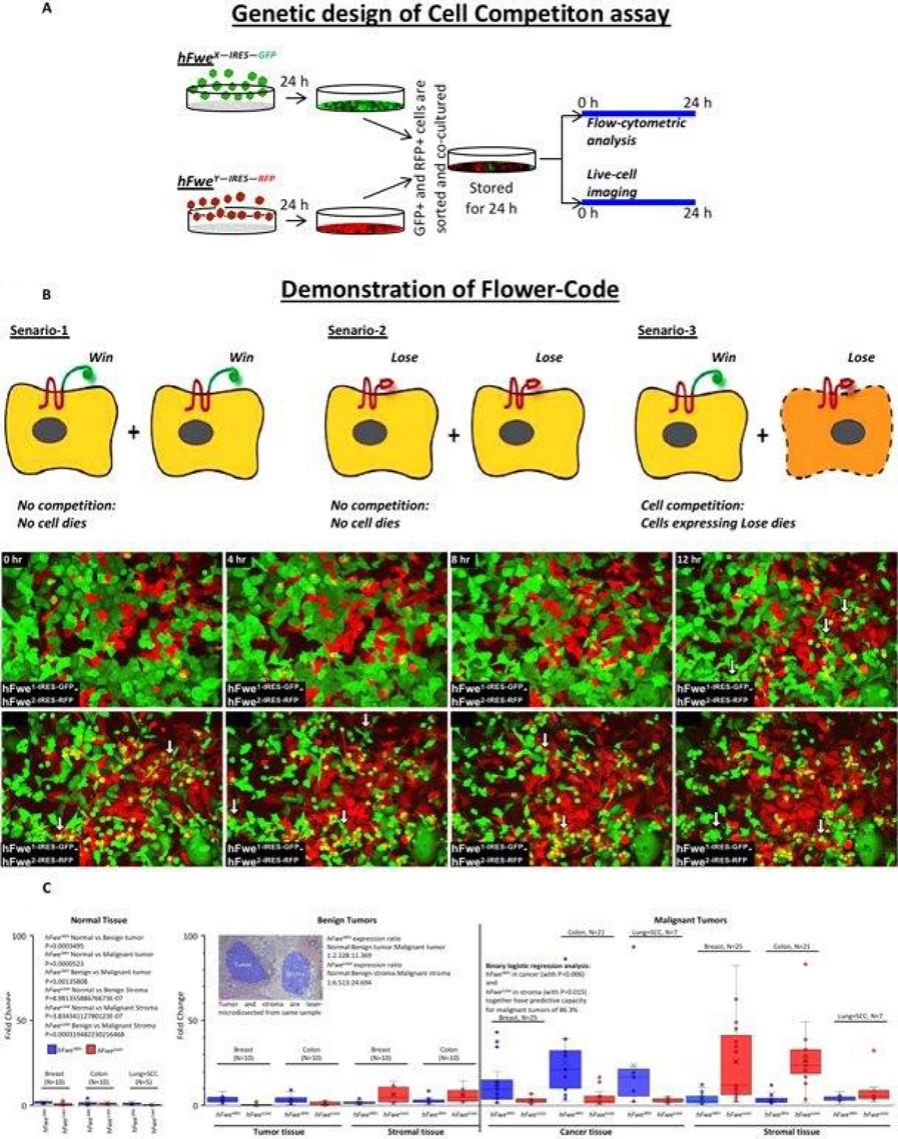
See text for description.

#### P201 Cell competition genes drive donor cell leukemia in AML patients

##### Rajan Gogna, PhD, MS, MBA^1^, Esha Madan, PhD, MS, MBA^1^, Christopher Pelham^2^, Taylor Parker^3^

###### ^1^Champalimaud Center For Unknown, Lisbon, Portugal; ^2^St. Louis College of Pharmacy, Lisbon, Portugal; ^3^IUPUI, Simon Cancer Center, Libon, Portugal

####### **Correspondence:** Rajan Gogna (rajan.gogna@research.fchampalimaud.org)

**Background**

Allogeneic hematopoietic stem cell transplantation (alloHSCT) improves overall survival in AML patients[1-4].

However, secondary leukemia is a major clinical problem, and surprisingly this can arise from healthy allogenic donor cells, termed donor cell leukemia (DCL)[5-8]. DCL is a rare complication (5%) associated with alloHSCT, where normal donor cells become transformed into aggressive leukemia or myelodysplastic syndrome in the host environment[9]. The mechanisms behind DCL remain unknown, however, some dormant donor cell mutations have been proposed to play some role. DNA sequencing has revealed that de-novo AML patients carry DNMT3A mutations (22%), which are associated with intermediate-risk cytogenetic profile and poor outcome[10]. Interestingly, the role of DNMT3A mutations was also highlighted in DCL. For example, an AML patient that received peripheral blood stem cells (PBSCs) from his HLA-matched brother developed DCL 27 months post- transplant. Whole-exome sequencing from specimens of the initial AML, first complete remission after chemotherapy, the first relapse, donor PBSCs, DCL at 27 and DCL at 36 months revealed that IDH2(R140Q) and DNMT3A(V150Gfs) mutations were present in the healthy donor cells at a low frequency and were potentially responsible for DCL[11]. Based on literature sets available and our preliminary data, we hypothesize that cell competition between host and donor cells contributes to DCL development, as its mechanisms allow the donor cells to acquire a Win phenotype and colonize the weak cellular microenvironment of the allogenic host. Cell competition results in the elimination of the less fit cells by the fitter cells through direct or indirect mechanisms[12-16]. Please refer to abstracts: P200

**Methods**

Flower-knockout MCF-7 cells were created using CRISPR and Win/Lose cell types were generated with help of lentiviral particles. These Win and Lose cells were allowed to compete (Fig-1a) and both populations were captured using Fluidigm single-cell capture technique (Fig-1b). Following which, both cell types were processed for single- cell RNA seq.

**Results**

RNA-seq results show that during competition the Winner cells have significantly reduced DNMT3A expression and activity. RNA-seq has revealed a novel set of uncharacterized genes that participate in this process.

**Conclusions**

Our results demonstrate a parallel behavior in DNMT3A status between alloHSCT donor cells which result in DCL and Winner cells from cell competition assay. We have identified a list of novel uncharacterized genes that may serve as biomarkers to select the appropriate alloHSCT donor cells that can help minimize the risk of DCL development.

**Acknowledgements**

We acknowledge Champalimaud Research Foundation for supporting and funding this research.

**References**

1. Li D, Wang L, Zhu H, Dou L, Liu D, Fu L, Ma C, Ma X, Yao Y, Zhou L, Wang Q, Wang L, Zhao Y, Jing Y, Wang L, Li Y, Yu L. Efficacy of Allogeneic Hematopoietic Stem Cell Transplantation in Intermediate-Risk AcuteMyeloid Leukemia Adult Patients in First Complete Remission: A Meta-Analysis of Prospective Studies. PLoS One. 2015 Jul 21;10(7):e0132620. doi: 10.1371/journal.pone.0132620. eCollection 2015. PubMed PMID: 26197471

2. Schiller GJ, Tuttle P, Desai P. Allogeneic Hematopoietic Stem Cell Transplantation in FLT3-ITD-Positive Acute MyelogenousLeukemia: The Role for FLT3 Tyrosine Kinase Inhibitors Post-Transplantation. Biol Blood Marrow Transplant. 2016 Jun;22(6):982-990. doi: 10.1016/j.bbmt.2016.01.013. Epub 2016 Jan 16. PubMed PMID: 26785334

3. Xu Y, Sun Y, Shen H, Ding L, Yang Z, Qiu H, Sun A, Chen S, Wu D. Allogeneic hematopoietic stem cell transplantation could improve survival of cytogenetically normal adult acute myeloid leukemia patients with DNMT3A mutations. Am J Hematol. 2015 Nov;90(11):992-7. doi: 10.1002/ajh.24135. PubMed PMID: 26223865.

4. Wang Y, Chen G, Cao R, Li J, He L, Guo X, Liang J, Shi P, Zhou Y, Xu B. Allogeneic hematopoietic stem cell transplantation improves the prognosis of p16- deleted adultpatients with acute lymphoblastic leukemia. Pharmacogenomics. 2017 Jan;18(1):77-84. doi: 10.2217/pgs-2016-0075. Epub 2016 Dec 14. PubMed PMID: 27967319.

5. Flynn CM, Kaufman DS. Donor cell leukemia: insight into cancer stem cells and the stem cell niche. Blood. 2007 Apr 1;109(7):2688-92. PubMed PMID: 17132724.

6. Gondek LP, Zheng G, Ghiaur G, DeZern AE, Matsui W, Yegnasubramanian S, Lin MT, Levis M, Eshleman JR, Varadhan R, Tucker N, Jones R, Gocke CD. Donor cell leukemia arising from clonal hematopoiesis after bone marrow transplantation. Leukemia. 2016 Sep;30(9):1916-1920. doi: 10.1038/leu.2016.63. Epub 2016 Mar 15. PubMed PMID: 26975880.

7. Ma H, Liu T. Development of donor cell leukemia following peripheral blood stem cell transplantation for severe aplastic anemia: A case report. Oncol Lett. 2016 Jun;11(6):3858-3862. Epub 2016 Apr 18. PubMed PMID: 27313707.

8. Wiseman DH. Donor cell leukemia: a review. Biol Blood Marrow Transplant. 2011 Jun;17(6):771-89. doi: 10.1016/j.bbmt.2010.10.010. Epub 2010 Oct 15. PubMed PMID: 20951819.

9. Shah NN, Bacher U, Fry T, Calvo KR, Stetler-Stevenson M, Arthur DC, Kurlander R, Baird K, Wise B, Giralt S, Bishop M, Hardy NM, Wayne AS. Myelodysplastic syndrome after allogeneic hematopoietic stem cell transplantation: diagnosticand therapeutic challenges. Am J Hematol. 2012 Sep;87(9):916-22. doi: 10.1002/ajh.23174. Epub 2012 Apr 4. PubMed PMID: 22473867.

10. Ley TJ, Ding L, Walter MJ, McLellan MD, Lamprecht T, Larson DE, Kandoth C, Payton JE, Baty J, Welch J, Harris CC, Lichti CF, Townsend RR, Fulton RS, Dooling DJ, Koboldt DC, Schmidt H, Zhang Q, Osborne JR, Lin L, O'Laughlin M, McMichael JF, Delehaunty KD, McGrath SD, Fulton LA, Magrini VJ, Vickery TL, Hundal J, Cook LL, Conyers JJ, Swift GW, Reed JP, Alldredge PA, Wylie T, Walker J, Kalicki J, Watson MA, Heath S, Shannon WD, Varghese N, Nagarajan R, Westervelt P, Tomasson MH, Link DC, Graubert TA, DiPersio JF, Mardis ER, Wilson RK. DNMT3A mutations in acute myeloid leukemia. N Engl J Med. 2010 Dec 16;363(25):2424-33. doi: 10.1056/NEJMoa1005143. Epub 2010 Nov 10. PubMed PMID: 21067377.

11. Yasuda T, Ueno T, Fukumura K, Yamato A, Ando M, Yamaguchi H, Soda M, Kawazu M, Sai E, Yamashita Y, Murata M, Kiyoi H, Naoe T, Mano H. Leukemic evolution of donor-derived cells harboring IDH2 and DNMT3A mutations after allogeneicstem cell transplantation. Leukemia. 2014 Feb;28(2):426-8. doi: 10.1038/leu.2013.278. Epub 2013 Sep 26. PubMed PMID: 24067491.

12. Gogna R, Shee K, Moreno E. Cell Competition During Growth and Regeneration. Annu Rev Genet. 2015;49:697-718. Epub 2015/12/04. doi: 10.1146/annurev-genet-112414-055214. PubMed PMID: 26631518.

13. Amoyel M, Bach EA. Cell competition: how to eliminate your neighbours. Development. 2014;141(5):988- 1000. Epub 2014/02/20. doi: 10.1242/dev.079129. PubMed PMID: 24550108; PubMed Central PMCID: PMCPMC3929405.

14. Petrova E, Lopez-Gay JM, Rhiner C, Moreno E. Flower-deficient mice have reduced susceptibility to skin papilloma formation. Dis Model Mech. 2012;5(4):553-61. Epub 2012/03/01. doi: 10.1242/dmm.008623. PubMed PMID: 22362363; PubMed Central PMCID: PMCPMC3380718.

15. Petrova E, Soldini D, Moreno E. The expression of SPARC in human tumors is consistent with its role during cell competition. Commun Integr Biol. 2011;4(2):171-4. Epub 2011/06/10. doi: 10.4161/cib.4.2.14232. PubMed PMID: 21655431; PubMed Central PMCID: PMCPMC3104570.

16. Merino MM, Rhiner C, Portela M, Moreno E. “Fitness fingerprints” mediate physiological culling of unwanted neurons in Drosophila. Curr Biol. 2013;23(14):1300-9. Epub 2013/07/03. doi: 10.1016/j.cub.2013.05.053. PubMed PMID: 23810538.

**Ethics Approval**

All animal studies performed in this research are approved by Champalimaud Institutional Review Board and the Portuguese Gov Ethical board (DGAV), the approval number is 0421/000/000.

**Fig. 1 (abstract P201).**
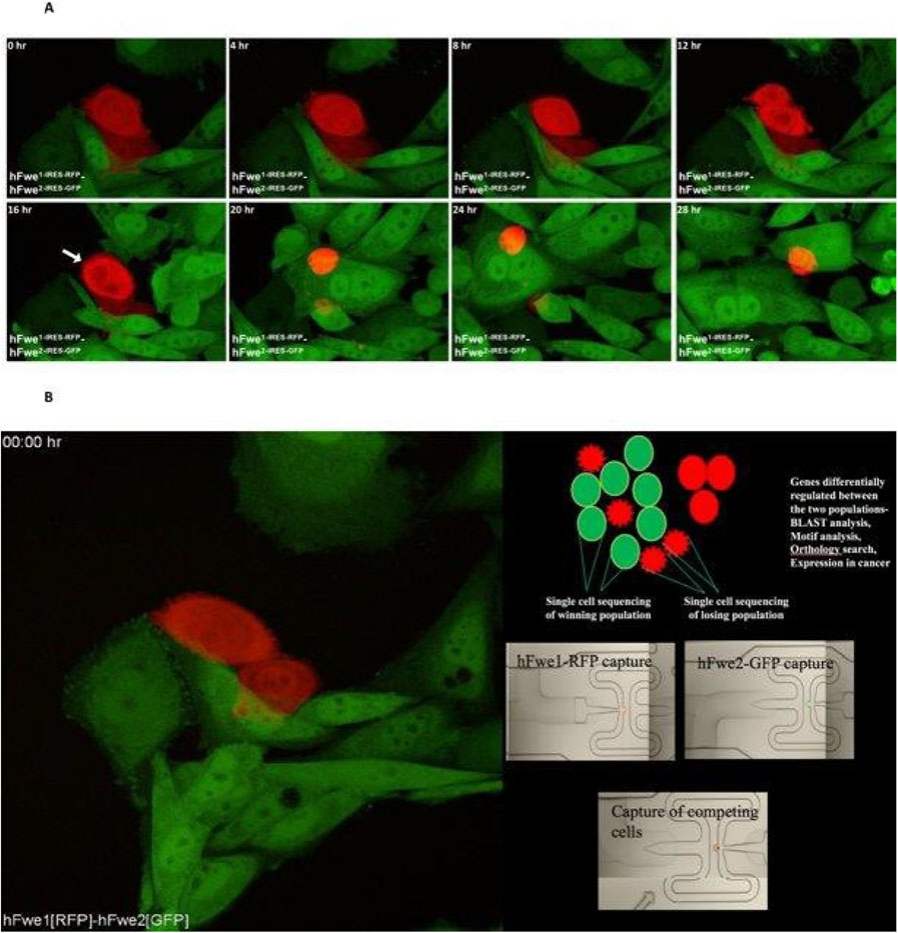
See text for description.

#### P202 Targeting the stress response kinase GCN2 to restore immunity in the tumor microenvironment

##### Lisa Marshall, Buvana Ravishankar, Lavanya Adusumilli, Mikhail Zibinsky, PhD, Deepa Pookot, Emily Huang, Oezcan Talay, PhD, Silpa Suthram, Jeffrey Jackson, Grant Shibuya, Akinori Okano, Paul Leger, Scott Jacobson, BS, Steve Wong, Sherra Johnson, Parcharee Tivitmahaisoon, Angela Wadsworth, BA, Jerick Sanchez, Martin Brovarney, David Chian, BA, Abood Okal, PhD, Delia Bradford, Christophe Colas, Andrea Kim, Gene Cutler, PhD, Jacob Schwartz, David Wustrow, PhD, Paul Kassner, PhD, Dirk Brockstedt

###### FLX Bio, Inc, South San Francisco, CA, USA

####### **Correspondence:** Buvana Ravishankar (bravishankar@flxbio.com)

**Background**

The tumor microenvironment (TME) is characterized by deficiencies in oxygen and key nutrients, such as glucose and amino acids. Stromal cells and Myeloid-derived suppressor cells (MDSC) within the tumor create a nutrient-poor environment that inhibits immune function and supports tumor growth [1]. GCN2 (general control nonderepressible 2), a stress response kinase, plays a key role in sensing and modulating the response to amino acid deprivation. GCN2 activation leads to an induction of the integrated stress response pathway in T cells leading to T cell anergy and apoptosis [2,3]. Here, we demonstrate that the pharmacologic inhibition of GCN2 restores the T cell proliferation and effector function in amino-acid deficient media and in MDSC-induced T cell suppression.

**Methods**

Mouse and human T cell viability, proliferation and function were assessed in vitro under amino-acid deprived conditions and in a co-culture with MDSCs. Pharmacodynamic markers including phospho-GCN2, phospho-EIF2α, and ATF4 were measured via western blot. Cell proliferation (CFSE dye dilution) and effector markers (IFNγ and Granzyme B) were measured by flow cytometry. Our FLX selective, sub-micro molar GCN2 inhibitor (GCN2i) was used to examine the role of GCN2 in T cell and MDSC function.

**Results**

Culturing mouse or human CD8+ T cells under low L-Tryptophan (TRP) conditions activated the GCN2/eIF2α/ATF4 pathway as shown by increased phospho-GCN2 and phospho-eIF2α levels, and enhanced ATF4 protein expression. GCN2/eIF2α pathway suppressed eIF2α phosphorylation and inhibited the increase in ATF4. Under these low TRP conditions or in MDSC co-culture, a GCN2i resulted in rescue of CD8+ T cell proliferation and improvement of cell viability. Additionally, increased levels of IFNγ and Granzyme B were observed in the presence of GCN2i.

**Conclusions**

The GCN2/eIF2α pathway is activated in immune cells during amino acid deprivation, and this induces a functional suppression of the immune response. Our results demonstrate that inhibition of GCN2 is an attractive approach for relieving immune suppression and promotion of T effector activation in the TME

**Acknowledgements**

Cesar Meleza, Minna Bui and Nathan Kozon

**References**

1. Turley S, Cremasco V, Astarita J. Immunological hallmarks of stromal cells in the tumor microenvironment. Nature Reviews Immunology. 2015: 15: 669–682.

2. Munn DH, Sharma MD, Baban B, Harding HP, Zhang Y, Ron D, Mellor AL. GCN2 kinase in T cells mediates proliferative arrest and anergy induction in response to indoleamine 2,3-dioxygenase. Immunity. 2005; 22(5): 633- 42.

3. Rodriguez P, et al. L-arginine deprivation regulates cyclin D3 mRNA stability in human T cells by controlling HuR expression. Journal of immunology. 2010; 185.9: 5198–5204.

**Ethics Approval**

The study was approved by FLX Bio's IACUC committee, approval number FL0002

#### P203 Immunomodulatory effects through inhibition of monocarboxylate transporters in melanoma

##### Satish Noonepalle, PhD^1^, Jennifer Kim, PhD^2^, Namratta Manhas, PhD^1^, Sophiya Ephrame, BS^1^, Erica Palmer, BS^1^, Melissa Hadley, MS^1^, Vincent Sandanayaka, PhD^3^, Alejandro Villagra, PhD^1^

###### ^1^George Washington University, Washington DC, DC, USA; ^2^Brigham and Women's Hospital, Boston, MA, USA; ^3^Nirogyone Therapeutics LLC, Worcester, MA, USA

####### **Correspondence:** Alejandro Villagra (avillagra@gwu.edu)

**Background**

Solid tumors under hypoxic conditions rely on glycolysis rather than oxidative phosphorylation for energy needs thereby generating lactate as a metabolic byproduct. Lactate is transported across the tumor cell membrane using monocarboxylate transporters, MCT1 & MCT4. Lactate is known to suppress immune cell function especially the cytotoxic CD8+ T-cells and NK cells resulting in local immune suppression of the tumor microenvironment (TME). Additionally, acidosis of local TME through lactate/H+ results in inflammation and angiogenesis by activation of VEGFR signaling. The net result of an increased lactate in TME is that it creates a conducive milieu for tumor growth and metastasis. Studies thus far have shown that MCT inhibitors mitigate the effects of lactate and promote immune function; however, the effect of lactate on immune cells in the context of tumor infiltration, immune cell composition, lineage differentiation and cytokine profiles are yet to be explored.

**Methods**

In this study, we used a novel MCT1/4 dual inhibitor NGY-A to demonstrate the effect of suppressing lactate in the local TME and restoring the anti-tumor immunity. In vivo experiments were performed with syngeneic mouse model of SM1 melanoma cells in C57BL/6 mice treated with NGY-A at a dosage of 10mg/kg administered intraperitoneally 5 days/week for 2 weeks. Total protein and RNA was extracted to perform immunoblot and real time PCR analyses. Expression of MCT1/4 was analyzed using skin cancer melanoma datasets on Cbioportal and R2 genomics platforms.

**Results**

Our preliminary data indicates that (1) MCT1/4 expression is upregulated in a subset of melanoma patients and it is associated with poor survival (2) In vivo analysis of SM1 melanoma mouse model in C57BL/6 mice, treatment with NGY-A significantly delayed tumor growth compared to the control group. (3) Analysis of tumors revealed that expression of immune suppressive B7 family genes Cd274 (PD-L1), Cd276 (B7-H3) and Lgals9 (Galactin-9) were significantly down regulated at both RNA and protein levels in NGY-A treated group compared to the control group. (4) Cytokine expression profiling of NGY-A treated tumors by RT-PCR indicated an increase in anti-tumor cytokines IFNγ, TNFα, IL-1β and decrease in pro-tumor cytokines TGFβ, IL-10 compared to control group tumors suggesting that NGY-A treatment can increase anti-tumor CD8+ T-cells, M1 macrophages and suppress tumor promoting M2-phenotype.

**Conclusions**

Our preliminary data strongly suggests that intervention of lactate transporters MCT1/4 with NGY-A exhibits significant anti-tumor effect by positively modulating the local TME to promote immune cell function.

#### P204 Characterization of novel dual A2A_A2B adenosine receptor antagonists for cancer immunotherapy

##### Mateusz Nowak, Michal Galezowski, Paulina Wegrzyn, Aneta Bobowska, Katarzyna Dziedzic, Joanna Szeremeta-Spisak, Marcin Nowogrodzki, Grzegorz Satala, Alicja Obara, Iwona Lozinska, Marcelina Dudek, Anita Janiga, Jacek Reus, Marek Wronowski, Magdalena Zastawna, Grzegorz Statkiewicz, Maciej Rogacki, Mateusz Swirski, Jakub Woyciechowski, Magdalena Ziembik, Karolina Grycuk, Foteini Soukou, Agnieszka Adamus, Karolina Wiatrowska, Natalia Lewandowska, Aniela Golas, Olga Pierzchala, Roderick Porter, Krzysztof Brzozka

###### Selvita S.A, Krakow, Poland

####### **Correspondence:** Krzysztof Brzozka (krzysztof.brzozka@selvita.com)

**Background**

Adenosine functions as a messenger molecule in many tissues including central nervous and cardiovascular systems. Recently the attention of researchers was attracted to immunological aspects of adenosine signaling and its involvement in the suppression of immune response in tumor microenvironment. Adenosine inhibits the biological functions of T lymphocytes infiltrating the cancer tissue by binding to the A2A receptor. The affinity to A2B receptor is believed to attenuate the response of innate system ie: dendritic cells. Thus blocking of both A2A and A2B signaling seems to be a viable approach for either standalone therapy or combination with other immunomodulating agents.

**Methods**

The activation of A2a R and A2b R increases intracellular levels of cAMP, subsequent phosphorylation of the CREB protein, which translates to lower excretions of certain types of cytokines by specific populations of primary cells. To assess the potency of antagonists under tumor like conditions either 100uM adenosine or 10uM synthetic adenosine agonist (NECA) was used. cAMP level was measured by TR-FRET based method. Cytokines levels were measured by alphaLISA method. Flow cytometry was used to measure adenosine related inhibition of CREB phosphorylation on T cells and B cells in blood harvested form animals dosed with our antagonist and ex vivo treated with NECA.

**Results**

We have developed a novel series of potent and dual A2A/A2B antagonists that retain its nanomolar potency in tumor-like adenosine rich environment. Our antagonists restore the cytokine release by activated CD4+ and CD8+ human T-lymphocytes after treatment with high concentrations of adenosine. We observe also the reversal of functional adenosine-induced suppression of NK cells. Most importantly presented compounds show improved pharmacological profile in comparison to A2A inhibitors tested in clinical trials. Currently our most advanced lead A2A/A2B inhibitors undergo an extensive in vivo efficacy and safety characterization.

**Conclusions**

Due to the abnormally elevated adenosine levels in tumor microenvironment, the efficacious adenosine receptor antagonist should be able to displace adenosine even in very high concentrations. We have demonstrated several in vitro models that dual A2A/A2B antagonists discovered in Selvita are able to reverse the immune suppression induced by adenosine concentration corresponding to those present in tumors.

#### P205 Preclinical and initial phase I clinical characterization of CPI-006: an anti-CD73 monoclonal antibody with unique immunostimulatory activity

##### Emily Piccione, PhD^1^, Andrew Hotson, PhD^1^, Glen Mikesell, BS^1^, Barbara Daine-Matsuoka, BS^1^, Chunyan Gu^1^, Trang Dao-Pick^1^, Craig Hill, PhD^1^, Antonett Madriaga^1^, Jennifer Rudnick^1^, Liang Liu^1^, W Jones^1^, Linda Hammerich, PhD^2^, Joshua Brody, MD^2^, Ginna Laport^1^, Richard Miller, MD^1^, Joseph Buggy^1^

###### ^1^Corvus Pharmaceuticals, Burlingame, CA, USA; ^2^Icahn School of Medicine at Mount Sinai, New York, NY, USA

####### **Correspondence:** Emily Piccione (epiccione@corvuspharma.com)

**Background**

The ecto-5’-nucleotidase CD73 generates immunosuppressive adenosine and functions as a co-stimulatory protein on lymphocytes [1]. CPI-006 is a humanized, FcγR binding-deficient anti-CD73 that fully inhibits enzymatic activity and does not induce antigen internalization. We report in vitro mechanistic studies, in vivo studies in cynomolgus monkeys, and preliminary data from an ongoing Phase 1/1b clinical trial.

**Methods**

CPI-006 was evaluated in vitro in human peripheral blood mononuclear cells (PBMCs) or purified B cells. Safety and toxicokinetic studies were performed in 16 cynomolgus monkeys; CPI-006 was dosed IV once weekly for five weeks up to 120 mg/kg. CPI-006 was given by IV infusion every 3 weeks in a Phase 1/1b trial (NCT03454451) to evaluate safety and biologic effects in advanced cancer patients.

**Results**

CPI-006 completely inhibited conversion of AMP to adenosine (IC50, 17nM). Blocking adenosine production with CPI-006 enhanced immune-cell mediated tumor killing (220pM) and reversed suppression of T cell proliferation (EC50, 70nM) and IFNγ secretion (EC50, 66nM). In vitro treatment of normal PBMCs or purified B cells with CPI- 006 induced B lymphocyte activation leading to ERK phosphorylation and increased CD69, CD25, and CD83 expression. B cell activation was independent of adenosine and completely blocked by the BTK inhibitor ibrutinib (0.1μM). No B cell death or apoptosis was observed. Cynomolgus monkeys showed complete occupancy of CD73 on CD8+ T cells in peripheral blood up to 7 days post-treatment at doses ≥ 10 mg/kg. The NOAEL was 120 mg/kg, the highest dose tested. CPI-006 was administered to five cancer patients at 1 or 3 mg/kg (data cutoff July 30, 2018). Serum levels of CPI-006 were detected up to 24 hours post-dose with complete elimination by day 8. Full target occupancy was achieved 0.5 hours after dosing with no occupancy by day 15, concordant with serum levels of antibody. At 0.5 hours, a decrease in peripheral B cells (median reduction 60%, range 33-82%), but not T cells, was observed in all five patients. B cell depletion was transient and was restored to baseline levels by day 15, consistent with the pharmacokinetics. No grade 3/4 adverse

**Conclusions**

CPI-006 both inhibits the enzymatic activity of CD73 and stimulates an intracellular signal to activate B cells.

Expression of CD69 and reversible reduction of circulating B cells is consistent with B cell activation and redistribution to lymphoid tissues through inhibition of S1P1 receptors [2]. These effects may have immunotherapeutic potential for patients with cancer.

**Trial Registration**

NCT03454451

**References**

1. Resta R, Yamashita Y, and Thompson LF. Ecto-enzyme and signaling functions of lymphocyte CD73. Immunological Revs. 1998; Vol 161: 95-109.

2. Shiow LR, Rosen DB, Brdickova N, Xu Y, An J, Lanier LL, Cyster JG, and Matloubian M. CD69 acts downstream of interferon-a/b to inhibit S1P1 and lymphocyte egress from lymphoid organs. Nature. 2006; Vol 440: 540-544.

#### P206 Using clear cell like-RenCa and papillary like-RenCa models of Kidney Cancer to study metabolic influences on the microenvironment and metastasis

##### Bradley Reinfeld, BA^1^, Katy Beckermann^2^, W. Rathmell, MD, PhD^2^, Peter Siska^3^, Jeffrey Rathmell, PhD^1^, Melissa Wolf^1^, Kirsten Young^1^, Gabriella Andrejeva, PhD^1^, Jamie Weyandt^4^

###### ^1^Vanderbilt University School of Medicine, Nashville, TN, USA; ^2^Vanderbilt University Medical Center, Nashville, TN, USA; ^3^University Hospital Regensburg, Nashville, TN, USA; ^4^Aegis, Nashville, TN, USA

####### **Correspondence:** Bradley Reinfeld (brad.reinfeld@gmail.com)

**Background**

The genetics of renal cell carcinoma (RCC) surround loss of function mutations in genes that regulate metabolism.

The most common genetic event in clear cell RCC (ccRCC) is loss of Von Hippel Lindau (VHL). In type II papillary RCC, 20% of patients lose key Krebs cycle enzyme, fumarate hydratase (FH). Through separate mechanisms, both mutations result in elevated hypoxia related signaling and demonstrate glycolytic phenotypes. This project focuses on how these mutational events create an immunoinhibitory metabolic milieu that ultimately promotes tumor establishment, progression, and metastasis.

**Methods**

Previously our lab has demonstrated differences in tumor interstitial fluid composition when comparing adjacent normal kidney parenchyma and RCC malignant tissue via 13C NMR. This work surrounds modeling the abnormal metabolic microenvironment of kidney cancer in relation to genetic features of the major classification sof RCC in vitro. By combing the OT-1 model antigen system with novel ribonucleic CRISPR/Cas9 approaches, we have a more genetically relevant tumor model whose elimination is antigen dependent. This approach has resulted in the establishment of clear cell-like RenCas (containing VHL knock out) and papillary-like RenCas (containing FH KO).

**Results**

From a metabolic standpoint, these cell lines both demonstrate the phenotypes of HIF elevated tumors with significantly increased glucose uptake, lactate production and glutaminolysis, but with varying metabolic demands. Additionally both KO cell lines accumulate lipid droplets via Oil Red O and electron microscopy. The FH-KO RenCas form larger tumors when grown in immunocompetent mice then their vector control. Intriguingly, this significant difference disappear is Rag deficient mice. By using these novel cell lines, we can evaluate how clinically relevant genetic events result in metabolic changes in the tumor microenvironment impacting immune cells and potential responsiveness to immunotherapy.

**Conclusions**

Our lab has developed novel immunocompetent models of RCC, which will allow us to investigate how tumor metabolic demands promote dysfunctional immune phenotypes. In doing so, we hope to find new synergistic therapeutic combinations that will increase the efficacy of immune checkpoint blockade via altering the metabolic components of the tumor microenvironment.

#### P207 Chronic T cell activation and metabolic stress promote the exhausted T cell state by inducing epigenetic inflexibility

##### Nicole Scharping, BS, Nicole Scharping, BS, Natalie Rittenhouse, Ashley Menk, BS, Ronal Peralta, Paolo Vignali, BA, Roderick O'Sullivan, Amanda Poholek, Greg Delgoffe, PhD

###### University of Pittsburgh, Pittsburgh, PA, USA

####### **Correspondence:** Greg Delgoffe (delgoffeg@upmc.edu)

**Background**

CD8+ tumor-infiltrating T lymphocytes (TIL) progressively succumb to a dysfunctional state known as ‘exhaustion’, characterized by poor effector function and sustained co-inhibitory marker expression, but the factors causing this hyporesponsive phenotype remain unclear. We recently demonstrated that exhausted TIL also exhibit progressive loss of functional mitochondria, due in part to repression of the transcriptional co-activator PGC1α, resulting in suppressed mitochondrial fusion and biogenesis. Enforcing PGC1α expression in tumor-specific T cells not only led to increased mitochondria, but improved functionality, decreased tumor burden, and increased survival in mouse B16 melanoma. These results lead us to hypothesize that T cell exhaustion may be driven by metabolic insufficiency.

**Methods**

To characterize both the drivers and consequences of T cell exhaustion, we developed models to identify how tumor-derived signals – hypoxia and chronic T cell receptor activation – induce metabolic insufficiency and T cell exhaustion *in vitro*. These *in vitro* findings were analyzed in correlation with *ex vivo* exhausted TIL from mouse B16 melanoma for comparison.

**Results**

Exploring the drivers of T cell exhaustion, we found T cells experiencing chronic activation or hypoxia alone < i >in vitro< /i > could carry out similar or superior effector functions, but experiencing both chronic activation and hypoxia simultaneously generated profound, persistent T cell dysfunction. This dysfunction was characterized by high PD-1, Tim-3, and Lag3 expression, loss of IFNγ cytokine production, decreased oxygen consumption due to loss of functional mitochondria, and required the transcriptional repressor Blimp1. Our studies suggest metabolic insufficiency does not contribute to exhaustion energetically, but rather causes alterations in epigenetic remodeling both in our < i >in vitro< /i > exhaustion assay and in TIL, leading to increased repressive histone methylation and decreased expression of essential T cell effector genes. We next identified a mechanism for this hypermethylation signature and found TIL have excessive mitochondrial reactive oxygen species (ROS), which caused DNA damage. Consequently, we metabolically reprogrammed tumor-specific T cells and found metabolic reprogramming was sufficient to reduce ROS, reduce repressive chromatin methylation, and prevent DNA damage.

**Conclusions**

Our findings support a model in which chronic activation drives transcriptional repression, fundamentally altering how T cells respond to hypoxic conditions and exposing a mechanism of T cell exhaustion. These data reveal potential metabolic avenues to rescue exhausted T cells and improve immunotherapy.

#### P208 Metabolic reprogramming augments CAR T cell function

##### Yiyang Wang, N/A^1^, Jason Lohmueller, PhD^2^, McLane Watson, BS^2^, Dayana Rivadeneira, PhD^2^, Greg Delgoffe, PhD^1^

###### ^1^UPMC Hillman Cancer Center, Pittsburgh, PA, USA; ^2^University of Pittsburgh, Pittsburgh, PA, USA

####### **Correspondence:** Greg Delgoffe (delgoffeg@upmc.edu)

**Background**

CAR T immunotherapy has emerged as an exciting approach for cancer treatment. Remarkable success has been demonstrated in the treatment of hematologic malignancies, yet to be replicated in solid tumors. This is thought to be due to many hurdles including tumor-associated immune suppression and alterations in the tumor metabolic environment. Current preclinical CAR T research has taken advantage of tumor graft models in immunodeficient mice, which may lead to neglect of the effect posed by the immune system. Our aim is to establish a fully murine model with mouse CAR T cells against antigens expressed in murine solid tumors. Furthermore, to combat the metabolic restrictive tumor microenvironment (TME), we attempt to develop gene-therapy strategies to make metabolically superior CAR T cells.

**Methods**

Murine CAR constructs containing the single-chain variable fragment that can recognize human CD19 were synthesized and transduced into mouse CD8+ T cells to generate murine CAR T cells. MC38 cells, (derived from murine adenocarcinoma) were modified to overexpress human CD19 and intradermally inoculated onto immunocompetent mice to establish the model. CAR T cells were first tested by in vitro cytotoxicity assay, then adoptively transferred into tumor bearing mice for tumor-infiltrating lymphocyte analysis. Retroviral transduction of genes involved in the mitochondrial biogenesis pathway served to reprogram these CAR T cells, which were further tested by bioenergetic assays including Seahorse extracellular flux analysis.

**Results**

In our murine model, tumor-infiltrating CAR T cells manifest an insufficient metabolic phenotype with repressed glucose uptake and loss of mitochondria mass. Their ability to produce antitumor cytokines is also suppressed.

Retroviral overexpression of Ppargc1a (encoding PGC1α) and Tfam (Transcription Factor A, Mitochondrial) in CAR T cells increases their basal oxygen consumption rate (both genes) and spare respiratory capacity (Ppargc1a), while leading to an elevated expression of markers for central memory phenotype, suggesting their potential to perform better antitumor function in the TME. Preliminary data in the treatment of established MC38 tumor suggests that mice treated with metabolically reprogrammed CAR T cells have slower tumor growth compared to those treated with normal CAR T cells.

**Conclusions**

CAR T cells that infiltrate solid tumors function at a metabolic disadvantage. Metabolic reprogramming of CAR T cells to favor mitochondrial biogenesis increases their respiratory capacity, thus enhancing the effector function and longevity of these cells in the tumor microenvironment. The utilization of gene therapy targeting metabolism of T cells may improve the efficacy of adoptive T cell therapies for solid malignancy.

**Ethics Approval**

Animal work was done in accordance with the Institutional Animal Care and Use Committee of the University of Pittsburgh.

#### P209 Antagonism of peroxisome proliferator activated receptor alpha by TPST-1120 suppresses tumor growth and stimulates anti-tumor immunity

##### Chan Whiting, PhD^1^, Nick Stock, PhD^2^, Davorka Messmer, PhD^2^, Austin Chen, PhD^2^, Lisa Rahbaek, PhD^2^, Allison Gartung, PhD^3^, Karin Stebbins, PhD^2^, Traci Olafson^2^, Alex Broadhead^2^, Ryan Clark, PhD^2^, Catherine Lee, MS^2^, Chris Baccei^2^, Dan Lorrain, PhD^2^, Alicia Levey, PhD^1^, Derek Metzger, BA^1^, Amanda Enstrom, PhD^1^, Jennifer McDevitt, PharmD, PhD^1^, David Spaner, MD, PhD^4^, Peppi Prasit, PhD^2^, Dipak Panigrahy, MD^3^

###### ^1^Tempest Therapeutics, San Francisco, CA, USA; ^2^Inception, San Diego, CA, USA; ^3^BIDMC, Boston, MA, USA; ^4^Sunnybrook, Toronto, Canada

####### **Correspondence:** Chan Whiting (cwhiting@tempesttx.com)

**Background**

TPST-1120 is a first-in-class selective antagonist of human PPARα, a transcription factor that induces expression of fatty acid oxidation (FAO) genes. Metabolic adaptions promote tumor survival and suppress tumor-specific immunity by upregulation of FAO. Results from multiple syngeneic and xenograft mouse models suggest PPARα blockade has intrinsic and extrinsic anti-tumor activity and induces tumor-specific immunity.

**Methods**

The effects of TPST-1120 as monotherapy or in combination with chemotherapy or anti-PD1 were evaluated in multiple syngeneic mouse models including B16 melanoma, MMTV mammary carcinoma, MC38 colon, Lewis lung carcinoma, ID8 ovarian, Panc07 pancreatic cancer, and xenograft CLL, melanoma, pancreatic and AML models. To characterize its mechanism of anti-tumor immunity, TPST-1120 was evaluated in knock-out models of CCL2, MBL, TSP-1, STING and BatF3. Immune modulation was characterized by M2/M1 macrophage flow cytometry phenotyping and ELISA measurement of plasma and tumor matrix protein thrombospondin-1 (TSP-1), which is involved in granulocyte migration and angiogenesis.

**Results**

TPST-1120 mediated PPARα antagonism resulted in potent anti-tumor immune responses and significant tumor regression, either as a monotherapy or in combination with chemotherapy or anti-PD1. TPST-1120 showed anti- tumor efficacy against syngeneic models of breast, lung, colon, pancreatic and melanoma in addition to xenograft models of CLL, AML, pancreatic and melanoma as monotherapy or with chemotherapy. TPST-1120 demonstrated cytotoxic effect on tumor cells in vitro. In pancreatic and breast cancer models, TPST-1120 together with chemotherapies gemcitabine and eribulin, respectively, had additive effects on controlling tumor growth. TPST- 1120 with anti-PD1 in ovarian orthotopic (ID8) and colon (MC38) models showed suppression of tumor growth and complete remission in some mice compared with either TPST-1120 or the checkpoint inhibitor alone. The combination also conferred protection against autologous tumor re-challenge in the ID8 model, strongly suggesting induction of immunological memory against the primary tumor. Preliminary studies in genetic knock-out mice, suggest macrophages and antigen cross-presenting dendritic cells are required for TPST-1120 activity, potentially through STING activation and TSP-1. Consistent with prior reports of the involvement of PPARα activation in promoting M2 macrophages, TPST-1120 skews toward an M1 effector macrophage phenotype and in vivo pretreated peritoneal macrophages enhance the uptake of whole tumor cells by FACS.

**Conclusions**

Through its unique mechanism of restricting FAO, TPST-1120 targets a metabolic pathway critical for survival of both tumor cells and suppressive immune cell populations infiltrating the tumor microenvironment and represents a promising new approach for patients with advanced malignancies.

### Cellular Therapy Approaches

#### P210 PD-1-positive tumor-infiltrating lymphocytes (TIL) for the next generation of adoptive T cell therapy

##### Michelle Abelson, PhD, Kenneth D'Argio, Angel Cedano-Hilton, Ian Frank, Krit Ritthipichai, DVM, PhD, Cecile Chartier

###### Iovance Biotherapeutics, Tampa Bay, FL, USA

####### **Correspondence:** Cecile Chartier (cecile.chartier@iovance.com)

**Background**

Adoptive T cell therapy with autologous TIL has demonstrated high response rates in patients with metastatic melanoma [1]. TIL products used for treatment are comprised of heterogenous T cells, which recognize tumor- specific antigens, mutation-derived patient-specific neoantigens, and non-cancer related antigens [2, 3]. Among them, neoantigen-specific T cells are main contributors to the anti-tumor activity of TIL [4]. Strategies enriching TIL for such T cells are thus expected to yield more potent therapeutic products, especially in epithelial cancers known to contain a high proportion of bystander T cells [5]. Several studies have demonstrated that expression of PD-1 on TIL identify tumor-specific T cells [6-8]. Presented here is the development of a new process to produce tumor antigen-specific-enriched TIL products for clinical application.

**Methods**

PD1-positive (PD1+) cells were sorted via flow cytometry directly from fresh tumor digests and expanded in vitro.

Samples from 4 melanomas, 3 sarcomas, 3 breast cancers, and 2 lung cancers were evaluated. Sorted PD-1+ TIL- derived product, sorted PD-1- TIL-derived product, and whole tumor digest (unsorted)-derived product were compared for cell count, phenotype, function, TCR Vβ repertoire, and tumor reactivity.

**Results**

PD-1+ cells, ranging between 1-90% of the CD3+ cells, were isolated from tumor digests. Upon expansion, the PD-1+ cells proliferated 3-fold less than the PD-1 cells, and high TIL numbers were obtained in 9 of the 12 products. Phenotypic analyses revealed no significant differences in terms of T cell lineages and memory subsets, or expression of various activation, differentiation, and exhaustion markers between the 3 types of products. The PD1+ TIL-derived products responded to PMA and to anti-CD3 stimulations by inducing CD107a mobilization and IFN gamma secretion to extents similar to the control products. Profiling of the TCRvβ repertoire demonstrated that PD- 1+ TIL-derived CD8+ cells displayed greater oligoclonality than their PD1- counterparts, likely reflecting antigen- driven clonal expansion at the tumor site. Whether this oligoclonal expansion will translate into superior tumor- specific recognition and killing is the subject of further investigation.

**Conclusions**

A process has been developed for the expansion of PD1+ TIL from a variety of histologies. Resulting products were shown to be phenotypically and functionally comparable with bulk TIL products. Consistent with prior reports [6, 8], our results suggest that in vitro expansion of PD1+ TIL can restore their effector functions and support the development of PD1+ TIL-derived product for the treatment of cancer.

**References**

1. Rosenberg SA, et al. Durable complete responses in heavily pretreated patients with metastatic melanoma using T-cell transfer immunotherapy. Clin Cancer Res. 2011; 7(13):4550-7.

2. Kvistborg P, et al. TIL therapy broadens the tumor-reactive CD8(+) T cell compartment in melanoma patients. Oncoimmunology. 2012;1(4):409-418.

3. Simoni Y, et al. Bystander CD8(+) T cells are abundant and phenotypically distinct in human tumour infiltrates. Nature. 2018; 557(7706):575-579.

4. Schumacher TN, Schreiber RD. Neoantigens in cancer immunotherapy. Science. 2015; 348(6230): 69-74.

5. Turcotte S, et al. Phenotype and function of T cells infiltrating visceral metastases from gastrointestinal cancers and melanoma: implications for adoptive cell transfer therapy. J Immunol. 2013; 191(5): 2217-25.

6. Inozume T, et al. Selection of CD8+PD-1+ lymphocytes in fresh human melanomas enriches for tumor-reactive T cells. J Immunother. 2010; 33(9): 956-64.

7. Gros A, et al. PD-1 identifies the patient-specific CD8(+) tumor-reactive repertoire infiltrating human tumors. J Clin Invest, 2014; 124(5): p. 2246-59.

8. Thommen DS, et al. A transcriptionally and functionally distinct PD-1(+) CD8(+) T cell pool with predictive potential in non-small-cell lung cancer treated with PD-1 blockade. Nat Med. 2018.

#### P211 Oxygen and pressure promote primary T cell expansion and can regulate population dynamics and cytokine release

##### Yunmin Li, PhD, Zachary Pappalardo, Mauricio Montano, Ann Lu, Bruce Adams

###### Xcell Biosciences Inc., San Francisco, CA, USA

####### **Correspondence:** Bruce Adams (bruce@xcellbio.com)

**Background**

T cell immunotherapy workflows typically require expansion of autologous T cells or genetic modification of T cells in vitro, followed by infusing of those cells back into the patients. Typically, T cells or genetically modified T cells are expanded using CD3 and CD28-based activation under standard cell culture conditions (18-20% O2). However, this approach is not always ideal or preferred because, for example, some donors fail to expand. Furthermore, expansion of T cells using activating agents can change cell phenotypes and states, and the state of cells could contribute to risk elements such as cytokine release syndrome. Because T cells in the body experience a broad range of O2 levels (ranging from 13%O2 in arterial blood to 5%O2 in venous blood), as well as a range of blood pressures (60 mmHg/1.2 PSI, 120 mmHg/2.3 PSI), we studied how environmental control might be used to optimize T cell growth and phenotype in workflows including: 1) activation-based expansion, 2) standard expansion, and 3) post-transfection viability.

**Methods**

To address expansion, human CD3+ T cells were seeded in expansion medium supplemented with IL2 (for activation, we used CD3 and CD28-based stimulation). A range of O2 concentrations and forces were tested for the ability to influence T cell growth and phenotype for up to 14 days using the AVATAR™ cell control system. Next, to address the issue of post-transfection viability, activated T cells were transfected by electroporation, and were recovered under a variety of different conditions and assessed for viability and expansion.

**Results**

CD3+ T cells grew faster, both without or with activation, resulting in as much as 51% more T cells compared to standard incubation at 2 weeks. T cells cultured under different O2 and pressure levels showed different proliferation rates but in general, T cell grew faster and had different cytokine secretion profiles when additional pressure was applied n the culture. In the case of transfection, we were able to improve post-transfection viability by as much as 35% with more than twice the number of cells after three days compared to those cultured under standard recovery conditions.

**Conclusions**

Environmental influences such as oxygen and pressure can be used to optimize expansion and/or state of T cells, including in difficult to grow samples, or cells that have been stressed by upstream manipulations such as transfection. This control can also be used to study phenotypic changes in cytokines release, metabolic markers and checkpoint receptor expression.

#### P212 Preinfusion product doubling time is associated with CAR T cell expansion and outcomes in ZUMA-1, the pivotal study of axicabtagene ciloleucel (axi-cel) in refractory large B cell lymphoma

##### Tiffany Adolf^1^, Frederick Locke, MD^2^, John Rossi, MS^3^, Caron Jacobson, MD^4^, David Miklos, MD^5^, Armin Ghobadi, MD^6^, Olalekan Oluwole, MBBS, MPH^7^, Lazaros Lekakis, MD^8^, Patrick Reagan, MD^9^, Yizhou Jiang, PhD^3^, Lianqing Zheng, PhD^3^, William Go, MD, PhD^3^, Adrian Bot, MD, PhD^3^

###### ^1^Nexus GG Science LLC; ^2^Moffitt Cancer Center, Tampa, FL, USA; ^3^Kite, a Gilead Company, Santa Monica, CA, USA; ^4^Dana-Farber Cancer Institute, Boston, MA, USA; ^5^Stanford University School of Medicine, Stanford, CA, USA; ^6^Washington University School of Medicine, St. Louis, MO, USA; ^7^Vanderbilt-Ingram Cancer Center, Nashville, TN, USA; ^8^University of Miami Health System, Miami, FL, USA; ^9^University of Rochester Medical Center, Rochester, NY, USA

####### **Correspondence:** John Rossi (jrossi@kitepharma.com)

**Background**

Axi-cel, an FDA-approved autologous anti-CD19 chimeric antigen receptor (CAR) T cell therapy, demonstrated an 82% objective response rate (ORR), including a 58% complete response (CR) rate in patients with refractory large B cell lymphoma (median 15.4 months follow-up) [1]. Grade ≥3 cytokine release syndrome (CRS) and neurologic events occurred in 12% and 31% of patients, respectively. This analysis examined associations of preinfusion product characteristics with CAR T cell expansion and clinical outcomes.

**Methods**

ORR and blood CAR T cell levels (peak and area under the curve from days 0–28 [AUC0-28] in ZUMA-1 were examined for associations with axi-cel product cell population doubling time (DT), a measure of preinfusion product T cell expansion kinetics. DT, measured between day 3 and final day of manufacturing, depends on the rates of cell proliferation and death during incubation with recombinant interleukin (IL)-2–supplemented medium. Major product T cell phenotypes were evaluated by flow cytometry. Associations were evaluated using logistic regression (nominal P values <.05 considered meaningful associations; not adjusted for multiplicity) and pairwise Spearman analysis (rs values) and visualized using quartile analysis bar charts and logistic regression predicted probability curves.

**Results**

Patients treated with products with shorter DT had higher ORR (P=.025; Figure A-B). Patients in the lowest product DT quartile (DT≤1.33 days) had 100% ORR. Patients in the highest product DT quartile (DT≥1.79 days) had 73% ORR. DT was also negatively associated with greater CAR T cell expansion post-infusion (peak CAR T cell levels, rs = -0.27; AUC0-28, rs = -0.29; quartile analysis, Figure C). Of 17 nonresponders, 12 had product DT>1.5 days. A precise CD4/CD8 T cell ratio of 1:1 was not required for achieving lowest DT and maximal CAR T cell expansion or ORR. Additional analyses, including associations between product DT, T cell phenotypes, and other clinical outcomes including toxicities and durable response, will be presented.

**Conclusions**

Preinfusion product T cell expansion kinetics, as measured by DT during manufacturing in presence of IL-2–supplemented medium, may be associated with ORR and in vivo CAR T cell expansion in patients treated with axi- cel. Poor product DT may limit in vivo CAR T cell expansion. Indices related to product DT, a component of product T cell fitness, may be useful in optimizing CAR T cell therapy.

**Trial Registration**

NCT02348216

**References**

1. Neelapu SS, Locke FL, Bartlett NL, et al. Axicabtagene ciloleucel CAR T-cell therapy in refractory large B-cell lymphoma. N Engl J Med. 2017;377:2531-2544.

**Fig. 1 (abstract P212).**
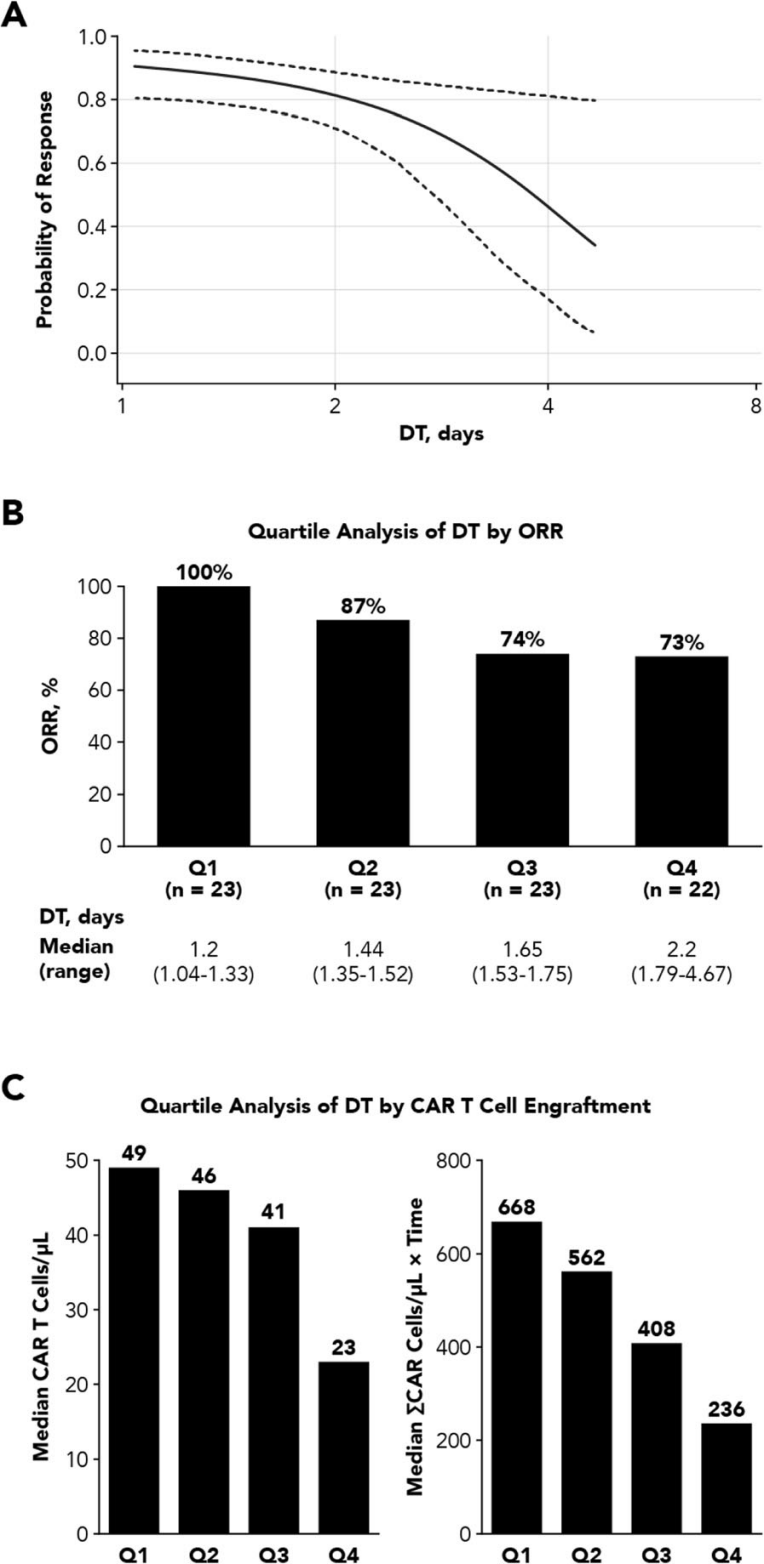
See text for description.

#### P213 CYAD-101: an allogeneic NKG2D CAR T cell therapy using a TCR inhibitory molecule

##### Sophie AGAUGUE, PhD, Alexandre Michaux, PhD, Eytan Breman, MSc, Sebastien Mauen, PhD, David Gilham, PhD

###### Celyad SA, Mont Saint Guibert, Belgium

####### **Correspondence:** Sophie AGAUGUE (sagaugue@celyad.com)

**Background**

Chimeric antigen receptor (CAR) T cells have demonstrated impressive clinical results in B cell malignancies. Most CAR T cell therapies rely on autologous peripheral blood cells that present some challenges including manufacturing time and product variability. Allogeneic T cells derived from a healthy donor may circumvent these issues. However, allogeneic T cells can induce graft versus host disease (GvHD), a response triggered by the recognition of non-self Human Leukocyte Antigen molecules expressed on recipient cells by the T Cell Receptor (TCR) of donor cells. To avoid GvHD, we targeted the TCR signalling by using a TCR inhibitory molecule (TIM) peptide consisting of a truncated form of CD3zeta. Our mechanistic studies suggest that TIM is acting as a dominant negative form of CD3zeta reducing downstream TCR signaling pathway activity. To assess TIM in the context of a CAR therapy, an allogeneic CAR T cell was developed by co-expressing TIM and a NKG2D-based CAR (referred as CYAD-101).

**Methods**

CYAD-101 CAR T cells were produced from five different healthy donors. In vitro and in vivo experiments have been performed to evaluate the culture parameters, phenotype, alloreactivity and potency against NKG2D ligand- expressing tumor targets.

**Results**

In vitro experiments confirmed the suppression of TCR alloreactivity of the TIM-combined CAR as compared to control cells. However, the CYAD-101 T cells showed variability between donors in terms of CD4/CD8 ratio and percentage of central and effector memory populations potentially relating to the variability of the starting material to produce CAR T cells. In addition, harvested CYAD-101 cells were mainly composed of a non-activated and non- exhausted population (i.e. CD25-/CD69- and PD1-/LAG3-). A concomitant reduction in the level of phospho-ZAP- 70 was observed in cells expressing the TIM. Importantly, in vitro cytotoxicity and cytokine production of CYAD- 101 cells upon co-culture with NKG2D ligand-expressing tumor cell lines was not affected by the insertion of TIM. Finally, compared to control T cells, CYAD-101 cells efficiently delayed in vivo tumor progression and increased survival of NSG mice bearing orthotopic colorectal tumors while avoiding the induction of GvHD.

**Conclusions**

Following these promising preclinical results showing the combination of effective anti-cancer activity and inhibition of alloreactivity of CYAD-101 CAR T cells, a phase I clinical trial will be shortly initiated to assess the safety, cell kinetics and clinical activity of CYAD-101 CAR T cells in patients with unresectable metastatic colorectal cancer.

**Ethics Approval**

Animal studies were approved by VetAgro-Sup / Lyon National Veterinary School ethical committe (project n° APAFIS#8565-2017010413316369 v4) and french authorities.Use of whole blood of healthy donors was approved by local ethical committees.

#### P214 Development of allogeneic gene-edited CAR T-cells: from preclinic to clinical proof of concept

##### Beatriz Aranda Orgilles, PhD, Agnes Gouble, Roman Galetto, PhD, Julianne Smith, Philippe Duchateau, David Sourdive, Laurent Poirot, PhD, Stephanie depil

###### Cellectis, New York, USA

####### **Correspondence:** Stephanie Depil (stephane.depil@cellectis.com)

**Background**

Adoptive immunotherapy using engineered T-cells has emerged as a powerful approach to treat cancer. The potential of this approach relies on the ability to redirect T-cell specificity through ex vivo genetic engineering and transfer of chimeric antigen receptors (CARs) or engineered TCRs. Transduction of patients’ blood cells with an anti-CD19 CAR for the treatment of Acute Lymphoblastic Leukemia (ALL) has led to complete response in the large majority of treated patients and the early approval of two CAR T-cell products. Autologous treatments require a complex manufacturing process and depend on the existence of a healthy T-cell population despite previous heavy chemotherapy treatments. The use of allogeneic cells (derived from healthy donors rather than the patients themselves) allows preparation of cells well ahead of a patient’s need for treatment. Moreover, allogeneic CAR T- cells offer the possibility to characterize in depth the starting material, generate multiple treatment doses from one process and, ultimately, more affordable access to treatment.

**Methods**

Using our proprietary nuclease-based gene editing technologies, we showed our capability to efficiently edit any gene in primary T-cells with very high precision.

**Results**

Here, we describe how TALEN® gene-editing technology allows to create CAR T-cells that can be used in allogeneic setting and, additionally, empowers them with improved safety and efficacy attributes. Among others, new features include expression control properties, resistance to standard oncology treatments, and prevention of fratricide of engineered CAR T-cells.

**Conclusions**

We have successfully developed GMP-compliant manufacturing of TALEN®-edited CAR T-cells for clinical use, which has led to two allogeneic CAR T-cell product candidates in the clinic. Preliminary data show expansion of allogeneic cells associated with antitumor activity, providing first clinical proof of concept for allogeneic CAR T- cell approaches. This technology offers unparalleled possibilities to design next generation cell immunotherapies not only in hematological malignancies but also in solid tumors.

#### P215 Preclinical evaluation of Deep™ IL-15 Primed PMEL cells demonstrates highly improved safety compared to systemic administration of IL-15

##### Philip Bardwell, PhD, Elena Geretti, Xiaoyan Liang, Santina Caruso, De-Kuan Chang, PhD, Jesse Lyons, Carlos Tassa, Sanela Bilic, Janice Lanista, Becker Hewes, MD, Jonathan Fitzgerald, PhD, Thomas Andresen, PhD

###### Torque Therapeutics, Cambridge, MA, USA

####### **Correspondence:** Thomas Andresen (tandresen@torquetx.com)

**Background**

Interleukin-15 (IL-15) is a promising candidate for tumor immunotherapy, since it is a strong activator of both CD8+ T and NK cells and in contrast to IL-2 does not activate regulatory T cells. Systemic administration of IL15- Fc to patients resulted in dose-limiting toxicities, likely due to activation of NK cells. Here we describe the safety of T cells loaded with Deep™ IL-15, a multimer of reversibly crosslinked IL-15/IL-15 Rα/Fc subunits (IL15-Fc). Deep IL-15 acts as an autocrine source of IL15-Fc providing T cell activation, expansion, and promotion of memory phenotypes. Additionally, Deep IL-15 limits systemic exposure to IL15-Fc thus avoiding hyperproliferation of endogenous cells, including NK cells, the primary mediators of IL-15 immunotoxicity.

**Methods**

Deep IL-15 was evaluated in an adoptive T cell therapy model by treatment of B16-F10 tumor-bearing mice with PMEL CD8+ T (PMEL) cells (10-27 x 106), carrying up to 158 μg of Deep IL-15 (>15-fold the maximum tolerated dose, MTD, of IL15-Fc). The toxicity of Deep-15 PMEL was compared with PMEL (10 x 106) co-injected with IL15-Fc (MTD of 10 μg/mouse) (PMEL + IL15-Fc). Readouts included IL15-Fc exposure, cytokine release, changes in endogenous T cells and histopathology. Additionally, Deep™ IL-15 was administered to naïve C57BL/6 mice (10-100 μg/mouse) to evaluate direct effects of systemic Deep IL-15.

**Results**

Deep-15 Primed PMEL cells deliver significantly improved anti-tumor activity compared to PMEL cells alone in the B16-F10 model. Compared to systemic co-administration of IL15-Fc with PMEL, treatment with Deep-15 PMEL obtained a 300-fold lower systemic exposure to IL15-Fc, 41-fold lower IFN-γ release, and no expansion of endogenous cells (CBC; flow cytometry: CD4+, CD8+ and NK cells). Histopathology of lungs, liver and spleen revealed minimal findings with Deep-15 PMEL, less pronounced when compared to PMEL + IL15-Fc. Additionally, injection of Deep™ IL-15 alone resulted in no weight loss, no IFN-γ release and no changes in endogenous CD4+, CD8+ and NK cells.

**Conclusions**

Torque’s novel Deep Primed technology offers the advantage of loading Deep™ IL-15 prior to cell infusion in ACT, at doses unachievable with systemic injection of IL15-Fc, resulting in controlled, cell-specific activation. Systemic injection of Deep IL-15 up to 15-fold of the IL15-Fc MTD resulted in no toxicity findings. Deep-15 PMEL cells were well tolerated, did not expand endogenous cells and resulted in minimal histopathological changes. Deep IL-15 Primed multi-targeted human T cells, TRQ15-01, are expected to start clinical evaluation in hematologic and solid tumors in late 2018.

**Ethics Approval**

All personnel received training from the Torque’s Animal Care and Use Committee (IACUC). All animal procedures were in strict accordance with the National Institutes of Health Guide for the Care and Use of Laboratory Animals and were approved by the Torque Animal Care and Use Committee.

#### P216 Select metabolic and costimulatory bolt-on transgenes enhance chimeric receptor-bearing T cell activity against solid tumors

##### Luke Barron, PhD, Kathleen Whiteman, Madaline Gilbert, Tapasya Pai, Megan Snyder, BS, Michael Fray, Allison Nelson, Tyler Johnson, Kelsey Lakeman, John Shin, Ryan Boomer, Seth Ettenberg, Kathleen McGinness, Greg Motz

###### Unum Therapeutics, Cambridge, MA, USA

####### **Correspondence:** Greg Motz (Greg.Motz@unumrx.com)

**Background**

The immunosuppressive features within solid tumors may limit the success of engineered T cell therapies. We sought to overcome key challenges in solid tumors by co-expressing novel transgenes in T cells bearing either Antibody-Coupled T cell Receptors (ACTRs) or Chimeric Antigen Receptors (CARs). We evaluated >100 metabolic and costimulatory genes for their ability to improve chimeric receptor T cell function in vitro and in vivo.

**Methods**

We compared the activity of T cells expressing parental ACTR or CAR constructs to T cells expressing these same parental constructs in combination with novel “bolt-on” transgenes. Constructs were virally transduced into primary human T cells. In vitro screening assays were developed to mimic a solid tumor microenvironment using common immunosuppressive factors like PGE2, TGF-beta, adenosine, kynurenine, low glucose, and immune-suppressive cells. T cell function was determined in these assays by monitoring T cell proliferation, IL-2 production, or resistance to chronic stimulation. In vivo anti-tumor activity was assessed against solid tumor xenograft mouse models.

**Results**

In vitro screening identified multiple classes of bolt-on costimulatory and metabolic genes that enhanced function in solid tumor-relevant assays in vitro. These genes represented diverse classes of costimulatory gene families and metabolic pathways, including glycolysis, the Krebs cycle, amino acid synthesis, and lactate pathways. Hits from in vitro screening were evaluated in tumor xenograft mouse models. Metabolism and costimulation bolt-on variants significantly improved activity over their respective chimeric receptor parents in both sensitive tumor models, where the parental construct is active and reduces tumor growth, and resistant tumor models where the parental construct is inactive. For example, the glucose transporter GLUT1 enabled bolt-on, but not parental, chimeric receptor- expressing T cells to proliferate in vitro despite limited glucose and cause regression of multiple solid tumor xenografts (Figure 1).

**Conclusions**

We have generated a panel of bolt-on transgenes to address challenges for T cells in the solid tumor microenvironment and evaluated a select set of these bolt-ons for their ability to enhance chimeric receptor T cell function, independent of parental chimeric receptor design. Variants were screened in assays established to mimic immunosuppressive aspects of solid tumors and in xenograft mouse models. We have shown that specific metabolism and costimulation bolt-on transgenes impart improved function to both ACTR- and CAR-expressing T cells relative to the parent chimeric receptor. Our results demonstrate a screening strategy to identify bolt-on transgenes that can overcome immunosuppressive challenges faced by T cell therapies in solid tumors.

**Consent**

This study was approved by Unum Therapeutics’ Institutional Animal Care and Use Committee (IACUC), approval number 2016-04-004.

**Fig. 1 (abstract P216).**
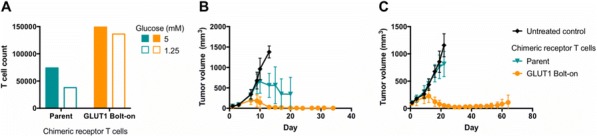
See text for description.

#### P217 Small molecule control of inducible MyD88/CD40 regulates Natural Killer cell expansion and anti-tumor activity

##### Joseph Bayle, Xiaomei Wang, PhD, Wei-Chun Chang, PhD, Daniel Jasinski, PhD, Jan Medina, Aaron Foster, PhD, David Spencer, PhD

###### Bellicum Pharmaceuticals, Houston, TX, USA

####### **Correspondence:** David Spencer (dspencer@bellicum.com)

**Background**

The intrinsic anti-tumor activity of Natural Killer (NK) lymphocytes has the potential to be used as an allogeneic cell therapy with reduced GvHD risk relative to αβ T cells, however its potential has been limited NK by poor expansion in vivo. Previously, we developed a Chimeric Antigen Receptor-T cell (CAR-T) strategy that uses rimiducid-based dimerization of inducible MyD88/CD40 (iMC) to control T cell expansion and survival [1]. Here we demonstrate that iMC can also be applied to NK cell growth and anti-tumor efficacy in vitro and in vivo. Furthermore, an orthogonally regulated switch, rapamycin-inducible Caspase-9 (iRC9), was used to provide safety.

**Methods**

Human donor-derived PBMCs were selected for CD56 positivity, activated with IL-15 and K562 feeder cells, and transduced with γ-retrovirus encoding control iRC9-2A-ΔCD19, iRC9-2A-ΔCD19-2A-iMC (dual-switch NK) or iRC9-2A-IL-15-2A-ΔCD19-2A-iMC (dual-switch/IL-15 NK).

**Results**

In cell growth assays iMC-expressing dual-switch NK cells selectively outgrew iRC9-only NK cells, and this effect was further stimulated by 1 nM rimiducid (Figure 1). Inflammatory cytokine and chemokine production was also dramatically (10 to 1000-fold) elevated by the expression and activation of iMC in NK cells. In cocultures with THP1 acute myeloid leukemia cells at increasing Target:Effector (T:E) ratios, presence (P < 0.001, two way ANOVA) and activation (P <0.001) of iMC dramatically increased tumor killing activity. Dual-switch NK anti- tumor activity was determined in xenografts of immunodeficient NSG mice bearing THP1 tumors (Fig 1). Unstimulated iMC with IL-15 or activation of iMC without IL-15 expression supported modest NK cell expansion, but rimiducid stimulation of iMC plus autocrine IL-15 showed enhanced NK expansion in vivo. Moreover, in tumor-free animals only dual-switch/IL-15 NK cells with weekly rimiducid stimulation expanded and persisted in vivo (up to 7 weeks). Cotransduction of a first generation CD123-targeted CAR to produce dual-switch/IL-15 CD123CAR-NK cells led to rimiducid-dependent control of THP1 tumor outgrowth in vivo beyond 40 days. Conversely, temsirolimus-mediated activation of the iRC9 safety switch rapidly (< 24 hours) ablated dual-switch NK cells in vivo.

**Conclusions**

Inducible MyD88/CD40 is an activation switch that supports NK cell expansion, persistence and anti-tumor activity in vitro and in vivo when paired with autocrine IL-15 expression. Tumoricidal activity can be further activated by target-specific CAR expression with safety controlled by the orthogonal iRC9 switch. This novel, regulated NK cell platform solves several of the challenges of NK cell-based therapy and should be readily translatable as an off-the- shelf cellular therapy for malignancies.

**References**

1. Foster, et al., Regulated expansion and survival of Chimeric Antigen Receptor-modified T cells using small molecule-dependent inducible MyD88/CD40. Molecular Therapy. 2017; 25: 2176-2188.

**Fig. 1 (abstract P217).**
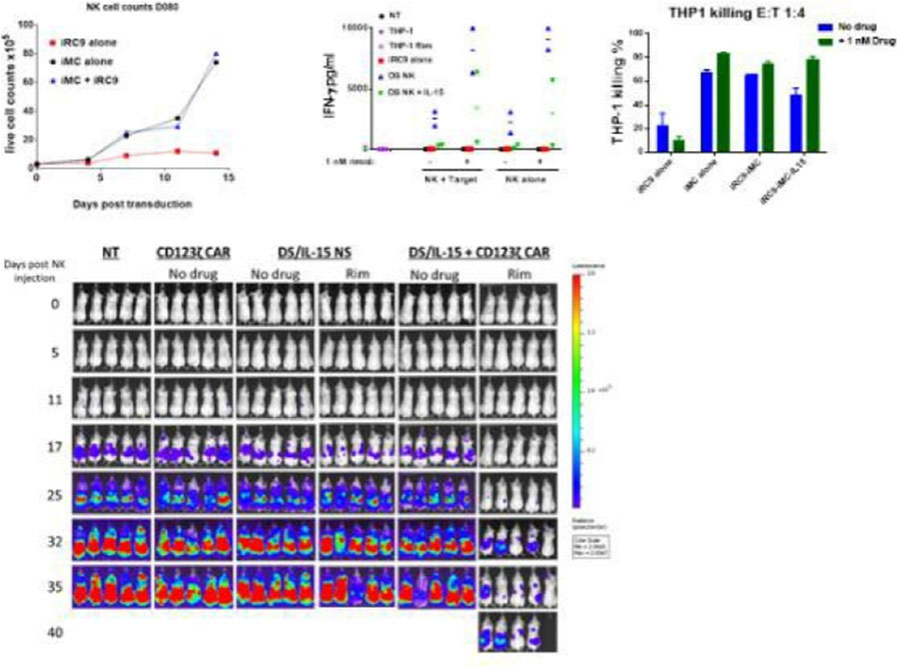
See text for description.

#### P218 Endogenous DAP10 provides optimal co-stimulation to NKG2D-based CAR T cells

##### Jennifer Bolsée, Eytan Breman, MSc, Thuy Nguyen, Sophie AGAUGUE, PhD, David Gilham, PhD

###### Celyad, Mont-Saint-Guibert, Belgium

####### **Correspondence:** Jennifer Bolsée (jbolsee@celyad.com)

**Background**

Chimeric antigen receptor (CAR) proteins are artificial proteins created by the fusion of an extracellular domain targeting one or several cell surface antigens, a hinge, a transmembrane domain and an intracellular part responsible for signal initiation and transmission to activate T cells. This part is usually composed of the intracellular domain of CD3ζ and one or two co-stimulatory domains (e.g. the cytoplasmic domain of CD28 and/or 4-1BB) leading to second and third generation CARs respectively. We developed a NKG2D-based CAR composed of the full length human NKG2D and the intracellular part of CD3ζ. NKG2D is a receptor expressed on NK cells and some T cell subsets, existing as a dimer that interacts with a co-adaptor protein called DAP10. Upon interaction of NKG2D with one of its ligands, the DAP10 cytoplasmic tail induces downstream signaling and provides co-stimulation. Thus, the NKG2D-based CAR T cell (termed CYAD-01) despite appearing as a first-generation CAR, functions as a second generation. Here, we investigated whether CYAD-01 efficacy could be enhanced by the addition of costimulatory domains or overexpression of DAP10.

**Methods**

In this study, different NKG2D-based constructs were created and compared side by side to our current construct.

Two different modifications of CYAD-01 CAR T cells were performed: DAP10 overexpression and addition of CD28 or 4-1BB co-stimulatory domains to generate “classical” CAR designs.

**Results**

Co-expression of DAP10 increased CAR expression at the surface of T cells without increasing IFN-γ secretion or cytolytic activity upon co-culture with target cells. Interestingly, addition of a co-stimulatory domain significantly decreased CAR expression, though that was not accompanied by any reduction in IFN-γ secretion or cytolytic activity when T cells were cultured with cancer cells. Further characterization of the cytokine secretion profile of the distinct CAR T cells by Luminex showed that no difference was observed in the cytokine secretion pattern in response to tumor cell lines or recombinant NKG2D ligands.

**Conclusions**

Our results uncover that the co-signaling delivered by the endogenous DAP10 is optimal for the in vitro NKG2D CAR T function, and that this signaling was as potent as traditional CD28 or 4-1BB based co-stimulation.

Overexpression of DAP10 did not further affect CAR T cell function. These results pinpoint that CAR design is likely to be optimal for the generation of effective NKG2D-based CAR T cells. In vivo studies are ongoing to assess whether anti-tumor efficacy and persistence could be affected by overexpression of DAP10 or addition of co- stimulatory domains.

#### P219 SQZ’ing cells to engineer next generation antigen presenting cell (APC) therapies

##### Matthew Booty, PhD, Scott Loughhead, Kelan Hlavaty, Ildefonso Vicente-Suarez, Katarina Blagovic, PhD, Melissa Myint, Brittany Stokes, MS, Defne Yarar, Howard Bernstein, MD, PhD, Armon Sharei

###### SQZ Biotechnologies, Watertown, MA, USA

####### **Correspondence:** Matthew Booty (matt.booty@sqzbiotech.com)

**Background**

The presentation of sufficient antigen on major histocompatibility complex class I (MHC-I) is a potential barrier to generating potent cancer immunization strategies. In this work, we use microfluidics-based Cell Squeeze® technology to deliver antigen directly to the cytosol of target APCs – resulting in the enhanced presentation of antigen on MHC-I. In addition to facilitating potent CD8+ T cell priming by professional APCs, this approach can make T cells effective, unorthodox APCs capable of priming CD8+ T cell responses in murine and human systems.

**Methods**

Protein and peptide antigens were delivered to the cytosol of purified murine or human T cells with Cell Squeeze®. The response to in vivo immunization was assessed by flow cytometry in a series of experiments using wild type C57BL/6 mice, MHC-I knockout mice, and/or adoptively transferred transgenic OT-I CD8+ T cells. Tumor experiments were conducted with the TC-1 cell line, which expresses the viral antigens E6 and E7 from human papilloma virus type 16 (HPV16).Human T cells were loaded with synthetic long peptides containing antigens from cytomegalovirus (CMV) or HPV16. These T cells were co-cultured with epitope-reactive human responder CD8+ T cells, and interferon gamma production was quantified to assess antigen-specific responses in vitro.

**Results**

In murine systems, we demonstrate that Cell Squeeze® delivers protein antigen to T cells effectively and that the immunogenic epitope is processed and presented on MHC-I. When adoptively transferred in vivo, T cells squeezed with ovalbumin drive the expansion of CD8+ T cells in both transgenic (OT-1) and endogenous response assays. In a tumor model for HPV associated cancers, TC-1, E7 loaded T cells have strong anti-tumor effects both prophylactically and therapeutically. Following therapeutic immunization, the anti-tumor responses correlate with an increase in antigen-specific CD8+ tumor infiltrating lymphocytes compared to untreated mice. In human cells, we demonstrate that primary donor-derived unstimulated T cells can be effectively loaded with CMV and HPV16 antigens using Cell Squeeze®. These T cells are capable of stimulating antigen-specific CD8+ T cell responses in vitro using both T cell clones and patient-derived memory populations. The SQZ process has been scaled to engineer >1 billion T cells per minute in preparation for clinical translation.

**Conclusions**

Through the direct cytosolic delivery of antigen, we have engineered T cells to function as potent APCs. This strategy has demonstrated significant potential to generate CD8+ T cell responses in both murine and human systems and has been scaled up for clinical implementation.

**Ethics Approval**

Human samples were supplied by an approved vendor and animal studies were conducted in accordance with SQZ Biotech's Animal Care Program and IACUC which operate according to principles set forth in the PHS Policy, the Guide for the Care and Use of Laboratory Animals - 8th edition.

#### P220 Generating allogeneic CAR T cells without gene editing

##### Simon Bornschein, PhD^1^, Alexandre Michaux, PhD^1^, Susanna Raitano, PhD^1^, Eytan Breman, MSc^1^, Céline Jacques-Hespel^1^, Fanny Huberty^1^, Laura Saerens^1^, Dorothée Daro^1^, Steven Lenger, PhD^2^, Hidevaldo Machado, PhD^2^, Jonathan Moore, PhD^2^, David Gilham, PhD^1^

###### ^1^Celyad S.A., MONT-SAINT-GUIBERT, Belgium; ^2^Horizon Discovery, Lafayette, CO, USA

####### **Correspondence:** Simon Bornschein (sbornschein@celyad.com)

**Background**

Current licensed CAR T products are an autologous therapy requiring collection, manufacture and formulation of the patients own T cells. Whilst clearly effective, there remain timely challenges to generate the cells (vein to vein) as well as a variability in patient’s cells, resulting in manufacturing failure and product inconsistency.Allogeneic, off-the-shelf CAR T cell therapy is attractive since the cells are generated from a single healthy donor, thus maintaining consistency, reducing vein to vein time and allowing for treatment of multiple patients from the same manufactured batch. However, the main limitation of allogeneic therapy is the recognition of patient Human Leukocyte Antigen by the donor T cell receptor (TCR) driving Graft-versus-Host Disease (GvHD). Gene editing to eliminate the TCR is currently the most used approach in the allogenic CAR T cell field. Whilst efficient, there are potential limitations to gene-editing. Consequently, we have explored non-gene edited based technologies for their applicability in the allogeneic CAR T cell field.

**Methods**

To disrupt functionality of the TCR complex, we engineered a TCR inhibitory peptide, TIM, consisting of a truncated, signaling incompetent, form of CD3z. In parallel, several shRNAs were tested for their ability to reduce TCR expression by means of targeting the CD3 complex in primary T cells. The best shRNA candidates were also combined with TIM and all these types of engineered T cells were then tested in vitro for response against mitogenic antibody stimulation (OKT3) and in vivo for control of xenoGvHD in the NSG mouse strain.

**Results**

shRNA specific for CD3e and CD3z reduced expression of their relevant targets but also significantly reduced cell surface expression of the TCR by contrast to TIM. Interestingly, combining TIM with shRNA led to a recovery in TCR expression. Preliminary results indicate that T cells, engineered with non-genome editing technologies showed reduced in vitro mitogenic response when challenged with anti-CD3 antibodies. In vivo experiments also indicated an inhibition of GvHD with all those technologies when the T cells were adoptively transferred to the NSG mouse. The potency of the combination of TIM and shRNA and the comparison with gene edited knock-out of the TCR will be presented during the conference.

**Conclusions**

These experiments demonstrate that non-gene editing approaches to the generation of allogeneic CAR T cells using single vector approaches are feasible and may offer an attractive alternative to gene editing.

**Ethics Approval**

The study was approved by local authorities and the Ethics Board.

#### P221 The co-expression of a single shRNA targeting MICA and MICB with a NKG2D-CAR (CYAD-01) generates CAR-T cells resistant to target driven fratricide and improves CYAD-01 cell persistence in vivo

##### Simon Bornschein, PhD^1^, Susanna Raitano, PhD^1^, Jérôme Marijsse, master^1^, Dorothée Daro^1^, Steven Lenger, PhD^2^, Hidevaldo Machado, PhD^2^, Jonathan Moore, PhD^2^, Sophie AGAUGUE, PhD^1^, David Gilham, PhD^1^

###### ^1^Celyad S.A., MONT-SAINT-GUIBERT, Belgium; ^2^Horizon Discovery, Lafayette, CO, USA

####### **Correspondence:** Simon Bornschein (sbornschein@celyad.com)

**Background**

NKG2D is an activating receptor most commonly expressed on NK cells and subsets of T cells. NKG2D is known to engage 8 different stress induced ligands (NKG2DL) broadly present on tumors but largely absent on healthy tissue. T cells bearing a CAR consisting of the full human NKG2D receptor fused to the intracellular domain of CD3z (CYAD-01 T cells) specifically recognize and kill cancer cell lines derived from hematological and solid tumors in vitro and in vivo. These data have supported translation into phase 1 clinical trials. However, while working through the dose levels of these trials, it has become clear that the ability to manufacture large numbers of CYAD-01 T cells is problematic. The underlying reason appears to be self-killing (fratricide) mediated by the transient expression of NKG2DL on activated T cells. Given the potential breadth of ligand binding, our efforts have focused upon identifying the main NKG2DL that are expressed on activated T cells and to develop a translationally relevant strategy to modulate this expression. We then questioned whether this strategy enabled CYAD-01 T cells to avoid fratricide and thereby achieve an improved in vivo persistence.

**Methods**

Molecular and cellular analyses identified the key NKG2DL on activated T cells. We then explored specific targeting of the ligands through the co-expression of shRNA within the CAR vector to determine the subsequent impact upon in vitro and in vivo function of T cells bearing the NKG2D CAR.

**Results**

Of the eight NKG2DL, MICA and MICB were transiently expressed on activated T cells while ULBP1 was restricted to CD8+ T cells. There was little evidence of expression of the other ligands on T cells. Parallel studies having identified MICA and MICB as major stimulators of the NKG2D CAR, we screened shRNA to target these ligands. Two shRNA were identified that reduced cell surface expression of MICA/MICB. Engineering of a single vector encoding the NKG2D CAR and shRNA generated T cells that had much reduced in vitro fratricide, maintained NKG2D specific effector function and improved engraftment in NSG mice. The anti-tumor efficacy of these cells is currently under investigation and will be reported during the conference.

**Conclusions**

A single vector encoding the NKG2D CAR with a single shRNA generates CYAD-01 T cells that are resistant to CAR-T driven fratricide. We continue to explore the characteristics of these CAR T cells with the intention of translating to a clinical trial during 2019.

**Ethics Approval**

The study was approved by local authorities and the Ethics Board.

#### P222 Genetic engineering of tumour infiltrating lymphocytes (TIL) with a novel recombinant growth factor receptor for treatment of solid tumours

##### John Bridgeman, BSc, MSc, PhD^1^, Michelle Le Brocq^1^, Joanne McCaffrey^1^, Tania Katopodi, PhD^1^, Gemma Owens, MB BCh, BSc, MRes^2^, Aysha Patel^1^, Ryan Guest^1^, Robert Hawkins^1^

###### ^1^Immetacyte Ltd, Manchester, UK; ^2^University of Manchester

####### **Correspondence:** Robert Hawkins (r.hawkins@cellulartherapeutics.co.uk)

**Background**

Lymphocytes isolated from tumour biopsies can be expanded ex vivo to huge numbers before readministration to patients whereupon theyimpart therapeutic benefit. So called Tumour infiltrating lymphocyte (TIL) therapy has proved hugely successful in numerous clinical trials for treatment of metastatic melanoma, and have also shown benefit in other indications. Persistence of therapeutic T-cells post infusion remains an issue which can be improved. In most situations preconditioning chemotherapy allows successful engraftment, and post-infusion IL-2 further potentiates the engraftment and persistence of T-cell infusions. However both treatments are associated with potentially severe toxicity and significantly increases the cost of treatment.

**Methods**

We have investigated the use of recombinant growth factor receptors to increase growth and survival of TIL.

Recombinant growth factor receptors (rGFR) based on the thrombopoietin receptor (TpoR) were delivered to primary T-cells or TIL using lentiviral gene transfer. These engineered cells were then cultured with IL-2 or the agonist mimetic drug Eltrombopag. In some situations the engineered cells were coculcured with continuous patient matched Melanoma or Ovarian tumour lines which were generated from dissociated tumour.

**Results**

Administration of the TpoR agonist mimetic drug Eltrombopag resulted in enrichment of engineered cells when cultured alone, furthermore, we observed enhanced growth and survival of engineered cells in an in vitro model of tumour targeting of melanoma and ovarian cancer against matched autologous tumour lines. We have further developed the basic receptor format to derive an optimised receptor with enhanced activity. We have also developed a method to deliver these rGFRs to TIL at high efficiency and in a GMP complient manner which will expedite their translation into a manufacturing process for clinical trial.

**Conclusions**

We have developed a technology based on the use of recombinant growth factor receptors which allows us to preferentially select engineered cells using a clinically available drug. We have demonstrated this technology in vitro in the setting of melanoma and ovarian cancer thus paving the way for use of this engineering approach in gene engineered TIL trials.

**Ethics Approval**

This study was approved by the UK Research Ethics Committee: Approval Number 169632

#### P223 In vitro analysis of Tumour Infiltrating Lymphocytes engineered with costimulatory antigen receptors delivering targeted costimulation

##### Tania Katopodi, PhD, John Bridgeman, BSc, MSc, PhD, Michelle Le Brocq, Aysha Patel, Martina Sykorova, Joanne McCaffrey, Ryan Guest, Robert Hawkins

###### Immetacyte Ltd, Manchester, UK

####### **Correspondence:** Robert Hawkins (r.hawkins@cellulartherapeutics.co.uk)

**Background**

Tumour infiltrating lymphocyte (TIL) therapy involves the ex vivo culture and expansion of lymphocytes – in particular T-cells – obtained from tumour biopsies, before readministration into the same patient. TIL therapy has proved effective in the amelioration of metastatic melanoma which has failed other standard treatment options, furthermore, trials in other cancer indications have shown encouraging results. As in other adoptive cell therapy settings, the anti-tumour response may, in certain circumstances, be inefficient, leading to tumour escape. This anti- tumour response may be subverted by the lack of adequate support signals delivered to the anti-tumour T-cells, for example, tumour cells rarely express any ligands for costimulatory receptors which are essential for full and sustained T-cell activation and cytokine secretion, the latter important for successful T-cell engraftment post infusion.

**Methods**

We have therefore sought to investigate the use of targeted costimulation, to this end T-cells were engineered using lentiviral vectors to express a chimeric costimulatory antigen receptor (CoStAR) which provides costimulation in response to defined tumour associated antigens. We have evaluated a number of target antigens across a spectrum of malignancies and have optimised assays using CoStARs targeting CEA and CA-125.

**Results**

Here we demonstrate that CoStARs targeting CEA (Colorectal, Gastric, Oesophageal) and CA-125 (Ovarian) enhance cytokine production upon engagement with their cognate antigen when signal 1 is delivered through the TCR. We have investigated a number of different CoStAR iterations with distinct signalling capacities and have optimised an engineering strategy which enables efficient delivery of CoStAR to TIL in a GMP compliant manner.

**Conclusions**

Targeted costimulation offers a means to enhance the activity, survival and proliferation of TIL. Our strategy and efficient gene transfer technologies paves the way for clinical trial of CoStAR in a spectrum of cancer indications.

**Ethics Approval**

The study was approved by the UK Research Ethics Committee - approval number 169632

#### P224 Adoptive cell therapy using tumor infiltrating lymphocytes for the treatment of bladder cancer

##### Brittany Bunch, PhD, Matthew Beatty, PhD, Jennifer Morse, MS, Michael Kidd, MacLean Hall, Autumn Joerger, Charles Peyton, Mayer Fishman, MD, PhD, Amod Sarnaik, MD, Michael Poch, Shari Pilon-Thomas, PhD

###### H. Lee Moffitt Cancer Center, TAMPA, FL, USA

####### **Correspondence:** Shari Pilon-Thomas (shari.pilon-thomas@moffitt.org)

**Background**

Patients with advanced bladder cancer have limited therapeutic options and a median overall survival between 12 and 15 months. Apart from the recent FDA approval of checkpoint inhibitors for patients with metastatic disease, therapy options have not changed in decades. Adoptive cell therapy (ACT) using tumor infiltrating lymphocytes (TIL) has improved the median overall survival in patients with metastatic melanoma to 52 months. Our goal is to study ACT of TIL in bladder cancer using both human tumor specimens and in vivo studies.

**Methods**

TIL was expanded from resected lymph node metastasis or primary bladder tumors after radical cystectomy. Tumors were minced into fragments and plated in high dose IL-2 alone or in combination with agonistic 4-1BB. Reactivity was tested by co-culture with autologous tumor and IFN-gamma ELISAs. In vivo, mice were inoculated with syngeneic MB49 bladder tumor cells expressing OVA. Murine TIL was isolated from subcutaneous tumors and tested for reactivity by co-culture and IFN-gamma ELISAs. In mice bearing orthotopic MB49-OVA tumors, OVA- specific T cells were delivered intravesically. Tumor volume was monitored via ultrasound.

**Results**

We were able to expand TIL from 32 out of 39 primary and metastatic patient samples. Reactive TIL was detected in 19 out of 30 samples (63%, 2 samples did not have autologous tumor for testing). The addition of 4-1BB improved TIL expansion from primary tumors. In vivo, murine TIL isolated from subcutaneous tumors were reactive against tumor digest and MB49-OVA cell lines, but not against irrelevant tumor. Finally, we have determined that T cells can infiltrate into bladder tumors within 3 hours after intravesical delivery. Intravesical T cell delivery resulted in a decrease tumor burden compared with PBS treated mice.

**Conclusions**

From these studies we have demonstrated the feasibility of expanding reactive TIL from bladder cancer specimens. In addition, we have developed an in vivo murine model to study TIL therapy for the treatment of bladder cancer. Further studies will compare the response between systemic and intravesical delivery of TIL in vivo and characterize immune cell infiltrates within the tumor microenvironment.

**Ethics Approval**

These studies were approved by Moffitt Cancer Center's Ethics Board and USFs Institutional Animal Care and Use Committee .

#### P225 Adoptive cell transfer with NY-ESO-1 specific TCR T cells (TBI-1301) results in persistence, cytokine release syndrome and anti-tumor activity

##### Marcus Butler, MD^1^, Valentin Sotov^1^, Megan Nelles^1^, Sarah Boross-Harmer^1^, Luisa Bonilla^1^, Sawako Elston^1^, Michael Fyrsta^1^, Diana Gray, MSc^1^, Abha Gupta^1^, Sevan Hakgor^1^, Ausmeema Hossain^1^, Michael Le^1^, Darya Lemiashkova^1^, Diane Liu^1^, Charlotte Lo^1^, Mark Comacho^1^, Aaron Hansen^1^, David Hogg, MD, FRCPC^1^, Habeeb Majeed^1^, Kiichi Murakami^1^, Jessica Nie^1^, Marc Ouellette^1^, Albiruni Razak^1^, Kendra Ross^1^, Adrian Sacher, MD^1^, Sam Saibil^1^, Elizabeth Scheid^1^, Anna Spreafico, MD PhD^1^, Brendan Van As^1^, Jennifer Yam^1^, Pamela Ohashi, PhD^1^, Shuichi Takahashi^2^, Shinya Tanaka^2^, Linh Nguyen, PhD^1^

###### ^1^Princess Margaret Cancer Centre, Toronto, ON, Canada; ^2^Takara Bio, Inc, Kasatsu, Japan

####### **Correspondence:** Marcus Butler (marcus.butler@uhn.ca)

**Background**

TBI-1301 is a novel cell therapy product produced by engineering autologous lymphocytes to express an affinity-enhanced NY-ESO-1-specific TCR using a proprietary retrovirus vector that encodes siRNA to silence endogenous TCR expression. Previously, Kageyama et al. reported that the adoptive transfer of TBI-1301 could result in anti- cancer responses and cytokine release syndrome (CRS) in patients (ASH Annual Meeting, 2017). In parallel to this Japanese study, we are conducting a single site phase Ib study at the Princess Margaret Cancer Centre where patients with NY-ESO-1-expressing solid tumors are infused with NYESO-1 specific T cells (TBI-1301).

**Methods**

Eligibility for study participation includes informed consent, HLA-A*02:01 or A*02:06 haplotype, NY-ESO-1 expression confirmed by immunohistochemistry, disease progression, and lack of curative standard therapy. Eligible patients undergo phlebotomy to harvest PBMC which are then processed locally using the same standard operating procedures and reagents as used for the Kageyama study to generate engineered TBI-1301 cells. The study design is to manufacture and infuse 5x10^9 cells (day 0) to 9 patients following modest lymphodepletion with cyclophosphamide (750 mg/m2 on day -3 and -2). Endpoints include safety assessment, evaluation of efficacy, and biological correlates for persistence of NY-ESO-1 T cells post infusion.

**Results**

Thus far, 6 patients (1 endometrial cancer, 3 synovial sarcoma, 2 melanoma) have been enrolled and undergone study treatment. An additional 3 patients are undergoing manufacture (2 synovial sarcoma and 1 ovarian cancer). 5/6 treated patients received 5x10^9 cells while 1 patient with synovial sarcoma received 2.1x10^9 cells due to lower dose of manufactured cells. To date, no DLTs have been observed. Despite the lack of fludarabine in the lymphodepletion regimen, all 3 patients with synovial sarcoma experienced clinical and laboratory evidence of grade 1 CRS with increased CRP, ferritin, and IL-6 levels. CRS resolved spontaneously in 2 patients while the third received tocilizumab with rapid resolution of fevers. In this patient, grade 3 pain at his tumor site prolonged his admission. Thus far, 2 confirmed partial responses by RECIST have been observed. Moreover, biomarker analysis demonstrated transient reduction over 3 weeks in Tregs (CD4+CD25+CD127lowFoxp3+) and persistence of NY- ESO-1 specific T cells for greater than 100 days post infusion. Interestingly, persisting NY-ESO-1 T cells expressed CD27, as well as PD-1 and TIGIT.

**Conclusions**

TBI-1301 appears to be safe and to possess anti-tumor activity. Ongoing biomarker analysis will allow for further development of this technology and potential development of combination clinical trials.

**Trial Registration**

NCT02869217

**Ethics Approval**

The study was approved by the University Health Network Ethics Board, approval number 15-9534.

#### P226 *DeepTM IL-15 primed* multi-targeted T cells demonstrate potent antigen-specific cytotoxic activity against human cancer cells

##### Shawn Carey, PhD, Beth Pearce, Darren Smith, Pengpeng Cao, PhD, Christine McInnis, PhD, Amy Shaw, Jonas Bruun, PhD, FABIO FACHIN, PhD, Becker Hewes, MD, Jonathan Fitzgerald, PhD, Thomas Andresen, PhD, Andy Rakestraw, PhD

###### Torque Therapeutics, Cambridge, MA, USA

####### **Correspondence:** Thomas Andresen (tandresen@torquetx.com)

**Background**

Adoptive transfer of tumor-directed T cells has demonstrated encouraging clinical efficacy in some hematological and solid tumors. However, widespread success of such therapies has been limited by (1) single-epitope targeting by genetically modified TCR and CAR T cell therapies and (2) insufficient support of transferred T cell survival and function. To direct immune activation in the tumor microenvironment, Torque has developed the *Deep-PrimedTM* T cell therapy platform in which cytotoxic T lymphocytes (CTLs) simultaneously targeting multiple tumor associated antigens (TAA) are primed with immune-stimulatory drugs tethered to their surface to provide localized and sustained support to tumor-directed CTLs. This study evaluates cancer cell-directed cytotoxicity by multi-target CTLs with and without *DeepTM IL-15*, which is a cell-associated crosslinked multimer of human IL15-Fc.

**Methods**

CTLs directed against cancer cells expressing TAA including MART-1 or PRAME were generated from healthy donors using Torque's modular TAA-priming approach. CTL-mediated cytotoxicity was assessed using a panel of partially HLA-matched human cancer cells with diverse TAA expression. Cytotoxic activity was compared with and without Deep IL-15, and kinetic analysis of CTL function including expansion, activation, and cytotoxicity was used to provide mechanistic understanding of Deep IL-15 impact on anti-cancer cell activity by CTLs.

**Results**

Using time course mixed culture models, we show that Deep IL-15 enhances the performance of tumor-directed CTLs. MART-1-targeted and PRAME-targeted CTLs elicit specific cytotoxicity against human cancer cells expressing their respective antigen target, and Deep IL-15 augments this activity. Multiplexed analysis of CTL function revealed that this Deep IL-15-driven improvement in cytotoxicity is accompanied by IL-15-mediated CTL support both pre- and post-exposure to cognate antigen. First, Deep IL-15 provides autocrine signaling that drives survival and expansion of antigen-specific CTLs prior to antigen exposure, enabling a more robust CTL response against TAA-positive cancer cells upon encounter. Additionally, once Deep IL-15 primed CTLs are exposed to antigen, they show prolonged antigen-specific activation, survival, and expansion, as well as persistent antigen- specific cytotoxicity, indicating the Deep IL-15 improves the durability of anti-tumor activity.

**Conclusions**

Tumor-directed CTLs generated using Torque's modular TAA-priming approach elicit potent cytotoxicity against cancer cells expressing multiple TAA including MART-1 and PRAME. By acting as a cell-tethered cytokine source of bioactive IL-15, Deep IL-15 enhances CTL survival, proliferation, function, and cytotoxicity. Clinical trials evaluating TRQ15-01, Torque's Deep IL-15 Primed multi-targeted T cells, will initiate later this year.

#### P227 Tethering IL-12 to the surface of T cells induces a broad immune activation and potent anti-tumor activity in mice without inducing systemic toxicities

##### De-Kuan Chang, PhD, Gulzar Ahmad, Jonathan Nardozzi, PhD, Douglas Jones, PhD, Thomas Andresen, PhD

###### Torque Therapeutics, Cambridge, MA, USA

####### **Correspondence:** Thomas Andresen (tandresen@torquetx.com)

**Background**

T cell-based immunotherapy has shown dramatic efficacy in some hematologic malignancies but translating thes successes to solid tumors has been limited. A key challenge has been overcoming the immunosuppressive tumor microenvironment, which inhibits T cell activity and survival. Interleukin-12 (IL-12) holds strong potential for reshaping the anti-inflammatory environment in solid tumors. Its clinical utility, however, has been limited by severe toxicities both from systemic administration and from genetic engineering of tumor-specific T cells. To overcome these obstacles, we developed the Deep Primed< sup >TM< /sup > platform to anchor potent immune modulators on T cells to support immune activation in the tumor microenvironment. Our approach is versatile and enables tunable loading and persistence of IL-12 on the T cell surface.

**Methods**

Safety and efficacy profile of an engineered Deep IL-12 cytokine was evaluated in an immune-competent adoptive cell therapy model. Briefly, we utilized CD8 T cells from PMEL mice, which contain a T cell receptor specific to the gp100 antigen expressed in B16-F10 melanoma cells. Deep IL-12 was loaded ex vivo onto PMEL T cells and adoptively transferred into C57BL/6J mice bearing B16-F10 tumors. Here we present tumor growth inhibition, cytokine secretion, toxicity biomarkers, blood and tissue chemistry, and immune cell activity from adoptive transfer of tumor-specific T cells primed with Deep IL-12.

**Results**

Deep IL-12 significantly improved anti-tumor efficacy of adoptively transferred PMEL T cells against established B16-F10 tumors as compared with cell therapy alone or systemic co-administration of IL-12. Deep IL-12 increased peak expansion and long-term engraftment of PMEL T cells, without inducing expansion of circulating NK cells, which are believed to be a key mediator of IL-12 toxicity. There were no observed overt toxicities despite a modest, transient increase in circulating IFNg. Doses of Deep IL-12 more than 100-fold above the efficacious dose level similarly induced modest, transient circulating cytokine levels and did not induce overt toxicities in the form of body weight loss, suggesting a strong therapeutic window for Deep IL-12. Multiple doses of PMEL T cells loaded with Deep IL-12 further improved anti-tumor activity, in contrast to multiple doses of PMEL T cells in the absence of the Deep IL-12, which provided minimal anti-tumor activity.

**Conclusions**

Our data demonstrates that tethering IL-12 to the immune cell surface using Deep IL-12 dramatically improves the efficacy of tumor-targeted cell therapy, while mitigating toxicities associated with systemic IL-12.

#### P228 Novel electroporation method for T cells enables quick CAR-T cell manufacture

##### Jian Chen, PhD, Xiaofeng Xia, PhD

###### Celetrix LLC, Manassas, VA, USA

####### **Correspondence:** Jian Chen (jchen@celetrix.com)

**Background**

CAR-T cells are currently manufactured for clinical use by infection of human T cells with viral vectors containing the CAR gene. T lymphocytes have to be stimulated and expanded ex vivo because the viral vectors infect fresh natural lymphocytes very poorly. The viral vector approach is extremely expensive due to the high cost of virus production and the high cost of long-term cell expansion that could take 10-14 days in a GMP facility. The viral vector approach also has a huge biological downside: ex vivo expanded T cells become bulky and lose efficacy against tumor cells. The use of electroporation technology in CAR-T cell manufacturing has attracted increasing interests for its low cost and the wide range of application including transposon based stable CAR expression, transient expression and genome editing. However, actual clinical use of electroporation technology in CAR-T has been difficult and several clinical trials have met significant problems due to the poor transfection efficiency and decreased T cell survival with traditional electroporation methods.

**Methods**

Through theoretical analysis of the other existing electroporation technologies, we found that they all have problems in electrophysical design. The problems include physical design of devices, electrical pulse selection, and buffer composition. The common electroporation cuvettes were changed to sealed cylindrical electroporation tubes. The buffer was also redesigned to support high efficiency and non-toxic electroporation. Specific protocols for PBMC were generated for the new electroporation system.

**Results**

With the new electroporation system, we can now achieve very high transfection efficiency for T cells while maintaining cell survival. For Sleeping Beauty transposon-based CAR expression, we found that over a period of two to three weeks the efficiency can get to 60% to 90% with fast cell proliferation. The protein expression time after electroporation is very short. For simple GFP plasmids we can observe GFP expression after only 30 minutes. Compared to other electroporation systems such as Amaxa Nucleofector, the new system produces at least two-fold increase in a combined efficiency/viability score.

**Conclusions**

Unlike viral vectors, electroporation works well on fresh natural T cells, thereby eliminating the need for expensive cell expansion and virus production altogether and cutting the huge economic burden of CAR-T therapy. By re- infusion of more natural T cells, the anti-tumor efficacy of CAR-T cells could be improved while the side effects of cytokine release syndrome could be minimized.

#### P229 Adapter mediated transduction with lentiviral vectors: A novel tool for cell-type specific gene transfer

##### Nicole Cordes, Master of Science, Carolin Kolbe, Thomas Schaser, Andrew Kaiser

###### Miltenyi Biotec, Bergisch -Gladbach, Germany

####### **Correspondence:** Thomas Schaser (thomasscha@miltenyibiotec.de)

**Background**

Efficient and safe gene transfer is essential for immunotherapeutic applications like CAR T cell therapy. Restricting lentiviral vector entry to the cell type of interest increases safety and efficacy. This has been shown for lentiviral vectors pseudotyped with measles virus envelope proteins (MV-LV). They are generated by ablating binding to the natural receptors and fusion of single-chain antibodies (scFV) specific for the target protein of choice to the viral envelope protein. Furthermore, MV-LVs enable gene transfer into quiescent T and B cells making it interesting for sophisticated applications. [1, 2]However, for each specificity a novel lentiviral vector has to be engineered and optimized. This process is laborious but also limited to the scFVs or targeting ligands that are available.

**Methods**

We describe here the development of a second generation MV-LV system in order to increase the flexibility and control of targetable lentiviral vectors. To this end, universally targetable MV-LV vectors were generated that are specific for a tag that is only present on adapter molecules that are specific for the antigen on the target cell.

**Results**

These vectors were shown to be functional in the presence of tagged adapter molecules. So far, tagged antibodies and antibody fragments (Fabs) were shown to be suitable adapter formats. No transduction was observed in the absence of adapter or in presence of untagged adapter molecules. Exclusive and selective transduction of the target population was demonstrated using marker gene transferring lentiviral vectors on co-cultures of cell lines. Flow cytometry analysis of transduced cells revealed a 100-1000 fold on-target to off-target ratio depending on the presence or absence of transduction enhancers. The flexibility and efficiency of the novel lentiviral vector system was demonstrated by changing the specificity and concentration of the adapter molecule. Selective transduction of CD4 and CD8 positive T cells within Pan T cells or even PBMC was initially shown with EGFP and confirmed with therapeutically active CARs. Furthermore, transduction of other relevant cell types like CD19 and CD20 expressing B cells as well as CD34 positive hematopoietic stem cells was demonstrated.

**Conclusions**

A highly selective, second generation lentiviral vector system was engineered that is superior in terms of flexibility and control. The gene transfer to the target cell population is now easily adjusted by using an antibody of choice, which renders laborious protein engineering obsolete. This adapter mediated transduction system may thereby be used for a variety of applications in the field of immunotherapy.

**References**

1. Funke S, et al. Targeted cell entry of lentiviral vectors. Mol Ther, 2008; 16(8): 1427-36.

2. Zhou, Q, et al. T-cell receptor gene transfer exclusively to human CD8(+) cells enhances tumor cell killing. Blood, 2012; 120(22): 4334-42.

#### P230 Identification of CD4+ and CD8+ T lymphocyte-epitopes from the cancer testis antigen Lactate dehydrogenase C

##### Julie Decock, PhD, Hibah Shaath, Remy Thomas, Salman Toor, Eyad Elkord, PhD

###### Qatar Biomedical Research Institute, Doha, Qatar

####### **Correspondence:** Julie Decock (jdecock@hbku.edu.qa)

**Background**

Cancer testis antigens (CTA) form a large family of tumor-associated antigens that have gained interest as candidate targets for immunotherapy. Several CTAs have been shown to be re-expressed in tumor cells and to elicit spontaneous strong immune responses. Lactate dehydrogenase C (LDHC) is an archetypical CTA with a restricted expression in normal non-germline tissues. Upregulation of LDHC has been observed in a variety of cancer types and has been associated with a shorter progression free survival in renal cell carcinoma [1,2]. Preliminary work in our group shows that LDHC is strongly expressed in breast cancer, thereby supporting its potential as a biomarker and/or immunotherapeutic target for breast cancer, including the aggressive triple negative breast cancer subtype.

**Methods**

A 15mer LDHC peptide library consisting of 81 peptides with an 11-residue overlap and different MHC preferences was subdivided into peptide subpools (PP) each containing 10-11 individual peptides. In vitro peptide stimulation of peripheral blood mononuclear cells from 7 healthy individuals was used to investigate cytotoxic immune responses against each subpool. Specific T cell responses were assessed using the interferon-γ ELISPOT assay and were defined positive if SFU/105 PBMCs ≥ 10. Multi-marker flow cytometry was used to phenotype the peptide-induced T lymphocytes as CD4+/CD8+ central memory cells (TCM), CD4+/CD8+ effector memory cells (TEM), naïve CD8+ cells and/or effector CD8+ cells.

**Results**

None of the donors tested HLA-A2 positive, warranting moderate resolution HLA typing of all donors in our study, which comprises the less well-studied Arab and Asian ethnic populations. We obtained a range of LDHC peptide-specific responses among the healthy donors. In 3 donors, no increase in IFN-γ release was observed. Donor 4 showed a response against PP2. Donor 2 showed responses against PP2, PP4 and PP5. Donor 3 and donor 7 showed responses to all peptide pools, with the highest responses against PP1, PP3 and PP6. Phenotyping of donor 3’s immune response against PP1 demonstrated a marked increase in IFN-γ secreting CD4+/CD8+ TEM cells (CD45RA- CD45RO+ CCR7- CD62L-) and CD8+ TCM cells (CD45RA- CD45RO+ CCR7+ CD62L+). Further analyses using additional donors are ongoing, including peptide-pulsed dendritic cell assays and individual peptide response analyses.

**Conclusions**

We observed several cytotoxic immune responses against LDHC-specific epitopes, including an increase in central and effector memory CD8+ T lymphocytes in a female Arab donor. Although preliminary, these results suggest that LHDC could be a potential target for cancer immunotherapy in addition to being a potential biomarker.

**References**

1. Koslowski M, Türeci Ö, Bell C, Krause P, Lehr H-A, Brunner J, Seitz G, Nestle FO, Huber C, Sahin U. Multiple Splice Variants of Lactate Dehydrogenase C Selectively Expressed in Human Cancer. Cancer Res. 2002;62:6750–5.

2. Hua Y, Liang C, Zhu J, Miao C, Yu Y, Xu A, Zhang J, Li P, Li S, Bao M, Yang J, Qin C, Wang Z. Expression of lactate dehydrogenase C correlates with poor prognosis in renal cell carcinoma. Tumor Biol 2017;39:1010428317695968.

**Ethics Approval**

The study was approved by the QBRI-HBKU (approval number 2016-002) and Hamad Medical Corporation's Ethics Boards (approval number 17132/17).

#### P231 Uncovering the phenotype, the functional and homing properties of NKG2D CAR T cells

##### Benjamin Demoulin^1^, Eytan Breman, MSc^2^, Sophie AGAUGUE, PhD^2^, David Gilham, PhD^2^, Panagiota Sotiropoulou, PhD^2^

###### ^1^Celyad SA; ^2^Celyad, Mont-St-Guibert, Belgium

####### **Correspondence:** Benjamin Demoulin (bdemoulin@celyad.com)

**Background**

T cells bearing a chimeric antigen receptor (CAR) consisting of the fusion of human NKG2D with the intracellular domain of CD3zeta (CYAD-01) can bind eight stress ligands expressed in many cancers. Initial observations of clinical responses in patients with relapsed/ refractory AML after treatment with CYAD-01 (THINK clinical trial; NCT03018405) supports the potential of this therapeutic approach. To better understand CYAD-01, we performed a thorough characterization of CYAD-01 cells from both healthy donors and AML patients.

**Methods**

CYAD-01 cells were produced from healthy donors and AML patients. Cell culture parameters, as well as the T cell phenotype, the activation / exhaustion status and chemokine receptor expression were assessed at regular intervals during and at the end of the process. CYAD-01 cells were evaluated for their cytotoxic activity and cytokine production upon co-culture with cancer cell lines.

**Results**

CYAD-01 cells from healthy donors consisted predominantly of CD8+ T cells, while the CD4/CD8 ratio of AML- derived CYAD-01 cells was more heterogeneous. In both, CYAD-01 cells consisted mainly of effector memory T cells. The chemokine receptor profile of healthy donor-derived CYAD-01 cells showed relatively high levels of CXCR3, CXCR4 and CCR4 and lower levels of CCR3 for CD8+ cells, and additionally high levels of CCR10 for CD4+ cells. The expression of CXCR4 in particular, suggests that CYAD-01 cells may respond positively to chemokine signalling driving the T cells towards the bone marrow, potentially important for AML treatment. Upon co-culture with cancer cell lines or autologous blasts in an AML sample, CYAD-01 cells secreted high levels of IFN-γ. Upon target challenge, healthy donor-derived CYAD-01 cells secreted a broad array of cytokines including IL-2, IL-4, IL-6 and IL-17A.

**Conclusions**

CYAD-01 cells present as an activated, non-exhausted, predominantly effector memory T cell population.

Interestingly, the expression of CXCR4 may support the ability of CYAD-01 cells to home to the bone marrow, an important property to facilitate targeting of AML leukemic stem cells. The breadth of cytokines produced by CYAD-01 cells confirms the ability of the NKG2D CAR to provide strong activation and co-stimulatory signalling. These results support the encouraging preliminary results from the THINK clinical trial.. Importantly, we are performing large scale investigation of patient material to investigate whether CYAD-01 cells can respond effectively to bone marrow chemokines and to understand if this feature is critical for the clinical responses seen to date in our THINK clinical trial. This will be presented during the conference.

#### P232 Differential effects of target ligands upon NKG2D CAR T cell activation

##### Eytan Breman, MSc^2^, Benjamin Demoulin^1^, Eytan Breman, MSc^2^, Martina Fontaine, PhD^2^, Nancy Ramelot, Master^2^, Sophie AGAUGUE, PhD^2^, David Gilham, PhD^2^, Panagiota Sotiropoulou, PhD^2^

###### ^1^Celyad SA; ^2^Celyad, Mont-St-Guibert, Belgium

####### **Correspondence:** Eytan Breman (ebreman@celyad.com)

**Background**

The Natural Killer group 2D (NKG2D) receptor binds to eight stress-induced ligands (NKG2DL): the major histocompatibility complex class I chain-related A and B (MICA, MICB) and the UL16 binding protein family (ULBP1-6). These ligands are absent from most normal tissues but frequently expressed in many tumors rendering NKG2D a promising means for cancer immunotherapy. We created a chimeric antigen receptor (CAR) T cell containing the full length human NKG2D fused to the CD3zeta signalling domain (termed CYAD-01). As different ligand profiles may elicit differing responses to CYAD-01 cells, we investigated the effect on the distinct ligands on CYAD-01 function in vitro.

**Methods**

To elucidate whether a specific NKG2DL expression pattern is required for NKG2D-based CAR function, CYAD- 01 cells were cocultured with cancer cell lines or in the presence of plate-bound NKG2DLs. Supernatants were harvested and analysed for cytokine secretion, and cytolytic activity was analysed by flow-cytometry.

**Results**

While most cell lines triggered cytotoxicity and cytokine production by CYAD-01 cells, there was a correlation of magnitude of response with relative MICA/B expression and no correlation with ULBP4 expression. To further understand the interaction of NKG2D with each specific ligand, CYAD-01 cells were cultured with individual plate- bound NKG2DL.Both MICA and MICB induced cytokine secretion by CYAD-01 cells from concentrations as low as 0.25 μg/mL, while 5μg/mL or higher concentrations of ULBP1, 2 or 3 were required. No cytokine production could be measured with ULBP4, 5 or 6. However, all the recombinant NKG2DL bound CYAD-01 cells as determined by flow cytometry. These data thus confirmed the trend observed with tumor cell lines.

**Conclusions**

Our data indicate that in a CAR-T context, MICA and MICB are potent activators of T cells engrafted with a NKG2D CAR, while ULBP1, 2 and 3 can induce NKG2D CAR T cell function. Recombinant ULBP4, 5 and 6 bind NKG2D but do not induce a strong effector response in vitro. Importantly, MICA and MICB are the predominant NKG2DL present in human tumors along with ULBP1 and 3. For instance, near 100% of AML blasts express one or more of these specific ligands. While the role of ULBP4,5 and 6 remains unclear, these observations demonstrate that there is a bias of ligand engagement by NKG2D CAR T cells towards target ligands that are broadly expressed across a wide variety of tumors making the approach highly attractive as a therapy.

#### P233 CD8+ tumor-infiltrating lymphocytes expanded from a NSCLC immunosuppressive environment display neoantigen-specific recognition

##### Lorenzo Federico, PhD^1^, Andrea Ravelli^1^, Cara Haymaker, PhD^1^, Marie-Andrée Forget, PhD^1^, Tatiana Karpinets, MS^1^, Jason Roszik, PhD^1^, Donastas Sakellariou-Thompson, BS^1^, Young Uk Kim, PhD^1^, Ankit Bhatta, MSc^1^, May Celestino, BS, MA^1^, Alexandre Reuben^1^, Annikka Weissferdt^1^, Ara Vaporciyan, MD^1^, Mara Antonoff, MD^1^, Garret Walsh^1^, Phillip Andrew Futreal, PhD^1^, Ignacio Wistuba, MD^1^, Jack Roth^1^, Zhang Jianjun^1^, Emily Roarty, PhD^2^, Lara Alvarez de Lacerda-Landry^1^, Stephen Swisher, MD^1^, Tina Cascone, MD, PhD^1^, Gregory Lizee, PhD^2^, John Heymach^1^, Boris Sepesi, MD^1^, Don Gibbons, MD^1^, Chantale Bernatchez^1^

###### ^1^MD Anderson Cancer Center, Houston, TX, USA; ^2^MD Anderson, Houston, TX, USA

####### **Correspondence:** Chantale Bernatchez (cbernatchez@mdanderson.org)

**Background**

The data presented here is part of the ImmunogenomiC prOfiling of Non-small cell lung cancer (ICON) project, a multigroup, cross-disciplinary prospective study designed to identify key immunophenotypic characteristics of early-stage non-small cell lung cancer (NSCLC).

**Methods**

Here, we report the results of ongoing analyses of multiparameter flow cytometry datasets and ex-vivo growth data of lymphocytes present in freshly resected fragments of tumors and matched uninvolved lung tissues from 133 patients. In addition, we present ELISPOT data providing preliminary evidence of selective mutant peptide recognition by sorted CD8+ tumor-infiltrating lymphocyte (TIL) rapidly expanded from 4 patients.

**Results**

Comparison of immunophenotype data in a subgroup of tumor and uninvolved-matched lung tissues (N=53) revealed that cytotoxic CD8+ T cells were on average significantly less abundant in the CD3+ T-cell fraction of the tumor in comparison to uninvolved lung tissues (44% vs. 52%, p< 0.01). CD8+ TIL showed higher mean expression of ICOS (37.6% vs. 11.2%, p< 0.000001) and of the proliferation marker Ki67 (17.3% vs. 6.4%, p< 0.00001). In addition, the CD8+ TIL fraction was found to be composed of a larger percentage of PD1 (57.9% vs 32.5%, p< 0.0001), TIM3 (7.1% vs 3.0%, p< 0.0001), LAG3 (3.7% vs 0.4%, p< 0.00001), and CD103 (56.6% vs 26.2%, p< 0.0001) positive cells as compared to uninvolved lung tissues, and contained significantly less cells expressing granzyme B (6.3% vs. 27.3%, p< 0.000001) and perforin (2.3% vs. 19.1%, p< 0.0000001), indicating that TIL were activated but functionally inhibited.Unsupervised clustering analysis revealed that T-cell infiltrates from tumors grouped in two distinct phenotypic clusters mainly characterized by differential enrichment in LAG3, TIM3, Ki67, and ICOS expressing cells in both CD4+ and CD8+ non-resident (CD103-) T cell fractions. Moreover, tumors segregated according to the percentage of CD8+ TIL and their expression of PD-1.Successful TIL expansion (>12 million cells in 5 weeks) was achieved in 90 of 133 patients tested (67.7% success rate). IFNg ELISPOT analysis of TIL activation in response to predicted neoantigen peptide challenge identified TIL reactivity against 8 private mutations of potential interest in 3 of 4 patients tested suggesting that expanded TIL products contain reactive clones capable of selective neoantigen peptide recognition.

**Conclusions**

Our work indicates that in spite of a highly immunosuppressive microenvironment, TIL can be expanded from the majority of early-stage NSCLC patients, display possible specific reactivity toward mutated peptides, and could thus represent a possible therapeutic option for NSCLC patients.

**Acknowledgements**

We thank all the dedication of our research nurses Mary Ann Gianan and Craig DeGraaf, blood team Patrice Lawson, Heather Napoleon, Mayra Vasquez and Eric Prado, all thoracic OR nurses and anesthesiologists at MD Anderson Cancer Center, and Heidi Wagner, Elena Bogatenkova and the Institutional Tissue Bank team. We could not have completed this work without them. This work was supported by generous philanthropic contributions to The University of Texas MD Anderson Cancer Center Lung Cancer Moon Shots Program and by the NIH/NCI under award number P30CA016672 and used the Tissue Biospecimen and Pathology Resource, Research Histopathology Facility, Bioinformatics Shared Resource, and Biostatistics Resource Group). Special thanks to our patients and their families.

**Ethics Approval**

The study was approved by MD Anderson’s IRB (LAB90-020, PA13-0589 and PA15-1112).

**Consent**

Written informed consent was obtained from the patient for publication of this abstract and any accompanying images. A copy of the written consent is available for review by the Editor of this journal

#### P234 NF-κB p50 deficient immature myeloid cell (p50-IMC) adoptive cell transfer activates the tumor microenvironment to slow tumor growth

##### Alan Friedman, MD, Rahul Suresh, PhD, Theresa Barberi, PhD, David Barakat, PhD, Kenneth Pienta, MD

###### Johns Hopkins University, Baltimore, MD, USA

####### **Correspondence:** Alan Friedman (afriedm2@jhmi.edu)

**Background**

NF-κB p50 binds DNA, but unlike NF-κB p65, it lacks a trans-activation domain and recruits co-repressors.

Absence of p50 results in activation of both macrophages and dendritic cells. Multiple solid tumors, including melanoma, fibrosarcoma, colon, prostate, pancreatic, and glioblastoma, exhibit impaired growth in syngeneic C57BL/6 p50-/- hosts, with M2-to-M1 tumor myeloid cell reprogramming and increased activated tumor T cells. We envision a cell-based therapy wherein patient-derived myeloid progenitors are expanded while targeting their p50 alleles, and then infused back into patients. Immature myeloid cells may localize to the tumor more effectively than mature macrophages. Administering chemotherapy prior to cell infusion may eliminate tumor myeloid cells, release neoantigens to encourage anti-tumor T cell activation, and reduce competing marrow-derived myeloid cell production.

**Methods**

Mice were inoculated with Hi-Myc prostate cancer (PCa), GL261-Luc GBM, or K-Ras(G12D)-Luc pancreatic ductal carcinoma (PDC) cells, the latter two orthotopically. 1-2 weeks later, mice received one intraperitoneal dose of 5-fluorouracil (5FU). 5 days later, lineage-negative marrow cells from p50-/- or WT mice that were expanded in media containing SCF/TPO/FL and transferred to M-CSF for one day were administered intravenously every 3-4 days (1E7 cells x 3). Tumor growth was monitored with calipers or IVIS imaging; myeloid and T cells were analyzed by flow cytometry.

**Results**

Mice bearing PCa showed significantly slowed tumor growth after receiving 5FU on d13 followed by p50-IMC on days 18, 21, and 25, versus mice receiving WT-IMC or 5FU alone (p=0.001), with ~5-fold reduced tumor volume on day 35. For mice implanted with GBM, 3 of 5 manifested very small tumors on day 21, in contrast to the control groups, and 4 of 10 PDC tumors shrank >10-fold in response to 5FU/p50-IMC. WT-IMC from CMV-Luc mice localized preferentially to tumor sites, with infused cells also in lung, spleen, and marrow. Progeny of CD45.2+ IMC were tracked in CD45.1+ tumor hosts. p50-IMC-derived CD11b+ myeloid cells were evident in prostate tumor, draining lymph nodes, spleen, and marrow, with tumor and nodal F4/80+ macrophages displaying an activated MHCII+CD11c+ phenotype. Total PCa CD8 T cells from 5FU/p50-IMC mice were ~5-fold higher than those from 5FU/WT-IMC mice, with increased IFNγ expression in response to PMA/ionomycin and increased surface PD-1, indicative of T cell activation and then exhaustion.

**Conclusions**

Adoptive cell transfer of murine p50-IMC, following a dose of 5FU, activates tumor myeloid and T cells to slow tumor growth and predicts the therapeutic utility of human p50-IMC against multiple solid tumors.

#### P235 STACT-TREX1: A novel tumor-targeting systemically-delivered STING pathway agonist demonstrates robust anti-tumor efficacy in multiple murine cancer models

##### Laura Glickman, PhD, Justin Skoble, PhD, Chris Rae, PhD, Anastasia Makarova, PhD, Marc D'Antonio, Andrew McGeehan, Christopher Thanos, PhD

###### Actym Therapeutics, Inc, Berkeley, CA, USA

####### **Correspondence:** Laura Glickman; Christopher Thanos (cthanos@actymthera.com)

**Background**

Delivery of immunotherapy to directly activate tumor-resident immune cells is required to elicit durable anti-tumor immunity. To this end, we have generated a microbial-based immunotherapy platform (STACT- Salmonella Typhimurium and Checkpoint Therapy) that utilizes a highly attenuated strain engineered to: (a) enhance tumor- specific colonization due to auxotrophic consumption of immunosuppressive adenosine, (b) reduce TLR activation to enhance tolerability and limit immunosuppressive inflammation, (c) enable delivery of engineered RNAi towards any tumor/immune target of interest (alone or in combination), and (d) enhance plasmid maintenance and nuclear delivery. For our initial RNAi target selection, a STACT-TREX1 strain was designed. TREX1 is a 3′ exonuclease immune checkpoint that degrades cytosolic DNA, thereby preventing it from binding cGAS and activating the STING pathway[1]. Mutations in TREX1 cause autoimmune disease and chilblain lupus, characterized by cGAS/STING-dependent overactivation of the type I interferon pathway[2,3]. Systemic delivery of small-molecule inhibitors targeting TREX1 are intractable due to its ubiquitous expression in healthy tissue. We have engineered our systemically-administered therapy to specifically deliver RNAi against TREX1 within the tumor microenvironment. This enables localized cGAS/STING signaling, production of type I interferon, and adaptive immunity against tumor neoantigens, rather than to the delivery vehicle itself.

**Methods**

A potent RNAi against murine TREX1 was selected by screening designed RNAi’s for knockdown of TREX1 gene expression in HEK293 cells, as assessed by qPCR and western blot. A TREX1 RNAi-encoding plasmid was electroporated into an engineered, highly attenuated Salmonella strain with multiple chromosomal modifications that attenuate pathogenicity and enhance tumor-specific plasmid delivery. STACT-TREX1 was evaluated for therapeutic efficacy in subcutaneous CT26 and MC38 colon carcinoma models, and B16.F10 melanoma.

**Results**

Tumor-specific colonization of STACT-TREX1 therapy was observed after tail vein administration. For the dual flank model, distal tumor colonization was observed following intratumoral injection. The therapy was well tolerated, and found to be 1000-fold enriched in tumors relative to spleen and liver. In multiple single and dual flank tumor models, potent tumor growth inhibition and complete tumor regressions were observed. Immune correlates were consistent with STING activation, and the anti-tumor effect was shown to be CD8+ T-cell dependent.

**Conclusions**

A microbial immunotherapy engineered to stimulate the STING pathway in the TME demonstrated significant potency in several murine models of cancer. This therapeutic platform can address multiple solid tumor types without the need for antigen-specific targeting. Beyond TREX1, this platform can be engineered to accommodate combinations of immunostimulatory genes and immune checkpoints in a single therapeutic modality.

#### P236 Flower-code: A novel immunotherapy target

##### Rajan Gogna, PhD, MS, MBA^1^, Christopher Pelham^2^, Esha Madan, PhD, MS, MBA^1^, Taylor Parker^3^, Carlos Carvalho, MD^1^, Antonio Beltran^1^

###### ^1^Champalimaud Centre For Unknown, Lisbon, Portugal; ^2^St. Louis College of Pharmacy, Lisbon, Portugal; ^3^IUPUI, Simon Cancer Center, Libon, Portugal

####### **Correspondence:** Rajan Gogna (rajan.gogna@research.fchampalimaud.org)

**Background**

Cell competition is emerging as a strong concept that explains oncogenic growth as an outcome of competition between neoplastic and stromal cells[1-9]. The enhanced growth potential of cancer cells allows them to behave as super-competitors that eliminate the surrounding normal stromal cell populations. Elimination of normal cells increases space and availability of nutrients, thereby promoting oncogenic growth[1,4-5]. We hypothesize that human cancers are constantly competing with the surrounding stromal tissue, and they use mechanisms governed by cell competition genes such as Flower (C9ORF7) (SITC abstract No.-P200

*Corresponding author email: rajan.gogna@research.fchampalimaud.org). Expression of Flower protein is low in normal body; but is upregulated in cancers; Flower Win and Lose isoforms are expressed in tumor and surrounding stromal tissue, respectively (Fig-1a). This information suggests Flower may serve as a valuable target for cancer immune-therapy.

**Methods**

The functional epitopes of the Win and Lose isoforms of Flower proteins were identified and monoclonal and polyclonal antibodies were raised against those epitopes. Breast, colon, and squamous cell carcinoma FFPE cancer samples were IHC stained. The anticancer potential of anti-Flower monoclonal antibodies was observed in nude mice bearing patient-derived (PDX) triple negative breast (TN) cancer xenografts. The mice were treated with standard care of therapy for TN cancers. The treatment cycles included TAC (5mg/kg docetaxel, 1 mg/kg doxorubicin, 35 mg/kg cyclophosphamide, 1x week) administered in cycles (3 weeks on, 1 week off) to tumor- bearing mice. Tumor growth and mouse survival (120 days) were observed in presence and absence of anti-Flower Ab (80 mg/kg). The effect of this combination therapy was observed on tumor growth, mice survival, and frequency and sites of metastatic lesions.

**Results**

IHC results show that tumor tissue is enriched with Flower Win, whereas Flower Lose isoforms are present in the stromal tissue surrounding the tumors (Fig-1b). Results show a significant reduction in volume of TN PDX observed over a period of 45 days (p<0.0001). Further the KM curves reveal that the mice survival (over 120 days) was significantly longer in the Anti-Flower Ab + TAC compared with TAC alone (P<0.0001) and with controls (P<0.0001). Lastly, combination therapy resulted in a significant reduction in metastatic frequency to kidney, liver, lung, brain, axillary and inguinal lymph nodes (P<0.0001).

**Conclusions**

This preclinical study demonstrated a significant improvement of survival, metastatic events and tumor regression of triple negative patient-derived tumors in mice treated with monoclonal anti-Flower ABs, compared with TAC alone. Preliminary data reveals comparable results with colon and pancreatic PDX tumor models.

**Acknowledgements**

We acknowledge Champalimaud Research Foundation for supporting and funding this research.

**References**

1. Gogna R, Shee K, Moreno E. Cell Competition During Growth and Regeneration. Annu Rev Genet. 2015;49:697- 718. Epub 2015/12/04. doi: 10.1146/annurev-genet-112414-055214. PubMed PMID: 26631518.

2. Penzo-Mendez AI, Chen YJ, Li J, Witze ES, Stanger BZ. Spontaneous Cell Competition in Immortalized Mammalian Cell Lines. PLoS One. 2015;10(7):e0132437. Epub 2015/07/23. doi: 10.1371/journal.pone.0132437. PubMed PMID: 26200654; PubMed Central PMCID: PMCPMC4511643.

3. Tamori Y, Deng WM. Tissue repair through cell competition and compensatory cellular hypertrophy in postmitotic epithelia. Dev Cell. 2013;25(4):350-63. Epub 2013/05/21. doi: 10.1016/j.devcel.2013.04.013. PubMed PMID: 23685249; PubMed Central PMCID: PMCPMC3891806.

4. Moreno E, Rhiner C. Darwin's multicellularity: from neurotrophic theories and cell competition to fitness fingerprints. Curr Opin Cell Biol. 2014;31:16-22. Epub 2014/07/16. doi: 10.1016/j.ceb.2014.06.011. PubMed PMID: 25022356; PubMed Central PMCID: PMCPMC4238900.

5. Levayer R, Moreno E. Mechanisms of cell competition: themes and variations. J Cell Biol. 2013;200(6):689-98. Epub 2013/03/20. doi: 10.1083/jcb.201301051. PubMed PMID: 23509066; PubMed Central PMCID: PMCPMC3601360.

6. Amoyel M, Bach EA. Cell competition: how to eliminate your neighbours. Development. 2014;141(5):988-1000. Epub 2014/02/20. doi: 10.1242/dev.079129. PubMed PMID: 24550108; PubMed Central PMCID: PMCPMC3929405.

7. Petrova E, Lopez-Gay JM, Rhiner C, Moreno E. Flower-deficient mice have reduced susceptibility to skin papilloma formation. Dis Model Mech. 2012;5(4):553-61. Epub 2012/03/01. doi: 10.1242/dmm.008623. PubMed PMID: 22362363; PubMed Central PMCID: PMCPMC3380718.

8. Petrova E, Soldini D, Moreno E. The expression of SPARC in human tumors is consistent with its role during cell competition. Commun Integr Biol. 2011;4(2):171-4. Epub 2011/06/10. doi: 10.4161/cib.4.2.14232. PubMed PMID: 21655431; PubMed Central PMCID: PMCPMC3104570.

9. Merino MM, Rhiner C, Portela M, Moreno E. “Fitness fingerprints” mediate physiological culling of unwanted neurons in Drosophila. Curr Biol. 2013;23(14):1300-9. Epub 2013/07/03. doi: 10.1016/j.cub.2013.05.053. PubMed PMID: 23810538.

**Ethics Approval**

All animal studies performed in this research are approved by Champalimaud Institutional Review Board and the Portuguese Gov Ethical board (DGAV), the approval number is 0421/000/000.

**Fig. 1 (abstract P236).**
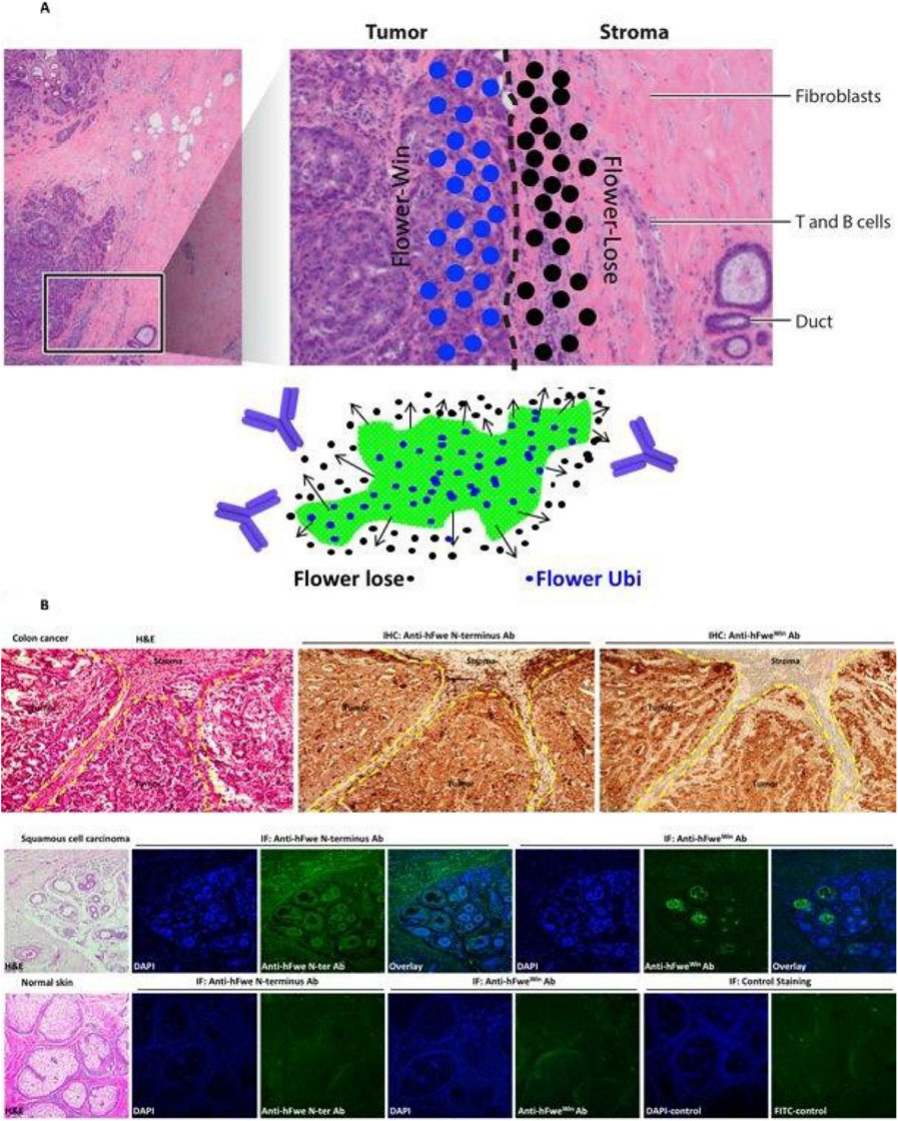
See text for description.

#### P237 CAR-modified killer MSC targeting GD2-positive Ewing sarcoma

##### Giulia Golinelli, PhD^1^, Massimo Dominici^1^, Giulia Grisendi^2^, Carlotta Spano^2^, Filippo Rossignoli^1^, Malvina Prapa^1^, Angela d'Esposito^1^

###### ^1^University of Modena and Reggio Emilia, Modena, Italy; ^2^Rigenerand S.R.L., Modena, Italy

####### **Correspondence:** Giulia Golinelli (giulia.golinelli@unimore.it)

**Background**

Ewing sarcoma (ES) is an aggressive mesenchymal-derived tumor representing the second most common malignancy in children and young adults. Despite a marked improvement in the prognosis of patients, the mortality caused by metastases and recurrent disease remains high calling for novel strategies to sustain remission and improve outcome. New avenues of research have been opened by using the pro-apoptotic agent TNF-related apoptosis inducing ligand (TRAIL) and cells carrying TRAIL close to tumor have shown to increase its bioavailability. Mesenchymal stromal cells (MSC) have been under investigation as vehicles for the delivery of anti- tumor agents. We previously demonstrated that MSC expressing TRAIL can induce apoptosis in a variety of sarcomas exerting also a relevant antitumor activity against in vivo models of ES. However, while the interaction between TRAIL and receptors is clear, more obscure is the manner in which MSC can selectively target tumors. As metastases are a great challenge in ES patients, dedicated strategies to drive MSC targeting and persistence to metastatic sites, particularly involving lungs in ES, shall be required. Here, in an effort to maximize the therapeutic profile of MSC TRAIL, minimize off-target effects and accounting for metastatic disease, we originally developed a strategy where TRAIL is delivered by MSC that are also modified by an anti-GD2 chimeric antigen receptor (CAR) to target GD2-positive ES cells.

**Methods**

The anti-GD2 CAR was expressed in MSC by viral transduction together with TRAIL. The anti-tumor activity of these functionalized MSC was in vitro assessed targeting GD2-positive or negative ES lines. The enhanced binding ability of functionalized MSC to GD2-positive ES cells and the specificity of interaction was investigated, as well, both in vitro and in vivo.

**Results**

The functionalized MSC expressed high levels of both TRAIL and CAR preserving a robust anti-tumor activity against ES lines. Most importantly, the functionalized MSC killing was further reinforced by an enhanced targeting thanks to improved cell-to-cell interactions. Based on in vitro findings, we started to assess the anti-GD2 CAR potential in an in vivo lung metastases ES model to better elucidate the advantage conferred by the CAR on MSC- based delivery.

**Conclusions**

Our results suggest that the CAR here described might be a powerful tool to redirect MSC carrying TRAIL against GD2-expressing tumors. This targeting strategy holds the promise to combine site-specific and prolonged retention of MSC in ES metastases, thereby providing a more effective delivery of TRAIL against this still incurable cancer even after metastatic dissemination.

**Acknowledgements**

The work is supported in parts by the “Associazione Italiana per la Ricerca sul Cancro” (AIRC, IG 2015), the Associazione Sostegno Ematologia Oncologia Pediatrica (ASEOP) and by “Progetto Dipartimenti Eccellenti MIUR 2017”

**Ethics Approval**

The study was approved by the Ministry of Health (Italy), approval number 1278/2015-PR.

#### P238 Regulation of in vivo anti-tumor activity of adoptively transferred CAR-T cells using FDA approved small molecule drugs

##### Jennifer Gori, PhD, Brian Dolinski, BS, Scott Heller, MS, Mara Inniss, Abhishek Kulkarni, Dan Jun Li, MD, Christopher Reardon, Dexue Sun, Karen Tran, MS, Michelle Ols, PhD, Jennifer Gori, PhD, Vipin Suri, Celeste Richardson, Steven Shamah, PhD

###### Obsidian Therapeutics, Cambridge, MA, USA

####### **Correspondence:** Jennifer Gori (jgori@obsidiantx.com)

**Background**

Adoptive cell therapy with chimeric antigen receptor (CAR)-modified T cells has demonstrated clinical efficacy in the treatment of B cell malignancies and multiple myeloma. More widespread application of CAR-T cell therapy has been restricted by concerns about safety and observations of limited efficacy. Uncontrolled expansion of CD19- targeting CAR-T cells has caused severe CRS-related toxicity in many patients. Tumor antigen expression on healthy tissues can lead to on-target/off-tumor toxicity. Furthermore, prolonged antigen-dependent or independent CAR signaling may contribute to functional exhaustion, limiting efficacy. Adoptive transfer of T cells expressing a reversible, titratable, regulated CAR would support exogenous control of the level, activity, and timing of CAR expression for sustained anti-tumor efficacy and a more favorable safety profile. To exert control over CAR-T cells, we used destabilizing domain (DD) technology, fusing CAR to small protein domains which are unstable and degraded intracellularly but are reversibly stabilized by small molecule ligand binding, enabling exogenous control.

**Methods**

Using structure-guided engineering and mutagenesis screens, we identified mutations that destabilize human phosphodiesterase 5 (PDE5) protein and restabilize in the presence of FDA approved PDE5 inhibitors. To provide exogenous control over CAR-T cells, we fused destabilizing mutant-containing PDE5 domains (PDE5-DDs) to CD19-CAR (CD19-CAR-PDE5-DD), transduced human T cells with DD-modified CAR, and evaluated ligand dose-responsive CAR expression and activity. To evaluate anti-tumor activity in vivo, we implanted NSG mice with CD19+luciferase+Nalm6 cells and transplanted unmodified T cells or CD19-CAR-PDE5-DD T cells. We orally dosed mice with ligand or vehicle and monitored tumor growth by bioluminescent imaging to track tumor progression. We applied Kaplan-Meier analysis and the log-rank test to compare survival curves and median survival time.

**Results**

Human T cells transduced with CD19-CAR-PDE5-DD and treated with PDE5 ligands showed ligand-dependent CAR expression and activity in vitro. CD19 tumor-bearing mice treated with CD19-CAR-PDE5-DD T cells and stabilizing ligand showed a dose-dependent delay in tumor progression relative to ligand treated unmodified T cell recipients. Tumor growth inhibition was significant across ligand doses for CD19-CAR-PDE5-DD T cell recipients relative to unmodified T cell recipients. A significant survival advantage was observed in mice treated with CD19- CAR-PDE5-DD T cells relative to mice treated with unmodified T cells (P<0.005).

**Conclusions**

PPDE5-regulated CD19-CAR-T cell activity supported robust anti-tumor efficacy and increased survival in vivo.

These preclinical studies demonstrate the potential to reversibly stabilize a DD-regulated CAR in vivo using FDA approved small molecules, toward enhancing safety and efficacy of CAR-T therapies for the treatment of cancer

#### P239 Novel solid tumor gene therapy approach mediated by engineered Bifidobacterium longum generates efficient tumor-derived IL-12 expression and results in local and systemic anti-tumor immunity

##### Sheetal Raithatha, Umesh Ramachandran, Melissa Dennis, Elena Topchy, Steven Zhao, Alexander Graves

###### Symvivo Corporation, Burnaby, Canada

####### **Correspondence:** Alexander Graves (agraves@symvivo.com)

**Background**

Intravenous infusion of the probiotic obligate anaerobe, Bifidobacterium longum, selectively colonizes solid tumor tissues in preclinical models. Seeking to therapeutically exploit this observation, bifidobacteria was engineered to selectively shuttle plasmid DNA (pDNA) into solid tumor tissues resulting in membranous expression of therapeutically relevant transgenes such as interleukin-12 (IL-12).

**Methods**

An engineered, multi-domain fusion protein expressed locally by tumor-colonizing bacteria coordinates the binding and secretion of therapeutic pDNA molecules into the tumor microenvironment, mediating efficient genetic transfection of cancer cells, ultimately achieving tumor-derived expression of therapeutic genes. Membrane-bound IL-12 tumor expression was achieved in syngeneic mouse tumor models of colorectal cancer (CT-26) and melanoma (B16F10).

**Results**

In both models, B.longum engineered to deliver the IL-12 gene administered intravenously was well-tolerated and selectively colonized tumors. Robust and increasing expression of IL-12 was demonstrated within tumor tissues. Bacteria and/or transgene expression was undetectable in a comprehensive assessment of normal tissues. As predicted, IL-12 expression by the tumor resulted in the activation of both adaptive and innate immune responses. Increased pro-inflammatory cytokine levels and elevated tumor CD8+/Treg ratios were associated with the recognition of cancer specific antigens. B.longum mediated IL-12 gene delivery significantly inhibited tumor growth and combination therapy with anti-PD1 and anti-CTLA4 synergistically improved these outcomes.

**Conclusions**

Taken together, selective bacterial colonization of solid tumors employing intravenously-infused, genetically- engineered B.longum achieves robust and enduring tumoral expression of therapeutically relevant genes such as IL-12. A first-in-human clinical trial is planned for 2019.

**Table 1 (abstract P239).**
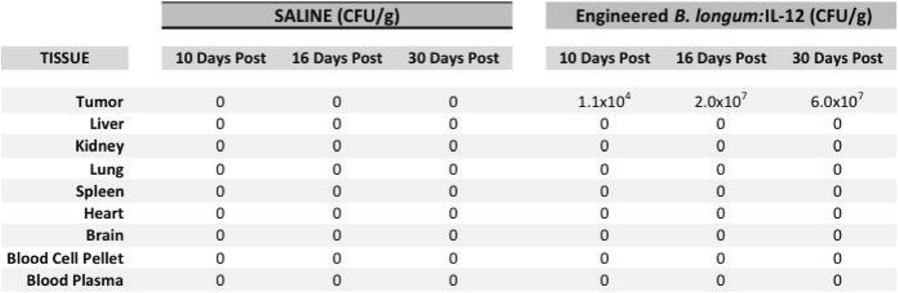
Biodistribution of engineered *B. longum* upon intravenous infusion

**Fig. 1 (abstract P239).**
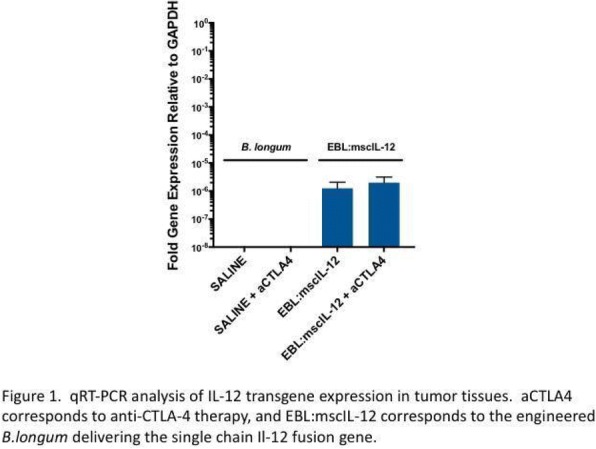
See text for description.

**Table 2 (abstract P239).**
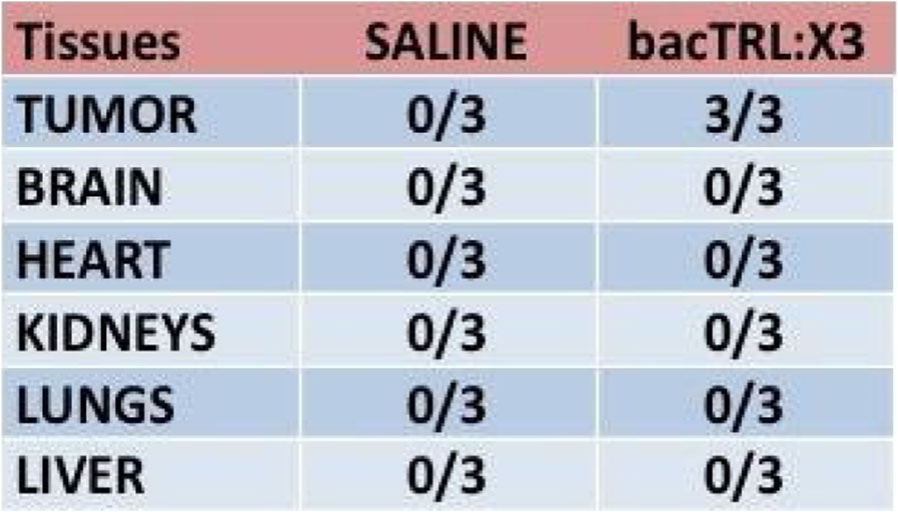
qRT-PCR analysis of transgene expression in tumor and non-tumor tissues

**Fig. 2 (abstract P239).**
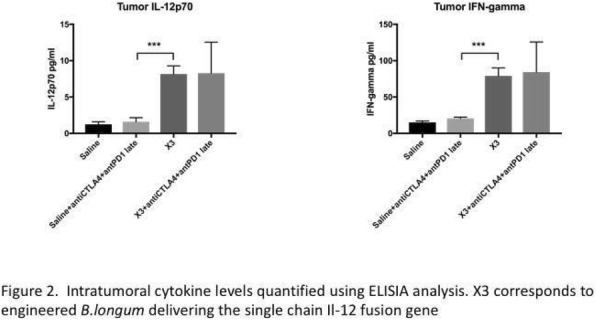
See text for description.

**Fig. 3 (abstract P239).**
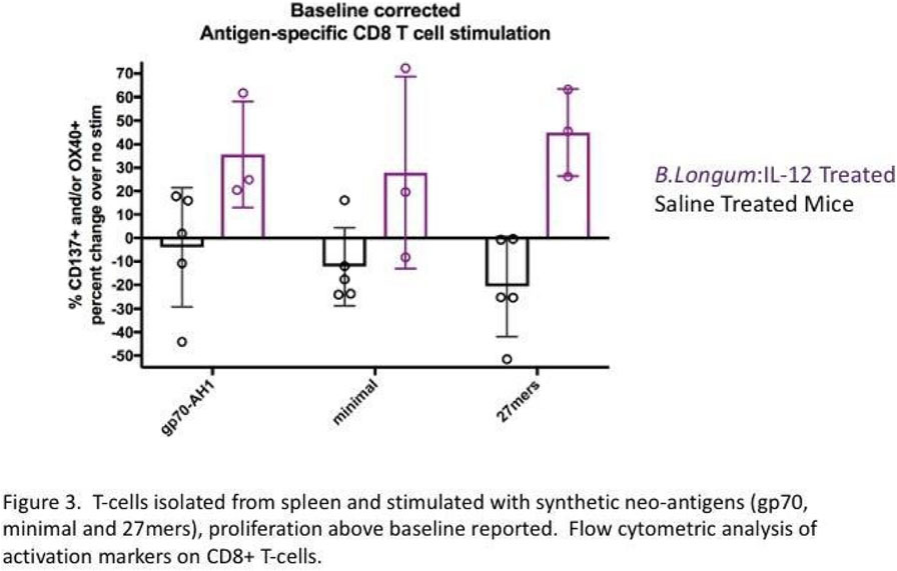
See text for description.

**Fig. 4 (abstract P239).**
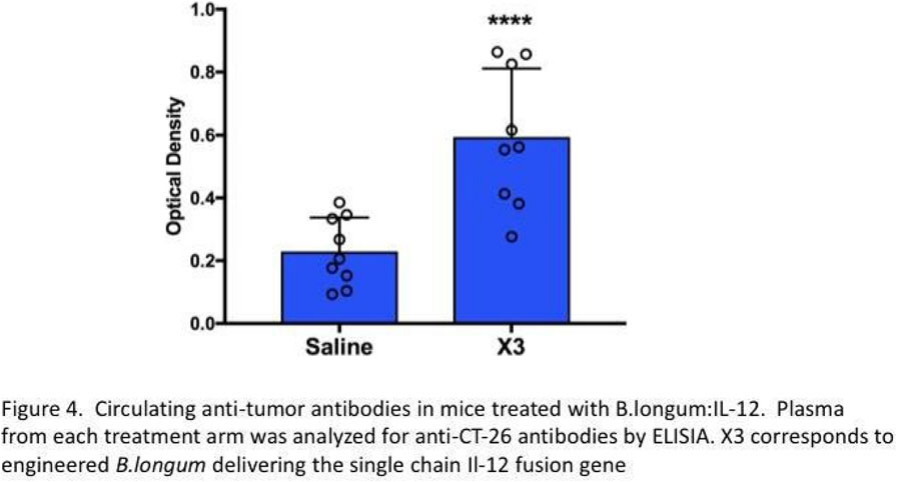
See text for description.

**Fig. 5 (abstract P239).**
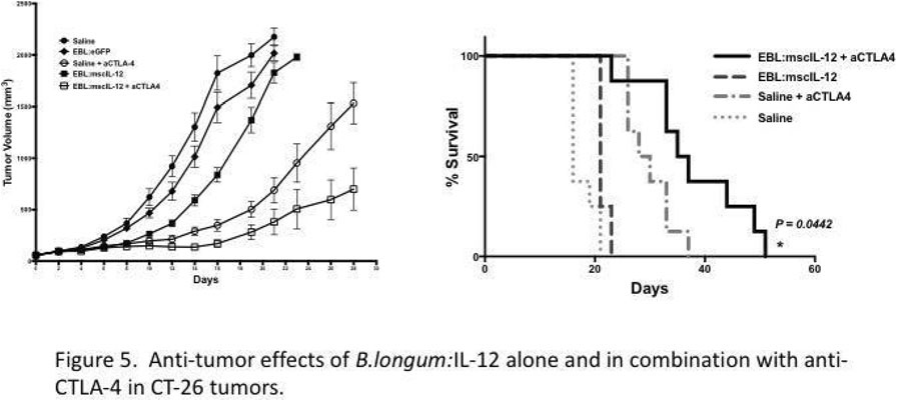
See text for description.

**Fig. 6 (abstract P239).**
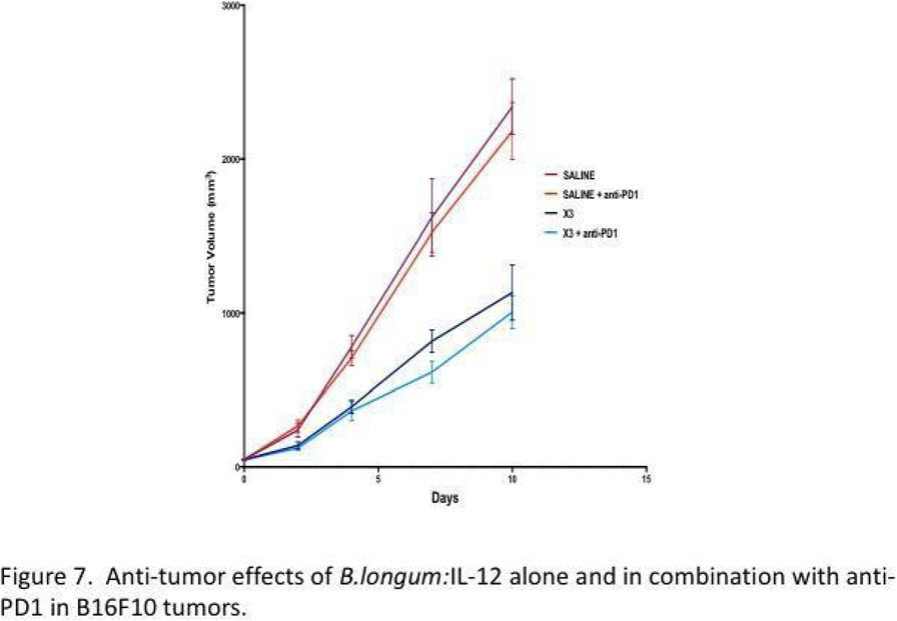
See text for description.

#### P240 Autologous Epstein-Barr virus (EBV)-specific T cells (baltaleucel T): preliminary results from a multicenter, multinational phase 2 study for treatment of EBV-associated NK/T cell lymphoma

##### Kurt Gunter, MD^1^, Yasuhiro Oki, MD^2^, Won Seog Kim^3^, Kirit Ardeshna, MB BChir MA MD FRCP FRCPath^4^, Yi Lin, MD PhD^5^, Jia Ruan, MD, PhD^6^, Pierluigi Porcu, MD^7^, Jonathan Brammer, MD^8^, Eric Jacobsen, MD^9^, Dok Hyun Yoon, MD^10^, Cheolwon Suh, MD PhD^10^, Felipe Suarez, MD PhD^11^, John Radford, MD^12^, Lihua Budde, MD PhD^13^, JIN SEOK Kim, MD PhD^14^, Gilles Salles, MD^15^, Hun Ju Lee, MD^2^, Catherine Bollard, MD^16^, Arnaud Jaccard, MD PhD^17^, Hye Jin Kang, MD PhD^18^, Shannon Inman, BS^1^, Maryann Murray^1^, Katherin Combs, RN^1^, Daniel Lee, MD PhD^19^, Ranjana Advani, MD^20^, Center director Rooney, PhD^21^, Helen Heslop, MD^21^

###### ^1^Cell Medica Inc, Houston, TX, USA; ^2^MD Anderson Cancer Center, Houston, TX, USA; ^3^Samsung Medical Center, Seoul, Korea, Republic of; ^4^University College London Hospitals, London, UK; ^5^Mayo Clinic, Rochester, MN, USA; ^6^Weill Cornell Medical School, New York, NY, USA; ^7^Thomas Jefferson University, Philadelphia, PA, USA; ^8^The Ohio State University, Columbus, OH, USA; ^9^Dana Farber Cancer Institute, Boston, MA, USA; ^10^Asan Medical Center, Seoul, Korea, Republic of; ^11^Hôpital Universitaire Necker, Paris, France; ^12^University of Manchester, Manchester, UK; ^13^City of Hope National Medical Center, Duarte, CA, USA; ^14^Yonsei University College of Medicine, SEOUL, Korea, Republic of; ^15^Centre Hospitalier - Université de Lyon, Pierre Benite, Auvergne, France; ^16^Children's National Medical Center, Houston, TX, USA; ^17^CHU de Limoges, Limoges, France; ^18^Korea Cancer Center Hospital, Seoul, Korea, Republic of; ^19^Houston Methodist Research Institute, Houston, TX, USA; ^20^Stanford Cancer Institute, Stanford, CA, USA; ^21^Baylor College of Medicine, Houston, TX, USA

####### **Correspondence:** Kurt Gunter (kurt.gunter@cellmedica.com)

**Background**

Advanced NK/T-cell lymphoma (NKTL) is a rare lymphoma associated with EBV expression and an aggressive course. Standard treatment for advanced disease involves asparaginase based regimens, however relapses are common. Herein we report early results from a phase 2 study (CITADEL, NCT01948180) of autologous EBV- specific T cells (baltaleucel T) for the treatment of NKTL.

**Methods**

The study was conducted in France, South Korea, the UK and the US. Patients with relapsed NKTL who had received prior asparaginase based therapy were eligible. 200 mL of whole blood was collected from the patient and the product was manufactured using EBV peptide stimulation and cytokines. Patients received 2 to 5 doses of 2x10e7 CD3+ cells/m2. The primary endpoint was overall response rate (ORR) by Lugano criteria, as assessed by independent review.

**Results**

As of 10 April 2018, 15 patients with relapsed NKTL were administered baltaleucel T, either as adjuvant therapy for patients without measurable disease due to bridging chemotherapy during manufacturing (n=5) or as treatment for patients with measurable active disease (n=10). Manufacturing success rate was approximately 71% under current release specifications. Two serious adverse events (AEs) (lymphoma pain and hyperbilirubinemia) were observed and attributed as possibly related to baltaleucel T. There were 10 unique non-serious product-related AEs of mild/moderate severity observed in 3 patients. No cytokine release syndrome or neurotoxicity was observed. Of the 5 patients with non-measurable disease, 2 remain on study without disease progression while 3 progressed. Of the 10 patients with measurable disease, 8 patients were evaluable for response and 2 patients withdrew early due to progression. In the predefined evaluable population (patients with measurable disease and imaging at 8 weeks), the ORR is 62.5% (5/8), and in all patients with measurable disease 50% (5/10). In the responding patients, the best response was 3 complete remissions (CRs) and 2 partial remissions (PRs). Median duration of response (DoR) was 3.5 months by Kaplan-Meier analysis. (DoR censored for hematopoietic stem cell transplantation or ongoing response.) For all 15 patients, the median overall survival (OS) is 13.4 months and the OS at 1 year is 60% with a median follow up 10.0 months (Figure 1). All responding patients remain alive.

**Conclusions**

Our study demonstrates feasibility, clinical activity and safety of administration of single agent autologous EBV-specific T cells in patients with advanced, relapsed NKTL in a multicenter, multinational trial. These results require validation in a larger cohort.

**Acknowledgements**

We thank the participating patients and their families.

**Trial Registration**

NCT01948180

**Ethics Approval**

The study was approved by all applicable institutional review boards and ethics committees.

**Fig. 1 (abstract P240).**
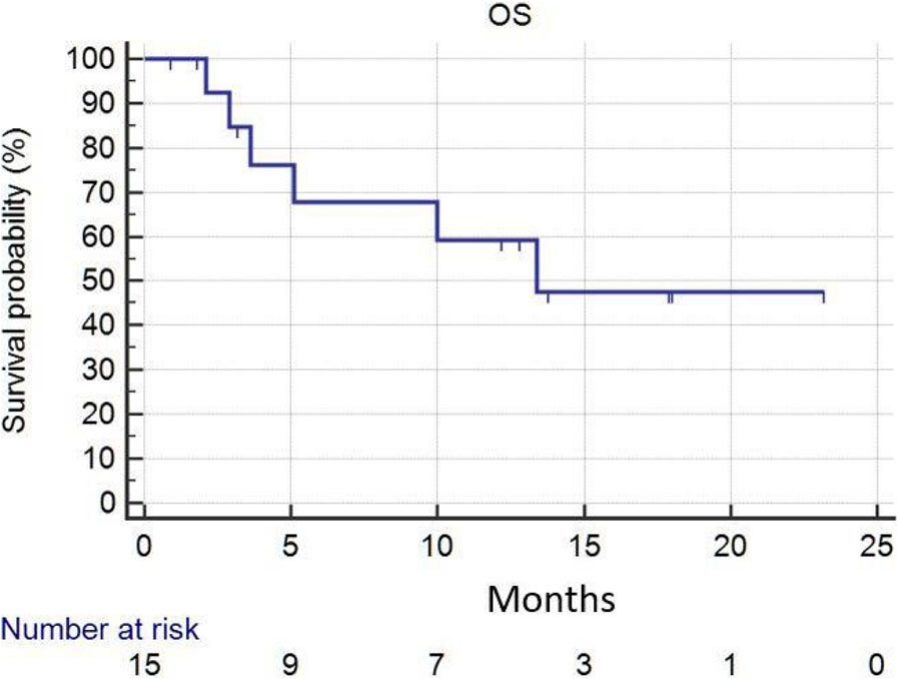
CITADEL OS estimated by Kaplan-Meier method (n=15)

#### P241 Melanoma-derived human CD4+ tumor-infiltrating lymphocytes (TIL) demonstrate a functional response to autologous tumor

##### MacLean Hall, Autumn Joerger, Ellen Scott, Ben Schachner, Allison Richards, Valerie Stark, Jeani Rich, Amy Weber, Doris Wiener, Krithika Kodumudi, PhD, Matthew Beatty, PhD, Amod Sarnaik, MD, Shari Pilon- Thomas, PhD

###### Moffitt Cancer Center, Tampa, FL, USA

####### **Correspondence:** Amod Sarnaik (amod.sarnaik@moffitt.org)

**Background**

Current strategies of Adoptive Cell Therapy (ACT) with Tumor-Infiltrating Lymphocytes (TIL) involve the expansion and selection of tumor-reactive CD8+ T cells for infusion into metastatic melanoma patients. Recent studies point to an expanded role of CD4+ T cells in anti-tumor immunity, warranting further investigation into the function and potential efficacy of this underexplored TIL population. [1,2]

**Methods**

TIL were isolated from surgically resected tissues from patients with metastatic melanoma at Moffitt Cancer Center.

TIL were expanded in media containing IL-2 and subjected to the Rapid Expansion Protocol (REP). CD4+ and CD8+ TIL were isolated using magnetic bead negative selection. TCRbeta sequencing was performed using Adaptive Biotechnologies AdaptImmune platform. CD4+ TIL were stimulated with autologous tumor or dendritic cells (DC), or anti-CD3/CD28 and assayed for cytokine production by ELISA and flow cytometry.

**Results**

A complete response (CR) was achieved following infusion of predominantly (88%) CD4+ TIL in one metastatic melanoma patient. TCRbeta analysis showed a 64.4% overlap between CD4+ infused TIL clones and peripheral blood two weeks after adoptive transfer, which declined to 2.3% at six weeks. CD4+ TIL from 11 additional patients stimulated with anti-CD3/CD28 showed a pleiotropic cytokine repertoire (IFN-gamma, TNF-alpha, IL-5, IL-6, and IL-13), which was confirmed by flow cytometry. Responders (n=4) displayed an elevated Th1/Th2 cytokine profile compared to non-responders (n=7; p=0.0725). CD4+ TIL from four additional patients displayed robust IFN-gamma production in response to DC loaded with autologous tumor. Upregulation of MHC class II on autologous tumor cells stimulated CD4+ TIL to produce both IFN-gamma (4.0%) and TNF-alpha (5.9%). Recognition of autologous tumor was diminished in the presence of antibody blockade of MHC class II.

**Conclusions**

CD4+ TIL persisted in vivo and demonstrated a MHC class II-restricted response to autologous tumor, marked by a Th1 polarized cytokine profile. These data support further examination of the mechanisms by which CD4+ TIL may improve therapeutic efficacy in ACT.

**References**

1. Bailey SR, et al. Human CD26high T cells elicit tumor immunity against multiple malignancies via enhanced migration and persistence. Nat Commun. 2017; 8(1): 1961.

2. Tran E, et al. Cancer immunotherapy based on mutation-specific CD4+ T cells in a patient with epithelial cancer. Science. 2014; 344(6184): 641-5.

**Ethics Approval**

MCC15781 was approved by USF IRB approval number Ame5_P112

*Corresponding author email: m.rucevic@olink.com5.MCC16992 was approved by Advarra IRB approval number 14.03.0083.MCC17057 was approved by USF IRB approval number Ame13_Pro00009061.MCC18377 was approved by Advarra IRB approval number MOD00206092.

#### P242 Withdrawn

#### P243 Tumour infiltrating lymphocyte therapy: clinical outcomes in pre-treated metastatic melanoma patients and biomarker correlations

##### Robert Hawkins, MD, PhD^1^, Manon Pillai, MB BS MD^1^, Ryan Guest^2^, Natallia Kirillova^2^, Paul Lorigan^1^, Michelle Le Brocq^2^, John Bridgeman, BSc, MSc, PhD^2^

###### ^1^Chrisitie Hospital, Manchester, UK; ^2^Immetacyte Ltd, Manchester, UK

####### **Correspondence:** Robert Hawkins (Robert.e.Hawkins@manchester.ac.uk)

**Background**

Adoptive cell therapy (ACT) with tumour infiltrating lymphocytes (TIL) has consistently demonstrated impressive clinical results in several studies in the management of metastatic melanoma. For patients who progress on current therapies (checkpoint inhibitors and B-RAF inhibitor based therapy) the outlook is poor and there is a clear unmet medical need. We describe our experience as the only UK cancer centre providing TIL therapy.

**Methods**

TIL are cultured from resected tumour samples as previously described. The process is GMP compliant with cells manufactured under an MHRA specials licence and the process is applicable to large scale use. Infusion is preceded by non-myeloablative lymphodepleting chemotherapy (high dose cyclophosphamide and fludarabine) and followed by intravenous high dose interleukin 2 (HD-IL2). A range of biomarkers were assessed for potential correlation with outcome.

**Results**

Nineteen patients with progressive metastatic cutaneous melanoma who have failed other therapies have been treated to date. All patients had metastatic disease and were heavily pre-treated with a combination of targeted agents and immunotherapies (anti-CTLA4 and anti-PD1 antibodies). 11/19 (57%) achieved an objective clinical response according to the response evaluation criteria in solid tumours. 4/19 (21%) achieved a complete response and all of those are on-going (18+ to 72+ months). Treatment is well tolerated. All patients experienced anticipated toxicities associated with pre-conditioning chemotherapy and HD-IL2, which were short lived and manageable on the medical ward. The only autoimmune toxicity seen is vitiligo and there was no clinically significant cytokine release syndrome seen. The median overall survival is 15 months but several patients also had prolonged stable disease or partial responses and there is a plateau in the Kaplan-Meir survival curve with estimate 5 year survival of 40%. A range of biomarkers correlate with response and survival.

**Conclusions**

We show that at our centre lympho-depleting chemotherapy followed by transfer of TIL and HD-IL2 is feasible and clinically effective, demonstrating tumour regression in over 50% of patients with metastatic cutaneous melanoma. Importantly long-term durable responses were seen including 21% complete durable remissions. Overall, there is clear durable clinical benefit in a group of patients with no other good current options for therapy. Biomarker analysis suggest the potential to select patients for treatment and way to improve the outcome even more. Base on pre-clinical data (1,2) we are also starting to explore TIL based therapy and engineered TIL in other cancers.

**References**

1. Owens GL, Price MJ, Cheadle EJ, Hawkins RE, Gilham DE, Edmondson RJ. Ex vivo expanded tumour- infiltrating lymphocytes from ovarian cancer patients release anti-tumour cytokines in response to autologous primary ovarian cancer cells. Cancer Immunol Immunother. 2018 Jul 23; doi: 10.1007/s00262-018-2211-3. 2. Baldan V, Griffiths R, Hawkins RE, Gilham DE. Efficient and reproducible generation of tumour-infiltrating lymphocytes for renal cell carcinoma. Br J Cancer. 2015 Apr 28; 112(9):1510-8.

#### P244 Reactive myelopoiesis triggered by lymphodepletion limits the efficacy of adoptive T cell therapy

##### Patrick Innamarato, BS^1^, Shari Pilon-Thomas, PhD^1^, Krithika Kodumudi, PhD^1^, Amy Weber^1^, Amod Sarnaik, MD^1^, Doris Weiner^1^, Jennifer Morse, MS^1^, Michael Kidd^1^, Patrick Verdugo^1^

###### ^1^Moffitt Cancer Center, Tampa, FL, USA; ^2^University of South Florida, Tampa, FL, USA

####### **Correspondence:** Shari Pilon-Thomas (shari.pilon-thomas@moffitt.org)

**Background**

Lymphodepleting chemotherapy administered before adoptive T cell therapy (ACT) enhances anti-tumor immune responses by increasing availability of cytokines necessary for the persistence and function of infused T cells. However, the induction of lymphopenia by nonmyeloablative chemotherapy also initiates the rapid expansion of highly immunosuppressive myeloid derived suppressor cells (MDSCs) that inhibit anti-tumor responses elicited by adoptively transferred T cells. Here, we investigated the role of lymphodepletion-induced MDSCs (LD-MDSCs) on ACT using mouse models and patients that received autologous tumor infiltrating lymphocytes (TIL).

**Methods**

In B16 tumor-bearing mouse models, single doses of cyclophosphamide and fludarabine were used to induce lymphodepletion. T cell proliferation was assessed by co-culturing MDSCs from C57BL/6 (WT) and IL-6KO mice and ACT models were carried out using donor pmel T cells. MDSC frequency and function were evaluated in a cohort of melanoma patients that received ACT with TIL pre- and post-infusion using flow cytometry and co-culture assays.

**Results**

Using mouse models, we show that the ability of IL-6KO MDSCs to suppress T cell proliferation is no different compared to WT mice. However, upon lymphodepletion the suppressive capacity of IL-6KO MDSCs is significantly attenuated, indicating that MDSCs arising after lymphodepletion are dependent on IL-6 signaling. We then confirmed these findings in a myeloid-specific IL-6R knockout model (IL-6RM-KO). In ACT models, we demonstrate that IL-6KO and IL-6RM-KO recipient mice exhibit significant delays in tumor growth and increased survival compared to WT mice receiving ACT. Melanoma patients that received ACT with TIL exhibited a dramatic increase (3-250 fold) of MDSCs one week after treatment with lymphodepleting chemotherapy and TIL infusion compared to the pre-treatment frequency. Serum levels of IL-6 in these patients are also elevated compared to healthy donors. At one week post-infusion of TIL, we assessed 24 patient PBMCs and show that patients with <10% PMN-MDSCs have improved overall survival, progression-free survival, and objective response rates. Furthermore, we show that at week one and week two post-infusion, one patient exhibited >60% PMN-MDSCs (CD15+LOX- 1+CD11b+) that inhibited T cell proliferation, expressed IL-6R, PD-L1, and high levels of ROS.

**Conclusions**

Together, these data suggest that targeting IL-6 signaling inhibits the immunosuppressive functions of MDSCs in a lymphodepleted setting and may enhance the efficacy of ACT with TIL. To our knowledge, this is the first study to demonstrate that MDSCs have a significant impact on the survival of patients receiving adoptive T cell therapy.

**Ethics Approval**

All animal protocols were reviewed and approved by the Institutional Animal Care and Use Committee at the University of South Florida. MCC15781 was approved by USF IRB approval number Ame5_P112

*Corresponding author email: m.rucevic@olink.com5.MCC16992 was approved by Advarra IRB approval number 14.03.0083.MCC17057 was approved by USF IRB approval number Ame13_Pro00009061.MCC18377 was approved by Advarra IRB approval number MOD00206092.

#### P245 Enrichment of rare antigen-specific CD8+ T cells directly from splenocytes improves output and throughput

##### Ariel Isser, John Hickey, BS, Kayla Gee, Sebastian Salathe, Yi Dong, Jonathan Schneck, MD, PhD

###### Johns Hopkins University, Baltimore, MD, USA

####### **Correspondence:** Jonathan Schneck (jschnec1@jhmi.edu)

**Background**

Adoptive immunotherapy, which involves expanding a patient’s tumor antigen-specific cytotoxic (CD8+) T cells, presents a unique challenge as only 1 in 10,000-1,000,000 CD8+ T cells are specific to a particular antigen, leading to production processes that are generally low throughput, expensive, and difficult to standardize. Our laboratory has previously reported that paramagnetic iron dextran artificial antigen presenting cells (aAPCs) conjugated with peptide loaded major histocompatibility complex (pMHC) and anti-CD28, a costimulatory molecule, can be used to enrich and expand antigen-specific CD8+ cells to clinically relevant levels using a magnetic column [1]. To make this technique more translational, we aimed to decrease cost and complexity while improving throughput by performing enrichments directly from splenocytes without a prior CD8+ purification step and by using larger particles compatible with a 96 well plate magnet to batch multiple antigens.

**Methods**

To compare splenocyte to CD8+ enrichments, purified splenocytes from C57BL/6 mice were split into two equal fractions, only of which underwent a CD8+ isolation. Both fractions were enriched with 100 nm KbSIY/anti-CD28 or KbTRP2/anti-CD28 aAPCs over a magnetic column and then cultured for a week, after which cell counts and MHC-Ig dimer stains were used to assess fold expansion and antigen-specificity. Plate based enrichments were performed in a similar manner, except that larger 300 nm aAPCs compatible with a plate magnet were used for the enrichments.

**Results**

Enrichment directly from splenocytes is not only feasible but results in 80% antigen-specificity and triple the number of antigen-specific cells as from CD8+ enrichments after a week of expansion. The expanded cells are polyfunctional as seen through intracellular cytokine stains. Helper T cells and dendritic cells are vital to the improved expansions seen in the splenocyte enrichments. Plate-based enrichments work effectively at a particle to cell dose above 680:1, result in robust expansions, and can be successfully used to expand three different populations of antigen-specific cells simultaneously.

**Conclusions**

Splenocyte as opposed to CD8+ enrichments provide a faster, cheaper, and more effective method to expand large populations of mostly antigen-specific cells, improving the scalability of cellular therapies that target patient- specific rare cancer neoantigens, lowering the risks of nonspecific off-target effects, and suggesting similar improvements may be seen with human PBMC as opposed to CD8+ enrichments. Moreover, 96 well plate based platforms can allow for wider screens of rare antigen-specific populations and simultaneous targeting of multiple cancer neoantigens.

**References**

1. Perica K, Bieler JG, Schütz C, Varela JC, Douglass J, Skora A, et al. Enrichment and expansion with nanoscale artificial antigen presenting cells for adoptive immunotherapy. ACS Nano. 2015; 9(7):6861–71.

#### P246 Targeting novel tumor-associated antigens with TCR-engineered T Cells

##### Mamta Kalra, PhD, Ali Mohamed, PhD, Zoe Coughlin, Thorsten Demberg, PhD, Amir Alpert, BS, Leoni Alten, Sebastian Bunk, PhD, Claudia Wagner, Jens Fritsche, PhD, Oliver Schoor, PhD, Agathe Bourgogne, Yannick Bulliard, PhD, Geoffrey Stephens, Dominik Maurer, PhD, Harpreet Singh, PhD, Carsten Reinhardt, MD, PhD, Tony Weinschenk, Steffen Walter, PhD

###### Immatics US Inc., Houston, TX, USA

####### **Correspondence:** Steffen Walter (Walter@immatics.com)

**Background**

A major constraint to the broad applicability of Adoptive Cellular Therapy is the limited number of known tumor- specific targets that are safe and effective, especially against solid tumors. Unlike CAR-T cells that recognize only surface expressed tumor antigens, T-Cell Receptor (TCR) engineered T cells can access a wider repertoire of tumor targets including intracellular antigens presented in context of HLA molecules.

**Methods**

Here, we present the development of TCR-engineered T-cell products against 3 novel peptide-MHC antigens identified and validated by Immatics’ proprietary XPRESIDENT® platform. This platform combined with an extensive safety assessment program supports our proprietary TCR Discovery platform to screen TCR candidates for potential off-target toxicities. Selected TCRs against specific peptide targets are prioritized into Immatics’ ACT pipeline for translational development into safe and effective T-cell therapeutics under ACTengine® (Adoptive Cellular Therapy with autologous engineered T cells) program. Under the first three ACTengine® programs (IMA201,202,203), we developed 3 unique T-cell products each expressing a transgenic TCR targeted against its own respective proprietary novel HLA-A*02:01 restricted tumor antigen.

**Results**

The manufacturing of ACTengine® products is based on a short and robust process with a “turnaround” time of 23-30 days from leukapheresis collection to released product, which includes a 14-day compendial sterility testing. Overall manufacturing scheme involves activation of T cells, followed by transduction with the viral vector expressing the respective TCRs, and expansion in the presence of cytokines. The ACTengine® products IMA201 and IMA202 generated for process qualification GMP runs consistently consisted of 20-80% (Avg-50%) transduced CD8+ T cells that rapidly expanded within a week’s time to meet clinical doses. Both ACTengine® IMA201 and IMA202 products comprised a substantial proportion of naïve and central memory T cells, known to be associated with long-time persistence in vivo. Further, these T-cell products demonstrated potent anti-tumor response in vitro as evaluated by cytokine release and cytotoxicity assays. Continued process improvement past the first 2 programs has led to further shortening of the expansion phase for IMA203 manufacturing to 3-4 days post-transduction compared to 5-8 days for IMA201 & IMA202. Consequently, IMA203 T cell products exhibit further improved phenotype and functionality. Taken together, all three ACTengine® products are best-in-class product candidates for cancer ACT awaiting clinical proof of concept. Phase I clinical studies of ACTengine® IMA201 and IMA202 are currently underway, in collaboration with MD Anderson Cancer Center; the ACTengine® IMA203 trial is expected to start enrolling patients in Q1, 2019.

#### P247 Potent ex vivo expanded, human CD34+ cord blood-derived natural killer cells for glioblastoma immunotherapy

##### Lin Kang, PhD, Shuyang He, William van der Touw, Bhavani Stout, Valentina Rousseva, Rathna Ravishankar, Robert Hariri, Xiaokui Zhang

###### Celularity, Warren, NJ, USA

####### **Correspondence:** Xiaokui Zhang (xiaokui.zhang@celularity.com)

**Background**

Glioblastoma Multiforme (GBM) is the most aggressive brain malignancy in adults, where the 5-year survival rate is less than 10%. Novel therapies are urgently needed. Celularity, Inc. is developing PNK-007, a culture-expanded NK cell population derived from human umbilical cord blood (UCB) hematopoietic stem/progenitor cells, for treatment of hematological malignancy and solid tumors including GBM.

**Methods**

UCB CD34+ cells were cultivated in the presence of cytokines including thrombopoietin, SCF, Flt3 ligand, IL-7, IL-15 and IL-2 for 35 days to generate PNK-007. Flow cytometry was used to evaluate the phenotypic characteristics of PNK-007. Cytotoxicity of PNK-007 against GBM cell lines was assessed by a 4h PKH26/TO-PRO-3 FACS based assay. The supernatants were collected from co-culturing PNK-007 with GBM cell lines for 24h and subjected to analysis of secreted cytokines. To identify killing mechanisms, blocking antibodies or perforin inhibitors were employed in cytotoxicity assay. The U-87MG orthotopic NSG mouse model was used for PNK-007 in vivo efficacy study.

**Results**

Multiple GBM cell lines were assessed for susceptibility to PNK-007-mediated cytotoxicity in vitro. In 4h cytotoxicity assay at an E:T ratio of 10:1, PNK-007 (n=6 donors) exhibited 59.4%±1.5%, 47.6%±10.5%, 37.7%±12.3%, and 8.5%±3.9% cytotoxicity against U-251, LN-18, U-87MG, and U-118MG cells, respectively. Increased production of IFN-γ, GM-CSF, and TNF-α was observed in the supernatants of PNK-007 co-cultured with GBM cell lines compared with those of PNK-007 alone or tumor cells alone. By using blocking antibodies and/or perforin inhibitor (Concanamycin A), we have identified that Perforin or TRAIL, or a combination of them; NKG2D or DNAM-1, or a combination of them; played an important role in PNK-007-mediated cytotoxicity. PNK-007 in vivo anti-GBM activity was assessed in a U-87MG orthotopic NSG model. 1E4 luciferase-expressing U-87MG cells were stereotactically injected into the cranium of NSG mice at Day0. Single dosing of 5E5 PNK-007 at Day14 or repeated dosing of 5E5 PNK-007 at Day14 and Day21 by intracranial injection (IC) significantly reduced Bioluminescence Imaging (BLI) compared with the PBS control (P<0.01 by T-test). Furthermore, PNK-007 with two repeated IC doses significantly reduced BLI compared with single IC dose (P<0.05 by T-test).

**Conclusions**

The results demonstrated that PNK-007 exhibited in vitro cytotoxicity against GBM cell lines and cytokine secretion activity following exposure to tumor cells. In vivo efficacy studies further demonstrated anti-tumor activity of PNK-007 in a U-87MG orthotopic NSG model. Taken together, our data support the application of PNK-007 for the development of an allogeneic adoptive immunotherapeutic for GBM patients.

#### P248 IL-2, IL-15 and IL-21 expanded tumor-infiltrating lymphocytes (TIL) for the treatment of patients with solid cancer

##### Dragan Kiselicki^1^, Julia Karbach^1^, Martin Rao, PhD^2^, Ernest Dodoo^2^, Evgueni Sinelnikov^3^, Akin Atmaca^1^, Markus Maeurer^2^, Elke Jaeger^1^

###### ^1^Krankenhaus Nordwest, Frankfurt, Germany; ^2^Champalimaud Foundation, Lisboa, Portugal; ^3^Zellwerk GmbH, Oberkrämer, Germany

####### **Correspondence:** Elke Jaeger (ej200161@aol.com)

**Background**

Checkpoint inhibitors and cellular therapies for patients with solid cancers provide new immunological treatment options

**Methods**

TIL were expanded from primary (P) or metastatic lesions (ML) in medium containing IL-2, IL-15 and IL-21 from 2 patients with pancreatic cancer (PDAC), 1 patient with glioblastoma (GB), 1 patient with fibrosarcoma (FS) and 1 patient with uveal melanoma (UVM). The GB patient received 2 subsequently clinical infusions from the same pre- expansion culture. 1 of the PDAC patients received 3 clinical TIL infusions also from the identical pre-expansion culture. The other patients received a single TIL dose with 2x10e9 TIL. All patients received preconditioning treatment with a single cyclophosphamide dose (60mg/kg) followed by up to 5x IL-2 infusions (600,000 IU/kg and 60,000 IU/kg in the GB patient). Immunophenotyping of TIL was performed by flow cytometry. Functional assays included IFN-γ production and CD107 induction. T-cell reactivity against autologous tumor cells was tested by standard Cr51 assay and IFN-γ production. Tumor lesions underwent whole exome sequencing. Patients were clinically evaluated according to RECIST criteria. Immune responses were analyzed by flow cytometry, including Th1/Th2/Th17 subsets, T-cell maturation and differentiation defined by CD45RA/CCR7, LAG-3, PD1, TCR analysis, TCR sequencing and recognition of synthetic peptide mutation-specific T-cell responses.

**Results**

TIL were reliably expanded using IL-2, IL-15 and IL-21 for 5/5 patients resulting in 8 individual TIL doses for i.v. infusion. TIL recognized the (i) autologous tumor cells defined by IFN-γ production and cytotoxicity, resided in the memory T-cell subset, (ii) exhibited a restricted TCR repertoire, (iii) strongly expressed CXCR3, reflecting tissue homing capacity and (iv) recognized individual tumor mutations presented as synthetic peptides. TIL infusion to a single patient with GB resulted in necrosis and complete tumor remission. More detailed analysis of resected stable or regressing lung metastatic lesions from a patient with pancreatic cancer who received 3 TIL infusions revealed a diverse yet restricted T-cell repertoire with preferential expansion of individual TCR VB families with a Th1/Th1* phenotype pattern suggesting a focused, local immune repertoire directed against metastatic tumor lesions (Figure 1).

**Conclusions**

IL-2, IL-15 and IL-15 expanded TIL from primary solid cancer and metastatic lesions promotes the cultivation of a focused T-cell product directed against the patient’s autologous tumor cells and offers a viable treatment modality for patients with solid cancer. .

**Ethics Approval**

Written informed consent has been obtained.

**Consent**

Written informed consent was obtained from the patients for publication of this abstract and any accompanying images. A copy of the written consent is available for review by the Editor of this Journal.

**Fig. 1 (abstract P248).**
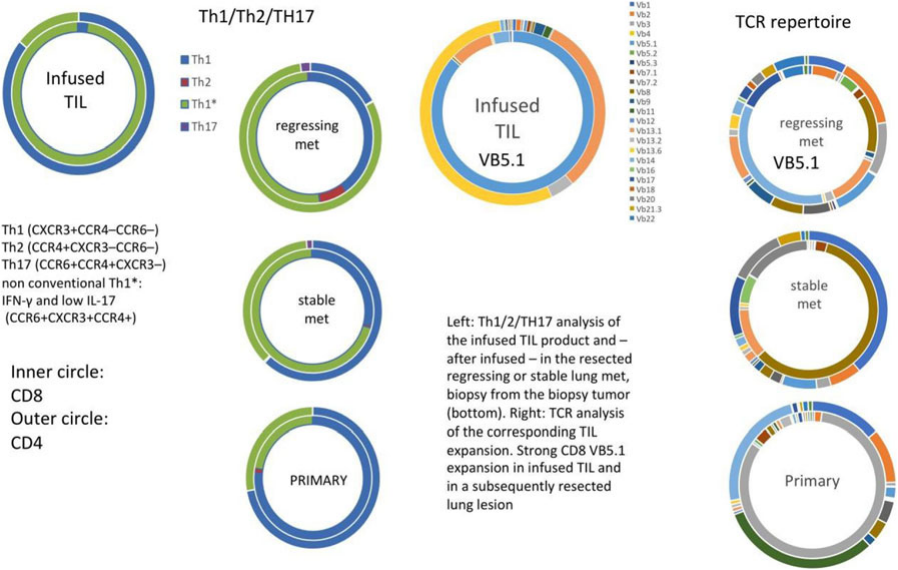
See text for description.

#### P249 Glypican-1 (GPC-1) specific CAR-T cells eradicate established solid tumor without adverse effects and synergize with anti-PD1 antibody therapies

##### Daiki Kato, DVM^1^, Tomonori Yaguchi, MD, PhD^2^, Kenji Morii, MS^2^, Satoshi Serada^3^, Tetsuji Naka^3^, Takayuki Nakagawa^1^, Ryohei Nishimura^1^, Yutaka Kawakami, MD, PhD^2^

###### ^1^The University of Tokyo, Tokyo, Japan; ^2^Keio University School of Medicine, Tokyo, Japan; ^3^National Institute of Biomedical Innovat, Osaka, Japan

####### **Correspondence:** Yutaka Kawakami (yutakawa@keio.jp)

**Background**

Chimeric antigen receptor transduced T cells (CAR-T) targeting CD19 are effective for B cell malignancies.

Although clinical trials of CAR-T therapies for patients with solid tumor have been challenged, these trials have not been successful due to problems including insufficient activities in tumor tissues, on-target/off-tumor lethal toxicities and antigen-loss. We have previously shown that glypican-1 (GPC-1) was preferentially overexpressed on squamous cell carcinoma, and generated anti-GPC-1 human CAR-T cells (hGPC1-CART) using our anti-GPC-1 Ab which recognizes both human and murine GPC-1. Although strong anti-tumor effects of the hGPC1-CART were shown in a xenograft model implanted with GPC-1+ human cancer cells, this model was not sufficient to evaluate adverse effects of CART due to severe xenoGVHD and to evaluate induction of CTL against endogenous tumor antigens, which may be important for augmenting anti-tumor effects and overcoming antigen-loss problem in solid tumors. Therefore, in this study, we generated anti-GPC-1 “murine” CAR-T cells (mGPC1-CART) using the same anti-GPC-1 Ab used for hGPC1-CART, and evaluated their characteristics in syngeneic mouse models.

**Methods**

C57BL/6 mice bearing murine GPC-1 transduced MC38 (MC38-mGPC1) or MCA205 (MCA205-mGPC1) were treated with mGPC1-CART, and antitumor effects, adverse effects, induction of endogenous tumor antigen specific T cells, and combination with anti-PD-1 Ab were evaluated.

**Results**

Intravenous administration of mGPC1-CART showed strong anti-tumor effects against GPC-1 transduced murine tumors in vivo without any overt adverse effects. Complete eradication of tumors was observed in the MCA205-mGPC1 model. No obvious damage or CAR-T cell infiltration was observed in evaluated normal tissues of the treated mice by immunohistochemical analyses. Furthermore, CTL responses against endogenous tumor antigens were enhanced by mGPC1-CART, indicating antigen spreading occurred. Since both tumor infiltrating mGPC1- CART and endogenous CD8+ T cells expressed PD-1, we also performed a combination therapy of mGPC1-CART and anti-PD-1 Ab in the MC38-mGPC1 model, and synergistic anti-tumor activities were shown without adverse effects.

**Conclusions**

These results indicate that GPC-1 specific CAR-T adoptive cell therapy in combination with anti-PD-1 Ab may be an attractive immunotherapy for solid cancers.

#### P250 Shorter ex vivo expansion of Th17 cells mediates potent anti-tumor regression in melanoma

##### Hannah Knochelmann, Michelle Nelson, PhD, Jacob Bowers, Megan Wyatt, MS, Aubrey Smith, Connor Dwyer, PhD, Chrystal Paulos, PhD

###### Medical University of South Carolina, Charleston, SC, USA

####### **Correspondence:** Hannah Knochelmann (knochelm@musc.edu)

**Background**

Adoptive T cell transfer therapy mediates potent immunity in patients with bulky metastatic malignancies but proves difficult to translate clinically due to production costs, time, and labor required to generate personalized T cell products. Though several CAR-T cell preparations were recently FDA approved, patients indicated for these therapies are at risk of insurance coverage denial due to the large costs of T cell manufacturing. As a result, methods of reducing production costs by generating T cells with potent antitumor properties more quickly are in high demand.

**Methods**

We proposed a method of shortened ex vivo expansion using Th17 cells to treat melanoma using the TRP-1 transgenic mouse model in which CD4+ T cells express a TCR that recognizes tyrosinase-related protein 1 on melanoma. Naïve CD4+ T cells were polarized to secrete IL-17, and infused into mice with B16F10 melanoma after a nonmyeloablative total body irradiation (5 Gy) preparative regimen. Antitumor efficacy of Th17 cells was studied kinetically over ex vivo expansion spanning 12 hours to 14-days. Functionality and biology of T cells was determined using flow cytometry, and serum cytokines were measured with multiplex array.

**Results**

We found that Th17 cells expanded only four days ex vivo can eradicate tumors even when only few cells (~200K) are infused into the animal. These day-4 cells mediate more potent antitumor responses than greater numbers (>25X more) of Th17 cells expanded up to two weeks. In contrast to long-term expanded cells, day-4 Th17 cells 1) express peak levels of IL-2Ralphaα and costimulatory molecules (CD28, OX40, ICOS), 2) persist at greater fold once infused in the animal, 3) induce significantly increased production of IL-6, IL-17, and GM-CSF within the tumor- bearing host, and 4) provide long-lived protection against tumor recurrence.

**Conclusions**

Our findings indicate that day-4 Th17 cells regress large melanoma tumors even in very low number. Four days after activation, Th17 cells are highly activated and induce a robust inflammatory response with the host compared to Th17 cells expanded long-term. These results highlight that transferring T cells only 4-days after activation, despite a lower yield than long-term expansion, can potentially improve efficacy, reduce expense, and improve accessibility of adoptive cell therapy to patients clinically.

**Ethics Approval**

Animal studies were approved by the IACUC of the MUSC Animal Resource Center (number 3039).

#### P251 Tumor-infiltrating lymphocytes (TIL) for the adoptive treatment of patients with pancreatic cancer

##### Joana Lerias, PhD^1^, Georgia Paraschoudi, MSc^1^, Martin Rao, PhD^1^, Nuno Couto^1^, Davide Valentini^1^, Mireia Castillo-Martin^1^, Antonio Beltran^1^, Evgueni Sinelnikov^2^, Hans Hofmeister^2^, Ana Vieira^1^, Javier Martin-Fernandez^1^, Andreia Maia^1^, Dário Ligeiro^3^, Carlos Carvalho^1^, Dragan Kiselicki^4^, Akin Atmaca^4^, Julia Karbach^4^, Elke Jäger^4^, Markus Maeurer^1^, Joana Lerias, PhD^1^

###### ^1^Champalimaud Foundation, Lisbon, Portugal; ^2^Zellwerk, Berlin, Germany; ^3^Centro de Sangue e Transplantação de Lisboa, IPST, Lisbon, Portugal; ^4^Krankenhaus Nordwest, Frankfurt, Germany

####### **Correspondence:** Markus Maeurer (markus.maeurer@fundacaochampalimaud.org)

**Background**

New treatment options are needed to offer the best suitable therapy for patients with pancreatic cancer. We studied in detail the quality and quantity of cellular immune response of a 65-year old female patient diagnosed with pancreatic ductal adenocarcinoma (PDAC) and stage IV peritoneal metastasis, who received a single dose of 60mg/kg cyclophosphamide, followed by 2x10e9 TIL at day 2, with sequent 5 doses of IL-2 (600,000 IL-2/kg). TIL were obtained from metastatic lesion and could be compared to TIL from the primary lesion as well as to T-cells obtained from a colon biopsy several weeks after TIL infusion.

**Methods**

Fresh tumor from primary (P) and peritoneal metastasis (PM) cancer lesions and colon tissue (C) were cultured in medium containing IL-2, IL-15 and IL-21. Immunophenotyping of TILs and peripheral blood mononuclear cells (PBMCs) was performed by high-content flow cytometry. Tumor exome sequencing was performed in primary and metastatic lesions, CD4+ and CD8+ TIL, as well as PBMCs were analyzed for TCR composition by CDR3 length analysis and tested for recognition of individual tumor mutations (represented by synthetic peptides) by IFN-γ production.

**Results**

P-TILs and PM-TILs were mainly effector memory (TEM), PBMCs were mostly comprised of central memory CD4+ T-cells (TCM) prior to T-cell therapy – as well as after immune-constitution. Comparison of TCR Vβ repertoire between P- and PM-TILs (86% Vβ13.1 in CD8+ P-TILs) showed a greater diversity in PM-TILs. 53/146 mutations were recognized in TIL from the primary lesion, 6/146 mutations in TIL from the metastatic lesion and 25/146 private antigens from T-cells expanded from colon biopsy after TIL infusion defined by IFN-gamma production. 60% of CD8+ PM-TILs tested CCR9 positive. Antigen-specific IFN-γ production in TIL from primary and metastatic cancer lesion showed strong responses to the melanoma-associated antigen Melan-A/MART-1 (AAGIGILTV) with high homology to common gram-negative bacterial species suggesting molecular mimicry (Figure 1).

**Conclusions**

The detailed molecular and functional examination of TIL from primary and metastatic pancreatic cancer lesions shows that TIL harvested from primary tumor lesion recognize a broad repertoire of private mutant target antigens, some which were shared among TIL from the metastatic lesion. TIL harvested from primary lesions may be used for later tumor recurrences – dependent on the mutational diversity. TIL recognition pattern may be driven by molecular mimicry for common bacterial species present in the intestine.

**Ethics Approval**

This study was approved by the Champalimaud Foundation Ethics Committee and some individuals patient signed as well the informed consent at the KHNW, Frankfurt, Germany.

**Consent**

Written informed consent was obtained from the patient for publication of this abstract and any accompanying images.

**Fig. 1 (abstract P251).**
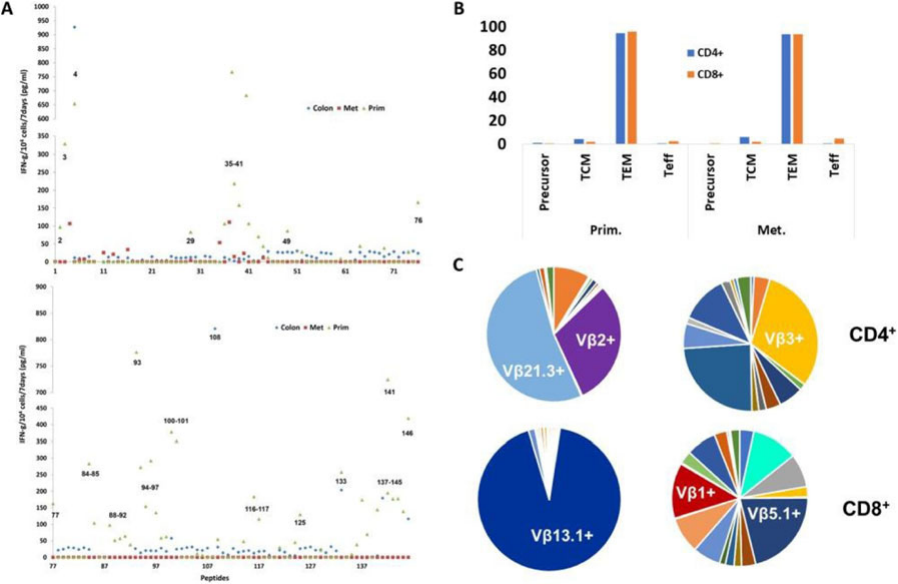
Characterization TILs derived from pancreatic tumor

#### P252 Fast quality gauging of tumor-infiltrating lymphocytes (TIL) for the treatment of patients with solid cancer

##### Georgia Paraschoudi, MSc in Cell and Molecular Biology, Sweden^1^, Joana Lérias^1^, Martin Rao, PhD^1^, Mireia Castillo-Martin^1^, Antonio Beltran^1^, Evgueni Sinelnikov^2^, Hans Hofmeister^2^, Javier Martin-Fernandez^1^, Andreia Maia^1^, Dário Ligeiro^3^, Carlos Carvalho^1^, Dodoo Ernest^4^, Dragan Kiselicki^4^, Julia Karbach^4^, Elke Jäger^4^, Ana Vieira^1^, Georgia Paraschoudi, MSc in Cell and Molecular Biology, Sweden^1^

###### ^1^Champalimaud Foundation, Lisbon, Portugal; ^2^Zellwerk, Oberkrämer, Germany; ^3^Centro de Sangue e Transplantação de Lisboa, IPST, Lisbon, Portugal; ^4^Krankenhaus Nordwest, Frankfurt, Germany

####### **Correspondence:** Georgia Paraschoudi (markus.maeurer@fundacaochampalimaud.org)

**Background**

Individually tailored therapy using Tumor- infiltrating T-cells (TILs) represents a viable treatment option for patients with advanced solid cancer.

**Methods**

Freshly harvested tumor tissue from 12 patients was tested for TIL expansion: one patient with glioblastoma (2 TIL expansions), one patient with fibrosarcoma, one with uveal melanoma, one patient with esophagus cancer, one patient with a benign lipoma, and 6 patients with pancreatic cancer (with 5 TIL expansions for an individual case harvested from different tumor sites) targeting between 1 to 2x 10e9 TILs for a single infusion. TIL were further characterized by phenotypic analysis by flow cytometry including CD3, CD4, CD8, CD45RA, CCR7, CXCR3, CCR4, CCR6, CCR9, CD103, LAG-3, CD57, HLA-DR and PD-1 and Tregs (CD4+CD25highCD127neg). The molecular diversity of TIL was gauged by TCR CDR3 analysis in sorted CD3+ and CD4+ TIL. Immunohistochemistry included detection of immune cells and NY-ESO-1 (Figure 1). Prior to testing of individual target mutant epitopes, identified by tumor exome sequencing, T-cell recognition was gauged using a broad panel of antigens associated with common viral pathogens (e.g. CMV, EBV, Flu), tumor–associated antigens (e.g. NY-ESO- 1, Melan-A/MART-1, survivin, mesothelin). Wildtype, as well as the most common KRAS mutations, wildtype MUC4 and MUC16 and a broad array of MUC4 and MUC16 mutations were tested for recognition by IFN-γ production (Figure 2).

**Results**

TIL could be reliably expanded using IL-2, IL-15 and IL-21: TIL expansion could be achieved in 12/12 patients with different cancer histologies resulting in 17 individual TIL preparations with an oligoclonal TCR repertoire and a strong CXCR3 expression in both CD4+ and CD8+ TIL enabling access to tissue. A fast orientation of TIL recognizing common targets, including common pathogens, non-mutant tumor associated antigens, as well as mutant tumor driver mutations (e.g. KRAS) aids to define very fast the quality and nature of individual TIL preparations. Strong IFN-production could be identified in individual TIL preparations against single KRAS mutants or against individual TAAs, e.g. NY-ESO-1 epitopes that was also associated with antigen protein expression in the matching tumor lesion.

**Conclusions**

IL-2, IL-15 and IL-21 expanded TILs from primary solid cancer or metastatic lesions lead to expansion of a focused T-cell product even from small biopsy lesions. ‘Fast’ functional screening of TIL for common TAAs and frequent tumor mutations may aid to select very fast individual TIL preparations for adoptive therapy or present a starting point to further expand T-cells with antigen-specific TCRs directed against defined target antigens.

**Ethics Approval**

This study was approved by the Champalimaud Foundation Ethics Committee and some individuals patient signed as well the informed consent at the KHNW, Frankfurt, Germany.

**Consent**

Written informed consent was obtained from the patient for publication of this abstract and any accompanying images.

**Fig. 1 (abstract P252).**
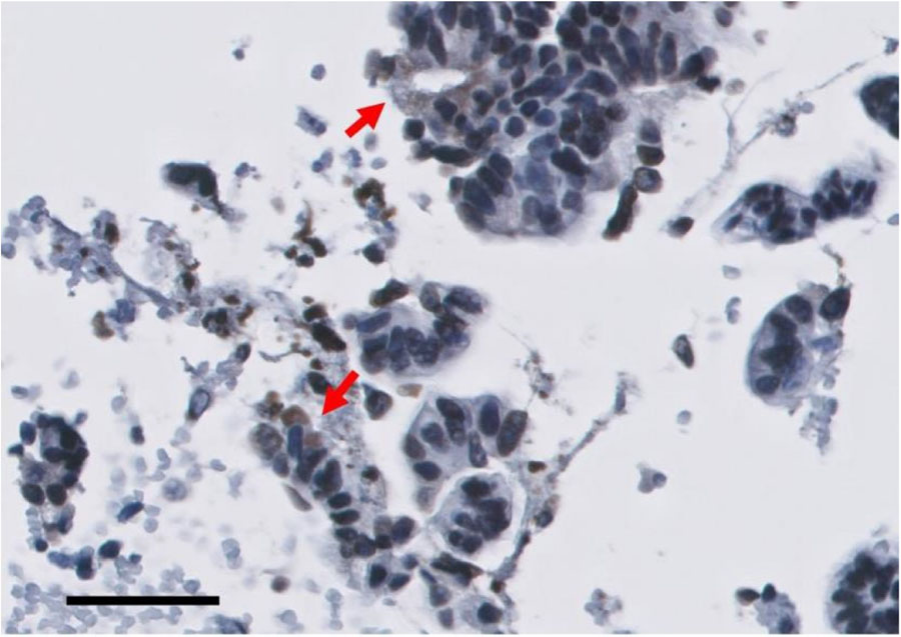
Immunohistochemical analysis of NYESO-1

**Fig. 2 (abstract P252).**
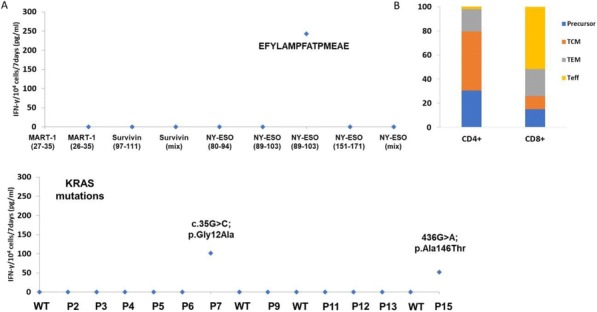
Characterization of TILs derived pancreatic tumor

#### P253 B cell structural diversity in early cultured tumor-infiltrating lymphocytes

##### Dario Ligeiro, PhD^1^, Hélder Trindade^1^, Joana Lérias^2^, Georgia Paraschoudi, MSc in Cell and Molecular Biology, Sweden^2^, Andreia Maia^2^, Catarina de Oliveira^2^, Javier Martin-Fernandez^2^, Martin Rao, PhD^2^, Carlos Carvalho, MD^2^, Mireia Castillo-Martin^2^, Markus Maeurer^2^

###### ^1^Centro de Sangue e Transplantação de Lisboa, IPST, Lisbon, Portugal; ^2^Champalimaud Foundation, Lisbon, Portugal

####### **Correspondence:** Dario Ligeiro (dario@ipst.min-saude.pt)

**Background**

The tumor-infiltrating B cells (TIB) role in anti-cancer immune responses is under debate with reports showing an association of B cell densities in tumor with either higher recurrence rates or a more favorable outcome. B-cells may serve as antigen presenting cells and producers of cytokines, suggesting both pro- and antitumor effects. The regulatory mechanisms and cell type of TIB in the tumor immune landscape, as well as their antigen-specificity remains largely unknown. Here we classify the B-cell repertoire diversity in early cultures of tumor infiltrating lymphocytes (TILs) and report their antigen-specificity for individual patients.

**Methods**

Freshly harvested tumor tissues (colorectal and pancreatic cancer) were procured for TIL expansion using IL-2, IL-15 and IL-21. Immunohistology with CD3 and CD20 mAbs was used to identify TLS (tertiary lymphoid structures). Flow cytometric analysis was performed with anti-CD19, CD20, CD27, IgM, CD5, CD3, CD4, and CD8. Diversity of immunoglobulin heavy chain (IGH) transcripts was evaluated with a CDR3 spectratyping assay for heavy chain variable segments (IGHV) IGHV1-6 families and common primers specific for IGHM and IGHG gene segments. TIB diversity was profiled based on number and distribution of in-frame peaks acquired by fluorescent capillary electrophoresis. B-cell specificity was tested using EBV-immortalization and testing wild type, as well as mutant target epitopes on streptavidin-biotin scaffold, followed by IgG detection with standard ELISA.

**Results**

TLS, containing CD20+ B-cells, can be identified in the tumor periphery of colorectal or pancreatic cancer specimens. B-cells can be identified up to 3% in early TIL cultures that are accessible for BCR receptor analysis, which showed IgM and IgG transcripts from most families of IGHV gene segments (Figure 1). The clonotype repertoire of TIB was found skewed with a predominance of oligo and monoclonal profiles; however, some TIL cultures did show significant structural diversity even for IgG BCR transcripts. B-cells could be immortalized and showed reactivity to mutant, but not to wildtype target antigens in individual cancer specimens.

**Conclusions**

B-cells constitute a set of viable immune cells with a diverse Ig repertoire in early cultured TIL. Detection of IgG shows that Ig switching took place and the recognition of mutant epitopes suggests antigen-driven Ig affinity maturation. Such mutant – reactive B-cells may augment tumor-specific T-cell responses, yet may also be tumor promoting, depending on the cytokine production pattern. Identification of individual IgG transcripts targeting mutant cancer targets may provide blueprints for the tailored therapy of cancer targeting individual cancer mutations.

**Ethics Approval**

This study was approved by Champalimaud Foundation Ethics Committee.

**Consent**

Written informed consent was obtained from the patient for publication of this abstract and any accompanying images. A copy of the written consent is available for review by the Editor of this Journal.

**Fig. 1 (abstract P253).**
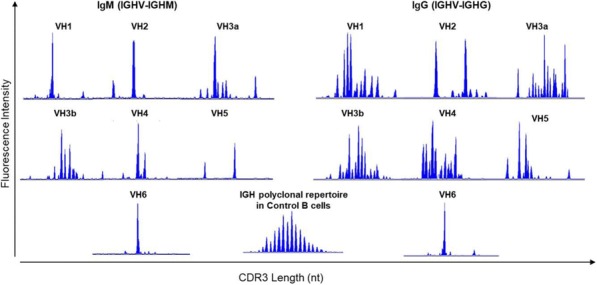
TIB repertoire in early-cultured TILs

#### P254 Natural Killer cells propagated under pressurized culture conditions show enhanced tumor killing activity

##### James Lim, Ann Lu, Nick Wilson, Albert Wong

###### Xcellbio, San Francisco, CA, USA

####### **Correspondence:** James Lim (james@xcellbio.com)

**Background**

Novel cell-based immunotherapies are in advanced clinical trials, transforming the way we treat cancers. These therapies, however, are currently limited to blood-based cancers. Critical challenges remain in treating solid tumors, where hypoxic and high pressure microenvironments can limit the efficacy of current cell-based therapies. Recent research have shown that T-cells propagated under normoxic conditions (21% O2) have decreased cytolytic activity and reduced tumor-killing rates than cells grown under hypoxic conditions [1]. This observation would indicate that improvements in cell culturing and manufacturing processes could generate therapeutically-superior cell products through the simple modulation of oxygen and pressure control. To test this hypothesis we examined the effects of hypoxia and high pressure conditions on another therapeutic lymphocyte, donor-derived Natural Killer (NK) cells.

**Methods**

NK cells were enriched using magnetic-bead based separation from the peripheral blood of 12 healthy donors. NK cells were expanded for 1 week under varying oxygen concentrations (1%, 5%, 15%, 21%) and pressurized conditions mimicking the arterial vasculature pressure (~2PSI). We performed FACS analysis against NK markers (CD16 and CD56), and against mediators of cell cytolysis, perforin and granzymeB. mRNAseq was performed on NK datasets at time of collection and after 7 days of expansion. Differential gene expression and pathway analysis was performed to compare conventional culture conditions (21% O2, 0 PSI) against conditions mimicking the arterial vasculature (15% O2 and 2PSI) and bone marrow environments (5% O2 and 2PSI). NK-Tumor cell killing assays were performed using an electrical impedance-based system against a prostate cancer cell line (DU145).

**Results**

We report that NK cells propagated under low oxygen and pressurized conditions show comparable expansion rates compared to conventional culture conditions. However, NK cells grown under conditions mimicking the bone marrow and arterial vasculature systems exhibited strong expression of both CD16 and CD56 markers. In addition, these NK cells showed elevated levels of both granzyme B and perforin. NK-tumor cell killing assays revealed superior cell-killing abilities of these NK cells compared to conventionally grown NK cells. mRNAseq analysis revealed both exhaustive and activated gene expression signatures for all samples, but NK cells grown under pressurized conditions (2PSI) were defined by a significant shift in their metabolic gene expression profile.

**Conclusions**

In summary, the insights generated from this study can enable advancements in cell-manufacturing processes to create effective therapies.

**References**

1. Gropper et. al., Culturing CTLs under hypoxic conditions enhances their cytolysis and improves their anti-tumor function. Cell Reports. 2017; 20, 2547-2555

#### P255 Results and perspectives from Phase 1 studies assessing the safety and clinical activity of multiple doses of a NKG2D-based CAR-T therapy, CYAD-01, in metastatic solid tumors

##### Alain Hendlisz, MD^1^, Sylvie Rottey, MD^2^, Mateusz Opyrchal, MD PhD^3^, Kunle Odunsi, MD, PhD^3^, Jean-Pascal Machiels, MD, PhD^4^, Solmaz Sahebjam, MD^5^, Leila Shaza, MD^1^, Sandrine Aspeslagh^1^, Marc Van den Eynde, MD^4^, Jean-Luc Canon, MD^6^, Javier Carrasco^6^, Ahmad Awada^1^, Eytan Breman, MSc^7^, Panagiota Sotiropoulou, PhD^7^, Sarah Snykers, PhD^7^, Nathalie Braun^7^, Caroline Lonez, PhD^7^, Anne Flament^7^, Bikash Verma, MD^7^, Frédéric Lehmann, MD^7^

###### ^1^Institut Jules Bordet, BRUSSELS, Belgium; ^2^Ghent University Hospital, Ghent, Belgium; ^3^Rosswell Park Comprehensive Cancer cente, Buffalo, NY, USA; ^4^Cliniques Universitaires Saint Luc, Brussels, Belgium; ^5^Moffitt Cancer Center, Tampa, FL, USA; ^6^Grand Hôpital de Charleroi (GHdC), Charleroi, Belgium; ^7^Celyad, Mont-St-Guibert, Belgium

####### **Correspondence:** Frédéric Lehmann (flehmann@celyad.com)

**Background**

Chimeric antigen receptor T-cell (CAR-Ts) therapies have yet to demonstrate positive results in the context of solid tumors likely because of the inability of classical CAR-Ts to infiltrate into the tumor and overcome a hostile immunosuppressive tumor microenvironment (TME). CYAD-01, a NKG2D receptor-based CAR-T, targets eight stress ligands expressed across the hematological/solid tumor divide and thus provides a unique opportunity to explore the challenges facing CAR-T cell therapy. Promising reports of objective responses in refractory AML patients by CYAD-01 as a standalone therapy provide confidence in the relevance of the target. Here, we provide an overview of our clinical observations relating to CYAD-01 in the solid tumor setting.

**Methods**

The THINK study evaluates multiple i.v. administrations of CYAD-01 with or without prior preconditioning therapy. The SHRINK trial is evaluating the CYAD-01 treatment i.v. administered concurrently to a standard-of- care (SoC) FOLFOX chemotherapy for metastatic colorectal cancer (mCRC) with the aim to favor infiltration into the immunosuppressive TME but also engraftment of the CYAD-01 cells.

**Results**

As of August 1, 2018, 14 patients with solid tumor indications (11 mCRC, 1 pancreatic and 2 ovarian) have been enrolled at three different dose-levels (DL, 3x10E8, 1x10E9 and 3x10E9 CYAD-01) without prior preconditioning in the THINK study. Over 28 injections 3 were associated with treatment-related grade 3/4 adverse events, with 1 cytokine release syndrome (CRS) grade 3 in DL-2 and 1 grade 4 CRS in DL-3. The CRS observed in the DL-3 patient was considered a dose limiting toxicity (DLT) and 5 patients recruited at the same dose showed no further evidence of severe toxicity. Two patients showed disease stabilization. A peak of MCP-1 in patient's sera correlated with CYAD-01 injections, while other pro-inflammatory cytokines were induced after the 2nd and 3rd CYAD-01 injections. The first of the 3 DL-1 patients (1x10E8) in the SHRINK study completed his full schedule of concomitant administration without any DLT occurrence.

**Conclusions**

As a standalone therapy, CYAD-01 has shown a reassuring safety profile and promising clinical activity in refractory AML but activity is not clear yet in solid tumors. Our initial attempts to boost the potency of CYAD-01 involve combining with SoC or preconditioning chemotherapy. Safety, clinical and translational research data comparing potential impact of these approaches on CYAD-01 potency will be presented, including any potential difference in terms of CYAD-01 kinetics. These studies will provide critical information to support the development of CAR-T therapy for solid tumors.

**Trial Registration**

NCT03018405, NCT03310008

**Ethics Approval**

The studies were approved by all relevant Belgian and US Institution‘s Ethics Boards and authorities.

#### P256 Evaluation of therapeutic T cell manufacture using long amplicon TCRβ immune repertoire sequencing

##### Geoffrey Lowman, PhD^1^, Lauren Miller, BS^1^, Timothy Looney, PhD^1^, Tor Espen Stav-Noraas^1^, Reidun Hartberg, PhD^2^, Hilde Almåsbak^1^, Mark Andersen, PhD^1^, Sjoerd van der Burg, PhD^2^, Elizabeth (Els) Verdegaal, PhD^2^, Noel de Miranda, PhD^2^

###### ^1^ThermoFisher Scientific, Carlsbad, CA, USA; ^2^Leiden University Medical Center, Leiden, Netherlands

####### **Correspondence:** Geoffrey Lowman (geoffrey.lowman@thermofisher.com)

**Background**

Following the demonstration of the tremendous potential of T cell therapies in blood cancers, the field is evolving rapidly with focus on commercial, cost-effective manufacture to offer therapies for larger treatment groups. There exists a need for streamlined and quality-controlled manufacturing processes, with closed-system operations and simplified workflows. We demonstrate the utility of long amplicon T cell receptor beta (TCRβ) sequencing to sample repertoire features of therapeutic T cells at various timepoints during the manufacturing process.

**Methods**

T cell populations from multiple donors are tracked in each step of the therapeutic T cell manufacture process, beginning with baseline repertoire measurement after isolation from the donor and expansion using CTS™Dynabeads™CD3/CD28 in CTS™ OpTmizer™ T Cell Expansion Serum Free Media with 5% CTS™ Immune Cell Serum Replacement. The repertoire is then examined after the viral transduction process, and final T cell product. We survey the TCRβ repertoire of therapeutic T cell populations using the OncomineTM TCR Beta – LR Assay, sequencing on the Ion Torrent S5 system, and repertoire analysis using Ion Reporter immune repertoire analysis software.

**Results**

Donor PBMC-derived T cells were isolated with high recovery (>90%) and purity (>95%) and uniformly stimulated (>95% CD25+day 3 post-activation). Activated T cells were expanded and preserved a young phenotype (CD28+CD62L+) at day 7-10. TCRβ sequencing was used to measure the initial pre-isolation T cell population revealing a diverse polyclonal repertoire (evenness = 0.76-0.88). Importantly, this clonal diversity persists and often increases post-isolation, through activation, expansion, bead removal, transduction, and in the final product (evenness = 0.90-0.96). The consistent increase in evenness with cell culture time suggests that the manufacturing process used here promotes a polyclonal (unbiased) T cell expansion.

**Conclusions**

Measurement of a therapeutic T cell repertoire provides a sequence level understanding of the diversity within a cell product. We demonstrate that repertoire sequencing can ensure a diverse repertoire of T cells are maintained during manufacture. A 48h turnaround time, from sample to analysis result allows this testing to occur at multiple timepoints in the manufacturing process using both the richness and evenness of the repertoire to track therapeutic T cell populations longitudinally. In addition, TCRβ repertoire sequencing of a therapeutic T cell product provides a rich baseline for further monitoring of the T cell repertoire after administration.

#### P257 Natural Killer and TCR gamma-delta T-cells are present in the tumor microenvironment and can be expanded for adoptive immunotherapy for epithelial cancer

##### Andreia Maia^1^, Joana Lérias^1^, Catarina de Oliveira^1^, Javier Martin-Fernandez^1^, Georgia Paraschoudi, MSc in Cell and Molecular Biology, Sweden^1^, Martin Rao, PhD^1^, Andreia Maia^1^, Dário Ligeiro^2^, Tin Htwe Thin, PhD^3^, Ana Isabel Vieira^1^, Carlos Cordon-Cardo, MD, PhD^3^, Carlos Carvalho, MD^1^, Markus Maeurer^1^

###### ^1^Champalimaud Centre for the Unknown, Lisbon, Portugal; ^2^IPST - Instituto Portugues do Sangue e T, Lisbon, Portugal; ^3^Icahn School of Medicine at Mount Sinai, New York, NY, USA

####### **Correspondence:** Andreia Maia (mireia.castillo@fundacaochampalimaud.pt)

**Background**

The immune system may control tumors, mainly through CD8+ T cells by recognition of major histocompatibility complex class I (MHC-I) molecules. Frequently, MHC-I loss occurs – which represents one of tumor immune evasion mechanisms. Natural Killer (NK) and TCR gamma-delta T-cells may still be able to recognize MHC-I negative tumor cells. NK and TCR gamma-delta T-cells, harvested from cancer lesions, can serve as immune effector cells in addition to conventional TCR alpha-beta T-cells, particularly in case of MHC-I negative tumors. The goal of this study is to evaluate MHC-I expression in different primary and metastatic tumors and to expand NK and TCR gamma-delta T-cells in sufficient numbers for adoptive therapy.

**Methods**

Paraffin-embedded tissue sections from one primary lung, four primary colon, two primary pancreas adenocarcinomas and four pancreatic adenocarcinoma metastases were analyzed by immunohistochemistry (IHC) with antibodies against CD56 (Novocastra), TCR γδ (Invitrogen) and HLA class I (Abcam). Number of NK and TCR gamma-delta T-cells/mm2 and percentage of MHC-I expression in tumor cells were recorded. From the same cases, fresh tumor tissue was cultured in medium containing IL-2, IL-15 and IL-21 to cultivate TILs, and percentage of TCR gamma-delta T-cells (CD3+ TCR γδ+) and NK cells (CD3- CD56+ CD16+) was analyzed by flow cytometry (FC). CD3+ TCR gamma-delta cells were further analyzed by TCR CDR3 analysis and NK cell receptors were genotyped.

**Results**

IHC analyses revealed presence of NK cells in all tumor samples, mostly in the peri-tumoral stroma, but also intraepithelially in a few cases (Figure 1A). The mean NK cells/mm2 was of 50.2 (range 14.1–155.7 cells/mm2). FC analyses from these samples showed that NK cells represented between 0%-27% in cytokine-expanded TILs (Figure 1E). TCR gamma-delta T-cells were observed in all tissue specimens except for the lung adenocarcinoma, showing a mean of 15.4 cells/mm2 (range 0-61.1 cells/mm2, Figure 1B). Interestingly, the tumor from which we expanded the highest percentage of TCR gamma-delta T-cells (52.3% in TILs) corresponded to a colon adenocarcinoma that showed the highest number of tissue TCR gamma-delta T-cells. TCR gamma-delta T-cells from colon adenocarcinoma specimens showed the presence of oligoclonal TCR Vdelta1+ TILs (Figure 1F). MHC-I evaluation showed consistent staining in all tumors, with absence of membranous expression in scattered tumor cells that accounted for 1%-10% of the entire tumor area (Figure 1C-D).

**Conclusions**

This study shows that NK and TCR gamma-delta T-cells are present and can reliably be expanded for functional analysis and adoptive TIL therapy.

**Ethics Approval**

This study was approved by the Champalimaud Foundation Ethics Committee

**Consent**

Written informed consent was obtained from the patient for publication of this abstract and any accompanying images. A copy of the written consent is available for review by the Editor of this journal

**Fig. 1 (abstract P257).**
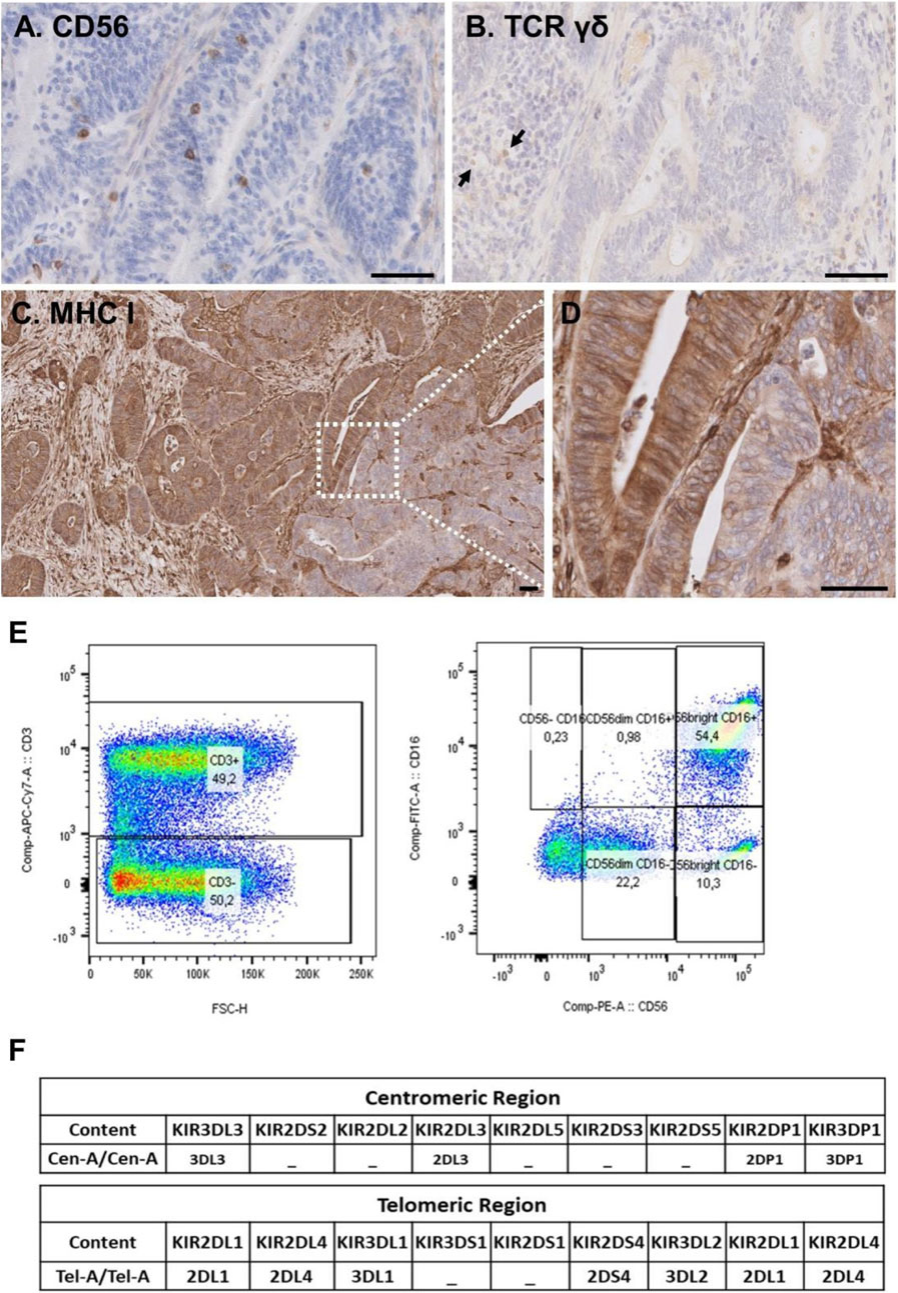
See text for description.

#### P258 sd-rxRNA to enhance NK cell activity for adoptive cell transfer

##### Melissa Maxwell, MS, James Cardia, Dingxue Yan, Ph D, Gerrit Dispersyn, PhD

###### RXi Pharmaceuticals, Marlborough, MA, USA

####### **Correspondence:** Melissa Maxwell (mmaxwell@rxipharma.com)

**Background**

NK cells are the body’s first line of defense against cancer cells. They are able to rapidly recognize and kill tumor cells without prior sensitization. Adoptive cell therapy (ACT) using NK cells shows promise against hematological cancers but the cytotoxic activity of these cells is limited by inhibitory receptors and pathways. Overexpression of such receptors has been shown to reduce NK cell-mediated cytotoxicity. Overcoming this inhibition would allow for a more potent antitumor response following ACT. We have developed a new class of stable, self-delivering RNAi compounds (sd-rxRNAs) that incorporate features of RNAi and antisense technology. sd-rxRNAs demonstrate potent activity, stability, and are rapidly and efficiently taken up by cells. We believe that sd-rxRNA targeting inhibitory receptors such as TIGIT and cbl-b may enhance the cytotoxic activity of NK cells used in ACT.

**Methods**

Freshly isolated human NK cells were isolated using negative selection and cultured in standard culture media containing IL-2. Twenty-four hours after isolation, cells were collected for transfection and the cell concentration was adjusted to ~1 x 106 cells/mL in RMPI media containing IL-2. Cells were seeded directly into 24-well plates containing chemically-optimized sd-rxRNAs ranging in final concentration from 0.125 μM to 2 μM. Taqman gene expression assays were used to determine expression levels of inhibitory receptors following the RNA to Ct 1-step protocol. In addition, cells were stained using fluorescently labeled antibodies for flow cytometry. Cytotoxic capabilities of these transfected NK cells against cancer cell lines were tested in a Real Time Cell Analysis (RTCA) assay

**Results**

Transfection with sd-rxRNA targeting inhibitory receptors resulted in consistent silencing without negative impact on NK cell viability. For example, 2 μM TIGIT sd-rxRNA results in a 90% reduction in TIGIT mRNA. The reduction is seen at least 7 days post-transfection and results in a 38% reduction in surface expression of TIGIT by flow cytometry. Similar results were obtained with cbl-b silencing. The effects of this downregulation resulted in increased cytotoxic capabilities of NK cells against cancer cell lines in an RTCA assay.

**Conclusions**

This is the first data to demonstrate the potential of sd-rxRNA to improve NK cell potency in ACT. By treating NK cells with sd-rxRNA targeting inhibitory receptors such as TIGIT ex vivo, prior to ACT, the anti-tumor response of these cells may be enhanced resulting in a more effective therapy for hematological malignancies.

#### P259 Regional delivery of chimeric antigen receptor (CAR)-engineered T cells with 4-1BB co-stimulation effectively targets TAG72-positive peritoneal ovarian tumors

##### John Murad, BS, MS, Anna Kozlowska, MSc, PhD, Maya Ramamurthy, Wen-Chung Chang, MS, Paul Yazaki, PhD, David Colcher, PhD, John Shively, PhD, Mihaela Cristea, MD, Stephen Forman, MD

###### City of Hope, Duarte, CA, USA

####### **Correspondence:** John Murad (spriceman@coh.org)

**Background**

Obstacles in developing effective Chimeric Antigen Receptor (CAR)-engineered T cell therapies for solid cancers include avoiding off-tumor on-target toxicity due to the lack of truly restricted tumor antigens, and achieving durable responses usually limited by T cell persistence, and tumor trafficking. Aberrant glycosylation of cell surface proteins on tumors represent unique targets for cell based immunotherapy. One of these targets, TAG72 is over- expressed in multiple solid tumors including epithelial ovarian cancers. Recent optimization of CAR T cell design to include intracellular co-stimulatory signaling has improved anti-tumor activity, cytokine production, and T cell persistence. Furthermore, route of T cell delivery in solid tumors seems to be important to the anti-tumor activity in some settings. Herein we evaluate TAG72 CARs with 4-1BB co-stimulation for the treatment of advanced ovarian cancers.

**Methods**

TAG72 targeting CARs with CD3-ζ stimulation and 4-1BB co-stimulation (TAG72-BBζ) were evaluated for activation and anti-tumor activity using in-vitro co-culture assays with TAG72-positive and negative ovarian cancer target cell lines and primary cancer patient samples, using flow cytometry and ELISA. Anti-tumor activity of TAG72-BBζ CARs was evaluated in-vivo by intravenous or intraperitoneal routes of administration in clinically relevant peritoneal ovarian cancer models in NSG mice.

**Results**

TAG72-CAR T cells containing a 4-1BB co-stimulatory domain demonstrated selective activation and targeting of TAG72-positive ovarian cancer cells. Furthermore, our CARs targeted TAG72-positive cancer cells obtained from ovarian cancer patient ascites in-vitro. Analysis of in-vivo therapeutic activity of TAG72-BBζ CARs in two clinically relevant ovarian peritoneal cancer models show that regional intraperitoneal, but not intravenous, delivery of TAG72-BBζ CARs exhibited more potent anti-tumor activity and extended survival in mice. Importantly, repeat administration of TAG72-BBζ CARs more effectively controlled tumor burden and extended overall survival. Tumor recurrences following CAR T cell therapy, in part due to tumor antigen heterogeneity and/or limited T cell persistence, were also observed.

**Conclusions**

TAG72-BBζ CAR T cells showed potent antigen-dependent cytotoxicity and cytokine production against multiple TAG72-positive ovarian cancer cell lines and patient-derived ovarian cancer ascites. Using in-vivo xenograft models of peritoneal ovarian tumors, regional intraperitoneal delivery of TAG72-BBζ CAR T cells significantly reduced tumor growth and extended overall survival of mice, and was further improved with repeat infusions of CAR T cells. However, antigen loss was observed in early recurring tumors, which coincided with a lack of T cell persistence. Taken together, we demonstrate efficacy with TAG72-CAR T cells for ovarian cancer, warranting further investigations as a therapeutic strategy for this disease.

**Ethics Approval**

The study was approved by the COH Institutional Review Board (IRB) and Office of Human Subjects Protection.

#### P260 Automated perfusion-based systems for research- and clinical-scale dendritic cell generation

##### Shashi Murthy, BS, PhD, Andrew Kozbial, BS, PhD, Lekhana Bhandary, BS, PhD

###### Northeastern University, Boston, MA, USA

####### **Correspondence:** Shashi Murthy (s.murthy@northeastern.edu)

**Background**

Dendritic cells (DCs) are effective vehicles for personalized therapies and there is substantial interest in leveraging their capabilities for improving patient outcomes [1,2]. However, the process of generating these DCs, typically from monocytes (MOs), in an efficient and reproducible process has proved challenging. We have developed automated closed systems, EDEN (large scale) and MicroDEN (small scale), for generating clinically relevant numbers of DCs from precursor MOs which can be sterile-transferred for downstream applications. These systems overcome many of the constraints of manual processing and are designed for the research environment as well as clinical manufacturing.

**Methods**

Enriched MOs were seeded into EDEN at a density of 206,000 MOs/cm2 using RPMI 1640 medium supplemented with 10% HI-FBS and 500 U/mL IL-4 and GM-CSF (R&D). Medium was continuously perfused and immature DCs were harvested on Day 6.Enriched MOs were seeded into MicroDEN at densities of 200k, 400k, and 600k MOs/cm2 using CellGenix DC Medium supplemented with 350 U/mL IL-4 and GM-CSF (CellGenix). Medium was continuously perfused and DCs were harvested on Day 6.

**Results**

Flow cytometry indicated that immature DCs (iDCs) from EDEN exhibited standard DC-SIGN (CD209), CD14, and CD80/83/86 (Figure 2). Antibody expression is comparable to prior results collected in MicroDEN and well plate controls. Approximately 25 million viable iDCs were harvested from EDEN for an iDC yield of 32% with the relatively low seeding density. These results indicate that EDEN can be used to generate clinically relevant numbers of iDCs in a single closed system. Flow cytometry indicated that MicroDEN generated phenotypically similar iDCs at each seeding density with an iDC yield of ~30% (Figure 3), similar to well plate controls. Allogeneic functional assays indicated that MicroDEN iDCs exhibited improved proliferative activity than well plate iDCs and generally exhibited better activity at lower seeding densities (Figure 4).

**Conclusions**

DCs generated in both EDEN and MicroDEN are phenotypically comparable and exhibit increased proliferative activity to standard manual culture. Upto 26 million DCs can be generated by MicroDEN at the highest seeding density whereas the output of EDEN is 26 million DCs at the lowest seeding density.

**Acknowledgements**

Funding from the NIH via U24 AI118665 is gratefully acknowledged.

**References**

1. Wang Z, Wu Z, Liu Y, Han W. New development in CAR-T cell therapy. J Hematol Oncol. 2017;10:53.2. Carreno BM, Magrini V, Becker-Hapak M, Kaabinejadian S, Hundal J, Petti AA, et al. A dendritic cell vaccine increases the breadth and diversity of melanoma neoantigen-specific T cells. Science. 2015;348:803-808.

**Fig. 1 (abstract P260).**
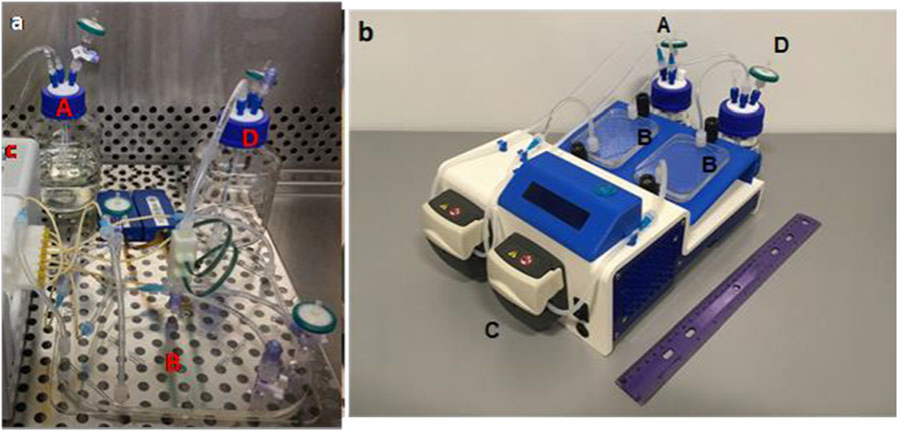
See text for description.

**Fig. 2 (abstract P260).**
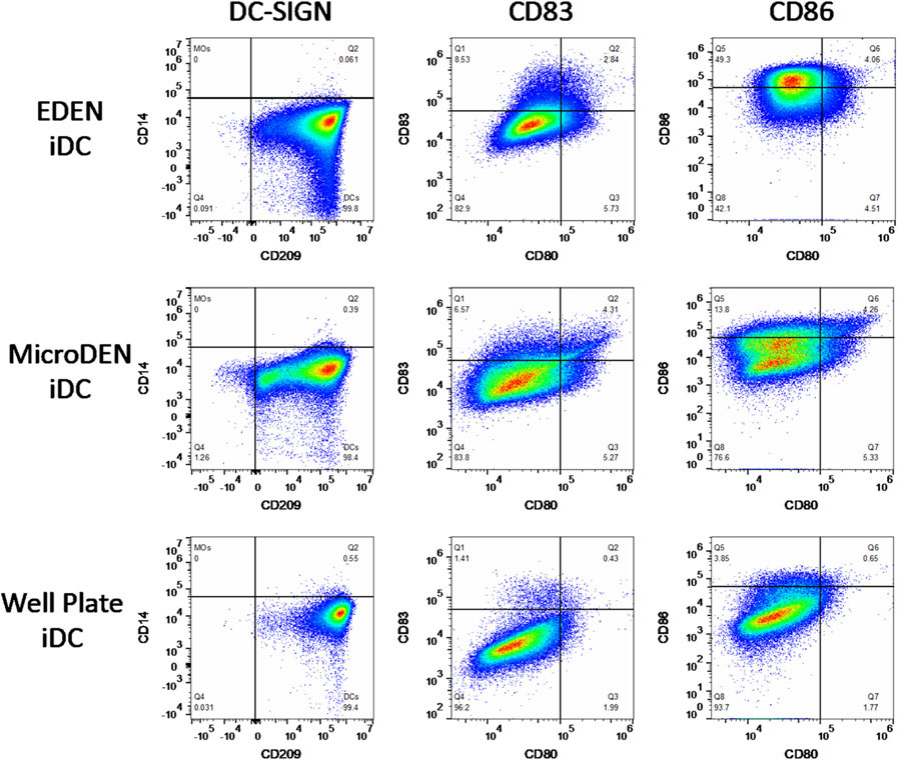
See text for description.

**Fig. 3 (abstract P260).**
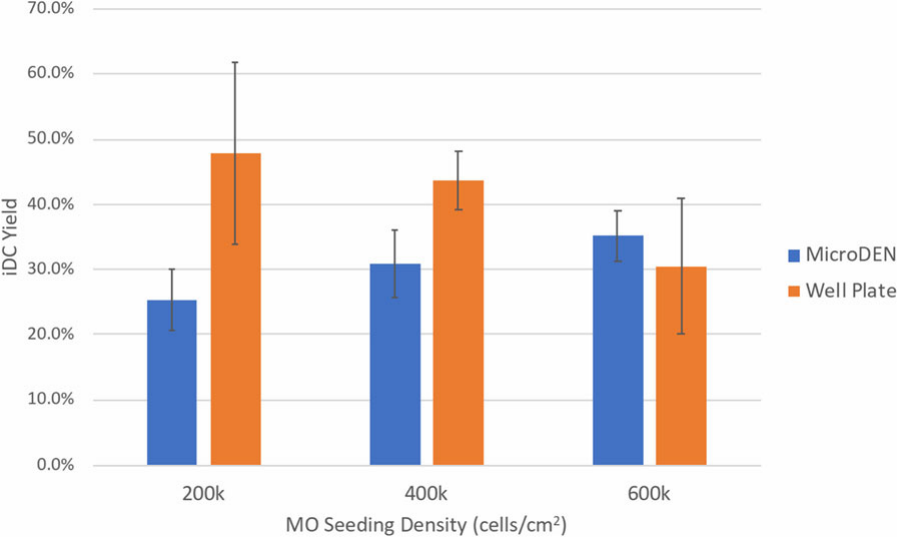
See text for description.

**Fig. 4 (abstract P260).**
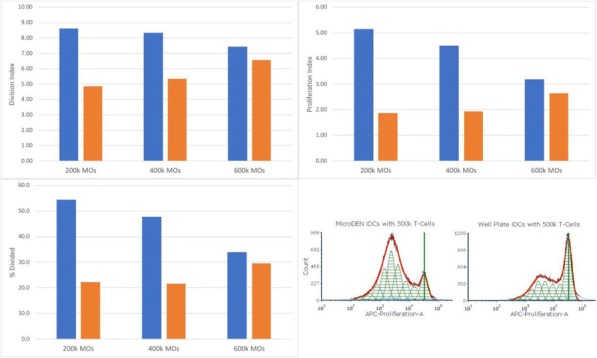
See text for description.

#### P261 Identification and pre-clinical development of tumour-reactive T-cell receptors from tumour-infiltrating lymphocytes

##### Natasha Myhill, MRes^1^, John Bridgeman, Dr^2^

###### ^1^University of Manchester, Manchester, UK; ^2^Immetacyte Ltd., Manchester, UK

####### **Correspondence:** Natasha Myhill (natasha.myhill@postgrad.manchester.ac.uk)

**Background**

Background: In the treatment of metastatic melanoma, tumour-infiltrating lymphocyte (TIL) therapy is one of the most promising immunotherapeutic options, with response rates of over 50% regularly reported. However, the tumour-reactive populations within the TIL products often remain poorly characterised, for example, whether the targets are comprised mainly of ‘neoantigens’ which are patient-specific or more common antigens shared between patients. By investigating whether T-cell receptors (TCRs) from TIL products reactive to common melanoma antigens can be utilised for treatment of multiple patients, we can better understand and shape TIL and TCR therapy to better target patient tumours.

**Methods**

Five HLA-A*02-restricted TCRs from one patient TIL sample that all recognise the shared melanoma antigen gp100 have been identified using an innovative paired TCR single cell sequencing technique. Initially, the gp100-TCRs have been cloned first into a TCR-negative Jurkat cell line for characterising their reactivity profiles using a series of flow cytometry based co-culture assays, before transduction into primary cells for assessment of tumour-killing potential. Matched and mismatched tumour lines, derived from patient tumour digest, were also characterised according to their tumour antigen expression using flow cytometry.

**Results**

Using the Jurkat cell model, results show that there are differences in the activation profile of the five TCRs, regarding their sensitivity to the gp100 index peptide when presented by T2 cells. The results also show that there is limited cross-reactivity between the TCRs, and that their activation profiles in response to HLA-A*02-expressing tumours derived from patient tumour samples reflect those seen for the gp100-index peptide. When the gp100-TCRs were cloned into primary CD8+ T-cells, intracellular cytokine staining by flow cytometry shows that all the TCRs respond to the gp100 index peptide and are capable of killing HLA-A*02 restricted tumour cells. The relative gp100 expression between the different tumours explains some of the differences in T-cell responses.

**Conclusions**

Out of the five candidate TCRs, gp100-5 exhibits the strongest activation profile when shown to the patient matched tumour line, indicating it might be the best at responding to lower levels of the antigen. These data allow us to better interrogate the overall reactivity profiles of TIL and investigate the specific shared antigen responses contained within these therapeutic cell populations.

**Acknowledgements**

I would like to acknowledge GigaGen for their involvement in the identification of the gp100-TCRs using their paired TCR single cell sequencing method.

#### P262 AIM ACT, a novel nanoparticle-based technology that generates therapeutic numbers of functional tumor- specific CD8+ T cells with central and effector phenotype in 14 days

##### Mathias Oelke, PhD^1^, Sojung Kim^1^, Juan Varela^1^, Tatsuya Yoshida^2^, Jeffrey Weber, MD, PhD^2^, Lauren Suarez^1^, Emily Lu^1^, Celine Walmacq^1^, Daniel Dembrow^1^, Daniel Bednarik^1^, Kenneth Carter, PhD^1^, Scott Carmer, MBA^1^

###### ^1^NexImmune, Gaithersburg, MD, USA;^2^New York University Medical Center, New York, NY, USA

####### **Correspondence:** Mathias Oelke (kjones@neximmune.com)

**Background**

Efficient ex-vivo generation of functional tumor-specific T cells with memory phenotype remains a significant hurdle for the broad application of adoptive cell transfer (ACT) protocols for the treatment of cancer. Genetically engineered T cells represent one option, but genetic manipulation of T cells presents significant challenges in terms of complexity and generation time. Here, we describe a novel nanoparticle-based approach for generating tumor- specific T cells at clinical grade and scale from the endogenous T cell repertoire using artificial antigen presenting cells (aAPC).

**Methods**

Our aAPC consist of a paramagnetic nanoparticle to which humanized HLA-A2-Ig dimer-molecules and anti-CD28 antibodies are covalently linked. aAPC are loaded with multiple HLA-A2 restricted peptides and used to magnetically enrich and expand tumor-specific CD8+ T cells. Using peptide loaded aAPC, a fully enclosed, semi- automated, GMP T cell expansion platform has been developed that consistently generates clinically relevant numbers of tumor-specific, central and effector memory CD8+ T cells in 14 days, providing an alternative to genetic manipulation of T cells.

**Results**

Starting from a healthy donor leukopak, CD8+ T cells were generated using an aAPC cocktail loaded with 5 HLA-A2 epitopes from AML tumor antigens WT1, PRAME and cyclin A1. On average (n>20), 1-2 x109 T cells were generated that were 90% memory T cells with about 50% central memory and 40% effector memory CD8+ T cells. AML-specific T cells were expanded 500 to >5000-fold from low frequency precursor populations. These T cells were fully functional, as demonstrated by intra-cellular cytokine analysis and tumor cell killing. The system was also used to generate Mart-1 specific T cells of the same quality from cryopreserved PBL from melanoma patients. Additional data analyzing the TCR repertoire of the expanded AML and melanoma-specific T cells will be presented.

**Conclusions**

AIM ACT is a novel nanoparticle-based T cell expansion platform for the rapid, streamlined generation of clinically-relevant numbers of tumor-specific, central and effector memory CD8+ T cells from donor and patient PBMC in 14 days. The results reported here describe a platform that will be used in a multi-institution phase I clinical trial of adoptive T cell transfer for the treatment of AML patients pre- and post-allogeneic hematopoietic stem cell transplant. The flexibility of the AIM ACT system, as demonstrated using both AML and melanoma antigens, shows the potential for clinical application in other heme and solid tumors. Additionally, the system can be used for targeting both known and neo-epitopes.

#### P263 IL13RA2 as a new target for T-cell based therapies of melanoma brain metastases

##### Maria Ramello, PhD, Ismahene Benzaid, PhD, Maritza Lienlaf, Vincent Law, Nikhil Khushalani, MD, Amod Sarnaik, MD, Inna Smalley, PhD, Keiran Smalley, PhD, Peter Forsyth, MD

###### Moffitt Cancer Center, Tampa, FL, USA

####### **Correspondence:** Maria Ramello (daniel.abatedaga@moffitt.org)

**Background**

Melanoma brain metastases (MBM) represent a common complication of advanced cutaneous melanoma, and a major unmet need. Adoptive T cell therapies using genetically modified T cells to express chimeric antigen receptors (CAR) have shown evidences of disease control in primary brain tumors. Based on the observation of immunologically relevant expression of IL13RA2 mRNA in metastatic lesions of melanoma patients, we tested the potential of this cancer/testes antigen as a target for cellular immunotherapies.

**Methods**

IL13RA2 expression was analyzed in patient-samples by flow cytometry. Activity of CAR-T cells was analyzed in vitro in co-cultures with target cells and in vivo, in a murine model of leptomeningeal disease (LMDz).

**Results**

Flow cytometry analyses confirmed the surface expression of IL13RA2 in MBM, as well as in soft tissues, in 12 out of 14 surgically resected lesions. We generated 2 novel 2nd generation CARs targeting IL13RA2 based on humanized monoclonal antibodies, which recognized the IL13RA2-expressing melanoma cell lines A375 and A375BR. Moreover, CAR-T cells exhibited a potent cytotoxic effect against Lu1205, M299 and WM1366 melanoma cell lines as measured by ACEA’s xCELLigence real-time cytotoxic assay. In order to test whether T cell function may be affected by soluble factors present in the cerebrospinal fluid (CSF), we stimulated PBMCs with anti-CD3/CD28 beads in presence or absence of CSF. We observed higher proliferation in presence of melanoma patient-derived CSF compared to IL-2-containing media, suggesting that CAR-T cells are not intrinsically inhibited by (and might benefit from) exposure to CSF. In order to study CAR-T function in vivo, we developed a murine model of LMDz, an aggressive form of progression of MBM. To induce LMDz, A375BR cells were injected into cisterna magna of NSG mice. At day 10, all mice exhibited a significant reduction in total body weight, suggesting disease progression. On day 11, 25*10^6 CAR-T cells or untransduced controls (UT) were intravenously administered. Following treatment, mice treated with IL13RA2-CAR-Ts exhibited stabilization in the body weight while those receiving UTs continued to lose weight. At day 10 post-adoptive cell transfer we found higher counts of T cells in CSF of CAR-T-treated mice compared to UT.

**Conclusions**

In summary, our results suggest that IL13RA2 is a plausible target for CAR-T-based therapy of MBM. In addition, we demonstrate that human CSF does not exert an intrinsically inhibitory effect on T cell proliferation. Our current efforts are focused on further characterizing the in vivo therapeutic efficacy of anti-IL13RA2 CAR-T cells against MBM.

**Acknowledgements**

We acknowledge Margaret Baldwin, Assistant Director, Direction of Comparative Medicine, USF, for cisterna magna injections.

**Ethics Approval**

This study was approved by USF and Chesapeake IRB approval numbers 50102 and pro00014483, respectively, and by USF IACUC R2385.

#### P264 Identification of tyrosine phosphorylation sites in CD28 domain and their role in CAR-T cell function

##### Maria Ramello, PhD, Bin Fang, PhD, John Koomen, PhD, Eric Haura, MD

###### Moffitt Cancer Center, Tampa, FL, USA

####### **Correspondence:** Maria Ramello (daniel.abatedaga@moffitt.org)

**Background**

Chimeric antigen receptors (CARs) contain an antigen-sensing ectodomain and a signaling endodomain responsible for the initiation of a phosphorylation cascade that drives T-cell activation. Numerous pathway-focused attempts to characterize CAR signaling properties have been described. However, a global system-level assessment of CAR- triggered signaling network has not been described.

**Methods**

In order to conduct an unbiased analysis of CAR-initiated signaling events, we designed a phosphoproteomic assay in which PSCA-specific CAR-T cells are stimulated with metabolically heavy-labeled pancreatic cancer cells naturally expressing PSCA. Phosphorylation events (pY and pS/T) were detected by LC-MS/MS in the co-culture extracts. Post-hoc analyses allowed us to discriminate signals corresponding to T cells, based on exclusion of heavy- labeled tumor proteins. Ingenuity Pathway Analysis™ was used to identify pathways that were significantly overrepresented among the differentially phosphorylated proteins. CARs bearing phosphomimetic or non- phosphorylatable substitutions in key Y residues were generated, to validate their functional relevance in co-culture experiments and in in vivo models of adoptive immunotherapy.

**Results**

We found 40 peptides (of 791) differentially phosphorylated between CAR-Ts and mock-transduced T cells, spanning multiple pathways. Following tumor cells recognition, 2nd generation CAR-Ts exhibited more pronounced changes in phosphorylation than 3rd generation counterparts. Interestingly, we detected phosphorylation in all four tyrosine (Y) residues contained in the CD28 cytoplasmic tail. Two of these residues (Y191/YNMN and Y209/PYAP) are well characterized in terms of their functional relevance. However, the role of Y206 and Y218 residues is poorly understood. To evaluate their biological relevance, we generated 8 different versions of PSCA- CARs, each one including a non-phosphorylatable alanine-substitution or a phosphomimetic glutamic-acid- substitution in one of the identified Y residues. Upon transduction of human T cells, we observed that alanine- substitution of Y218 and glutamic-acid-substitution of Y191 severely compromised CAR expression as well as cytokine production after co-culture with target cells. Moreover, IFNγ and IL-2 production in response to tumor recognition were reduced when Y206 and Y209 were mutated with either of both alanine or glutamic-acid residues. Most importantly, we found that Y206A and Y218A substitutions reduced the anti-tumor efficacy of PSCA-CAR-Ts in vivo.

**Conclusions**

In summary, we developed a mass spectrometry platform to assess signaling events in CAR-Ts in a co-culture system. We identified four Y residues within the CD28 domains that are phosphorylated upon CAR activation, and established their relevance for CAR expression and/or function. A deeper understanding of molecular events controlled by these phosphosites will allow us to design new CARs with enhanced functional properties.

**Acknowledgements**

We acknowledge Christopher Anermann for technical assistance.

**Ethics Approval**

This study was approved by USF’s IACUC, approval number R2385

#### P265 Inhibiting immune evasion in CNS tumors by reversing epigenetic gene silencing of chemoattractant cytokines

##### Nivedita Ratnam, PhD, Heather Sonnemann, Amber Giles, PhD

###### National Cancer Institute, Bethesda, MD, USA

####### **Correspondence:** Nivedita Ratnam (mark.gilbert@nih.gov)

**Background**

While immunotherapy may be a potential therapeutic strategy for CNS malignancies, concerns remain regarding the successful trafficking of cytotoxic immune cells into the tumor. CNS tumors have multiple strategies of immune evasion including the epigenetic silencing of chemoattractant cytokines. However, interferon gamma (IFNg) from activated T cells can induce tumor cells to express chemoattractants CXCL9 and CXCL10. In ovarian cancer models, the histone methyltransferase inhibitor, GSK126, enhanced expression of these chemokines, thereby increasing T cell trafficking to the tumor. Here, we tested if GSK126 could likewise increase T cell migration to glioma tumors.

**Methods**

Gene expression of CXCL9 and 10 by qRT-PCR was determined in human and murine gliomas cell lines after treatment with a combination of IFNg and GSK126. ELISA assays were conducted to quantify secreted chemokines under similar treatment conditions. Cell migration assays were performed using tumor condition media (TCM) from human and mouse glioma cells treated with IFNg and GSK126 using healthy donor T cells (from PBMC) or mouse splenic T cells. Expression of CXCR3, the receptor for CXCL9 and 10 on T cells was measured in response to treatment with GSK126.

**Results**

CXCL9 and CXCL10 were enhanced both at the RNA and protein level in glioma cells treated with IFNg and GSK126 compared to vehicle treatment. This corresponded to increased T cell migration to GSK126-treated TCM. Treatment with GSK126 caused an increase in CXCR3 expression as well as an upward trend in gene expression of IFNg on T cells.

**Conclusions**

Our data support that epigenetically silencing the expression of chemoattractant cytokines is a mechanism of inhibiting immune cell trafficking into primary brain tumors. Histone methyltransferase inhibitors such as GSK126 reverse this gene silencing, resulting in increased T cell migration towards tumor cells. Thus, combinatorial treatment strategies involving GSK126, could potentially increase therapeutic efficacy by inhibiting immune evasion and increasing immune cell trafficking to the tumor; providing a potential rational combination with other immunotherapy regimens under investigation for primary brain tumors including checkpoint blockade using anti- PD-1 and/or anti-CTLA-4.

#### P266 Improving T cell functionality for adoptive cell transfer therapy in metastatic colon cancer

##### Sruthi Ravindranathan, PhD^1^, Mohammad Raheel Jajja^1^, Christopher Petersen^1^, Ramireddy Bommireddy^1^, Periasamy Selvaraj^1^, Lily Yang^1^, Bassel El-rayes, MD^1^, Brianna Flynn, MS^2^

###### ^1^Emory University, Atlanta, GA, USA; ^2^Isoplexis, Branford, CT, USA

####### **Correspondence:** Sruthi Ravindranathan (ewaller@emory.edu)

**Background**

Checkpoint inhibitors have shown promise in the management of several solid cancers, however this has not been replicated in colon cancer. This poor response has been attributed to high molecular heterogeneity and low frequencies of tumor infiltrating lymphocytes. We have previously demonstrated that T cells isolated from heavily treated lymphoma patients when treated with inhibitors of PI3K delta (idelalisib) and vasoactive intestinal peptide (VIP) signaling results in decreased levels of senescent T cells and increased frequency of stem memory and central memory T cells [1]. Additionally, we have demonstrated that tumor membrane vesicles (TMVs) prepared from human tumor cells decorated with IL-12 and B7-1 (IL-12. B7-1 TMVs), stimulate expansion of tumor antigen specific T cells [2]. Thus, current study aims to combine both strategies to obtain tumor antigen specific T cells with enhanced anti-tumor activity and in-vivo persistence.

**Methods**

PBMCs were obtained from four consented colon cancer patients by Ficoll lymphocyte separation medium. Cells were then cultured for 14 days in RPMI complete media with 10% FBS, 50uM 2-mercaptoethanol, 30U/ml IL-2, anti-CD3/CD28 beads and in the presence or absence of idelalisib and/or antagonist for VIP (VIPhyb). The polyfunctionality strength index (PSI) of the expanded cells was determined via single cell analysis measured using in-depth cytokine analysis on the IsoCode Chip from Isoplexis. Fresh colon cancer tissue obtained from consented patients with metastatic colorectal cancer was implanted into immunocompromised mice NOD-SCID-gamma (NSG) and monitored for tumor growth. After sequentially transplanting in NSG mice for 3-5 passages, the tumors were isolated, lysed to generate cell membranes and incorporated with human IL-12 and B7-1 using glycosylphosphatidylinositol (GPI) anchor. The expression of IL-12 and B7-1 on the TMVs was confirmed using flow cytometric analysis.

**Results**

PBMCs from colon cancer patients expanded with VIPhyb and idelalisib resulted in enhanced PSI in CD4+ T cells (Figure 1). The elevated PSI was predominated by effector proteins associated with anti-tumor profile. Flow cytometric analysis of hIL-12. hB7-1 TMVs confirmed expression of human IL-12 and B7-1 (Figure 2).

**Conclusions**

Treating PBMCs isolated from colon cancer patients with VIPhyb and idelalisib increases the polyfunctionality strength index of CD4+ T cells. Further, if TMVs decorated with IL-12 and B7-1 are included in the culture, we expect to successfully expand tumor antigen specific T cells that can be adoptively transferred to colon cancer patients. Experiments are underway to test this hypothesis in vitro and in PDX mice with human colon cancer.

**References**

1. Petersen CT, Hassan M, Morris AB, Jeffery J, Lee K, Jagirdar N, Staton AD, Raikar SS, Spencer HT, Sulchek T, Flowers CR, Waller EK. Improving T-cell expansion and function for adoptive T-cell therapy using ex vivo treatment with PI3Kdelta inhibitors and VIP antagonists. Blood Adv. 2018;2(3):210-23.

2. Nagarajan S, Selvaraj P. Human tumor membrane vesicles modified to express glycolipid-anchored IL-12 by protein transfer induce T cell proliferation in vitro: a potential approach for local delivery of cytokines during vaccination. Vaccine. 2006;24(13):2264-74.

**Ethics Approval**

The study was approved by Emory's Institutional review board, approval number 00054023 The study was approved by Emory's Institutional Animal Care and Use Committee, protocol number 2005055

**Fig. 1 (abstract P266).**
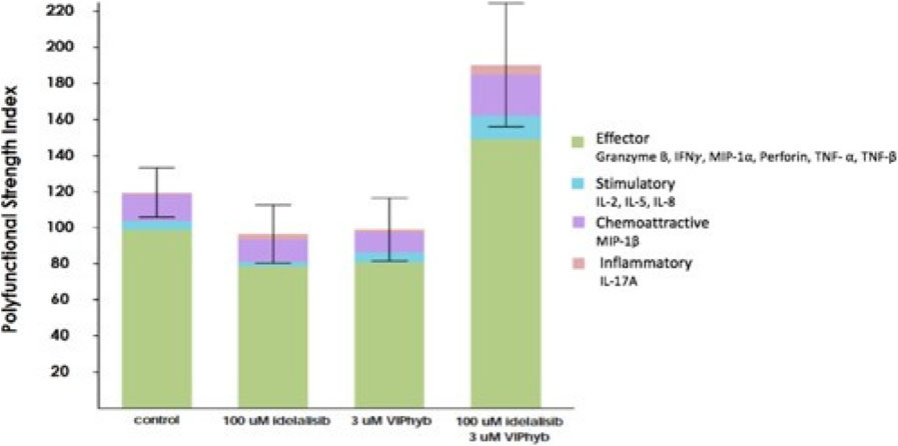
Polyfunctional strength index (PSI) of CD4+ cells

**Fig. 2 (abstract P266).**
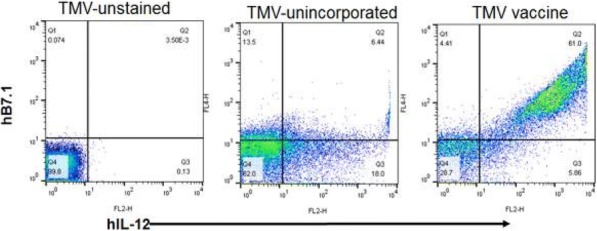
TMVs generated from PDX tumor decorated IL-12 B7-1

#### P267 Hijacked CAR19 T cells have potent activity against solid tumors

##### Paul Rennert, PhD, Fay Dufort, PhD, Lihe Su, PhD, Lan Wu, PhD, Alyssa Birt, Roy Lobb, PhD, Christine Ambrose, PhD

###### Aleta Biotherapeutics, Natick, MA, USA

####### **Correspondence:** Paul Rennert (paul.rennert@aletabio.com)

**Background**

CAR T cells targeting CD19 (CAR19s) can eradicate B cell leukemias and lymphomas. The effectiveness of CAR19s is driven by their robust expansion and persistence properties, supported by normal CD19+ B cells. Thus CD19+ B cells serve as a non-tumor dependent, self-renewing source of antigen. We re-engineer CAR19s to secrete retargeting fusion proteins (FPs) by encoding expression cassettes downstream of the CAR sequence. By hijacking CAR19s, we utilize their inherent persistence properties. By designing multi-specific FP, we directly counter the clinically critical issues of tumor heterogeneity and antigen loss.

**Methods**

A MCSV promoter-based lentiviral vector was used to express the CAR19 construct and FPs. FP expression cassettes were designed to encode the CD19 proteins extracellular domain and one or two scFv sequences, separated from the CAR sequence by a P2A cleavage site. This modular design is termed IMPACTtm (Integrated Modular Proteins for Adoptive Cell Therapy). Primary T cells were transduced with an IMPACT- lentiviral vector encoding a CD19-anti-Her2 FP. FP secretion was measured across multiple donors. Next, an anti-EGFR scFv was added, creating a CD19-anti-Her2-anti-EGFR FP. FP activity was analyzed using binding and cytotoxicity assays. Primary T cells transduced with the CAR19/CD19-anti-Her2+ FP were assayed for in vivo efficacy using Her2+ tumor cells implanted into NSG mice.

**Results**

The CD19/anti-Her2 and anti-Her2/EGFR FPs were highly potent in cytotoxicity assays targeting Her2+, EGFR+ and dual positive solid tumor cell lines. The concentration of CD19/anti-Her2 fusion protein required redirect the CAR19 to kill 50% of the tumor cells was 10 pM (0.7 ng/ml). Transduced primary donor T cells secreted ~ 20 ng/ml of the CD19/anti-Her2 FP in cell culture and expressed CAR19 on the cell surface. All CAR19-based primary T cells killed CD19+ Nalm6 tumor cells. Redirected cytotoxic activity against Her2+ SKOV3 tumor cells was demonstrated in vitro and in vivo. A bispecific FP containing CD19 linked to anti-Her2 and anti-EGFR scFv was very potent against tumor cells expressing both antigens (IC50=0.75 pM). For each antigen, cytotoxicity was specifically mediated by the secreted fusion protein. Additional program examples of multi-specific targeting for diverse hematologic and solid tumor types will be shown.

**Conclusions**

The IMPACT platform addresses critical issues in cell therapy including CAR persistence, antigen escape and antigen heterogeneity, and provides important solutions for treating both hematologic and solid tumors. The potency of redirected cytotoxicity supports clinical development of CAR19/IMPACT programs, four of which are now ready for IND enabling studies.

#### P268 Heterogeneity of pancreatic cancer tumor-infiltrating T cells at the single cell level

##### Donastas Sakellariou-Thompson, BS, Aislyn Schalck, Mark Hurd, PhD, Gauri Varadhachary, Milind Javle, MD, Anirban Maitra, MBBS, Nicholas Navin, PhD

###### MD Anderson Cancer Center, Houston, TX, USA

####### **Correspondence:** Donastas Sakellariou-Thompson (chaymaker@mdanderson.org)

**Background**

Pancreatic ductal adenocarcinoma (PDAC) has a 5-year survival rate of only 8%, and attempts to improve its outcome with checkpoint blockade have been ineffective. Although the T-cell infiltrate is poor, the presence of CD3+ tumor-infiltrating lymphocytes (TIL) in PDAC is correlated with better survival and our previous work demonstrated that tumor-reactive CD8+ TIL can be expanded from PDAC. While exploring the TIL repertoire, high frequency T-cell clones overlapping between the tumor and uninvolved-tissue were observed, suggesting the presence of tissue-resident memory T cells (Trm). These cells are often marked by expression of CD103, and recent work in other cancer types showed that tumor-reactive cells can be found in the Trm subset of CD8+ TIL.

**Methods**

High-order flow cytometry was performed on TIL from 5 primary human PDAC tumor samples examining expression of CD103 and several costimulatory and inhibitory markers. Additionally, single-cell RNA sequencing (scRNA-seq) was performed on the same samples to generate gene expression profiles paired with T-cell receptor sequences. Unbiased clustering was used to visualize the TIL clusters. TIL clones were defined as significantly expanded if they had a < 0.05 FDR-adjusted probability of being observed at, or above, the measured frequency when sampled from a multinomial distribution of all observed clones at even frequencies.

**Results**

Flow cytometry revealed that most PDAC samples did not contain substantial CD103+ population except for one. Therefore sequencing data was explored to find a Trm gene signature in PDAC that may not be principally defined by CD103. To date, 2,237 TIL have been sequenced. Initial clustering of CD8+ TIL showed a Trm-like group (e.g. CXCL13, LAG3, CTLA4 and CD39) and an associated mitotic subset (e.g. STMN1, H2AFZ, TUBB, and Ki67) whose phenotype aligned with assessed markers by flow cytometry. However, these groups were almost exclusively mapped to the sole CD103+ sample. After removing this sample to better define the CD103- populations, the resulting CD8+ clusters revealed a cytotoxic TIL population with no clear Trm-like signature within the effector memory subset. CD4+ TIL split into two distinct groups containing an activated Treg cluster and a conventional CD4+ T-cell cluster. The expression profile of expanded TCR clonotypes was also explored and will be discussed.

**Conclusions**

This study shows the potential of combining multi-parameter flow cytometry and scRNA-seq in defining functional heterogeneity of TIL in PDAC.

**Ethics Approval**

The study was approved by the Institutional Review Board of the University of Texas MD Anderson Cancer Center (LAB00-396, PA15-0014).

**Consent**

Written informed consent was obtained from the patients for publication of this abstract. A copy of the written consent is available for review by the Editor of this journal.

#### P269 Global phosphoproteomic analysis of chimeric antigen receptor and T cell receptor signaling enables design of modified receptors with distinct properties

##### Alexander Salter, BS, Richard Ivey, Anusha Rajan, Jacob Kennedy, Valentin Voillet, PhD, Jeffrey Whiteaker, Raphael Gottardo, PhD, Stanley Riddell, MD, Alexander Salter, BS

###### Fred Hutchinson Cancer Research Center, Seattle, WA, USA

####### **Correspondence:** Alexander Salter (apaulovi@fredhutch.org)

**Background**

Chimeric antigen receptors (CARs) are synthetic proteins that redirect T cell specificity to tumor-associated antigens by mimicking certain aspects of T cell receptor (TCR) signaling. Most CARs contain the T cell-activating CD3z endodomain and a costimulatory domain from CD28 or 4-1BB. T cells expressing CD28/CD3z or 4-1BB/CD3z CARs are effective at treating refractory B cell malignancies but exhibit differences in effector function and cell fate that impact clinical efficacy and toxicity. These differences are assumed to result from activation of divergent signaling cascades, but the signaling pathways initiated by CARs and how these pathways compare to TCRs are poorly understood.

**Methods**

We developed a shotgun mass spectrometry platform to study CAR and TCR stimulation-induced protein phosphorylation events in primary human T cells. By coupling this technology to RNAseq, immunoprecipitations, in vitro measures of T cell function, and xenograft mouse models, we comprehensively traced how CAR and TCR- induced changes in protein phosphorylation alter T cell fate and function.

**Results**

Stimulation of CD28/CD3z or 4-1BB/CD3z CAR T cells activated nearly identical phosphoprotein signaling cascades. Instead, the major difference between the two CARs related to signaling kinetics and intensity whereby CD28/CD3z CAR stimulation produced faster and greater magnitude changes in protein phosphorylation. Increased CD28/CD3z CAR signal strength was related to CAR-Lck association and promoted an effector cell-like transcriptional profile and greater susceptibility to T cell exhaustion in a xenograft model of disseminated lymphoma. Mass spectrometry-guided mutagenesis of the CAR CD28 signaling domain enabled creation of fully- functional mutant CD28/CD3z CARs with reduced intensity. Further comparison of CAR to TCR signaling identified signaling adapters not engaged by CAR stimulation and provided a framework for altering CAR structure to engage these molecules.

**Conclusions**

CD28/CD3z and 4-1BB/CD3z CAR signaling is qualitatively similar and differs primarily in kinetics and magnitude. Increased TCR signaling strength is known to promote short-lived effector responses at the expense of memory formation and we propose that signal strength is also a major determinant of CAR T cell function and fate. Modification of CAR structure to fine-tune signal quantity and/or engage accessory signaling adapters may lead to more effective therapeutic receptors.

**Acknowledgements**

This work was supported by R01CA114536, FHCRC Bezos Immunotherapy Pilot Award, and FHCRC Interdisciplinary Cancer Research Training Grant.

**Ethics Approval**

Primary T cells were obtained from peripheral blood of healthy adults under a Fred Hutchinson Cancer Research Center Institutional Review Board-approved protocol (# 344). Informed consent was obtained from all enrollees.

#### P270 A novel, human sdAb-based, CAR against CD33, effectively targets AML in vitro and in vivo, and spares hematopoietic stem cells

##### Dina Schneider, PhD^1^, Ying Xiong, PhD^1^, Peirong Hu^1^, Darong Wu, MS^1^, Weizao Chen^2^, Tianlei Ying^2^, Zhongyu Zhu^1^, Dimiter Dimitrov, PhD^3^, Boro Dropulic, PhD, MBA^1^, Rimas Orentas, PhD^4^

###### ^1^Lentigen, a Miltenyi Biotec Company, Gaithersburg, MD, USA; ^2^National Cancer Institute, NIH, Frederick, MD, USA; ^3^University of Pittsburgh, Frederick, MD, USA; ^4^Seattle Children’s Research Institute, Seattle, WA, USA

####### **Correspondence:** Dina Schneider (dina.schneider@lentigen.com)

**Background**

Acute myeloid leukemia (AML) remains a challenging disease, and better treatment options are needed. Therapies targeting the AML cell surface antigen CD33 include the approved antibody drug conjugate gemtuzumab ozogamycin (My96) and investigational CART approaches incorporating CD33-binding domains derived from humanized scFvs. Single-domain antibodies (sdAbs), due to their small size, lack of mispairing risk, and human origin, are an attractive alternative option to scFv targeting domains for CARs, but have not been extensively evaluated.

**Methods**

We designed a novel chimeric antigen receptors utilizing sdAb-based targeting sequence (CAR33VH) derived by screening a human sdAb phage-display library, or the My96 scFv (My96CAR), serving as a comparator. Lentiviral expression vectors encoding each CAR construct incorporating the targeting domain in frame with a CD8 hinge and transmembrane domain, a 4-1BB costimulatory domain and a CD3 zeta activation domain, were transduced into activated primary human T cells. CAR T cells were characterized in vitro and in vivo for efficacy, specificity and toxicity against AML and for reactivity to hematopoietic stem cells (HSC) in vitro.

**Results**

The novel sdAb-based CAR33VH demonstrated robust and CD33-specific cytotoxicity in short-term and long-term killing assays against leukemic cell lines, and target-specific induction of IFN-gamma, TNF-alpha and IL-2 in response to CD33+ lines HL-60, MOLM-14 and KG-1a. Studies in A431 cells stably transduced with either full length or the clinically relevant truncated isoform of CD33, revealed that CAR33VH targets the V Ig-like domain of the full length CD33M isoform, as does the My96 CAR. Colony forming unit assays using human CD34+ HSCs pre-incubated with CAR T cells at E:T ratio of 20:1 overnight, and then transferred together to MethoCult medium supplemented with SCF, IL-3, IL-6, EPO, G-CSF, and GM-CSF for fourteen days, revealed no apparent toxicity to hematopoiesis of erythroid or myeloid lineages. In an in vivo AML model, NSG mice engrafted with the MOLM-14 leukemia cell line stably expressing firefly luciferase, both CAR33VH and CARMy96 efficiently eliminated tumors as documented by IVIS imaging.

**Conclusions**

We demonstrate for the first time the feasibility and efficacy of employing a human sdAb-derived binding domain in an anti-AML CAR design. CAR33VH was efficient in tumor killing in vitro and in vivo, and showed comparable functionality to the scFv-based My96CAR. Notably, CAR33VH had no detrimental effect on the development of myeloid or erythroid lineages in vitro, suggesting a potential for hematopoietic recovery following appropriate regulation of CAR expression in the therapeutic T cell population.

#### P271 Titratable and reversible regulation of IL12 or IL15 with FDA-approved drugs enhances CAR-T therapy

##### Steven Shamah, PhD, Brian Dolinski, BS, Kutlu Elpek, Tucker Ezell, Michelle Fleury, Jennifer Gori, PhD, Jia Gwee, Scott Heller, MS, Mara Inniss, Abhishek Kulkarni, Meghan Langley, BS, Dan Jun Li, MD, Grace Olinger, MS, Michelle Ols, PhD, Benjamin Primack, Christopher Reardon, Michael Schebesta, Dexue Sun, Zoe Tarasiewicz, BA, Karen Tran, MS, Michael Briskin, Celeste Richardson, Vipin Suri, Steven Shamah, PhD, Steven Shamah, PhD

###### Obsidian Therapeutics, Cambridge, MA, USA

####### **Correspondence:** Steven Shamah (sshamah@obsidiantx.com)

**Background**

Adoptive cell therapy with chimeric antigen receptor (CAR) modified T cells has demonstrated clinical efficacy in the treatment of B cell malignancies and multiple myeloma. Efficacy of CAR-Ts in other indications has been hindered by limited CAR-T cell expansion, immunosuppression by the tumor microenvironment, and on-target/off- tumor toxicity. Armoring CAR-Ts to produce interleukin-15 (IL15) or interleukin-12 (IL12) has been shown to drive CAR-T expansion and resistance to immunosuppression, respectively, enhancing anti-tumor activity. However, published data suggest that the uncontrolled expression of either would likely compromise safety. Technologies that provide precise control over cytokine activity could therefore broaden clinical application of CAR-T therapies.

**Methods**

We developed a platform that equips immune cells with functionalities regulated via the administration of FDA- approved small molecule ligands. We utilize small, fully human protein sequences called destabilizing domains (DDs) that confer reversible destabilization to a fused target protein. In the absence of ligand, the fusion protein is degraded, whereas the presence of ligand restores expression and functionality. Stabilization is titratable by small molecule ligand dosing and reversible, providing fine-tuned control over the timing and concentration of target protein levels.

**Results**

We developed DDs based on the human phosphodiesterase 5 (PDE5), dihydrofolate reductase (DHFR), and estrogen receptor (ER) proteins, each of which can be regulated by FDA-approved ligands with distinct pharmacokinetic properties. Using these clinically translatable DDs, we created regulated IL12 and IL15 constructs that provide exogenous regulation of cytokines for enhanced CAR-T function. Transduction of T cells with lentivirus expressing cytokine-DD fusions led to titratable cytokine production upon exposure to ligands that stabilize PDE5, DHFR or ER-regulated DDs. ER-based DDs fused to IL12, led to low levels of basal IL12 expression in the absence of ligand and rapid induction of IL12 in an ER ligand concentration-dependent manner. Treatment of T cells expressing PDE5-based DDs fused to a membrane-bound IL15-IL15 receptor alpha chimera (mbIL15) supported rapid dose- dependent increases in cell surface mbIL15 expression upon exposure to PDE5 stabilizing ligands. These findings demonstrate how cytokine levels can be precisely regulated by manipulation of drug concentration and kinetics. The functional regulation of DD-fused cytokines and enhancement of anti-tumor activity using FDA-approved drugs in a mouse CAR-T tumor model is currently in testing.

**Conclusions**

Destabilizing domains fused to proteins, coupled with FDA-approved drug administration, support exogenous control of cytokines toward the development of CAR-T cell products with enhanced efficacy and more favorable safety profiles.

#### P272 A fully-closed, high efficiency manufacturing technology platform for the production of T cell therapies targeting multiple tumor antigens

##### FABIO FACHIN, PhD, Christine McInnis, PhD, Amy Shaw, Shawn Carey, PhD, Jonas Bruun, PhD, Rachel Klaski, Pengpeng Cao, PhD, Elisabeth Brown, Andy Rakestraw, PhD, Becker Hewes, MD, Jonathan Fitzgerald, PhD, Thomas Andresen, PhD

###### Torque Therapeutics, Cambridge, MA, USA

####### **Correspondence:** FABIO FACHIN (ffachin@torquetx.com)

**Background**

Adoptive cell therapy (ACT) is a promising approach for treating tumors refractory to other treatment modalities. ACTs, however, are still largely limited to genetically-modified approaches that recognize a single antigen, require harsh pre-treatments or adjuvant drugs to enable ACT survival and function *in-vivo*, and are often associated with severe toxicities. Torque’s *Deep-PrimedTM* cell therapy platform uses novel cell process engineering to generate cytotoxic T lymphocytes (CTLs) that target multiple tumor antigens and that are tethered to Deep-IL15, a crosslinked multimer of human IL15-Fc,to deliver directed immune activation in the tumor microenvironment. Torque’s *SlipstreamTM* cell process is semi-automated and fully closed, and its modular automation design enables both large-scale and decentralized manufacturing.

**Methods**

Following apheresis, patients’ T cells and monocytes are enriched, and monocytes are rapidly differentiated into peptide-loaded mature dendritic cells (mDC) for presentation to autologous T cells. Antigen-presenting mDC are co- cultured with T cells, promoting expansion of low-frequency, antigen-specific T cells directed towards multiple tumor associated antigens (TAA). Following expansion, the multi-target T cells are loaded with Deep IL-15, cryopreserved and formulated for multiple infusions. Here, we present product composition, specificity, cytotoxicity, and the advantage of Deep IL-15 across development and GMP lots of CTLs generated from healthy donors.

**Results**

Torque's modular antigen-priming process reliably generates billions of T cells to obtain multiple drug doses via a single manufacturing run. Across donors and across both process development and GMP sites, Torque's process reliably expands endogenous antigen-specific CTLs and results in a balanced ratio of CD8+ and CD4+ T cells. The CTL products display a mixture of memory and effector phenotypes, with low expression of exhaustion markers. The products are further characterized by a unique signature against multiple TAA epitopes, with a mean 11% TAA- specificity [5-20%]. TCR sequencing reveals that the majority of clones in the final product originate from rare clones, undetectable in the incoming apheresis. Post thaw, the Deep IL-15 tethered to the CTLs ensures prolonged survival, increased expansion, and cytotoxicity against antigen-expressing cell lines.

**Conclusions**

Torque's SlipstreamTM manufacturing process uses proprietary biology processes and automation to produce Deep-PrimedTM T-Cells with high efficiency using a cGMP process. Torque's lead clinical program, TRQ15-01, is expected to start in 2018 addressing indications in both hematologic and solid tumors.

#### P273 T cells cultured in the presence of TLR9 agonist gain a CD25(high)CD39(low), pro-inflammatory phenotype and regress melanoma in vivo

##### Aubrey Smith^2^, Hannah Knochelmann^2^, Connor Dwyer, PhD^2^, Megan Wyatt, MS^2^, Michelle Nelson, PhD^3^

###### ^1^Medical University of South Carolina, Charleston, SC, USA; ^2^MUSC, Charleston, SC, USA; ^3^Aptevo Therapeutics, Seattle, WA, USA

####### **Correspondence:** Aubrey Smith (paulos@musc.edu)

**Background**

ACT therapy effectiveness is enhanced by preconditioning patients with a non-myeloablative lymphodepleting regimen [1-3]. In a mouse model of ACT one mechanism by which lymphodepletion enhances transferred T cells efficacy is by activating the innate immune system[4]. Microbes, which translocated from the radiation-injured gut, were responsible for activating innate immune cells through Toll-like receptors (TLRs), which in turn, enhanced the function of CD8+ T cells[4]. Recently, administration of TLR agonists directly to the animal has proven an efficacious therapy[5, 6]. Additional immunostimulatory molecules such as IL-2, vaccine, or anti-OX40 agonist are necessary for antitumor efficacy with TLR agonist injection[5, 6]. Thus, we questioned whether generating a potent T cell product could be achieved by simply culturing anti-tumor T cells ex vivo with TLR9 agonist, CpG.

**Methods**

We employed the Pmel mouse model of ACT in which murine T cells express a transgenic TCR that specifically recognizes the peptide gp100 expressed by melanoma. Splenocytes acquired from this mouse were cultured in the presence of CpG-ODN 1668. Following culture cells were interrogated at the phenotypic and functional level in culture and/or infused into melanoma-bearing mice to determine their anti-tumor efficacy.

**Results**

We found that cells treated with CpG in vitro have a unique, proinflammatory, phenotype with high expression of IL-2Rα(CD25), but diminished expression of the ectonucleotidease CD39 (known to promote an immunosuppression). Further cells treated with CpG in vitro have enhanced anti-tumor efficacy in vivo compared to vehicle-expanded cells. CpG does not act directly on murine T cells in culture, as neither CD8+ isolated T cells nor day 3 cultured cells (>90% T cells) do not gain the CD25highCD39low when treated with CpG. Cells treated with CpG on day 3 of culture also do not have anti-tumor efficacy above that of vehicle treated cells. Finally, removing B cells from the culture on day 0 prevents the acquisition of the phenotype associated with CpG.

**Conclusions**

A potent T cell product can be generated by the addition of the TLR9 agonist, CpG, to the cell culture. These cells have a distinct, pro-inflammatory phenotype; they are CD25highCD39low and secrete overt INFgamma, Granzyme A and Granzyme B. Further, this phenotype and the potency of T cells generated is dependent on the presence of B cells in the culture as the removal of B cells prior to treatment with CpG in culture yielded a cell product with a similar phenotype and anti-tumor efficacy to that of vehicle treated cells.

**References**

1. Goff SL, et al. Randomized, prospective evaluation comparing intensity of lymphodepletion before adoptive transfer of tumor-infiltrating lymphocytes for patients with metastatic melanoma. J Clin Oncol. 2016; 34(20):2389-97.

2. June CH. Adoptive T cell therapy for cancer in the clinic. J Clin Invest. 2007; 117(6):1466-76.

3. June CH. Principles of adoptive T cell cancer therapy. J Clin Invest. 2007; 117(5):1204-12.

4. Paulos, CM, et al. Microbial translocation augments the function of adoptively transferred self/tumor-specific CD8+ T cells via TLR4 signaling. J Clin Invest. 2007; 117(8): 2197-204.

5. Nelson MH, et al. Toll-like receptor agonist therapy can profoundly augment the antitumor activity of adoptively transferred CD8(+) T cells without host preconditioning. J Immunother Cancer. 2016; 4: 6.

6. Sagiv-Barfi I, et al. Eradication of spontaneous malignancy by local immunotherapy. Sci Transl Med. 2018; 10(426).

#### P274 Functional screening of different anti-B7H6 CAR designs

##### Sophie Agaugué, PhD^1^, Lorraine Springuel^1^, Benjamin Demoulin^1^, Martina Fontaine, PhD^1^, Adam Hargreaves, Dr^2^, Dorothée Daro^1^, Jennifer Bolsée, PhD^1^, Céline Jacques-Hespel^1^, Valérie Steenwinckel, PhD^1^, David Gilham, PhD^1^, Sophie Agaugué, PhD^1^

###### ^1^Celyad, SA, Villejuif, France; ^2^PathCelerate, Alderley Park, UK

####### **Correspondence:** Sophie Agaugué (lspringuel@celyad.com)

**Background**

B7H6, a stress-induced ligand for the NK-activating receptor Nkp30, is widely expressed on the surface of transformed cells yet absent from healthy tissues under steady state conditions. In cancers, B7H6 expression is associated with tumor progression, poor prognosis and lymph node metastasis, while B7H6 expression on tumor cells can be upregulated by conventional cancer therapies. Therefore, B7H6 represents a highly attractive target for the immunotherapy of a broad range of high risk cancers. In this work, we investigated the optimal CAR design to develop an effective B7H6-targeted CAR T cell therapy.

**Methods**

B7H6 expression was assessed on a large panel of cancer cell lines by qRT-PCR and Flow Cytometry, as well as in patient samples from neuroblastoma, colorectal and ovarian tumors by IHC. The optimal CAR design to redirect T cells against B7H6 was investigated by engineering various constructs differing in the origin of their targeting moiety (murine versus humanized scFv) and the nature of the costimulatory signaling module (CD28, 4-1BB or a combination thereof). Primary human T cells transduced with the distinct CARs were assessed for proliferation, viability, CAR expression, cellular phenotype and in vitro functionality, cytotoxicity and cytokine production, upon co-culture with B7H6-bearing cancer cells.

**Results**

We verified that B7H6 RNA and protein were expressed by human cancer cell lines of various types and confirmed by immunohistochemistry B7H6 expression in primary tumors, validating B7H6 as an attractive target for immunotherapy. Importantly, B7H6 was highly expressed in ovarian cancer, a tumor type with high unmet medical need. Based on preliminary results, CAR T cells bearing the different generations of anti-B7H6 CAR were successfully produced, although the 4-1BB-containing 2nd generation murine CAR showed very low levels of membrane expression. Expansion and viability of CAR T cells bearing the humanized scFv were reduced compared to their murine counterparts. Moreover, humanized CAR T cells released interferon γ in their supernatant and expressed markers of activation and exhaustion, reminiscent of self-reactivity. Importantly, the 2nd generation CAR comprised of the murine scFv fused to CD28-CD3ζ tail endowed T cells with the best functionality to all other constructs when co-cultured with B7H6-expressing cancer cell lines.

**Conclusions**

Functional screening of different designs indicate the B7H6-targeting CAR comprised of murine scFv fused to CD28-CD3ζ signaling tail as our prime candidate warranting further preclinical investigation for the development of a clinical product. In vivo experiments using xenograft models for assessing anti-tumor efficacy will soon be initiated.

**Ethics Approval**

All tissue samples (primary tumors and normal tissues) were acquired under appropriate IRB/ethical approvals in place in the country from which the tissue was sourced. All animal and human samples study protocols were approved by local ethical committees and authorities.

#### P275 Pooling signaling and costimulatory domains in a flexible CARpool design

##### Jennifer Bolsée, PhD^2^, Lorraine Springuel^1^, Amélie Velghe^2^, Sophie AGAUGUE, PhD^2^, David Gilham, PhD^2^

###### ^1^Celyad, SA, Villejuif, France; ^2^Celyad, Mont-saint-Guibert, Belgium

####### **Correspondence:** Jennifer Bolsée (jbolsee@celyad)

**Background**

CARs are modular receptors that consist of a target binding moiety fused to structural domains including an extracellular spacer, a transmembrane region and intracellular signaling domains. These signaling regions typically comprise a tandem alignment of co-stimulatory (e.g. CD28, CD137) and activatory (CD3ζ) domains that upon target binding initiate activation of T cell effector functions. This linear configuration displays a rigid spatial orientation and ratio of co-stimulation to activation domains. To address this, we have developed a novel mix-and -match approach (CARpool) where the costimulatory signal is provided in trans on accessory proteins that associate with the antigen binding chain via transmembrane-mediated interactions, potentially driving the ability to tailor T cells responses upon CAR activation.

**Methods**

Exploiting the ability of NK activating receptors to assemble as multi-subunit complexes via membrane-embedded opposite charge interactions, several CD3ζ-containing CAR chains were designed using the transmembrane and cytoplasmic domains of NKG2D or NKp44, able to associate with DAP10 and DAP12 respectively. Each CAR included a B7H6 specific scFv. The CAR- and accessory protein-encoding sequences were co-expressed using 2A self-cleaving sites within the pSFG vector backbone. These constructs were compared to a classical second- generation CAR construct containing an intracellular CD28 costimulatory domain. Primary human T-cell populations expressing the diverse constructs were screened for CAR expression, T cell phenotype and in vitro function (cytokine secretion and cytolytic activity) upon co-culture with B7H6-expressing cell lines.

**Results**

NKG2D-based CAR complexes were moderately expressed at the cell surface but bound B7H6 and released cytokines upon co-culture with B7H6-expressing cancer cells. Modification of the position of the charged residue within the transmembrane domain of the CAR is being used to modulate the surface expression of the receptor. NKp44-based CAR complexes were more frequently expressed on primary T cells and binding to B7H6 was confirmed, validating the feasibility of the approach although functionality of these NKp44 based receptors appears currently to be more limited. We are optimizing these receptors including hinge domains in order to enhance functionality.

**Conclusions**

These studies provide proof-of-concept for a novel modulatory CAR design where it is possible to incorporate or interchange optimal costimulatory domain(s) depending on the target of interest and in a stoichiometrically controlled way. Notably, this does not necessitate subcloning the CAR chain itself. Importantly, recapitulating physiological TCR activation by providing co-stimulation in trans within the CARpool may result in optimal downstream signaling, thereby enhancing anti-tumoral activity of CAR T cells.

**Ethics Approval**

Human samples study protocols were approved by local ethical committees and authorities.

#### P276 Characterization of systemic and local immunity following adoptive transfer of NY-ESO-1 SPEAR T-cells in synovial sarcoma (NCT01343043)

##### Samik Basu, MD, Justina Stadanlick, Indu Ramachandran, PhD, Daniel Lowther, Rebecca Dryer-Minnerly, PhD, Ruoxi Wang, Svetlana Fayngerts, Daniel Nunez, Natalie Bath, MSc, Gareth Betts, PhD, Karen Chagin, MD, Thomas Faitg, PhD, Wayne Higgins, Malini Iyengar, PhD, Luca Melchiori, Siva Samavedam, Jonathan Silk, PhD, Alex Tipping, PhD, Trupti Trivedi, MS, Erin Van Winkle, Lilli Wang, Rafael Amado, MD, Gwendolyn Binder, Samik Basu, MD

###### Adaptimmune, Philadelphia, PA, USA

####### **Correspondence:** Samik Basu (samik.basu@adaptimmune.com)

**Background**

Gene-modified autologous T-cells expressing NY-ESO-1c259, an affinity-enhanced T-cell receptor (TCR) reactive against the NY-ESO-1-specific HLA-A*02-restricted peptide SLLMWITQC (SPEAR T-cells; GSK 794), have demonstrated clinical activity in patients with advanced synovial sarcoma (SS). The factors contributing to gene- modified T-cell expansion and the changes within the tumor microenvironment following T-cell infusion remain unclear. Here, we report on phenotypic and functional studies on T-cells, sera, and tumor biopsies from SS patients treated with NY-ESO-1 SPEAR T-cells.

**Methods**

Engineered T-cell persistence was determined by qPCR for the vector backbone in post-infusion PBMC samples.

Serum cytokines were measured via a multiplexed electrochemiluminescent MSD immunoassay. Multiplexed gene expression analysis and immunohistochemistry for immune markers (e.g. CD8) were performed on formalin-fixed paraffin-embedded (FFPE) tumor biopsies from patients prior to and following adoptive T-cell transfer. Additionally, RNA in situ hybridization (RNAish) was performed on FFPE tumor biopsies to detect the presence or absence of gene-modified T-cells within the tumor microenvironment following adoptive transfer. Clinical responses were assessed by RECIST v1.1.

**Results**

The magnitude of gene-modified T-cell expansion within two weeks after infusion was associated with response in patients with high expression of intra-tumoral NY-ESO-1 antigen expression (2+ or 3+ in ≥ 50% cells by IHC). Patients receiving a fludarabine-containing conditioning regimen experienced an increase in serum levels of the homeostatic lymphocyte cytokines IL-7 and IL-15. Prior to infusion, the SS microenvironment exhibited minimal leukocyte infiltration. CD163+ tumor-associated macrophages (TAMs) were the dominant population. An increase in leukocytes and lymphocytes within the tumor microenvironment was observed at the post-infusion tumor biopsy, at approximately 8 weeks. At time points greater than 8 weeks post infusion, the tumor microenvironment was minimally infiltrated with a TAM-dominant leukocyte infiltrate. Notably, genes encoding tumor-associated antigens and antigen presentation did not significantly change within the tumor post-T-cell infusion. Gene-modified NY- ESO-1c259TCR T-cells were capable of infiltrating the SS tumor microenvironment in a subset of tumor samples tested.

**Conclusions**

Our studies elucidate some of the factors that underpin response and resistance to adoptive gene-modified T-cell transfer in solid malignancies. Furthermore, these data demonstrate that non-T-cell inflamed tumors of a type that are often resistant to immune checkpoint blockade can be successfully treated with adoptive T-cell based immunotherapy.

**Trial Registration**

NCT01343043

**Ethics Approval**

The protocol was approved by each center’s Institutional Review Board, and all patients signed informed consent forms.

#### P277 Genetic modification of IL13Rα2-CAR T cells to express secretory or membrane-bound IL-15 enhances their anti-glioma activity without discernible differences

##### David Steffin, MD^1^, Haley Houke^2^, Tim Sauer, MD^1^, Irina Balyasnikova, PhD^3^, Stephen Gottschalk^2^, Center director Rooney, PhD^1^, Giedre Krenciute, PhD^2^

###### ^1^Texas Children's Hospital, Houston, TX, USA; ^2^St. Jude Children's Research Hospital, Memphis, TN, USA; ^3^Northwestern University, Chicago, IL, USA

####### **Correspondence:** David Steffin (dhsteffi@texaschildrens.org)

**Background**

Immunotherapy with genetically modified T cells expressing chimeric antigen receptors (CARs) has the potential to improve outcomes for patients with glioblastoma (GBM), a type of brain cancer with dismal outcomes [1]. We have shown that IL13Rα2-CAR T cells have potent antitumor activity in preclinical GBM models, and that expression of secretory (s) IL15 further enhances their anti-glioma activity [2]. Several studies have suggested that membrane bound (mb) IL15 has greater biological activity than sIL15 [3]. However, the effector function of CAR T cells expressing mbIL15 or sIL15 have never been directly compared. Thus, the aim of this study was to generate IL13Rα2-CAR.sIL15 and IL13Rα2-CAR.mbIL15 T cells, and compare their anti-glioma activity.

**Methods**

We generated two retroviral vectors encoding an IL13Rα2-CAR with a CD28.ζ endodomain and sIL15 or mbIL15 separated by 2A sequence; mbIL15 consisted of IL15 linked to the CD8α stalk and transmembrane domain. In addition, both vectors encoded CD20 to facilitate detection of transduced T cells and to serve as a potential suicide switch. Genetically-modified T cells were generated by retroviral transduction. Transgene expression was confirmed by FACS analysis (IL13Rα2-CAR, CD20, mbIL15) or ELISA (sIL15).

**Results**

In cytotoxicity assays, IL13Rα2-CAR, IL13Rα2-CAR.sIL15 and IL13Rα2-CAR.mbIL15 T cells readily killed IL13Rα2+ U373 glioma cells with no significant differences between effector T-cell populations. Expression of sIL15 or mbIL15 significantly enhanced the expansion of IL13Rα2-CAR T cells upon stimulation with U373 glioma cells and CAR T cells expressing sIL15 secreted significantly higher amounts of INFg when compared to IL13Rα2- CAR.mbIL15. However, there was no significant difference in the fold-expansion of IL13Rα2-CAR.sIL15 and IL13Rα2-CAR.mbIL15 T cells.

**Conclusions**

We demonstrate here that expression of sIL15 or mbIL15 in IL13Rα2-CAR T cells enhance their anti-glioma activity. Our in vitro studies indicate so far that there is no significant difference in the effector function of IL13Rα2-CAR.sIL15 and IL13Rα2-CAR.mbIL15 T cells. In vivo studies are in progress to confirm our findings.

Thus, genetic modification of IL13Rα2-CAR T cells with sIL15 or mbIL15 presents a promising strategy to enhance their anti-glioma activity.

**References**

1. Fangusaro, J. Pediatric high grade glioma: a review and update on tumor clinical characteristics and biology. Frontiers in oncology. 2012;2:105.

2. Krenciute, G.; Krebs, S.; Torres, D.; Dotti, G.; Lesniak, M.S.; Balyasnikova, I.V.; Gottschalk, S. Charachterization and functional analysis of scFv-based CARs to redirect T cells to IL13Rα2-positive glioma. Journal for immunotherapy of cancer. 2015;3(S2):P116.

3. Imamura, M.; Shook, D.; Kamiya, T.; Shimasaki, N.; Chai, S.M.; Coustan-Smith, E.; Imai, C.; Campana, D. Autonomous growth and increased cytotoxicity of natural killer cells expressing membrane-bound interleukin-15. Blood. 2014:blood-2014-2002-556837.

#### P278 Identification of senescent T-cell subsets that impact gene-engineered T cell manufacturing success and product consistency

##### Jeffrey Teoh, PhD, Daniel Cossette, Sara Cooper

###### Juno Therapeutics, a Celgene Company, Seattle, WA, USA

####### **Correspondence:** Jeffrey Teoh (ryan.larson@junotherapeutics.com)

**Background**

Autologous chimeric antigen receptor (CAR) T-cell therapy has demonstrated significant clinical benefit in hematological malignancies. The autologous nature of CAR T-cell therapeutic modalities introduces heterogeneity into the manufacturing process. Both pre- and post-manufacturing memory T-cell phenotypes have been demonstrated to correlate with clinical outcome [1,2], and enrichment for early memory T-cell subsets is associated with improved manufacturing success [3]. However, little is known regarding the mechanisms by which T-cell phenotypic heterogeneity impacts the manufacturing process and pre-infusion CAR T-cell profiles. Here we demonstrate the presence of a T-cell subset that does not appear to expand in CAR T cell manufacturing, but continues to consume growth factors as well as drive process and product heterogeneity.

**Methods**

CD19-directed CAR T cells were activated through engagement of CD3/CD28, transduced, and expanded in vitro.

Throughout this process, T cells were assessed for expansion, viability, activation, memory differentiation state, and cell cycle state. Functionality of the final drug product was also assessed, including cytokine production and cell survival following CAR-specific stimulation.

**Results**

The majority of T cells upregulated activation markers, including CD25 and CD69, in the presence of the CD3/CD28 stimulus, whereas only a subset of activated T cells entered cell cycle based on Ki67 expression. The Ki-67+ fraction primarily comprised cells expressing CD27 and CD28, whereas Ki67- populations were enriched for CD57+ cells and corresponded with CD27-CD28- phenotypes. CD57+ cells exhibited activated phenotypes and persisted throughout early process stages. The proportion of CD57+ T cells decreased after 48hrs, which coincided with T-cell expansion and increased viability. To directly assess the impact of CD57+/- T cells in CAR T-cell manufacturing, CD57+ cells were positively selected from starting material and combined with CD57- cells at various ratios prior to activation. The proportion of CD57+ cells in starting material correlated with process duration, and CAR T cells did not expand in the condition containing 95% CD57+ T cells.

**Conclusions**

Whereas CD27+ T cells appear to contribute to the majority of expanded cells in process manufacturing, CD57+ cells do not expand and appear to contribute minimally to the final drug product. CD57+ cells consume IL-2, occupy activation reagents, and contribute to cell counts used for establishing and maintaining cell culture. Thus, CD57+ T cells can directly impact upstream process operations and introduce variability in downstream expansion processes and product attributes. Selective depletion of CD57+ T cells at process initiation could augment cellular manufacturing success and further improve drug product consistency.

**References**

1. Fraietta JA, Lacey SF, Orlando EJ, Pruteanu-Malinici I, Gohil M, Lundh S, Boesteanu AC, Wang Y, O'Connor RS, Hwang WT, Pequignot E, Ambrose DE, Zhang C, Wilcox N, Bedoya F, Dorfmeier C, Chen F, Tian L, Parakandi H, Gupta M, Young RM, Johnson FB, Kulikovskaya I, Liu L, Xu J, Kassim SH, Davis MM, Levine BL, Frey NV, Siegel DL, Huang AC, Wherry EJ, Bitter H, Brogdon JL, Porter DL, June CH, Melenhorst JJ. Determinants of response and resistance to CD19 chimeric antigen receptor (CAR) T cell therapy of chronic lymphocytic leukemia. Nat Med. 2018; 24(5):563-571.

2. Larson RP, Lower R, DeVries T, Jiang Y, Hause RJ, Getto R, Christin B, Yee NK, Bowen MA, Weber C, Li D, Albertson T, Sutherland C, Ramsborg CG. Defined cell composition and precise control over JCAR017 dose enables identification of relationships between chimeric antigen receptor T cell product attributes, pharmacokinetics, and clinical endpoints in NHL. Cancer Res. 2018; 78(13 Suppl):Abstract nr 960.

3. Singh N, Perazzelli J, Grupp SA, Barrett DM. Early memory phenotypes drive T cell proliferation in patients with pediatric malignancies. Sci Trans Med. 2016; 8(320):320ra3

#### P279 CRISPR/Cas9 enables the efficient production of allogeneic CAR-T cells engineered to contain multiple genome edits to enhance therapeutic T-cell function

##### Demetrios Kalaitzidis, PhD, Ashley Porras, BA, Dan Henderson, Sushant Karnik, Katie Levitsky, BS, Jason Sagert, PhD, Zinkal Padalia, MS, Mary Lee Dequeant, PhD, melanie allen, BS, Hanspeter Waldner, Henia Dar, PhD, Chandirasegaran Massilamany, PhD, Paul Tetteh, Dakai Mu, BA, Elaine Huang, PhD, Thao Nguyen, Sarah Spencer, PhD, Kelly Maeng

###### Crispr Therapeutics, Cambridge, MA, USA

####### **Correspondence:** Demetrios Kalaitzidis (jonathan.terrett@crisprtx.com)

**Background**

The CRISPR/cas9 system allows for rapid assessment of the consequences of perturbing many genes while at the same time deriving potentially lead molecules for cell and gene therapies

**Methods**

This technology was applied to primary human T cells to produce allogeneic CAR-T cells by multiplexed genome editing. A robust system has been developed for site-specific integration of CAR and multiplexed KO generation by utilizing homology-directed repair (HDR) with Cas9 ribonucleoprotein (RNP) and an AAV6-delivered donor template.

**Results**

The CRISPR/cas9 multi editing system was used to discover the following in the allogeneic CAR T cell setting: 1) multiple edits (>5) can be applied efficiently producing stable non transformed CAR T cells, 2) the effects of single and multiple edits on CAR T function can be examined efficiently to determine gene edits that improve CAR T cell function, 3) the consequence of these effects for on target and off target activity can be used to rapidly generate lead gRNAs, 4) next generation cell therapies can be defined towards targeting solid tumors with allogeneic CAR T cells.

Here we show the effects in vitro and in vivo of knocking out multiple genes singly and in combination, including the response to multiple antigen challenges and the ability to overcome PDL1 induced resistance.

**Conclusions**

Producing CAR T cells with multiple edits could be an important step towards enhancing the ability of this therapeutic class to tackle solid tumors with improved efficacy over the current therapeutics.

#### P280 IL-12 cytokine priming of low avidity tumor specific T cells enhances tumor clearance and prevents exhaustion

##### Christopher Tucker, BS, Jason Mitchell

###### University of Minnesota, Minneapolis, MN, USA

####### **Correspondence:** Christopher Tucker (bfife@umn.edu)

**Background**

Checkpoint blockade therapy has largely failed in patients that lack immune infiltration of their tumor. To generate a de novo immune response in these patients, adoptive cellular therapy (ACT) has been attempted with the transfer of high avidity tumor reactive T cells. This has resulted in significant autoimmunity and long term therapeutic failure. We and others have hypothesized this autoimmunity is due to this use of high avidity T-cells reacting to healthy tissue. We therefore hypothesized that low avidity T-cells could provide comparable tumor control but limit these autoimmune side effects with the optimal cytokine priming regimen.

**Methods**

Using high and low avidity transgenic tumor reactive CD8+ T-cells specific for tyrosinase-related protein-2 (TRP2) we investigated how IL-2 alone or IL-12/IL-2 priming affected these two different T-cell populations. We crossed these TRP2 specific transgenic mice to GFP and Brainbow Red mice to track antigen specific T-cell populations during adoptive transfer and investigated cytokine production and cell surface markers with flow cytometry and two-photon microscopy.

**Results**

Low avidity TRP2 T-cells primed with IL-2 alone failed to control melanoma growth, and became tolerized within the tumor environment. Additionally these T-cells were poorly cytolytic to tumor targets and underwent increased apoptosis when co-cultured with tumor cells. To overcome this deficiency, our lab had previously published that IL- 12 cytokine priming during activation of adoptively transferred T-cells provided superior tumor control. With these experiments we show that IL-12 restores low avidity T-cell functionality and tumor cytolysis potential while preventing T-cell exhaustion by down regulation of PD-1. We additionally show that PD-1 checkpoint blockade provides no additional therapeutic benefit when transferring IL-12 primed CD8+ T-cells. Finally we show that with tumor antigen overexpression and IL-12 priming low avidity T-cells can provide long term tumor control.

**Conclusions**

Taken together these data indicate that IL-12 priming of low avidity T-cells could be an effective approach in clinical ACT protocols. This protocol could provide superior safety margins in cancer patients by avoiding autoimmunity while maintaining therapeutic efficacy.

#### P281 Endogenous MHC class II restricted CD4+ T cell responses to recurrent driver mutations in melanoma and non-small cell lung cancer (NSCLC)

##### Joshua Veatch, MD, PhD^1^, Brenda Jesernig, MS^1^, Sylvia Lee^2^, Stanley Riddell, MD^1^

###### ^1^Fred Hutchinson Cancer Research Center, Seattle, WA, USA; ^2^University of Washington, Seattle, WA, USA

####### **Correspondence:** Joshua Veatch (jveatch@fhcrc.org)

**Background**

T cell responses to mutated neoantigens are thought to mediate clinical responses to immune checkpoint inhibition and adoptive cell transfer in cancers with high mutation burdens. Most T cell responses to neoantigens recognize patient specific mutations, posing an obstacle for design of vaccination or adoptive transfer strategies. Here, we identify endogenous T cell responses in patients with cancer to 3 different recurrent oncogenic driver mutations: BRAF V600E, KRAS G12V and the ERBB2 (Her2) internal tandem duplication (Her2-ITD).

**Methods**

Neoantigen-reactive T cells were expanded and cloned after stimulating lymphocytes from melanoma and NSCLC patients with peptide panels spanning mutations identified by exome sequencing.

**Results**

MHC class II restricted CD4+ T cells specific to the BRAF V600E mutation found in 40% of melanoma were isolated from a melanoma patient following a durable complete response to tumor infiltrating lymphocyte therapy.

BRAF V600E specific cells showed a Th1 memory phenotype, were preferentially localized to the tumor site, and expanded and persisted in blood greater than 2 years after TIL therapy. The tumor from this patient had fewer than 30 somatic mutations and the BRAF V600E specific CD4+ T cell response was correlated with robust and persistent CD8+ T cell responses to multiple self–antigens. We also identified CD4+ T cell responses to the recurrent KRAS G12V and Her-ITD mutations in 2 different patients with lung adenocarcinoma. In all cases, the T cells recognized MHC class II expressing cells expressing the mutant but not wildtype sequences, and the Her2-ITD specific T cells localize preferentially to the tumor relative to the normal lung. T cell receptors (TCR) were isolated and conferred specificity to BRAF V600E, KRAS G12V and Her2-ITD following gene transfer. Each TCR was restricted by common class II HLA allele found in 10-25% of the population.

**Conclusions**

This study greatly expands the number of driver mutations that can be targeted by immunotherapy. Clinical evaluation of adoptive transfer or vaccination strategies should help interrogate the role of MHC class II restricted CD4+ T cells in human anti-tumor immunity, and could have clinical activity across multiple patients.

**Ethics Approval**

These studies were approved by the Institutional review board at the Fred Hutchinson Cancer Research Center, approval numbers 2643, 1765 and 1246.

#### P282 Pathogen-reduced human platelet lysate as a serum replacement maintains CAR T cells in a less- differentiated phenotype associated with superior anti-tumor function

##### Norihiro Watanabe, PhD^1^, Alejandro Torres Chavez^1^, Emanuele Canestrari, PhD^2^, Christina Dann, PhD^2^, Ann Leen^1^, Juan Vera^1^

###### ^1^Baylor College of Medicine, Houston, TX, USA; ^2^Cook Regentec, Indianapolis, IN, USA

####### **Correspondence:** Norihiro Watanabe (nxwatana@txch.org)

**Background**

Many groups have shown that the maintenance of a naive-like (TN) and central memory (TCM) CAR T cell phenotype during in vitro production is associated with prolonged in vivo T cell persistence resulting in potent anti- tumor effects. In this study we explored the impact of different media supplements on CAR T cell phenotype and function.

**Methods**

As a model system we used T cells modified with a second generation CAR targeting prostate stem cell antigen (PSCA) containing a CD28/CD3z endodomain, as previously published by our group. Three days post-retrovirus transduction in the presence of IL2 with 10% fetal bovine serum (FBS), CAR T cells were divided into 3 groups that were cultured in medium supplemented with either 10% FBS, 10% human AB serum (ABS) or 10% pathogen reduced human platelet lysate (PR HPL). Following a minimum of 7 days in culture under these conditions we evaluated CAR T cell growth, phenotype, and short- and long-term *in vitro* cytolytic activity and *in vivo* anti-tumor effects.

**Results**

We monitored T cell expansion over a 14-day period and found that CAR T cell growth was similar among the conditions. Interestingly, populations of CCR7+ cells (TN and TCM) were dramatically increased when CAR T cells were maintained in 10% PR HPL (CD4+: 66.7±2.1%; CD8+: 69.6±4.7%) compared to 10% FBS (CD4+: 36.2±3.3%; CD8+: 20.8±3.1%) or 10% ABS (CD4+: 15.8±1.6%; CD8+: 7.3±1.5%). Although CAR T cells maintained in PR HPL exhibited slightly lower cytolytic ability in a 5 hr 51Cr release assay because of highly enriched/less differentiated T cell populations, they showed potent anti-tumor effects with enhanced T cell expansion in a 9 day coculture assay. Importantly, these CAR T cells (1x10e6 cells/mouse) were able to eliminate established subcutaneous tumors in xenograft mice engrafted with PSCA-expressing tumors while cells maintained in either FBS or ABS only delayed but did not eliminate tumors. Furthermore, when we performed a tumor re- challenge experiment in mice who cleared their primary tumor by injecting with 2x10e6 cells/mouse, only animals that initially received PR HPL cultured CAR T cells exhibited CAR T cell accumulation and expansion at the re- challenge tumor site, resulting in anti-tumor effects, suggesting their capacity for long-term *in vivo* persistence.

**Conclusions**

Our work indicates that exposure of CAR T cells to PR HPL during *ex vivo* culture leads to improved maintenance of less differentiated T cells with enhanced persistence and *in vivo* anti-tumor killing ability.

#### P283 Allogeneic CAR T-cells resistant to both T- and NK-cell cytotoxicity

##### Alan Williams^1^, Laurent Poirot, PhD^2^, Philippe Duchateau^2^, brian Busser^2^, Alexandre juillerat^3^, Stephane depil^2^, Sonal Temburni-Blake, MS^3^

###### ^1^Cellectis. Inc, NYC, NY, USA; ^2^Cellectis, Paris, France; ^3^Cellectis.Inc, NYC, USA

####### **Correspondence:** Alan Williams (julien.valton@cellectis.com)

**Background**

The last years have seen the adoptive transfer of engineered autologous T-cells making great strides in the development of new treatments against cancer. The use of third party donor-derived T-cells represents an attractive alternative to generate CAR T-cells readily accessible to patients. These off-the-shelf cells derived from a possibly non-HLA-matched donor nevertheless carry the risk of graft-versus-host-disease (GvHD) through the expression of their endogenous T-cell receptors (TCRs).The recent advances in precise genome editing using designer nucleases allowed to mitigate this issue as demonstrated by the molecular remissions observed in patients after infusion of universal TALEN® multiplex gene-edited CAR T-cells. In particular, these universal CAR T-cells were engineered to reduce the risk of graft versus host disease (GvHD) by TALEN® inactivation of TCRαβ. The clinical outcome of CAR T-cell therapies is intimately linked to the ability of effector cells to engraft and proliferate in order to eradicate tumor cells within patients. Although the transient activity of off-the-shelf CAR T-cells represents an important safety feature, the possibility to extend their therapeutic window may be desirable in particular disease indications and after lymphodepleting regimens where the patient’s immune system has been restored. Thus, we are developing novel approaches to render these cells less visible to both host T- and NK-cells.

**Methods**

The single genetic disruption of the beta-2 microglobulin (B2M) gene, a required component of all MHC class I molecules, promotes resistance to host CD8+ T-cell attack but may trigger NK-cell activation, leading to the rejection of the edited T-cells lacking MHC-I. We therefore further engineered these B2M negative cells via a TALEN®-mediated and B2M gene-specific targeted integration matrix to express inhibitors of NK cytotoxicity.

**Results**

We have identified an NK-cell inhibitor that successfully blocks the so-called NK-cell “missing self-response” in vitro and in vivo. Altogether, the precise TALEN®-mediated inactivation of B2M coupled with insertion of an NK- cell inhibitor enabled the generation of allogeneic CAR T-cells, resistant to host T- and NK-cells, with improved persistence and long-term anti-tumor activity.

**Conclusions**

We believe that this strategy provides a frame work to generate a universal CAR T-cells that will enable large scale utilization of adoptive T-cell therapies and thus benefit a broader range of patients.

#### P284 Importance of the enzymatic activity of CD26 expressed on tumor-specific Th17 cells for adoptive cell therapy

##### Megan Wyatt, MS^2^, Stefanie Bailey^2^, Hannah Knochelmann^2^, Aubrey Smith^2^, Connor Dwyer, PhD^2^, Michelle Nelson, PhD^2^

###### ^1^MUSC, Charleston, SC, USA; ^2^Medical University of South Carolina, Charleston, SC, USA

####### **Correspondence:** Megan Wyatt (paulos@musc.edu)

**Background**

Adoptive T-cell immunotherapy (ACT), an exciting breakthrough in cancer treatment, utilizes patients’ own tumor-specific T-cells to fight their cancer. However, not all T cells are equal in their tumor-fighting ability. We have recently discovered that CD4+ T cells that express dipeptidylpeptidase 4, or CD26, possess enhanced antitumor properties in three different aggressive models [1]. CD26 has several important functions that may play a role in antitumor responses, including enzymatic cleavage of chemokines involved in T cell migration. We sought to determine whether the CD26 protein is necessary for the observed enhanced anti-tumor efficacy, or if it is just a marker of an excellent memory cell population.

**Methods**

B16F10 melanoma tumors were subcutaneously injected on either wild-type (WT) mice or CD26-/- mice, both on a C57BL/6 background. Prior to ACT, the mice were given drinking water +/- 75 mg/kg daily dose of sitagliptin, an inhibitor of CD26 enzymatic activity. CD26+ Th17 cells isolated and expanded from TYRP transgenic mice expressing the TRP-1 T cell receptor that recognizes tyrosinase were adoptively transferred into the tumor-bearing mice, and tumor burden was measured for 128 days, during which sitagliptin treatment was constant.

**Results**

As has been previously demonstrated [2], sitagliptin treatment enhanced anti-tumor response of the Th17 cells in WT hosts initially. However, as the experiment progressed many of the initial responders regressed, so that survival +/- sitagliptin in WT mice was not significantly different. In the absence of host CD26, the Th17 cells drastically decreased tumor burden and increased survival (8/10 responders; survival of WT vs CD26-/- p=0.016, WT+sitagliptin vs. CD26-/- p=0.008, Mantel-Cox Logrank test). In sitagliptin-treated CD26-/- mice, the anti-tumor effect of the Th17 cells was diminished and 9/10 mice lost control of their tumor by day 78 post-treatment (survival of CD26-/- vs. CD26-/- +sitagliptin p=0.005, Mantel-Cox Logrank test).

**Conclusions**

The results of this study indicate that CD26 enzymatic activity is important for the ability of Th17 cells to produce an anti-tumor response, as the antitumor response of Th17 cells was lost in CD26-/- mice treated with sitagliptin.

Further studies utilizing CD26-/- TYRP donor cells to ensure the specificity of the sitagliptin-mediated effects are planned, as are experiments with human T cells with CD26 removed via CRISPR/Cas9 methods. This work will help define the antitumor role of CD26 in cells utilized for adoptive cell therapy.

**References**

1. Da Silva RB, Laird ME, Yatim N, Fiette L, Ingersoll MA, Albert ML. Dipeptidylpeptidase 4 inhibition enhances lymphocyte trafficking, improving both naturally occurring tumor immunity and immunotherapy. Nat Immunol. 2015;16(8):850-858.

2. Bailey SR, Nelson MH, Majchrzak K, Bowers JS, Wyatt MM, Smith AS, Neal LR, Shirai K, Carpenito C, June CH, Zilliox MJ. Human CD26 high T cells elicit tumor immunity against multiple malignancies via enhanced migration and persistence. Nat Commun. 2017;8(1):1961.

**Ethics Approval**

The study was approved by the Institutional Animal Care and Use Committee at the Medical University of South Carolina, approval number 3039.

#### P285 A clinical study on CD33-directed chimeric antigen receptor modified NK cells

##### Qiao Li, PhD^4^, Leiming Xia^1^, Jing Zhang^2^, Liu Liu^1^, Bin Li^1^, Tan Li^1^, Yi Wang^1^, Lin Yang^3^, Yangyi Bao^1^, Alfred Chang^4^, Max Wicha, MD^4^

###### ^1^The First People’s Hospital of Hefei, Hefei, China; ^2^University of Michigan Rogel Cancer Center; ^3^Persongen Biomedicine (Suzhou) Co., Ltd, Suzhou, China; ^4^University of Michigan Rogel Cancer Cent, Ann Arbor, USA

####### **Correspondence:** Qiao Li (qiaoli@med.umich.edu)

**Background**

About 20-40% of the patients with acute myeloid leukemia (AML) show no response to current therapy, and 50-70% of the patients relapse after complete response. These patients are categorized as refractory and relapsed (R/R) AML. The 1-year survival rate of these patients is limited to <30% and further dropped to approximately 10% for 5- year survival. More effective treatment for this disease is urgently needed. As a novel immune cell-based therapy, chimeric antigen receptor (CAR) modified NK (CAR-NK) cell treatment may represent a promising strategy in treating hematological malignancies.

**Methods**

Higher CD33 is expressed on both AML cells and leukemia stem cells (LSCs), compared with normal hematopoietic stem cells. Therefore, CD33-directed CAR-NK (CD33-CAR-NK) treatment may lead to the development of a novel strategy for R/R AML. In this clinical study, three patients with R/R AML expressing high levels of CD33 (40-70%) were enrolled. Two were M1 subtypes, and the other was M2a subtype. All three patients had received a series of standard chemotherapy regimen with no significant therapeutic effect before 3×109 allogeneic CD33-CAR-NK cells were infused. 1×109 CD33-CAR-NK cells were infused every other day for a total of three infusions.

**Results**

After CD33-CAR-NK cell infusion, the main side effects include repeated low-grade fever and significant rises of serum cytokines, e.g. IFN-γ, TNF-α, IL-2, IL-6 and IL-10. However, these cytokines reduced to normal level before discharge. In addition, we detected serum K+, Ca2+, creatinine and glutamic-pyruvic transaminase after CD33- CAR-NK infusion, and found none of these three indexes was significantly changed, compared with those before the CD33-CAR-NK infusion. After CD33-CAR-NK cell infusions, the proportion of leukemic cells in the patients’ bone marrow significantly decreased from 40% to 26%; the proportion of CD33+ cells in bone marrow reduced from 48.82% to 32.56%, and the proportion of CD33+CD34+ cells in bone marrow reduced from 40.59% to 27.26% respectively in a 53 years old male patient with AML-M1.

**Conclusions**

This initial clinical work indicates that CD33-CAR-NK therapy may offer a new therapeutic option for R/R AML, which aims to reduce the tumor burden, eliminate LSCs, and provide opportunity to combine it with other therapies for more effective treatment of the R/R AML patients.

**Acknowledgements**

Medicine and technique Foundation of Hefei city, China; Longenbaugh Foundation, USA.

**Trial Registration**

NCT02944162.

**Ethics Approval**

This study was approved by the Ethics Board of the First People’s Hospital of Hefei, China, approval number 2016-001-01.

#### P286 Combination of PD-1 blockade and RetroNectin-activated cytokine-induced killer cells in pre-heavily treated NSCLC: a retrospective study

##### Lingdi Zhao^1^, Lu Han^2^, Yong Zhang^2^, Tiepeng Li^2^, Yonghao Yang^2^, Wei Li^2^, Yiman Shang^2^, Hongwei Lin^2^

###### ^1^Affiliated Cancer Hospital of Zhengzhou University & Henan Cancer Hospital, Zhengzhou, Peoples Republic of China; ^2^Affiliated Cancer Hospital of Zhengzhou, Zhengzhou, China

####### **Correspondence:** Lingdi Zhao (gaoquanli2015@126.com)

**Background**

Lung cancer is one of the most common malignancies worldwide, with high morbidity and mortality. Great advances have been made recently in the treatment of advanced non-small cell lung cancer (NSCLC); however, almost all patients eventually fail after first- or second-line therapy. There is no standard regimen for patients with advanced NSCLC after failure of second-line therapy. Therefore, it is of great importance to explore treatment regimens for these patients.

**Methods**

We retrospectively analyzed patients with advanced NSCLC who received anti-programmed cell death protein (PD)-1 antibody combined with RetroNectin activated cytokine-induced killer (R-CIK) cells after failure of at least two regimens from October 2015 to April 2018 in our department. A total of seven patients were included in this study. Descriptive statistics were used to summarize the patients’ characteristics, treatment-related adverse events (AEs), overall responses, and R-CIK cell phenotypes. Survival was calculated using the Kaplan-Meier method.

**Results**

Of the seven patients, five were men and two were women. The median age was 54 years (range 42-64 years). The median number of previous treatment regimens was 3 (range 2-7). Partial remission was achieved in two patients, stable disease in four patients, and one patient experienced progressive disease. The median time-to-progression was 4.8 months. At present, there are four patients still alive, and the median overall survival has not been reached. During treatment, grade 2 fever occurred in two patients and grade 2 interstitial pneumonia in one patient; no AEs over grade 2 occurred.

**Conclusions**

PD-1 blockade combined with R-CIK cells is safe and effective in patients with advanced NSCLC who have failed at least two treatment regimens.

### Clinical Trials (Completed)

#### P287 HyPeR: A phase 1, dose escalation study of Guadecitabine (SGI-110) a second generation hypomethylating agent in combination with Pembrolizumab (MK3475) in patients with refractory solid tumours

##### Malaka Ameratunga, MBBS^1^, Maxime Chénard-Poirier^2^, Sanjena Mithra^3^, Ricardo Morilla^4^, Ruth Riisnaes^4^, Penny Flohr^4^, Rita Pereira, BSc^4^, Ana Ferreira, MSc^4^, Ines Figueiredo^4^, Suzanne Carreira^4^, Claudia Bertan^4^, Wei Yuan^4^, David Dolling^4^, Mateus Crespo^4^, Bora Gurel^4^, Bob Brown^5^, Naina Patel^5^, Joanna Dawes^4^, Toby Prout^4^, Mona Parmar^4^, Alison Turner^4^, Holly Tovey^1^, Emma Hall^1^, Anna Minchom^1^, Udai Banerji^1^, Nina Tunariu^1^, Juanita Lopez^1^, Dionysis Papadatos-Pastos^6^

###### ^1^Royal Marsden Hospital, Sutton, UK; ^2^CHU de Québec - Université Laval, Sutton, UK; ^3^University College London Hospitals, London, UK; ^4^Institute of Cancer Research, London, UK; ^5^Imperial College London, London, UK; ^6^University College Hospitals London, London, UK

####### **Correspondence:** Malaka Ameratunga (johann.de-bono@icr.ac.uk)

**Background**

Guadecitabine is a second-generation DNA hypomethylating agent which induces epigenetic expression of immune related genes and increases the infiltration of interferon-producing T-cells into tumours; it can potentially enhance the anti-cancer activity of pembrolizumab [1,2]. The aim of this investigator-initiated study is to evaluate the safety and efficacy of guadecitabine in combination with pembrolizumab and identify the recommended phase 2 dose (RP2D).

**Methods**

Patients with advanced solid tumours, refractory to standard therapy received guadecitabine via subcutaneous injection on days 1-4 and pembrolizumab intravenously on day 1 of each 21-day cycle following a 4-week sensitising run-in period with guadecitabine. The study used a 3+3 design, with a dose-finding cohort (Part A) followed by an ongoing expansion cohort (Part B) at the RP2D and includes correlative pharmacokinetic and pharmacodynamic studies. Paired pre- and post-treatment tumour biopsies are to be evaluated for PD-L1 expression, tumour infiltrating lymphocytes, gene expression by RNAseq and methylome studies. Longitudinal analysis of peripheral blood CD3, CD4 and CD8 lymphocytes by flow cytometry using a multi-level mixed effect model was performed. The trial was reviewed by a central research ethics committee (REC#16/LO/1605).

**Results**

Following completion of Part A, 12 evaluable patients (2 mesothelioma, 2 PD-L1 negative NSCLC, 2 cholangiocarcinoma, 2 cervical, 2 breast, an ovarian and colorectal cancer) have been treated (6 each at 45mg/m2 and 30mg/m2 guadecitabine). Two DLTs (neutropenia, febrile neutropenia) were reported at 45mg/m2, whereas none were at 30mg/m2. The most common toxicities were reversible neutropenia, fatigue and abdominal pain. The recommended Phase II doses (RP2D) were 30mg/m2 for guadecitabine days 1-4 with 200mg IV pembrolizumab q21 days. Overall, 3 patients (colorectal cancer, NSCLC, cervical cancer) had prolonged stable disease > 6 months’ duration. A patient with cervical adenocarcinoma had a 69% drop in Ca125. A patient with PD-L1 negative NSCLC with a STK11 aberration (associated with resistance to PD-1 inhibition [3]) had a PFS of 8 months. (Figure 1) A further patient with PD-L1 negative NSCLC was taken off study for PD, but on follow-up CT demonstrated a partial response, in the absence of intervening therapy. Longitudinal analysis of peripheral blood lymphocytes by flow cytometry over time showed evidence of increasing CD3 counts by 0.16%/day (p=0.03) and for CD4 by 0.17%/day (p=0.007), but not CD8 counts. Enrollment in the expansion phase is ongoing.

**Conclusions**

The RP2D of this combination is 30mg/m2 guadecitabine D1-4 and pembrolizumab 200mg q21 with evidence of biological and anti-tumour activity.

**Trial Registration**

Eudra-CT 2016-000760-41

**References**

1. Coral S, Parisi G, Nicolay HJ, et al. Immunomodulatory activity of guadecitabine, a 5-aza-2'-deoxycytidine- containing demethylating dinucleotide. Cancer Immunol Immunother.2013;62:605-14

2. Wang LX, Mei ZY, Zhou JH, Yao YS, Li YH, Xu YH, et al. Low dose decitabine treatment induces CD80 expression in cancer cells and stimulates tumour specific cytotoxic T lymphocyte responses. PLoS One. 2013;8(5):e62924

3. Skoulidis, Ferdinandos, et al. “STK11/LKB1 Mutations and PD-1 Inhibitor Resistance in KRAS-Mutant Lung Adenocarcinoma.” Cancer discovery (2018): CD-18.

**Ethics Approval**

The trial was reviewed by a central research ethics committee (REC#16/LO/1605).

**Fig. 1 (abstract P287).**
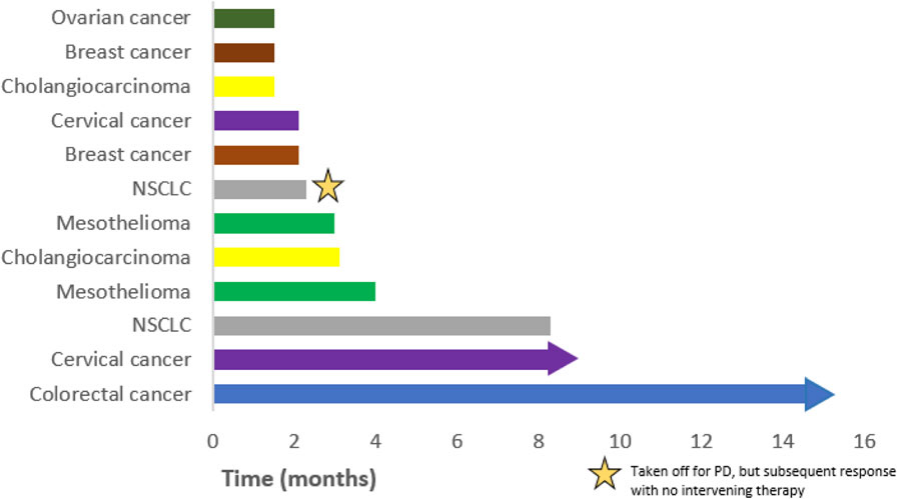
Swimmer's plot

#### P288 IMAGE 1 (Immune Modulation And Gemcitabine Evaluation 1), a randomized, open-label phase II trial comparing gemcitabine with and without IMM-101 in advanced pancreatic cancer

##### Angus Dalgleish, MD, PhD (dalgleis@sgul.ac.uk)

###### St. George's University of London, London, UK

**Background**

The effectiveness of immunotherapy for pancreatic cancer is limited by the impenetrable nature of the primary tumor and the complex mix of cellular and biochemical negative regulators in the tumor microenvironment. To complement previously published encouraging results from the IMAGE 1 trial [1], we present here updated results to include the long- term follow-up of this study.

**Methods**

Patients (n=110) with advanced pancreatic cancer and WHO performance status 0-2 were allocated randomly to receive IMM 101 (a myeloid dendritic cell activator containing heat-killed Mycobacterium obuense [NCTC 13365]) + gemcitabine (Gem) (n=75) or Gem alone (n=35) for a 12-cycle maximum. Twelve of the thirteen patients who completed this protocol, (11 IMM-101 + Gem, 1 Gem alone) entered a follow-up sub-study to monitor long-term survival and tolerability, all receiving IMM 101 with or without adjunctive chemotherapy.

**Results**

Median overall survival (OS) was longer for patients from the IMM-101 + Gem group (6.7 v. 5.6 months, hazard ratio [HR] 0.67, 95% confidence interval [CI] 0.44 to 1.04; log rank p-value 0.0706) with the OS increase more pronounced for the pre-defined subset of patients (n=92) with metastatic disease (7.0 v. 4.4 months, HR 0.53, 95% CI 0.33 to 0.86; log rank p-value 0.0093). Longer follow-up of this metastatic group, including data from the sub- study, revealed survival probabilities for the IMM-101 treated group of 18.3% at 18 months, 11.4% at 24 and 30 months and 5.7% at 36 months. The Gem-alone group had a survival probability of 2.3% at 18 months with no patients with survival data beyond this time point. Extended exposure to IMM-101 did not appear to compromise tolerability with maximum exposure of 46.5 months observed.

**Conclusions**

First line IMM-101 in combination with Gem produced extended survival benefits for metastatic pancreatic cancer patients compared to Gem alone. The survival probabilities beyond 24 months are comparable to those seen with the nab-paclitaxel-Gem combination [2], but without any notable incremental toxicity burden.

**Acknowledgements**

Funded by Immodulon Therapeutics, London, UK

**Trial Registration**

IMAGE 1: EUDRACT Number: 2010-022757-42; multiple ethics committee approvals obtained in accordance with requirements of participating countries and trial centers.

**References**

1. Dalgleish AG, et. al. Randomised, open-label phase II study of gemcitabine with and without IMM-101 for advanced pancreatic cancer. Br J Cancer 2016; 115, 789-796.

2. Goldstein D, et. al. nab-Paclitaxel plus gemcitabine for metastatic pancreatic cancer: Long-term survival from a phase III trial. J. Natl. Cancer Inst. 2015; 107 (2), dju413.

**Ethics Approval**

multiple ethics committee approvals obtained in accordance with requirements of participating countries and trial centers.

#### P289 Initial results from a phase 1a/b study of etigilimab (OMP-313M32), an anti-T cell immunoreceptor with Ig and ITIM domains (TIGIT) antibody, in advanced solid tumors

##### Sunil Sharma, MD^1^, Kathleen Moore^2^, Niharika Mettu^3^, Ignacio Garrido-Laguna^4^, Susanna Ulahannan, MD, MMed^2^, Vivek Khemka, MD^1^, Ann Kapoun^5^, Leonardo Faoro^6^

###### ^1^Honor Health Research Intitute, Scottsdale, AZ, USA; ^2^University of Oklahoma, Oklahoma City, OK, USA; ^3^Duke University, Durham, NC, USA; ^4^University of Utah, Salt Lake City, UT, USA; ^5^OncoMed Pharmaceuticals, Redwood City, CA, USA; ^6^Oncomed Pharmaceuticals Inc, Redwood City, CA, USA; ^7^Sarah Cannon Research Institute, Nashville, TN, USA

####### **Correspondence:** Sunil Sharma (jbendell@tnonc.com)

**Background**

TIGIT is an immune-checkpoint expressed on T and NK cells. Etigilimab is a novel IgG1 anti-TIGIT antibody that has inhibitory as well as ADCC characteristics. Anti-TIGIT demonstrates preclinical in-vivo anti-tumor effects as a single agent and with anti-PD-1. Initial results from the phase 1a dose escalation portion of the study are presented.

**Methods**

This study enrolled subjects with advanced solid tumors into either a Ph 1a single-agent portion (dose escalation in all comers + expansion in selected tumor types) or a Ph 1b combination [PD-(L)1 refractory] portion with nivolumab (dose escalation). Objectives included safety, maximum tolerated dose (MTD), determining the recommended Ph 2 dose (RP2D), pharmacokinetics, immunogenicity, efficacy and biomarkers. Dose escalation followed a modified 3+3 framework.

**Results**

18 subjects were treated in the dose escalation portion of the Phase 1a with doses ranging from 0.3 to 20 mg/kg Q2W. Tumor types included colorectal cancer (6), endometrial cancer (3), pancreatic cancer (3), and 6 other tumor types (1 each). No dose-limiting toxicities were observed; thus, the RP2D was 20 mg/kg Q2W. The most frequent treatment-related AEs were rash (33.3%), fatigue (16.7%), nausea (16.7%), pruritus (16.7%), and cough (11.1%). Immune-related adverse events included rash (33.3%), pruritus (16.7%), autoimmune hepatitis (5.5%) and stomatitis (5.5%). Grade 3 or higher treatment-related AEs included rash (16.7%), and fatigue, hypophosphatemia, and autoimmune hepatitis (5.5% each). Four (22%) subjects had stable disease as best response (longest durations were 210 and 226 days), 12 had progressive disease, and 2 were not evaluable. The expansion cohort in phase 1a and dose-escalation in phase 1b are ongoing. Updated data, including PK and biomarker data will be presented.

**Conclusions**

TIGIT is a potential therapeutic target against cancer. Etigilimab has been well tolerated at doses up to 20 mg/kg Q2W. Evidence of immune activation was shown in multiple subjects with immune-related AEs. Early signs of potential efficacy have been observed in subjects with prolonged stable disease.

**Trial Registration**

clinicaltrials.gov NCT03119428

**Ethics Approval**

The study was approved by the Institutional Review Boards of all participating institutions.

#### P290 Immunologic biomarkers in a multi-center, single arm, open label Phase II clinical trial of mFOLFOX6 and pembrolizumab in patients with advanced colorectal cancer

##### Matthew Farren, PhD^1^, Yan Tong^2^, Ziuye Liu^2^, Bert O'Neil^2^, Tanios Bekaii-Saab, MD^3^, Anne Noonan^4^, Christopher McQuinn, MD^5^, Thomas Mace, PhD^5^, Walid Shaib, MD^1^, Christina Wu, MD^1^, Bassel El-rayes, MD^1^, Safi Shahda, MD^2^, Matthew Farren, PhD^1^

###### ^1^Emory University, Atlanta, GA, USA; ^2^Indiana University, Indianapolis, IN, USA; ^3^Mayo Clinic, Phoenix, AZ, USA; ^4^Ohio State University, Columbus, OH, USA; ^5^The Ohio State University, Hilliard, OH, USA

####### **Correspondence:** Matthew Farren (gregory.b.lesinski@emory.edu)

**Background**

Colorectal cancer (CRC) accounts for an estimated 135,000 new cases and 50,000 deaths annually in the USA.

Although PD-1/PD-L1-targeted immune checkpoint blockade has survival benefit in CRC patients with microsatellite instable (MSIhigh) tumors, most patients remain refractory to this therapy. We hypothesized that chemotherapy might potentiate the clinical response to pembrolizumab and improve outcomes in CRC.

**Methods**

Advanced/recurrent CRC patients received pembrolizumab (200mg IV every 3 weeks) and mFOLFOX6 (oxaliplatin, leucovorin, and 5′fluorouracil) on days 1 and 15 of a 28-day cycle. To assess the immunological impact of treatment, peripheral blood was collected at baseline and prior to treatment on cycle 1 day 15 (C1D15), on C3D1, and at the end of treatment. Plasma and leukocytes were isolated and cryopreserved. 40 soluble factors (via bioplex) and >200 immune cell phenotypes and populations (via FACS) were characterized. All studies were IRB-approved and registered on clinicaltrials.gov (NCT02375672).

**Results**

Patients on this trial experienced a median progression free survival (PFS) of 7.4 months, and a median overall survival (OS) of 17.7 months. Patients were dichotomized based on outcome (i.e. greater-than or less-than median PFS or OS) and analyzed to determine how baseline levels of, or changes in, individual (univariate) soluble biomarkers or immune cell phenotypes were associated with patient outcome. Our analysis found that patients who experienced greater OS had significantly greater baseline levels of circulating cytokines that could broadly be described as pro-inflammatory, such as IL-2 (p=0.0366) or CD40L (p=0.0045), among others. Moreover, these patients had a greater relative frequency of circulating CD8+ T cells (compared to CD4+) at baseline (p=0.0181) and, perhaps more interestingly, had a lower proportion of naïve CD4+ and CD8+ T cells at baseline (p=0.0131 and 0.0437, respectively). This suggests greater responsiveness to FOLFOX + pembrolizumab among patients with a greater preexisting anti-tumor immune response. Conversely, patients with higher baseline frequencies of circulating regulatory T cells experienced shorter OS (p=0.0343), as did those patients whose circulating levels of VEGF increased over the course of treatment (p=0.0276), arguing that preexisting or emergent immunosuppression antagonized treatment efficacy. Ongoing analyses are exploring additional hypotheses related to longitudinal immunologic changes and impact upon biomarkers of immunogenic cell death relative to clinical outcomes.

**Conclusions**

Overall, our data identifies several potential immune mediators through which chemotherapy acts to potentiate the response to immunotherapy in CRC and several immunological predictors for benefit from combined pembrolizumab + FOLFOX therapy.

**Trial Registration**

ClinicalTrials.gov registry number NCT02375672

**Ethics Approval**

This study was approved by the Indiana University IRB. Protocol number 1502701251A010

#### P291 A targeted modified IL-2 (NHS-IL2) combined with stereotactic body radiation therapy (SBRT) in patients with advanced melanoma refractory to checkpoint inhibitors: Results from a Phase IIa trial

##### Omid Hamid, MD^1^, David Lawson, MD^2^, Karl Lewis, MD^3^, Jose Lutzky, MD, FACP^4^, C. Lance Cowey^5^, Beatrice Brunkhorst^6^, Meng Li^6^, Wanping Geng^6^, Christine Hicking^7^, Vijay Kasturi, MD^6^, Howard Kaufman^8^, Lisa Jolly^9^

###### ^1^The Angeles Clinic and Research Institute, Los Angeles, CA, USA; ^2^Emory University, Atlanta, GA, USA; ^3^University of Colorado, Aurora, CO, USA; ^4^Mount Sinai Comprehensive Cancer Center, Miami Beach, FL, USA; ^5^Baylor Taylor Charles A. Sammons Cancer, Dallas, TX, USA; ^6^EMD Serono, Inc, Billerica, MA, USA; ^7^Merck KgaA, Darmstadt, Germany; ^8^Massachusetts General Hospital, Boston, MA, USA; ^9^Bioscript Group, Macclesfield, Cheshire, UK

####### **Correspondence:** Omid Hamid (ohamid@theangelesclinic.org)

**Background**

Benefits of IL-2 in cancer patients are limited by toxicity. NHS-IL2 is a fusion protein comprising IL-2, modified to bind high-affinity IL-2 receptors, and a human IgG2 monoclonal antibody (NHS76) targeting DNA from necrotic tumor cells. Phase I studies demonstrated a tolerable safety profile, and preclinical studies demonstrated activity as monotherapy and in combination [1–3]. We report data from a Phase IIa open-label, parallel-group dose-escalation trial designed to determine the maximum tolerated dose (MTD) and tolerability of NHS-IL2 combined with SBRT in patients with advanced melanoma refractory to checkpoint inhibition (NCT01973608).

**Methods**

Adults with unresectable/metastatic melanoma who failed ipilimumab +/- anti-PD1, and ECOG PS 0–1 were randomized to one of three dose groups: low (0.3mg/kg), intermediate (1.0mg/kg), and high (dose escalation: 3+3 design 1.8–3.6mg/kg, dose-expansion at MTD or 3.6mg/kg). Patients received NHS-IL2 (1-hour intravenous infusion) 4 days after SBRT to a single lesion in 21-day cycles until disease progression, unacceptable toxicity, or consent withdrawal. Primary objective: MTD of NHS-IL2 + SBRT. Exploratory objectives included efficacy (best overall response [BOR] by RECIST and immune-related [ir]RECIST), safety, immunohistochemistry, pharmacokinetics, pharmacodynamics, biomarker assessments. Paired biopsies were obtained and CD8+/FOXP3- positive cells were counted manually. Peripheral T-cell subsets were assessed by flow cytometry.

**Results**

Twelve patients were treated before early study termination (June 2015, reassessment of pipeline priorities; 0.3mg/kg n=2; 1.0mg/kg n=2; 1.8mg/kg n=6; 2.4mg/kg n=2). MTD was not reached; one patient (1.8mg/kg) had a dose-limiting toxicity (angioedema). Serious drug-treatment-emergent adverse events (AEs) occurred in 4/12 patients (1.8mg/kg n=3; 2.4mg/kg n=1); none were considered study drug-related, none were fatal. No severe cardiovascular AEs were observed, no new risks were identified. BOR was unevaluable in 4/12 patients. BOR was SD in 5/12 patients (four patients with disease control ≥4.3 months; 3/5 patients with previous ipilimumab + anti- PD1 treatment) and PD in 3/12 by irRECIST. BOR was SD in 2/12 patients and PD in 6/12 by RECIST. Serum NHS-IL2 exposure increased dose-proportionally up to 2.4mg/kg; t1/2 was 8–16 hours, with no significant accumulation following multiple doses. Changes in soluble serum factors, PBMC subsets, gene signatures, and immune cell infiltration upon NHS-IL2 treatment will be presented.

**Conclusions**

NHS-IL2 combined with SBRT had acceptable safety and tolerability, and demonstrated disease control in heavily pretreated patients with advanced melanoma who failed ipilimumab +/- anti-PD1 without the toxicities often associated with IL-2 treatment. Further evaluation of NHS-IL2, including combination with immune checkpoint inhibitors, may be warranted.

**Acknowledgements**

Medical writing assistance, provided by Lisa Jolly (Bioscript Science, Macclesfield, UK) was funded by Merck KGaA, Darmstadt, Germany.

**Trial Registration**

NCT01973608

**References**

1. Gillies SD, Lan Y, Hettmann T, Brunkhorst B, Sun Y, Mueller SO, Lo KM. A low-toxicity IL-2-based immunocytokine retains antitumor activity despite its high degree of IL-2 receptor selectivity. Clin Cancer Res. 2011;17:3673-3685.

2. Gillessen S, Gnad-Vogt US, Gallerani E, Beck J, Sessa C, Omlin A, Mattiacci MR, Liedert B, Kramer D, Laurent J, Speiser DE, Stupp R. A phase I dose-escalation study of the immunocytokine EMD 521873 (Selectikine) in patients with advanced solid tumours. Eur J Cancer. 2013;49:35–44.

3. van den Heuvel MM, Verheij M, Boshuizen R, Belderbos J, Dingemans AM, De Ruysscher D6, Laurent J, Tighe R, Haanen J, Quaratino S. NHS-IL2 combined with radiotherapy: preclinical rationale and phase Ib trial results in metastatic non-small cell lung cancer following first-line chemotherapy. J Trans Med. 2015;13:32.

**Ethics Approval**

This study was approved by Copernicus IRB (CGIRB) approved under IRB Tracking #: QUI1-13-405 .

#### P292 Changes in intra- and perinodular heterogeneity patterns on serial computed tomography are associated with overall survival in Nivolumab-treated non-small cell lung cancer

##### Mohammadhadi Khorrami, PhD^1^, Prateek Prasanna, PhD^1^, Anant Madabhushi, PhD^1^, Mohammadhadi Khorrami, PhD^1^

###### ^1^Case Western Reserve University, Cleveland, OH, USA; ^2^Cleveland Clinic, Pepper Pike, OH, USA

####### **Correspondence:** Mohammadhadi Khorrami (velchev@ccf.org)

**Background**

Programmed cell death (PD)-1 immune checkpoint inhibitors have been approved to treat stage III unresectable, or stage IV metastatic Non-Small Cell Lung Cancer (NSCLC) patients. There are no validated clinical biomarkers to identify the patients who are likely to derive benefit from checkpoint inhibitor therapy such as Nivolumab. Apart from changes in RECIST measurements, changes in other image characteristics post immunotherapy are poorly understood. Previous studies have shown that more heterogeneous tumors with irregular patterns of intensities have a better overall survival in patients treated with Nivolumab. In addition to heterogeneity in PD-L1 status, other micro-environmental factors like heterogeneous and inefficient vasculature which contributes to regions of hypoxia and acidosis may cause treatment failure. These characteristics may manifest as distinct patterns within and around nodules on computed tomography (CT) images. Our work shows that changes between baseline, and post-treatment radiomic textural features within nodules and surrounding habitat on CT are associated with overall patient survival (OS).

**Methods**

Non-contrast CT scans from 73 NSCLC patients, with pre- and 2-week post-Nivolumab treatment, were acquired retrospectively from Cleveland Clinic Foundation. 454 intra-nodular texture features as well as 7426 features from annular rings around the nodule (0-30mm from nodule boundary) were extracted from the baseline and post- treatment temporal CT scans. The normalized differences in feature statistics across the two time-points were then computed to yield a set of ‘delta-radiomic’ descriptors. Cox proportional hazard model was employed to evaluate the ability of the features in predicting OS in a univariate and multivariate setting. In addition, Kaplan–Meier survival analysis and log-rank statistical tests were performed to assess the discriminative ability of the features.

**Results**

A multivariate Cox regression analysis indicated that intranodular Laws Energy (Hazard ratio (HR): 1.924; 95% CI: 1.220, 3.038; log-rank p = 0.0049), intranodular Haralick sum average (HR: 4.06; 95% CI: 1.890, 8.738; p = 0.00033), intranodular Haralick difference average (HR: 1.098; 95% CI: 1.0256, 1.177; p = 0.0074) and perinodular Laws Energy (HR: 1.86; 95% CI: 1.049, 3.318; p = 0.017) were predictors of OS, while no significant differences were observed in age or gender. The concordance index (CI) of the multi-variate radiomic model was 0.70. Figure 1 shows KM curve using Linear Discriminate Analysis (LDA) classifier and 3-fold cross validation for top 4 features.

**Conclusions**

Our results suggest that changes in certain radiomic texture features between baseline and post-treatment CT scans following Nivolumab could potentially identify overall survival in NSCLC patients.

**Fig. 1 (abstract P292).**
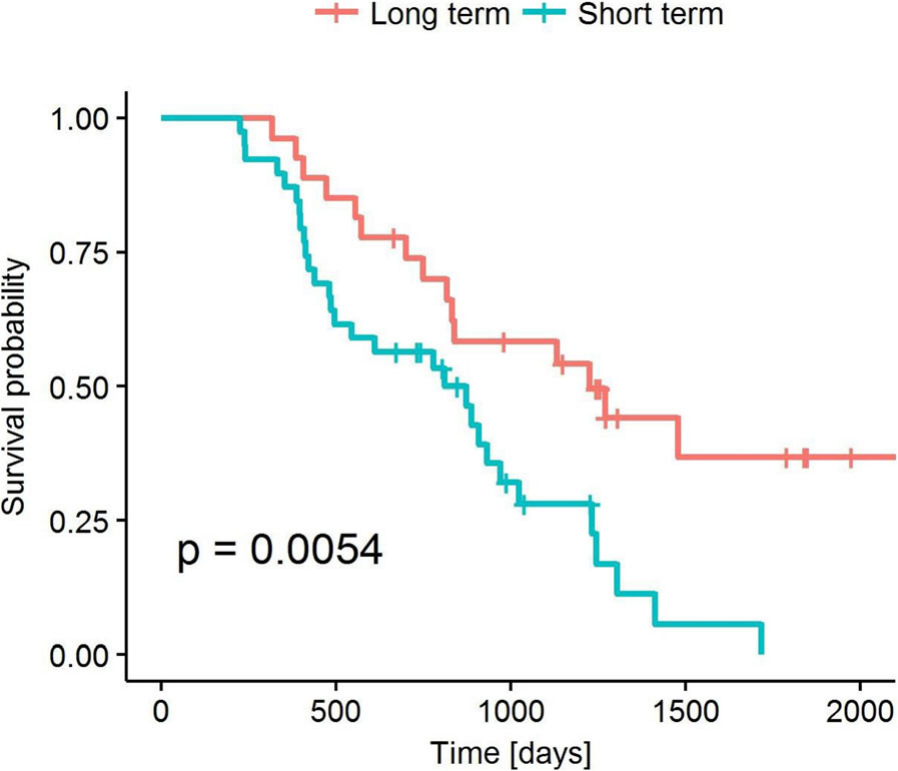
See text for description.

#### P293 Autologous tumor cell vaccination combined with systemic CpG-B and IFNα promotes activation of dendritic cells and T cells and induces clinical responses in metastatic Renal Cell Carcinoma

##### Bas Koster, MD^1^, Saskia Santegoets^2^, Jorien Harting, MD^1^, Arnold Baars, MD, PhD^1^, Marieke van Ham, PhD^3^, Rik Scheper, PhD^1^, Erik Hooijberg, PhD^4^, Tanja de Gruijl, PhD^1^

###### ^1^Amsterdam UMC, Cancer Center Amsterdam, Amsterdam, Netherlands; ^2^Leiden University Medical Center, Leiden, Netherlands; ^3^Sanquin Research, Amsterdam UMC, Amsterdam, Netherlands; ^4^Netherlands Cancer Institute, Amsterdam, Netherlands

####### **Correspondence:** Bas Koster (vandeneertwegh@vumc.nl)

**Background**

Currently, immune checkpoint blockade is overtaking targeted therapy as first-line treatment of metastatic Renal Cell Carcinoma (mRCC). However, a majority of patients still fails to respond. It has become clear that response to immune checkpoint inhibitors relies on T cells reactive to highly individualized neo-antigens. In this light, there is a resurgence in interest for autologous tumor vaccination approaches. In this study the toxicity and efficacy of vaccination with irradiated autologous tumor cells in combination with a class B CpG oligodeoxynucleotide (CpG- B, CPG7909) and Granulocyte-macrophage colony-stimulating factor (GM-CSF) followed by systemic CpG-B and interferon-α (IFNα) administration was examined in patients with mRCC.

**Methods**

A single-arm Phase II trial was conducted, in which patients with mRCC were intradermally vaccinated with a minimum of three whole-cell vaccines containing 0.7 – 1.3 x 10^7 irradiated autologous tumor cells (ATC), admixed with 1 mg CpG-B and 100 μg GM-CSF, followed by bi-weekly subcutaneous (SC) injections with 8 mg CpG-B and SC injections with 6 MU IFNα three times per week.

**Results**

Fifteen patients were treated according to the protocol. Treatment was well tolerated. Objective clinical responses occurred in three patients, including one long-term complete response and two partial responses. Disease stabilization occurred in another three patients. Positive delayed type hypersensitivity (DTH) responses to ATC were absent before the treatment but present in 13 out of the 15 patients during treatment, suggestive for a treatment induced specific antitumor response. Further immune monitoring on circulating mononuclear cells revealed that treatment resulted in activation of plasmacytoid dendritic cells, non-classical monocytes and upregulation of both PD-1 and CTLA4 on effector T cells. Moreover, a pre-existing ex vivo IFNγ response to ATC was associated with clinical response on treatment.

**Conclusions**

Autologous tumor cell vaccination combined with systemic CpG-B and IFNα is tolerable, safe, immunogenic and able to elicit anti-tumor responses in patients with advanced RCC. Immune activation and treatment-induced up- regulation of PD-1 and CTLA4 on circulating T cells further suggest an added benefit of combining this approach with immune checkpoint blockade.

**Ethics Approval**

The study was approved by the Institutional Review Board of the VU University Medical Center, approval number

2003-37.

#### P294 Confirmatory study validates a MALDI prognostic signature for IL-2 response and the adverse prognostic role of the serum apoptotic marker Hepatocyte Growth Factor (HGF) in Renal Cell Carcinoma (RCC)

##### Michael Lotze, MD^1^, Shuyan Zhai, PhD^1^, Daniel Normolle, PhD^1^, Ryan Sullivan, MD^2^, Heinrich Roder, DPhil^3^, Joanna Roder, PhD^4^, David McDermott, MD^5^, Theodore Logan, MD^6^, Marc Ernstoff, MD^7^, Thomas Olencki, DO^8^, David Friedland, MD^1^, Rahul Parikh, MD, PhD^1^, Jodi Maranchie, MD^1^, Mary Jo Buffo, BS^9^, Lisa Butterfield, PhD^1^

###### ^1^University of Pittsburgh, Pittsburgh, PA, USA; ^2^Massachusetts General Hospital, Needham, MA, USA; ^3^Biodesix, Inc., Boulder, CO, USA; ^4^Biodesix, Inc, Steamboat Springs, CO, USA; ^5^Beth Israel Deaconess, Boston, MA, USA; ^6^University of Indiana, Indianapolis, IN, USA; ^7^Dartmouth University, Buffalo, NY, USA; ^8^Ohio State University, Columbus, OH, USA; ^9^University of Pittsburgh Medical Center, Pittsburgh, PA, USA; ^10^UPMC Hillman Cancer Center, Pittsburgh, PA, USA

####### **Correspondence:** Michael Lotze (applemanlj@upmc.edu)

**Background**

Aldesleukin (recombinant interleukin-2, IL-2) was FDA-approved for mRCC in 1992 with a 5-10% rate of durable CRs and 25% ORR. Hydroxychloroquine (HCQ) inhibits autophagy, promoting tumor apoptosis. In murine models, IL-2 and HCQ is associated with diminished toxicity and increased efficacy [1]. We hypothesized that a MALDI mass spectrometric signature associated with response to IL-2 in melanoma could stratify outcomes in RCC and that the adverse role of HGF [2], an apoptotic marker [3] promoting autophagy [4] could be confirmed.

**Methods**

We studied high-dose IL-2 in combination with oral HCQ for patients with mRCC. Patients (pts) received IL-2, 600,000 IU/kg, every 8 hours up to 14 doses/cycle. HCQ was administered orally, starting 2 weeks prior to IL-2 and continued up to one year. The HCQ dose was 600 mg (17pts) or 1200 mg (13pts) daily. MALDI mass spectra were generated for pretreatment (and on D1 after 14d HCQ) serum samples, blindly classified as Early/Late (poor/good outcomes, respectively), based on the melanoma algorithm. Pretreatment serum was also tested for HGF. Overall survival (OS) was compared between Early/Late groups and between high and low HGF (dichotomized at the median) using log-rank test and proportional hazards ratios (PHR).

**Results**

Of 30 patients in the study, 29 were evaluable for response. MALDI test classification and HGF (high vs low) were both univariate significant predictors of OS at Day 1 after IL-2. HGF also predicted OS at Day -14. Although MALDI test classification was associated with HGF level (Mann-Whitney p = 0.042 (Day-14) and p=0.021 (Day 1), an exploratory investigation of simultaneous stratification by both markers revealed that Early and low HGF and Late patients regardless of HGF level had very good OS, significantly better than Early patients and high HGF (p=0.002).Table 1. Classifiers MALDI and HGF PHR (95% CI) Log-rank pDay -14 HGF (high vs low) 10.516 (1.233-89.702) 0.009 MALDI test (Early vs Late) 4.998 (0.582-42.929) 0.104Day 1 HGF (high vs low) 12.581 (1.425-111.084) 0.005 MALDI test (Early vs Late) >100 (no deaths in late) 0.030

**Conclusions**

IL-2 plus HCQ was well tolerated and clinically active with encouraging PFS of >17 months at the 600 mg HCQ dose (>4x greater than historical controls). A MALDI test developed in melanoma predicting OS in mRCC was validated. The prognostic power of the apoptotic marker and cMET ligand, HGF was confirmed. A combination of both markers showed potential for improved stratification, requiring future validation.

**Trial Registration**

NCT01550367; approved as IRB 11-074.

**References**

1. Liang X, De Vera ME, Buchser WJ, Romo de Vivar Chavez A, Loughran P, Beer Stolz D, Basse P, Wang T, Van Houten B, Zeh HJ 3rd, Lotze MT. Inhibiting systemicautophagy during interleukin 2 immunotherapy promotes long-term tumor regression. Cancer Res. 2012 Jun 1;72(11):2791-801.

2. Tanimoto S, Fukumori T, El-Moula G, Shiirevnyamba A, Kinouchi S, Koizumi T, Nakanishi R, Yamamoto Y, Taue R, Yamaguchi K, Nakatsuji H, Kishimoto T, Izaki H, Oka N, Takahashi M, Kanayama HO. Prognostic significance of serum hepatocyte growth factor in clear cell renal cell carcinoma: comparison with serum vascular endothelial growth factor. J Med Invest. 2008 Feb;55(1-2):106-11.

3. Vogel S, Trapp T, Börger V, Peters C, Lakbir D, Dilloo D, Sorg RV. Hepatocyte growth factor-mediated attraction of mesenchymal stem cells for apoptoticneuronal and cardiomyocytic cells. Cell Mol Life Sci. 2010 Jan;67(2):295-303.

4. Wang Y, Liu J, Tao Z, Wu P, Cheng W, Du Y, Zhou N, Ge Y, Yang Z. Exogenous HGF prevents cardiomyocytes from apoptosis after hypoxia via up-regulating cell autophagy. Cell Physiol Biochem. 2016;38(6):2401-13.

**Ethics Approval**

The study was approved by the University of Pittsburgh Institution's Ethic Board as IRB 11-074.

#### P295 A Phase 1, open-label, multicenter, dose escalation study of mRNA-2416, a lipid nanoparticle encapsulated mRNA encoding human OX40L, for intratumoral injection to patients with advanced malignancies

##### Shilpa Gupta, MD^1^, Todd Bauer, MD^2^, Ryan Sullivan, MD^3^, Robert Andtbacka, MD, CM, FACS, FRCSC^4^, Ding Wang, MD^5^, Geoffrey Shapiro, MD, PhD^6^, Khanh Do, MD^6^, Kurt Schalper, MD, PhD^7^, Patricia Gaule, PhD^7^, Tal Zaks, MD, PhD^8^, Joshua Frederick, PhD^8^, Lisa Johansen^8^, Kristen Hopson, PhD^8^, William Randolph^8^, Sima Zacharek, PhD^8^, Robert Meehan, MD^8^, Antonio Jimeno, MD, PhD^9^

###### ^1^University of Minnesota, Minneapolis, MN, USA; ^2^Sarah Cannon at Tennessee Oncology, Nashville, TN, USA; ^3^Massachusetts General Hospital, Needham, MA, USA; ^4^University of Utah Huntsman Cancer, Salt Lake City, UT, USA; ^5^Henry Ford Health System, Detroit, MI, USA; ^6^Dana Farber Cancer Institute, Boston, MA, USA; ^7^Yale Cancer Center, New Haven, CT, USA; ^8^Moderna Therapeutics, Cambridge, MA, USA; ^9^University of Colorado Denver, Denver, CO, USA

####### **Correspondence:** Shilpa Gupta (guptash@umn.edu)

**Background**

Blocking co-inhibitory checkpoints has become a standard treatment in diverse solid and hematologic malignancies, however, checkpoint inhibitors alone are not sufficient to induce robust and durable tumor regressions in the majority of patients. Generating optimal anti-tumor T-cell responses requires T-cell receptor activation and co- stimulation, which may occur via ligation of tumor necrosis factor (TNF) receptor family members, such as OX40. The OX40 receptor (TNFRSF4, cluster of differentiation [CD]134) is expressed on activated immune effector cells such as T-cells and natural killer (NK) cells [1]. It’s ligand, OX40L, is a homo-trimeric transmembrane protein normally expressed on antigen-presenting cells upon immune stimulation [2]. Binding of OX40 by OX40L in the presence of a recognized antigen promotes the expansion CD4+ and CD8+ T-cells and enhances memory responses while inhibiting regulatory T cells. Induction of OX40L expression by tumor cells, or other cells presenting tumor antigens may trigger a specific cell-mediated immune response with systemic anti-tumor effects. mRNA-2416 is a novel mRNA-based therapy that encodes human OX40L. Durable tumor regressions have been observed in preclinical models treated with mRNA-2416 at both injected and un-injected distal tumor sites.

**Methods**

Patients with locally advanced, recurrent or metastatic solid malignancy or lymphoma were treated with mRNA-2416 in a standard 3+3 phase 1 dose escalation study to assess safety and efficacy of intratumoral injection every 2 weeks in 28-day cycles. Tumor biopsies were collected at screening and on-treatment from one of three timepoints/locations (C1D2 or C2D2 from injected tumor, or C1D22-28 from un-injected tumor). All paired biopsies are evaluated by multiplexed Quantitative Immunofluorescence (QIF) and by RNA sequencing to characterize OX40L expression, and biomarkers of adaptive anti-tumor immune response following treatment.

**Results**

As of the July 2018 data cut off, 23 patients have been treated (melanoma 8, sarcoma 5, HNSCC 3, ovarian 2, neuroblastoma 1, salivary 1, appendiceal 1, NSCLC 1, colorectal 1) during dose escalation at dose levels from 1-8mg. No dose limiting toxicities have been reported. Related adverse events (AEs) of at least grade 3 consisted of 2 serious AEs; a skin ulceration due to tissue defect after regression of injected tumor, and an injection related reaction. No related grade 4/5 AEs were reported. Increased expression of OX40L protein in both tumor and immune cells post-treatment was detected by preliminary QIF analyses.

**Conclusions**

mRNA-2416 is tolerable at all dose levels studied, and yielded productive expression of OX40L protein. Translational data continues to be collected and analyzed.

**Trial Registration**

NCT03323398

**References**

1. Compaan DM, Hymowitz SG. The crystal structure of the costimulatory OX40-OX40L complex. Structure. 2006;14(8):1321–1330.

2. Mallett S, Fossum S, Barclay AN. Characterization of the MRC OX40 antigen of activated CD4 positive T lymphocytes--a molecule related to nerve growth factor receptor. EMBO J. 1990;9(4):1063-1068.

**Ethics Approval**

This study was approved by each Institution's IRB. e.g. University of Minnesota approval number STUDY00000068.

#### P296 Withdrawn

#### P297 Systemic activation and polyclonal expansion of CD8 T cells in cancer patients on Pegilodecakin alone or in combination with anti-PD-1

##### Martin Oft, MD^4^, Aung Naing, MD, FACP^1^, Jeffrey Infante, MD^2^, Kyri Papadopoulos^3^, Ivan Chan, PhD^4^, Cong Shen^5^, Navneet Ratti, BS, MBA^6^, Bianca Rojo, MSc^4^, Karen Autio, MS, MD^5^, Deborah Wong, MD PhD^7^, Manish Patel, MD^8^, Patrick Ott, MD, PhD^9^, Gerald Falchook, MD^10^, Shubham Pant, MBBS^1^, Annie Hung, BA^4^, John Mumm, PhD^11^, Matthew Adamow^5^, Scott McCauley, BA^12^, Phillip Wong, PhD^5^, Peter Van Vlasselaer, PhD^13^, Joseph Leveque^4^, Edward Garon, MD^7^, Nizar Tannir, MD, FACP^1^, Martin Oft, MD^4^

###### ^1^MDACC, Houston, TX, USA; ^2^SCRI, Nashville, TN, USA; ^3^START, San Antonio, TX, USA;^4^ARMO BioSciences, Redwood City, CA; ^5^MSKCC, New York, NY, USA; ^6^ARMO, Redwood City, CA, USA; ^7^UCLA, Santa Monica, CA, USA; ^8^Florida Cancer Specialists, Sarasota, FL, USA; ^9^DFCI, Boston, MA, USA; ^10^SCRI at Health One, Denver, CO, USA; ^11^AROM BioSciences, Gaithersburg, MD, USA; ^12^ARMO BioScinces, Redwood City, CA, USA; ^13^Armo BioScience, Redwood City, CA, USA

####### **Correspondence:** Martin Oft (martin.oft@armobio.com)

**Background**

Immunooncology therapies aim to induce activation and expansion of tumor reactive CD8+ T cells. Tumors with low mutational burden have a reduced response rate to checkpoint inhibition, presumably due to a reduced number of tumor reactive T cells. Pegilodecakin induces the expansion of tumor reactive T cells in preclinical tumor models. Pegilodecakin (PEGylated IL-10 or AM0010) monotherapy has been reported to achieve 25% objective tumor responses (ORR) in intermediate to poor risk renal cell cancer (RCC) in median 4th line of treatment (LOT) (range 1-8) (Naing A et al, JCO, 2016).

**Methods**

Patients received pegilodecakin (daily self-administration) alone or combination with pembrolizumab or nivolumab (following standard schedule and dose). Samples were collected post written consent from patients enrolled in a multi-basket trial and analyzed in accordance with the IRB. Systemic and cellular immune responses were assessed by serum cytokine analysis, PBMC flow cytometry and T cell clonal analysis by TCR deep sequencing. Immune fluorescence (IF) or immunohistochemistry was performed on formaldehyde fixed archival, pretreatment biopsies and on-treatment biopsies (CD8, granzyme B, phospho-STAT-3 or LAG-3, T-bet/CD3, HLA-A).

**Results**

Pegilodecakin treatment induced a systemic immune response biased towards Th1 cytokines and cytotoxic effector molecules (GranzymeB, FasL, lymphotoxinB) in the serum. PBMC analysis by Flow cytometry in pre and post treatment samples show invigoration of exhausted, T cells, with increased proliferation of LAG3+PD1+ CD8+ T cells throughout pegilodecakin treatment. The magnitude of PD-1+Lag-3+Ki-67+CD8+ T cells correlated with the objective response to pegilodecakin. T-cell clonal analysis (TCR sequencing of PBMCs) on pegilodecakin showed >10fold expansion of several hundred, previously undetected T-cell clones per patient, correlating with objective tumor response. On-treatment biopsies showed that Pegilodecakin increased GzmB+, Phospho-Stat3+ and Lag-3+ CD8+ T cells and HLA-A expression in the tumor. Patient on pegilodecakin + anti-PD-1 had an increased overall response rate compared to historical control (Table 1, 2), including responses in NSCLC patients with low tumor mutational burden.

**Conclusions**

Pegilodecakin treatment induced the hallmarks of CD8+ T-cell immunity in cancer patients, including the systemic elevation of IFNg and GranzymeB levels, expansion and activation of CD8+ TILs, invigoration and expansion of PD-1+/Lag-3+ CD8+ T-cell sub-set and the de-novo expansion of T-cell clones. The elements of the pegilodecakin induced immune activation correlated with the achievement of objective response. Combination of pegilodecakin with anti-PD-1 may provide a treatment alternative for patients and indications with low tumor antigen and tumor mutational burden.

**Trial Registration**

NCT0200944

**Ethics Approval**

The study was approved by the IRBs of all contributing Institutions.

**Table 1 (abstract P297).**
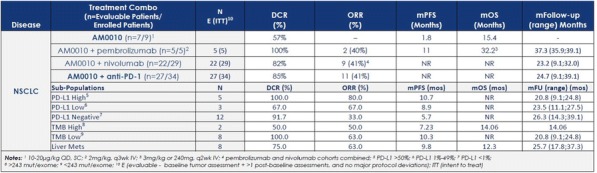
Pegilodecakin + anti-PD-1 in NSCLC

**Table 2 (abstract P297).**
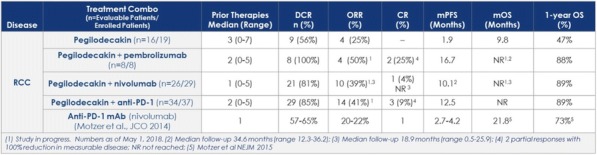
Pegilodecakin + anti-PD-1 in RCC

#### P298 T cell response induced by the addition of IL-7 treatment following sipuleucel-T immunotherapy in metastatic castration resistant-prostate cancer

##### Russell Pachynski, MD^2^, Lauren Harshman, MD^4^, Leonard Appleman, MD, PhD^5^, Paul Monk, MD^6^, Rhonda Bitting^7^, Omer Kucuk, MD^8^, Frederick Millard, MD^9^, John Seigna^10^, Steven Fling, PhD^11^, Nirasha Ramchurren, PhD^11^, Bruce Hess, BS^11^, Leonard D'Amico, PhD^11^, Andreanne Lacroix, BS^11^, Judith Kaiser, MBA, BSN, RN^11^, Michel Morre, DVM, MSc^12^, Anne GREGOIRE, phD^12^, Martin Cheever, MD^11^, Evan Yu, MD^13^

###### ^1^Washington University School of Medicine, St Louis, MO, USA; ^2^Washington University, St Louis, MO, USA; ^3^University of Chicago, Chicago, IL, USA; ^4^Dana Farber Cancer Institute, Boston, MA, USA; ^5^University of Pittsburg, Pittsburgh, PA, USA; ^6^Ohio State University, Columbus, OH, USA; ^7^Wake Forest Baptist Health, Winston-salem, NC, USA; ^8^Emory University, Atlanta, GA, USA; ^9^UCSD, San Diego, CA, USA; ^10^Dartmouth-Hitchcock, Lebanon, NH, USA; ^11^Fred Hutchinson Cancer Research Center, Seattle, WA, USA; ^12^Revimmune, Boulogne, MD, France; ^13^University of Washington, Seattle, WA, USA; ^14^UCSF, San Francisco, CA, USA

####### **Correspondence:** Russell Pachynski (lfong@medicine.ucsf.edu)

**Background**

Sipuleucel-T is a FDA-approved autologous cellular immunotherapy for asymptomatic or minimally symptomatic metastatic castration resistant prostate cancer (mCRPC). Treatment with sipuleucel-T results in humoral B cell responses, and can induce robust, antigen-specific T cell responses that correlate with overall survival. IL-7 is a homeostatic cytokine that can enhance the generation of naïve T cells and promote the generation of memory T cells. We hypothesized that treatment with IL-7 after sipuleucel-T would augment and prolong cellular immune responses compared to observation.

**Methods**

We performed a randomized phase 2 clinical trial where chemotherapy-naïve mCRPC patients receiving sipuleucel- T were either followed (observation arm) or treated (within 7 days) with recombinant human IL-7 10mcg/kg SC weekly x 4. Peripheral immune responses were evaluated at weeks 1 (pre-IL-7), 6, and 11 after completion of sipuleucel-T. T cell responses to PAP and PA2024 were assessed by ELISPOT, while ELISAs were used to assess antibody responses to these antigens. Flow cytometry was used to assess any treatment-induced modulation of peripheral T cells and other leukocyte subsets.

**Results**

A total of 54 patients were enrolled from 2015 to 2017 and randomized 1:1 into observation (n=26) or IL-7 (n=28) arms. Treatment with IL-7 was generally well tolerated, with injection site reactions being the most common toxicity. Clinical data will be further evaluated and presented later. For the IL-7, but not the observation arm, an expansion of all lymphocyte subsets was seen, with 2-3-fold increases over baseline in CD3, CD4, CD8 T cells, and CD56bright NK cells. Within the IL-7 arm, statistically significant increases over baseline were seen in T cells at weeks 6 and 11. However, compared to the observation arm, increases in absolute lymphocyte and T cell numbers for the IL-7 arm were statistically significant only at week 6. Patients in the IL-7 arm, but not observation arm, showed a significant reduction in neutrophil to lymphocyte ratio (NLR) over the course of treatment. However, there were no significant differences seen in frequencies of antigen-reactive (PAP or PA2024) T cells or antibody responses between observation and IL-7 arms.

**Conclusions**

Here, we present results from the first randomized trial comparing IL-7 vs observation following sipuleucel-T treatment in mCRPC patients. While IL-7 treatment did not increase the frequency of antigen specific T cells, it significantly expanded lymphocyte populations, suggesting IL-7 may result in an increase in absolute number of antigen-specific T cells in patients. Further analysis of T-cell clonal expansion is pending.

**Trial Registration**

NCT01881867

**Ethics Approval**

The study was IRB approved at each participating institution.

#### P299 Combination of subcutaneously administered TLR9 agonist lefitolimod with CTLA-4 checkpoint inhibitor ipilimumab - A phase I trial in patients with advanced solid tumors

##### Matthew Reilley, MD^1^, Casey Ager, BS^2^, Martin Meng^2^, Apostolia Tsimberidou, MD, PhD^2^, Sarina Piha-Paul, MD^2^, Timothy Yap, MD^2^, Siqing Fu, MD, PhD^2^, Aung Naing, MD, FACP^2^, Jordi Rodon^2^, Ly Nguyen^2^, Priya Bhosale, MD^2^, Manuel Schmidt, MSc^3^, Matthias Baumann^3^, Funda Meric-Bernstam, MD^2^, Michael Curran, PhD^2^, David Hong, MD^2^

###### ^1^University of Virginia, Charlottesville, VA, USA; ^2^MD Anderson Cancer Center, Houston, TX, USA; ^3^Mologen AG, Berlin, Germany

####### **Correspondence:** Michael Curran (mcurran@mdanderson.org)

**Background**

Toll-like receptors (TLR) have generated significant interest as an effective means of stimulating the immune system that results in the killing of tumor cells. TLR9 agonists can function as immunomodulators and are therefore useful targets for immunotherapy. Counter-regulatory signals exist that suppress the development of an anti-tumor response, such as IL-10, Treg cells, and PD-L1. Therefore, the efficacy of TLR9 against advanced malignancies may be enhanced by addition of an immune check-point blockade inhibitor to improve immune activation. Lefitolimod (MGN1703) is a covalently-closed dumbbell-shaped DNA molecule that functions as a TLR9 agonist. We developed a clinical trial combining lefitolimod with ipilimumab (anti-CTLA4) in patients with advanced malignancies.

**Methods**

This is a single-center investigator-initiated trial conducted at the University of Texas MD Anderson Cancer Center.

Eligible patients with advanced solid tumors without prior exposure to TLR agonists were enrolled in a dose escalation 3+3 phase 1 trial. Lefitolimod was administered subcutaneously weekly at 4 increasing dose levels and ipilimumab infused intravenously every 3 weeks at 3mg/kg. Peripheral blood and tumor biopsies were collected for analysis of changes in tumor immunity.

**Results**

To date 18 patients have enrolled and received at least one treatment. No dose-limiting toxicities (DLTs) have been encountered at any dose level. Thus, the maximum tolerated dose (MTD) of the combination was determined to be the highest dose level with lefitolimod at 120mg weekly and ipilimumab 3mg/kg every 3 weeks. The most common adverse events (AEs) include fatigue (72%), hepatic enzyme elevation (50%), anemia (44%), dyspnea (44%), myalgias (44%), abdominal pain (39%), anorexia (39%), cough (39%), rash (33%), fever (28%), hyperglycemia (28%), and nausea (28%). Grade 3 AEs occurring in more than one patient include dyspnea, hyperglycemia, fatigue, abdominal pain, and hypokalemia; only one episode of fatigue was felt to be possibly related to therapy. Two patients experienced a best response of stable disease for 45 weeks (primary peritoneal carcinoma) and 24 weeks (high-grade pancreatic neuroendocrine tumor) respectively at 30mg and 60mg dose levels of lefitolimod. Flow analysis of tumor cell immune infiltrates shows increased proportion and activation of cytotoxic T lymphocytes. PD- 1 expression increased after treatment suggesting potential benefit from additional PD-1 blockade.

**Conclusions**

The combination is safe and well tolerated in patients with advanced cancers. Expansion cohorts with intratumoral administration are ongoing.

**Trial Registration**

ClinicalTrials.gov Identifier: NCT02668770.

**Ethics Approval**

This study was approved by MD Anderson Cancer Center institution’s review board approval number 2015-0135.

**Consent**

Written informed consent was obtained from the patient for participation in the study. A copy of the written consent is available for review by the Editor of this journal.

#### P300 First-in-human phase 1 study of IT1208, a defucosylated humanized anti-CD4 depleting antibody, in patients with advanced solid tumors

##### Kohei Shitara, MD^1^, Satoshi ueha^2^, Shigeyuki Shichino^2^, Hiroyasu Aoki^3^, Haru Ogiwara, MD, PhD^2^, Tetsuya Nakatsura^4^, Toshihiro Suzuki^4^, Manami Shimomura^4^, Toshiaki Yoshikawa^4^, Kayoko Shoda^4^, Shigehisa Kitano^5^, Makiko Yamashita^5^, Takayuki Nakayama^5^, Akihiro Sato^6^, Sakiko Kuroda^6^, Masashi Wakabayashi^6^, Shogo Nomura^6^, Satoru Ito^7^, Kouji Matsushima^2^, Toshihiko Doi, MD, PhD^6^

###### ^1^National Cancer Hospital East, Kashiwa, Japan; ^2^Tokyo University of Science, Tokyo, Japan; ^3^The University of Tokyo, Tokyo, Japan; ^4^National Cancer Center, Kashiwa, Japan; ^5^National Cancer Center Hospital, New York, NY; ^6^National Cancer Center Hospital East, kashiwa, Japan; ^7^IDAC Therapeutics Inc, Tokyo, Japan

####### **Correspondence:** Toshihiko Doi (tdoi@east.ncc.go.jp)

**Background**

Transient CD4+ T cell depletion led to the proliferation of tumor-specific CD8+ T cells in the draining lymph node and increased infiltration of PD-1+CD8+ T cells into the tumor, which resulted in strong anti-tumor effects in tumor-bearing mice. [1] Here we report a first-in human study of IT1208, a defucosylated humanized anti-CD4 monoclonal antibody engineered to exert potent antibody-dependent cellular cytotoxicity.

**Methods**

Patients with advanced solid tumors for whom no standard therapy was available, were treated with intravenous infusion of IT1208 at dose of 0.1 or 1.0 mg/kg. First patient in each cohort was treated as single administration and other patients received two administrations of IT1208 on day 1 and 8 followed by safety and efficacy assessment. To assess cellular and molecular effects of IT1208 on the tumors, histological and transcriptomic analyses of pre- and post-treatment tumor samples were performed. The study was approved by ethics board in each institution.

**Results**

Eleven patients were enrolled in 0.1 mg/kg (n=4) and 1.0 mg/kg cohort (n=7), having gastric or gastro-esophageal (n=6), colorectal (n=3), esophageal (n=1) and pancreatic cancer (n=1, also having colon cancer). Grade 1 or 2 infusion-related reaction was observed in all patients but manageable. No other treatment related immune mediated adverse events or infections were observed. Decreased CD4+ cells in peripheral bloods by IT1208 were observed in all patients, especially in patients with two administrations of 1.0 mg/kg, reducing CD4+ cells count from median 395/μL at baseline to 3.5/μL at nadir. CD8+ cells increased on day 29 compared with baseline in most patients, which resulted in remarkably decreased CD4/8 ratios. One microsatellite stable colon cancer patient with lung and liver metastases achieved durable partial response, showing increased infiltration of Ki67+CD8+ cells into tumors after IT1208. Moreover, transcriptomic profiling of the liver metastatic site of the patient revealed upregulation of the expression of interferon-stimulated genes, T cell activation-related genes and antigen presentation-related genes after IT1208. Additional 3 patients with gastric or esophageal cancer achieved stable disease lasting at least 3 months.

**Conclusions**

Single agent IT1208 successfully depleted CD4+cells with manageable safety profile and encouraging preliminary efficacy signals, which warrants further investigations especially in combinations with immune check point inhibitors.

**Trial Registration**

UMIN000026564

**References**

1. Ueha S, Yokochi S, Ishiwata Y, et al. Robust Antitumor Effects of Combined Anti-CD4-Depleting Antibody and Anti-PD-1/PD-L1 Immune Checkpoint Antibody Treatment in Mice. Cancer Immunol Res. 2015;3:631-40.

#### P301 MLH1/ MSH2 expression and the prognosis of colorectal cancer in 978 Chinese patients

##### Yu Wang, MD, Shuiming Wang, Youping Deng

###### Oncology, nanjing, China

####### **Correspondence:** Yu Wang; Youping Deng (dengy@hawaii.edu)

**Background**

The purpose of this study was to evaluate the prognostic significance of MLH1/ MSH2 status in a large cohort of stage I to IV colorectal cancer (CRC) patients using the immunohistochemical analysis. The relationship among MLH1/ MSH2, clinical outcome and OS (Overall Survival) was also investigated.

**Methods**

The study included 978 patients with colorectal cancer (173 stage I, 395 stage II, 286 stage III and 124 stage IV) who underwent curative surgical resection. We used immunohistochemical to analyze MMR status through MLH1 and MSH2 expression. And we also used gene scanning to verify the microsatellite state of the patients.

**Results**

801 (81.99%) colorectal cancers showed positive MLH1/ MSH2 status while 177 (18.01%) colorectal cancers demonstrated negative MLH1/ MSH2 status using immunohistochemical analysis. Patients with positive MLH1/ MSH2 more frequently suffered from right-side colon, with 1-3 lymph nodes metastasis, with mucus, positive surgical margin (P<0.05), but with no evidence related with age, gender, tumor size, differentiation and TNM stage.Patients with negative MLH1/MSH2 showed significantly better OS than positive one(P<0.05).Univariate and multivariate analysis demonstrated that the independent predictive factors of colorectal cancer were TNM stage, lymph nodes metastasis, mucus, surgical margin and MLHl/MSH2 status(P<0.05). Gene scan denmostrated that 173(90.02%) were MSS, 12 (6.38 %) were MSI-H and 3(1.60%) were MSI-L. That is consistent with the immunohistochemical results.

**Conclusions**

This study confirmed the positive prognostic role of MLHl/MSH2.

#### P302 Phase II trial of reirradiation (ReRT) plus Pembrolizumab for locoregional inoperable recurrence or second primary squamous cell carcinoma of the head and neck (SCCHN): Analysis of early toxicity

##### Dan Zandberg, MD^1^, Tejan Diwanji, MD^2^, Robert Morales, MD^2^, Tiffani Tyer^2^, James Snider, MD^2^, Alexander Engelman, MD^2^, Soren Bentzen, PhD^2^, Thomas DeMora, MD^3^, Robert Malyapa, MD^2^, Navid Saeidi^2^, Ana Ponce Kiess, MD^4^, Josh Lubek, MD^2^, Robert Ord, MD^2^, Donita Dyalram, MD^2^, Kyle Hatten, MD^2^, Jeffrey Wolf, MD^2^, Rodney Taylor, MD^2^, John Papadimitriou, MD, PhD^2^, Ranee Mehra, MD^4^, Harry Quon, MD^4^, Hyunseok Kang, MD, MPH^4^, John Ridge, MD, PhD^3^, Jessica Bauman, MD^3^, William Regine, MD^2^, Mohan Suntharalingam, MD^2^, Scott Strome, MD^2^, Kevin Cullen, MD^2^

###### ^1^UPMC Hillman Cancer Center, Pittsburgh, PA, USA; ^2^University of Maryland, Baltimore, MD, USA; ^3^Fox Chase Cancer Center, Philadelphia, PA, USA; ^4^Johns Hopkins University, Baltimore, MD, USA

####### **Correspondence:** Dan Zandberg (zandbergdp@upmc.edu)

**Background**

Based on pre-clinical synergy between radiation and anti-PD-1 mAb, we initiated a phase II trial of ReRT plus pembrolizumab. The trial includes a stopping boundary for toxicity for the first 20 patients that completed 7 weeks of therapy. This abstract reports the early toxicity (up to 3 months post RT) for these patients. To our knowledge this is the first report of ReRT plus immunotherapy in SCCHN.

**Methods**

Main inclusion criteria are: unresectable (or declined resection) locoregional recurrence or second primary SCCHN (excluding salivary or cutaneous), prior RT completed >6 months ago and >50% of tumor volume previously radiated at doses > 45 Gy. ReRT is 1.2Gy BID for 5 weeks to total 60Gy (IMRT or Proton RT). Pembrolizumab is dosed 200mg IV q 3 weeks starting the first day of radiation. PET/CT is done 3 months after ReRT (after 6th cycle of pembrolizumab) to evaluate response with continued pembrolizumab until progression or 2 years. Primary endpoint is PFS (n=48) and the trial design used continuous monitoring of the incidence of G4/5 non-hematologic toxicity within the first 7 weeks of treatment using a Pocock-type stopping boundary in the first 20 patients.

**Results**

Twenty patients completed 7 weeks of therapy as of 7/18/18. Median age was 61 (50-79), primary sites were oral cavity (40%), oropharynx (40%), larynx (5%), and nasopharynx (15%), median time from prior RT was 72 months (10-300), 1 patient received proton RT, all others IMRT. All patients completed prescribed ReRT and the 2 cycles of pembrolizumab concurrent, and 79% completed all 6 cycles (1 patient is still on pembrolizumab within 3 months of RT). There were 423 AEs and 88 were treatment related (TRAEs: probable/definite from treatment either ReRT and/or pembrolizumab). Common TRAEs (any grade) experienced by patients’ included: mucositis (60%), radiation dermatitis (40%), fatigue (30%), dysphagia (25%), weight loss (15%), dysgeusia (15%). Hyperthyroidism (1 patient) and Hypothyroidism (2 patients) were the only immune related AEs. There were 4 G3 TRAEs which occurred in 2 patients (1 patient with dysphagia, radiation dermatitis, salivary duct inflammation) consistent with an incidence of 10% with an exact binomial upper 1-sided 95% CI of 28.3%. There were no treatment related G4/5 adverse events.

**Conclusions**

Evaluation of early toxicity shows that ReRT plus pembrolizumab was overall well tolerated. The pre-specified toxicity boundary was not crossed and accrual will continue towards the primary endpoint of PFS.

**Ethics Approval**

The study was approved by the IRB and University of Maryland, Baltimore. Approval Number HP-00061458

### Clinical Trials (In Progress)

#### P303 Correlation between prior surgery and immune related gastrointestinal toxicity among women receiving olaparib and tremelimumab for the treatment of recurrent ovarian cancer

##### Jaryse Harris, BA^1^, Carolyn Muller, MD^1^, Teresa Rutledge^1^, Olivier Rixe, MD, PhD^1^, Aisha Sethi^1^, Phyllis Gimotty, PhD^2^, Katherine Morris, MD, FACS^3^, Sarah Adams, MD^1,2^

###### ^1^University of New Mexico, Albuquerque, NM, USA; ^2^University of Pennsylvania, Philadelphia, PA, USA; ^3^University of Oklahoma, Oklahoma City, OK, USA; ^4^University of New Mexico Comprehensive C, Albuquerque, NM, USA

####### **Correspondence:** Sarah Adams (sarah.foster.adams@gmail.com)

**Background**

Ovarian tumors have been relatively resistant to immune checkpoint blockade, despite a strong rationale for immune therapy in this disease [1]. We recently demonstrated that targeted therapy with a poly(adenosine diphosphate- ribose) polymerase (PARP) inhibitor synergized with CTLA4 immune checkpoint blockade to achieve long-term survival in ovarian tumor models [2]. Based on these results, we launched INST 1419: A phase I/II study of the combination of olaparib and tremelimumab in BRCA1 and BRCA2 mutation carriers with recurrent ovarian cancer (NCT02571725). This ongoing trial has demonstrated clinical responses among patients treated to date. In keeping with prior studies of CTLA4 antibody therapy we have observed immune related adverse events [3]. Here we report an association between prior enteric surgery and the emergence of Grade 3 gastrointestinal toxicity.

**Methods**

Women with a confirmed germline mutation in BRCA1 or BRCA2 who have recurrent ovarian, tubal or peritoneal cancer with measurable disease are eligible for this study. Olaparib is administered orally at 300 mg twice daily, and tremelimumab intravenously at 10mg/kg every 28 days for four to six cycles followed by maintenance dosing every 12 weeks. The primary endpoint is overall response rate measured by modified WHO immune related response criteria [4]. Adverse events are scored using Common Terminology Criteria for Adverse Events. The target enrollment is 50 subjects. Response data is currently available for 13 patients.

**Results**

Of 13 patients who completed at least 3 cycles of treatment and were assessable for response, five (38%) experienced Grade 3 gastrointestinal toxicity. Four had colitis and one had gastritis requiring hospitalization for supportive care and steroids. Notably, four of these patients had a history of gastrointestinal surgery: three women with Grade 3 colitis had a history of colon resection and one patient with Grade 3 gastritis had a prior gastric sleeve surgery. None of the eight patients without Grade 3 gastrointestinal toxicity had a history of gastrointestinal surgery. The association between prior enteric surgery and Grade 3 gastrointestinal toxicity is significant (Fisher’s exact test, p=0.007).

**Conclusions**

Emerging data from our ongoing phase II trial demonstrates an association between prior enteric surgery and immune related gastrointestinal toxicity in response to combination therapy with a CTLA4 antibody and a PARP inhibitor. While these results will need to be validated in a larger patient cohort, they suggest that surgical history can be used to anticipate immune related toxicity in patients receiving immune checkpoint antibodies.

**Trial Registration**

This study is registered at clinicaltrials.gov NCT02571725.

**References**

1. Lavoue V, et al. Immunity of human epithelial ovarian carcinoma: the paradigm of immune suppression in cancer. J Transl Med, 2013;11:147.

2. Higuchi T, et al. CTLA-4 blockade synergizes therapeutically with PARP inhibition in BRCA1-Deficient ovarian cancer. Cancer Immunol Res, 2015; 3(11): 1257-68.

3. Hodi FS, Mihm MC, Soiffer RJ, Haluska FG, Butler M, Seiden MV, et al. Biologic activity of cytotoxic T lymphocyte-associated antigen 4 antibody blockade in previously vaccinated metastatic melanoma and ovarian carcinoma patients. Proceedings of the National Academy of Sciences of the USA of America 2003;100(8):4712-17.

4. Wolchok JD, Hoos A, O'Day S, et al. Guidelines for the evaluation of immune therapy activity in solid tumors: immune-related response criteria. Clin Cancer Res. 2009 Dec 1;15(23):7412-20.

**Ethics Approval**

This study was approved by the Western IRB in March 2016, study number 1159154, approval number 20152180.

#### P304 A phase 1 study of MGD007, a humanized gpA33 x CD3 DART® protein, in combination with MGA012, an anti-PD-1 antibody, in patients with relapsed/refractory metastatic colorectal cancer

##### Richard Kim, MD^1^, David Ryan, MD^2^, Stacey Stein, MD^3^, James Cleary, MD, PhD^4^, Liqin Liu, PhD^5^, Ralph Alderson, PhD^5^, Francine Chen, MD^5^, Peter Lung, BS, HT (ASCP)^5^, Allan Reduta, BA^5^, Syd Johnson, PhD^5^, Jan Baughman, MPH^5^, Ezio Bonvini^5^, Paul Moore, PhD^5^, Joanna Lohr, PhD^5^, Jon Wigginton, MD^5^, Jan Davidson- Moncada, MD, PhD^5^, John Powderly, MD, CPI^6^

###### ^1^Moffitt Cancer Center, Tampa, FL, USA; ^2^Massachusetts General Hospital, Boston, MA, USA; ^3^Yale Cancer Center, New Haven, CT, USA; ^4^Dana Farber Cancer Institute, Boston, MA, USA; ^5^MacroGenics, Inc., Rockville, MD, USA; ^6^Carolina BioOncology, Huntersville, NC, USA

####### **Correspondence:** Richard Kim; Jan Davidson- Moncada (davidsonj@macrogenics.com)

**Background**

The benefit of checkpoint blockade for colorectal cancer (CRC) is restricted to mismatch repair deficient patients, indicating the need for other means to further leverage T-cell mediated antitumor responses in this population. MGD007 is a gpA33 x CD3 bispecific DART protein designed to recruit/expand host T cells, via their CD3 component, and to mediate tumor cell killing through engagement of glycoprotein A33 (gpA33), a cell surface target on >95% of CRC tumors. MGA012, also known as INCMGA00012, is an anti-PD-1 antibody undergoing Phase 1 investigation. MGD007 demonstrated drug targeting to both CD3 T cells and gpA33 in a Phase 1 monotherapy trial. Concomitant with its ability to mediate T-cell killing of CRC tumor cells, preclinical data show that MGD007 induces expression of PD-1/PD-L1 on T cells and CRC tumor cell lines, respectively. Furthermore, MGA012 enhances MGD007-mediated CTL activity and interferon gamma release upon exposure to gpA33-positive CRC and T cells, while MGD007 anti-tumor activity in preclinical mouse models is enhanced upon combination with anti-PD-1. Based on the two molecules’ intended complementary mechanisms of action, the current study will investigate whether the novel, mechanism-based combination of MGD007 (T-cell agonist) and MGA012 (checkpoint inhibitor) could mediate enhanced antitumor activity compared to either modality alone.

**Methods**

This Phase 1b/2 study is designed to characterize safety, tolerability, dose-limiting toxicities (DLTs), and maximum tolerated dose (MTD)/maximum administered dose (MAD) of MGD007+MGA012 in patients with relapsed/refractory metastatic CRC. Patients are eligible irrespective of KRAS/MMR status, after treatment with 2-5 prior standard therapy regimens in the metastatic setting, or if they did not tolerate fluoropyrimidines, oxaliplatin or irinotecan chemotherapy or are not good candidates for standard of care. Secondary objectives include characterization of pharmacokinetics, pharmacodynamics, and immunogenicity of the combination, and investigation of the preliminary antitumor activity as measured by objective response rate, disease control rate, and progression-free survival rate at 16 weeks using both Response Evaluation Criteria in Solid Tumors (RECIST 1.1), and immune-related RECIST. Translational studies will investigate the immune modulatory activity of MGD007+MGA012. The study will include a 3+3+3 design Dose Escalation Phase to determine the MTD or MAD of the combination, followed by a Cohort Expansion Phase treating patients at the MTD or MAD to further define the safety and initial antitumor activity of the combination. Patients may be treated for up to 12 cycles in the absence of disease progression, DLT, or other criteria for permanent discontinuation. Enrollment is ongoing.

**Trial Registration**

NCT03531632

**Ethics Approval**

The study was approved by each institution's Insitutional Review Board.

#### P305 A phase 1, open label, dose escalation study of MGD009, a B7-H3 x CD3 DART® protein, in combination with MGA012, an anti-PD-1 antibody, in patients with relapsed or refractory B7-H3-expressing tumors

##### Alexander Spira, MD, PhD, FACP^1^, Stacie Goldberg, MD^2^, James Strauss, MD^3^, Johanna Bendell, MD^4^, Gregory Cote^5^, E Rahma, MD^6^, Marwan Fakih, MD^7^, Ralph Alderson, PhD^2^, Liqin Liu, PhD^2^, Ross La Motte-Mohs, PhD, BS^2^, Amy Worth^2^, Ashley Lowe^2^, Jan Baughman, MPH^2^, Tony Wu, PhD^2^, Syd Johnson, PhD^2^, Ezio Bonvini^2^, Paul Moore, PhD^2^, Jon Wigginton, MD^2^

###### ^1^Virginia Cancer Specialists, Fairfax, VA, USA; ^2^MacroGenics, Inc., Rockville, MD, USA; ^3^Mary Crowley Cancer Research, Dallas, TX, USA; ^4^Sarah Cannon Research Institute, Nashville, TN, USA; ^5^Massachusetts General Hospital, Boston, MA, USA; ^6^Dana Farber Cancer Institute, Boston, MA, USA; ^7^City of Hope, Duarte, CA, USA

####### **Correspondence:** Alexander Spira; Stacie Goldberg (goldbergs@macrogenics.com)

**Background**

T cells naturally undergo activation-induced upregulation of co-inhibitory pathways, which may limit the antitumor immune response. Blocking these inhibitory pathways may enhance the antitumor activity of CD3 bispecifics. MGD009 is a clinical stage B7-H3 x CD3 DART protein designed to redirect T cells to kill B7-H3-expressing tumor cells. B7-H3, a member of the B7 family of immune regulators, is overexpressed in a variety of solid tumors and has limited expression in normal tissues. In preclinical studies, MGD009 causes T-cell infiltration, activation and expansion in the tumors. MGD009 upregulates PD-1 on T cells and PD-L1 on tumor cells and immune cells in vitro. Preliminary observations in patients enrolled in the ongoing Phase 1 dose escalation trial with MGD009 alone indicate evidence of PD-1 up-regulation on both peripheral CD4 and CD8 T cells. MGA012, also known as INCMGA00012, is an anti-PD-1 antibody that has shown clinical activity in an ongoing Phase 1 trial. In vitro and in vivo studies have shown enhanced antitumor activity with the combination of MGD009 and MGA012 beyond that achieved with MGD009 alone. It is hypothesized that T-cell checkpoint inhibition with MGA012, combined with activation and enhancement of redirected T-cell killing with MGD009, could mediate greater anti-tumor activity than either agent alone in patients with B7-H3 expressing solid tumors.

**Methods**

This is a Phase 1, open-label, dose escalation, and cohort expansion study (NCT03406949) designed to characterize the safety, tolerability, pharmacokinetics, pharmacodynamics, immunogenicity, and preliminary antitumor activity of MGD009 in combination with MGA012. Dose escalation uses a 3+3+3 design, with patients treated every 2 weeks with escalating doses of IV MGD009 (starting dose 3 μg/kg), and MGA012 at a dose of 3 mg/kg in all cohorts. Antitumor activity is assessed by both conventional Response Evaluation Criteria in Solid Tumors (RECIST) 1.1 and immune-related RECIST. The study consists of a Dose Escalation Phase to determine the maximum tolerated dose (MTD) or maximum administered dose (MAD -- if no MTD is defined) of the combination, followed by a Cohort Expansion Phase to further define the safety and initial antitumor activity of the combination with the doses established in the Dose Escalation Phase. Patients with B7-H3-expressing, unresectable, locally advanced or metastatic solid tumors of any histology will be enrolled in the Dose Escalation Phase. Expansion cohorts will be limited to 6 tumor types (N=20/cohort) treated at the MTD/MAD of the combination. The study is ongoing at approximately 6 U.S. centers.

**Trial Registration**

NCT03406949

**Ethics Approval**

This study was approved by each participating institution's Institutional Review Board.

#### P306 A phase 1/2, first-in-human, dose escalation study of MGC018 (anti-B7-H3 antibody-drug conjugate) alone and in combination with MGA012 (anti-PD-1 antibody) in patients with advanced solid tumors

##### John Powderly, MD, CPI^1^, Deryk Loo^2^, Anthony Joshua, MD^3^, Johanna Bendell, MD^4^, Alexander Spira, MD, PhD, FACP^5^, Joanna Lohr, PhD^2^, Pepi Pencheva, MD^2^, Jichao Sun, PhD^2^, Jan Baughman, MPH^2^, Ezio Bonvini^2^, Jennifer Brown^5^, Nehal Lakhani, MD, PhD^6^, Jon Wigginton, MD^2^

###### ^1^Carolina BioOncology, Huntersville, NC, USA; ^2^MacroGenics, Inc., San Francisco, CA, USA; ^3^St. Vincent's Hospital, Syndey, Australia; ^4^Sarah Cannon Research Institute, Nashville, TN, USA; ^5^Virginia Cancer Specialists, Fairfax, VA, USA; ^6^START Midwest, Grand Rapids, MI, USA

####### **Correspondence:** John Powderly; Jon Wigginton (wiggintonj@macrogenics.com)

**Background**

Antibody-drug conjugates (ADCs) are a powerful class of agents for targeted cancer treatment, combining the specificity of a monoclonal antibody with highly-potent cytotoxic “payloads” for selective delivery of cytotoxic agents to cancer cells, offering potential for increased efficacy while minimizing exposure to normal tissues. Immune-checkpoint blockade is a promising approach, and inhibitors of programmed death-1 (PD-1), PD-1 ligand, and cytotoxic T-lymphocyte–associated protein 4 (CTLA-4) are approved for patients with various solid tumors; however, a significant proportion do not derive clinical benefit. B7-H3, a member of the B7 family of immune regulators, is overexpressed in a wide range of solid tumors, with limited normal tissue expression. MGC018 is an ADC targeted against B7-H3 with a linker-duocarmycin payload, conjugated to an anti-B7-H3 monoclonal antibody. MGC018 demonstrated favorable binding properties; potent cytotoxic activity toward multiple B7-H3-expressing human tumor cells in vitro; antitumor activity in B7-H3 human tumor xenografts (breast cancer, ovarian cancer, lung cancer and melanoma); a favorable tissue cross-reactivity profile across 34 normal human tissues; and acceptable safety following repeat-dose administration in cynomolgus monkeys. It is hypothesized that MGC018 monotherapy will mediate antitumor activity against B7-H3-positive tumors. Further, that administration of this B7- H3-directed cytotoxic agent could enhance tumor cell death and drive “auto-vaccination” of the host immune system, with added engagement of a T-cell response by sequenced administration of MGC018 in combination with anti-PD-1 antibody (MGA012; also known as INCMGA00012).

**Methods**

This Phase 1/2, bifurcated-design study will characterize safety, dose-limiting toxicities (DLTs), and maximum tolerated/administered dose (MTD/MAD) for MGC018 as monotherapy (Module A) or in combination with MGA012 (Module B) in patients with advanced solid tumors. Pharmacokinetics, immunogenicity, and impact of treatment on various measures of immune function and tumor cell death will be assessed. Tumor assessments will occur every 42-84 days, and response status will be defined using RECIST v1.1 and irRECIST. Module B will commence after the MTD/MAD of MGC018 monotherapy (Module A) is defined. Each module consists of a Dose Escalation (3+3+3 design) followed by a Cohort Expansion Phase. Patients with solid tumors of any histology will be enrolled in the Dose Escalation Phases; Cohort Expansion will include patients with SCCHN, prostate carcinoma, triple negative breast cancer, and uveal melanoma. Patients who do not experience DLT/unacceptable toxicity or meet criteria for permanent discontinuation may undergo additional cycles. Patients will be followed for survival every 3 months for 2 years following last dose.

**Trial Registration**

Pending

**Ethics Approval**

Each institution will obtain Institutional Review Board approval prior to enrollment of subjects.

#### P307 An open-label, phase 1B study of NEO-PV-01 + CD40 agonist antibody (APX-005M) or Ipilimumab with Nivolumab in patients with advanced or metastatic melanoma

##### Omid Hamid, MD^1^, David Spigel, MD^2^, Patrick Ott, MD, PhD^3^, Siwen Hu-Lieskovan, MD, PhD^4^, Karl Lewis, MD^5^, Michael Gordon, MD^6^, Lisa Cleary^7^, Melissa Moles^7^, Richard Gaynor, MD^7^, Matthew Goldstein, MD, PhD^7^, Les Brail^7^, Keith Flaherty, MD^8^

###### ^1^Angeles Clinic, Los Angeles, CA, USA; ^2^Sarah Cannon, Nashville, TN, USA; ^3^Dana-Farber Cancer Institute, Boston, MA, USA; ^4^UCLA, Los Angeles, CA, USA; ^5^University of Colorado Denver, Aurora, CO, USA; ^6^Honor Health, Scottsdale, AZ, USA; ^7^Neon Therapeutics, Cambridge, MA, USA; ^8^Massachusetts General Hospital, Boston, MA, USA

####### **Correspondence:** Omid Hamid; Les Brail (lbrail@neontherapeutics.com)

**Background**

Cancer cells contain unique DNA mutations that result in altered amino acid sequences known as neoantigens.

Growing evidence supports a central role for neoantigens as targets for tumor directed immune responses. Tumor mutational burden and neoantigen load have been associated with anti-tumor activity of checkpoint inhibitors. NEO- PV-01 is a personal neo-antigen vaccine designed specifically for the molecular profile of each individual’s tumor. In the ongoing NT-001 study, NEO-PV-01 administered in combination with nivolumab has been shown to be well tolerated and to generate neoantigen specific immune responses in patients with melanoma. Here we describe a clinical trial, NT-003, combining NEO-PV-01 in combination with either nivolumab, nivolumab and ipilimumab, or the CD40 agonist antibody APX-005M and nivolumab. Immune modulatory antibodies that enhance T cell priming offer a rational combination partner with vaccines to further enhance induction of de novo T cell reactivity and to expand existing T cell responses against neoantigens.

**Methods**

NT-003 is a multi-arm, phase 1B study designed to evaluate the safety of administering NEO-PV-01 using an alternative vaccination schedule, or combination regimens with APX005M or ipilimumab with nivolumab in patients with advanced or metastatic melanoma. Please see Figure 1 for design. Patients undergo a baseline tumor biopsy and HLA typing. DNA and RNA sequencing is performed on tumor as well as peripheral blood. NEO-PV-01 is custom designed for each individual patient and contains up to 20 peptides approximately 14-35 amino acids in length. The peptides are pooled into four groups and mixed with poly-ICLC at the time of administration. On Day 1, patients will begin treatment with nivolumab (Q2W). Beginning at Week 12, patients begin immunizations with NEO-PV-01. Also at beginning at week 12, patients will begin to receive the co-administered therapy/(s) depending upon their cohort and arm assignment (Figure 1). The primary endpoint is safety. Secondary endpoints are ORR, CBR, PFS, and assessment of response conversion beginning on Week 12. Exploratory objectives include extensive immune monitoring with antigen-specific analyses over multiple timepoints of both peripheral blood and tumor ( NCT03597282).

**Trial Registration**

NCT03597282

**Fig. 1 (abstract P307).**
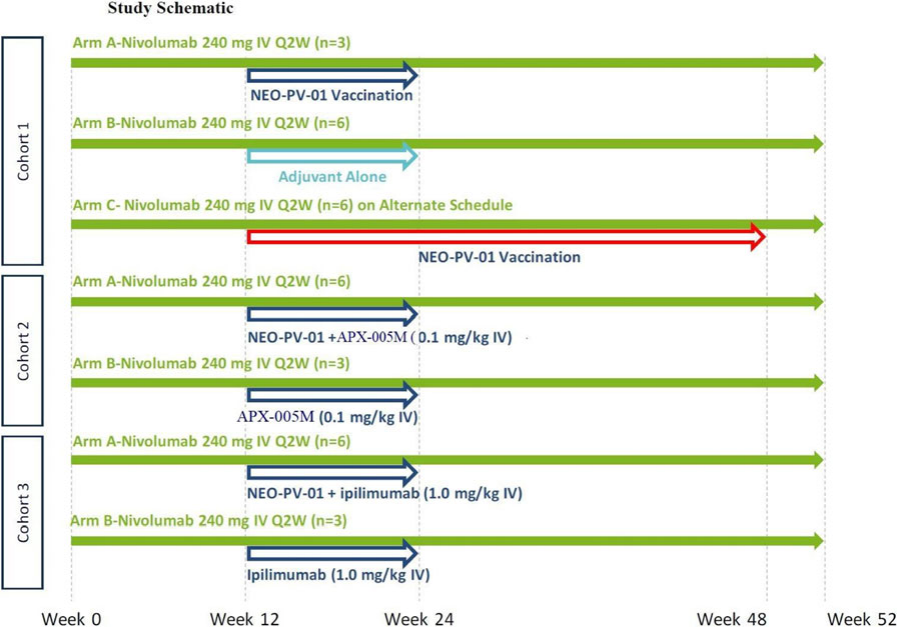
See text for description.

#### P308 A Phase 1 study of MEDI5752, a bispecific antibody that preferentially targets PD-1 and CTLA-4 expressing T cells, in patients with advanced solid tumors

##### Jeffery Brubaker^1^, Ben Tran^2^, Mark Voskoboynik^3^, James Kuo^4^, Yung-Lue Bang, MD^5^, Hyun-Cheo Chung, MD, PhD^6^, Myung-Ju Ahn^7^, Sang-We Kim^8^, Ayesh Perera^1^, Daniel Freeman^1^, Ikbel Achour, PhD^1^, Raffaella Faggioni, PhD^1^, Feng Xiao^1^, Charles Ferte, MD, PhD^1^, Charlotte Lemech, MBBS BSc(med) MD(res)^4^

###### ^1^MedImmune, Gaithersburg, MD, USA; ^2^Peter MacCallum Cancer Center, Melbourne, Australia; ^3^Nucleus Network, Melbourne, Australia; ^4^Scientia Clinical Research, Sydney, Australia; ^5^Seoul National University Hospital, Seoul, Korea, Republic of; ^6^Yonsei Cancer Center, Yonsei University, Seoul, Korea, Republic of; ^7^Samsung Medical Center, Seoul, Korea, Republic of; ^8^Asan Medical Center, Songpa-Gu, Korea, Republic of

####### **Correspondence:** Charles Ferte (fertec@MedImmune.com)

**Background**

Based on demonstrated clinical activity and manageable safety profiles, checkpoint inhibiting antibodies blocking PD 1, PD-L1, or CTLA-4 have received regulatory approvals for the treatment of various malignancies [1-5]. The combination therapy with anti-PD-1 and anti-CTLA-4 agents is approved by FDA for metastatic melanoma, renal cell carcinoma and microsatellite instability-high (MSI-H) or mismatch repair deficient (dMMR) metastatic colorectal cancer, based on improved overall survival versus either agent alone [6-10]. Numerous clinical studies of combination immunotherapy are currently investigating the same combination across a range of solid tumors [11- 15]. Although the efficacy of these drug combinations is dose dependent, the toxicity associated with anti-CTLA-4 agents, in particular, is dose limiting, thereby potentially affecting treatment outcomes with combination therapy.MEDI5752 is a bispecific humanized IgG1 monoclonal antibody that binds PD-1 and CTLA-4. In contrast to the combination therapy, MEDI5752 exhibits a novel T cell targeting mechanism that could provide a favorable toxicity profile. In addition, we have shown that MEDI5752 can impact cell surface expression of PD-1. Based on these novel mechanisms of action, MEDI5752 may show improved efficacy and safety in comparison to co- administration of conventional anti-PD-1/anti-PD-L1 and anti-CTLA-4 antibodies.

**Methods**

This is a Phase 1, first-time-in-human, multicenter, open-label study in patients with advanced solid tumors. The dose-escalation phase will evaluate approximately six MEDI5752 dose levels to identify a maximum tolerated dose. Dose escalation will be followed by two dose-expansion cohorts in defined setting with patients with advanced or metastatic solid tumor and tested against a control arm. Subjects will remain on treatment until confirmed progressive disease, initiation of alternative cancer therapy, unacceptable toxicity, or other reason for discontinuation. The primary endpoints are safety and efficacy (objective response in the dose-expansion phase). Secondary endpoints include additional efficacy assessment across both phases, pharmacokinetics, and immunogenicity.

**Trial Registration**

NCT03530397

**References**

1. Opdivo Prescribing Information. Princeton, NJ: Bristol-Myers Squibb Company, 2018. [revised 2018 Apr].

2. Keytruda Prescribing Information. Whitehouse Station, NJ: Merck Sharp & Dohme Corp, 2018 [revised 2018 Jun].

3. Tecentriq Prescribing Information. San Francisco (CA): Genentech, Inc, 2018. [revised 2019 Jul].

4. Imfinzi Prescribing Information. Wilmington (DE): AstraZeneca Pharmaceuticals LP, 2018.[revised 2018 Feb].

5. Yervoy Prescribing Information. Princeton (NJ): Bristol-Myers Squibb Company, 2018. [revised 2018 Apr].

6. Larkin J, Chiarion-Sileni V, Gonzalez R, Grob JJ, Cowey CL, Lao CD, et al. Combined nivolumab and ipilimumab or monotherapy in untreated melanoma. N Engl J Med. 2015;373:23-34.

7. Postow MA, Chesney J, Pavlick AC, Robert C, Grossmann K, McDermott D, et al. Nivolumab and ipilimumab versus ipilimumab in untreated melanoma. N Engl J Med. 2015;372:2006-2017.

8. Motzer RJ, Tannir NM, McDermott DF, Frontera OA, Melichar B, Choueiri TK, et al. Nivolumab plus ipilimumab versus sunitinib in advanced renal-cell carcinoma. N Engl J Med. 2018;378:1277-1290.

9. Overman MJ, Lonardi S, Wong KYM, Lenz H-J, Gelsomino F, Aglietta M, et al. Durable clinical benefit with nivolumab plus ipilimumab in DNA mismatch repair–deficient/microsatellite instability–high metastatic colorectal cancer. J Clin Oncol. 2018;36:773-779.

10. Broderick JM. Nivolumab, ipilimumab combo approved by FDA for MSI-H/dMMR colorectal cancer. Pharmacy Times, 11 July, 2018. Available at: https://www.pharmacytimes.com/news/nivolumab-ipilimumab-combo-approved-by-fda-for-msihdmmr-colorectal-cancer. Accessed 18 July, 2018.

11. Antonia S, Goldberg SB, Balmanoukian A, Chaft JE, Sanborn RE, Gupta A, et al. Safety and antitumour activity of durvalumab plus tremelimumab in non-small cell lung cancer: a multicentre, phase 1b study. Lancet Oncol. 2016a;17(3):299-308.

12. Haddad R, Gillison M, Ferris RL, Harrington K, Monga M, Baudelet C, et al. Double-blind, two-arm, phase 2 study of nivolumab (nivo) in combination with ipilimumab (ipi) versus nivo and ipi-placebo (PBO) as first-line (1L) therapy in patients (patients) with recurrent or metastatic squamous cell carcinoma of the head and neck (R/M SCCHN)—CheckMate 714. Ann Oncol. 2016;27(suppl_6):1017TiP-TiP.

13. Kelley RK, Abou-Alfa GK, Bendell JC, Kim T-Y, Borad MJ, Yong W-P, et al. Phase I/II study of durvalumab and tremelimumab in patients with unresectable hepatocellular carcinoma (HCC): Phase I safety and efficacy analyses. J Clin Oncol. 2017;35(15_suppl):4073.

14. Janjigian YY, Ott PA, Calvo E, Kim JW, Ascierto PA, Sharma P, et al. Nivolumab ± ipilimumab in patients with advanced (adv)/metastatic chemotherapy-refractory (CTx-R) gastric (G), esophageal (E), or gastroesophageal junction (GEJ) cancer: CheckMate 032 study. J Clin Oncol. 2017;35(15_suppl):4014.

15, Escudier B, Tannir NM, McDermott DF, Frontera OA, Melichar B, Plimack ER, et al. LBA5CheckMate 214: Efficacy and safety of nivolumab + ipilimumab (N+I) v sunitinib (S) for treatment-naïve advanced or metastatic renal cell carcinoma (mRCC), including IMDC risk and PD-L1 expression subgroups. Ann Oncol. 2017;28(suppl_5):mdx440.029- mdx440.029.

#### P309 Phase I dose-finding study of MIW815 (ADU-S100), an intratumoral STING agonist, in patients with advanced solid tumors or lymphomas

##### Janis Callister^1^, Funda Meric-Bernstam, MD^2^, Theresa Werner^3^, Stephen Hodi^4^, Wells Messersmith, MD^5^, Nancy Lewis^6^, Craig Talluto^7^, Mirek Dostalek^6^, Aiyang Tao^6^, Sarah McWhirter^8^, Damian Trujillo^8^, Jason Luke, MD, FACP^9^

###### ^1^Articulate Science, Manchester, UK; ^2^MD Anderson Cancer Center, Houston, TX, USA; ^3^University of Utah, Salt Lake City, UT, USA; ^4^Dana-Faber Cancer Institute, Boston, MA, USA; ^5^University of Colorado Cancer Center, Aurora, CO, USA; ^6^Novartis Pharmaceuticals Corporation, East Hanover, NJ, USA; ^7^Novartis Institutes for BioMedical Resea, Cambridge, MA, USA; ^8^Aduro Biotech Inc, Berkeley, CA, USA; ^9^The University of Chicago Medicine, Chicago, IL, USA

####### **Correspondence:** Funda Meric-Bernstam (fmeric@mdanderson.org)

**Background**

MIW815 (ADU-S100) is a novel synthetic cyclic dinucleotide that can activate human STING (STimulator of INterferon Genes) in antigen-presenting cells. In preclinical models, STING pathway activation can induce tumor antigen-specific T-cell priming within the tumor microenvironment, leading to antitumor immunity and tumor destruction.

**Methods**

Eligible patients (≥2 accessible tumors; Eastern Cooperative Oncology Group Performance Status ≤1) include those with advanced/metastatic solid tumors or lymphomas with progressive disease despite standard of care or for whom there is no standard treatment. MIW815 (ADU-S100) is administered by weekly intratumoral injections (3 weeks on/1 week off) at escalating doses (starting dose: 50 μg) in 28-day cycles. Primary objectives are to characterize safety and tolerability and to identify a recommended dose for future studies. Secondary objectives include preliminary efficacy, pharmacokinetics (PK), and pharmacodynamics (PD). The study is currently in dose escalation.

**Results**

As of June 15, 2018, 41 heavily pretreated patients (median age 62 years; range 26–80 years) with various solid tumors or lymphomas were enrolled. Thirty-five patients have discontinued from the study for the following reasons: disease progression (n=26), physician/patient decision (n=8), and death (n=1); 6 patients continue to receive treatment. No dose-limiting toxicities (DLTs) were reported during the first cycle at any dose level. The most common (≥10% of patients) treatment-related AEs (TRAEs) were pyrexia (n=7; 17.1%), injection site pain (n=6; 14.6%), and headache (n=6; 14.6%). Grade 3/4 TRAEs included increased lipase (n=2; 4.9%) and elevated amylase, tumor pain, dyspnea, respiratory failure, and injection site reaction (n=1 each; 2.4%). Systemic MIW815 (ADU- S100) exposure increased with dose. On-treatment tumor biopsies showed increases in CD8 T cells infiltrating the injected tumors in a subset of patients. Preliminary antitumor activity, PK analysis, and PD data from injected lesions, non-injection lesions, and peripheral blood, will be presented.

**Conclusions**

Intratumoral injection of MIW815 (ADU-S100) was well tolerated in doses tested thus far in patients with advanced solid tumors and lymphoma, with no DLTs reported to date. Trials evaluating combinations of MIW815 (ADU-S100) with anti-PD1 or anti-CTLA4 antibodies are ongoing.

**Ethics Approval**

This study was approved by an independent ethics committee or institutional review board at each site.

#### P310 NANT Cancer Vaccine an orchestration of immunogenic cell death by overcoming immune suppression and activating NK and T cell therapy in patients with third line or greater TNBC and head & neck SCC

##### Eric Carlson^1^, Mira Kistler, MD^2^, Patrick Soon-Shiong, MD, FRCS, FACS^1^, John Lee, MD^1^, Chaitali Nangia, MD^2^, Leonard Sender^2^, Frank Jones, PhD^3^

###### ^1^Nantkwest, Los Angeles, CA, USA; ^2^Chan Soon-Shiong Institute for Medicine, El Segundo, CA, USA; ^3^NantCell, CULVER CITY, CA, USA

####### **Correspondence:** John Lee (John.Lee@NantKwest.com)

**Background**

Triple negative breast cancer (TNBC) and head & neck squamous cell cancer (HNSCC) have multiple mechanisms to prevent immune recognition and immune eradication that lead to the creation of an immune suppressive tumor microenvironment. We hypothesize that effective and sustained response against tumors requires a coordinated approach that: 1. reverses the immune-suppressive tumor microenvironment, 2. induces immunogenic tumor cell death and 3. reengages NK and T-cell tumor response against a 4. cascade of tumor antigens. To test this hypothesis, we have developed the NANT Cancer Vaccine: a temporospatial approach that combines: metronomic low dose chemotherapy, SBRT, off-the-shelf cryopreserved allogeneic NK cells, yeast and adenoviral tumor associated antigen vaccines, IL-15RαFc superagonist N-803 immunostimulatory cytokine, with checkpoint inhibitor.

**Methods**

A phase 1b, single-arm, open-label trial of the NANT Cancer Vaccine in patients with recurrent metastatic third line or greater TNBC and HNSCC was initiated. Treatment occurred in 3-week cycles of low-dose chemotherapy (aldoxorubicin, cyclophosphamide, cisplatin, nab-paclitaxel, 5-FU/L), antiangiogenic therapy (bevacizumab), SBRT, engineered allogeneic high affinity CD-16 NK-92 cells (haNK), IL-15RαFc (N-803), adenoviral vector- based CEA, MUC1, Brachyury, HER2 vaccine (Ad), yeast vector-based RAS, Brachyury and CEA vaccine (Ye), and an IgG1 PD-L1 inhibitor, avelumab plus cetuximab. The primary endpoint is incidence of treatment-related adverse events. Secondary endpoints include ORR, DCR, PFS, and OS.

**Results**

To date, 3 patients with 3rd-line or greater TNBC and 2 patients with 4th-line or greater HNSCC have been treated with the NANT Cancer Vaccine. All treatment was completed in the outpatient setting, with no immune-related adverse events. Two of the three patients with TNBC experienced partial response (78% and 62% decrease by irRC). Both patients with HNSCC experienced objective tumor response (100% and 47% decrease by irRC). Four hematologic DLT’s were observed and managed with a planned dose reduction of cisplatin. All responding patients are still undergoing therapy.

**Conclusions**

This preliminary data suggests that the NANT Cancer Vaccine of low-dose chemoradiation combined with innate and adaptive immunotherapy can be administered safely in an outpatient setting without any observed increased irAE’s. Preliminary efficacy result of four out of five patients (80%) with confirmed overall responses, with one of these four patients demonstrating a complete response with 5th-line metastatic disease is encouraging.

**Trial Registration**

NCT03387111 NCT03387085

**Ethics Approval**

These studies were approved by IRB Company, approval numbers 2018-0001-CSSIFM and 18-0002-CSSIFM.

#### P311 Positive identification of neoepitope specific T cell by tumor-normal DNA & RNA sequencing from breast cancer patient leading to yeast-based vaccine phase 1 trial delivering tumor specific neoepitopes

##### Peter Sieling, PhD^1^, Steve Benz^2^, Zach Sanborn, PhD^1^, Kayvan Niazi^1^, Tom King^3^, Shahrooz Rabizadeh, PhD^1^, Andrew Nguyen^2^

###### ^1^NantBio, Culver City, CA, USA; ^2^Nantomics, Rockville, MD, USA; ^3^NantCell, Culver City, CA, USA

####### **Correspondence:** Shahrooz Rabizadeh (shahrooz@nantworks.com)

**Background**

Patient specific neoepitope discovery and subsequent vaccine development promise an opportunity for immunological memory, tumor clearance, and durable remission. Screening for neoepitope specific T cells in breast cancer patients utilizing our proprietary tumor-normal DNA & RNA sequencing technology led to successful identification of peptide-specific T cell clones isolated from biopsied tissue. All T cell clones specifically recognized the same tumor-specific mutations and not their wild-type counterpart, and demonstrated CDR3 T cell receptor diversity. The polyclonal nature of isolated T cells and the specificity with which they engaged mutant protein demonstrated the validity of this bioinformatic approach to neoepitope identification and formed the basis for this clinical trial. Based on this platform, we have initiated a clinical trial of neoepitope vaccine delivery using a yeast vehicle.

**Methods**

This study requires tumor-normal DNA and RNA sequencing to identify tumor specific DNA mutations expressed by tumor RNA and presented by patient MHC I and II (also identified by DNA sequencing). Tumor specific neoepitopes are expressed as a polytope in S. cerevisiae and delivered subcutaneously to patients enrolled in this trial. This phase 1 study aims to determine primarily, safety, dosing, including recommended phase 2 dose (RP2D), and secondarily, efficacy (disease recurrence rate, disease-free survival and overall survival) of a personalized neoepitope yeast-based vaccine in subjects who have completed potentially curative therapy for their solid cancer (eg, colorectal cancer, breast cancer, head and neck squamous cell carcinoma, melanoma) (NCT03552718).

**Results**

Our data demonstrate that our proprietary algorithms accurately predict tumor specific neoepitopes, which appropriately engender CD4+ and CD8+ T cell responses. These responses enable tumor clearance in combination with immunostimulants such as IL-15RαFc (N-803) and IL-12. Our previous ex vivo studies of patient-derived TILs further provide evidence of neoepitope engagement, establishing clonal T cell populations reactive against predicted neoepitopes in a patient specific manner [1]. The yeast delivery vehicle in this trial has previously induced immunogenicity against tumor associated antigens in previous clinical studies [2]. Trial sites are being actively recruited and activated.

**Conclusions**

Neoepitopes have been successfully identified using tumor-normal next generation sequencing. Personalized neoepitope technology promises significant advances against cancer. Data from this trial will establish safety and an immunological strategy to direct patients with cancer towards durable responses.

**Trial Registration**

NCT03552718

**References**

1. Würfel F, Erber R, Huebner H, Hein A, Lux MP, Jud S, Kremer A, Kranich H, Mackensen A, Haberle L, Hack C, Rauh C, Wunderle M, Gaß P, Rabizadeh S, Brandl A, Langemann H, Volz B, Nabieva N, Schulz-Wendtland R, Dudziak D, Beckmann MW, Hartmann A, Fasching P, Rübner M. TILGen: A Program to Investigate Immune Targets in Breast Cancer Patients – First Results on the Influence of Tumor-Infiltrating Lymphocytes. Breast Care. 2018; 13:8-14.

2. King T, Guo Z, Hermreck M, Bellgrau D, Rodell T. Construction and Immunogenicity Testing of Whole Recombinant Yeast-Based T-Cell Vaccines. Methods Mol. Biol. 2016; 1404:529-545.

**Ethics Approval**

This study was approved by IRB company, approval number 2018-0033-CSSIFM.

#### P312 Withdrawn

#### P313 INCMGA 0012-201: A phase 2 study of INCMGA00012 in patients with metastatic Merkel cell carcinoma (mMCC)

##### Geoffrey Gibney, MD^1^, Inderjit Mehmi, MD^2^, Sadhna Shankar^3^, Jeffrey Marine^3^, Chuan Tian^3^, Igor Puzanov, MD, MSCI, FACP^4^

###### ^1^MedStar Georgetown Cancer Institute, Washington, DC, USA; ^2^WVU Cancer Institute, Morgantown, WV, USA; ^3^Incyte Corporation, Wilmington, DE, USA; ^4^Roswell Park Comprehensive Cancer Center, Buffalo, NY, USA

####### **Correspondence:** Geoffrey Gibney (geoffrey.t.gibney@gunet.georgetown.edu)

**Background**

INCMGA00012 is a humanized IgG4 monoclonal antibody against human PD-1 that lacks antibody-dependent cell-mediated cytotoxicity directed against effector lymphocytes. It has demonstrated acceptable tolerability with no dose-limiting toxicity at doses up to 10 mg/kg administered every 2 weeks in an ongoing phase 1 study. Clinical activity has been seen in multiple tumor types. Full and sustained receptor occupancy of INCMGA00012 on both CD4+ and CD8+ T cells along with complete loss of competing fluorescently labeled anti–PD-1 staining (eJBio105 clone) were seen at all dose levels evaluated in the phase 1 study.In patients with mMCC, response rates decline with increasing number of previous chemotherapy regimens [1]. Blockade of the PD-1/PD-L1 pathway has resulted in objective responses in up to 60% of chemotherapy-naive patients. Approximately 30% of chemotherapy- refractory patients showed an objective response to treatment with the anti–PD-L1 antibody avelumab. Additionally, there is no clear correlation of response rates with PD-L1 or Merkel cell polyomavirus status of the tumor.INCMGA00012 offers the convenience of once every 4 weeks (Q4W) flat dosing.

**Methods**

This is a phase 2, open-label study designed to characterize the efficacy and safety of INCMGA00012 in patients with mMCC. The primary endpoint is the overall response rate (ORR) in chemotherapy-naive patients with mMCC. The secondary endpoints include ORR in the full study population (chemotherapy-naive plus chemotherapy- refractory patients). Other secondary endpoints consist of duration of response, disease control rate, progression-free survival, and overall survival in the chemotherapy-naive patients alone, and the full study population. Patients with mMCC and measurable disease per RECIST v1.1, who have not been previously treated with any anti–PD-1 or anti– PD-L1 therapy and are either chemotherapy-naive or have received no more than 3 prior chemotherapy regimens, are eligible for participation. Approximately 90 patients will be enrolled globally. INCMGA00012 will be administered at a flat dose of 500 mg as an intravenous infusion over 60 minutes Q4W. Disease assessments will be conducted every 8 weeks. Study treatment may continue up to 2 years if there is no unacceptable drug-related toxicity or disease progression.

**Trial Registration**

NCT03599713

**References**

1. Colunga A, Pulliam T, Nghiem P. Merkel cell carcinoma in the age of immunotherapy: Facts and hopes. Clin Cancer Res. 2018; 24:2035-2043.

**Ethics Approval**

The study was approved by institutional review boards or independent ethics committees of participating institutions.

#### P314 An open-label, phase 1B study of NEO-PV-01 with Pembrolizumab plus chemotherapy in patients with advanced or metastatic nonsquamous Non-small Cell Lung Cancer

##### Mark Awad, MD PhD^1^, David Spigel, MD^2^, Lisa Cleary^3^, Melissa Moles^3^, Richard Gaynor, MD^3^, Matthew Goldstein, MD, PhD^3^, Ramaswamy Govindan, MD^4^

###### ^1^Dana Farber Cancer Institute, Boston, MA; ^2^Sarah Cannon Cancer Institute, Nashville, TN, USA; ^3^Neon Therapeutics, Cambridge, MA, USA; ^4^Washington University in St. Louis, Saint Louis, MO, USA

####### **Correspondence:** Mark Awad (Mark_Awad@dfci.harvard.edu)

**Background**

Neoantigens arise from unique DNA mutations in cancer cells and are attractive targets for tumor directed immune responses. The anti-tumor activity of checkpoint inhibitors has been associated with tumor mutational burden as well as neoantigen load. The combination of carboplatin/pemetrexed plus anti-PD1 therapy for the treatment of front-line non-squamous NSCLC has demonstrated improved efficacy over chemotherapy alone [1]. This combination reduces the risk of early progression and may also modulate the tumor microenvironment. Vaccines targeting neoantigens offer a rational combination with chemotherapy and anti-PD1 as a highly specific way to induce de novo T cell reactivity and to expand existing T cell responses against neoantigens. Here, we describe a clinical trial combining NEO-PV-01, a personal neoantigen vaccine designed specifically for the molecular profile of each individual’s tumor, with carboplatin/pemetrexed plus anti-PD1.

**Methods**

NT-002 is a single-arm, phase 1B study designed to evaluate the safety of administering NEO-PV-01 + adjuvant (Poly-ICLC) with pembrolizumab plus carboplatin and pemetrexed in patients with advanced non-squamous non- small cell lung carcinoma who have received no prior systemic treatment. Patients undergo a baseline tumor biopsy and HLA typing. DNA and RNA sequencing are performed on tumor as well as peripheral blood to serve as normal DNA controls. NEO-PV-01 is custom designed for each individual patient and contains up to 20 peptides 14-35 amino acids in length. The peptides are pooled into four groups and mixed with the adjuvant Poly-ICLC at the time of subcutaneous administration. On Day 1, patients will begin with 4 treatment cycles of pembrolizumab plus chemotherapy. Beginning on Cycle 5 (Week 12), patients will receive pembrolizumab monotherapy Q3W up to Week 103. Also beginning at Week 12, patients receive five priming immunizations with NEO-PV-01 over a three- week period followed by booster vaccinations at Weeks 19 and 23. The primary endpoint is safety. Secondary endpoints are ORR, CBR, PFS, and assessment of response conversion between Week 12 and Week 24. Exploratory endpoints include extensive immune monitoring with antigen-specific analyses over multiple timepoints of both peripheral blood and tumor.

**Trial Registration**

NCT03380871

**References**

1. Gandhi L, et. al. Pembrolizumab plus chemotherapy in metastatic Non–Small-Cell Lung Cancer. New England Journal of Medicine. 2018; 378:2078-2092

**Ethics Approval**

This study was approved by institutional ethic's boards at the Dana Farber Cancer Institute, Washington University in St. Louis, and Sarah Cannon Cancer Center.

#### P315 Phase I/II study of the anti–LAG-3 antibody MK-4280 in combination with pembrolizumab for the treatment of hematologic malignancies

##### Gareth Gregory^1^, Pier Zinzani^2^, John Palcza, MS^3^, Jane Healy^3^, Robert Orlowski, MD^3^, Arun Balakumaran, PhD, MD^3^, Philippe Armand^4^

###### ^1^School of Clinical Sciences at Monash Health, Monash University, Melbourne, Austrailia; ^2^Institute of Hematology “Seràgnoli” University of Bologna, Bologna, Italy; ^3^Merck & Co., Inc., Kenilworth, NJ, USA; ^4^Dana-Farber Cancer Institute, Boston, MA, USA

####### **Correspondence:** Gareth Gregory (gareth.gregory@petermac.org)

**Background**

Most lymphomas, including classic Hodgkin lymphoma (cHL), diffuse large B-cell lymphoma (DLBCL), and indolent B-cell lymphomas are not readily curable in the relapsed/refractory (R/R) setting, and new options for treating those malignancies are urgently needed. Pembrolizumab, a humanized, high-affinity antibody against programmed death 1 (PD-1), has demonstrated effective antitumor activity and acceptable safety in patients with R/R cHL and R/R primary mediastinal large B-cell lymphoma. Lymphocyte activation gene-3 (LAG-3) is a cell surface immunomodulatory receptor commonly co-expressed with PD-1 on exhausted T cells and may serve as an escape pathway for lymphoma subjected to PD-1 blockade. Dual blockade of PD-1 and LAG-3 demonstrated synergistic activity in mouse models of colon adenocarcinoma and fibrosarcoma. MK-4280 is a humanized anti– LAG-3 monoclonal antibody that blocks the interaction between LAG-3 and its ligand MHC class II. This study will evaluate the safety and efficacy of MK-4280 plus pembrolizumab in patients with selected hematologic malignancies.

**Methods**

This phase 1/2 multisite study (ClinicalTrials.gov: NCT03598608) will enroll patients with PD-1/PD-L1 inhibitor–naive cHL (cohort 1), PD-1/PD-L1 inhibitor–refractory R/R cHL (cohort 2), R/R DLBCL (cohort 3), and R/R indolent B-cell lymphoma (cohort 4). The study will have a safety lead-in phase to establish the preliminary recommended phase 2 dose (RPTD) followed by an efficacy expansion phase. In the safety lead-in phase, a modified toxicity probability interval design will be used to establish RPTD of MK-4280 plus pembrolizumab. Dose-limiting toxicities will be assessed during the first cycle. Eligibility criteria are age ≥18 y, ECOG PS 0/1, adequate organ function, and meeting the standard eligibility criteria for pembrolizumab studies, such as no prior receipt of anti–PD-1 antibody and no active infection necessitating systemic therapy. Patients will receive pembrolizumab 200 mg Q3W and MK-4280 for 35 cycles or until disease progression, unacceptable toxicity, or withdrawal from study. Tumor response will be assessed by CT/PET Q12W to confirm complete response or as clinically indicated, using revised response criteria for malignant lymphoma. Patients will be monitored for adverse events (AEs) until 30 days after study treatment end (90 days for serious AEs). The primary objective of this study is to determine the safety, tolerability, and to establish a preliminary RPTD. Secondary end points include objective response rate per investigator review and pharmacokinetics of MK-4280 and pembrolizumab. The safety lead-in phase will enroll ≥14 patients and the efficacy expansion phase will enroll approximately 120 patients (30 per cohort).

**Trial Registration**

ClinicalTrials.gov: NCT03598608

**Ethics Approval**

The protocol and its amendments were approved by the appropriate institutional review board or independent ethics committee.

**Consent**

All patients will provide written informed consent.

#### P316 Phase 1b dose-escalation and dose-expansion study to evaluate safety, tolerability, pharmacokinetics, and antitumor activity of ADCT-301 (Camidanlumab Tesirine) in patients with advanced solid tumors

##### Igor Puzanov^1^, Shivaani Kummar, MD^2^, Patricia LoRusso, DO^3^, Kyri Papadopoulos^4^, Francesca Zammarchi, PhD^5^, Hans Cruz^5^, Jens Wuerthner, MD PhD^5^, Johanna Bendell, MD^6^

###### ^1^Roswell Park Comprehensive Cancer Center, Buffalo, NY, USA, Buffalo, NY, USA; ^2^Stanford University, CA, USA, Stanford, CA; ^3^Yale Cancer Center, New Haven, CT, USA, New Haven, CT, USA; ^4^South Texas Accelerated Research Therapeutics (START), San Antonio, TX, USA; ^5^ADC Therapeutics, London, UK; ^6^Sarah Cannon Research Institute, Tennessee Oncology, Nashville, TN, USA, Nashville, TN, USA

####### **Correspondence:** Igor Puzanov (igorpuza@buffalo.edu)

**Background**

CD25+ regulatory T-cells (Tregs) suppress tumor-specific T-cell–mediated immune responses and contribute to cancer progression [1]. Modifying the intra-tumoral balance of effector T-cells and Tregs by blocking or depleting CD25+ Tregs is a strategy for tumor eradication, either as a standalone approach or in combination with other immuno-oncology therapies [1,2]. ADCT-301 (camidanlumab tesirine [Cami-T]), an anti-CD25 human monoclonal antibody conjugated to a potent pyrrolobenzodiazepine dimer toxin (Figure 1), is a candidate for selective depletion of tumor-infiltrating CD25+ Tregs. A surrogate of Cami-T, sur301, has shown strong and durable antitumor activity in mouse colon adenocarcinoma-derived models. This study aims to characterize the safety and tolerability of Cami- T in patients with selected advanced solid tumors. A separate study of Cami-T in patients with relapsed/refractory Hodgkin lymphoma and non-Hodgkin lymphoma (NCT02432235) is ongoing.

**Methods**

This is a Phase 1b, multicenter, open-label, dose-escalation and dose-expansion study planned for adult patients with locally-advanced or metastatic solid tumors. Patients should be refractory to or intolerant of existing therapies. The primary objective of the study is to characterize the safety and tolerability of Cami-T and to identify the recommended dose(s) and schedule(s) for future studies. Secondary objectives are to evaluate the preliminary antitumor activity, pharmacokinetics and immunogenicity of Cami-T. A fresh tumor biopsy for biomarker investigations is required to participate. Based on the literature on CD25+ Treg tumor content, patients with the following tumor types will be eligible: head and neck, non-small cell lung, gastric and esophageal, pancreas, bladder, renal, melanoma, triple negative breast, and ovarian cancers. Patients who had a prior organ or allogeneic bone marrow transplant, history of symptomatic autoimmune disease, significant medical comorbidities, or received major surgery or antitumor therapies within the last 14 days will be excluded. The first cohort will receive a dose of 20 μg/kg every 3 weeks, selected as the minimum dose with potential antitumor activity based on mouse models and data from the Phase I study of Cami-T in hematological malignancies. Subsequent dose escalation up to 300 μg/kg is planned to identify the recommended dose(s) for expansion. The study plans to enroll up to 50 patients with an expected start in Q3 2018.

**Conclusions**

A new Phase 1 study is planned to assess the safety and preliminary efficacy of Cami-T for the management of advanced solid tumors.

**Acknowledgements**

Study sponsored by ADC Therapeutics

**References**

1. Sasidharan Nair V, Elkord E. Immune checkpoint inhibitors in cancer therapy: a focus on T-regulatory cells. Immunol Cell Biol. 2018;96:21–33.

2. Arce Vargas F, Furness AJS, Solomon I, et al. Fc-optimized anti-CD25 depletes tumor-infiltrating Regulatory T cells and synergizes with PD-1 blockade to eradicate established tumors. Immunity. 2017;46:577–586.

**Fig. 1 (abstract P316).**
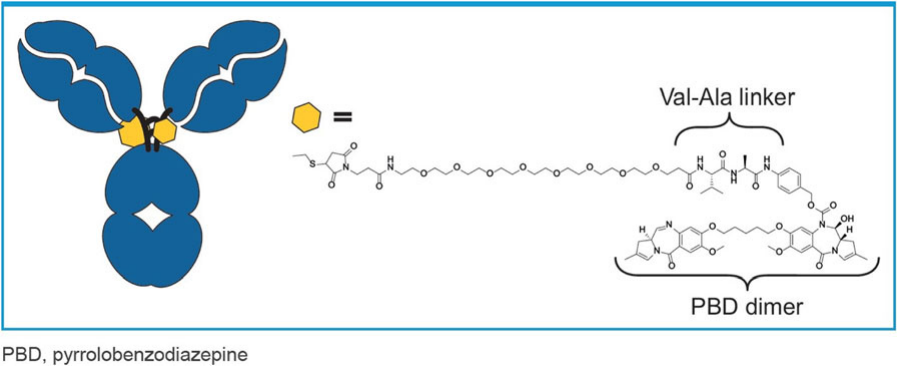
See text for description.

#### P317 Withdrawn

#### P318 HITM-SURE: Phase Ib CAR-T hepatic artery infusion trial for stage IV adenocarcinoma using Pressure- Enabled Drug Delivery technology

##### Steven Katz, MD^1^, Prajna Guha, PhD^2^, John Hardaway, MD, PhD^2^, Ethan Prince, MD^3^, Ashley Moody, BSN, RN^2^, Jill Slansky, PhD^4^, Kimberly Jordan, PhD^4^, Richard Schulick, MD, FACS^4^, Robert Knight, MD^5^, Jerry Zeldis, MD, PhD^5^, Vincent Armenio, MD^1^, N. Joseph Espat, MD, FACS^1^, Richard Junghans, PhD, MD^6^

###### ^1^Roger Williams Medical Center, Boston University, Providence, RI, USA; ^2^Roger Williams Medical Center, Providence, RI, USA; ^3^Roger Williams Medical Center, Warren Alpert Medical School of Brown University, Providence, RI, USA; ^4^University of Colorado, Aurora, CO, USA; ^5^Sorrento Therapeutics, Inc., San Diego, CA, USA; ^6^Immunotherapy Consultations, Boston, MA, USA

####### **Correspondence:** Steven Katz (skatz@chartercare.org)

**Background**

Prior Hepatic Immunotherapy for Metastases (HITM) phase I/Ib studies demonstrated the safety and biologic activity of anti-CEA CAR-T cell hepatic artery infusions (HAI) for CEA+ liver metastases (LM). Here we report preliminary HITM-SURE data using Pressure-Enabled Drug Delivery (PEDD) technology for HAI to overcome high intra-tumoral pressures.

**Methods**

Candidates had unresectable CEA+ LM and failed > 1 line of systemic chemotherapy. Enrolled patients received 3 HAI of 10^10 second generation (IgCD28TCR) anti-CEA CAR-T cells (Sorrento Therapeutics) via a PEDD (Surefire Medical) device and low dose IL-2 (50,000 IU/kg/day). Objectives were to evaluate the safety profile of CAR-T HAI with PEDD and to secondarily assess clinical response.

**Results**

At study conclusion, 4 male and 1 female pts completed treatment—mean age 56.3 yrs (38-64) with 1-2 lines of prior chemotherapy. There was an average of 7.4 LM with an average maximal diameter of 2.8 cm. Mean CAR expression was 68.1% and production time of 14.6 d. In vitro targeted cytotoxicity was 53.5%. Reduction in serum CEA was observed in all pts (avg decrease 15 ng/mL, range 3-39 ng/mL). Compared to previous HITM CAR-T HAI trials with a standard catheter, PEDD significantly increased the frequency of CAR-T 5.2-fold within LM, as detected by quantitative PCR (p=0.03). No Grade (G) 4 or 5 AEs related to CAR-T HAIs via PEDD were detected. G1/2/3 events were largely attributed to IL-2 infusion and were comparable to prior HITM studies. One pt experienced grade 3 colitis, which resolved with IL-2 dose reduction and had colon biopsies that were negative for CAR-T by PCR and immunofluorescence. Twelve-month follow-up imaging in one pt with stage IV pancreatic carcinoma revealed no evidence of LM on PET and his primary pancreatic tumor was stable. Serum and LM biopsies from this pt reveal increased expression of IFN-g and IL-6 in LM, with decreased expression of IL-17, PD- L1, IDO and GM-CSF. A second pt with stage IV pancreatic cancer had no evidence of LM on PET at 6 weeks following CAR-T infusions. Average overall survival post-treatment is 8.3 mo (2.3-16.3) with 4 pts alive at the time of data evaluation.

**Conclusions**

Early results from the HITM-SURE study indicate that HAI of CAR-T using PEDD is well tolerated and results in encouraging activity against CEA+ LM. The median OS compares favorably with prior HITM studies and presently approved second/third line regimens. Final results will inform design and device choice for larger studies.

**Trial Registration**

NCT02850536

**Ethics Approval**

The study was approved by the Roger Williams Medical Center institutional review board, approval number 16-350-74.

#### P319 PD-1 Blockade with pembrolizumab during concurrent chemoradiation for locally advanced non-small cell lung cancer

##### Salma Jabbour, MD^1^, Abigail Berman^2^, Roy Decker, MD PhD^3^, Andrew Zloza^1^, Steven Feigenberg^2^, Scott Gettinger, MD^3^, Charu Aggarwal, MD MPH^2^, Corey Langer, MD^2^, Charles Simone, MD^4^, Jeffrey Bradley^5^, Yong Lin^1^, Salma Jabbour, MD^6^, Jyoti Malhotra, MD^1^

###### ^1^Rutgers Cancer Institute of New Jersey, New Brunswick, NJ, USA; ^2^University of Pennsylvania, Philadelphia, PA, USA; ^3^Yale University, New Haven, CT, USA; ^4^University of Maryland, Baltimroe, MD, USA; ^5^Washington University, St. Louis, MO, USA; ^6^Rutgers Cancer Institute, New Brunswick, NJ, USA

####### **Correspondence:** Salma Jabbour (jabbousk@cinj.rutgers.edu)

**Background**

Despite the curative potential of chemoradiation therapy (CRT) for inoperable, locally advanced non-small cell lung cancer (LA-NSCLC), the historical 2- year overall survival rate is only 55-60%. A recent trial showed that Programmed Death Ligand 1 (PD-L1) blockade in Stage III NSCLC improves progression-free survival when used as consolidation after CRT. Limited data exist about incorporation of PD-L1 inhibition during CRT. We sought to assess the safety and toxicity of PD-1 inhibition using pembrolizumab during CRT for LA-NSCLC.

**Methods**

In this multi-center Phase I clinical trial using a 3+3 trial design, for Stage III NSCLC, we evaluated the timing and dosing of pembrolizumab with concurrent chemotherapy (carboplatin + paclitaxel weekly) and RT (60 Gy in 2 Gy/day). In Regimen 1, we started pembrolizumab at full dose (200 mg IV Q3weeks) 56-84 days after the first day of CRT (2-6 weeks after CRT end). In Regimen 2: we gave pembrolizumab at reduced dose (100 mg IV Q3weeks) throughout the full course of treatment starting on Day 28 of CRT. In Regimen 3: we gave pembrolizumab at full dose starting on Day 28 of CRT. In Regimen 4: we gave pembrolizumab at reduced dose starting on Day 0. In Regimen 5: we gave pembrolizumab at full dose starting on Day 0. In all regimens, we continued pembrolizumab for up to 18 doses and delivered pembrolizumab as monotherapy after CRT. Dose Limiting Toxicity (DLT) was defined as Grade ≥4 Pneumonitis.

**Results**

We enrolled 19 subjects from 8/2016-7/2018, and 15 received pembrolizumab on Regimens 1-5. In Regimen 1, the DLT assessment period could last up to 15 weeks from the Day 0 of CRT. Median age was 71 years (range 53-84 years). We observed no DLT upon completion of Regimen 5 or in any of the cohorts. Therefore, we recommended the dose for further study as CRT + pembrolizumab 200 mg IV on the first day of CRT. Treatment-related adverse events (AE) included: Grade 2 nephritis (n=1, Regimen 1); Grade 3 pneumonitis (n=1, Regimen 2; 7 months after CRT); Grade 3 hyperglycemia (n=1, Regimen 5). We continue for an enrollment of an additional 6 patients on Regimen 5.

**Conclusions**

In evaluating pembrolizumab during CRT for LA-NSCLC, we did not observe any DLT of Grade ≥4 Pneumonitis. We plan to perform additional correlative testing including, baseline PD-L1 status and CD8+ T cell vs CD4+FoxP3+ T cell infiltration during therapy.

**Trial Registration**

NCT02621398

**Ethics Approval**

The study was approved by the Rutgers Institutional Review Board, approval number Pro20150002247.

**Consent**

Written informed consent was obtained from the patient for publication of this abstract and any accompanying images. A copy of the written consent is available for review by the Editor of this journal

#### P320 A phase 1/1b study to evaluate the safety and tolerability of AB928, a novel dual adenosine receptor antagonist, in combination with chemotherapy in patients with breast or gynecologic malignancies

##### Paul de Souza, MBBS, MPH, PHD, FRACP^1^, Chee Khoon Lee^2^, Katrin Sjoquist^2^, Shu Pan^3^, Amanda Idan^3^, Aimee Rieger, BS^4^, Wade Berry, BA^4^, Lixia Jin^4^, Lisa Seitz, MSc^4^, Devika Ashok, PhD^4^, Matthew Walters, PhD^4^, Dana Piovesan, MSc^4^, Joanne Tan, PhD^4^, Susan Lee, PhD^4^, Adam Park, BS^4^, Daniel DiRenzo, PhD^4^, Joyson Karakunnel, MD, MSc^4^

###### ^1^University of Western Sydney, Sydney, Australia; ^2^St George Public Hospital, Kogarah, Australia; ^3^St George Private Hospital, Kogarah, Australia; ^4^Arcus Biosciences, Inc., Hayward, CA, USA

####### **Correspondence:** Joyson Karakunnel (jkarakunnel@gmail.com)

**Background**

In many tumors, extracellular adenosine contributes to an immunosuppressed tumor microenvironment via activation of the A2a receptor (A2aR) and A2b receptor (A2bR) expressed on intratumoral immune cells. AB928 is a novel, selective, small molecule antagonist of both A2aR and A2bR with the ability to potently block the immunosuppressive effects of high concentrations of adenosine in the tumor microenvironment. AB928 differs from other known A2aR antagonists based on its dual mode of action, its minimal loss of potency due to nonspecific binding to plasma proteins, and its lack of penetrance through a healthy blood-brain barrier. Preclinically, combining adenosine receptor inhibition with either chemotherapy or anti-programmed cell death-1 resulted in greater tumor control in mouse models, suggesting that AB928 may have synergistic activity when paired with either chemotherapy or checkpoint inhibitors in oncology patients. A phase 1 study with AB928 in healthy volunteers has been completed.

**Methods**

A phase 1/1b, open-label, dose-escalation (3+3 design) study is evaluating the safety/tolerability (including dose-limiting toxicities), pharmacokinetics, pharmacodynamics, and clinical activity of AB928 in combination with pegylated liposomal doxorubicin (PLD) in patients with breast or gynecologic malignancies. Patients are eligible if they have pathologically confirmed triple-negative breast cancer or ovarian cancer that is metastatic, advanced or recurrent with progression for which no alternative or curative therapy exists. Patients may have received up to 5 lines of prior therapies for advanced/recurrent and progressive disease (unlimited number of hormonal therapies permitted). AB928 is administered orally once daily at a starting dose of 75 mg (Cohort 1) escalating to 150 mg (Cohort 2) and 200 mg (Cohort 3), and PLD is administered at the standard regimen of 40 mg/m2 intravenously every 4 weeks. Intermediate AB928 doses may be evaluated based on data from cohorts that have been explored. Doses that have exceeded the maximum tolerated dose will not be evaluated. Following identification of the recommended phase 2 dose of AB928 and PLD during dose escalation, each tumor cohort (triple-negative breast and ovarian cancer) may be expanded to further evaluate the combination. The primary endpoint is safety/tolerability, and secondary endpoints are pharmacokinetics, pharmacodynamics (receptor occupancy in peripheral blood and immunomodulatory activity in select immune subsets), and clinical activity (objective response rate, duration of response, disease control rate [complete response, partial response, or stable disease for >6 months], and progression-free survival per Response Evaluation Criteria in Solid Tumors v1.1, and overall survival). The study is recruiting.

**Ethics Approval**

This study was approved by Bellberry Human Research Ethics committee (HREC), 129 Glen Osmond Road Eastwood South Australia 5063; Institutional Review Board; approval number 2018-04-306.

#### P321 A phase 1/1b study to evaluate the safety and tolerability of AB928, a novel dual adenosine receptor antagonist, in combination with chemotherapy in patients with gastrointestinal malignancies

##### Paul de Souza, MBBS, MPH, PHD, FRACP^1^, Chee Khoon Lee^2^, Katrin Sjoquist^2^, Shu Pan^3^, Amanda Idan^3^, Aimee Rieger, BS^4^, Wade Berry, BA^4^, Lixia Jin^4^, Lisa Seitz, MSc^4^, Devika Ashok, PhD^4^, Matthew Walters, PhD^4^, Dana Piovesan, MSc^4^, Joanne Tan, PhD^4^, Susan Lee, PhD^4^, Adam Park, BS^4^, Daniel DiRenzo, PhD^4^, Joyson Karakunnel, MD, MSc^4^

###### ^1^University of Western Sydney, Sydney, Australia; ^2^St George Public Hospital, Kogarah, Australia; ^3^St George Private Hospital, Kogarah, Australia; ^4^Arcus Biosciences, Inc., Hayward, CA, USA

####### **Correspondence:** Joyson Karakunnel (jkarakunnel@gmail.com)

**Background**

In many tumors, extracellular adenosine contributes to an immunosuppressed tumor microenvironment via activation of the A2a receptor (A2aR) and A2b receptor (A2bR) expressed on intratumoral immune cells. AB928 is a small molecule A2aR and A2bR antagonist with the ability to potently block the immunosuppressive effects of high adenosine concentrations in the tumor microenvironment. Preclinically, combining adenosine receptor inhibition with either chemotherapy or anti-programmed cell death-1 resulted in greater tumor control in mouse models, suggesting AB928 may have synergistic activity when paired with either chemotherapy or checkpoint inhibitors in oncology patients. A phase 1 study with AB928 in healthy volunteers has been completed.

**Methods**

A phase 1/1b, open-label, dose-escalation (3+3 design) study is evaluating the safety/tolerability, pharmacokinetics, pharmacodynamics, and clinical activity of AB928 in combination with mFOLFOX in patients with gastrointestinal malignancies. Patients are eligible if they have histologically confirmed gastroesophageal or colorectal cancer that is metastatic or locally advanced and unresectable for which no alternative or curative therapy exists, or standard therapy is not considered appropriate by the participant and treating physician. Patients must have received ≤5 lines of prior therapies and must not have received prior oxaliplatin treatment, except those who have received prior oxaliplatin-based therapy as the most recent regimen in the adjuvant setting if completed ≥6 months prior to enrollment. AB928 is administered orally once daily at a starting dose of 75 mg (Cohort 1) escalating to 150 mg (Cohort 2) and 200 mg (Cohort 3), and mFOLFOX is administered at the standard regimen (oxaliplatin 85 mg/m2 intravenously [IV] every 2 weeks, leucovorin 400 mg/m2 IV every 2 weeks, and 5-fluorouracil 400 mg/m2 IV bolus + 2400 mg/m2 [continuous 46-hour infusions on Days 1 and 2]). Intermediate AB928 doses may be evaluated based on data from cohorts that have been explored. Doses that have exceeded the maximum tolerated dose will not be evaluated. Following identification of the recommended phase 2 dose of AB928 and mFOLFOX during dose escalation, each tumor cohort (gastroesophageal and colorectal cancer) may be expanded to further evaluate the combination. The primary endpoint is safety/tolerability and secondary endpoints are pharmacokinetics, pharmacodynamics (receptor occupancy in peripheral blood and immunomodulatory activity in select immune subsets), and clinical activity (objective response rate, duration of response, disease control rate [complete response, partial response, or stable disease for >6 months], progression-free survival per Response Evaluation Criteria in Solid Tumors v1.1, and overall survival). The study is recruiting.

**Ethics Approval**

This study was approved by Bellberry Human Research Ethics committee (HREC), 129 Glen Osmond Road Eastwood South Australia 5063; Institutional Review Board; approval number 2018-04-307.

#### P322 A phase 1/1b study to evaluate the safety and tolerability of AB928, a novel dual adenosine receptor antagonist, in combination with carboplatin/pemetrexed and pembrolizumab in lung cancer patients

##### Paul de Souza, MBBS, MPH, PHD, FRACP^1^, Chee Khoon Lee^2^, Katrin Sjoquist^2^, Shu Pan^3^, Amanda Idan^3^, Aimee Rieger, BS^4^, Wade Berry, BA^4^, Lixia Jin^4^, Lisa Seitz, MSc^4^, Devika Ashok, PhD^4^, Matthew Walters, PhD^4^, Dana Piovesan, MSc^4^, Joanne Tan, PhD^4^, Susan Lee, PhD^4^, Adam Park, BS^4^, Daniel DiRenzo, PhD^4^, Joyson Karakunnel, MD, MSc^4^

###### ^1^University of Western Sydney, Sydney, Australia; ^2^St George Public Hospital, Kogarah, Australia; ^3^St George Private Hospital, Kogarah, Australia; ^4^Arcus Biosciences, Inc., Hayward, CA, USA

####### **Correspondence:** Joyson Karakunnel (jkarakunnel@gmail.com)

**Background**

In many tumors, extracellular adenosine contributes to an immunosuppressed tumor microenvironment via activation of the A2a receptor (A2aR) and A2b receptor (A2bR) expressed on intratumoral immune cells. AB928 is a novel, selective, small molecule antagonist of both A2aR and A2bR with the ability to potently block the immunosuppressive effects of high concentrations of adenosine in the tumor microenvironment. Preclinically, combining adenosine receptor inhibition with either chemotherapy or anti-programmed cell death-1 has resulted in greater tumor control in mouse models, suggesting that AB928 may have synergistic activity when paired with either chemotherapy or checkpoint inhibitors in oncology patients. A phase 1 study with AB928 in healthy volunteers has been completed.

**Methods**

A phase 1/1b, open-label, dose-escalation (3+3 design) and dose-expansion study was designed to evaluate the safety/tolerability (including dose-limiting toxicities), pharmacokinetics, pharmacodynamics, and clinical activity of AB928 in combination with standard-of-care carboplatin/pemetrexed plus pembrolizumab [1] in patients with nonsquamous non small-cell lung cancer (NSCLC). Patients are eligible if they have pathologically confirmed nonsquamous NSCLC that is metastatic or recurrent with progression. Patients may have received up to 5 lines of prior therapies in dose escalation and up to 3 lines of prior therapies in dose expansion. AB928 is administered orally once daily at a starting dose of 75 mg (Cohort 1) escalating to 150 mg (Cohort 2) and 200 mg (Cohort 3), and carboplatin/pemetrexed plus pembrolizumab is administered at the standard regimen (carboplatin: AUC 5 mg/mL/min intravenously [IV] every 3 weeks [Q3W]; pemetrexed: 500 mg/m2 IV Q3W; pembrolizumab 200 mg IV Q3W). Intermediate AB928 doses may be evaluated based on data from cohorts that have been explored. Doses that have exceeded the maximum tolerated dose will not be evaluated. Following identification of the recommended phase 2 dose of AB928 in combination with carboplatin/pemetrexed plus pembrolizumab during dose escalation, the lung cancer cohort will be expanded to further evaluate this combination. The combination of AB928 and carboplatin/pemetrexed without pembrolizumab may also be evaluated in dose expansion. The primary endpoint is safety/tolerability and secondary endpoints are pharmacokinetics, pharmacodynamics (receptor occupancy in peripheral blood and immunomodulatory activity in select immune subsets), and clinical activity (objective response rate, duration of response, disease control rate [complete response, partial response, or stable disease for >6 months], and progression-free survival per Response Evaluation Criteria in Solid Tumors v1.1, and overall survival). FDA submission completed with start-up for recruitment ongoing.

**References**

1. Gandhi L, Rodriguez-Abreu D, Gadgeel S, et al. Pembrolizumab plus chemotherapy in metastatic non-small-cell lung cancer. N Engl J Med. 2018;378:2078-2092.

#### P323 A phase 1 study to evaluate the safety and tolerability of AB928, a novel dual adenosine receptor antagonist, with AB122, a programmed cell death-1 inhibitor, in patients with advanced malignancies

##### Lisa Seitz, MSc, Aimee Rieger, BS, Wade Berry, BA, Lixia Jin, Devika Ashok, PhD, Matthew Walters, PhD, Dana Piovesan, MSc, Joanne Tan, PhD, Susan Lee, PhD, Adam Park, BS, Daniel DiRenzo, PhD, Joyson Karakunnel, MD, MSc

###### Arcus Biosciences, Inc., Hayward, CA, USA

####### **Correspondence:** Joyson Karakunnel (jkarakunnel@gmail.com)

**Background**

In many tumors, extracellular adenosine contributes to an immunosuppressed tumor microenvironment via activation of the A2a receptor (A2aR) and A2b receptor (A2bR) expressed on intratumoral immune cells. AB928 is a novel, selective, small molecule antagonist of both A2aR and A2bR with the ability to potently block the immunosuppressive effects of high concentrations of adenosine in the tumor microenvironment. Preclinically, combining adenosine receptor inhibition with either chemotherapy or anti-programmed cell death-1 resulted in greater tumor control in mouse models, suggesting that AB928 may have synergistic activity when paired with either chemotherapy or checkpoint inhibitors in oncology patients. A phase 1 study with AB928 in healthy volunteers has been completed. AB122 is a fully human monoclonal antibody targeting PD-1.

**Methods**

A phase 1, open-label, dose-escalation (3+3 design) study is evaluating the safety/tolerability (including dose-limiting toxicities), pharmacokinetics, pharmacodynamics, and clinical activity of AB928 in combination with AB122 in patients with advanced malignancies. Patients are eligible if they have pathologically confirmed non-small cell lung cancer, squamous cell carcinoma of the head and neck, renal cell carcinoma, breast cancer, colorectal cancer, melanoma, bladder cancer, ovarian cancer, endometrial cancer, Merkel cell carcinoma, or gastroesophageal cancer that is metastatic, advanced or recurrent with progression for which no alternative or curative therapy exists.

Patients must have received standard of care, and may have received up to 5 lines of prior therapies. AB928 is administered orally once daily at a starting dose of 75 mg (Cohort 1) escalating to 150 mg (Cohort 2) and 200 mg (Cohort 3), and AB122 is administered intravenously every 2 weeks at 240 mg. Intermediate AB928 doses may be evaluated based on data from cohorts that have been explored; however, doses must not exceed the maximum tolerated dose. Following identification of the recommended phase 2 dose of AB928 and AB122 during dose escalation, select tumor cohorts may be expanded to further evaluate this combination. The primary endpoint is safety/tolerability and secondary endpoints are pharmacokinetics, pharmacodynamics (receptor occupancy in peripheral blood and immunomodulatory activity in select immune subsets), immunogenicity, and clinical activity (objective response rate, duration of response, disease control rate [complete response, partial response, or stable disease for >6 months], and progression-free survival per Response Evaluation Criteria in Solid Tumors v1.1, and overall survival). This study is recruiting.

**Ethics Approval**

This study was approved by Bellberry Human Research Ethics committee (HREC), 129 Glen Osmond Road Eastwood South Australia 5063; Institutional Review Board approval number 2018-05-360.

#### P324 A phase II clinical trial of Ipilimumab/Nivolumab combination immunotherapy in patients with rare upper gastrointestinal, neuroendocrine and gynaecological malignancies

##### Oliver Klein^1,4^, Kee Damien^2^, Ben Markman^3^, Rachael Chang Lee, FRACP^4^, Siddharth Menon^3^, Jodie Palmer, BA/BSc, PhD, Grad Dip Law (IP)^5^, Andreas Behren, PhD^5^, Jonathan Cebon, MD, PhD^4^

###### ^1^Medical Oncology, Austin Health, Heidelberg, Australia; ^2^Peter MacCallum Cancer Centre, Melbourne, Australia; ^3^Monash Medical Centre, Melbourne, VIC, Australia; ^4^Olivia Newton John Cancer Centre, Melbourne, Australia; ^5^Olivia Newton John Cancer Research Insti, Heidelberg, Australia

####### **Correspondence:** Oliver Klein (oliver.klein@onjcri.org.au)

**Background**

Ipilimumab/Nivolumab combination treatment is so far the most efficacious immunotherapy regimen. It has demonstrated significant clinical activity in patients with metastatic melanoma, renal cell carcinoma, microsatellite instable colorectal cancer and non-small cell lung cancer and response rates are higher compared to single agent anti-PD-1 therapy. Patients with rare cancers represent an unmet medical need and have an inferior overall survival compared to patients with more common malignancies. No therapies including immunotherapies have systematically been investigated in this patient population. This trial investigates the efficacy of Ipilimumab/Nivolumab immunotherapy in patients with rare cancers and aims to identify tumour type agnostic biomarkers that can predict for response.

**Methods**

60 patients with advanced rare upper gastrointestinal, neuroendocrine and gynaecological malignancies and an ECOG performance status of 0 or 1 were included in the trial. All patients received nivolumab 3mg/kg and ipilimumab 1mg/kg every 3 weeks for four doses, followed by nivolumab 3mg/kg every 2 weeks. Treatment continued for 96 weeks, until disease progression or the development of unacceptable toxicity. The primary endpoint is clinical benefit rate (overall response rate and stable disease at week 12) with overall survival for each tumour type being a descriptive endpoint. Acquisition of tumour tissue prior to study enrolment was mandatory and a panel of genomic assays and immuno- histochemistry- and - fluorescence are used to identify tumour type agnostic biomarkers

**Background**

Ipilimumab/Nivolumab combination treatment is so far the most efficacious immunotherapy regimen. It has demonstrated significant clinical activity in patients with metastatic melanoma, renal cell carcinoma, microsatellite instable colorectal cancer and non-small cell lung cancer and response rates are higher compared to single agent anti-PD-1 therapy. Patients with rare cancers represent an unmet medical need and have an inferior overall survival compared to patients with more common malignancies. No therapies including immunotherapies have systematically been investigated in this patient population.

**Methods**

60 patients with advanced rare upper gastrointestinal, neuroendocrine and gynaecological malignancies and an ECOG performance status of 0 or 1 were included. All patients received nivolumab 3mg/kg and ipilimumab 1mg/kg every 3 weeks for four doses, followed by nivolumab 3mg/kg every 2 weeks. Treatment continued for 96 weeks, until disease progression or the development of unacceptable toxicity. The primary endpoint is clinical benefit rate (overall response rate and stable disease at week 12) with overall survival for each tumour type being a descriptive endpoint. Acquisition of tumour tissue prior to study enrolment was mandatory and a panel of genomic assays and immuno-histochemistry and -fluorescence are used to identify tumour type agnostic biomarkers.

**Results**

As per 31 July 2018 43 patients have been enrolled and an updated efficacy and safety analysis will be presented at the meeting. So far 23 patients have undergone their first restaging at week 12. The objective response rate for the evaluable patient population is 43 % with responses being seen in a wide range of malignancies including biliary tract cancers, adrenocortical carcinoma, atypical bronchial carcinoid and rare gynaecological cancers as uterine clear cell carcinoma. Grade 3/4 immune related adverse events were detected in 19% of patients.

**Conclusions**

Our preliminary data demonstrate that Ipilimumab/Nivolumab combination treatment has considerable efficacy in a wide range of advanced rare malignancies for which treatments are very limited or no standard treatments are available. The rate of high grade immune related toxicity is in keeping with the experience in previously reported clinical trials using the same dosing regimen. Biomarker studies are ongoing to identify tumour agnostic markers of response.

#### P325 A phase 1 dose escalation study of TSR-033, an anti-LAG3 monoclonal antibody, in patients with advanced solid tumors

##### Kelly Stratton, MD^1^, Aurélien Marabelle, MD PhD^2^, Geoffrey Shapiro, MD, PhD^3^, Jong Chul Park, MD^4^, Solmaz Sahebjam, MD^5^, Srimoyee Ghosh, PhD^6^, Jian Chen^6^, Taylor Eves^6^, Ying Wang^6^, Amita Patnaik, MD FRCP(C)^7^

###### ^1^College of Medicine, The University of Oklahoma, Oklahoma City, OK, USA; ^2^Gustave Roussy, Villejuif Cedex, France; ^3^Dana-Farber Cancer Institute, Boston, MA, USA; ^4^Massachusetts General Hospital, Boston, MA, USA; ^5^Moffitt Cancer Center & Research Institute, University of South Florida, Tampa, FL, USA; ^6^TESARO, Inc., Waltham, MA, USA; ^7^South Texas Accelerated Research Therapeutics, San Antonio, TX, USA

####### **Correspondence:** Kelly Stratton (kelly-stratton@ouhsc.edu)

**Background**

Lymphocyte activation gene 3 (LAG3) is an immune checkpoint receptor found on effector and regulatory T cells that controls T cell response, activation, and growth. Progressive expression of immune checkpoint receptors, including LAG3, contributes to T cell exhaustion and compromises antitumor immune response. LAG3 is frequently co-expressed with PD-1 on tumor-infiltrating T cells across a variety of tumor types and has been shown to interact with major histocompatibility complex (MHC) class II, on antigen-presenting cells, to attenuate T cell activation.TSR-033 is an investigational, humanized, anti-LAG3 monoclonal antibody that blocks the interaction of the LAG3 receptor with MHC class II. LAG3 blockade with TSR-033 in combination with anti-PD-1 therapy (TSR-042) boosts immune function and elicits antitumor immunity in preclinical models. Clinical data demonstrate that combined anti-PD-1 and anti-LAG3 checkpoint blockade leads to increased antitumor activity compared with anti- PD-1 alone in patients who have progressed on anti-PD-1 therapy.

**Methods**

TSR-033 is being investigated in a multicenter, open-label, first-in-human phase 1 trial enrolling patients with advanced or metastatic solid tumors who have progressed after, or are intolerant to, available or approved therapies.

The primary objective of the current part of this study is to evaluate the safety and tolerability of TSR-033 as a monotherapy. Patients received IV infusion of TSR-033 monotherapy every 14 days in 4 escalating dose levels.

**Results**

As of July 13, 2018, 26 patients have been treated with monotherapy: 3 patients at 20 mg, 10 patients at 80 mg, 8 patients at 240 mg, and 5 patients at 720 mg. Adverse events that occurred in >15% of patients were arthralgia (5 patients, 19%), back pain (5 patients, 19%), decreased appetite (5 patients, 19%), and nausea (5 patients, 19%). No adverse events of grade 3 or higher were considered related to study drug. Adverse events occurring in >5% of patients and considered to be immune related were arthralgia (2 patients, 8%). One dose-limiting toxicity (grade 1; myasthenia gravis) occurred with TSR-033 monotherapy at 80 mg. Exposure increased in a dose proportional manner and importantly, receptor occupancy on peripheral T cells increased as the exposure increased, confirming target engagement in the periphery.

**Conclusions**

TSR-033 monotherapy was well tolerated across multiple dose levels. Adverse events were manageable and consistent with the safety profiles of other immune checkpoint inhibitors. Dose escalation of TSR-033 in combination with TSR-042 (anti-PD-1) is ongoing.

**Trial Registration**

clinicaltrials.gov NCT03250832

#### P326 GARNET: Preliminary safety, efficacy, pharmacokinetic, and biomarker characterization from a phase 1 clinical trial of TSR-042 (anti-PD-1 monoclonal antibody) in patients with recurrent/advanced NSCLC

##### Desamparados Roda Perez, PhD, MD^1^, Janakiraman Subramanian^2^, Joanna Pikiel^3^, Maria-Pilar Barretina-Ginesta^4^, José Trigo, MD^5^, Wei Guo^6^, Sharon Lu^6^, David Jenkins, PhD^6^, Kai Yu Jen^6^, Hadi Danaee^6^, Steven Dunlap^6^, Ellie Im^6^, Victor Moreno, PhD, MD^7^

###### ^1^University Hospital, Valencia, Spain; ^2^Saint Luke's Cancer Institute, Kansas City, USA; ^3^Regional Center of Oncology, Gdansk, Poland; ^4^Institut Català d'Oncologia, Hospital Universitari Dr. J. Trueta, Girona, Spain; ^5^Hospital Universitario Virgen de la Victoria, Málaga, Spain; ^6^TESARO, Inc., Waltham, USA; ^7^START Madrid-FJD, Hospital Fundación Jiménez Díaz, Madrid, Spain

####### **Correspondence:** Desamparados Roda Perez (derope@hotmail.com)

**Background**

TSR-042 is a humanized monoclonal antibody targeting programmed death (PD)-1 receptor, effectively blocking interaction with its ligands PD-L1 and PD-L2. TSR-042 is being evaluated in patients with advanced solid tumors in the ongoing phase 1 GARNET trial (NCT02715284). Weight-based dose escalation (part 1) and fixed-dose safety studies (part 2A) have been completed. We present safety/efficacy data from the non-small cell lung cancer (NSCLC) cohort at the recommended phase 2 dose (RP2D).

**Methods**

NSCLC patients with previously treated recurrent/advanced disease were enrolled. Patients received the RP2D of TSR-042: 500 mg Q3W for cycles 1-4 and 1000 mg Q6W thereafter. Serum was collected for pharmacokinetic (PK) analysis. Antitumor activity was assessed with immune-related Response Evaluation Criteria in Solid Tumors (irRECIST). Tumor PD-L1 expression was measured and PD-L1 tumor proportion scores (TPS) were categorized as <1%, 1-49%, and ≥50%.

**Results**

A total of 35 patients were enrolled. The median number of prior lines of therapy for metastatic disease was 1.

Among these patients, 34 (97%) experienced ≥1 treatment-emergent adverse event (AE), 11 (31%) had grade ≥3 AEs, and 1 (3%) experienced a grade ≥3 drug-related AE (fatigue). The most common AEs were fatigue/asthenia (31%), nausea (29%), and diarrhea (20%). Two patients (6%) had drug-related immune-related AEs of hypothyroidism and hyperthyroidism, respectively. No cases of drug-related pneumonitis were observed. PD-L1 TPS results were available for 26 patients; of these only 1 patient (4%) had TPS ≥50%, 25 (96%) had TPS<50%, and 13 (50%) had TPS <1%. In the overall population of 35 patients there were 9 (26%) partial responses (confirmed and unconfirmed), and 11 patients (31%) had stable disease. Of the 9 patients who responded, treatment is ongoing for 7 patients. PK at RP2D was consistent with previous studies.

**Conclusions**

TSR-042 demonstrated robust clinical activity in previously treated recurrent/advanced NSCLC patients whose tumor PD-L1 status was predominantly TPS <50%, including in patients with TPS <1%. Both overall response rate and toxicity appear competitive with approved anti-PD-1 agents studied in the same setting.

**Trial Registration**

clinicaltrials.gov NCT02715284

#### P327 Higher dose single-agent intratumoral G100 (a TLR4 agonist) results in increased biomarker activity and improved clinical outcomes in patients with follicular lymphoma

##### Christopher Flowers, MD, MS^1^, Carlos Panizo^2^, Weiyun Ai, MD, PhD^3^, Iris Isufi, MD^4^, Alex Herrera, MD^5^, Nancy Bartlett, MD^6^, Craig Okada, MD, PhD^7^, Bela Kis, MD, PhD^8^, Luis de la Cruz Merino Sr.^9^, Javier Briones^10^, Jorge Chaves, MD^11^, Elizabeth Cull^12^, Locke Bryan, MD^13^, Roch Houot, MD, PhD^14^, Kim Linton, PhD^15^, Ian Chau^16^, Gottfried von Keudell^4^, Hailing Lu, MD, PhD^17^, Frank Hsu, MD^17^, Ahmad Halwani, MD^18^

###### ^1^Emory University, Atlanta, GA, USA; ^2^Clinica Universidad de Navarra, Pamplona, Spain; ^3^University of California San Francisco, San Francisco, CA, USA; ^4^Yale University, New Haven, CT, USA; ^5^City of Hope, Duarte, CA, USA; ^6^Washington University, St. Louis, MO, USA; ^7^Oregon Health and Science University, OR, USA; ^8^Moffitt Cancer Center, Tampa, FL, USA; ^9^Hospital Universitario Virgen Macarena, Seville, Spain; ^10^Hospital De La Santa Creu I San Pau, Barcelona, Spain; ^11^Northwest Medical Specialties, Tacoma, WA, USA; ^12^Greenville Health System Cancer Institute, Tocoma, WA, USA; ^13^Georgia Cancer Center,Augusta University, Augusta, GA, USA; ^14^Centre Hospitalier Universitaire Pontchaillou, Rennes, France; ^15^The Christie NHS Foundation Trust & The University of Manchester, UK; ^16^Royal Marsden Hospital, London, USA; ^17^Immune Design, Seattle, WA, USA; ^18^Huntsman Cancer Institute, Salt Lake City, UT, USA

####### **Correspondence:** Frank Hsu (Frank.Hsu@immunedesign.com)

**Background**

G100 (G) is a toll-like receptor 4 (TLR4) agonist that activates the innate and adaptive immune system. This ongoing phase 1/2 study demonstrated that intratumoral (IT) G100 alone or with pembrolizumab (P) was safe, induced systemic clinical responses in follicular lymphoma (FL) patients (pts) including relapsed/refractory (R/R) disease. Compared to G alone, the addition of P resulted in a higher response rate, abscopal (untreated sites) tumor shrinkage and increased CD8 tumor infiltrating lymphocytes (TILs), which were associated with clinical responses. An association between baseline tumor TLR4 expression by immunohistochemistry (IHC) and clinical response was observed. We now present clinical and biomarker data for G100 alone at a higher 20μg dose (Part 3) and compare it to 10μg (Part 2).

**Methods**

14 (9 R/R) FL (Part 3) and 13 (7 R/R) FL (Part 2) pts were enrolled. Pts received 6-9 doses of IT G100 weekly to a site treated with low dose radiation (RT, 2Gy x2 doses). A 2nd course of G100 could be given without RT. Responses were evaluated by IrRC criteria. Blood samples and tumor biopsies from treated and/or abscopal sites were obtained pre- and post-G100 for TLR4 expression by IHC, lymphocyte markers and RNA expression.

**Results**

Median observation was 15mo vs 8mo for Part 2 vs Part 3. Best overall response rate (ORR) and 9mo ORR were 23% vs 29% and 15% vs 29% for 10 vs 20μg dose. Abscopal tumor shrinkage (≥10%) was 54% vs 79% (all pts) and 29% vs 78% (R/R pts) at 10μg vs 20μg dose. Among pts with baseline TLR4high (≥50%) tumor expression, ORR was 17% for 10μg (n=6) vs 57% vs for 20μg (n=7) dose. No Grade ≥3 adverse events were observed. At the 20μg dose, a trend toward higher CD8 TILs and greater reduction of CD20+ tumor cells was observed (p=0.03). Clinical response was associated with baseline TLR4high expression (p=0.002) as well as post-G100 increase in CD8 TILs (p=0.02), and a decrease in CD20+ tumor cells (p=0.03) (all pts). G100 significantly increased T- and NK-cell, and macrophage (CD163, CD68, FcgR2A, FcgR3A, IRF1, MRC1, MSR1, granzyme) gene expression (p<0.05) as analyzed by NanoString.

**Conclusions**

G100 20μg dose is safe and demonstrates an early trend toward better clinical responses. This dose induces greater changes in tumor biomarkers that are associated with clinical response (increase of TILs, decrease of CD20+ tumor cells) and supports further development of the 20μg dose of IT G100.

**Trial Registration**

NCT02501473

**Ethics Approval**

The study was approved by participating institutions’ Ethics Board.

#### P328 Preoperative window of opportunity trial of nivolumab with or without tadalafil in patients with squamous cell carcinoma of the head and neck

##### Adam Luginbuhl, MD, Jennifer Johnson, MD, Larry Harshyne, Joseph Curry, MD, Rita Axelrod, MD, Ralph Zinner, MD, Benjamin Leiby, PhD, Madalina Tuluc, MD, Christopher Snyder, David Cognetti, MD, Ulrich Rodeck, MD PhD, Athanassios Argiris, MD PhD

###### Thomas Jefferson Univesity, Philadelphia, PA, USA

####### **Correspondence:** Jennifer Johnson (jennifer.m.johnson@jefferson.edu)

**Background**

Checkpoint modulation to augment or inhibit components of the immune system is of great interest to the clinical management of squamous cell carcinoma of the head and neck (SCCHN). PD-1 blockade using nivolumab has proven to be efficacious in the metastatic and recurrent disease setting, whereas efficacy in treatment naïve patients amenable to curative surgery is yet to be determined. Combining PD-1 blockade with other therapeutic modalities has emerged as a promising strategy in several malignancies. This window of opportunity trial is designed to test molecular and therapeutic effects of the combination of nivolumab with tadalafil. Tadalafil is a phosphodiesterase-5 inhibitor and has been found to augment tumor specific immunity in SCCHN patients. Both in mouse and human studies, tadalafil decreases myeloid-derived suppressor cells (MDSCs) associated with reduced arginase and iNOS expression and intratumoral Treg abundance. Conversely, tadalafil reportedly increases cytotoxic effector T cells (CD8+) within the tumor. The window of opportunity format allow us to relate treatment-associated changes in the tumor environment to therapeutic outcomes.

**Methods**

This is an investigator-initiated, multi-institutional window of opportunity randomized trial in patients with SCCHN, who are candidates for complete surgical resection. Patients are randomized 1:1 to receive nivolumab 240 mg intravenously every 2 weeks for 2 doses with or without oral tadalafil 10 mg once daily for 4 weeks. Surgery is performed at approximately 4 weeks from first nivolumab infusion. The primary endpoint is correlative analysis of immune cell polarization in peripheral blood and tumor specimens pre and post treatment. Secondary endpoints include additional immune profiles, exosomes, and tissue surgical wound healing and tumor response assessed by repeat imaging before surgery. The sample size is 25 patients per arm (50 total). Enrollment began in September 2017 and 25 patients have been enrolled over 10 months.

**Acknowledgements**

Sidney Kimmel Cancer Center at Thomas Jefferson UniversityBristol-Myers Squibb

**Trial Registration**

NCT03238365

**Ethics Approval**

The study was approved by Thomas Jefferson University Institutution‘s Ethics Board, approval number #17P.210

#### P329 Phase 3 KEYNOTE-716 study: Adjuvant therapy with pembrolizumab versus placebo in resected high-risk stage II melanoma

##### Jason Luke, MD, FACP^1^, Paolo Ascierto, MD^2^, Matteo Carlino, MBBS, PhD, BMedSC, F^3^, Alexander Eggermont^4^, Jean-Jacques Grob, MD^5^, Axel Hauschild^6^, John Kirkwood, MD^7^, Georgina Long, MBBS, PhD, BSc, FRAC^8^, Peter Mohr^9^, Caroline Robert^4^, Jeffrey Gershenwald, MD^10^, Andrew Poklepovic, MD^11^, Merrick Ross, MD^10^, Richard Scolyer^12^, James Anderson^13^, Sama Ahsan^13^, Nageatte Ibrahim, MD PhD^13^, Vernon Sondak^14^

###### ^1^University of Chicago, Chicago, MA, USA; ^2^Istituto Nazionale Tumori IRCCS “Fondazi, Napoli, Italy; ^3^Westmead Hospital, Blacktown, Australia; ^4^Gustave Roussy Cancer Centre, Villejuif, France; ^5^Aix-Marseille Université, Marseille, France; ^6^University Hospital Schleswig-Holstein, Kiel, Germany; ^7^UPMC Hillman Cancer Center, Pittsburgh, PA, USA; ^8^The University of Sydney, Sydney, Australia; ^9^Elbe Kliniken Buxtehude, Germany; ^10^MD Anderson Cancer Center, Houston, TX, USA; ^11^VCU Massey Cancer Center, Richmond, VA, USA; ^12^Royal Prince Alfred Hospital, Sydeney, Australia; ^13^Merck & Co., Inc., Kenilworth, NJ, USA; ^14^Moffit Cancer Center, Tampa, FL, USA

####### **Correspondence:** Jason Luke (lukehorse@hotmail.com)

**Background**

Adjuvant pembrolizumab showed significantly longer recurrence-free survival compared with placebo in resected stage III melanoma in the KEYNOTE-054 study [1]. KEYNOTE-716 (NCT03553836) is a randomized, placebo- controlled, multicenter phase 3 study of adjuvant pembrolizumab in patients with surgically resected high-risk stage II melanoma.

**Methods**

Patients must be ≥12 years of age and have newly diagnosed, completely resected stage IIB/IIC cutaneous melanoma, defined by the AJCC Cancer Staging Manual, 8th edition [2] (including negative sentinel lymph node biopsy and no evidence of distant metastasis). Patients cannot have mucosal or uveal melanoma or have received prior treatment for melanoma, including radiation, beyond resection of primary disease within 12 weeks of the start of study therapy. The study has a 2-part design. In the double-blind phase (part 1), patients will be randomly assigned 1:1 to receive pembrolizumab 200 mg for patients ≥18 years or 2 mg/kg for patients 12-17 years (maximum dose, 200 mg) or placebo every 3 weeks for 17 cycles. Stratification: 1 stratum for pediatric patients (12-17 years); 3 strata for adult patients per T stage (T3b/T4a/T4b). Study treatment will begin within 12 weeks of complete resection. Tumor imaging will be performed every 24 weeks while treatment is ongoing, at the end of treatment, every 6 months for the first 3 years off treatment, and then yearly for up to 2 years or until recurrence (up to 5 years of total imaging). Adverse events will be graded per NCI Common Terminology Criteria for Adverse Events, version 4.0. In the unblinded phase (part 2), patients with confirmed recurrence may be rechallenged (patients received pembrolizumab in part 1) or crossed over to pembrolizumab (patients received placebo in part 1). Resected local or distant recurrence or unresectable disease will be treated for an additional 17 or 35 cycles, respectively. Tumor imaging in part 2 will occur every 12 weeks while treatment is ongoing. The primary end point is recurrence-free survival; secondary end points are distant metastasis-free survival, overall survival, and safety. Approximately 954 patients will be enrolled.

**Trial Registration**

ClinicalTrials.gov identifier, NCT03553836

**References**

1. Eggermont AMM, et al. N Engl J Med. Adjuvant pembrolizumab versus placebo in resected stage III melanoma. 2018;378:1789-801.2. Amin MB et al., eds. AJCC Cancer Staging Manual, 8th edition. New York: Springer International Publishing; 2017.

**Ethics Approval**

The study was approved by the relevant Institutional Review Boards for each institution.

#### P330 Harnessing the power of lymphodepletion and checkpoint blockade: A Nivolumab/Ipilimumab-primed immunotransplant for relapsed/refractory diffuse large B cell lymphoma patients

##### Thomas Marron, MD PhD^1,2^, Netonia Marshall, PhD^2^, Dana Ostrowski, BA^2^, Amir Steinberg, MD^2^, Linda Hammerich, PhD^2^, Sacha Gnjatic, PhD^2^, Nina Bhardwaj, MD, PhD^2^, Joshua Brody, MD^2^, Thomas Marron, MD PhD^2^

###### ^1^Tisch Cancer Institute, The Mount Sinai Hospital, New York, NY, USA; ^2^Icahn School of Medicine at Mount Sinai, New York, NY, USA

####### **Correspondence:** Thomas Marron (thomas.marron@mountsinai.org)

**Background**

Patients with relapsed/refractory diffuse large B-cell lymphoma (DLBCL) have a poor prognosis, with only a quarter of patients achieving a CR following autologous stem cell transplant (ASCT)(1). Novel immunotherapies offer promise, however, checkpoint blockade achieves a very low complete response rate(2), and chimeric antigen receptor T cells (CAR-T) achieve durable complete remissions in fewer than a third of patients, often due to exhaustion or antigen loss(3,4). Cellular therapies like CAR-T utilize lymphodepleting chemotherapy to achieve rapid proliferation and anti-tumor activity of cytotoxic T cells infused after chemotherapy(5-7), and we have documented similar homeostatic proliferation of T cells following ASCT, however, these cells develop an exhausted phenotype. We therefore hypothesized that combined checkpoint blockade and lymphodepletion may result in synergistic clinical efficacy and provide the added benefit of a poly-clonal/multiantigen-specific T cell response. In the A20 lymphoma murine model we treated mice growing tumors with dual checkpoint blockade (DCBA, anti-PD-1 and anti-CTLA-4)—priming polyclonal anti-tumor T cells in vivo—and then transferred their cells into tumor bearing recipients following lymphodepletion(8). As in humans, following adoptive transfer into a lymphodepleted recipient CD8 T cells expand but also upregulate functional checkpoint receptors limiting their activity. The addition of DBCA before and after adoptive transfer into a lymphodepleted host—termed immunotransplant—increases anti- tumor response by increasing activation, cytokine production, and proliferation in a common gamma chain cytokine- dependent manner. Immunotransplant improved survival over either component given alone.

**Methods**

We have translated these murine findings into an immunotransplant trial in DLBCL patients with chemo-insensitive disease or disease relapsed following ASCT or CAR-T therapy. Patients will receive 2 cycles of DCBA (ipilimumab and nivolumab) given at three-week intervals, and subsequently undergo apheresis and cryopreservation of their PBMCs. Patients then receive lymphodepleting conditioning with fludarabine and cyclophosphamide, according to the dosing schedule used in the ZUMA-1 trial of KTE-C19 CAR-T cells[9]. Patients then are infused with their pre- chemotherapy cells, and upon count recovery patients will receive an additional two cycles of DCBA followed by maintenance nivolumab.The primary endpoint is clinical efficacy, defined as the proportion of patients who achieve complete response. Secondary endpoints include correlation of imaging with molecular remission (determined by blood IgVH), one and two-year overall survival and progression-free survival, overall response rate, as well as assessment for dynamic changes in the stool microbiome and correlates with outcome.

**Results**

This trial opened to accrual in May of 2018 (NCT0330544), enrollment is ongoing.

**Trial Registration**

NCT0330544

**References**

1. Vose JM, Zhang MJ, Rowlings PA, et al. Autologous transplantation for diffuse aggressive non-Hodgkin's lymphoma in patients never achieving remission: a report from the Autologous Blood and Marrow Transplant Registry. Journal of clinical oncology : official journal of the American Society of Clinical Oncology. 2001;19(2):406-413.

2. Lesokhin AM, Ansell SM, Armand P, et al. Nivolumab in Patients With Relapsed or Refractory Hematologic Malignancy: Preliminary Results of a Phase Ib Study. Journal of clinical oncology : official journal of the American Society of Clinical Oncology. 2016;34(23):2698-2704.

3. Schuster SJ, Bishop MR, Tam CS, et al. Primary analysis of Juliet: a global, pivotal, phase 2 trial of CTL019 in adult patients with relapsed or refractory diffuse large B-cell lymphoma. Am Soc Hematology; 2017.

4. Turtle CJ, Hay KA, Hanafi L-A, et al. Durable Molecular Remissions in Chronic Lymphocytic Leukemia Treated With CD19-Specific Chimeric Antigen Receptor–Modified T Cells After Failure of Ibrutinib. Journal of Clinical Oncology. 2017;35(26):3010-3020.

5. Dudley ME, Wunderlich JR, Robbins PF, et al. Cancer regression and autoimmunity in patients after clonal repopulation with antitumor lymphocytes. Science. 2002;298(5594):850-854.

6. Gattinoni L, Finkelstein SE, Klebanoff CA, et al. Removal of homeostatic cytokine sinks by lymphodepletion enhances the efficacy of adoptively transferred tumor-specific CD8+ T cells. J Exp Med. 2005;202(7):907-912.

7. Kochenderfer JN, Dudley ME, Kassim SH, et al. Chemotherapy-refractory diffuse large B-cell lymphoma and indolent B-cell malignancies can be effectively treated with autologous T cells expressing an anti-CD19 chimeric antigen receptor. Journal of clinical oncology : official journal of the American Society of Clinical Oncology. 2015;33(6):540-549.

8. Marshall N, Hammerich L, Upadhyay R, Marron TU, Brody J. Converting a tumor from checkpoint blockade- resistant to checkpoint blockade-sensitive: Immunotransplant for aggressive lymphoma. Journal of Clinical Oncology. 2016;34(15_suppl):e14538-e14538.

9. Locke FL, Neelapu SS, Bartlett NL, et al. CT019 - Primary results from ZUMA-1: a pivotal trial of axicabtagene ciloleucel (axicel; KTE-C19) in patients with refractory aggressive non-Hodgkin lymphoma (NHL) AACR; April 2, 2017, 2017; Washington D.C.

**Ethics Approval**

The protocol for this clinical trial has been approved by the Icahn School of Medicine Institutional Review Board (IRB, GCO#17-2164) and FDA. This trial is being performed under the supervision of the Mount Sinai IRB and in accordance with principles of the Declaration of Helsinki.

**Fig. 1 (abstract P330).**
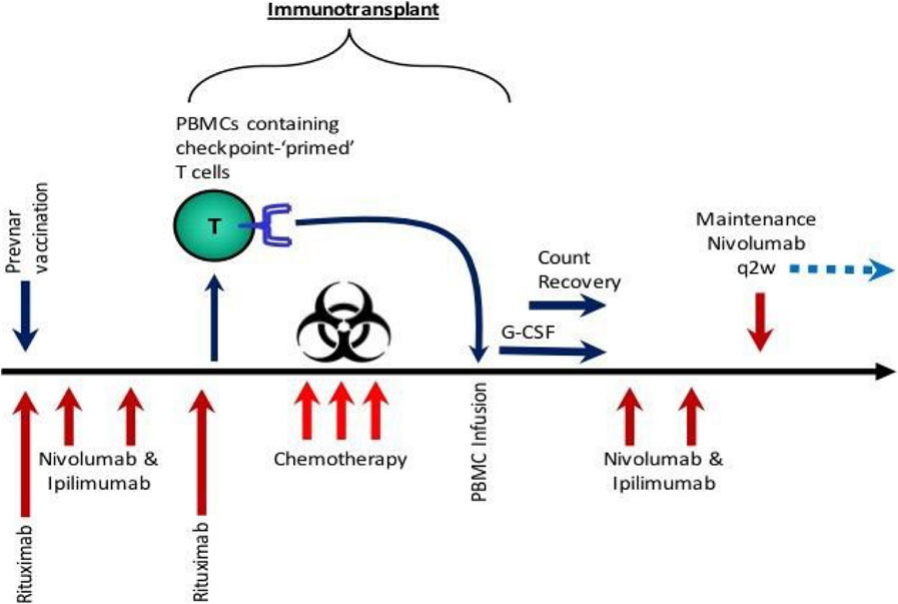
Trial Schematic

#### P331 A phase 1a clinical trial of NLG802, a prodrug of indoximod with enhanced pharmacokinetic properties

##### Olivier Rixe, MD, PhD^1^, Thomas George, MD^2^, Heloisa Soares^1^, Agnieszka Marcinowicz^3^, Nicholas Vahanian, MD^3^, Charles Link, MD^3^, Edouard Dupis^1^, Eugene Kennedy, MD, FACS^3^, Mario Mautino, PhD^3^

###### ^1^University of New Mexico, Albuquerque, NM, USA; ^2^Univ of Florida Health Cancer Center, Gainesville, FL, USA; ^3^NewLink Genetics, Ames, IA, USA

####### **Correspondence:** Eugene Kennedy (gkennedy@linkp.com)

**Background**

Indoximod contributes to enhanced antitumor immunity by relieving IDO-mediated immunosuppression by mechanisms that involve modulation of AhR signaling and mTOR activation, which leads to multiple immunomodulatory effects including a shift from suppressive Foxp3+ Treg toward Th17 helper T cells as well as the downregulation of IDO expression in DCs. Indoximod has demonstrated an excellent safety profile in human clinical trials at doses of up to 1200 mg bid. Increasing doses above this level generally does not result in increased plasma concentration or drug exposure due to limiting dose-dependent oral bioavailability. In order to improve bioavailability of indoximod we developed NLG802, a prodrug of indoximod that increases oral bioavailability of indoximod ~ 5-fold in non-human primates.

**Methods**

NLG802 is being evaluated in a standard 3+3 dose escalation Phase 1a study in patients with recurrent advanced solid malignancies. Doses being evaluated include 180, 360, 720, 1080 and 1440 mg bid. NLG802 is administered orally in repeating 28 day cycles until toxicity or disease progression. Endpoints include safety, toxicity, pharmacokinetics, and determination of an MTD or MBED.

**Results**

Single oral dosing of NLG802 at 180 mg (n=3) and 363 mg (n=4) resulted in indoximod Cmax of 7.2±2.7 μM and 11.8±1.6 μM and AUC(0-inf) 49±8 and 92±25 μMh, respectively. Multiple oral dosing of NLG802 at 180 mg bid (n=3) and 363 mg bid (n=4) resulted in indoximod Cmax of 6.5±2.8 μM and 18.5±3.1 μM and AUC(0-inf) 52±22 and 125±24 μMh, respectively. Among NLG802 treated patients the most common adverse events, regardless of attribution, are fatigue (50%), nausea (40%), and peripheral edema (40%). Serious adverse events include constipation, weakness, and esophagitis all attributed to underlying disease. The most common NLG802 related adverse events are fatigue (20%) and nausea (20%). No subjects have experienced a Grade 4/5 adverse event. No subjects discontinued therapy due to an adverse event.

**Conclusions**

Oral doses of NLG802, as compared to a molar equivalent oral dose of indoximod, produce an average increase of 4-fold in Cmax and AUC after a single dose and 5-6 fold increase in AUC and Cmax after continuous bid dosing.

Additional data on other dose cohorts will be available at the time of the presentation.

**Ethics Approval**

The study was approved by Western Institutional Review Board (WIRB) on 28May2017, WIRB PRO NUM 2017011

#### P332 CheckMate 9TM: Phase 3 study of nivolumab + cisplatin + radiotherapy in cisplatin-eligible patients with intermediate- or high-risk locally advanced squamous cell carcinoma of the head & neck

##### Maura Gillison, MD, PhD^1^, Vincent Gregoire, MD, PhD^2^, Makoto Tahara^3^, Quynh Thu Le, MD^4^, Wilfried Budach^5^, Mark Lynch, PhD^6^, Justin Kopit^6^, Vijayvel Jayaprakash, MBBS, PhD^6^, Peng Sun^6^, Robert Ferris, MD, PhD^7^

###### ^1^MD Anderson Cancer Center, Houston, TX, USA; ^2^Centre Léon Bérard, Brussels, Belgium; ^3^National Cancer Center, Kashiwa, Japan; ^4^Stanford University, Stanford, CA, USA; ^5^University of Düsseldorf, Düsseldorf, Germany; ^6^Bristol-Myers Squibb, Wallingford, CT, USA; ^7^University of Pittsburgh Cancer Institute, Pittsburgh, PA, USA

####### **Correspondence:** Maura Gillison (mgillison@mdanderson.org)

**Background**

Squamous cell carcinoma of the head and neck (SCCHN) is the most common pathological type of head and neck cancer [1]. Approximately 60% of patients present with locally advanced disease [2], for which cisplatin-based chemoradiotherapy is the standard of care [3]. However, for patients with intermediate- and high-risk SCCHN, outcomes remain poor [4]. In preclinical models, radiotherapy in combination with or preceded by administration of an immune checkpoint inhibitor enhances antitumor immune responses in addition to having a direct cytotoxic effect [5]. Nivolumab is an anti–PD-1 antibody that has shown improved survival in patients with recurrent/metastatic SCCHN post-platinum therapy [6]. CheckMate 9TM (NCT03349710) is an ongoing, randomized, double-blind, placebo-controlled, phase 3 trial investigating nivolumab-based treatment in intermediate- or high-risk locally advanced SCCHN. The study is enrolling patients in 2 cohorts: cisplatin-eligible and cisplatin-ineligible; here, we discuss the cisplatin-eligible cohort of the study.

**Methods**

The cisplatin-eligible cohort of CheckMate 9TM includes patients who are considered eligible for cisplatin-based chemoradiation due to having creatinine clearance ≥60 mL/min. Eligibility for enrollment in CheckMate 9TM includes: histologically confirmed SCCHN of the oral cavity, oropharynx, hypopharynx, or larynx with locally advanced disease that is unresectable or resectable but suitable for an organ-sparing approach, no prior radiotherapy or systemic treatment, and Eastern Cooperative Oncology Group status of 0-1. Patients eligible for inclusion in this trial could have tumors with either intermediate or high risk of recurrence based on baseline factors including human papillomavirus p16 status, tumor staging and smoking history. Approximately 580 patients will be enrolled and randomized 1:1 to treatment with either nivolumab plus cisplatin in combination with radiotherapy or corresponding placebo plus cisplatin in combination with radiotherapy (Figure 1). Treatment will continue until progression or completion of maintenance treatment. Primary endpoint: event-free survival. Secondary endpoints: duration of loco- regional control, overall survival, and proportion of patients without meaningful symptom deterioration at 24 weeks after treatment completion. Enrollment is ongoing in 11 countries with an estimated study completion date of November 2022.

**Acknowledgements**

The patients and families for making this trial possible; the contributions of the study teams from the various sites involved in the trial; the protocol manager for this study, Anne Delvaux; Bristol-Myers Squibb (Princeton, NJ) and ONO Pharmaceutical Company Ltd. (Osaka, Japan); the study was supported by Bristol-Myers Squibb; all authors contributed to and approved the submission; writing and editorial assistance was provided by Brooke Middlebrook, Evidence Scientific Solutions Inc., funded by Bristol-Myers Squibb.

**Trial Registration**

Clinicaltrials.govNCT03349710

**References**

1. Ferlay J, Soerjomataram I, Dikshit R, et al. Cancer incidence and mortality worldwide: sources, methods and major patterns in GLOBOCAN 2012. Int J Cancer. 2015;136:E359-386.

2. SEER Cancer Statistics Review (CSR) 1975-2014.

3. Georges P, Rajagopalan K, Leon C, et al. Chemotherapy advances in locally advanced head and neck cancer. World J Clin Oncol. 2014;5:966-972.

4. Szturz P, Vermorken JB. Treatment of elderly patients with squamous cell carcinoma of the head and neck. Front Oncol. 2016;6:199.

5. Teng F, Kong L, Meng X, Yang J, Yu J. Radiotherapy combined with immune checkpoint blockade immunotherapy: Achievements and challenges. Cancer Lett. 2015;365:23-29.

6. Ferris RL, Blumenschein G, Jr., Fayette J, et al. Nivolumab for recurrent squamous-cell carcinoma of the head and neck. N Engl J Med. 2016;375:1856-1867.

**Ethics Approval**

This study was approved by each institution’s Review Board/Independent Ethics Committee.

**Fig. 1 (abstract P332).**
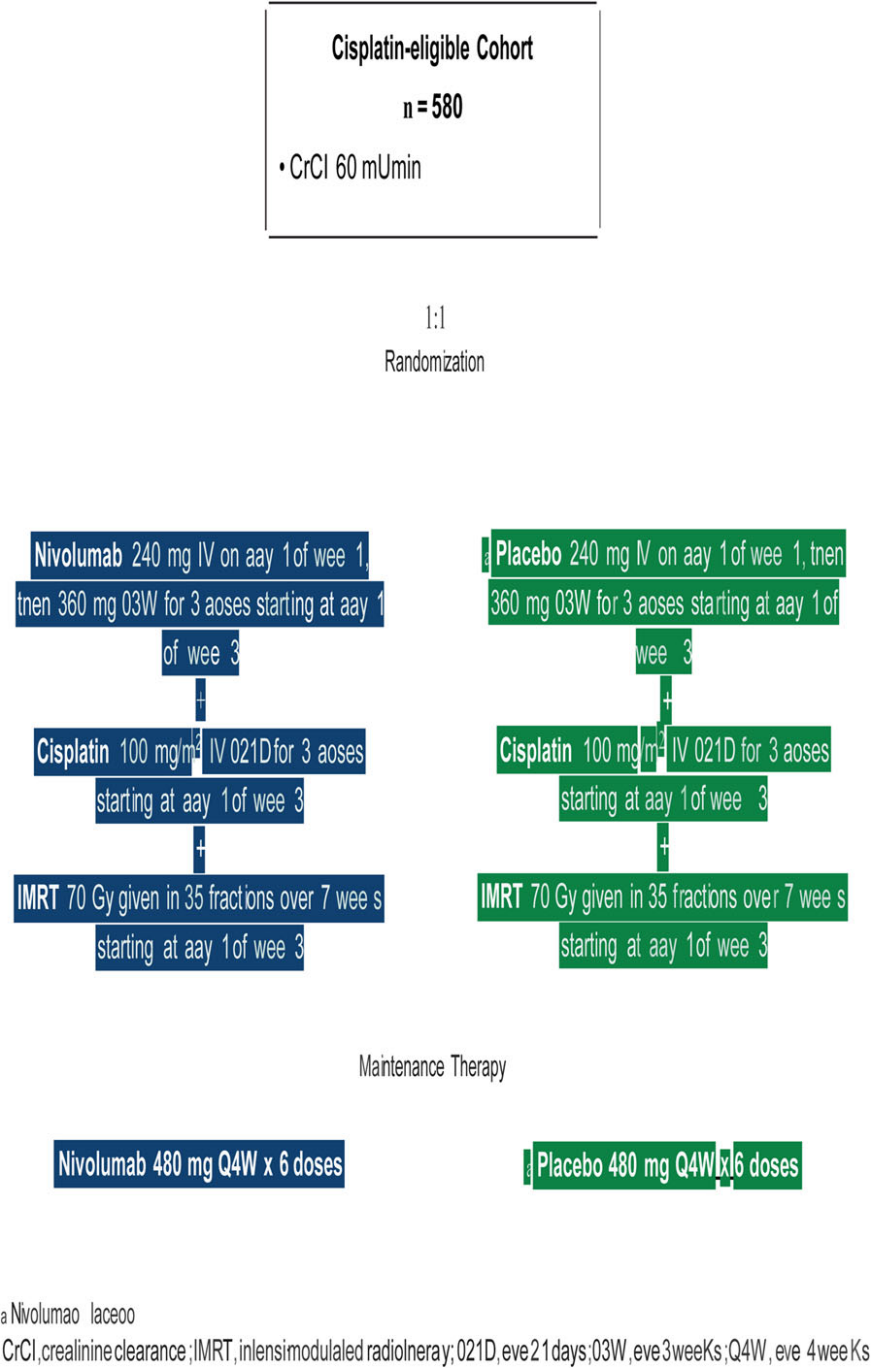
See text for description.

#### P333 CheckMate 9TM: Phase 3 study of nivolumab + radiotherapy (RT) vs cetuximab + RT in cisplatin-ineligible patients with intermediate-/high-risk locally advanced squamous cell carcinoma of the head/neck

##### Robert haddad, MD^1^, Kevin Harrington, MD^2^, Lisa Licitra, MD^3^, Peter Brossart^4^, Dennis Soulières^5^, Loren Mell, MD^6^, Laurence Toms^7^, Justin Kopit^7^, Mark Lynch, PhD^7^, Vijayvel Jayaprakash, MBBS, PhD^7^, Ezra Cohen, MD^8^

###### ^1^Dana-Farber Cancer Institute, BOSTON, MA, USA; ^2^Royal Marsden NHS Foundation Trust/The Institute of Cancer Research, UK; ^3^Fondazione IRCCS Istituto Nazionale dei Tumori and University of Milan, Milan, Italy; ^4^University Hospital of Bonn, Bonn, Germany; ^5^Centre Hospitalier de l’Université de Montréal, Montreal, Canada; ^6^University of California San Diego School of Medicine, San Diego, CA, USA; ^7^Bristol-Myers Squibb, Princeton, NJ, USA; ^8^University of California San Diego, Moores Cancer Center, La Jolla, CA, USA

####### **Correspondence:** Robert haddad (robhaddad@msn.com)

**Background**

The standard of care for patients with locally advanced squamous cell carcinoma of the head and neck (SCCHN) is cisplatin-based radiotherapy [1]. Treatment options for patients ineligible to receive cisplatin due to advanced age or comorbidities [2] are limited to radiotherapy alone or in combination with cetuximab, and associated with modest efficacy or increased toxicity [3]. As the majority of patients with locally advanced SCCHN eventually develop recurrent/metastatic disease, there is an unmet need for better treatment options [4]. Nivolumab is an anti–PD-1 antibody that has shown improved survival and acceptable tolerability in patients with recurrent/metastatic SCCHN post-platinum therapy [5]. CheckMate 9TM (NCT03349710) is an ongoing, randomized, double-blind, placebo- controlled, phase 3 trial investigating nivolumab-based treatment in intermediate- or high-risk locally advanced SCCHN. The study is enrolling patients in 2 cohorts: cisplatin-eligible and cisplatin-ineligible; here, we discuss the cisplatin-ineligible cohort of the study.

**Methods**

The cisplatin-ineligible cohort of CheckMate 9TM includes patients who are considered ineligible for cisplatin-based chemoradiation due to the presence of ≥1 of the following: age ≥70 years, creatinine clearance <60 and >30 mL/min, or severe hearing loss (minimal hearing threshold ≥80 dB in either ear). Eligibility for enrollment in CheckMate 9TM includes: histologically confirmed SCCHN of the oral cavity, oropharynx, hypopharynx, or larynx with locally advanced disease that is unresectable or resectable but suitable for an organ-sparing approach, no prior radiotherapy or systemic treatment, and Eastern Cooperative Oncology Group status of 0-1. Patients eligible for inclusion in this trial could have tumors with either intermediate or high risk of recurrence based on baseline factors including human papillomavirus p16 status, tumor staging and smoking history. Approximately 466 patients will be enrolled and randomized 1:1 to either nivolumab plus radiotherapy or cetuximab plus radiotherapy (Figure 1). Patients in each treatment arm will also be administered the corresponding placebo. Treatment will continue until progression or completion of maintenance treatment. Primary endpoint: event-free survival. Secondary endpoints: duration of loco-regional control, overall survival, and proportion of patients without meaningful symptom deterioration at 24 weeks after treatment completion. Enrollment is ongoing in 11 countries with an estimated study completion date of November 2022.

**Acknowledgements**

The patients and families for making this trial possible; the contributions of the study teams from the various sites involved in the trial; the protocol manager for this study, Anne Delvaux; Bristol-Myers Squibb (Princeton, NJ) and ONO Pharmaceutical Company Ltd. (Osaka, Japan); the study was supported by Bristol-Myers Squibb; all authors contributed to and approved the submission; writing and editorial assistance was provided by Brooke Middlebrook, Evidence Scientific Solutions Inc., funded by Bristol-Myers Squibb.

**Trial Registration**

Clinicaltrials.govNCT03349710

**References**

1. Georges P, Rajagopalan K, Leon C, et al. Chemotherapy advances in locally advanced head and neck cancer. World J Clin Oncol. 2014;5:966-972.

2. Ahn MJ, D'Cruz A, Vermorken JB, et al. Clinical recommendations for defining platinum unsuitable head and neck cancer patient populations on chemoradiotherapy: A literature review. Oral oncology. 2016;53:10-16.

3. Baxi SS, O'Neill C, Sherman EJ, et al. Trends in chemoradiation use in elderly patients with head and neck cancer: Changing treatment patterns with cetuximab. Head Neck. 2016;38 Suppl 1:E165-171.

4. Szturz P, Vermorken JB. Treatment of elderly patients with squamous cell carcinoma of the head and neck. Front Oncol. 2016;6:199.

5. Ferris RL, Blumenschein G, Jr., Fayette J, et al. Nivolumab for recurrent squamous-cell carcinoma of the head and neck. N Engl J Med. 2016;375:1856-1867.

**Ethics Approval**

This study was approved by each institution’s Review Board/Independent Ethics Committee.

**Fig. 1 (abstract P333).**
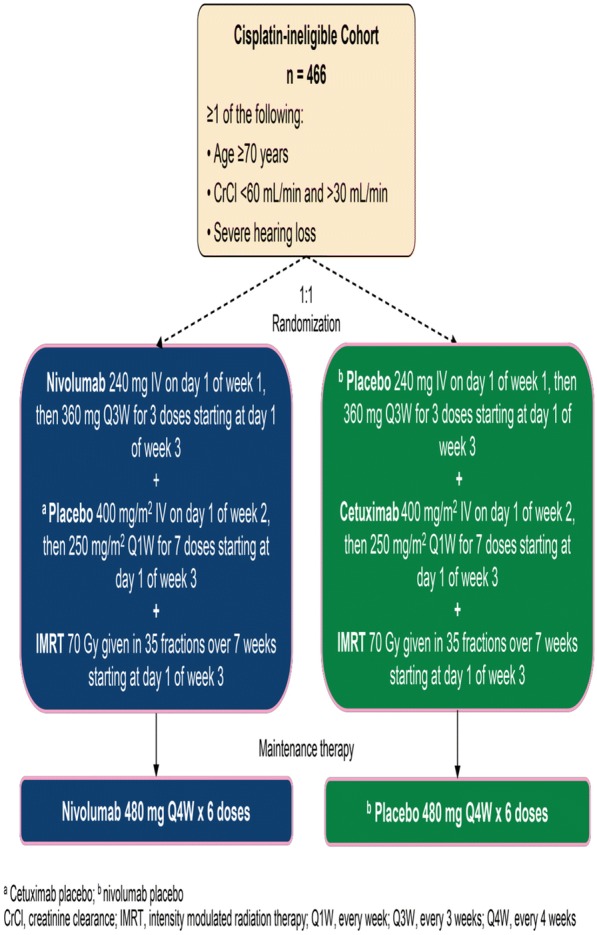
See text for description.

#### P334 AI-designed dual peptide vaccine plus novel combination adjuvant showed markedly induction of antigen- specific CTLs with disease stabilization in last line patients with GI cancers

##### Masao Nakajima, MD^1^, Shoichi Hazama^2^, Koji Tamada, MD PhD^3^, Keiko Udaka^4^, Yasunari Kouki^5^, Toshinari Uematsu^5^, Hideki Arima^5^, Shun Doi, PhD^6^, Hiroto Matsui^1^, Sinsuke Kanekiyo^1^, Yoshitaro Shindo^1^, Yukio Tokumitsu^1^, Shinobu Tomochika^1^, Shin Yoshida^1^, Michihisa Iida^1^, Nobuaki Suzuki^1^, Shigeru Takeda^1^, Shigeru Yamamoto^1^, Shigefumi Yoshino^5^, Tomio Ueno^7^, Hiroaki Nagano^1^

###### ^1^Yamaguchi Univ. Graduate school of medicine, Ube, Japan; ^2^Yamaguchi Univ. School of Medicine, Ube, Japan; ^3^Yamaguchi University, Ube, Japan; ^4^Kochi Medical School, Kochi, Japan; ^5^Yamaguchi University hospital, Ube, Japan; ^6^CYTLIMIC Inc, Tokyo, Japan; ^7^Kawasaki University School of Medicine, Kurashiki, Japan

####### **Correspondence:** Masao Nakajima; Shoichi Hazama (hazama@yamaguchi-u.ac.jp)

**Background**

Based on the exploratory analysis of our previous studies of peptide vaccine, we concluded that the combination of adjuvants hLAG-3Ig + Poly-ICLC was essential to overcome the therapeutic limitation of the traditional peptide studies by controlling negative immune checkpoints and enhancing CTLs induction. Another issue with peptide vaccines is human leukocyte antigen (HLA) restriction. Hence, we developed novel multi-HLA-binding peptides derived from the tumor antigens, HSP70 and GPC3, and confirmed the high expression in many types of cancer. After the presentation at ASCO 2017, we expanded pts in recommended dose to confirm safety and explored biomarkers for efficacy.

**Methods**

We identified HSP70- and GPC3-peptide that have high binding affinity to each of HLA-A2402, 0201, and 0206 by a binding prediction system (NEC Corporation). In this phase I study of a novel peptide cancer vaccine therapy for patients with metastatic solid cancer, the primary objective was to evaluate its safety and toxicity. Secondary objective was to examine the immune and clinical response, and also to confirm the recommendable dose. This study used a three-tiered dose-escalation strategy with 3 pts’ cohorts. In addition to the 3 scheduled cases, 8 more cases were added and 11 cases were enrolled at the recommended dose. Moreover, to explore biomarkers for efficacy, we analyzed the exhaustion markers on T cells and immune-suppressive cells in PBMC of the treated patients.

**Results**

Seventeen HLA-A*24:02-, 02:01-, and 02:06-matched pts (esophagus (EC), 5; colon (CRC), 6; liver (HCC), 4; pancreas, 1; stomach, 1) were treated in this study. No severe adverse effects related to the treatment were encountered. Peptide-specific CTL induction to HSP70 and GPC3 was observed in 15 and 16 pts, respectively. We observed decreased tumor marker expression in 10 cases, and disease control was observed in 5 pts (18, 4, 3, 3, 2 M). OS rates in CRC, HCC, and EC were 80%, 100%, and 60% at 6M, and 60%, 75% and 0% at 12M, respectively. Low expression of PD1 on CD4+ T cells (p = 0.02) and low proportion of Treg Fraction II (p = 0.03) in the PBMC were the significant favorable biomarker for OS.

**Conclusions**

The combination cancer vaccine therapy using multi-HLA-restricted peptides and hLAG-3Ig + Poly-ICLC was safe and effective for treating the last line patients with metastatic solid tumors. The proportion of the PD-1+/CD4+ T cells and Treg Fraction II were the significant biomarker for OS.

**Ethics Approval**

The study was approved by Yamaguchi University‘s Ethics Board, approval number 12345. (UMIN ID: 000020440)

**Consent**

Written informed consent was obtained from the patient for publication of this abstract and any accompanying images. A copy of the written consent is available for review by the Editor of this journal.

#### P335 A phase 1 study of ALX148: CD47 blockade in combination with anticancer antibodies to bridge innate and adaptive immune responses for advanced malignancy

##### Nehal Lakhani, MD, PhD^1^, Patricia LoRusso, DO^2^, Laura Chow, MD^3^, Yung-Jue Bang, MD PhD^4^, Philip Fanning^5^, Yonggang Zhao, PhD, MBA^5^, Jaume Pons^5^, Hong Wan^5^, Sophia Randolph, MD, PhD^5^, Wells Messersmith, MD^6^

###### ^1^START Midwest, Grand Rapids, MI, USA; ^2^Yale Cancer Center, New Haven, CT, USA; ^3^Seattle Cancer Care Alliance, Seattle, WA, USA; ^4^Seoul National University Hospital, Seoul, Korea; ^5^ALX Oncology Inc., Burlingame, CA, USA; ^6^University of Colorado Cancer Center, Aurora, CO, USA

####### **Correspondence:** Nehal Lakhani; Sophia Randolph (srandolph@alxoncology.com)

**Background**

Tumors upregulate CD47, a marker of self, to evade the anticancer immune response. Blocking CD47 disrupts a key myeloid immune checkpoint and enhances innate and adaptive immunity against cancer[1]. ALX148 is a fusion protein comprising a high affinity CD47 blocker with an inactive human Fc domain to extend serum half-life and minimize toxicity. ALX148 safely enhances activity of anticancer targeted antibodies and checkpoint inhibitors (CPI) in nonclinical models[1]. Single agent ALX148 is well tolerated by patients with no maximum-tolerated dose (MTD) reached, a maximum-administered dose (MAD) of 30 mg/kg every other week, and no dose-dependent hematologic toxicity (SITC 2017, #241; ASCO 2018, #3068), negating the need for priming/loading administration strategies. Part 2 of this study evaluates the safety of ALX148 in combination with pembrolizumab, trastuzumab or rituximab in patients with advanced malignancy.

**Methods**

The primary objective of Part 2 is to characterize the safety profile of ALX148 administered with established anti-cancer antibodies. Patient cohorts with solid tumor malignancy receive escalating doses of intravenous ALX148 in combination with pembrolizumab or trastuzumab (HER2-positive tumors). Patients are also evaluated for response, PK, and CD47 target occupancy (TO). Preliminary combination data from the fully enrolled solid tumor dose escalation and ongoing expansion cohorts are reported as of July 20, 2018 and will be updated at the time of presentation.

**Results**

Twenty-five patients with advanced solid tumors received ALX148 in combination with pembrolizumab or trastuzumab as of data cutoff. Patients were heavily pretreated with a median of 5 (2-12) prior regimens. Median age was 56 (32-74) yrs. Thirteen patients (52%) experienced predominantly single and low grade treatment-related adverse events (TRAEs). No ALX148 MTD was reached with a MAD of 10 mg/kg, once weekly. Of the initial 8 evaluable patients in the pembrolizumab cohort, there was 1PR with 48% tumor reduction at first evaluation (NSCLC, PD-L1 <10%, CPI-refractory), 2SD (NSCLC, PD-L1 0%, > 24 weeks; appendiceal cancer, MSS). Of the initial 8 evaluable patients in the trastuzumab cohort, there were 3SD (1-gastroesophageal junction with 27% tumor reduction at first evaluation, trastuzumab-resistant; 2-breast). Initial PK and CD47 TO of ALX148 in combination were similar to that of single agent administration.

**Conclusions**

ALX148 is well tolerated with preliminary antitumor activity in combination with pembrolizumab or trastuzumab in patients with advanced solid tumors. No MTD was reached in either combination. Combination expansion cohorts are ongoing, with ALX148 administered 10 mg/kg, once weekly.

**Trial Registration**

ClinicalTrials.gov identifier NCT03013218.

**References**

1. Kauder S, et al., ALX148 blocks CD47 and enhances innate and adaptive antitumor immunity with a favorable safety profile. PLOS ONE. In Press.

#### P336 An international, single-arm, phase 2 study of INCMGA00012 in patients with advanced squamous carcinoma of the anal canal (SCAC) who have progressed following platinum-based chemotherapy (NCT03597295)

##### Sheela Rao^1^, May Cho^2^, Anne Demols^3^, Talal Kayyal^4^, Hiral Parekh^5^, Gerard Kennealey, MD^6^, Chuan Tian^6^, Melissa Catlett^6^, Marwan Fakih, MD^7^

###### ^1^The Royal Marsden, London, UK; ^2^UC Davis Comprehensive Cancer Center, Sacramento, CA, USA; ^3^CUB Hôpital ERASME, Brussels, Belgium; ^4^Renovatio Clinical, Houston, TX, USA; ^5^University of Florida, Gainesville, FL, USA; ^6^Incyte Corporation, Wilmington, DE, USA; ^7^City of Hope Comprehensive Cancer Cente, Duarte, CA, USA

####### **Correspondence:** Sheela Rao (Sheela.Rao@rmh.nhs.uk)

**Background**

A standard treatment for refractory advanced SCAC has not yet been established; however, preliminary phase 2 results with PD-1 inhibitors showed promising activity [1,2]. INCMGA00012 is a humanized IgG4 monoclonal antibody that recognizes human PD-1 and has demonstrated acceptable tolerability with evidence of clinical activity in a phase 1 study of solid tumors (NCT03059823) [3]. This trial will evaluate the safety and efficacy of INCMGA00012 in patients with locally advanced or metastatic SCAC, including those with well-controlled human immunodeficiency virus (HIV) infection.

**Methods**

INCMGA 0012-202 is a phase 2, open-label, single-arm study, with planned enrollment of 81 patients who have locally advanced or metastatic SCAC following ≤2 prior systemic treatments, at least 1 of which included platinum. HIV+ patients should be well controlled on highly active antiretroviral therapy (HAART). Other key eligibility criteria include age ≥18 years, measurable disease by RECIST v1.1, and Eastern Cooperative Oncology Group performance status of 0 to 1. Daily prednisone dose of ≤10 mg or equivalent is allowed. Patients will receive INCMGA00012 500 mg by intravenous infusion once every 28 days up to 2 years in the absence of clinical disease progression or intolerable toxicity, or discontinuation for any other reason. Disease assessments will be performed every 8 weeks. The primary endpoint is overall response rate. Secondary endpoints include duration of response, disease control rate, progression-free survival, overall survival, safety, and pharmacokinetics. Health-related quality of life and relevant biomarkers are exploratory endpoints.

**Acknowledgements**

Clinical trial management was provided by Tristan Richard (Incyte Corporation, Wilmington, DE).

**Trial Registration**

NCT03597295

**References**

1. Morris VK, Salem ME, Nimeiri H, et al. Nivolumab for previously treated unresectable metastatic anal cancer (NCI9673): a multicentre, single-arm, phase 2 study. Lancet Oncol. 2017; 18:446-453.

2. Ott PA, Piha-Paul SA, Munster P, et al. Safety and antitumor activity of the anti-PD-1 antibody pembrolizumab in patients with recurrent carcinoma of the anal canal. Ann Oncol. 2017; 28:1036-1041.

3. Lakhani N, Mehnert J, Rasco D, et al. A phase 1 study of the safety, tolerability, and pharmacokinetics (PK) of MGA012 (anti-PD-1 antibody) in patients with advanced solid tumors. J Immunother Cancer. 2017; 5(Suppl 2):Abstract P249.

**Ethics Approval**

The study was approved by institutional review boards or independent ethics committees of participating institutions.

#### P337 Phase I dose-escalation and expansion study of intratumoral CV8102, a RNA-based TLR- and RIG-1 agonist with or without anti-PD1antibodies in patients with advanced solid tumors

##### Lucie Heinzerling^1^, Juergen Krauss^2^, Carsten Weishaupt^3^, Patrick Terheyden^4^, Benjamin Weide, MD^5^, Ralf Gutzmer^6^, Peter Mohr^7^, Juergen Becker, MD, PhD^8^, Felix Kiecker^9^, Angelika Daehling^10^, Fatma Doener, PhD^10^, Regina Heidenreich^10^, Sarah-Katharina Kays^10^, Ute Klinkhardt, MD^10^, Birgit Scheel^10^, Oliver Schönborn- Kellenberger^11^, Tobias Seibel^10^, Tanja Strack^10^, Ulrike Gnad-Vogt, MD^10^, Madelaine Schroth^10^

###### ^1^University Hospital of Erlangen, Germany; ^2^Nationales Centrum für Tumorerkrankungen, Heidelberg, Germany; ^3^Universitaetsklinikum Muenster, Muenster, Germany; ^4^Universitätsklinikum Schleswig-Holstein, Lübeck, Germany; ^5^Universitäts-Hautklinik Tuebingen, Tuebingen, Germany; ^6^Haut-Tumor-Zentrum Hannover, Germany; ^7^Elbe Kliniken Stade-Buxtehude GmbH, Germany; ^8^Translational Skin Cancer Research, Essen, Germany; ^9^Charité Universitätsmdizin Berlin, Berlin, Germany; ^10^CureVac AG, Tuebingen, Germany; ^11^Cogitars GmbH, Heidelberg, Germany

####### **Correspondence:** Ulrike Gnad-Vogt (Ulrike.Gnad-Vogt@curevac.com)

**Background**

Intratumoral (IT) activation of innate immune signaling pathways is a promising approach to overcome the immunosuppressive tumor microenvironment andinduce/restore anti-tumor immunity. CV8102, a single-stranded non-coding RNA complexed with a cationic peptide that signals via TLR-7/-8 and RIG-I [2], has shown to stimulate transient upregulation of inflammatory cytokines, chemokines and IFN-γ related genes at the injection site, followed by activation of T, NK, NKT and migratory dendritic cells in the draining lymph nodes [1]. IT CV8102 demonstrated dose-dependent anti-tumor activity and synergized with systemic PD1 inhibition in preclinical models. This Phase I study investigates CV8102 as single agent and in combination with systemic anti-PD1 antibodies.

**Methods**

Patients (pts) with advanced inoperable melanoma (MEL), cutaneous/head and neck squamous cell or adenoid cystic carcinoma (cSCC, SCCHN, ACC) are eligible for single agent CV8102, MEL and SCCHN pts without response after 12 weeks of anti-PD1 treatment are eligible for the combination part.Pts are treated with up to 8 IT injections of CV8102 into a single tumor lesion over a 12 week period. Dose-escalation for single agent and anti-PD1 combination follows a Bayesian logistic regression model. Responses are assessed Q8W/Q12W per RECIST 1.1/irRECIST and. pre- and on-treatment samples collected for biomarker analyses.. After determination of the recommended dose expansion cohorts are planned.

**Results**

As of July 06, 2018, 9 pts (4 MEL, 2 SCCHN, 2 ACC, 1 cSCC) have been treated with single agent CV8102 (doses from 25 μg up to 150μg). 2 pts have been treated with CV8102 (25 and 50 μg) in combination with anti-PD1 antibodies. No dose limiting toxicities occurred, most common AEs were mild to moderate injection site reactions and flu-like symptoms.So far 6 pts are evaluable for tumor response after single agent CV8102 at their initial assessment. Notably, one MEL pt treated at the 150 μg dose experienced a complete regression of the injected as well as non-injected lesions. This patient also experienced a marked increase of IL-6 and CRP at 6 and 24 hours after the first injection, respectively. 4 pts achieved stable disease, including one SCCHN pt treated at 100 μg who experienced shrinkage of a non-injected lymph node metastasis. Dose escalation is continuing and updated safety and efficacy results will be presented.

**Conclusions**

CV8102 was so far well tolerated and early signs of clinical activity including regression of non-injected lesions were observed after single agent treatment.

**References**

1. Heidenreich R, Jasny E, Kowalczyk A, Lutz J, Probst J, Baumhof P, Scheel B, Voss S, Kallen KJ, Fotin-Mleczek M. A novel RNA-based adjuvant combines strong immunostimulatory capacities with a favorable safety profile. Int J Cancer. 2015 ;137:372-84.

2. Ziegler A, Soldner C, Lienenklaus S, Spanier J, Trittel S, Riese P, Kramps T, Weiss S, Heidenreich R, Jasny E, Guzmán CA, Kallen KJ, Fotin-Mleczek M, Kalinke U. A New RNA-Based Adjuvant Enhances Virus-Specific Vaccine Responses by Locally Triggering TLR- and RLH-Dependent Effects. J Immunol. 2017 ;198:1595-1605.

**Ethics Approval**

The study was approved by Universitätsklinikum Tübingen Medizinische Fakultät Ethik-Kommission, approval number 785/2016AMG1

#### P338 Phase II neoadjuvant and immunologic study using the anti B7-H3 antibody, Enoblituzumab, in patients with localized prostate cancer

##### Eugene Shenderov, MD, PhD^1^, Karim Boudadi, MD^1^, Angelo Demarzo, MD, PhD^1^, Mohamad Allaf, MD^1^, Onur Ertunc, MD^1^, Igor Vidal, MD^1^, Carolyn Chapman, AAS^1^, Hao Wang^1^, Jim Vasselli, MD^2^, Jon Wigginton, MD^2^, Jan Davidson-Moncada, MD, PhD^2^, Rehab Abdallah, MB BCh^1^, Tanya O'Neal, BS, MS^1^, Christian Pavlovich, MD^1^, Trinity Bivalacqua, MD PhD^1^, Ashley Ross, MD^1^, Charles Drake, MD, PhD^3^, Drew Pardoll, MD, PhD^1^, Emmanuel Antonarakis, MD^1^

###### ^1^Johns Hopkins Hospital, Baltimore, MD, USA; ^2^Macrogenics, Rockville, MD, USA; ^3^Columbia University, New York, NY, USA

####### **Correspondence:** Eugene Shenderov (Eugene.Shenderov@jhmi.edu)

**Background**

Prostate cancer (PCa) is the second-most-common cause of cancer-related deaths in men [1, 2]. Immune-checkpoint blockade has resulted in unprecedented treatment advances in multiple tumor types, despite yielding modest results in PCa. While CTLA-4 and PD-L1 are infrequently expressed in PCa, B7-H3 (another B7 superfamily member) is highly expressed in many PCas, may modulate anti-tumor immune responses, and is associated with worse prognosis [3-5]. Binding (blocking) B7-H3 is now clinically possible with the recent development of enoblituzumab (MacroGenics), a humanized Fc-optimized (for antibody-dependent cell-mediated cytotoxicity [ADCC]) monoclonal antibody that binds B7-H3 with high affinity and specificity [6, 7]. Here we describe a study to test the hypothesis that neoadjuvant enoblituzumab treatment in patients with high-risk localized PCa will lead to partial pathological responses and reduced biochemical recurrence following prostatectomy, initially by modulating T cell immunity in the tumor microenvironment (TME) and also through direct tumor killing via ADCC.

**Methods**

Thirty two (32) men with intermediate- and high-risk localized prostate cancer (Gleason sum 7-10) were consented on an IRB-approved single-center, single arm, phase 2 study evaluating the safety, anti-tumor effect, and immunogenicity of neoadjuvant enoblituzumab given prior to radical prostatectomy at Johns Hopkins. Participants receive enoblituzumab at a dose of 15 mg/kg IV given weekly for 6 doses prior to radical prostatectomy. Two weeks after the last dose of enoblituzumab, prostates are harvested at radical prostatectomy, and examined for secondary and correlative endpoints.

**Results**

To-date, the trial has enrolled 20/32 patients, with minimal clinical toxicity having been noted. We have optimized B7-H3 immunohistochemical (IHC) staining, showing specific B7-H3 staining (Figure 1) and the ability to stain for the CD8+ T cell infiltrate in enoblituzumab-treated prostatectomy samples (Figure 2). Furthermore, B7-H3 detection in post-treatment biopsies is consistent with preferential B7-H3 expression in tumor versus normal cells. Moreover, CD8+ T cell quantitation in the initial enoblituzumab-treated prostatectomy samples shows a statistically significant increase in infiltrate compared to age- and stage-matched untreated prostatectomy controls (Figure 3, median 98 vs 46 cells/mm2, P=0.003).

**Conclusions**

This study aims to explore the impact of B7-H3 blockade on PSA recurrence following prostatectomy, the effects on the prostate gland TME, and whether B7-H3 IHC staining can be used to predict response or resistance to B7-H3– targeted therapies. The described finding of enhanced CD8 infiltration suggests that enoblituzumab alters the TME in a fashion that results in enhanced CD8 T cell infiltration – a hallmark of responsiveness to immunotherapy.

**Acknowledgements**

Study sponsors: Emmanuel Antonarakis, M.D. (PI); MacroGenics, Inc., Rockville, MD; 2018 Conquer Cancer Foundation Young Investigator Award

**Trial Registration**

ClinicalTrials.Gov NCT02923180

**References**

1. Murphy SL, Xu J, Kochanek KD, Curtin SC, Arias E. Deaths: Final Data for 2015. National vital statistics reports: from the Centers for Disease Control and Prevention, National Center for Health Statistics, National Vital Statistics System. 2017;66(6):1-75.

2. Siegel RL, Miller KD, Jemal A. Cancer statistics, 2018. CA: A Cancer Journal for Clinicians. 2018;68(1):7-30.

3. Benzon B, Zhao SG, Haffner MC, Takhar M, Erho N, Yousefi K, Hurley P, Bishop JL, Tosoian J, Ghabili K, Alshalalfa M, Glavaris S, Simons BW, Tran P, Davicioni E, Karnes RJ, Boudadi K, Antonarakis ES, Schaeffer EM, Drake CG, Feng F, Ross AE. Correlation of B7-H3 with androgen receptor, immune pathways and poor outcome in prostate cancer: an expression-based analysis. Prostate Cancer Prostatic Dis. 2017;20(1):28-35.

4. Parker AS, Heckman MG, Sheinin Y, Wu KJ, Hilton TW, Diehl NN, Pisansky TM, Schild SE, Kwon ED, Buskirk SJ. Evaluation of B7-H3 Expression as a Biomarker of Biochemical Recurrence After Salvage Radiation Therapy for Recurrent Prostate Cancer. International Journal of Radiation Oncology Biology Physics. 2011;79(5):1343-9.

5. Roth TJ, Sheinin Y, Lohse CM, Kuntz SM, Frigola X, Inman BA, Krambeck AE, Mckenney ME, Karnes RJ, Blute ML, Cheville JC, Sebo TJ, Kwon ED. B7-H3 Ligand Expression by Prostate Cancer: A Novel Marker of Prognosis and Potential Target for Therapy. Cancer Research. 2007;67(16):7893-900.

6. Powderly J. 2015 Society for Immunotherapy of Cancer (SITC) Annual Meeting in National Harbor, MD. Dr. John Powderly II of the Carolina BioOncology Institute, Huntersville, NC, presented “Interim Results of an Ongoing Phase 1, Dose Escalation Study of MGA271 (Fc-optimized Humanized Anti-B7-H3 Monoclonal Antibody) in Patients with Refractory B7-H3-Expressing Neoplasms or Neoplasms Whose Vasculature Expresses B7-H3”.

7. Loo D, Alderson RF, Chen FZ, Huang L, Zhang W, Gorlatov S, Burke S, Ciccarone V, Li H, Yang Y, Son T, Chen Y, Easton AN, Li JC, Rillema JR, Licea M, Fieger C, Liang TW, Mather JP, Koenig S, Stewart SJ, Johnson S, Bonvini E, Moore PA. Development of an Fc-Enhanced Anti–B7-H3 Monoclonal Antibody with Potent Antitumor Activity. Clinical Cancer Research. 2012;18(14):3834-45.

**Ethics Approval**

The study was approved by Johns Hopkins Institutions's Ethics Board, approval number IRB00070748.

**Fig. 1 (abstract P338).**
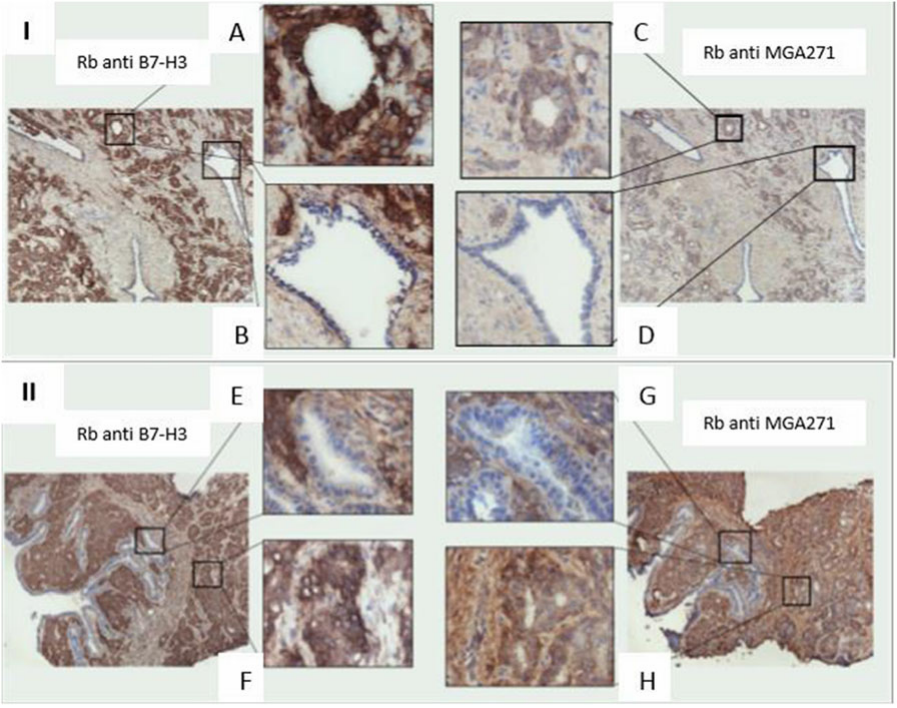
Enoblituzumab binds B7-H3 with high affinity and specificity

**Fig. 2 (abstract P338).**
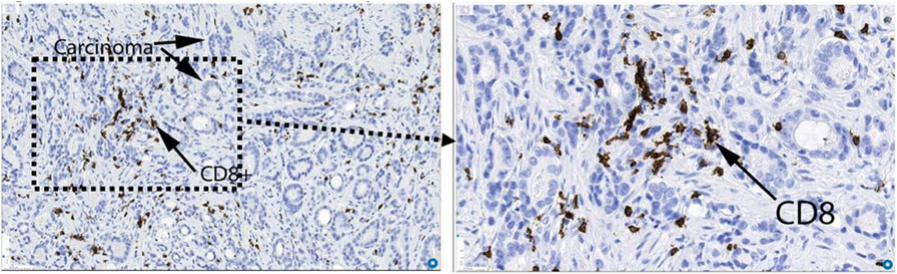
Enoblituzumab Treated Prostatectomy CD8+ T Cell Infiltrates

**Fig. 3 (abstract P338).**
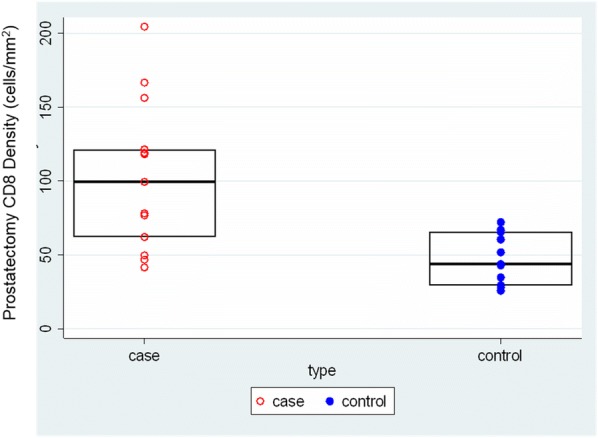
CD8+ T Cell Quantitation in Enoblituzumab-treated Prostates

#### P339 HCRN GI16-288: A phase II trial of perioperative CV301 vaccination in combination with nivolumab and systemic chemotherapy for resectable hepatic-limited metastatic colorectal cancer

##### Kristen Spencer, DO, MPH^1^, Daniella Portal, BS^1^, Christopher Heery, MD^2^, Lynne Bauman, MS^2^, Howard Hochster, MD^1^, Elizabeth Poplin, MD^1^, Usha Malhotra, MD^1^, Richard Alexander, MD^1^, David August, MD^1^, Timothy Kennedy, MD^1^, Miral Grandhi, MD^1^, Russell Langan, MD^1^, Edmund Lattime, PhD^1^, Dirk Moore, PhD^1^, Michael Kane, RPh, BCOP^1^, Liesel Dudek, RN, OCN, CCRP^1^, Sarah Nabeel^1^, Darren Carpizo, MD, PhD^1^

###### ^1^Rutgers Cancer Institute of New Jersey, New Brunswick, NJ, USA; ^2^Bavarian Nordic, Morrisville, NC, USA

####### **Correspondence:** Kristen Spencer *Corresponding author email: spencekr@cinj.rutgers.edu

**Background**

Approximately 50% of patients with colorectal cancer (CRC) develop metastatic disease, most frequently in the liver. Surgical resection is the only potential cure, however recurrence occurs in a majority of cases. To date, perioperative chemotherapy has not translated into an overall survival (OS) benefit [1, 2]. Immune checkpoint inhibitors are active in mCRC patients with high microsatellite instability [3], however studies in an array of CRCs have demonstrated a relationship between the tumor immune microenvironment and outcomes, implying immune strategies may be used in a broader population [4, 5].CV301, formerly PANVAC, is a vector-based vaccine that expresses the tumor antigens carcinoembryonic antigen (CEA) and mucin 1 (MUC1), and in a phase II study in patients with mCRC who had undergone complete resection of liver metastases did not improve RFS, but significantly improved OS as compared to an unvaccinated contemporary control cohort (median OS NR vs 44.1 mos) [6]. This study highlighted that: 1) the optimal use of this vaccine may be in patients free of detectable (and immunosuppressive) disease and 2) the gradual immune response generated through epitope spreading may mean long-term outcomes such as OS is a more appropriate primary endpoint. Additionally, CV301 up-regulates PD-L1 in the tumor microenvironment, suggesting a role for rational immunotherapy combinations [7].

**Methods**

This is a multi-center Phase II randomized study to determine whether the addition of CV301 vaccination to perioperative Nivolumab and mFOLFOX improves 3-year OS in 78 patients undergoing surgical resection for mCRC. Eligible patients are those with resectable hepatic-limited mCRC and ECOG performance status

**Acknowledgements**

This trial is managed by Hoosier Oncology Group and sponsored by Bavarian Nordic and Bristol-Myers Squibb.

**Trial Registration**

Clinicaltrials.gov: NCT02840994

**References**

1. Nordlinger B, Sorbye H, Glimelius B, Poston GJ, Schlag PM, Rougier P, Bechstein WO, Primrose JN, Walpole ET, Finch-Jones M, Jaeck D, Mirza D, Parks RW, Collette L, Praet M, Bethe U, Van Cutsem E, Scheithauer W, Gruenberger T. Perioperative chemotherapy with FOLFOX4 and surgery versus surgery alone for resectable liver metastases from colorectal cancer (EORTC Intergroup trial 40983): a randomised controlled trial. Lancet. 2008;371(9617):1007-16.

2. Nordlinger B, Sorbye H, Glimelius B, Poston GJ, Schlag PM, Rougier P, Bechstein WO, Primrose JN, Walpole ET, Finch-Jones M, Jaeck D, Mirza D, Parks RW, Mauer M, Tanis E, Van Cutsem E, Scheithauer W, Gruenberger T. Perioperative FOLFOX4 chemotherapy and surgery versus surgery alone for resectable liver metastases from colorectal cancer (EORTC 40983): long-term results of a randomised, controlled, phase 3 trial. Lancet Oncol. 2013;14(12):1208-15

3. Overman MJ, McDermott R, Leach JL, Lonardi S, Lenz HJ, Morse MA, Desai J, Hill A, Axelson M, Moss RA, Goldberg MV, Cao ZA, Ledeine JM, Maglinte GA, Kopetz S, Andre T. Nivolumab in patients with metastatic DNA mismatch repair-deficient or microsatellite instability-high colorectal cancer (CheckMate 142): an open-label, multicentre, phase 2 study. Lancet Oncol. 2017;18(9):1182-1191.

4. Galon J, Costes A, Sanchez-Cabo F, Kirilovsky A, Mlecnik B, Lagorce-Pagès C, Tosolini M, Camus M, Berger A, Wind P, Zinzindohoué F, Bruneval P, Cugnenc PH, Trajanoski Z, Fridman WH, Pagès F. Type, density, and location of immune cells within human colorectal tumors predict clinical outcome. Science. 2006; 313(5795):1960- 4.

5. Pagès F1, Berger A, Camus M, Sanchez-Cabo F, Costes A, Molidor R, Mlecnik B, Kirilovsky A, Nilsson M, Damotte D, Meatchi T, Bruneval P, Cugnenc PH, Trajanoski Z, Fridman WH, Galon J. Effector memory T cells, early metastasis, and survival in colorectal cancer. N Engl J Med. 2005;353(25):2654-66.

6. Morse MA, Niedzwiecki D, Marshall JL, Garrett C, Chang DZ, Aklilu M, Crocenzi TS, Cole DJ, Dessureault S, Hobeika AC, Osada T, Onaitis M, Clary BM, Hsu D, Devi GR, Bulusu A, Annechiarico RP, Chadaram V, Clay TM, Lyerly HK. A randomized phase II study of immunization with dendritic cells modified with poxvectors encoding CEA and MUC1 compared with the same poxvectors plus GM-CSF for resected metastatic colorectal cancer. Ann Surg. 2013;258(6):879-86

7. Foy SP, Sennino B, dela Cruz T, Cote JJ, Gordon EJ, Kemp F, Xavier V, Franzusoff A, Rountree RB, Mandl SJ. Poxvirus-based active immunotherapy with PD-1 and LAG-3 dual immune checkpoint inhibition overcomes compensatory immune regulation, yielding complete tumor regression in mice. PLoS One. 2016 Feb 24;11(2):e0150084.

**Ethics Approval**

This study was approved by the Rutgers Cancer Institute of New Jersey Institutional Review Board (Pro20170000595).

**Fig. 1 (abstract P339).**
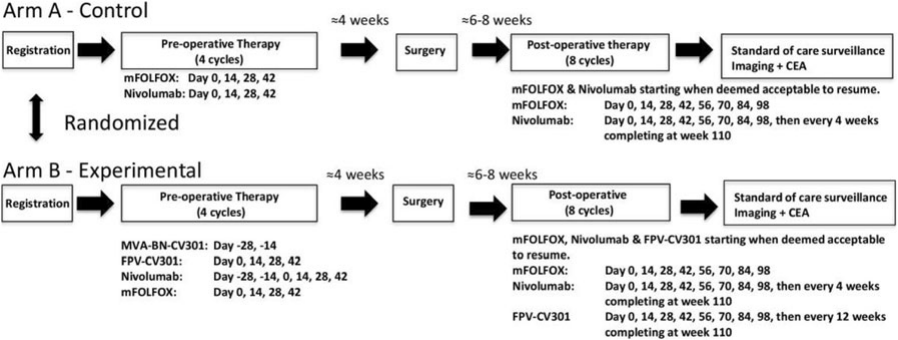
See text for description.

#### P340 Pharmacokinetic and pharmacodynamic characterization of ALX148, a CD47 blocker, in patients with advanced malignancy and non-Hodgkin lymphoma

##### Hong Wan^1^, Feng Jin, PhD^1^, Nehal Lakhani, MD, PhD^2^, Patricia LoRusso, DO^3^, Laura Chow, MD^4^, Wells Messersmith, MD^5^, Sangetha Bollini^1^, Laura Doyle^1^, Steven Kauder, PhD^1^, Philip Fanning^1^, Jaume Pons^1^, Sophia Randolph, MD, PhD^1^

###### ^1^ALX Oncology, Burlingame, CA, USA; ^2^START Midwest, Grand Rapids, MI, USA; ^3^Yale Cancer Center, New Haven, CT, USA; ^4^Seattle Cancer Care Alliance, Seattle, WA, USA; ^5^University of Colorado Cancer Center, Aurora, CO, USA

####### **Correspondence:** Hong Wan (Hong@alxoncology.com)

**Background**

ALX148 is a fusion protein comprising a high affinity CD47 blocker with an inactive human Fc domain to extend serum half-life and minimize toxicity[1]. ALX148 safely enhances the activity of anticancer antibodies in nonclinical models[1] and is currently being investigated in a Phase 1 study (SITC 2017, #241; ASCO 2018, #3068). The objective of this analysis was to characterize the pharmacokinetics (PK) and pharmacodynamics (PD) of ALX148 in patients with advanced solid tumors and lymphomas as a single agent and in combinations with pembrolizuab or trastuzumab. Translational PK and PD modeling was conducted to estimate target occupancy (TO) in human tumors.

**Methods**

Data from the first-in-human Phase 1 study in 48 patients with advanced malignancy were used for this analysis. Multiple intravenous (IV) doses in the range 0.3 – 30 mg/kg were administered once weekly (QW) or once every two weeks (QoW). ALX148 was quantitated using an immunoassay in serum samples collected after first and subsequent doses. CD47 TO was measured in peripheral blood by flow cytometry. Noncompartmental PK analysis and PK/PD modeling were conducted with Phoenix and NONMEM programs. Preclinical PK/PD analysis was performed with ALX148 in tumor bearing mice, and serum PK and TO in peripheral blood, spleen and tumor compartments were measured.

**Results**

Maximum serum concentration (Cmax) and area under the concentration curve (AUC) of ALX148 exhibited greater than dose-proportional increase at 0.3 - 3 mg/kg QW, indicating nonlinearity in PK. At 10 mg/kg QW and 30 mg/kg QoW, increases in Cmax and AUC were dose proportional. The PK parameters and observed TO data were consistent with the presence of a CD47 antigen sink saturated at doses ≥ 3 mg/kg QW. ALX148 PK profile (10 mg/kg QW) was not changed when dosed in combination with pembrolizumab or trastuzumab, with a steady-state half-life of ~ 16 days. In tumor bearing mice, administration of ALX148 demonstrated dose dependent TO in peripheral blood, spleen and tumor. Using PK/PD models established from preclinical data and extrapolated based on human PK and peripheral PD data, a tissue/tumor TO >85% was projected to be maintained at ≥ 5 mg/kg QW in patients over the dosing interval.

**Conclusions**

ALX148 exhibited clinical PK properties typical of antibody-type protein therapeutics directed towards cell-surface targets. ALX148 PK approached linear range and maintained complete peripheral TO over the dosing interval at ≥ 3 mg/kg QW. Initial data suggests ALX148 PK/PD profiles are not impacted by combination drugs.

**Trial Registration**

ClinicalTrials.gov identifier NCT03013218

**References**

1. Kauder S et al., ALX148 blocks CD47 and enhances innate and adaptive antitumor immunity with a favorable safety profile. PLOS ONE. In Press.

#### P341 Phase 1 study of CA-170, a first-in-class, orally available, small molecule immune checkpoint inhibitor (ICI) dually targeting VISTA and PD-L1, in patients with advanced solid tumors or lymphomas

##### Timothy Wyant, PhD^1^, Yung-Lue Bang, MD^2^, Jeffrey Sosman, MD^3^, Adil Daud, MBBS MD^4^, Funda Meric- Bernstam, MD^5^, Javier Garcia-Corbacho^6^, Manish Patel, MD^7^, James Lee, MD, PhD^8^, Kyu-Pyo Kim^9^, Joshua Brody, MD^10^, Sun Young Rha^11^, Marta Gil Martin^12^, Santiago Ponce Aix, MD^13^, Erika Hamilton, MD^14^, Radhakrishnan Ramchandren, MD^15^, Myung-Ju Ahn^16^, James Spicer, MD, PhD^17^, Simon Pacey^18^, Gerald Falchook, MD^19^, Hongwei Wang, MD, PhD^1^, Guangxin Xu^20^, Lisa Adams, BS^20^, Anna Ma, MS^20^, Robert Ghararvi^20^, David Tuck^20^, John Powderly, MD, CPI^21^

###### ^1^Curis Inc, Lexington, MA, USA; ^2^Seoul National University, Seoul, Korea, Republic of; ^3^Northwestern, Chicago, IL, USA; ^4^UCSF, San Francisco, CA, USA; ^5^MDACC, Houston, TX, USA; ^6^Hospital Clinic i Provincial, Barcelona, Spain; ^7^Florida Cancer Specialist SCRI, Sarasota, FL, USA; ^8^UPMC, Pittsburgh, PA, USA; ^9^Asan Medical Center, Seoul, Korea, Republic of; ^10^Mt. Sinai, New York, NY, USA; ^11^Yonsei University Health System - Severa, Seoul, Korea, Republic of; ^12^Catalan Institute of Oncology, Catalon, Spain; ^13^Hospital Universitario 12 de Octubre, Madrid, Spain; ^14^Sarah Cannon Research Insitute/Tennessee, Nashville, TN, USA; ^15^Karmanos, Detroit, MI, USA; ^16^Samsung Medical Center, Seoul, Korea, Republic of; ^17^King's College Guy's, London, UK; ^18^Cambridge University Hospitals NHS Found, Cambridge, UK; ^19^SCRI, Denver, CO, USA; ^20^Curis, Lexington, MA, USA; ^21^Carolina BioOncology Institute, Huntersville, NC, USA

####### **Correspondence:** Hongwei Wang (hwwang.2004@gmail.com)

**Background**

VISTA and PD-1 are independent immune checkpoints that negatively regulate T-cell function. VISTA is expressed on immune and tumor cells and is found to be upregulated in tumors as a potential resistance mechanism after ICI therapy. Pre-clinical studies demonstrated that dual blockade of both checkpoints can be synergistic. CA-170 directly targets VISTA and PDL1/L2 and demonstrated significant anti-tumor activity in multiple preclinical models.

**Methods**

Enrollment initially followed accelerated titration and subsequently switched to 3+3 design. Two dosing schedules [once daily (QD) and twice daily (BID)] were evaluated in dose escalation. Cohorts of selected dose levels were expanded with additional patients. The expansion phase allows for enrollment of patients with selected indications. Primary objectives: safety, maximum tolerated dose (MTD) and recommended Phase 2 dose. Secondary objectives: pharmacokinetics (PK) and anti-tumor activity.

**Results**

A total of 59 patients have been treated across 9 dose levels (50 – 800 mg QD and 600 – 1200 mg BID) with 52 evaluable for dose limiting toxicities (DLTs). Enrolled tumor types included lung (20%), colorectal (17%), head and neck (14%), and ovarian cancer (8%). No DLT has been observed thus far. The most common treatment-emergent AEs (all grades) were fatigue (30%), nausea (27%), vomiting (21%), decreased appetite (20%), anemia (18%), constipation (18%), cough (16%), headache (13%), pyrexia (13%), and insomnia (12%). These were predominantly low grade and self-limiting events. Two reversible SAEs possibly related to drug were reported: one grade 3 lipase elevation and one grade 3 vomiting at 800 mg QD. Three newly enrolled patients are pending restaging. Fifty-one patients were evaluable for anti-tumor activity with 25 showing stable disease, 9 on study for ≥ 6 cycles (2 ongoing in cycle 26 and 8; most prolonged SD = 18 months), and 7 showing tumor shrinkage. CA-170 exhibited approximately dose proportional plasma exposure for both QD and BID schedules with T1/2 no longer than 12 hours. Compared with QD steady state plasma levels, BID dosing at the same dose resulted in approximately 50% increase in Cmax, 4.3-fold increase in Cmin, and an approximately two-fold increase in AUC/day.

**Conclusions**

These data suggest CA-170 has an acceptable safety profile with preliminary signs of anti-tumor activity and approximately dose proportional PK profile. MTD has not been reached. These data warrant the continued clinical development of CA-170. The study is ongoing with evaluation of potentially pharmacologically active BID dose in selected VISTA-expressing cancer types, such as mesothelioma.

**Trial Registration**

NCT02812875

**Ethics Approval**

This study was approved by the ethics committees of the following institutions: Seoul National University Hospital, Northwestern, UCSF, MDACC, Hospital Clinic de Barcelona, Florida Cancer Specialists/Sarah Cannon Research Institute, UPMC, Asan Medical Center, Mt. Sinai, Yonsei University Health System - Severance Hospital, Catalan Institute of Oncology, Hospital Universitario 12 de Octubre, Tennessee Oncology/Sarah Cannon Research Institute, Karmanos, Samsung Medical Center, King’s College London, Guy’s Hospital, University of Cambridge, Sarah Cannon Research Institute at HealthONE, Carolina BioOncology Institute

**Consent**

Written informed consent was obtained from the patient for publication of this abstract and any accompanying images. A copy of the written consent is available for review by the Editor of this journal

#### P342 Nivolumab plus cisplatin/pemetrexed or cisplatin/gemcitabine as induction in resectable NSCLC

##### Ralph Zinner, MD^2^, Scott Cowan, MD^2^, Charalambos Solomides, MD^2^, Craig Hooper, PhD^2^, Larry Harshyne, PhD^2^, Grace Lu-Yao, PhD^2^, Hushan Yang, PhD^2^, Linda Phan, BS^2^, Dawn Poller, BS^2^, Sung Whang, DNP, CRNP^2^, Benjamin Leiby, PhD^2^, Marie Werner-Wasik, MD^2^, Bo Lu, MD^2^, Jennifer Johnson, MD^2^, Rita Axelrod, MD^2^, Argiris Athanassios, MD PhD^2^, Natathiel Evans, MD^2^

###### ^1^Thomas Jefferson University Hospital, Philadelphia, PA, USA; ^2^Thomas Jefferson University, Philadelphia, PA, USA

####### **Correspondence:** Ralph Zinner (ralph.zinner@jefferson.edu)

**Background**

For patients (pts) with stage IB (>4cm)-IIIA Non-small-cell lung cancer (NSCLC), multi-modality therapy yields a modest improvement in 5 year post-surgical overall survival (OS), with comparable benefit for induction and postoperative adjuvant chemotherapy (chemo). Induction can speed the discovery of promising regimens by using pathologic response as a surrogate for OS. About 20% of pts treated with induction chemo have major pathologic response (MPR) (< 10% viable tumor) at primary and lymph nodes while pathologic complete responses (pCR) average 4%. MPR was strongly associated with improved OS [1]. PD-1 checkpoint inhibitors (CI), nivolumab (nivo), pembrolizumab (pembro), and the PD-L1 CI, atezolizumab, are established in advanced NSCLC as 2nd line therapy, and pembro is approved as a single agent as 1st line treatment of pts with PD-L1 high expressing tumors. In a phase III 1st line NSCLC study, pts with high mutational burden tumors had superior OS with nivo plus ipilimumab compared to doublet chemo. Pembro plus carboplatin with pemetrexed (P) was approved as 1st line therapy based on a randomized phase II study in advanced NSQ NSCLC showing improved clinical response and PFS compared to chemo alone with no increase in grade III toxicity. We therefore hypothesize that the addition of nivo to induction cisplatin (C) P or C gemcitabine (G) will increase the MPR rate over induction chemo alone compared to historical controls.

**Methods**

This is an investigator initiated trial for pts with newly diagnosed clinical stage I-IIIA (stage I > 4cm) SQ and NSQ NSCLC. Induction is C 75mg/m2 IV q 3w x 3 plus either P 500 mg/m2 IV q 3wks x 3 or G 1250mg/m2 IV d1, d8 q 3 wks x 3 plus nivo 360mg IV q 3w x 3. Surgery is planned 3 wks after the last dose. The primary outcome is MPR. Secondary outcomes include safety, pCR, overall clinical response rate, clinical CR, 1 year PFS, OS and exploratory outcomes assessing markers of immune bias. Enrollment will be 34 pts. NCT03366766

**References**

1. Hellmann MD, Chaft JE, William WN Jr., Rusch V, Pisters KM, Kalhor N, Pataer A, Travis WD, Swisher SG, Kris MG. Pathological response after neoadjuvant chemotherapy in resectable non-small-cell lung cancers: proposal for the use of major pathological response as a surrogate endpoint. Lancet Oncol. 2014;15;e42-50.

**Ethics Approval**

The study was approved by Thomas Jefferson University Hospital Internal Review Board approval number 17P545

**Consent**

There is no identifiable information on any of the patients.

#### P343 Nivolumab plus weekly carboplatin and paclitaxel as induction therapy in resectable locally advanced head and neck cancer

##### Ralph Zinner, MD^1^, David Cognetti, MD^2^, Joseph Curry, MD^2^, Adam Luginbuhl, MD^2^, Richard Goldman, MD^2^, Charalambos Solomides, MD^2^, Madalina Tuluc, MD^2^, Stacey Mardekian, MD^2^, Craig Hooper, PhD^2^, Larry Harshyne, PhD^2^, Grace Lu-Yao, PhD^2^, Hushan Yang, PhD^2^, Linda Phan, BS^2^, Dawn Poller, BS^2^, Benjamin Leiby, PhD^2^, Voichita Bar-Ad, MD^2^, Jennifer Johnson, MD^2^, Rita Axelrod, MD^2^, Argiris Athanassios, MD PhD^2^

###### ^1^Thomas Jefferson University Hospital, Philadelphia, PA, USA; ^2^Thomas Jefferson University, Philadelphia, PA, USA

####### **Correspondence:** Ralph Zinner (ralph.zinner@jefferson.edu)

**Background**

Despite multimodality standard therapy, patients (pts) with resectable locally advanced squamous cell carcinoma of the head and neck (SCCHN) are at high risk for locoregional and distant recurrence. Although induction chemotherapy followed by surgery and postoperative radiotherapy did not show improved overall survival (OS) in 2 phase III trials of pts with resectable oral cancer, pts with pathologic complete response (pCR) and/or major pathologic response (MCR) had improved progression free survival (PFS) and OS [1,2]. In addition, major tumor responses may decrease surgical morbidity and reduce postoperative radiation fields [2]. Therefore, induction regimens which improve pCR/ MCR rates may improve survival and morbidity. PD-1 checkpoint inhibitors, nivolumab and pembrolizumab, are established in recurrent or metastatic SCCHN as 2nd line therapy. In a randomized phase III in 1st-line advanced non-squamous non-small cell lung cancer, carboplatin plus pemetrexed plus pembrolizumab improved clinical response, PFS, and OS compared to chemotherapy alone. We hypothesize that pts with newly diagnosed, previously untreated SCCHN, induction nivolumab in combination with weekly carboplatin and paclitaxel will increase the pCR rate at the primary cancer compared to the historical control chemotherapy alone.

**Methods**

This is an investigator-initiated trial for pts with newly diagnosed (AJCC 8th) stage III or IV HPV negative and stage III HPV positive SCCHN (oral cavity, oropharynx, hypopharynx, and larynx) who are surgical candidates. Pts receive induction with carboplatin AUC IV Q1 wk x 6 plus paclitaxel 100 mg/m2 IV Q1 wk x 6 with nivolumab 240mg IV Q2 wks x 3, all beginning on day 1 with surgery wk 8. The primary objective is pCR at the primary site. Secondary objectives include safety, pCR at all sites, MCR at primary site, overall clinical response, clinical complete response, 1 year PFS, OS and exploratory objectives assessing markers of immune bias. Two of planned 37 pts have been enrolled. NCT03342911

**References**

1. Zhong LP, Zhang CP, Ren GX, Guo W, William WN Jr, Hong CS, Sun J, Shu HG, Tu WY, Li J, Cai YL, Yin QM, Wang LZ, Wang ZH, Hu YJ, Ji T, Yang WJ, Ye WM, Li J, He Y, Wang YA, Xu LQ, Zhuang Z, Lee JJ, Myers JN, Zhang ZY. Long-term results of a randomized phase III trial of TPF induction chemotherapy followed by surgery and radiation in locally advanced oral squamous cell carcinoma.Oncotarget,2015;6:18708-14.

2. Bossi P, Lo Vulio S, Buzzo M, Mariani L, Granata R, Orlandi E, Locati L, Scaramellini G, Fallai C, Licitra L. Preoperative chemotherapy in advanced resectable OCSCC: long-term results of a randomized phase III trial. Ann Oncol. 2014;25:462-6

**Ethics Approval**

This study was approved by Thomas Jefferson University Internal Review Board approval number 17P502

**Consent**

There is no identifiable information on any patient.

### Combination Therapy

#### P344 Spherical Nucleic Acid (SNA) TLR9 agonists induce long-term tumor-specific immune responses in synergy with PD-1 checkpoint inhibition

##### Bart Anderson, PhD, SubbaRao Nallagatla, PhD, Richard Kang, PhD, Ekambar Kandimalla, PhD

###### Exicure, Skokie, IL, USA

####### **Correspondence:** Bart Anderson (banderson@exicuretx.com)

**Background**

Novel spherical nucleic acid (SNA) configuration of toll-like receptor (TLR) 9 agonist oligonucleotides are designed to trigger innate and adaptive immune responses against tumor cells in cancer patients. SNAs are densely-packed, radially-oriented 3-dimensional arrangements of oligonucleotides surrounding a liposomal nanoparticle. This 3D- architecture increases cellular uptake compared to conventional “linear” oligonucleotides that are not in SNA configuration. SNAs enter cells and localize to endosomes, which is where TLR9 proteins are localized, making SNAs ideal TLR9 agonists.Immune checkpoints are inhibitory pathways crucial for maintaining self-tolerance and modulating the duration and amplitude of physiological immune responses. However, tumors use immune- checkpoint pathways, particularly the PD-1 / PD-L1 pathway, as a major mechanism of immune resistance. TLR9 agonists combined with anti-PD-1 antibodies show synergistic anti-cancer effects. Here, we assess SNA immunostimulation and anti-tumor effects combined with an anti-PD-1 antibody.

**Methods**

Uptake of fluorescently-labeled oligonucleotides was measured by microscopy and flow cytometry. Mouse serum cytokines were measured by multiplex ELISA.Subcutaneously implanted EMT-6 cells were used as a tumor model. PD-1 and PD-L1 expression were measured using RT-qPCR. Tumor growth was assessed following SNA and anti- PD-1 treatment. Surviving mice were re-challenged with EMT-6 cells or with distinct syngenic tumor cells.The immunological response to subcutaneously administered SNAs in monkeys was assessed by Luminex-based measurement of serum cytokines and flow cytometric measurement of immune cell activation.

**Results**

Oligonucleotide uptake in SNA format was significantly increased (P<0.0001) compared to linear oligonucleotide (Fig.1). TH1-type cytokines were induced in wild-type but not TLR9-deficient mice, confirming TLR9-dependent immunostimulation (Fig.2).SNA treatment upregulated PD-1 and PD-L1 expression in EMT-6 tumors (Fig.3). Combined SNA plus anti-PD-1 treatment, but not anti-PD-1 monotherapy, produced complete anti-tumor responses. Re-challenge with EMT-6 tumor cells in mice previously experiencing a complete response resulted in tumor rejection, but tumors were not rejected when mice were re-challenged with distinct tumor cell lines, verifying adaptive immune responses against EMT-6 cells (Fig.4).SNA administration to NHP elicited both TH1-type cytokines in serum and activated immune cells including B-cells and pDCs (Fig.5).

**Conclusions**

TLR9-agonist SNAs induced potent, TLR9-dependent TH1-type immune responses in mice and monkeys and increased checkpoint inhibitor expression in the tumor. SNA plus anti-PD-1 combination therapy induced tumor- specific immunity and memory. These data support the clinical investigation of SNAs in immuno-oncology. One such SNA, AST-008, is undergoing a Phase 1a clinical trial and is planned for testing in cancer patients combined with an anti-PD-1 antibody.

**Fig. 1 (abstract P344).**
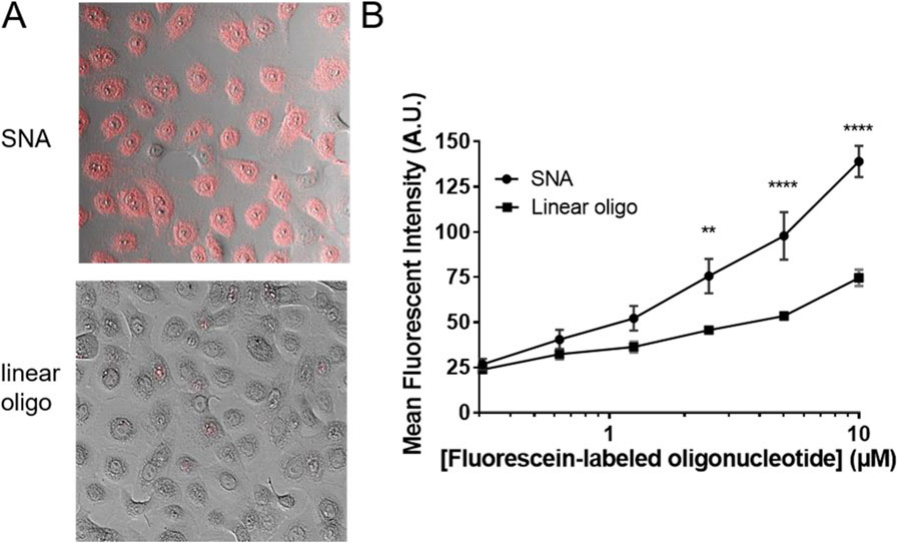
See text for description.

**Fig. 2 (abstract P344).**
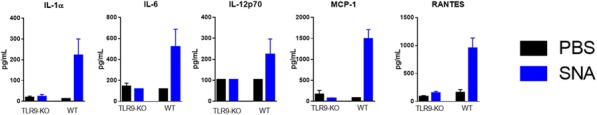
See text for description.

**Fig. 3 (abstract P344).**
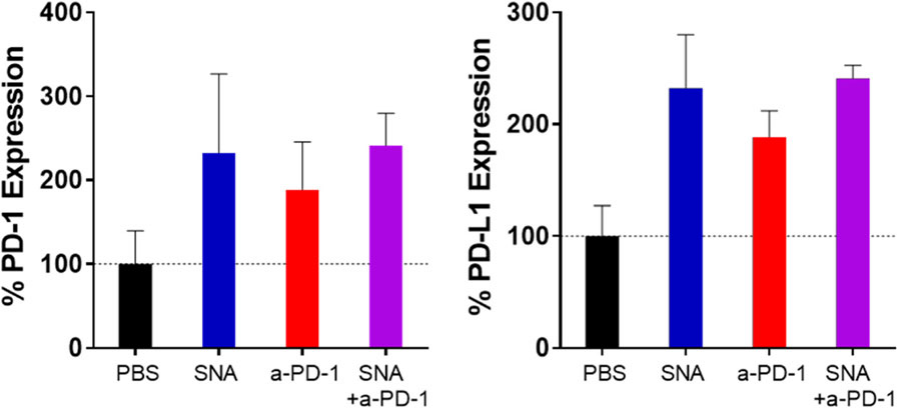
See text for description.

**Fig. 4 (abstract P344).**
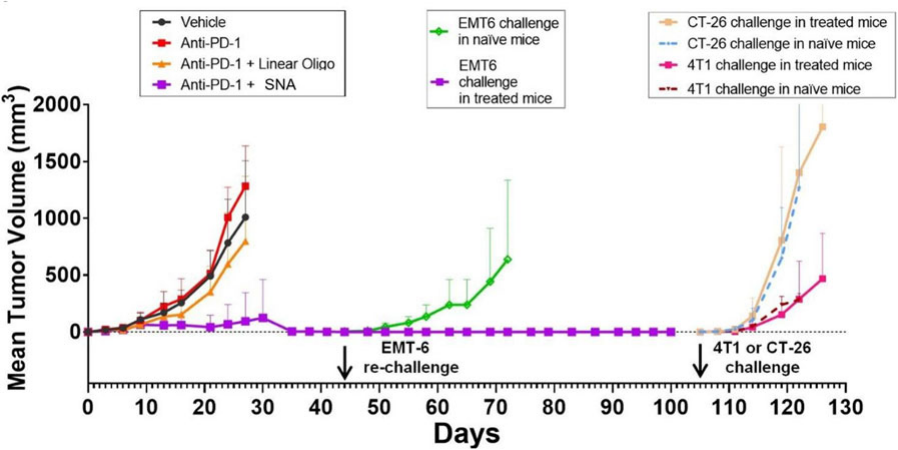
See text for description.

**Fig. 5 (abstract P344).**
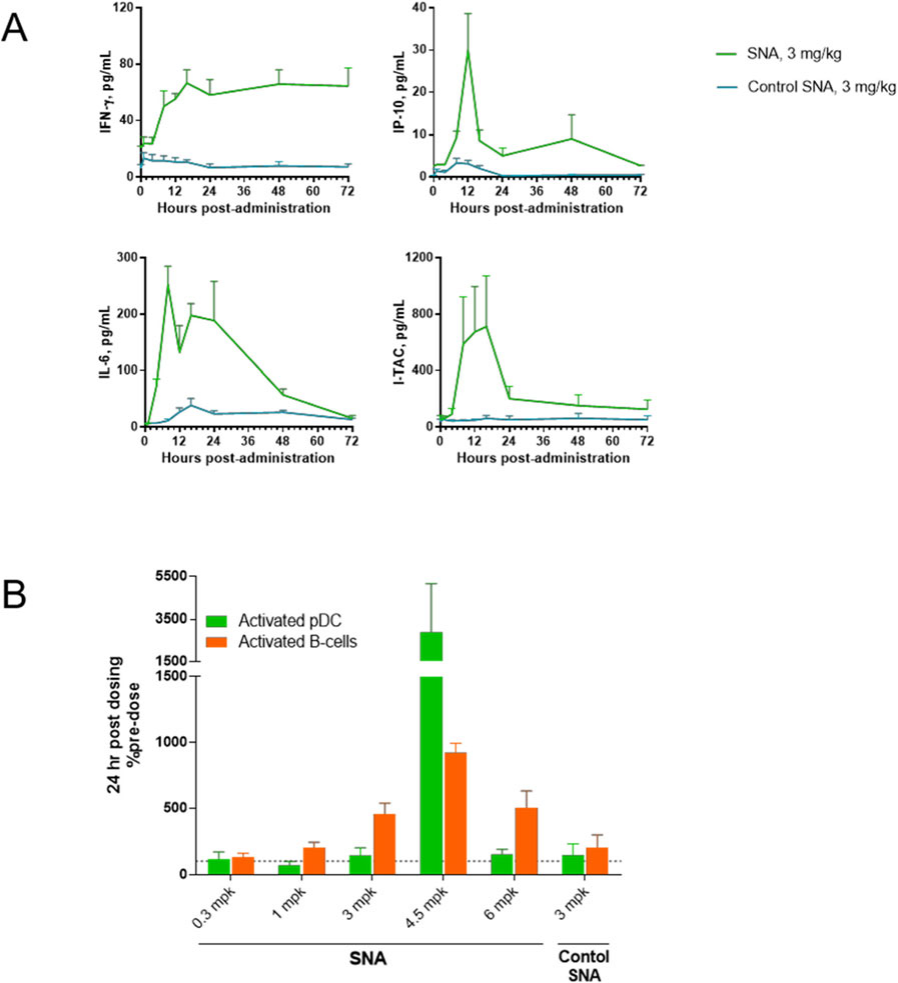
See text for description.

#### P345 Depletion of plasma arginine with pegzilarginase enhances the anti-tumor activity of immune checkpoint inhibitors

##### Mark Badeaux, PhD, Giulia Agnello, PhD, Danlee Enzler, BS, Leslie Priddy, BS, Jason Wiggins, BS, Christopher Daige, Scott Rowlinson, PhD

###### Aeglea Biotherapeutics, Austin, TX, USA

####### **Correspondence:** Scott Rowlinson (swr@aegleabio.com)

**Background**

Tumors unable to endogenously synthesize L-Arginine (arginine) due to defects in the arginine biosynthetic pathway are highly sensitive to arginine depletion. Pegzilarginase is a bioengineered human PEGylated arginase 1 with enhanced pharmacological properties that displays single-agent anti-tumor activity in a number of preclinical solid tumor models and clinical activity in dose escalation studies. Extracellular depletion of arginine directly impairs tumor cell growth, inducing both autophagy and apoptosis [1]. Given the relationship between autophagy and antigen presentation [2], we hypothesized that pegzilarginase activity could enhance immune cell recruitment and function in the tumor microenvironment, and therefore may exhibit enhanced anti-tumor activity in combination with immuno-oncology (IO) agents.

**Methods**

Pegzilarginase, either as a monotherapy or in combination with IO agents (anti-CTLA-4 Ab, anti-PD-L1 Ab), was administered to Balb/c mice bearing subcutaneous CT26 tumors. Tumor volumes were measured at regular intervals until survival endpoints were reached. At pre-determined time points, blood was collected for cytokine analyses, and flow cytometry was employed to assess intratumoral cell viability and immunophenotyping.

**Results**

Treatment with pegzilarginase or IO agents alone demonstrated greater anti-tumor activity relative to control treatment. The combination regimen of either anti-CTLA-4 Ab or anti-PD-L1 Ab with pegzilarginase induced greater anti-tumor activity than either IO monotherapy, including an increase in complete responses (CRs) in anti- PD-L1 Ab studies (37.5% CRs in combination arm vs. 0% in anti-PD-L1 Ab arm). Tumors treated with pegzilarginase underwent autophagy and early apoptosis during the initial period of systemic arginine depletion. Pegzilarginase treatment increased the fraction of CD8+ T cells expressing early activation markers CD69 and CD25. The combination treatment regimen induced a significant increase in serum interferon-gamma levels, as well as an increase in total and activated intratumoral CD8+ T cells relative to either monotherapy.

**Conclusions**

Pegzilarginase monotherapy enhances tumor immune infiltration and early CD8+ T cell activation. The combination of pegzilarginase with IO agents results in greater anti-tumor activity than either IO agent alone and is marked by an increase in tumor-infiltrating immune cells, including a large number of activated cytotoxic T cells. These findings support pursuit of clinical studies combining pegzilarginase with immuno-oncology agents.

**References**

1. Patil M et al. Arginine dependence of tumor cells: Targeting a chink in cancer’s armor. Oncogene. 2016; 35: 4957-722. You L et al. The crosstalk between autophagic and endo-exosomal pathways in antigen processing for MHC presentation in anticancer T cell immune responses. J Hematol Oncol. 2017; 10: 165.

#### P346 A phase 1b/2 study of the safety and efficacy of HBI-8000-nivolumab combination in melanoma (MEL), renal cell carcinoma (RCC) and non-small cell lung cancer (NSCLC)

##### Reid Bissonnette^1^, Nikhil Khushalani, MD^2^, Zeynep Eroglu, MD^2^, Andrew Brohl, MD^2^, Thai Ho, MD, PhD^3^, Heather Yeckes-Rodin, MD^4^, Michael Kurman, MD^1^, Mireille Gillings, PhD^1^, Gloria Lee, MD, PhD^1^

###### ^1^HUYA Bioscience International, San Diego, CA, USA; ^2^H. Lee Moffitt Cancer Center, Tampa, FL, USA; ^3^Mayo Clinic Arizona, Phoenix, AZ, USA; ^4^Hematology-Oncology Associates of the Treasure Coast, Port St Lucie, FL, USA

####### **Correspondence:** Gloria Lee (glee@huyabio.com)

**Background**

HBI-8000 is a Class I selective oral benzamide histone deacetylase inhibitor (HDACi). It is in registration trials in Japan and Korea for lymphoma and marketed in China. Its demonstrated immune modulatory effects include (i) enhanced immune cell-mediated cytotoxicity; (ii) enhanced tumor infiltration, and (iii) decreased tumor infiltration and expansion of T-regulatory and myeloid derived suppressor cells that suppress antitumor immunity.

**Methods**

The safety of HBI-8000 in combination with standard dose nivolumab (NIVO) was evaluated in a Phase 1b trial utilizing a 3+3 design escalating from 20, 30 to 40 mg twice weekly (BIW). The recommended Phase 2 dose (RP2D) was determined by dose limiting toxicity (DLT). In Phase 2, the disease cohorts were expanded to explore the efficacy of HBI-8000 at RP2D in combination with NIVO. Pharmacokinetics (PK), thorough QTc and pharmacodynamics (PD) were also studied.

**Results**

Phase 1b evaluated 15 pts (6 RCC, 5 MEL, 4 NSCLC,) for DLT; DLTs (1 fatigue G3 and 1 headache G3) were observed at 40 mg BIW. 30 mg BIW was selected as the RP2D. Tumor responses were 6 PRs (40%; 3 MEL, 2 RCC, 1 NSCLC); 5 SD; and 4 PD. At RP2D, 47 pts with Mel, NSCLC and RCC with or without prior checkpoint inhibitor (CPI) therapy were enrolled. As of Jul 20 2018, 20 MEL CPI-naïve pts (including Ph1) were evaluable for response with an objective response rate of 65% (1 CR, 10 PR). The median follow-up was 110 days, median time to response 56 days (range: 53-112) and the median progression-free survival had not been reached (range: 12 – 515+). Thirteen patients continue on treatment. The most common side effects attributed to HBI-8000 were Grade 3 hypophosphatemia (N=11), neutropenia (N=6), lymphopenia (N=5), fatigue (N=3), thrombocytopenia (N=2), nausea/anorexia (N=2), headache, diarrhea, anemia, transaminase elevation (N=1 each), and G4 lymphopenia (N=1). No increase of irAEs were detected. A QTc study with time-matched PK found no evidence of QTc prolongation. Patient accrual is ongoing.

**Conclusions**

The combination of HBI-8000 and nivolumab was well tolerated without new toxicity concerns. Preliminary efficacy is encouraging, especially in CPI-naïve MEL. Investigations in additional indications, including NSCLC and RCC are ongoing.

**Trial Registration**

NCT02718066

**Ethics Approval**

The study was approved by participating study site's Institutional Review Board, and sponsor is in full compliance with all GCP and FDA regulations

#### P347 Targeting phosphatidylserine enhances the anti-tumor response to radiation therapy and immune checkpoint blockade in a preclinical melanoma model

##### Sadna Budhu, PhD, Rachel Giese, MD, Aditi Gupta, BA, Sara Schad, BS, Olivier De Henau, MD, Roberta Zappasodi, PhD, Luis Campesato, PhD, Christopher Barker, MD, Jedd D. Wolchok, MD, PhD, Taha Merghoub, PhD

###### Memorial Sloan-Kettering Cancer Center, New York, NY, USA

####### **Correspondence:** Jedd D. Wolchok (wolchokj@mskcc.org)

**Background**

Phosphatidylserine (PS) is a phospholipid that is exposed on surface of apoptotic cells, viable tumor cells, and activated immune cells. It has been shown to promote immunosuppressive signals in the tumor microenvironment. In a mouse B16 melanoma model, targeting PS in combination with immune checkpoint blockade promoted greater anti-tumor activity than either agent alone. This combination was shown to enhance T cell infiltration and activation in the tumors of treated animals. Radiation therapy (RT) is an effective focal treatment of primary solid tumors but is less effective in treating metastatic solid tumors as a monotherapy. RT induces immunogenic tumor cell death and enhances tumor-specific T cell infiltration in treated tumors.

**Methods**

8-10 mice/group were injected intradermally on the right hind limb with 100,000 B16F10 melanoma cells. 7-10 days after implantation, tumors were treated locally with a single dose of 15Gy RT or 3-doses of 8Gy or 15Gy given every 2 days. 1 day after RT, mice were given antibodies to PS (mch1N11) and PD-1 (RMP 1-14) intraperitoneally every 3 days. Tumor surface area and overall survival of mice were used to determine efficacy of the combinations. For FACS analysis, tissues were collected between 1-10 days after RT in all treated groups.

**Results**

Local RT of B16 melanoma causes an increase in PS expression on the surface of viable tumor and immune infiltrates. Treatment of animals with an antibody that targets PS (mch1N11) synergizes with RT to improve anti- tumor activity and overall survival. The triple combination of mch1N11, RT and anti-PD-1 treatment displayed greater anti-tumor and survival benefit. We found an increase in proinflammatory M1-macrophages after treatment with RT and mch1N11. There was also an increase in CD8 T cell activation in the triple combination. We found that treatment of mice whose tumors are refractory to anti-PD-1 benefited from the combination RT and mch1N11 in both reduction of tumor burden and overall survival. Finally, we found an increase of PS expression on immune cells in the blood of melanoma patients 4-7 days post RT supporting the rationale for translating of this therapy into the clinic.

**Conclusions**

This finding highlights the potential of combining these agents to improve outcome in patients that are refractory to anti-PD-1 and may inform the design of future clinical trials with the human PS targeting antibody (Bavituximab) in multiple cancers.

#### P348 Survival and immune modulation in homologous recombination deficient murine ovarian tumors using the PARP inhibitor, rucaparib and immune agonist, NKTR-214

##### Deborah Charych, PhD^1^, Liliane Robillard, PhD^2^, Andrew Simmons, PhD^2^, Thomas Harding, PhD^2^, Minh Nguyen^2^, Rachel Dusek, PhD^2^

###### ^1^Nektar Therapeutics, San Francisco, CA, USA; ^2^Clovis Oncology, San Francisco, CA, USA

####### **Correspondence:** Deborah Charych (dcharych@nektar.com)

**Background**

Ovarian cancer is the most lethal cancer of the female reproductive system and its treatment remains an unmet medical need.[1] One major advance is the use of poly(ADP-ribose)polymerase (PARP) inhibitors, now approved for both treatment and maintenance-treatment of recurrent ovarian cancer. Although ovarian cancer has low response to checkpoint inhibitors, IL2 has shown clinical promise in platinum-resistant and refractory disease.[2] The IL2-pathway agonist NKTR-214 activates and mobilizes CD8T and NK cells to tumor in human and mouse via the IL2Rβγ complex. The unique mechanistic combination of synthetic lethality (rucaparib) plus lymphocytic immune activation (NKTR-214) may enhance durable responses.

**Methods**

Two ovarian lines with genetic alterations frequent to human high grade serous ovarian cancer were used: ID8-BRCA2-/- (ID8) orthotopic primary tumors form direct contact with murine ovarian stroma, secondary carcinomatosis and extensive ascites with disease progression similar to human and BR5FVB1 (BR5) with p53-/-, BRCA1-/-, myc and Akt. Tumor cells were implanted orthotopically into immune competent mice (n=10/group) by intraperitoneal injection (ID8) or subcutaneously (BR5) and monitored respectively for frank ascites/survival or growth inhibition of established (125 mm3) tumors. Mice were treated with rucaparib (150mg/kg BID×28 days), NKTR-214 (0.8mg/kg Q9D×3), or combination. A triplet was included in ID8 (rucaparib+NKTR-214+anti-pD1). Immune modulation was evaluated by IHC and gene expression.

**Results**

In the orthotopic ovarian survival model, treatment with rucaparib or NKTR-214 increased median survival compared to vehicle (70 and 81 days respectively versus 53 days, p<0.0005). In contrast, median survival was 101 days for rucaparib+NKTR-214 doublet. Triplet therapy rucaparib+NKTR-214+anti-PD1 did not significantly add survival, median 104 days. In BR5, treatment with NKTR-214+rucaparib suppressed tumor growth by 88.5% by day 59 with 50% tumor-free mice by day 113 compared to 0% for either single agent. An increase in infiltrating cytotoxic CD8 T and NK cells to the tumor with complementary induction of dendritic cells, neutrophils and interferon-gamma-induced chemokines was observed in tumors after rucaparib+NKTR-214.

**Conclusions**

The combination of synthetic lethality (rucaparib) with lymphocytic stimulation (NKTR-214) provided significantly increased survival and durable complete response in orthotopic and subcutaneous ovarian cancer models. These unique murine tumors demonstrated disease progression and genetic aberrations parallel to human tumorigenesis, atypical of syngeneic preclinical studies. Profiling of tumors suggested the activity of this combination is through antigen priming of infiltrating memory T cells, increased NK cell recruitment and enhanced cytotoxicity of tumor infiltrates.

**References**

1. Torre LA, Trabert B, DeSantis CE, Miller KD, Samimi G, Runowicz CD, Gaudet MM, Jemal A, Siegel RL: Ovarian cancer statistics, 2018. CA Cancer J Clin (2018).

2. Vlad AM, Budiu RA, Lenzner DE, Wang Y, Thaller JA, Colonello K, Crowley-Nowick PA, Kelley JL, Price FV, Edwards RP: A phase ii trial of intraperitoneal interleukin- 2 in patients with platinum-resistant or platinum-refractory ovarian cancer. Cancer immunology, immunotherapy (2010) 59(2):293-301.

**Ethics Approval**

The study was conducted in accordance with humane, responsible, ethical and scientifically sound use of animals at Crown Biosciences International, Santa Clara, CA (HQ) in compliance with US and Chinese governments, international regulations, and AAALAC accreditation requirements. These include coordination with IACUC for implementation of the company's animal care and use policies, programs and Standard Operating Procedures.

#### P349 Spatial distribution analysis reveals increased PD1 expression on cytotoxic T cells leading to tumor regression upon combined MEK and HDAC inhibition in spontaneous PDAC mouse model

##### Phyllis Cheung, PhD^1^, Jens T. Siveke^1^, Anna Bazarna^1^, Christian Neander^1^, Konstantinos Savvatakis^1^, Sven- Thorsten Liffers^1^, Marija Trajkovic-Arsic^1^, Aayush Gupta^2^, Alexander Herner^2^, Phyllis Cheung, PhD^1^

###### ^1^University Hospital Essen, DKFZ, Essen, Germany; ^2^Technischen Universität München, Munich, Germany

####### **Correspondence:** Jens T. Siveke (j.siveke@dkfz-heidelberg.de)

**Background**

Immunotherapy has demonstrated limited efficacy in pancreatic ductal adenocarcinoma (PDAC), which is likely due to the extensive desmoplasia comprising heterogeneous cell populations with complicated interactions. Integrated, multi-omic and multiplexed technologies are essential for understanding the cellular changes in the complex microenvironment upon therapeutic treatments. Emerging preclinical studies have demonstrated the ability of MEK and HDAC inhibitors (MEKi and HDACi) to sensitize tumor cells to immune checkpoint inhibition. Here, we evaluated the impact of MEKi alone, or in combination with HDACi, on the immune landscape of PDAC using gene expression array, multiplexed immunofluorescence imaging and spatial distribution analysis.

**Methods**

Spontaneous PDAC mouse models were treated with MEKi and/or HDACi and the tumors were profiled using Nanostring PanCancer Immune Panel and gene expression array, followed by validation using immunohistochemical (IHC) and immunofluorescent (IF) staining at protein level. Spatial distribution of immune cells and tumor cells was investigated by multiplexed IF staining and analyzed computationally for quantification, co-expression and spatial distribution.

**Results**

Nanostring analysis and GSEA demonstrated that MEKi treatment alone resulted in M2 macrophage reduction, and increase in infiltrating cytotoxic T cells and PD1 expression. Upon combination with HDACi, PD1 level was further augmented, while iNOS and cleaved caspase 3 were increased when compared to MEKi alone. Multiplexed IF staining and computational analysis showed that the augmented PD1 expression induced by combined treatment was largely found on CD8+ T cells. Notably, spatial distribution analysis showed that the tumor cells in close proximity of PD1+CD8+ cells expressed high levels of iNOS and cleaved caspase 3, and low level of proliferation marker Ki67. The findings suggest that the PD1+CD8+ cells are spatially associated with increased apoptosis and reduced proliferation in tumor cells. Indeed, better survival was observed in mice with combined treatment, although statistical significance cannot be reached due to small sample size.

**Conclusions**

Our findings provide evidence for the beneficial role of PD1 expression on CD8+ cytotoxic cells in anti-tumor responses induced by combined MEK and HDAC inhibition. Besides, the application of multiplexed imaging and spatial distribution analysis improve our understanding of cellular interactions upon treatment and therefore provide new insights into the optimization of potential therapies.

**Ethics Approval**

Animal experiments for establishment and analysis of immune-based therapeutic strategies as described above in the genetically-induced, spontaneous, immunocompetent PDAC model have been approved by the Landesamt für Natur, Umwelt und Verbraucherschutz (LANUV) Nordrhen-Westfalen.

#### P350 First-line nivolumab plus ipilimumab is associated with lower costs per responder versus sunitinib among patients with advanced renal cell carcinoma

##### Toni Choueiri^1^, Keith Betts, PhD^2^, Shuo Yang, PhD^3^, Ella Du, MESc^2^, Sumati Rao, PhD^3^, Saby George, MD, FACP^4^

###### ^1^Dana-Farber Cancer Institute, Boston, MA, USA; ^2^Analysis Group, Inc., Los Angeles, CA, USA; ^3^Bristol-Myers Squibb, Lawrence Township, NJ, USA; ^4^Roswell Park Comprehensive Cancer Center, Buffalo, NY, USA

####### **Correspondence:** Toni Choueiri (toni_choueiri@dfci.harvard.edu)

**Background**

In the CheckMate 214 (CM214) clinical trial, nivolumab plus ipilimumab (N+I) demonstrated superior objective response rates (ORRs), more durable responses, and longer overall survival compared with sunitinib (S) as first-line treatment for patients with intermediate/poor-risk advanced renal cell carcinoma (aRCC). This study compared the cost per responder (CPR) and cost per month of response (CPMR) of N+I versus S as first-line treatment from a third-party US payer perspective, to better assess the value of this immuno-oncology combination and optimize treatment decisions.

**Methods**

CPR and CPMR over 1 year and over the available trial follow-up period were calculated by dividing the total cost per patient by the ORR and mean duration of response (mDOR), respectively, over each assessment period. ORR and mDOR, as well as other clinical inputs (drug dosage, adverse event frequency, subsequent treatments, and death rates) were obtained from CM214 for each treatment arm. Costs of drug acquisition and administration, all-cause adverse events, subsequent treatment, and terminal care were included in calculating total cost per patient accrued in each assessment period from a US payer perspective. The statistical differences in CPR and CPMR between the two treatments were assessed using the delta method.

**Results**

With a median follow-up of 25.2 months in CM214, N+I had a higher ORR and mDOR in each assessment period than S, and the incremental benefit of N+I in mDOR versus S increased over time. For both assessment periods, N+I had a lower CPR and CPMR (Table 1). Over 1 year, the CPR for N+I was $55,036 lower than S (P=0.260). The difference in CPR between N+I and S increased over time, and over the available follow-up period the CPR for N+I was $126,249 lower than S (P=0.113). Considering duration of response, the CPMR for N+I was $9,099 lower (P=0.094) over 1 year and $15,973 lower (P=0.004) over the available follow-up period compared with S. The cost savings of N+I increased over time as well.

**Conclusions**

First-line N+I for aRCC was associated with lower CPR and CPMR, due in part to superior ORR and mDOR compared with S. With longer follow-up time, the clinical benefits of N+I were more pronounced compared with S, indicating that the combination immunotherapy is a more cost-effective first-line choice for intermediate/poor-risk aRCC.

**Acknowledgements**

The authors thank Matt Driver and Yichen Lu (Analysis Group, Inc.) for analytical support, and PPSI (a PAREXEL company) for editorial support, funded by Bristol-Myers Squibb.

**Trial Registration**

NCT02231749

**Table 1 (abstract P350).**
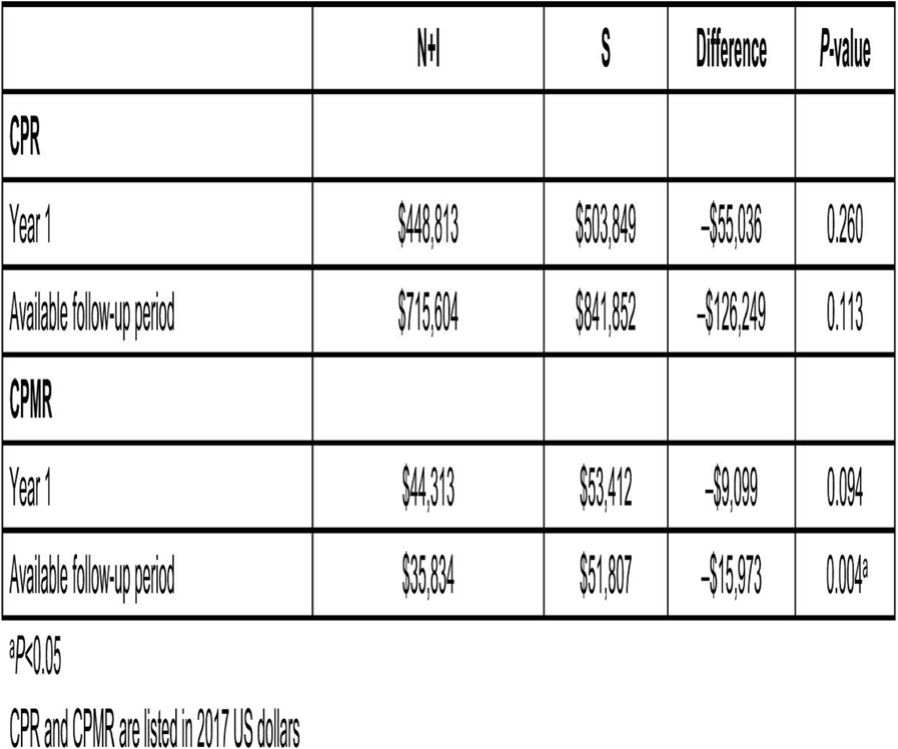
See text for description.

#### P351 ADU-S100 (MIW815) synergizes with checkpoint inhibition to elicit an anti-tumor CD8+ T cell response to control distal tumors

##### Kelsey Sivick Gauthier, PhD^1^, Anthony Desbien, PhD^1^, Gabrielle Reiner^1^, Leticia Corrales^1^, Weiwen Deng^1^, Laura Glickman, PhD^2^, Natalie Surh^1^, Brian Francica^1^, David Kanne^1^, Justin Leong, PhD^1^, Ken Metchette^1^, Lianxing Zheng^3^, Charles Cho^4^, Yan Feng^3^, Jeffrey McKenna^3^, Steve Bender, PhD^4^, Chudi Ndubaku^1^, Meredith Leong^1^, Thomas Dubensky, Jr.^5^, Andrea van Elsas^6^, Sarah McWhirter^1^

###### ^1^Aduro Biotech, Berkeley, CA, USA; ^2^Aduro Biotech, currently Actym Therapeutics, Berkeley, CA, USA; ^3^Novartis Institutes for BioMedical Research, Cambeidge, MA, USA; ^4^Genomics Institute of the Novartis Research Foundation, San Diego, CA, USA; ^5^Aduro Biotech, currently Tempest Therapeutics, Berkeley, CA, USA; ^6^Aduro Biotech Europe, Oss, Netherlands

####### **Correspondence:** Kelsey Sivick Gauthier (KGauthier@aduro.com)

**Background**

STimulator of Interferon Genes (STING) is a critical component of an innate immune pathway that activates robust anti-viral and anti-tumor responses in mouse models [1,2,3,4]. Small molecule agonists of STING are being developed as cancer immunotherapeutics due to potent anti-tumor efficacy and induction of immunity to rechallenge in pre-clinical models [5]. Activation of the STING pathway by intratumoral (IT) injection of synthetic cyclic dinucleotides (CDNs) induces type I interferons in tumor resident-myeloid subsets, activation of antigen presenting cells, expansion of tumor-specific CD8+ T cells and control of tumors [5,6,7].

**Methods**

In this study, we explored the benefit of combining CDN IT therapy with immune checkpoint blockade. ADU-S100 (MIW815), a CDN under clinical evaluation, was administered by IT injection in syngeneic mouse tumor models to assess the efficacy in combination with checkpoint inhibition.

**Results**

In mice bearing dual flank 4T1 mammary carcinoma tumors resistant to anti-PD-1 treatment, adding a single dose of ADU-S100 with anti-PD-1 induced eradication of both injected and non-injected tumors, leading to complete responses, demonstrating that ADU-S100 potentiates the activity of checkpoint blockade. Tumor control was CD8+ T cell-dependent and correlated with an enhanced CD8+ T cell effector profile in both the periphery and in non- injected tumors. Combining a single injection of ADU-S100 with anti-PD-1 also elicited enhanced tumor control in the MC-38 colon carcinoma model compared to ADU-S100 or anti-PD-1 treatment alone. Moreover, in the poorly immunogenic B16.F10 model, adding ADU-S100 to the ineffective combination therapy of anti-PD-1 and anti- CTLA-4 induced tumor-specific CD8+ T cell responses and tumor control, leading to multiple complete responses and durable immunity in surviving animals.

**Conclusions**

Together, these results highlight the immune correlates of STING-mediated anti-tumor efficacy and illustrate the potential of combining ADU-S100 (MIW815) with checkpoint inhibitors for the treatment of human cancer. Clinical trials of ADU-S100 in combination with anti-PD-1 or with anti-CTLA-4 are ongoing and could further elucidate the immunological mechanism of action and therapeutic effect in humans.

**References**

1. Diamond MS, Kinder M, Matsushita H, Mashayekhi M, Dunn GP, Archambault JM, et al. Type I interferon is selectively required by dendritic cells for immune rejection of tumors. The Journal of Experimental Medicine. 2011;208(10):1989–2003.

2. Fuertes MB, Kacha AK, Kline J, Woo S-R, Kranz DM, Murphy KM, et al. Host type I IFN signals are required for antitumor CD8 T cell responses through CD8α dendritic cells. The Journal of Experimental Medicine. 2011;208(10):2005–16.

3. Ishikawa H, Barber GN. STING is an endoplasmic reticulum adaptor that facilitates innate immune signalling. Nature. 2008;455(7213):674–8.

4. Woo S-R, Fuertes MB, Corrales L, Spranger S, Furdyna MJ, Leung MY, et al. STING-Dependent Cytosolic DNA Sensing Mediates Innate Immune Recognition of Immunogenic Tumors. Immunity. 2014;41(5):830–42

5. Corrales L, Glickman LH, McWhirter SM, Kanne DB, Sivick KE, Katibah GE, et al. Direct activation of STING in the tumor microenvironment leads to potent and systemic tumor regression and immunity. Cell Reports. 2015;11(7):1018-30.

6. Francica BJ, Ghasemzadeh A, Desbien AL, Theodros D, Sivick KE, Reiner GL, et al. TNFα and radioresistant stromal cells are essential for therapeutic efficacy of cyclic dinucleotide STING agonists in nonimmunogenic tumors. Cancer Immunology Research. 2018;6(4):422–33.

7. Corrales L, McWhirter SM, Dubensky TW, Gajewski TF. The host STING pathway at the interface of cancer and immunity. Journal of Clinical Investigation. 2016Jan;126(7):2404–11.

**Ethics Approval**

All animals were used according to protocols approved by Institutional Animal Use Committee of Aduro Biotech and maintained in specific pathogen-free conditions in a barrier facility.

#### P352 Blocking colony stimulating factor 1 receptor (CSF-1R) and tropomyosin receptor kinase A (TrkA) improves the anti-tumor efficacy of immune checkpoint blockade

##### Colm Duffy, BA, Stephen Mok, James Allison, PhD

###### The University of Texas MD Anderson Cancer Center, Houston, TX, USA

####### **Correspondence:** Colm Duffy (crduffy@mdanderson.org)

**Background**

Established tumors can escape immune responses by secreting the cytokine colony stimulating factor 1 (CSF-1), stimulating the proliferation and recruitment of immunosuppressive myeloid cells to the tumor microenvironment by binding to colony stimulating factor-1 receptor (CSF-1R). Additionally, the neurotrophin nerve growth factor (NGF) is a ligand for tropomyosin receptor kinase A (TrkA), which is over-expressed on multiple tumor types. Signaling through TrkA via the Akt and MAPK pathways regulates the survival, proliferation and invasion of tumor cells. PLX7486 is a novel orally bioavailable small molecule Trk and CSF-1R dual-inhibitor that is now being studied in Phase I clinical trials. We hypothesized that PLX7486 would synergize with immune checkpoint blockade to result in a greater antitumor effect, by targeting Trk signaling directly on cancer cells and by inhibiting the recruitment of immunosuppressive myeloid cells through CSF-1R, thus enabling an improved antitumor T cell response.

**Methods**

Various cancer cell lines were assessed by Western blot to determine whether TrkA and CSF-1 are expressed. Murine cancer cells, myeloid cells and T cells were treated with various concentrations of PLX7486 to determine effects on cell viability. Mice were subcutaneously implanted with MC38, B16F10 or MT4 cancer cells and treated with combined PLX4786 and anti-CTLA-4 or anti-PD-1 to determine the effects on tumor growth and on subpopulations of immune cells within the tumor microenvironment.

**Results**

We confirmed the expression of TrkA receptor on multiple murine cancer cell lines in vitro and exposed them to PLX7486, showing a direct cytotoxic effect with an IC50 of 5-8μM on most of the cell lines and an inhibition of AKT pathway signaling in MC38 cells. PLX7486 also had a direct cytotoxic effect on bone marrow-derived macrophages and the murine macrophage cell line RAW264.7 with an IC50 <1μM, while it had no effect on activated T cells in vitro. Combining PLX7486 and anti-CTLA-4 or anti-PD-1 in vivo in MC38, B16F10 or MT4 tumor models resulted in increased antitumor effects and a reduction in immune suppression within the tumor microenvironment.

**Conclusions**

The combined treatment groups of PLX7486 and anti-CTLA-4 or anti-PD-1 showed significant superiority in vivo in multiple tumor models. Our work provides rationale for testing this combined therapy in cancer patients.

**Acknowledgements**

We thank F. Hasan for illustration and members of the Allison lab for helpful discussions. This work was supported by a grant from Cancer Prevention and Research in Texas to J.P.A. (R1203). C.R.D. was supported by the CPRIT Research Training Grant (RP170067). S.M. is a CRI Irvington Postdoctoral Fellow. J.P.A. is a Co-Director of the Parker Institute for Cancer Immunotherapy.

**Ethics Approval**

The study was approved by MD Anderson Cancer Center’s Institutional Animal Care and Use Committee, Study # 00001221-RN01

#### P353 Strategic combination of multiple immune-oncology agents to engage, expand, and enable immune responses against tumors

##### Kellsye Fabian, PhD^1^, Michelle Padget^1^, Anthony Malamas^1^, Rika Fujii^1^, John Lee, MD^2^, Jeffrey Schlom, PhD^1^, James W. Hodge, PhD, MBA^1^

###### ^1^CCR, NCI, NIH, Bethesda, MD, USA; ^2^NantKwest, Culver City, CA, USA

####### **Correspondence:** James W. Hodge (jh241d@nih.gov)

**Background**

We hypothesize that in order for immunotherapy mount an effective and sustainable response against tumors, multiple levels of immune cell-tumor interaction must be interrupted. Therefore, optimal therapy of established tumors would require multiple agents that would 1) engage the immune response and generate tumor specific effector cells; 2) expand the number and breadth of the immune effector cells; and 3) enable the anti-tumor activity of these immune cells in the tumor microenvironment.

**Methods**

4T1-bearing Balb/c mice and MC32a-bearing C57BL/6 mice were treated with vaccine (adenovirus-Twist or adenovirus-CEA), cytokine (IL-15 superagonist), antibody agonist for co-stimulatory receptors (anti-OX40 and anti- 4-1BB), immune checkpoint inhibitor (anti-PD-L1), and chemotherapy (docetaxel). Primary and metastatic tumor growth inhibition and generation of anti-tumor immune effector cells were used as primary efficacy endpoints.

**Results**

Administration of 1-2 agents had no antitumor activity while the combination of 3-5 agents had modest antitumor effects. On the other hand, the concurrent treatment with all six agents (hexatherapy) was able to significantly induce anti-tumor responses and reduce tumor burden. Hexatherapy modified the tumor immune landscape by favoring effector T cells and limiting the immunosuppressive cell populations, thus improving the CD4+ T cell:Treg and CD8+:Treg ratios. Furthermore, tumor infiltrating T cells in the mouse cohort that received hexatherapy have higher proliferative capacity (Ki67+) and have significantly less exhausted phenotype showing less PD-1 and CTLA-4- expression.

**Conclusions**

These data demonstrate that strategic combination of multiple immune-oncology agents that can engage, expand, and enable the immune response is imperative for optimal anti-tumor therapy.

**Ethics Approval**

The study was approved by the NCI ACUC, protocol number LTIB-30.

#### P354 Co-clinical trials of MEK inhibitor, anti PD-L1 and anti CTLA-4 combination treatment in Non-Small Cell Lung Cancer

##### Pierre-Olivier Gaudreau, David Peng, PhD, Bertha Leticia Rodriguez, Jared Fradette, Laura Gibson, Samrat Kundu, PhD, Limo Chen, PhD, Jennifer Wargo, MD, MMSc, Don Gibbons, MD, PhD

###### MD Anderson Cancer Center, Houston, USA

####### **Correspondence:** Pierre-Olivier Gaudreau (PGaudreau@mdanderson.org)

**Background**

Immunotherapies involving the PD-1 / PD-L1 axis have revolutionized the treatment of non-small cell lung cancer (NSCLC), but novel combination therapies are needed to improve the overall response rate. Current work by our group using mutant KRAS and TP53 (KP) mouse models of NSCLC have shown that rationally designed therapies combining PD-L1 immune checkpoint blockade with MEK inhibitors significantly decrease tumor growth and metastasis compared to either monotherapies in syngeneic KP mice tumors. Despite these encouraging results, therapeutic resistance still occurs. Reverse Phase Protein Array (RPPA) and FACS analyses from these tumors showed an increase in Tregs and CTLA-4 immune checkpoint expression. As anti CTLA-4 checkpoint blockade is particularly effective in increasing the CD8 / Treg lymphocyte ratio [1], we hypothesized that the addition of this agent as a novel triple combination therapeutic strategy may improve the outcome by depleting Tregs and neutralizing CTLA-4 expression.

**Methods**

Using in vivo KP subcutaneous tumors (sv129 genetic background), we compared the triple combination of the MEK inhibitor selumetinib, anti PD-L1 and anti CTLA-4 or IgG2b isotype control antibodies. Tumor sizes were assessed weekly with digital calipers, and tumor weights and lung metastasis were quantified visually at the end of treatment following mice euthanasia. Fresh cells were characterized by FACS to establish the tumor-infiltrating immune cell profile. Tumor specimens were processed for RPPA and custom codeset Nanostring analyses for further mechanistic insights. In an upcoming single center, Phase I / II clinical trial, two combination schedules of selumetinib, tremelimumab and durvalumab will be compared with historical controls in patients (n = 40) with previously treated, metastatic NSCLC. The primary objective is to assess progression-free survival, and secondary objectives include further clinical outcomes and markers of response and resistance in pre- and on- treatment biopsies.

**Results**

The addition of anti CTLA-4 to anti PD-L1 and MEK inhibitor treatment improved survival in the epithelial KP mouse model (log-rank test, p=0.0078) (Figure 1). Animal trials using the mesenchymal KP model, along with correlative analyses (i.e., FACS, RPPA, Nanostring) for both models, are currently underway. The Phase I / II clinical protocol is undergoing regulatory review.

**Conclusions**

The combination of a MEK inhibitor, anti PD-L1 and anti CTLA-4 improves survival in epithelial KP tumor models of NSCLC. Correlative analyses to gain mechanistic insights of efficacy are ongoing. Accrual for the Phase I / II clinical trial is expected to start in early 2019.

**Acknowledgements**

This work is supported by an operating grant to D.L. Gibbons from the NIH/NCI (“The Role of Epithelial-Mesenchymal Transition in Re-Wiring KRAS Mutant Lung Cancer”; grant number: 1R37CA214609-01A1). P.O. Gaudreau is supported by the Fonds de Recherche Québec–Santé’s (FRQS) Resident Physician Health Research Career Training Program program (grant number: 32667).

**Trial Registration**

ClinicalTrials.gov identifier: NCT03581487MD Anderson Cancer Center trial number: 2017-0888

**References**

1. Wei SC, Levine JH, Cogdill AP et al. Distinct Cellular Mechanisms Underlie Anti-CTLA-4 and Anti-PD-1 Checkpoint Blockade. Cell 2017; 170: 1120-1133.

**Ethics Approval**

The clinical trial discussed in the abstract was approved by the MD Anderson Cancer Center IRB (protocol 2017-0888).

**Fig. 1 (abstract P354).**
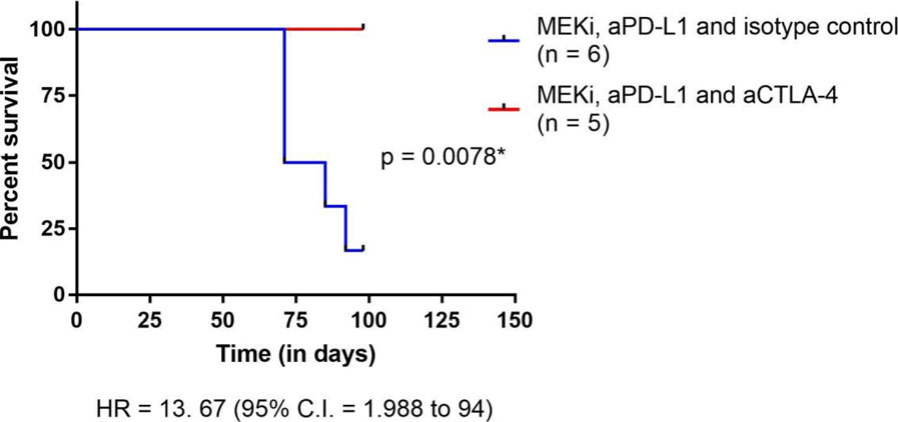
See text for description.

#### P355 Nivolumab plus ipilimumab is associated with lower number needed to treat compared with sunitinib for preventing death in advanced renal cell carcinoma

##### Saby George, MD, FACP^1^, Keith Betts, PhD^2^, Shuo Yang, PhD^3^, Ella Du, MESc^2^, Jennifer Johansen, PharmD, BCPS^3^, Sumati Rao, PhD^3^, Toni Choueiri^4^

###### ^1^Roswell Park Comprehensive Cancer Center, Buffalo, NY, USA; ^2^Analysis Group, Los Angeles, CA, USA; ^3^Bristol-Myers Squibb, Lawrence Township, NJ, USA; ^4^Dana-Farber Cancer Institute, Boston, MA, USA

####### **Correspondence:** Saby George (saby.george@roswellpark.org)

**Background**

Nivolumab plus ipilimumab (N+I) demonstrated superior efficacy and safety outcomes compared with sunitinib (S) as first-line treatment of intermediate/poor-risk advanced or metastatic renal cell carcinoma (aRCC) in the CheckMate 214 trial. To further quantify the clinical benefits and risks associated with these two treatments, this study estimated the number needed to treat (NNT) and number needed to harm (NNH) for N+I versus S in previously untreated intermediate/poor-risk aRCC.

**Methods**

For patients with intermediate/poor-risk aRCC in CheckMate 214, the rates of objective response (ORR), overall survival (OS), and grade 3/4 adverse events (AEs) over 12 and 24 months were calculated using patient-level data. The NNTs were calculated for ORR and OS as the inverse of the absolute risk reduction between N+I and S over 12 and 24 months among all randomized patients (N+I, 425; S, 422). Similarly, the NNHs were calculated as the reciprocals of the absolute risk differences between the two treatments for treatment-related and all-cause grade 3/4 AEs among all treated patients (N+I, 423; S, 416).

**Results**

The NNT and NNH analyses showed consistent benefits of N+I over S in clinical efficacy and safety. At month 12, one death would be prevented if 12.50 (95% confidence interval [CI], 7.30-43.39) patients were treated with N+I instead of S (Table). At month 24, the NNT to prevent one death with N+I versus S was reduced to 8.18 (95% CI, 5.20-19.23). When ORR was assessed, the NNT to achieve one additional responder with N+I versus S was 6.32 (95% CI, 4.53-10.48) at month 12 and 6.62 (95% CI, 4.67-11.36) at month 24. When safety was evaluated, for every 4.32 (95% CI, 3.24-6.47) patients treated with S instead of N+I, one additional patient would have experienced a treatment-related grade 3/4 AE over 12 months (Table). The NNH for an all-cause grade 3/4 AE over 12 months was 6.02 (95% CI, 4.37-9.64) and remained similar over 24 months.

**Conclusions**

The NNT/NNH analysis showed that among previously untreated patients with intermediate/poor-risk aRCC, N+I provides greater clinical benefits and is associated with significantly lower risks of grade 3/4 AEs compared with S. The survival benefit of N+I over S was even more pronounced when a longer follow-up period was evaluated, indicated by the reduction in NNT from month 12 to month 24. Future analyses based on longer-term data are warranted to quantify the benefits and risks associated with N+I beyond 2 years.

**Acknowledgements**

The authors thank Matt Driver and Lei Yin from Analysis Group for analytical support. Editorial support was provided by PPSI (a PAREXEL company), funded by Bristol-Myers Squibb.

**Trial Registration**

NCT02231749

**Table 1 (abstract P355).**
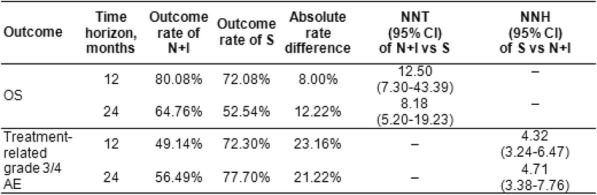
See text for description.

#### P356 CXCR4 antagonism converts the cold tumor into hot tumor by enhancing the infiltration of antigen-specific CD8 T-cells in the TME leading to increased anti-tumor effects of anti-OX40

##### Pankaj Gaur, PhD^1^, Vivek Verma^1^, Rahul Nandre, PhD^1^, Ella Sorani, PhD^2^, Abi Vainstein-Haras, MD^2^, Galia Oberkovitz^2^, Amnon Peled, PhD^3^, Seema Gupta, PhD^1^, Samir Khleif, MD^1^, Stephen Shaw, PhD^4^, Osnat Bohana- Kashtan^4^

###### ^1^Georgetown University Medical Center, Washington, DC, USA; ^2^BiolineRx Ltd., Modi'in, Israel; ^3^Hadassah Hebrew University Hospital, Jerusalem, Israel; ^4^Bioline Rx, Sandwich, UK

####### **Correspondence:** Samir Khleif (snk48@georgetown.edu)

**Background**

CXCR4 helps to retain the hematopoietic stem cells (HSC) in the bone marrow (BM). CXCR4 binds to its ligand CXCL12/SDF1 which is constitutively expressed in the BM thereby inhibiting the mobilization of CXCR4 expressing immune progenitor cells. Moreover, increased numbers of effector cells in the tumor microenvironment (TME) are directly correlated to enhanced immunotherapeutic efficacy. Therefore, CXCR4 antagonism will result in movement of immune cells to the periphery, increasing the infiltration of effector cells into the TME. Furthermore, signaling through OX40 is known to enhance the effector functions of CD8 T-cells and also generate immune memory. However, as a single agent anti-OX40 has not yet shown promising results in the clinic. Therefore, we hypothesized that combining anti-OX40 with CXCR4-antagonist will enhance the functionality of tumor-infiltrated effector cells as well as produce durable anti-tumor responses by augmenting immune memory.

**Methods**

To assess the anti-tumor response of CXCR4 in combination with anti-OX40, tumor-bearing mice were treated with anti-OX40 antibody (1 mg/kg twice weekly) and/or CXCR4 antagonist (BL-8040; 4 doses; 24 h apart; 20 mg/kg) in the presence of tumor-specific antigen priming (E7-peptide; 3 doses/ one week apart). Tumor growth and mice survival were recorded. Anti-tumor immune responses were determined in the tumors obtained 3-4 days after the second vaccination using flow cytometry.

**Results**

We found that BL-8040 given with specific antigenic stimulation results in increased anti-tumor immune response leading to significant decrease in tumor growth (p≤0.001 at day 21) and prolonged mice survival. BL-8040 significantly increases the numbers of total and antigen-specific CD8+ T-cells in the TME. Furthermore, in accordance with our hypothesis combination of CXCR4-antagonist and anti-OX40 treatment resulted in an increase in the functionality of tumor-infiltrated effector cells as determined by the numbers of total and antigen-specific granzyme B+ and IFN-gamma+ CD8+ T-cells, leading to significant delay in tumor growth and prolonged mice survival. In addition, this combination increased the central memory (CD62+ CD44+) in the TME, enhancing the durability of anti-tumor immune response.

**Conclusions**

Based on these findings, we conclude that CXCR4 antagonism converts cold tumors into hot tumors by facilitating the infiltration of tumor-specific effector cells into the TME. Further, anti-OX40 enhances the anti-tumor immune response by augmenting the effector functions of the tumor-infiltrated CD8+ T-cells and maintaining the durability of response by generation of immune memory. Hence, we demonstrate that the combination of agonist anti-OX40 with CXCR4-antagonist is a promising immunomodulatory strategy for cancer immunotherapy.

#### P357 Entinostat increases the frequency of tumor-specific effector T-cells and their functionality is enhanced by anti-OX40 leading to durable anti-tumor effects

##### Rahul Nandre, PhD^1^, Vivek Verma^1^, Pankaj Gaur, PhD^1^, Hua Wang^2^, Peter Ordentlich, PhD^3^, Lei Wang, PhD^3^, Seema Gupta, PhD^1^, Samir Khleif, MD^1^

###### ^1^Georgetown University Medical Center, Washington, DC, USA; ^2^Augusta University, Augusta, GA, USA; ^3^Syndax Pharmaceuticals, Waltham, MA, USA

####### **Correspondence:** Samir Khleif (snk48@georgetown.edu)

**Background**

The epigenetic deregulation of T-cells and enhanced numbers of immunosuppressive cells in the TME are associated with decreased anti-tumor effects. Hence, targeting the epigenetic modifications using modulators such as histone- deacetylase inhibitors (HDACi) provides the basis for a potential role for these agents in cancer immunotherapy. Entinostat, an HDACi has been shown to reprogram the TME by impacting the numbers of CD8T-cells and immunosuppressive cells, resulting in enhanced anti-tumor activity when combined with immune-checkpoint blockade [1]. However, the combination effect of Entinostat with anti-OX40 remains poorly explored. Signaling through OX40 is known to enhance the effector functions of CD8T-cells. However, as a single agent anti-OX40 has not yet shown promising results in the clinic. Therefore, we hypothesized that the combination of Entinostat with anti-OX40 will enhance the effector-functions of CD8T-cells while simultaneously reducing the immunosuppressive cells in the TME, leading to improved anti-tumor effects.

**Methods**

In TC-1 mouse tumor model, Entinostat (3mg/kg) in combination with anti-OX40 (1mg/kg) and tumor-specific vaccine (E7-peptide; 3 doses one-week apart) was given. Tumor growth and mice survival were recorded. Three days after the second immunization, immune-responses were determined in the tumors.

**Results**

We show that Entinostat significantly increases the numbers of tumor-infiltrated CD8 and CD4 cells and reduces the frequency of immunosuppressive Tregs and MDSCs leading to a significant delay in tumor growth. However, none of the mice show complete tumor rejection and ultimately succumb to tumor burden. Importantly, Entinostat synergizes with anti-OX40, resulting in complete tumor regression in 100% of the mice that remain tumor free for rest of their lives. This effect is found to be associated with enhanced total and antigen-specific granzymeB+ and IFN-gamma+ CD8T-cells in the TME. In addition, anti-OX40 further reduces the Tregs and MDSCs in the TME. To test the durability of the anti-tumor response induced by Entinostat+anti-OX40, the mice with complete tumor rejection were re-challenged with the tumor. Although, the tumors grew avidly in untreated animals, none of the treated mice showed tumor development, clearly establishing a durable anti-tumor response after combination treatment.

**Conclusions**

These results highlight the ability of Entinostat in increasing the numbers of effector T-cells in the TME. Anti-OX40 significantly enhanced the functionality of these tumor-infiltrated effector cells leading to induction of robust and durable anti-tumor responses. Importantly, anti-OX40 further decreased the numbers of immunosuppressive populations in the TME. These data highlight that Entinostat enhances the anti-tumor efficacy of anti-OX40, which can be a promising strategy for cancer-immunotherapy.

**References**

1. Terranova-Barberio M, et al. HDAC inhibition potentiates immunotherapy in triple negative breast cancer. Oncotarget. 2017; 8:114156-114172.

#### P358 ROR-gamma agonist induces long-lived Th17 cells in the TME leading to increased anti-tumor effects of agonist anti-OX40

##### Pankaj Gaur, PhD^1^, Vivek Verma^1^, Rahul Nandre, PhD^1^, Laura Carter, PhD^2^, Xiao Hu, PhD^2^, Xikui Liu, PhD^2^, Seema Gupta, PhD^1^, Samir Khleif, MD^1^

###### ^1^Georgetown University Medical Center, Washington, DC, USA; ^2^Lycera Corp, Ann Arbor, MI, USA

####### **Correspondence:** Samir Khleif (snk48@georgetown.edu)

**Background**

T-cell costimulation through OX40 has been shown to promote expansion and proliferation of effector T-cells leading to enhanced effector functions, memory generation and immune inflammatory anti-tumor responses. However, treatment with anti-OX40 as a single agent has not led to major positive clinical outcomes. In preclinical models, we have recently shown that combining anti-PD-1 concurrently with anti-OX40 negates the effects of agonist anti-OX40 making identification of combination partners crucial for anti-tumor therapy. ROR-gamma-t, a master transcription factor is known to drive Type 17 T-cell differentiation. Synthetic, small molecule ROR-gamma agonists have been shown to enhance Type 17 T-cell effector functions and survival, decrease immune suppressive mechanisms and modulate expression of a number of costimulatory and coinhibitory molecules. We hypothesized that ROR-gamma agonist could enhance the anti-tumor effects of anti-OX40.

**Methods**

We tested using TC-1, a mouse tumor model where vaccine is used to prime the immune system and assessed the effects of agonist anti-OX40 antibody (1 mg/kg twice weekly) combined with a ROR-gamma agonist (LYC-54143; 100 mg/kg BID given continuously till the end of study) on growth of established tumors and survival.

**Results**

We found that the ROR-gamma agonist significantly delayed tumor growth and prolonged mice survival, which was by induction of Th17 cells in the TME. Analysis of the tumor microenvironment revealed that the functionality of ROR-gamma induced Th17 cells was significantly enhanced upon anti-OX40 treatment. Moreover, this treatment increased the numbers of total CD4+ T-cells including ROR-gamma-t+ and highly activated INF-gamma+ cells and decreased the Treg numbers. Furthermore, we found that ROR-gamma agonist+anti-OX40 resulted in an increase in the numbers of total and antigen-specific, granzyme B+, and IFN-gamma+ CD8+ T-cells as well as increased the central memory (CD62+ CD44+) in the TME.

**Conclusions**

ROR-gamma agonist enhances the anti-tumor effects of anti-OX40 leading to reduced tumor growth and prolonged mouse survival. These anti-tumor effects are mediated by generation of activated antigen-specific CD8+ and IFN- gamma+ Th17 cells with simultaneous decrease in the numbers of Tregs in the TME.

#### P359 Immune cell responses and dose scheduling optimization of radiation and anti-PD-L1 treatment combination using a joint experimental and systems modeling approach

##### Gabriel Helmlinger, PhD^1^, Yuri Kosinsky, PhD^2^, Simon Dovedi, PhD^3^, Kirill Peskov, PhD^2^, Veronika Voronova, MSc^2^, Lulu Chu, PhD^1^, Donald Stanski, MD^1^

###### ^1^AstraZeneca, Waltham, MA, USA; ^2^M&S-Decisions LLC, Moscow, Russian Federation; ^3^MedImmune, Manchester, UK

####### **Correspondence:** Gabriel Helmlinger (gabriel.helmlinger@astrazeneca.com)

**Background**

We explored the mechanistic interactions between radiation therapy (RT) and the host immune system using a quantitative systems modeling approach to investigate how distinct variables may impact the immunogenicity of RT+ immuno-oncology (IO) combination therapies. In addition to its cytoreductive effect RT also modulates the tumor microenvironment (TME) to facilitate an anti-tumor immune response. Combination therapies of RT + anti-PD-L1 mAb blockade have indeed shown synergy in a number of preclinical studies [1,2]. In the PACIFIC clinical trial, Imfinzi, an anti-PD-L1 mAb, was effective as a consolidation therapy following platinum-based chemoradiotherapy, with statistically and clinical significant improvements in OS over placebo [3,4].

**Methods**

Based on data from [1], we developed an externally validated mechanistic population model of the origination and development of an anti-tumor T cell immune response linked to CT26 tumor size dynamics, following treatment with anti-PD-L1 mAb therapy alone and in combination with RT in a mouse model (Fig 1A). Variability in individual tumor size dynamics was taken into account using a mixed-effects model at the level of tumor infiltrating T cell influx.

**Results**

Upon external validation, the model was used prospectively to predict anti-tumor efficacy in a broad range of therapeutically-realistic RT and anti-PD-L1 mono- and combination dosing schedules. In full agreement with [1], scheduling of an anti–PD-L1 mAb with concomitant administration of RT – and not preceding RT - was required to maximize efficacy benefits (Fig 1B). The model also highlighted a pivotal role for the immune response in RT- induced tumor shrinkage: RT may indeed accelerate the development of an immune response by improving tumor antigen presentation, thereby inhibiting tumor growth and delaying the accumulation of immuno-suppressive regulatory T cells (Treg) as a result. The model accounted for the adaptive expression of PD-L1 in response to inflammatory changes in the local TME as a negative feedback which could be overcome by blockade of this axis. Combinations of RT and anti-PD-L1 treatments may offset the immuno-suppressive impact of Treg and PD-L1 expression over time, thereby inducing a sufficiently robust accumulation of cytotoxic T cells with subsequent tumor shrinkage or rejection.

**Conclusions**

This modeling study provides quantitative mechanistic insights into the links between RT and anti-tumor immune responses. The model may be used to determine appropriate combinations and schedules of immuno-modulation and RT to maximise the therapeutic potential of RT/IO combination therapy.

**References**

1. Dovedi S, et al. Acquired resistance to fractionated radiotherapy can be overcome by concurrent PD-L1 blockade. Cancer Res. 2014;74:5458-5468.

2. Deng L, et al. Irradiation and anti–PD-L1 treatment synergistically promote antitumor immunity in mice. J Clin Invest. 2014;124:687–695.

3. Antonia S, et al. Durvalumab after chemoradiotherapy in stage III non-small-cell lung cancer. N Engl J Med. 2017;377:1919-1929.

4. https://www.astrazeneca.com/media-centre/press-releases/2018/imfinzi-significantly-improves-overall-survival-in-the-phase-iii-pacific-trial-for-unresectable-stage-iii-non-small-cell-lung-cancer-25052018.html

**Fig. 1 (abstract P359).**
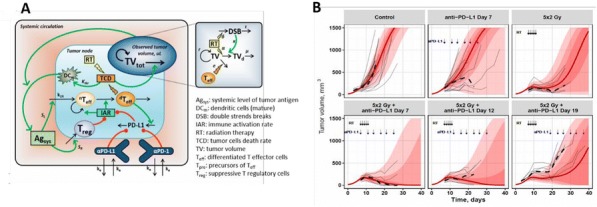
See text for description.

#### P360 Enhanced anti-tumor efficacy of mesothelin-targeted immunotoxin LMB-100 combined with anti-PD-1 antibody

##### Qun Jiang, PhD, Daniel Rathkey, Jingli Zhang, Ira Pastan, MD, Raffit Hassan, MD

###### National Institutes of Health, Bethesda, MD, USA

####### **Correspondence:** Raffit Hassan (hassanr@mail.nih.gov)

**Background**

LMB-100 is a recombinant immunotoxin, currently in phase I clinical trials that targets mesothelin (MSLN) a cell surface protein highly expressed in mesothelioma and lung adenocarcinoma. Given the high expression of PD-L1 in mesothelioma and lung cancer and promising clinical activity of PD-1/PD-L1 checkpoint inhibitors in these cancers, we aimed to evaluate if LMB-100 in combination with αPD-1 antibody will result in greater anti-tumor efficacy.

**Methods**

We established a human MSLN expressing syngeneic mouse model using PD-L1 positive mouse lung adenocarcinoma cell line 531LN2 stably transfected with a vector encoding hMSLN. Mice bearing 531LN2-hMSLN subcutaneous tumors were given no treatment, or intravenous LMB-100 alone, or intraperitoneal αPD-1 antibody alone, or LMB-100 plus αPD-1 antibody. Tumor growth and overall survival was analyzed. Using NanoString gene expression assay, we analyzed cancer associated immune gene expression induced by the drug administration. We then depleted CD8+ T cells using αCD8 antibody to identify its role in the drug induced anti-tumor effects. Finally, we evaluated LMB-100/αPD-1 combination in a mesothelin and PD-L1 positive patient derived mesothelioma RH63 humanized mouse model transplanted with healthy donor PBMCs.

**Results**

In mice bearing 531LN2-hMSLN tumors, tumor growth was significantly inhibited by LMB-100/αPD-1 treatment compared to mice treated with either drug alone. The median tumor volumes were 865mm3, 420mm3, 277mm3, and 65mm3 in untreated, LMB-100 treated, αPD-1 treated, and combination treated groups respectively on day 34 post tumor inoculation (p<0.001). The median overall survival was 38 days without treatment, 52 days with either LMB- 100 or PD-1 antibody alone, and 74 days with the combination (p<0.05). We observed a significant increase of CD8+ T cells and Th1 cytokine signaling gene expression in tumors treated with LMB-100 /αPD-1 compared to either agent alone. After depletion of CD8+T cells, the anti-tumor benefits were significantly negated in LMB- 100/αPD-1treated mice, suggesting their important roles. Furthermore, we observed similar LMB-100/αPD-1 combination-enhanced anti-tumor efficacy in healthy donor PBMCs transplanted mesothelioma RH63 humanized mouse model. The median tumor volumes were 350mm3, 306mm3, 248mm3, 216mm3and 135mm3 in untreated, PBMCs transplanted, LMB-100 treated, PBMCs plus αPD-1 treated, and PBMCs plus LMB-100/αPD-1 combination treated groups respectively on day 41 post tumor inoculation (p<0.001).

**Conclusions**

Our study demonstrates that LMB-100/αPD-1 antibody combination enhances CD8 T cells mediated anti-tumor efficacy in hMSLN expressing syngeneic lung cancer mouse model and humanized mesothelioma mouse model. Combination treatment with immune checkpoints and LMB-100 could be useful to treat patients with mesothelin positive cancers.

**Acknowledgements**

We thank Dr. Jonathan M. Kurie from MD Anderson Cancer Center for providing us 531LN2 cell line as a gift.

#### P361 Phase 1/2 study of mogamulizumab (anti-CCR4), to deplete regulatory T-cells, in combination with PD-1 checkpoint inhibitor nivolumab in subjects with locally advanced or metastatic solid tumors

##### David Hong, MD^2^, Olivier Rixe, MD, PhD^3^, Vi Chiu, MD, PhD^3^, Patrick Forde, MD^4^, Tomislav Dragovich, MD^5^, Yanyan Lou, MD^6^, Asha Nayak, MD^7^, Rom Leidner, MD^8^, James Atkins, MD^9^, Agron Collaku, PhD^1^, Floyd Fox, PhD^1^, Margaret Marshall, MD^1^, Anthony Olszanski, MD, RPh^10^

###### ^1^Kyowa Kirin Pharmaceutical Development, Inc., Princeton, NJ, USA; ^2^MD Anderson Cancer Center, Houston, TX, USA; ^3^University of New Mexico Comprehensive Cancer Center, Albuquerque, NM, USA; ^4^Sidney Kimmel Comprehensive Cancer Center, Johns Hopkins University School of Medicine, Baltimore, MD, USA; ^5^Banner MD Anderson Cancer Center, Gilbert, AZ, USA; ^6^Mayo Clinic Cancer Center, Jacksonville, FL, USA; ^7^Georgia Cancer Center, MARTINEZ, GA, USA; ^8^Providence Cancer Center, Portland, OR, USA; ^9^Southeastern Medical Oncology Center, Goldsboro, NC, USA; ^10^Fox Chase Cancer Center, Phildelphia, PA, USA

####### **Correspondence:** David Hong (dshong@mdanderson.org)

**Background**

Tumors induce an immunosuppressive environment by recruiting regulatory T-cells (Tregs) that inhibit immune antitumor activity and T-cell activation via cell cycle checkpoints. Mogamulizumab (Moga), an antibody targeting anti-CC-chemokine receptor 4 (CCR4), eliminates CCR4+ cells in T cell malignancies, depletes a subset of high expressing CCR4+ Tregs, and is approved to treat CCR4+ T cell lymphomas in Japan. Nivolumab (Nivo), an antibody targeting the programmed cell death-1 (PD-1) checkpoint, is approved to treat several solid and hematologic tumors. We hypothesized that simultaneously blocking two suppressive pathways by combining Moga and Nivo may enhance antitumor activity.

**Methods**

We conducted a Phase 1 dose finding study in the U.S. to identify the maximum tolerated dose (MTD) for Moga+Nivo combination, and a Phase 2 expansion using the MTD regimen in tumor-specific cohorts. Subjects were excluded if previously treated with any drug targeting T cell stimulation or checkpoint pathways. The primary objective was to assess safety and tolerability. The secondary objectives were to evaluate antitumor activity based on overall response rate (ORR), time to response (TTR), duration of response, progression-free survival (PFS), and overall survival (OS). Exploratory objectives included examination of biomarkers by flow cytometry and immunohistochemistry

**Results**

A total of 114 subjects were enrolled and treated: n=4 in a single Phase 1 cohort and n=110 in 7 tumor-specific Phase 2 cohorts (see Table 1). There were no dose-limiting toxicities in Phase 1, and all Phase 2 subjects received the planned dose of 1 mg/kg Moga + 240 mg Nivo. Treatment emergent adverse event rates for all cancer types are shown in Table 2 (Table 2). There were 2 complete responses (both in subjects with ovarian cancer) and 10 partial responses, for an ORR (Table 1) of 10.5% (95% CI, 5.6, 17.7) and a median TTR of 2.34 months. For all subjects, median PFS was 2.6 months (95% CI, 2.3, 3.7) and median OS was 9.5 months (95% CI, 6.0, 13.8). The Moga+Nivo combination depleted effector Treg immunosuppressive populations in peripheral blood and within tumor stroma; however, this expected depletion was not correlated with treatment response.

**Conclusions**

Moga+Nivo combination therapy in multiple solid tumors demonstrated an expected safety profile and some antitumor activity. There was no additive antitumor effect seen with the combination of the two agents in this study. Further study may elucidate the effects of Moga on effector T cells.

**Trial Registration**

NCT02705105

**Ethics Approval**

This study was approved by an Institutional Review Board at each investigative site.

**Table 1 (abstract P361).**
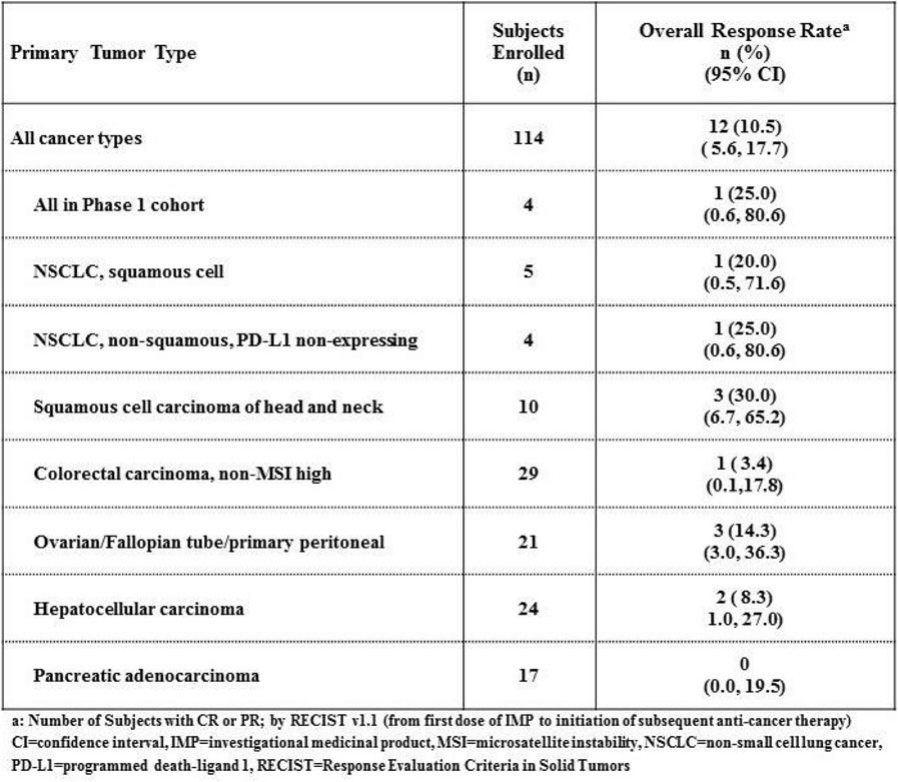
See text for description.

**Table 2 (abstract P361).**
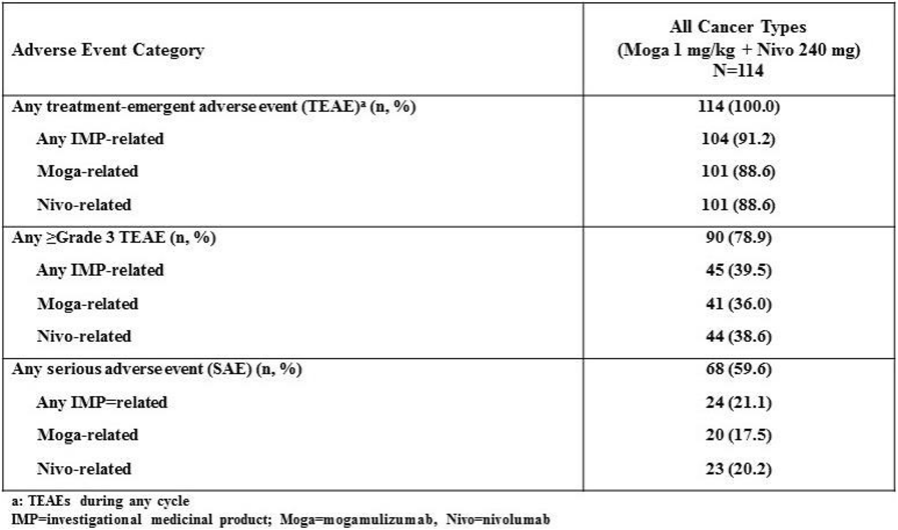
See text for description.

#### P362 TTI-621 (SIRPαFc), an immune checkpoint inhibitor blocking the CD47 “do not eat” signal, enhances the anti-tumor effect of radiation and targeted therapy in ovarian cancer models

##### Lei Cui, PhD, BS^2^, Hui Chen, BS^2^, April Lewtak, MSc^2^, Sean Oh^2^, Carole Galligan, PhD^2^, Simone Helke^2^, Julia Berchadsky, PhD^2^, Bob Uger, BS, PhD^2^, Lisa Johnson, PhD^2^

###### ^1^Trillium Therapeutics, Mississauga, Canada; ^2^Trillium Therapeutics Inc., Mississauga, Canada

####### **Correspondence:** Lisa Johnson (lisa@trilliumtherapeutics.com)

**Background**

Ovarian cancer (OVCa) is the most lethal gynecologic malignancy. With standard treatment demonstrating a high relapse rate, novel treatment strategies are needed. PARP inhibitors are approved as monotherapy agents for BRCA mutated OVCa, and function as radiosensitizers. Both radiotherapy (RT) and PARP inhibitors induce immunogenic cell death, release tumor antigens, and enhance the infiltration of immune cells, including macrophages, to tumor sites. Thus, innate checkpoint inhibition may enhance the anti-tumor effect of DNA damaging agents.CD47 is an immune checkpoint that binds signal regulatory protein alpha (SIRPα) and delivers a “do not eat” signal to suppress macrophage phagocytosis. It is frequently overexpressed by tumors to evade macrophage mediated destruction. TTI- 621 (SIRPαFc), an immune checkpoint inhibitor consisting of the CD47 binding domain of human SIRPα linked to the Fc region of IgG1, blocks the CD47 “do not eat” signal and engages macrophages Fcγ receptors, thereby enhancing phagocytosis and antitumor activity. Here we report the efficacy of the combination of TTI-621 and DNA damaging therapeutics, RT and PARP inhibition, in BRCA competent and knock-down OVCa xenografts.

**Methods**

TTI-621, RT and niraparib (a PARP inhibitor) was evaluated alone or in combination in intraperitoneal tumor xenografts of BRCA competent and knock-down luciferase-expressing OVCa cells in NOD/SCID mice. TTI-621 (10 mg/kg) was administered intraperitoneally 1 hour prior to RT, 3 times per week for 3 weeks. Niraparib (50 mg/kg) was administered 1 hour prior to RT, 5 times per week for 1 week. Mice were treated with whole abdomen RT at a dose of 2 Gy for 2 fractions. Treatment efficacy was assessed by bioluminescent imaging (BLI) and survival. Systemic toxicity was evaluated by clinical parameter scoring.

**Results**

While TTI-621 monotherapy inhibited tumor growth in the BRCA competent xenograft model, the combination of TTI-621 and RT significantly improved survival compared to RT alone. Mice bearing BRCA knock-down tumors had improved survival with the TTI-621+niraparib combination compared to vehicle control. TTI-621 significantly enhanced survival when combined with RT, with extended survival observed in the RT+TTI-621+niraparib group. No chronic toxicity was observed for all the treatments.

**Conclusions**

The current study provides supportive evidence for combining innate modulation (TTI-621) with radiation therapy to improve overall survival in patients with OVCa. Additionally, patients with BRCA mutated tumors may benefit from triple therapy with TTI-621, niraparib and RT. Further studies are needed to assess the effect in models with intact adaptive immune systems.

**Ethics Approval**

The study was approved by the University Health Network's Animal Use Committee, protocol number 5603.

#### P363 Pegzilarginase in combination with agonist anti-OX40 therapy enhances T cell priming and effector function leading to improved tumor regression and survival

##### Melissa Kasiewicz, BS^1^, Annah Rolig, Ph D^1^, Elizabeth Sturgill, PhD^1^, Mark Badeaux, PhD^2^, Scott Rowlinson, PhD^2^, William L. Redmond, PhD^1^

###### ^1^Earle A. Chiles Research Institute, Portland, OR, USA; ^2^Aeglea BioTherapeutics, Austin, TX, USA

####### **Correspondence:** William L. Redmond (william.redmond@providence.org)

**Background**

Tumor cells defective in enzymes required for arginine biosynthesis are dependent upon arginine uptake from the environment. Extracellular depletion of arginine directly affects tumor cells, inducing autophagy and apoptosis. Pegzilarginase (AEB1102) is a bioengineered, pegylated, human arginase 1 (Aeglea Biotherapeutics) currently in phase I clinical trials. This arginine-depleting agent has been shown to both inhibit arginine auxotrophic tumor growth and to enhance the efficacy of PD-L1 blockade in preclinical models. In the current study, we investigated the therapeutic efficacy and mechanism of action of combined pegzilarginase/anti-OX40 (aOX40) immunotherapy. We hypothesized that pegzilarginase/aOX40 treatment would synergize to enhance T cell priming and effector function leading to improved tumor regression and survival.

**Methods**

Efficacy studies were conducted in CT26 (colon) or MCA-205 (sarcoma) tumor-bearing mice. Eight days after subcutaneous tumor implantation, mice were treated with pegzilarginase (3 mg/kg; q7dx4; ip) and/or aOX40 mAb (10 mg/kg; d8, d12; ip). Seven days post-treatment (d15), the phenotype and effector status of T cells and myeloid populations within the lymph nodes (LN) and tumor were evaluated by flow cytometry. In additional cohorts, gene expression profiling (single cell RNAseq; scRNAseq) was performed 3 days post-treatment (d11). Survival studies were conducted in both models with tumor measurements taken twice weekly until tumor burden was greater than 150mm2. Data represents the results of 2-3 independent experiments (n=10/group) and for phenotyping assays, significance was determined by using a one-way ANOVA with a p-value cut-off of 0.05.

**Results**

We observed a significant reduction in tumor growth and increased overall survival following pegzilarginase/aOX40 therapy versus monotherapy in CT26 (p<0.01) and MCA-205 (p<0.01) tumor-bearing mice. Flow analysis revealed increased CD8+ T cell activation and effector function, as evidenced by higher levels of granzyme A, IFN-g and TNF-a. Evaluation of tumor infiltrating lymphocytes (TIL) showed increased granzyme A+ CD8+ T cells, but no differences among effector CD4 T cells, supporting the hypothesis that pegzilarginase /aOX40 therapy augments CD8 T cell-mediated anti-tumor immunity. Preliminary analysis of MDSC populations suggests a trend toward higher PD-L1 expression following combination therapy.

**Conclusions**

Collectively, these data demonstrate that pegzilarginase in combination with OX40 agonists can significantly impair tumor growth while promoting both T cell proliferation and effector function. These insights support further exploration of this novel combination approach in future clinical trials.

#### P364 Systemic anti-tumor immunity and immune memory formation by a novel TLR7/8 targeting agent NKTR- 262 combined with CD122-biased immunostimulatory cytokine NKTR-214

##### Saul Kivimae, PhD, Marlene Hennessy, BS, Rhoneil Pena, Yolanda Kirksey, MSc, Wildaliz Nieves, PhD, Phi Quach, BS PhD, Janet Cetz, BS, Zhongxu Ren, PhD, Haiying Cai, BS, BoLiang Deng, PhD, Wen Zhang, PhD, Fiore Cattaruzza, Christie Fanton, PhD, Neel Anand, PhD, Werner Rubas, PhD, Stephen Doberstein, PhD, Loui Madakamutil, Jonathan Zalevsky, PhD

###### Nektar Therapeutics, San Francisco, CA, USA

####### **Correspondence:** Saul Kivimae (skivimae@nektar.com)

**Background**

NKTR-262 is a novel intratumorally administered TLR7/8 agonist currently being investigated in the clinic in combination with NKTR-214, a systemically administered CD122-biased cytokine. NKTR-262 promotes an immune stimulatory environment and local injection site tumor antigen production limiting agonist release to systemic circulation. When NKTR-262 is administered in combination with NKTR-214 the combined effect of innate immune stimulation and enhanced antigen presentation with sustained T cell activation leads to systemic tumor immunity.

**Methods**

Syngeneic mouse tumor models with diverse histologies (CT26, EMT6, 4T1) were assessed for NKTR-262 and NKTR-214 combination treatment efficacy and immune system activation. Tumors were inoculated bilaterally to assess abscopal efficacy and systemic dissemination of anti-tumor CD8+ T cells. Once established, tumors were treated with a single unilateral peritumoral dose of NKTR-262, while NKTR-214 was administered i.v. on q9dx3 schedule. Efficacy correlating immune cell phenotyping was conducted from tumors and blood by flow cytometry to assess combination treatment synergy. Regression of NKTR-262-injected and contralateral tumors was assessed by tumor size measurements. NKTR-262 and NKTR-214 combination effect on T cell clonality and TIL infiltration were assessed by Adaptive ImmunoSEQ platform in the CT26 tumor model. Durable anti-tumor immune memory formation was assessed by tumor volume and immune phenotyping measurements in tumor rechallenge studies in CT26 and EMT6 models.

**Results**

NKTR-262 and NKTR-214 in combination showed efficacy in all tested tumor models, from significant tumor growth inhibition to complete tumor clearance in multiple models. Synergistic efficacy correlated with sustained systemic expansion of tumor antigen specific CD8+ T cells that specifically required coordinated activation of the innate and adaptive arms of the immune system. NKTR-262 treatment significantly increased clonality and infiltration of NKTR-214 expanded T cells, accelerating expansion of tumor infiltrating clones. NKTR-262 and NKTR-214 combination treatment led to durable immune memory and resistance to secondary tumor challenge in multiple models correlating with spontaneous cytotoxic T cell response in secondary tumor lesions.

**Conclusions**

We present a designed combination therapy that mimics a natural immune response by activating a broad immune cell network in multiple nonclinical tumor models. Combining NKTR-262 with NKTR-214 engages the entire immune activation cascade required for systemic tumor clearance. Combination treatment coordinates tumor antigen presentation and costimulatory signaling with tumor antigen recognizing CD8+ T cell expansion to produce a sustained systemic anti-tumor immune response. A comprehensive anti-tumor immune activation by coordinated engagement of innate and adaptive immune cells may increase the success of immune therapy for patients.

**Ethics Approval**

All animal care and procedures were ethically approved and performed according to the AAALAC accredited Nektar Therapeutics IACUC guidelines, approval number 2017-001.

#### P365 Triple checkpoint blockade targeting PD-1, TIM-3, and LAG3 improves T cell reinvigoration and antitumor efficacy over single and double combinations

##### Johanna Kaufmann, PhD, Geeta Sharma, Srimoyee Ghosh, PhD, Sujatha Kumar, PhD, Kevin Coleman, PhD, Sridhar Ramaswamy, MD, David Jenkins, PhD

###### TESARO, Inc., Waltham, MA, USA

####### **Correspondence:** Johanna Kaufmann (jkaufmann@tesarobio.com)

**Background**

Single T cell checkpoint blockade (CPB) with anti-PD-1/PD-L1 antibodies induces durable antitumor responses in subsets of patients. However, multiple immune checkpoint receptors (ICRs) are sequentially upregulated upon T cell activation and are also markers of an exhausted phenotype after chronic activation in the context of infections and cancer. We and others have described expression of the ICRs PD-1, TIM-3, and LAG3 on various tumor-infiltrating immune cell types across tumor types, suggesting their targeting may have applicability for the treatment of multiple cancer types. Here, we explore the functional effects of triple blockade of PD-1, TIM-3, and LAG3 on T cell activation, tumor immune contexture, and antitumor activity in preclinical models.

**Methods**

To examine the ability of combination CPB to reverse T cell exhaustion, splenocytes from mice with a transgenic CD4+ T cell receptor were stimulated with 2 different peptide sequences of myelin basic protein to produce responsive effector or exhausted T cells. CD4+ T cells were restimulated in the presence of antibodies and activity was quantified by IFN-γ release. To examine antitumor activity and pharmacodynamic effects in vivo, Balb/c mice were inoculated with the syngeneic breast cancer cell line EMT6 and treated with antibodies alone or in combination. Tumor growth was measured, and tumor-infiltrating lymphocytes were characterized by flow cytometry.

**Results**

Triple combination treatment with anti-PD-1, anti-TIM-3, anti-LAG3 antibodies was able to fully reverse the exhausted phenotype of CD4+ T cells in vitro, as determined by restored IFN-γ release. In contrast, single antibody treatments only partially reinvigorated T cells, while double combinations displayed an intermediate effect. This improved reinvigoration translated into increased antitumor efficacy in vivo. Control of tumor growth increased incrementally from single (6-24% tumor growth inhibition [TGI]) to double (40-50% TGI) and triple combinations (61% TGI). This reduction of tumor growth was associated with significant increases in overall immune cell infiltrate, CD8+ T cell infiltration, and M1/M2 macrophage ratios and a decrease in intratumoral CD11b+ cell numbers after triple combination treatment, suggesting a broad modulation of the tumor microenvironment.

**Conclusions**

Triple blockade of PD-1, TIM-3, and LAG3 resulted in highly effective reversal of T cell exhaustion and achieved improved tumor control over single or double combinations. By targeting immune checkpoints expressed on multiple cell types, additional mechanisms of tumor immune control were engaged. Taken together, these data support the concept of double and triple combinations of blocking antibodies to PD-1, TIM-3, and LAG3.

#### P366 Blockade of the PD-1/PD-L1 pathway with the Anti-PD-1 mAb, MGA012, enhances the biological activity of the B7-H3 x CD3 bispecific DART® molecule, MGD009

##### Liqin Liu, PhD, Yinhua Yang, Ralph Alderson, PhD, Jonathan Li, PhD, Qihong Xu, Daorong Liu, Robert Burns, Vatana Long, Syd Johnson, PhD, Ezio Bonvini, Paul Moore, PhD

###### MacroGenics, Rockville, MD, USA

####### **Correspondence:** Paul Moore (moorep@macrogenics.com)

**Background**

While checkpoint inhibitors have dramatically improved disease outcomes for patients with certain types of tumors, a significant proportion of patients do not benefit from these agents. Moreover, checkpoint inhibitors are most effective in immunogenic tumors with high mutational burden and pre-existing T-cell infiltration, an indication of an ongoing but thwarted immune response. Combinations with agents that have complementary mechanisms of actions, such as T-cell recruiting agents, may provide expanded benefit to patients with resistance or limited response to checkpoint inhibitor treatment. MGD009, is a clinical stage B7-H3 x CD3 bispecific DART molecule designed to redirect T cells to lyse B7-H3-positive tumor cells. Preclinical studies demonstrated that MGD009 mediates potent anti-tumor activity associated with T-cell activation, expansion and infiltration into tumor sites. Notably, MGD009 activity is also associated with upregulation of PD-1 on T cells and PD-L1 on both tumor and T cells. To address whether the antitumor activity of MGD009 could be further enhanced by coordinating blockade of the PD-1/PD-L1 pathway, we have performed combination studies of MGD009 with MGA012, a clinical-stage anti-PD-1 mAb. also known as INCMGA012.

**Methods**

T-cell receptor (TCR)-mediated signaling was evaluated using a PD-1/PD-L1 dependent co-culture reporter system in the presence of MGD009 ± MGA012. In vitro redirected T-cell killing assays were performed using JIMT-1/Luc as target cells and T cells as effectors. In vivo studies were conducted in human PBMC-reconstituted xenografts in MHC class I-null NSG™ mice. Flow cytometry and cytokine multiplex assays were used to evaluate surface/intracellular markers and cytokine levels.

**Results**

Blockade of the PD-1/PD-L1 checkpoint axis with MGA012 enhanced B7-H3 expression-dependent, MGD009- induced NFAT signaling beyond that observed with MGD009 alone in a co-culture reporter assay. MGA012 augmented MGD009-mediated tumor cell lysis of B7-H3+ve tumor cells in redirected T-cell killing assays. In vivo anti-tumor activity of MGD009 was further enhanced by the addition of MGA012 in a human PBMC-reconstituted mouse xenograft model. Mechanism of action studies revealed that MGD009 and MGA012 co-operate to augment granzyme A/B, perforin expression, T-cell activation and expansion beyond that achieved with MGD009 alone and in a B7-H3-dependent manner. Significantly, MGA012 further increased the fraction of central and effector memory T-cells induced by MGD009.

**Conclusions**

The combination of MGD009 with MGA012 exerts enhanced cellular signaling and T-cell responses in vitro and increased anti-tumor activity in vivo beyond that achieved with MGD009 alone. These proof-of-principle studies provides rationale for clinically testing this combination approach.

**Ethics Approval**

All in vivo studies were reviewed and approved by MacroGenics Institutional Animal Care and Use Committee (IACUC)

#### P367 Synergistic anti-tumor effects of TLR4 agonist G100 and anti-OX40 antibody

##### Hailing Lu, MD, PhD, Alec Betancur, Howard Lee, Jan Ter Meulen, MD, PhD

###### Immune Design, Seattle, WA, USA

####### **Correspondence:** Hailing Lu (hailing.lu@immunedesign.com)

**Background**

Intratumoral injection of G100 (Glucopyranosyl Lipid A in stable emulsion) has shown potent anti-tumor effects. Clinical trials evaluating G100 in patients with follicular lymphoma (FL) (NCT02501473) have shown significant objective responses in treated and non-treated (abscopal) lesions (Flowers, ASH 2017). Mechanistic studies have shown that the systemic anti-tumor effects of G100 are mediated by CD8 T cells. Tumor biopsies from FL patients demonstrated increased CD8 T cell infiltration post-G100 treatment, which correlate with clinical response. We hypothesize that the ability of G100 to increase T cell infiltration into tumors will synergize with therapies that separately activate T cells via OX40.

**Methods**

The combination therapy (G100+α-OX40 Ab) was studied in a bilateral A20 lymphoma and B16 melanoma models. In the A20 model, BALB/c mice received inoculation with 5E6 A20 cells on both flanks on Day 0. In the B16 model, C57BL/6 mice received an inoculation with 1E5 B16-ova or B16-F10 cells on Day 0 on the right flank and in some studies a 2nd tumor inoculation on Day 3 on the left flank. Treatment started after tumors were established (Days 5-8). Mice received treatment with G100 alone (10 μg, IT, 3x/week, injected into tumor on one side only), α- OX40 Ab (clone OX86, 200 μg, IP, 1x/week), or the combination of G100 plus α-OX40 Ab. Tumor growth was monitored via caliper measurement.

**Results**

G100+α-OX40 was more potent than either single agent in controlling the growth of both G100-treated and abscopal tumors. In the A20 model, the overall survival rate after complete regression of both treated and abscopal tumors was 20% in the G100 monotherapy group, 10% in the α-OX40 monotherapy group, and 60% in the G100+α-OX40 group (p=0.01 for G100+α-OX40 vs. G100 alone; p=0.0037 for G100+α-OX40 vs. α-OX40). At three months post primary tumor inoculation, all survival mice rejected a tumor re-challenge with A20 cells. In the B16 melanoma model, G100+α-OX40 resulted in better tumor control and significantly longer survival compared to either monotherapy (p=0.0016 for G100+α-OX40 vs. G100 or α-OX40 alone). Complete regression of treated and abscopal lesions only occurred in mice receiving combination therapy. The survival mice rejected re-challenge with B16-ova and with wildtype B16-F10 tumors, indicating long-term memory responses.

**Conclusions**

Combination therapy using intratumoral injection of G100 with systemic delivery of anti-OX40 has synergistic anti-tumor effects in preclinical tumor models, which supports clinical evaluation.

#### P368 Combination of a dipeptidyl peptidase inhibitor BXCL701 and biased CD122 agonist NKTR-214 with anti- PD1 provides functional immunological memory through inflammatory cell death

##### John MacDougall, PhD^1^, Snigdha Gupta, PhD^2^, Veena Agarwal^1^, Luca Rastelli^1^, Annie An^3^, wenqing yang, PhD^3^, Henry Li^3^, Deborah Charych, PhD^4^, Jonathan Zalevsky, PhD^4^, Vincent O'Neill^1^

###### ^1^BioXcel Therapeutics, New Haven, CT, USA; ^2^BioXcel Corporation, Gurugram, India; ^3^Crown Bioscience, San Diego, CA, USA; ^4^Nektar Therapeutics, San Francisco, CA, USA

####### **Correspondence:** John MacDougall (jmacdougall@bioxceltherapeutics.com)

**Background**

BXCL701 (Talabostat; Val-boroPro) is a potent inhibitor of dipeptidyl peptidases, including DPP8, DPP9, and fibroblast activation protein (FAP). Utilizing Artificial Intelligence approaches BXCL701 was uncovered as an agent that would potentially synergize with existing immunotherapies as novel combinations for cancer treatment. Our hypothesis was confirmed with the observation that BXCL701, in combination with an anti-PD-1 antibody and NKTR-214 (a CD122-biased agonist) results in complete and durable response with functional demonstration of immunologic memory in a syngeneic mouse model of pancreatic cancer (Pan02) [1]. In this case, it was only the triple combination that generated complete and durable responses implying that immune activation by these three agents were non-redundant and complementary.

**Methods**

Here we extend those observations to other syngeneic mouse models (MC-38, Wehi-164) in which this combination of agents was similarly able to generate complete and durable responses with functional immunologic memory. However, complete and durable responses were not observed in all models tested (RM-1, B16F10). We used the differential responses observed in these models to assess what tumor associated immune cells might correlate with response to this triple combination.

**Results**

Using IHC and flow cytometry based immunophenotyping data we found that tumor models which were responsive to the triple combination had high densities of tumor associated macrophages, whereas those models that were less responsive had low macrophage densities. Recent literature demonstrating that inhibition of DPP8 and DPP9 in macrophages by BXCL701 activates the Nlrp1b inflammasome, resulting in an inflammatory cell death termed pyroptosis, supports a mechanistic based hypothesis for tumor responses [2,3].

**Conclusions**

Thus, it is proposed that BXCL701 stimulated macrophages rapidly prime the tumor microenvironment for other immune effector cells, those of which are similarly primed by a combination of checkpoint inhibition and NKTR- 214 stimulation. These data validate that a complete anti-tumor response in these models requires engagement of multiple cell types of the immune system, both innate and adaptive. These data provide the basis for a mechanistically based predictive biomarker that can potentially be used in the clinical application of this triple combination therapy.

**References**

1. Rastelli L, Gupta S, Dahiya A, Jagga Z, Nandabalan K, Upmanyu S. Efficacy and immune modulation by BXCL701 a dipeptidyl peptidase inhibitor, NKTR-214 a CD122-biased immune agonist with PD1 blockade in murine pancreatic tumors. 2018 ACSO Annual Meeting, Abstract #3085

2. Okondo MC, Johnson DC, Sridharan R, Go EB, Chui AJ, Wang MS, Poplawski SE, Wu W, Liu Y, Lai JH, Sanford DG, Arciprete MO, Golub TR, Bachovchin WW, Bachovchin DA. DPP8 and DPP9 inhibition induces pro- caspase-1-dependent monocyte and macrophage pyroptosis. Nat Chem Biol. 2017 Jan;13(1):46-53.

3. Okondo MC, Rao SD, Taabazuing CY, Chui AJ, Poplawski SE, Johnson DC, Bachovchin DA. Inhibition of Dpp8/9 Activates the Nlrp1b Inflammasome. Cell Chem Biol. 2018 Mar 15;25(3):262-267

#### P369 Pegilodecakin, a pegylated human IL-10 (AM0010), enhances the cytotoxicity of CAR-T cells *In Vitro*

##### Scott McCauley, BA, Rakesh Verma, PhD, Martin Oft, MD

###### ARMO Biosciences, Redwood City, CA, USA

####### **Correspondence:** Scott McCauley (scott.mccauley@armobio.com)

**Background**

Immune therapies rely on expansion of anti-tumor T cells for tumor regression and successful therapeutic outcomes. Recent studies of Pegilodecakin, a pegylated interleukin-10 (IL-10), have demonstrated that doses greatly exceeding typical endogenous levels can drive a productive tumor specific T cell expansion and response. In a large Phase I/Ib study, Pegilodecakin achieved objective responses across multiple tumor types, alone and in combination with chemotherapies and Programmed Cell Death-1 (PD-1) inhibitors. Agents that improve the functional expansion of chimeric antigen receptor (CAR) T cells, post adoptive transfer, hold promise to improve the therapeutic efficacy of CAR-T therapy in patients. Here we report on early *in vitro* studies to demonstrate that Pegilodecakin significantly enhances the anti-tumor cytotoxic T lymphocyte (CTL) activity of CAR-T cells.

**Methods**

The engineered Primary CD19 CAR-T cells were generated by isolating human T cells from whole blood and transducing these T cells with a CD19-targeted CAR. The CD19 CAR-T cells were then activated, expanded, and tested in a Real-time Cytotoxicity Assay (RTCA) against HeLa cells stably expressing human CD19 (CD19-HeLa). Multiple effector-to-target (E:T) ratios were tested and the CTL activity was measured through cell-sensor impedance in an electronic microtiter plate. Cytotoxicity was measured for CD19 CAR-T alone or in combination with varied concentrations of Pegilodecakin, and was directly compared with controls, including Pegilodecakin alone or Pegilodecakin with non-transduced T cells. To functionally validate the CTL activity, we measured the levels of Granzyme-B and Interferon-gamma (IFNg) in the culture supernatants by ELISA at the end of the RTCA.

**Results**

The RTCA revealed that Pegilodecakin in combination with the CD19 CAR-T showed a significant increase in CTL activity (P<0.001) against CD19-HeLa cells as compared to the CD19 CAR-T alone. Functionally, when combined with Pegilodecakin, CD19 CAR-T cells produced significantly higher levels of Granzyme-B (p<0.0005) and IFNg (p<0.02) as measured at the end of the RTCA. Controls, including Pegilodecakin alone (without CAR-T or non- transduced T cells) and non-transduced T cells in combination with Pegilodecakin, had significantly lower CTL activity by RTCA or ELISA (Granzyme-B or IFNg).

**Conclusions**

We demonstrate that when combined with Pegilodecakin, the CTL activity of the CD19 CAR-T is significantly improved. Additionally, this combination demonstrates significantly improved functional activity of these CD19 CAR T cells, including significantly increased Granzyme-B and IFNg levels relative to controls. These encouraging results warrant further examination of Pegilodecakin/CAR-T therapy combinations *in vivo*, with an aim towards improving the clinical use and activity of CAR-T therapies.

**Acknowledgements**

CAR-T platform was made available by ProMab Biotechnologies, who generated the *in vitro* data in this study

#### P370 Forced expression of OX40L and inhibition of IDO within the murine glioblastoma microenvironment creates a potent anti-tumor immune response

##### Teresa Nguyen, BS^2^, Yisel Rivera-Molina, PhD^2^, Francisco Puerta-Martinez, PhD^2^, Debora Kim^2^, Xuejun Fan, BS^2^, Frederick Lang, MD^2^, Hong Jiang, PhD^2^, Candelaria Gomez-Manzano, MD^2^, Juan Fueyo^2^

###### ^1^The University of Texas, Houston, TX, USA; ^2^MD Anderson Cancer Center, Houston, TX, USA

####### **Correspondence:** Juan Fueyo (jfueyo@mdanderson.org)

**Background**

An immunosuppressive tumor-microenvironment characterizes glioblastoma (GB). Regulatory T-cells can activate indolamine-2,3-dioxygenase (IDO) causing immunosuppression. IDO is upregulated in GB patients, and correlates with a poor prognosis. The use of the oncolytic adenovirus, Delta-24-RGD, has been shown to induce complete responses in a subset of GB patients by immune mechanisms that activate anti-tumor cytotoxic properties of T-cells. This cytotoxic effect can be enhanced by the addition of immune agonists, such as OX40L, a T-cell co-stimulator. We hypothesized that combining IDO inhibition (Indoximod) and Delta-24-RGD armed with OX40L (D24- RGDOX) will have an enhanced therapeutic effect in GB.

**Methods**

A GB mouse model was used to determine therapeutic efficacy of D24-RGDOX and Indoximod as single agents or in combination. C57BL/6 mice were intracranially implanted with syngeneic GB cells, followed by intratumoral viral injections and/or Indoximod treatments. We sacrificed the mice on day 24 post-tumor implantation for immunological studies, including co-culture of splenocytes from differentially treated mice with cancer cells for determining secretion levels of IFN-gamma. We also quantified T-cells of different immunophenotypes of brain infiltrating lymphocytes (BILs) by flow cytometry. Lastly, we performed a survival study.

**Results**

The co-culture experiment showed that splenocytes from the combination-treated mice produced the highest amount of IFN-gamma compared to either single agent treatment (ANOVA, p<0.0001). Additionally, immunophenotyping of murine BILs by flow cytometry revealed that combination-treated mice led to the highest percentage of CD45+ cells (ANOVA, <0.0001). Moreover, mice treated with the combination therapy yielded the highest infiltration of PD-1+TIM-3+ exhausted CD8 T-cells compared to the Delta-24-RGDOX or Indoximod treated groups (ANOVA, p<0.0001), and correlated with complete tumor elimination as shown by H&E staining. Interestingly, the survival data shows that the combination treatment induced a greater survival benefit compared to Indoximod or D24- RGDOX alone, resulting in the most long-term survivors (mean survival, days, Indoximod=37, D24-RGDOX=47, combination=109 days, Logrank test for trend, p<0.0001) and re-challenged survivors.

**Conclusions**

The co-culture results indicate increased activation of T-cells by the combination treatment. The increase of CD45 cells in combination-treated mice compared to Delta-24-RGDOX indicate enhanced infiltration of immune cells by Indoximod. Moreover, the increase of exhausted T-cells in combination-treated mice and the accompanied complete tumor regression suggest there is a process of active tumor-targeting T-cells converting into exhaustive T-cells during therapy. The re-challenge survival data indicate the establishment of immune memory by D24-RGDOX and Indoximod, and support the use of IDO inhibitors with armed oncolytic adenoviruses as a potential treatment for GB.

#### P371 Reprogramming the immune phenotype of Rb-deficient tumor cells using BET inhibition

##### Brian Olson, PhD^1^, Christina Hong^1^, Riyue Bao, PhD^2^, Gregory Lesinski, PhD, MPH^1^, Akash Patnaik, MD, PhD^2^

###### ^1^Emory University, Atlanta, GA, USA; ^2^University of Chicago, Chicago, IL, USA

####### **Correspondence:** Brian Olson (brian.olson@emory.edu)

**Background**

Immune checkpoint blockade has revolutionized the treatment of several malignancies. However, subsets of patients fail to respond, highlighting the urgency of identifying predictive biomarkers to guide rational combinatorial treatment strategies. We have previously shown tumors lacking a T cell-inflamed gene signature (a predictor of immunotherapeutic efficacy) are enriched for loss of the tumor suppressor RB1. Based on this observation, we examined the immunological consequences of RB1 loss in prostate tumor cells and whether targeted therapeutics aimed at reversing the molecular consequences of RB1 loss (namely inhibitors of the bromodomain and extraterminal (BET) domain family of proteins) can reprogram the immune phenotype of Rb-deficient tumors.

**Methods**

Isogenic murine prostate tumor cells with or without Rb expression were generated and interrogated for changes in markers related to an immunosuppressive phenotype, including chemokine and checkpoint ligand expression and effects on T cell migration in vitro and in vivo. These cell lines were then evaluated for the effects of RB1 loss towards susceptibility to BET inhibition, in terms of effects on tumor cell viability as well as effects on immune phenotype.

**Results**

Loss of RB1 in prostate tumor cells resulted in decreased expression of chemokines associated with immune infiltration and function, increased expression of multiple checkpoint ligands, as well as soluble factors resulting in decreased T cell migration. When examined in tumor-bearing animals in vivo, RB1 loss translated into decreased immune infiltration into the tumor microenvironment. Tumor cells lacking RB1 had increased susceptibility to BETi-mediated tumor cell death, with cells surviving BET inhibition displaying pharmacodynamic inhibition of BET family member function. Rb-deficient tumor cells also displayed increased susceptibility to BETi-mediated reprogramming of their immune phenotype, including decreased expression of checkpoint ligands such as PD-L1, Gal9, and VISTA, decreased expression of chemokines associated with tumor growth and immune suppression such as CXCL1 and CXCL5, as well as a complete restoration of T cell migration.

**Conclusions**

We show that loss of RB1 results in an immunosuppressive tumor microenvironment, and that Rb-deficient tumor cells have increased susceptibility to BET inhibition in terms of tumor cell-intrinsic and extrinsic efficacy. Targeting the immunological consequences following BET inhibition in Rb-deficient tumors may provide a rational approach for combined pharmacological and immune-based treatment strategies for individuals with Rb-deficient malignancies.

**Acknowledgements**

American Cancer Society (BMO), Prostate Cancer Foundation (AP/BMO), NCI National Cancer Institute Prostate SPORE-University of Chicago/Northwestern University (AP).

**Ethics Approval**

This study was approved by the Emory University Institutional Animal Care and Use Committee; protocol number 3000268.

#### P372 BETAMUNE, a replication competent type 5 adenovirus carrying a TGF-Beta trap, reverses resistance to a PD-L1 inhibitor in an immunocompetent mouse model

##### Christopher Larson, MD PhD, Corey Carter, MD, Bryan Oronsky, MD, PhD, Tony Reid, MD PhD

###### EpicentRx Inc, La Jolla, CA, USA

####### **Correspondence:** Bryan Oronsky (boronsky@epicentrx.com)

**Background**

Checkpoint inhibitors have permanently changed the therapeutic landscape for multiple tumor types previously associated with a dismal prognosis. However, for the subset of patients that initially benefit from these inhibitors, lethal secondary resistance often develops. We evaluated combination therapy with a preclinical oncolytic adenovirus called BETAMUNE, armed with a TGF-β “trap” that neutralizes the immunosuppressive cytokine, TGF- β, and a checkpoint inhibitor, anti-PD-L1, in PD-L1 resistant tumors. The study, which was performed in an immunocompetent mouse model, demonstrated that the combination of BETAMUNE with PD-L1 blockade reversed PD-L1 resistance, potentially representing a future paradigm shift for patients that are primarily or secondarily resistant to checkpoint inhibitors.

**Methods**

Murine KRAS mutant lung adenocarcinoma cell line, ADS-12, was established in house. BETAMUNE, produced in HEK-293 cells, carries a disruption in E1A and a TGFβR-IgG fusion using the mouse isoforms of those genes for immunologic compatibility with an immunocompetent mouse. 129S4/SvJae mice, implanted with subcutaneous ADS-12 tumors, were randomized into treatment groups 10 mice per group. Treatment involved intratumoral injections of either viral storage buffer or BETAMUNE at 10(to the power of 9) PFU/dose on days 0, 4, and 8, plus intraperitoneal injections of either phosphate buffered saline (PBS) or 200 μg anti-PD-L1 antibody (clone 10F.9G2, BioXcell) diluted in PBS on days 1, 5, 9, and 13.

**Results**

All mice tolerated the treatments without obvious signs of toxicity. No activity was evident with anti-PD-L1 antibody alone. Treatment with BETAMUNE alone in these larger tumors led to complete responses in four of ten mice, and combination therapy led to complete responses in seven of ten mice. Tumor volume was smaller in the combination therapy group compared to 19k-mTGFβR-IgG alone ten days after starting treatment (p<0.01).

**Conclusions**

This study demonstrates that localized oncolytic infection with BETAMUNE is safe and abrogates resistance to systemic PD-L1 immunotherapy, which strongly supports further evaluation of this combination in upcoming Phase 1 and Phase 2 clinical studies in 2019.

**Ethics Approval**

All applicable international, national, and/or institutional guidelines for the care and use of animals were followed.

#### P373 Synergistic efficacy of duvelisib with checkpoint or co-stimulatory antibodies in a B cell lymphoma model: Advantages of dual inhibition of PI3K-delta and PI3K-gamma

##### Jonathan Pachter, PhD, David Weaver, PhD

###### Verastem, Needham, MA, USA

####### **Correspondence:** Jonathan Pachter (jpachter@verastem.com)

**Background**

Duvelisib is an oral dual inhibitor of phosphoinositide 3-kinase (PI3K)-delta and PI3K-gamma which has shown clinical activity as monotherapy in chronic lymphocytic leukemia (CLL), small lymphocytic lymphoma (SLL), follicular lymphoma (FL), and T cell lymphoma [1,2]. Recent publications have demonstrated that PI3K-delta inhibition reduces immunosuppressive Tregs and enriches memory T cells [3,4], whereas PI3K-gamma inhibition reduces immunosuppressive myeloid cells [5,6]. Hence, we postulated that duvelisib may augment the efficacy of immune checkpoint or co-stimulatory antibodies.

**Methods**

Mice bearing syngeneic A20 B cell lymphoma tumors (60-90 mm3) were treated with vehicle, duvelisib, anti-PD-1, anti-PD-1 + duvelisib, anti-OX40, or anti-OX40 + duvelisib. Tumor volumes were measured by caliper. Tregs, macrophages and MDSCs were quantified by flow cytometry from mice bearing A20 tumors after 8 days of treatment.

**Results**

In the A20 model, duvelisib, anti-PD-1 and anti-OX40 treatments each induced tumor growth delay. When duvelisib and anti-PD-1 were combined in mice with pre-existing A20 tumors, strong anti-tumor synergy was observed. When anti-OX40 and duvelisib were combined, tumor regression was observed which correlated with strong reduction of tumor Tregs, M2 macrophages and MDSCs. To assess immune memory, tumor-free mice following anti-OX40 alone or anti-OX40 + duvelisib were injected with A20 cells in the contralateral flank with no further treatment. Whereas mice that had received anti-OX40 alone grew new tumors upon A20 re-challenge, all tumor-free mice that had received anti-OX40 + duvelisib did not grow tumors upon re-challenge and showed elevated memory T cells in blood and spleen. These findings indicate that the anti-OX40 + duvelisib treatment established immune memory, potentially contributing to the observed tumor regression. Mechanistically, duvelisib was found to reduce Tregs, M2 macrophages and MDSCs in the context of combinations with PD-1 or OX40 antibodies, and duvelisib (dual PI3K- delta/gamma inhibition) was found to inhibit all 3 immunosuppressive cell populations more effectively than idelalisib (PI3K-delta only) or IPI-549 (PI3K-gamma only).

**Conclusions**

These data demonstrate that duvelisib treatment stimulates anti-tumor immunity. Furthermore, the unique dual inhibition of PI3K-delta and PI3K-gamma appears to make duvelisib especially effective in enhancing the anti-tumor efficacy of immune checkpoint and co-stimulatory antibodies. These data support further exploration of duvelisib in combination with anti-PD-1/PD-L1 or co-stimulatory antibodies in patients with various cancers.

**References**

1. Flinn IW, O'Brien S, Kahl B, Patel M, Oki Y, Foss FF, Porcu P, Jones J, Burger JA, Jain N, Kelly VM, Allen K, Douglas M, Sweeney J, Kelly P, Horwitz S. Duvelisib, a novel oral dual inhibitor of PI3K-delta gamma is clinically active in advanced hematologic malignancies. Blood 2018; 131:877-887.

2. Horwitz SM, Koch R, Porcu P, Oki Y, Moskowitz A, Perez M, Myskowski P, Officer A, Jaffe JD, Morrow SN, Allen K, Douglas M, Stern H, Sweeney J, Kelly P, Kelly V, Aster JC, Weaver D, Foss FM, Weinstock DM. Activity of the PI3K-delta,gamma inhibitor duvelisib in a phase 1 trial and preclinical models of T-cell lymphoma. Blood 2018; 131: 888-898.

3. Ali K, Soond DR, Pineiro R, Hagemann T, Pearce W, Lim EL, Bouabe H, Scudamore CL, Hancox T, Maecker H, Friedman L, Turner M, Okkenhaug K, Vanhaesebroeck B. Inactivation of PI(3)K p110delta breaks regulatory T- cell-mediated immune tolerance to cancer. Nature 2014; 510:407-411.

4. Abu Eid R, Ahmad S, Lin Y, Webb M, Berrong Z, Shrimali R, Kumai T, Ananth S, Rodriguez PC, Celis E, Janik J, Mkrtichyan M, Khleif SN. Enhanced therapeutic efficacy and memory of tumor-specific CD8 T cells by ex vivo PI3K-delta inhibition. Cancer Res 2017; 77:4135-4145.

5. Kaneda MM, Messer KS, Ralainirina N, Li H, Leem CJ, Gorjestani S, Woo G, Nguyen AV, Figueiredo CC, Foubert P, Schmid MC, Pink M, Winkler DG, Rausch M, Palombella VJ, Kutok J, McGovern K, Frazer KA, Wu X, Karin M, Sasik R, Cohen EE, Varner JA. PI3Kgamma is a molecular switch that controls immune suppression. Nature 2016; 539:437-442.

6. De Henau O, Rausch M, Winkler D, Campesato LF, Liu C, Cymerman DH, Budhu S, Ghosh A, Pink M, Tchaicha J, Douglas M, Tibbitts T, Sharma S, Proctor J, Kosmider N, White K, Stern H, Soglia J, Adams J, Palombella VJ, McGovern K, Kutok JL, Wolchok JD, Merghoub T. Overcoming resistance to checkpoint blockade therapy by targeting PI3K-gamma in myeloid cells. Nature 2016; 539: 443-447.

#### P374 Sex-based heterogeneity of response to immunotherapy in non small cell lung cancer

##### Fabio Conforti, MD^1^, Laura Pala^1^, Vincenzo Bagnardi^2^, Giuseppe Viale^1^, Tommaso De Pas, MD^1^, Elisabetta Pennacchioli^1^, Emilia Cocorocchio^1^, Pier Francesco F. Ferrucci, MD^1^, Richard Gelber^3^, Aron Goldhirsch^1^

###### ^1^European Institute of Oncology, Milan, Italy; ^2^University of Milan-Bicocca, Milan, Italy; ^3^Dana-Farber Cancer Institute, Harvard, Boston, MA, USA

####### **Correspondence:** Fabio Conforti (fabio.conforti@ieo.it)

**Background**

Recently, four large randomized clinical trials (RCTs) demonstrated that pembrolizumab administered alone or in combination with chemotherapy as first line systemic treatment, improved overall survival (OS) of patients with advanced non-small cell lung cancer (NSCLC), as compared with standard chemotherapy alone. [1,4]We previously demonstrated a significant sex-based heterogeneity of the efficacy of anti-CTL4A and anti-PD-1 drugs in several solid tumors, with male patients obtaining higher benefit than females. [5] Given the complex sex-dimorphism of immune system function and response, we hypothesized that the direction of such heterogeneity could be different using different immunotherapeutic strategies. Here, we provide evidence suggesting that adding pembrolizumab to chemotherapy compared with chemotherapy alone in advanced NSCLC leads to an impressive greater OS benefit in women as compared with the benefit observed for men.

**Methods**

We performed a meta-analysis of four RCTs (i.e Keynote 24, 42, 189 and 407; Table1), to assess the interaction between patients’ sex and the efficacy of the two experimental immunotherapeutic strategies (i.e. pembrolizumab alone or pembrolizumab plus standard chemotherapy).[1-4] We tested the null hypothesis that both evaluated immunotherapeutic strategies have homogeneous effect in the same sex.

**Results**

Analysis included 2754 patients, 847 (31%) of whom were females. Results showed that male patients treated with pembrolizumab monotherapy had a significantly reduced risk of death as compared with males treated with standard chemotherapy: pooled-OS HR 0.76 (95% CI, 0.65-0.88;fig.1). In females, pembrolizumab alone was not better than standard chemotherapy: pooled-OS HR 0.90 (95% CI, 0.71-1.15). By contrast, pembrolizumab administered with chemotherapy was associated with a very large OS advantage compared with chemotherapy alone in female patients but a significantly smaller benefit was seen in males (female pooled-OS HR, 0.32; 95% CI, 0.23 -0,46; males pooled-OS HR, 0.69; 95% CI, 0.55 -0.87). The heterogeneity of the efficacy of the two immunotherapeutic strategies in male and female patients was highly significant: the pooled interaction (i.e. the pooled estimate of the ratios of the HRs in males and in females reported in each trial) was 0.82 (95% CI, 0.62-1.1) for pembrolizumab alone, indicating a greater effect of pembrolizumab alone in men with respect to women, and 2.1 (95% CI, 1.36- 3.25) for pembrolizumab plus chemotherapy, indicating a greater effect of pembrolizumab plus chemotherapy versus chemotherapy alone in women as compared with men (p-heterogeneity=0.004; Figure1).

**Conclusions**

These data highlight the need for different immunotherapeutic strategies to be tested taking into account sex-related heterogeneity of responsiveness

**Acknowledgements**

We thank Shari Gelber for editorial assistance.

**References**

1. Reck M, Rodríguez-Abreu D, Robinson AG, et al. Pembrolizumab versus chemotherapy for PD-L1–positive non–small-cell lung cancer. N Engl J Med. 2016;375:1823-1833.

2. Gandhi L, Rodríguez-Abreu D, Gadgeel S, et al. Pembrolizumab plus chemotherapy in metastatic non-small-cell lung cancer. N Engl J Med. 2018;378:2078-2092.

3. Paz-Ares G, Luft A, Ali Tafresh A, et al. Phase 3 study of carboplatin-paclitaxel/nab-paclitaxel (Chemo) with or without pembrolizumab (Pembro) for patients (Pts) with metastatic squamous (Sq) non-small cell lung cancer (NSCLC). J Clin Oncol 2018; 36. (suppl; abstr 105)

4. Lopes G, Wu Y, Kudaba I, et al. Pembrolizumab (pembro) versus platinum-based chemotherapy (chemo) as first-line therapy for advanced/metastatic NSCLC with a PD-L1 tumor proportion score (TPS) ≥ 1%: Open-label, phase 3 KEYNOTE-042 study. J Clin Oncol 2018; 36 (suppl; abstr LBA4)

5. Conforti F, Pala L, Bagnardi V, et al. Cancer immunotherapy efficacy and patients’ sex: a systematic review and meta-analysis. Lancet Oncol. 2018; (18)30261-4. pii: S1470-2045

**Table 1 (abstract P374).**
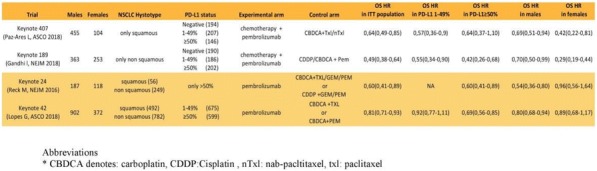
See text for description.

**Fig. 1 (abstract P374).**
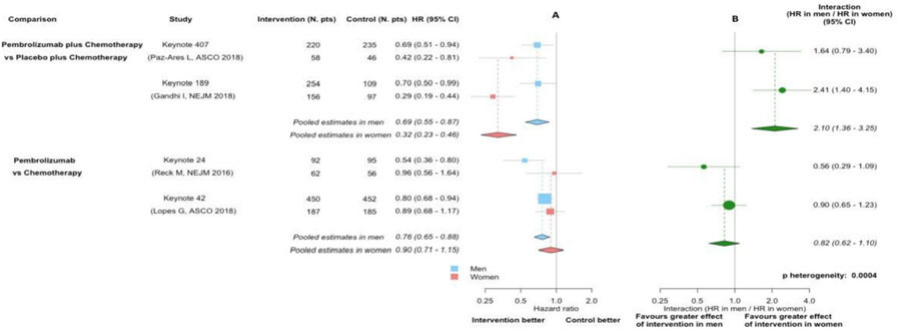
See text for description.

#### P375 Simultaneous costimulatory T-cell engagement and checkpoint inhibition by PRS-344/ONC0055, a 4-1BB / PD-L1 bispecific compound for tumor localized activation of the immune system

##### Marina Pavlidou, PhD^1^, Janet Peper^1^, Lucia Pattarini^2^, Christian Barthels^1^, Eva-Maria Hansbauer^1^, Rachida Bel Aiba^1^, Milan Blanusa^1^, Alix Scholer-Dahirel^2^, Maximilien Grandclaudon^2^, Celine Grand^2^, Jamila Elhmouzi- Younes^2^, Matthieu Rivière^2^, Véronique Blanc, PhD^2^, Christine Rothe^1^, Shane Olwill^1^

###### ^1^Pieris Pharmaceuticals, Freising, Germany; ^2^Institut de Recherches Servier Oncology, Croissy Sur Seine, France

####### **Correspondence:** Christine Rothe (rothe@pieris.com)

**Background**

Multiple lines of evidence show that 4-1BB (CD137), a key costimulatory immunoreceptor, is a highly promising therapeutic target in cancer. Current antibody-based approaches showed immune cell activation not only in tumor tissues but also in the periphery, associated with dose-limiting on-target toxicity and a limited therapeutic window due to peripheral immune cell activation. To overcome this limitation, we generated PRS-344/ONC055, a 4- 1BB/PD-L1 bispecific that is designed to promote 4-1BB clustering by bridging 4-1BB-positive T cells with PD-L1. PD-L1 is the primary ligand of the T cell receptor PD-1 and is expressed in a wide variety of tumors resulting in an inhibitory interaction with PD-1 in the tumor microenvironment. Preclinical evidence suggests that combining 4- 1BB-induced T cell activation and expansion with “anti-PD-L1 mediated” immune checkpoint blockade may overcome the limitation of single agent therapy and offer benefit to ICP-resistant or non-responsive patients. PRS- 344/ONC0055 has been designed to provide the potential of a combinatorial therapy in one molecule but also favors the localized activation of antigen-specific T cells in the tumor microenvironment, potentially reducing peripheral toxicity.

**Methods**

Anticalin® proteins are 18 kDa protein therapeutics derived from human lipocalins. We utilized phage display technologies to generate an Anticalin protein binding to 4-1BB with high affinity and specificity. PRS- 344/ONC0055 was obtained by fusion of the 4-1BB-specific Anticalin protein to a PD-L1-targeting monoclonal antibody with an engineered IgG4 backbone.

**Results**

The bispecific fusion protein PRS-344/ONC0055 targets 4-1BB and PD-L1 with similar affinities as compared to parental building blocks and is capable of binding both targets simultaneously. We show that the bispecific molecule retains its ability to block (PD-1 / PD-L1) receptor-ligand interaction with similar potency to the parental PD-L1 antibody. In ex vivo cell based assays, PRS-344/ONC0055 induces a dose-dependent T cell activation only in the presence of PD-L1 positive cells. We show that PRS-344/ONC0055 synergistic activity is stronger than that mediated by the combination of clinically relevant 4-1BB and PD-L1 benchmark antibodies.

**Conclusions**

We report potent costimulatory T cell engagement of the immunoreceptor 4-1BB in a PD-L1-dependent manner, utilizing the 4-1BB/PD-L1 bispecific compound PRS-344/ONC0055. This approach has the potential to provide a localized activation of the immune system with high efficacy and reduced peripheral toxicity. Furthermore, the direct, PD-L1-targeting activity of PRS-344/ONC0055 provides an additional therapeutic benefit by checkpoint blockade. Taken together our data outlines proof of concept functionality of PRS-344/ONC0055 and supports IND- enabling studies of this promising compound.

#### P376 Targeting vasoactive intestinal peptide signaling to enhance immunotherapy against solid tumors

##### Sruthi Ravindranathan, PhD, Rebecca Pankove, MS, Yiwen Li, MS, Shuhua Wang, MD, Edmund K. Waller, MD, PhD, FACP, Mohammad Zaidi, MD, MS, Gregory Lesinski, PhD, MPH

###### Emory University, Atlanta, GA, USA

####### **Correspondence:** Edmund K. Waller (ewaller@emory.edu)

**Background**

Vasoactive intestinal peptide (VIP) is a neuropeptide synthesized by nerve terminals, the pancreas, GI tract, and immune cells. VIP signaling represents an immune checkpoint pathway as it is produced upon inflammation and induces an immunosuppressive microenvironment. We have previously shown that daily administration of VIPhyb, a competitive peptide antagonist of VIP signaling, improves overall tumor free survival in mouse models of leukemia [1]. In solid tumors, human gene expression data shows VIP expression to greatly vary between different tumors, with highest levels in pancreatic exocrine cancers and lowest in melanoma. We hypothesize that VIP expression by cancers represents a targetable pathway for immune escape, and that antagonists of VIP signaling would induce an anti-tumor response when used alone or in combination with other immune checkpoint inhibitors.

**Methods**

Murine melanoma (B16F10, D4M.3A), breast cancer (4T1, 4T07) and pancreatic cancer (MT5, panc02) cells were cultured for 24 hours and their supernatants were tested for VIP concentration using an enzyme immunoassay. Growth of subcutaneously implanted melanoma (B16F10) and pancreatic cancer (MT5) in C57BL/6 mice was monitored every day following daily administration of 10ug VIPhyb (subcutaneously) starting from day -1 and/or 200ug of IgG2a or anti-PD-1 antibody (intraperitoneally) on day -1 and every 3 days thereafter. The treatment was continued until mice were euthanized when the tumor volume reached 500mm^3. The tumor tissues were stained for VIP, CD8 and DAPI, while the splenocytes were analyzed via flow cytometry for levels of CD3, CD4+, CD8+, CD4+PD1+ and CD8+PD1+ T cells.

**Results**

Supernatants from pancreatic cancer cells had significantly higher levels of VIP when compared to melanoma and breast cancer cells (Table 1).Treatment of immune competent mice bearing melanoma or pancreatic cancer cells with the combination of VIPhyb and anti-PD-1 (combination group) produced complete and durable regression of tumors in 20% of the mice in the melanoma model (Figure 1) and increased median survival in the pancreatic cancer model (Figure 2). Also, there was a significant difference in the frequency of PD1 expressing CD4+ T cells in the spleen of mice in the combination group (Figure 3). Further, immunohistochemistry of the tumor tissue sections showed increased CD8+ T cell infiltration in the tumor of mice in the combination group (Figure 4).

**Conclusions**

Blocking VIP-signaling is a novel immunotherapeutic approach in preclinical solid tumor models. Improving half-life of VIPhyb are underway, to enhance the effectiveness of this peptide even in tumors with potentially high levels of VIP.

**References**

1. Petersen, C T, Li, J M, & Waller, E K, Administration of a vasoactive intestinal peptide antagonist enhances the autologous anti-leukemia T cell response in murine models of acute leukemia. OncoImmunology, 2017; 6(5), e1304336.

**Ethics Approval**

The study was approved by Emory's Institutional Animal Care and Use Committee (IACUC), protocol number 3000202

**Table 1 (abstract P376).**
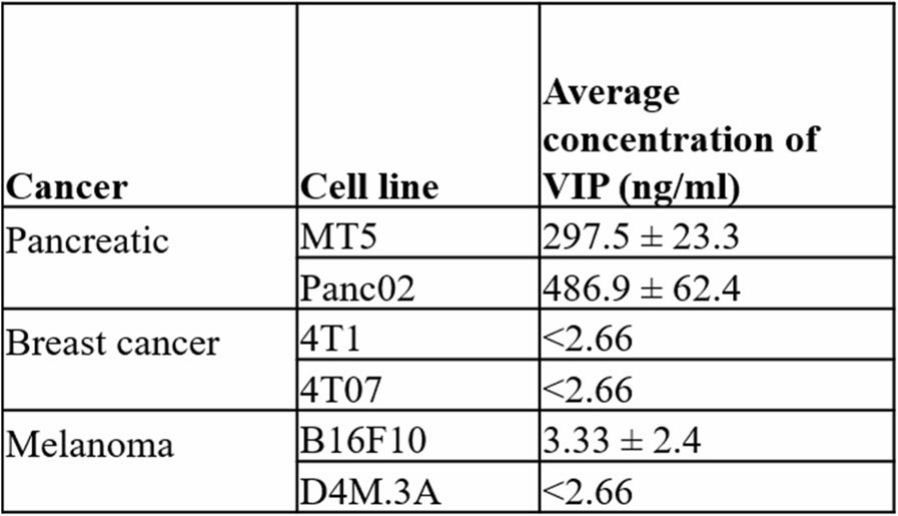
Murine pancreatic cancer cells produce more VIP

**Fig. 1 (abstract P376).**
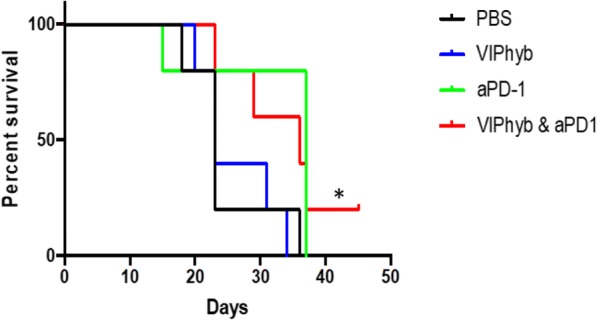
VIPhyb+anti-PD-1 in melanoma models

**Fig. 2 (abstract P376).**
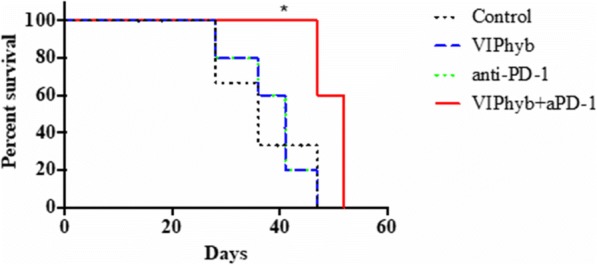
VIPhyb+anti-PD-1 in pancreatic cancer models

**Fig. 3 (abstract P376).**
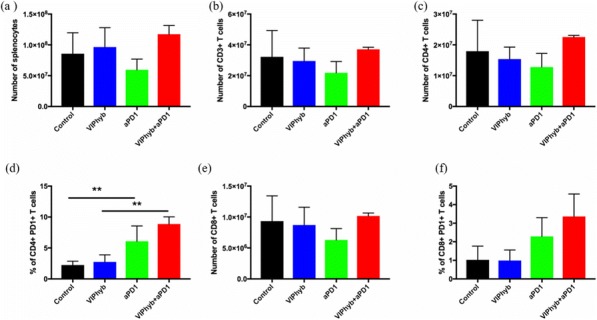
VIPhyb+anti-PD1 increases CD4+ PD1+ T cells

**Fig. 4 (abstract P376).**
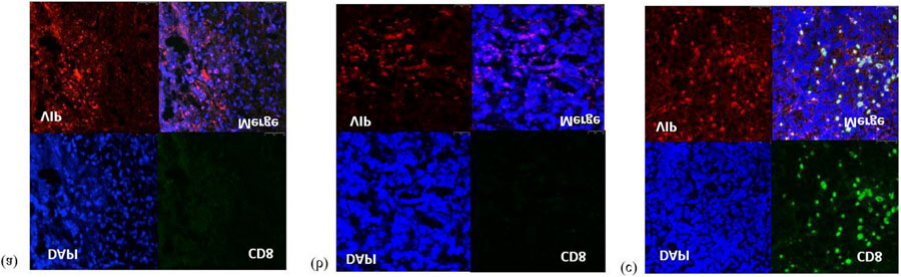
VIPhyb+ anti-PD-1 increases CD8+ T cells in tumor

#### P377 Combination immunotherapy anti-PD-1 anti-body (Ab) with CBT-101 (c-MET inhibitor) demonstrates enhanced activity in tumors not dependent on c-MET

##### Mamatha Reddy, Gavin Choy, PharmD, Sarath Kanekal, PhD, Sanjeev Redkar, PhD

###### CBT pharmaceuticals, Inc., Pleasanton, CA, USA

####### **Correspondence:** Sanjeev Redkar (sanjeev.redkar@cbtpharma.com)

**Background**

HGF/c-MET signaling mobilizes neutrophils in response to cancer immunotherapies. Neutrophils recruited to T-cell-inflamed microenvironments acquire immunosuppressive properties. c-MET+ neutrophils suppress therapy- induced T-cell expansion and effector functions. Glodde N et al [1] have shown that c-MET inhibition promoted adoptive T-cell transfer in murine cancer models by increasing effector T-cell infiltration in tumors. This therapeutic effect was independent of tumor cell-intrinsic c-MET dependence. In cancer patients, high serum levels of HGF correlated with high neutrophil counts and poor responses to checkpoint blockade therapies. Therefore, c-MET inhibitor (CBT-101) co-treatment may improve responses to cancer immunotherapy in settings beyond c-MET- dependent tumors.

**Methods**

Safety and efficacy of CBT-101, anti-PD-1Ab and combination were evaluated in three syngeneic mouse models, MC-38 (colorectal), H-22 (liver) and RENCA (renal). Tumor cells were inoculated in C57BL/6 mice and treatment was initiated when tumors reached a mean volume of approximately 100 mm3. Mice were randomized into four groups of ten animals per group and treated with either vehicle, CBT-101 (10 mg/kg oral daily in MC-38 and H-22 models and 20 mg/kg oral daily in RENCA), anti-PD-1 Ab (10 mg/kg intraperitoneally twice weekly), or a combination of CBT-101 plus anti-PD-1. Animals were checked daily for morbidity and mortality. Body weights (BW) and tumor volumes (TV) were measured twice weekly. In the MC-38 model, tumor tissue was collected at the end of the study and formalin fixed. Double IHC analysis of c-MET and neutrophils was used to quantify the expression of Met+ neutrophils.

**Results**

In MC-38 study, mean percent tumor growth inhibition (TGI) of combination demonstrated 65% tumor growth inhibition, versus 39% and 33% for anti-PD-1 and CBT-101 respectively. In H-22 study, there was no activity with CBT-101, 35% with anti-PD-1 and 60% TGI with combination. In RENCA study individual agents showed 60 to 65% activity while combination group demonstrated 80% TGI. The combination regimen was well tolerated by all animals with no loss in BW. Met+ neutrophils were significantly increased in the anti-PD-1 Ab group and dropped to the levels of vehicle group with combination treatment.

**Conclusions**

CBT-101 and Anti-PD-1 Ab combination treatment enhances host anti-tumor response in murine tumor models. Encouraged by these results, a Phase 1/2 clinical trial has been initiated to establish a safe dose combination of CBT-501 + CBT-101 primarily and nivolumab + CBT-101, secondarily in select solid tumors.

**Trial Registration**

Pending

**References**

1. Glodde N et al: Reactive Neutrophil Responses Dependent on the Receptor Tyrosine Kinase c-MET Limit Cancer Immunotherapy, Immunity, 47: p789–802, 2017

#### P378 NKTR-214 (CD122-biased agonist) and NKTR-262 (TLR7/8 agonist) combination treatment pairs local innate immune activation with systemic CD8+ T cell expansion to enhance anti-tumor immunity

##### Annah Rolig, Ph D^2^, Daniel Rose, BS in biology^2^, Saul Kivimäe^3^, Deborah Charych, PhD^3^, Werner Rubas, PhD^3^, Jonathan Zalevsky, PhD^3^, William L. Redmond, PhD^2^

###### ^1^Earle A. Chiles Research Institute, Robert W. Franz Cancer Center, Portland, OR, USA; ^2^Earle A. Chiles Research Institute, Portland, OR, USA; ^3^Nektar Therapeutics, San Francisco, CA, USA

####### **Correspondence:** William L. Redmond (william.redmond@providence.org)

**Background**

Radiation therapy (RT) remains the standard of care for many human cancers. Combining NKTR-214, a CD122-biased cytokine agonist conjugated with releasable polyethylene-glycol (PEG) chains, with local RT significantly enhanced therapeutic efficacy in preclinical models. Mechanistically, NKTR-214 provides sustained signaling through the IL-2 receptor pathway (IL-2Rβγ) to preferentially activate and expand effector CD8+ T and NK cells and RT modulates the tumor microenvironment (TME) to induce antigen-release. Together, NKTR-214/RT treatment resulted in improved therapeutic responses compared to either treatment alone. However, abscopal responses in murine tumors were modest, leading us to explore alternative approaches with the potential to elicit more robust tumor-antigen specific responses. In the current study, we evaluated the extent to which NKTR-262, a polymer-modified TLR7/8 agonist prodrug, modulates the TME and synergizes with NKTR-214 treatment. We hypothesized that NKTR-214/NKTR-262 immunotherapy would promote synergistic activation of immunostimulatory innate immune responses along with systemic adaptive anti-tumor responses to significantly improve abscopal responses, tumor regression, and overall survival.

**Methods**

Tumor-bearing mice (CT26; 4T1) received NKTR-214 (0.8 mg/kg; iv), RT (16 Gy x 1), and/or intratumoral NKTR-262 (0.5 mg/kg). The activation status of CD4+, CD8+, and NK cells in the blood, lymph node, and/or tumor (7 days post-treatment) was evaluated by flow cytometry. Effects on innate immune subsets (macrophages, monocytes) including M1/M2 polarization were evaluated by flow cytometry and immunohistochemistry (1 day post-treatment). Data represents the result of 1-2 independent experiments (n=5-14/group). For immune markers, statistical significance was determined using a 1-way ANOVA with a p-value cut-off of 0.05.

**Results**

NKTR-214/RT resulted in increased absolute lymphocyte counts and expression of T cell activation markers (Ki-67, PD-1, granzyme A) in the blood and tumor. Compared to NKTR-214/RT, NKTR-214/NKTR-262 resulted in significantly improved survival (p<0.05) and expansion of activated CD8+ T cells (GzmA+; Ki-67+; ICOS+; PD- 1+) in the blood (p<0.05). In the tumor, both combination treatments resulted in a similar CD8+ T cell density. NKTR-262/NKTR-214 induced higher frequencies of GzmA+ CD8+ T cells exhibiting reduced expression of suppressive checkpoint receptors PD-1+ and TIM-3+ (p<0.05). The increased CD8+ T cell differentiation was associated with a significant increase in M1 monocytes (p<0.05) and reduced presence of M2 monocytes.

**Conclusions**

Combined NKTR-214/NKTR-262 therapy induced robust anti-tumor immunity characterized by systemic CD8+ T cell expansion, enhanced intratumoral CD8+ T cell effector function, and favorable myeloid polarization resulting in improved tumor regression and tumor-free survival.

#### P379 Nanobodies® as a platform for multispecific targeting of immunomodulatory receptors

##### Anandi Sawant, PhD^1^, Douglas Linn, PhD^2^

###### ^1^Merck & Co., Palo Alto, CA, USA; ^2^Merck & Co, Boston, MA, USA

####### **Correspondence:** Anandi Sawant (anandi.sawant@merck.com)

**Background**

While the clinical successes of immunotherapeutic antibodies targeting PD-1 and CTLA-4 have revolutionized the treatment of cancer, a majority of patients still fail to achieve objective responses. Tumors use several mechanisms to escape immune surveillance, and emerging clinical data suggest that targeting multiple immunomodulatory pathways can provide significant benefit over monotherapy. Nanobodies® (Nbs) are antibody-like therapeutic proteins based on immunoglobulin single variable domains.

**Methods**

Several Nbs can be linked together for multispecific targeting. In collaboration, Merck & Co., Inc. and Ablynx have developed bispecific PD-1/LAG-3 Nbs consisting of either one or two anti-PD-1 and LAG-3 modules (LAG- 3mono/PD-1mono, LAG-3bi/PD-1bi) linked to an albumin-binding module for half-life extension.

**Results**

Nb panels were triaged through a number of in vitro binding, blocking, and functional assays to find highly potent PD-1 or LAG-3 leads that were then benchmarked against respective antibodies (Abs). In vivo, bispecific PD- 1/LAG-3 Nbs demonstrated notable anti tumor effects in multiple tumor models. In certain models, both bispecific PD-1/LAG-3 Nbs performed favorably relative to the combination of anti-PD-1 and anti-LAG-3 antibodies. Interestingly, a bispecific Nb with single modules for each target (LAG-3mono/PD-1mono) was as efficacious as a bispecific Nb that allowed for avid bivalent interactions with each target (LAG-3bi/PD-1bi). Both bispecific Nbs induced infiltration and activation of T cells within tumors and the expression of anti-tumor immunity-associated genes.

**Conclusions**

Targeting two inhibitory immunomodulatory receptors with a single drug holds promise of achieving clinical benefit in patients that have failed monotherapy options.

#### P380 Treatment-free survival (TFS), with and without immunomodulatory medications (IMMs), as a novel outcome applied to immuno-oncology (IO) agents in advanced melanoma

##### Meredith Regan, PhD^1^, Lillian Werner^1^, Ahmad Tarhini, MD, PhD^2^, Sumati Rao, PhD^3^, Komal Gupte-Singh, PhD^3^, Corey Ritchings, PharmD^3^, Michael Atkins, MD^4^, David McDermott, MD^5^

###### ^1^Dana-Farber Cancer Institute, Boston, MA, USA; ^2^Cleveland Clinic Taussig Cancer Institut, Cleveland, OH, USA; ^3^Bristol-Myers Squibb, Princeton, NJ, USA; ^4^Georgetown Lombardi Comprehensive Cance, Washington, DC, USA; ^5^Beth Israel Deaconess Medical Center, Boston, MA, USA

####### **Correspondence:** Meredith Regan (mregan@jimmy.harvard.edu)

**Background**

Patients discontinuing IO agents may experience periods of disease control without the need for subsequent systemic anticancer therapy, but may still require IMMs. This study simultaneously characterizes the disease control and IMM use during the treatment-free period.

**Methods**

Pooled data from the CheckMate 067 (phase 3) and 069 (phase 2) trials of nivolumab plus ipilimumab (NIVO+IPI; N=407), nivolumab (NIVO; N=313), and ipilimumab (IPI; N=357) for advanced melanoma were analyzed. IPI was given for 4 doses and NIVO was given until progression or unacceptable toxicity. Persistent use of IMMs and duration of IMM use initiated after IO protocol therapy cessation associated with any-grade treatment-related adverse events were included in the assessment. TFS was defined as the area between two Kaplan-Meier curves for conventional time-to-event endpoints from randomization: (A) time to IO protocol therapy cessation and (B) time to subsequent therapy or death [1]. TFS was subdivided as TFS with and without IMM use by a third endpoint: (C) time to cessation of both IO protocol therapy and IMM use. Area under each Kaplan-Meier curve was estimated by the 36-month restricted mean time-to-event. Area under the overall survival (OS) curve was partitioned as survival after subsequent therapy initiation (mean OS-B), TFS (mean B-A), time on IO protocol therapy (mean A), and as TFS with (mean C-A) and without (mean B-C) IMMs. Each area was summarized by its percentage of the 36-month period.

**Results**

At 36 months, 58% of NIVO+IPI, 52% of NIVO, and 36% of IPI patients were alive. Few patients remained on protocol therapy (11% NIVO+IPI, 17% NIVO, and 0% IPI) and many were surviving free of subsequent therapy initiation (47% NIVO+IPI, 37% NIVO, and 15% IPI). The 36-month restricted mean OS was longer for NIVO+IPI and NIVO than IPI (Table 1). Patients on NIVO spent a longer time on protocol therapy compared with NIVO+IPI or IPI. Mean TFS was longest for NIVO+IPI at 31% of the period; 13% with and 18% without IMMs. TFS without IMMs was longer for NIVO+IPI than NIVO (+3.2 months; 95% CI 1.7, 4.7) and shorter than IPI (-0.7 months; 95% CI -2.5, 1.2).

**Conclusions**

TFS, defined by the area between Kaplan-Meier curves for time to IO protocol therapy cessation and time to subsequent therapy or death, was longer for NIVO+IPI compared with NIVO or IPI in patients with advanced melanoma. When IMM use was considered, TFS without IMMs was longer for NIVO+IPI than NIVO.

**References**

1. Regan MM, Werner L, Tarhini A, et al. Treatment-free survival, a novel outcome applied to immune-oncology agents in advanced melanoma. Presented at ASCO June 1-5, 2018; abstract 9531.

**Table 1 (abstract P380).**
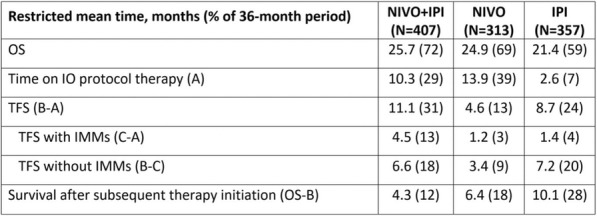
See text for description.

#### P381 Schweinfurthin natural products promote immune-dependent regression of murine melanoma and improve anti-PD-1-based immunotherapy to achieve durable complete responses and protective immunity

##### Kathleen Kokolus, MS, PhD^1^, Jeremy Haley, MS^1^, Emily Koubek^1^, Raghavendra Gowda, PhD^1^, Saketh Dinavahi, PhD^1^, Arati Sharma, PhD^1^, David Claxton, MD^2^, Klaus Helm, MD^1^, Joseph Drabick, MD^2^, Gavin Robertson, PhD^1^, Jeffrey Neighbors, PhD^1^, Raymond Hohl, MD^2^, Todd Schell, PhD^1^

###### ^1^Penn State College of Medicine, Hershey, PA, USA; ^2^Penn State Cancer Institute, Hershey, PA, USA

####### **Correspondence:** Todd Schell (tschell@psu.edu)

**Background**

Metastatic melanoma is a significant clinical problem with a 5-year survival rate of 15-20%. Recent approval of new immunotherapies and targeted inhibitors have improved the prognosis for these patients. In particular, antibody- based therapies that block the PD-1/PD-L1 checkpoint inhibitory pathway have achieved an increased overall response rate of 30-40% in metastatic melanoma. However, durable complete response rates are reported only around 15%, highlighting the need to identify approaches that can increase the therapeutic efficacy. Schweinfurthins are a family of plant-derived products that demonstrate anticancer activity against a variety of tumor types. Specific mechanisms of activity remain only partially defined but these compounds are known to modulate cholesterol metabolism. Given recent studies demonstrating that immune-based approaches may synergize with cholesterol inhibiting drugs, we investigated how schweinfurthins may impact anti-PD-1-mediated immunotherapy using an aggressive murine melanoma model.

**Methods**

Two different schweinfurthin analogs were tested for concentration-dependent in vitro growth inhibition of three human melanoma cell lines as well as the murine B16.F10 melanoma cells. B16.F10 cells treated with increasing concentrations of schweinfurthins were evaluated for surface expression of calreticulin using flow cytometry. Groups of B16.F10 melanoma-bearing C57BL/6 mice were administered schweinfurthin analogs for five consecutive days with or without administration of anti-PD-1 antibody twice a week for three weeks. Tumor growth and mouse survival was monitored. H&E stains were used to evaluate tumor regression. The impact of schweinfurthin administration on immune cell composition was determined by flow cytometry.

**Results**

Two schweinfurthin compounds differentially reduced the growth of human and murine melanoma cells in vitro and induced plasma membrane surface localization of the ER-resident protein calreticulin in B16.F10 melanoma cells, an indicator of immunogenic cell death. Both compounds improved anti-PD-1-mediated immunotherapy of established tumors in immunocompetent C57BL/6 mice either by delaying tumor progression or resulting in complete tumor regression. A 5-day course of schweinfurthin alone was associated with transient tumor regression in the absence of anti-PD-1 and this initial regression required an intact immune system as tumors were unaffected in NOD scid gamma (NSG) mice.

**Conclusions**

Schweinfurthins promote an immune-dependent initial tumor regression and improve the efficacy of anti-PD-1 therapy, leading to enhanced and durable anti-tumor immunity. This combination approach can potentially be translated to improve outcomes for metastatic melanoma patients treated with anti-PD1 therapy.

**Acknowledgements**

Supported, in part, by the Rose Dunlap Endowment and the Pennsylvania DOH using Tobacco CURE Funds (SAP #4100072562). KMK was supported by National Cancer Institute/National Institutes of Health training grant T32 CA060395. Schweinfurthin compounds were provided by Terpenoid Therapeutics Inc.

**Ethics Approval**

Animal studies were performed in accordance with institutional guidelines under protocol #46864 approved by the Penn State College of Medicine Institutional Animal Care and Use Committee

#### P382 CD28-negative memory tumor infiltrating lymphocytes (TILs) maintain an activated cytotoxic phenotype

##### Lillian Seu, PhD^1^, Bijal Kakrecha, BS^1^, Paul Fischer, MSc^1^, Malinda Aitken^2^, Alice Tang^3^, Steven Nadler, PhD^2^, Laurence Menard, PhD^2^

###### ^1^Bristol-Myers Squibb, ewing, NJ, USA; ^2^Bristol Myers Squibb, Princeton, NJ, USA; ^3^University of Pennsylvania, Philadelphia, USA

####### **Correspondence:** Laurence Menard (laurence.menard@bms.com)

**Background**

CD28 is constitutively expressed on human T cells, and promotes survival, proliferation signals, and induces the T cell growth factor interleukin-2. Enrichment of the senescent CD28-negative T cell population is a feature of an aging immune system, and is accelerated in various inflammatory conditions caused by chronic infections and/or autoimmune conditions. Recently, it has been shown that PD1 counteracts CD28 function via active de- phosphorylation of the cytoplasmic tail of CD28. Due to the repression by the critical immune checkpoint (IC) target PD-1, an understanding of CD28 regulation of tumor T cells would greatly aid in reinvigoration strategies with PD1/PDL1-based immunotherapies.

**Methods**

Flow cytometry of immune cells from freshly isolated tumors and matching peripheral blood was conducted across several tumor indications (renal cell carcinoma, non-small cell lung carcinoma, melanoma, colorectal cancer, head and neck squamous cell carcinoma, and others.) Boolean gating as well as high dimensionality tSNE analyses were performed to assess CD28 expression levels on CD4+ and CD8+ T cells, along with memory (CD45RO, CCR7), cytotoxic (CD107a), and activation (CD38) markers. (Figure 1, 2)

**Results**

In this report, we show that CD28 expression levels are diminished in TILs as compared to matching peripheral T cells. Concordant with observations in peripheral T cells, tumor associated CD4+ T cells retain higher levels of CD28 expression than CD8+ T cells. We additionally show that effector memory CD4+ TILs have highly correlated expression levels of CD28 and PD1 (CD4 Effector memory: Spearman r=0.62, P=0.001), while not true of the CD8 effector memory compartment in tumors (Spearman r=0.13, P=ns). Moreover, follow-up tSNE analysis revealed in a subset of tumors (e.g., renal cell carcinoma) a population of CD8+ CD45RO+ CD28-negative memory T cells with a CD107a (cytotoxic) and CD38 (activated) phenotype. Longitudinal immunophenotyping of PD1/PDL1-treated donors reveal extensive variability in CD28+ T cell frequencies across all peripheral T cell memory subsets.

**Conclusions**

We report an assessment of CD28 profiling in various tumor indications that show that CD28 expression is most significantly lost on central memory and effector memory CD8+ T cells in the tumor. Despite the loss of this critical co-stimulatory receptor, a cytotoxic profile (CD107a) is retained on CD28-negative CD8+ T cells across many tumor indications. Studies are currently aimed at understanding which agonist signals are sufficient to maintain and activated phenotype of these CD28 negative memory T cell populations.

**Ethics Approval**

The study was approved by BMS Institutution‘s Ethics Board

**Fig. 1 (abstract P382).**
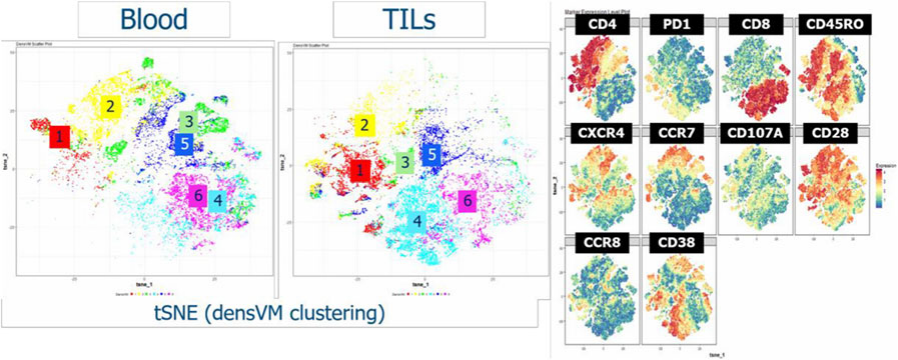
tSNE T cell analysis: matching Blood and TILs

**Fig. 2 (abstract P382).**
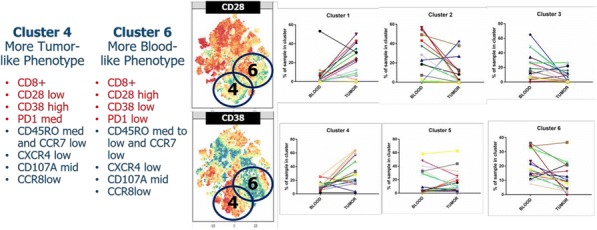
tSNE overlay using machine-learning approaches

#### P383 Combination therapy with M7824 (MSB0011359C) and NHS-muIL12 enhances antitumor efficacy in preclinical cancer models

##### Colleen Stanton^1^, Chunxiao Xu, PhD^2^, Bo Marelli^2^, Jin Qi^2^, Guozhong Qin^2^, Huakui Yu^2^, Molly Jenkins^2^, Kin-Ming Lo^2^, Joern-Peter Halle^2^, Yan Lan, MD^2^

###### ^1^Nucleus Global; ^2^EMD Serono Research and Development, Belmont, MA, USA

####### **Correspondence:** Yan Lan (yan.lan@emdserono.com)

**Background**

PD-1/PD-L1 pathway inhibition is a clinically validated approach in cancer therapy. However, most patients do not respond to the monotherapy due to multiple immunosuppressive mechanisms. Combining anti-PD-1/PD-L1 with other immunotherapeutic agents targeting additional immunomodulatory pathways in the tumor microenvironment (TME) is one strategy to overcome resistance and improve response rates. M7824 is an innovative first-in-class bifunctional fusion protein composed of two extracellular domains of TGF-β receptor II (a TGF-β “trap”) fused to a human anti-PD-L1 IgG1 monoclonal antibody. Through simultaneous blockade of the PD-L1 and TGF-β pathways, M7824 demonstrated enhanced anti-tumor activity in preclinical models [1]. NHS-IL12, and the surrogate NHS- muIL12, are immunocytokines designed to target tumor necrotic regions to deliver IL-12 into the TME, where they can activate NK cells and CD8+ T cells to increase their cytotoxic functions. The surrogate NHS-muIL12 has demonstrated antitumor efficacy in preclinical models [2]. This study is designed to investigate whether M7824 treatment may further benefit from combination therapy with NHS-muIL12.

**Methods**

Mice bearing MC38, EMT-6, or 4T1 tumors were treated with M7824, NHS-muIL12, or combination therapy. Tumor growth and survival were assessed in each model, and tumor recurrence following remission and rechallenge was evaluated in the EMT-6 model. Immune cell populations in the spleens and tumors were evaluated by flow cytometry and the frequency of tumor antigen-reactive IFNγ-producing CD8+ T cells was evaluated by an ELISpot assay in the MC38 model.

**Results**

Combination of M7824 and NHS-muIL12 enhanced antitumor activity and extended the survival relative to either monotherapy in preclinical tumor models. Combination therapy also enhanced the proliferation, infiltration, and cytotoxicity of CD8+ T cells relative to monotherapies. In addition, the combination therapy increased the frequency of tumor antigen-reactive T cells and induced the generation of tumor-specific immune memory, as demonstrated by protection against tumor rechallenge.

**Conclusions**

These data demonstrate that combination therapy with M7824 and NHS-muIL12 improved anti-tumor efficacy in multiple preclinical tumor models and suggest that combining these therapies may be a promising therapeutic strategy for patients with solid tumors.

**References**

1. Lan Y, et al. Enhanced preclinical antitumor activity of M7824, a bifunctional fusion protein simultaneously targeting PD-L1 and TGF-β. Sci Trans Med. 2018;10(424).

2. Fallon J, et al. The immunocytokine NHS-IL12 as a potential cancer therapeutic. Oncotarget 2014;5(7):1869-84.

#### P384 M7824 (MSB0011359C) is an effective combination partner with standard-of-care therapies in tumor models

##### Yan Lan, MD^2^, Rinat Zaynagetdinov, MD, PhD^2^, Kenneth Hance^4^, Chia Lin Chu, PhD^5^, Chunxiao Xu, PhD^2^, Bo Marelli^2^, Jin Qi^2^, Guozhong Qin^2^, Huakui Yu^2^, Giorgio Kradjian^2^, Yanping Zhang^2^, Molly Jenkins^2^, Joern-Peter Halle^2^, Kin-Ming Lo^2^

###### ^1^Nucleus Global; ^2^EMD Serono Research and Development, Billerica, MA, USA; ^3^EMD Serono Research & Development, Billerica, MA, USA; ^4^GlaxoSmithKline, Collegeville, PA, USA; ^5^EMD Serono Research and Development Inst, Billerica, MA, USA

####### **Correspondence:** Yan Lan (yan.lan@emdserono.com)

**Background**

We have recently reported enhanced preclinical anti-tumor activity of M7824, which is an innovative first-in-class bifunctional fusion protein composed of two extracellular domains of TGF β receptor II (a TGF-β “trap”) fused to a human anti–PD-L1 IgG1 monoclonal antibody [1]. In Phase 1 and Phase 1b expansion studies in patients with advanced solid tumors, M7824 has shown early evidence of clinical activity [2]. This study is designed to investigate the efficacy of M7824 in combination with standard-of-care therapies in different murine models.

**Methods**

Combination of M7824 with Ox/5-FU was tested in the MC38 colorectal cancer model, with cisplatin, doxorubicin, radiation therapy (RT), or anti-VEGF A (B20) in the 4T1 breast cancer model, with gemcitabine in the MB49 bladder cancer model, with pazopanib in the RENCA renal carcinoma model, and with anti-CTLA4 in the B16 melanoma model.

**Results**

Combination of M7824 and Ox/5-FU significantly decreased tumor volume and weight in the MC38 model and increased the frequency of p15E-reactive, IFNγ-producing CD8+ T cells. In the 4T1 model, combination of M7824 and doxorubicin or RT, enhanced tumor growth inhibition and extended survival, and combination of M7824 and cisplatin or anti-VEGF A likewise decreased tumor volume relative to monotherapies. Combination of M7824 and pazopanib significantly decreased tumor weight in the orthotopic RENCA model. Finally, combination of M7824 with anti-CTLA4 significantly decreased tumor volume in the B16 model.

**Conclusions**

Taken together, these results support the combination of M7824 with conventional standard-of-care therapies. The enhanced efficacy seen in multiple murine models also supports the broad application of M7824 combination therapies in different cancer indications.

**References**

1. Lan Y, et al. Enhanced preclinical antitumor activity of M7824, a bifunctional fusion protein simultaneously targeting PD-L1 and TGF-β. Sci Trans Med. 2018;10(424).2. Strauss J, et al. phase I trial of M7824 (MSB0011359C), a bifunctional fusion protein targeting PD-L1 and TGFβ, in advanced solid tumors. Clin Cancer Res. 2018;24(6):1287-1295.

#### P385 Preliminary biomarker analysis of sitravatinib in combination with nivolumab in NSCLC patients progressing on prior checkpoint inhibitor

##### Vanessa Tassell^1^, James Christensen^1^, Peter Olson^1^, Igor Rybkin, MD, PhD^2^, Ticiana Leal, MD^3^, Alexander Spira, MD, PhD, FACP^4^, Collin Blakely, MD^5^, Manish Patel, DO^6^, Leora Horn, MD^7^, Kai He, MD, PhD^8^, David Berz, MD^9^, Ryan Ramaekers, MD^10^, Alison Savage^11^, Timothy Larson^12^, Donald Richards^13^, Tammy Roque^14^, Anthony Pham^15^, Massarelli Erminia^16^, James Uyeki^17^, Abhinav Chandra, MD, FACP^18^, Robert Jotte^19^, Wangjian Zhong, MD^20^, David Hong, MD^21^, Joshua Lang, MD MS^3^, Jennifer Schehr^3^, Julio Fernandez Banet^22^, Kai He, MD, PhD^23^, Adam Pavlicek^22^

###### ^1^Mirati Therapeutics, San Diego, CA, USA; ^2^Henry Ford Health System, Detroit, MI, USA; ^3^University of Wisconsin, Madison, WI, USA; ^4^Virginia Cancer Specialists, Fairfax, VA, USA; ^5^University of San Francisco, San Francisco, CA, USA; ^6^University of Minnesota Masonic Cancer C, Minneapolis, MN, USA; ^7^Vanderbilt University, Nashville, TN, USA; ^8^The Ohio State University Comprehensive, Columbus, OH, USA; ^9^Beverly Hills Cancer Center, Beverly Hills, CA, USA; ^10^Saint Francis Cancer Treatment Center, Grand Island, NE, USA; ^11^Hematology Oncology Associates - Barnett, Medford, OR, USA; ^12^Minnesota Oncology Hematology, Minneapolis, MN, USA; ^13^Texas Oncology - Longview Cancer Center, Houston, TX, USA; ^14^Texas Oncology, Sherman, TX, USA; ^15^Northwest Cancer Specialists, P.C., Tualatin, OR, USA; ^16^City of Hope - Duarte, Duarte, CA, USA; ^17^Texas Oncology - South Austin, Austin, TX, USA; ^18^Yuma Regional Cancer Center, Yuma, AZ, USA; ^19^Rocky Mountain Cancer Centers - Denver, Denver, CO, USA; ^20^Baptist Health, Louisville, KY, USA; ^21^MD Anderson, Houston, TX, USA; ^22^Monoceros Biosystem Inc., San Diego, CA, USA; ^23^x, Columbus, OH, USA

####### **Correspondence:** Ticiana Leal (tbleal@medicine.wisc.edu)

**Background**

Sitravatinib is a spectrum-selective tyrosine kinase inhibitor which targets TAM receptors (Axl, MER), VEGFR2, KIT and MET. Inhibition of these receptors may enhance anti-tumor activity of nivolumab by modifying the tumor microenvironment and enhancing a T cell-mediated anti-tumor immune response. Sitravatinib in combination with nivolumab has demonstrated signs of clinical activity in advanced non-squamous NSCLC patients (pts) who have become refractory to prior checkpoint inhibitor therapy (CIT). Understanding the mechanism of action and molecular characteristics of responding patients is critical and we report on the preliminary biomarker analysis.

**Methods**

Study objectives include evaluation of safety, efficacy, and correlative science endpoints in pts who have progression of disease (PD) on or after CIT. Sitravatinib is administered orally in continuous 28- day cycles; nivolumab is administered intravenously 240 mg every 2 weeks. Key objectives include overall response rate, safety, evaluation of tumor PD-L1 expression, tumor mutation profile, and circulating and tumor infiltrating immune cell populations.

**Results**

As of June 26, 2018, the CIT-experienced cohorts enrolled 64 pts and 46/64 have had at least one on-study tumor assessment. Clinical benefit (CB) defined as confirmed partial response (PR) and stable disease (SD) of >/= 14 weeks has been seen in 19/46 pts. Evaluation of tumor PD-L1 expression indicated a trend toward a positive PD-L1 result in pts with CB compared with pts with no CB (PD or SD < 14 weeks). Profiling of tumor mutation status in plasma (GuardantOMNITM) indicated no significant correlation of tumor mutation burden (TMB) with CB. Interestingly, pts exhibiting STK11 (LKB1) mutations (3) all demonstrated PD as best response. Baseline and post-treatment (C1D15) analysis of selected circulating immune cell populations in peripheral blood were evaluated utilizing flow cytometry. A significant increase in T-effector cells (CD8+/CD4-/CD3+/CD45RA/CD62L-) at C1D15 was observed in subsets of pts with CB compared with pts with no CB. In an exploratory analysis, MHC I expression in circulating tumor cells (CTCs) was evaluated. In one patient, baseline CTCs showed negative MHC I expression at PD and subsequently had increased expression of MHC I on CTCs after treatment with sitravatinib and nivolumab, which correlated with 13.3% reduction in target lesions. Additional baseline and post-treatment analyses will be included.

**Conclusions**

The combination of sitravatinib with nivolumab is clinically active in pts progressing on prior CIT regimens. Preliminary analysis of correlative endpoints suggests that PD-L1 positivity and induction of a T-effector cell response may correlate with clinical outcome.

**Ethics Approval**

This study was approved by Copernicus (WIRB), approval number PRA0-16-311.

#### P386 CTX-471, a novel agonistic antibody targeting CD137, enhances the anti-tumor activity of tumor antigen- targeted antibodies and immune checkpoint inhibitors when used in combination

##### Ugur Eskiocak, PhD^1^, Wilson Guzman, BS^1^, Robert Tighe, BS^1^

###### ^1^Compass Therapeutics, Cambridge, MA, USA; ^2^Compass Therapeutics LLC, Cambridge, MA, USA

####### **Correspondence:** Robert Tighe (robert.tighe@compasstherapeutics.com)

**Background**

CD137 (4-1BB) is a member of the TNFR superfamily that provides costimulatory signals to activated cytotoxic lymphocytes. CTX-471 is a novel agonistic antibody that recognizes a unique epitope of CD137 that is shared by human, cynomolgus monkey, and mouse. CTX-471 displays a wide therapeutic window in preclinical studies, making it a promising agent for clinical IO combinations. Prior reports have identified CD137 agonists as effective combination partners for tumor antigen-targeted antibodies and immune checkpoint inhibitors (ICIs) [1,2].

**Methods**

Combination study of CTX-471 plus trastuzumab (IgG1 anti-Her2) or cetuximab (IgG1 anti-EGFR) in immunocompetent mice was conducted with CT26 murine CRC cells engineered to express the human targets. To circumvent spontaneous tumor rejection, the cell lines were inoculated into Balb/c-SCID mice followed by adoptive transfer of Balb/c splenocytes and initiation of treatment five days later. In the CTX-471 plus cetuximab study, FACS-based immunophenotyping was performed. CTX-471 was tested in combination with PD-L1 blockade (CTX- PD-L1) in the EMT-6 orthotopic breast carcinoma model and the C1498 disseminated AML model. Combination with anti-PD-1 was assessed in the PANC-02 orthotopic tumor model.

**Results**

CTX-471 combination with trastuzumab was synergistically effective against CT26-huHer2 tumors curing 8/8 mice, while CTX-471 monotherapy achieved 3/8 cures and trastuzumab monotherapy was ineffective. CTX-471 combination with cetuximab had synergistic activity against CT26-huEGFR tumors, curing 4/8 mice. CTX-471 and cetuximab lacked monotherapy efficacy in this model. CTX-471 combined with cetuximab caused an increase in CD8+ TILs and a decrease in Treg, positively shifting the CD8/Treg ratio. Significant increases in MHC class II+ M1 macrophages was also observed. We compared sequential vs. concomitant dosing schedules of CTX-471 plus ICI. In the EMT6 tumor model, CTX-471 plus CTX-PD-L1 showed enhanced efficacy when given sequentially, whereas concomitant dosing negatively impacted efficacy. In the PANC02 model, CTX-471 plus anti-PD-1 showed improved efficacy that was unimpacted by dosing schedule. In the disseminated C1498 AML model both dose schedules of CTX-471 plus CTX-PD-L1 synergistically improved overall survival, with concomitant dosing curing 30% of the animals. All cured mice rejected tumors upon rechallenge.

**Conclusions**

Our promising preclinical results support the clinical testing of CTX-471 in combination with approved antigen-targeted antibodies and ICIs. Interestingly, the sequential dosing of costimulatory agonists and ICIs appears to be important in some, but not all, preclinical models. Understanding the clinical implications of this observation will require further research. IND-enabling toxicology studies for CTX-471 are ongoing and a Phase 1 clinical trial is planned for 2019.

**References**

1. Kohrt HE, Houot R, Weiskopf K, et al. Stimulation of natural killer cells with a CD137-specific antibody enhances trastuzumab efficacy in xenotransplant models of breast cancer. J Clin Invest. 2012;122(3):1066-75.

2. Shindo Y, Yoshimura K, Kuramasu A, et al. Combination immunotherapy with 4-1BB activation and PD-1 blockade enhances antitumor efficacy in a mouse model of subcutaneous tumor. Anticancer Res. 2015;35(1):129-36.

**Ethics Approval**

The described animal studies were approved by Compass Therapeutics Institutional Animal Care and Use Committee under protocol CTX-016-01.

#### P387 A multicenter, phase II study in patients with first line NSCLC, or recurrent PD-X refractory NSCLC or with recurrent HNSCC receiving Eftilagimod Alpha in combination with Pembrolizumab (TACTI-002)

##### Frederic Triebel, MD, PhD^1^, Christian Mueller, MSc^1^, Chrystelle Brignone, PhD^1^, Tatsiana Skrahina^1^, Martin Forster^2^

###### ^1^Immutep, Orsay, France; ^2^UCL Cancer Institute, London, UK

####### **Correspondence:** Frederic Triebel (ftriebel@immutep.com)

**Background**

Eftilagimod alpha is a recombinant soluble LAG-3Ig fusion protein binding to MHC class II molecules and mediating antigen presenting cell (APC) activation followed by CD8 T-cell activation. Combining an APC activator with an immune checkpoint inhibitor (ICI) aims to increase efficacy without additional toxicity. A Phase I study (abstract number: P398 *Corresponding author email: ftriebel@immutep.com) has assessed the safety of the eftilagimod alpha plus pembrolizumab combination.

**Methods**

The study is designed according to Simon's optimal two-stage design, with objective response rate as primary endpoint. Secondary endpoints include progression free survival and overall survival. During the first stage of the study patients (pts) will be recruited into this multicenter, open label, Phase II study for each of three indications: A: 1st line, PD-X naïve NSCLC; B: 2nd line, PD-X refractory NSCLC; C: 2nd line PD-X naive HNSCC. In case there are more responses than a certain threshold observed in patients recruited in the initial stage (N1), additional patients (N2) will be recruited. In total 110 patients are planned to be enrolled. Eftilagimod alpha will be administered as 30 mg subcutaneous injection every 2 weeks for the first 8 pembrolizumab cycles (200 mg every 3 weeks) and every 3 weeks for the 9 following cycles (clinicaltrial.gov link to be added).

**Results**

Trial in progress

**Conclusions**

Trial in progress

**Trial Registration**

EudraCT: 2018-001994-25

**Fig. 1 (abstract P387).**
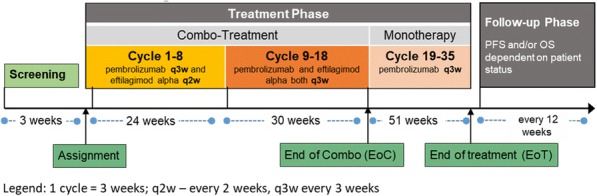
TACTI-002 trial design

#### P388 Combination of the Wee1 inhibitor adavosertib (AZD1775) with anti-PD-L1 shows activity in pre-clinical studies

##### Viia Valge-Archer, PhD, Matthew King, PhD, Chrysiis Michaloglou, PhD, Anisha Solanki, Jennifer Hare, PhD, Susan Critchlow, PhD

###### AstraZeneca, Little Chesterford, UK

####### **Correspondence:** Viia Valge-Archer (viia.valge-archer@astrazeneca.com)

**Background**

Inhibition of DNA damage repair (DDR) pathways may enhance anti-tumour immunity via immune recognition and response to increased DNA damage. Immune priming may be consolidated by combination with immune checkpoint blockade to further increase anti-tumour responses.Inhibition of Wee1 by adavosertib (AZD1775) can induce replication stress and aberrant entry into mitosis in cells, which can result in cell death via replication or mitotic catastrophe [1].

**Methods**

Human and murine T cell proliferation was measured as CTV dilution in response to anti-CD3 / anti-CD28 stimulation or mixed lymphocyte reaction in the presence or absence of compounds. Tumour cells were treated in vitro for 24 to 72 hours prior to assessment. Alterations in tumour or T cell phenotype were measured by flow cytometry or immunofluorescent microscopy.In vivo syngeneic tumour models were implanted subcutaneously into Balb/c (CT26) or C57/Bl6 (MC38) mice and treated with adavosertib or anti-murine PD-L1 at the indicated doses and schedules.

**Results**

Proliferation and viability of activated human T cells show dose-dependent inhibition by adavosertib treatment, along with increased S-phase gamma-H2AX expression and 53BP1 foci, which are markers of DNA damage. Similar effects were observed in both human and murine T cells, with proliferation IC50’s of 350 nM and 149 nM, respectively. Results of in vivo PD studies demonstrate that a 2-5-fold decrease in number or frequency of tumour infiltrating T cells under continuous adavosertib dosing can be mitigated by scheduling. Combination of adavosertib with anti-PD-L1 resulted in approx. 2-fold increased numbers of tumour infiltrating CD8+ T cells and significantly increased Ki67 expression of tumour infiltrating T cells over vehicle or anti-PD-L1 alone. Treatment with adavosertib also resulted in increases in tumour cytokine and chemokine gene expression consistent with increased immune priming and activation.Despite the impact of adavosertib on T cell proliferation, anti-tumour efficacy studies in a murine syngeneic tumour model do not show antagonism of anti-PDL1 activity by adavosertib. Rather, positive schedule-dependent combination anti-tumour activity of adavosertib + anti-PD-L1 is observed, including 3/10 complete responses (CR) with the combination using a schedule that showed no CR with either monotherapy.

**Conclusions**

Pre-clinical data provides support for further investigation of combinations of adavosertib plus immune checkpoint blockade. Studies are ongoing to explore further dose and scheduling, as well as molecular mechanisms underpinning combination activity, including interferon/STING and immunogenic pathways.

**References**

1. Matheson CJ, Backos DS, Reigan P. Targeting WEE1 Kinase in Cancer. Trends Pharmacol Sci. 2016; 37:872-881.

**Consent**

All studies were conducted in accordance with UK Home Office legislation, the Animal Scientific Procedures Act 1986 (ASPA) and with AstraZeneca Global Bioethics policy. All experimental work is outlined in project licence a which has gone through the AstraZeneca Ethical Review Process.

#### P389 Digital spatial profiling on uveal melanoma tissue treated with combined radiofrequency ablation and ipilimumab

##### Trieu My Van, PhD^1^, Lisette Rozeman, MD^2^, Sarah Warren, PhD^3^, Joseph Beechem, PhD^3^, John Haanen, MD PhD^2^, Christian Blank, MD, PhD^2^

###### ^1^NKI/Nanostring, Amsterdam, Netherlands; ^2^NKI, Amsterdam, Netherlands; ^3^Nanostring, Seattle, WA, USA

####### **Correspondence:** Christian Blank (C.blank@nki.nl)

**Background**

Uveal melanoma (UM) is a rare disease which shows limited response to anti-CTLA4 and anti-PD1 antibodies [1,3-6]. Preclinical experiments in murine melanoma model indicated a durable response with additional radiofrequent ablation (RFA) and CTLA-4 blockade by enhancing antigen presentation within the tumor area [7,8]. However, RFA and immunotherapy are not suitable for all melanoma patients and the incidences of adverse events (AE) also vary. Thus, personalized therapy would lower the risk for AE and predict the response rate after cancer therapy. Here, we used the novel digital spatial profiling (DSP) technology to characterize the protein profiles in UM patients before and upon combination therapy.

**Methods**

The SECIRA-UM trial is a phase 1b/2 study assessing the safety and efficacy of the combination of RFA and IPI in UM patients with at least 2 unresectable liver lesions. In phase 1b, patients underwent RFA of one liver lesion and received 4 courses of ipilimumab (IPI) to define the recommended dose for phase 2. Primary endpoints of phase 2 were confirmed objective response rate (ORR) and disease control rate (DCR) according to RECIST 1.1, secondary endpoints were progression free survival (PFS) and overall survival (OS).DSP technology uses a cocktail of primary antibodies conjugated to unique DNA oligos with a UV photocleavable linker. Oligos in the Region of interest (ROI) on formalin-fixed paraffin-embedded (FFPE) tissues are released via UV mediated linker cleavage and counted on the nCounter™ platform.

**Results**

The combination of RFA-IPI 10mg/kg slightly improved the median PFS and OS compared to RFA-IPI 3mg/kg. However, the most common RFA related toxicities, which were transient elevation of liver enzymes and flank pain, did not differ between the different dosing cohorts. To understand the response of UM after combination therapy we used the DSP technology to characterize the protein expression profiles in UM biopsies. Our DSP analysis provided a detailed profile of 39 proteins/biopsy. Here, we show the comparison of expression profiles in responding vs non- responding patients, before vs after combination treatment and between the different dosing cohorts.

**Conclusions**

Our trial results showed a safe but limited clinical activity of RFA-IPI 3mg/kg while RFA-IPI 10mg/kg had a higher toxicity rate but showed a trend towards longer overall survival. Moreover, usage of the DSP technologies strongly advances our knowledge of protein expression levels within the tumor area and will have a high impact on future trial designs.

**Trial Registration**

CA184-180/N11RFA

**References**

1. Krantz BA, Dave N, Komasubara KM, Marr BP, Carvajal RD. Uveal melanoma: epidemiology, etiology, and treatment of primary disease. Clin Ophthalmol. 2017; 11: 279-289.

2. Maio M, Danielli R, Chiarion-Sileni V, Pigozzo J, Parmiani G, Ridolfi R, De Rosa F, Del Vecchio M, Di Guardo L, Queirolo P, Picasso V, Marchetti P, De Galitiis F, Mandala M, Guida M, Simeone E, Ascierto PA. Efficacy and safety of ipilimumab in patients with pre-treated, uveal melanoma. Annals of Oncology. 2013; 2911-2915

3. Luke JJ, Callahan MK, Postow MA, Romano E, Ramaiya N, Bluth M, Giobbie-Hurder A, Lawrence DP, Ibrahim N, Ott PA, Flaherty KT, Sullivan RJ, Harding JJ, D'Angelo S, Dickson M, Schwartz GK, Chapman PB, Wolchok JD, Hodi FS, Carvajal RD. Clinical activity of ipilimumab for metastatic uveal melanoma: a retrospective review of the Dana-Farber Cancer Institute, Massachusetts General Hospital, Memorial Sloan-Kettering Cancer Center, and University Hospital of Lausanne experience. Cancer. 2013. 119(20):3687-95

4. Kelderman S, van der Kooij MK, van den Eertwegh AJ, Soetekouw PM, Jansen RL, van den Brom RR, Hospers GA, Haanen JB, Kapiteijn E, Blank CU. Ipilimumab in pretreated metastastic uveal melanoma patients. Results of the Dutch Working group on Immunotherapy of Oncology (WIN-O). Acta Oncol. 2013

5. Algazi AP, Tsai KK, Shoushtari AN, Munhoz RR, Eroglu Z, Piulats JM, Ott PA, Johnson DB, Hwang J, Daud AI, Sosman JA, Carvajal RD, Chmielowski B, Postow MA, Weber JS, Sullivan RJ. Clinical outcomes in metastatic uveal melanoma treated with PD-1 and PD-L1 antibodies. Cancer. 2016. 3344-3353

6. van der Kooij MK, Joosse A, Speetjens FM, Hospers GA, Bisschop C, de Groot JW, Koornstra R, Blank CU, Kapiteijn E. Anti-PD1 treatment in metastatic uveal melanoma in the Netherlands. Acta Oncol. 2017. 101-103.

7. den Brok MH, Sutmuller RP, van der Voort R, Bennink EJ, Figdor CG, Ruers TJ, Adema GJ. In situ tumor ablation creates an antigen source for the generation of antitumor immunity. Cancer Res. 2004. 4024-9

8. den Brok MH, Sutmuller RP, Nierkens S, Bennink EJ, Frielink C, Toonen LWJ, Boerman OC, Figdor CG, Ruers TJM, Adema GJ. Efficient loading of dendritic cells following cryo and radiofrequency ablation in combination with immune modulation induces anti-tumour immunity. Br J Cancer. 2006. 896–905

#### P390 Vasoactive Intestinal Peptide Antagonist Synergizing with PD1 Antibody Inhibits the Tumor Growth of Breast Cancer

##### Shuhua Wang, MD, Sruthi Ravindranathan, PhD, Yiwen Li, MS, Rebecca Pankove, MS, Parvin Forghani, Edmund K. Waller, MD, PhD

###### Emory University, Atlanta, GA, USA

####### **Correspondence:** Edmund K. Waller (ewaller@emory.edu)

**Background**

Breast cancer is known to be the most common cancer in women with annual incidence of 170 million globally [1]. Novel immunotherapeutic approaches that target the immunosuppressive tumor microenvironment (TME) are required to boost the endogenous immune response [2]. Several studies have shown that the inhibition of myeloid- derived suppressor cells (MDSCs) in the TME effectively promotes T cell immunity against breast cancer. We have previously shown that Silibnin, a natural flavonoid from milk thistle can effectively reduce MDSC accumulation in blood and tumor in 4T1 tumor bearing mice [3]. Additionally, in murine leukemia models, we have observed that inhibiting vasoactive intestinal peptide (VIP) signaling reduces tumor burden and increases survival. The current study thus explores the effect of inhibiting VIP signaling with/without checkpoint inhibitors in murine breast cancer model.

**Methods**

Balb/c mice were injected 2.5 x 105 4T1cells subcutaneously on the right flank on day 0 and treated with VIPhyb (subcutaneously), anti-PD-1 (aPD1) (intraperitoneally) or a combination of VIPhyb and anti-PD-1 (aPD1+VIPhyb) starting from day 1. Mice treated with PBS and IgG2a were considered as control. Tumor volume was measured every day and mice were euthanized either upon ulceration or when their tumors reached the IACUC endpoint (500mm3). Spleens were then harvested and analyzed for frequency of Ki67 or PD1 expressing T cells, NK cells, B cells and MDSCs.

**Results**

On day 13 and 14 post 4T1 inoculation, the combination treatment group had the smallest tumor burden (P<0.05, combination vs control group) (Fig 1). Higher frequencies of CD4+ PD1+ T cells were found in the combination group compared to control, anti-PD-1 or VIPhyb alone treated groups (p<0.05), consistent with result using the combination of VIPhyb and anti-PD1 in other solid tumor models studied in our lab. Higher levels of CD4+Ki-67+ cells were seen in the combination therapy treated group compared to control, aPD1 or VIPhyb treated group (p=NS). We observed a similar level of MDSC across all experimental groups (p=NS) (Fig 2).

**Conclusions**

Combination therapy with anti-PD-1 and VIPhyb treatment suppressed breast cancer growth and enhanced proliferation of antigen experienced CD4+ T cells. Inhibition of tumor growth was associated with increased numbers of antigen-experienced Ki67+ PD1+ T cells. Since silibinin has been previously shown to decrease the MDSC population, current experiments are testing the addition of silibinin to the combination therapy treatment.

**References**

1. Ferlay J, Soerjomataram I, Dikshit R, Eser S, Mathers C, Rebelo M, Parkin DM, Forman D, Bray F. Cancer incidence and mortality worldwide: sources, methods and major patterns in GLOBOCAN 2012. Int J Cancer. 2015 Mar 1; 136(5):E359-86. doi:10.1002/ijc.29210. Epub 2014 Oct 9.

2. Umansky V, Blattner C, Gebhardt C, Utikal J. The Role of myeloid-derived suppressor cells (MDSC) in cancer progression.Vaccines (Basel). 2016 Nov 3; 4(4).pii: E36. Review.

3. Forghani P, Khorramizadeh MR,Waller EK.. Silibinin inhibits accumulation of myeloid-derived suppressor cells and tumor growth of murine breast cancer. Cancer Medicine. 2014; 3(2):215-224.

**Ethics Approval**

The study was approved by Emory Institute Animal Care and Use Committee, protocol number 3000202

**Fig. 1 (abstract P390).**
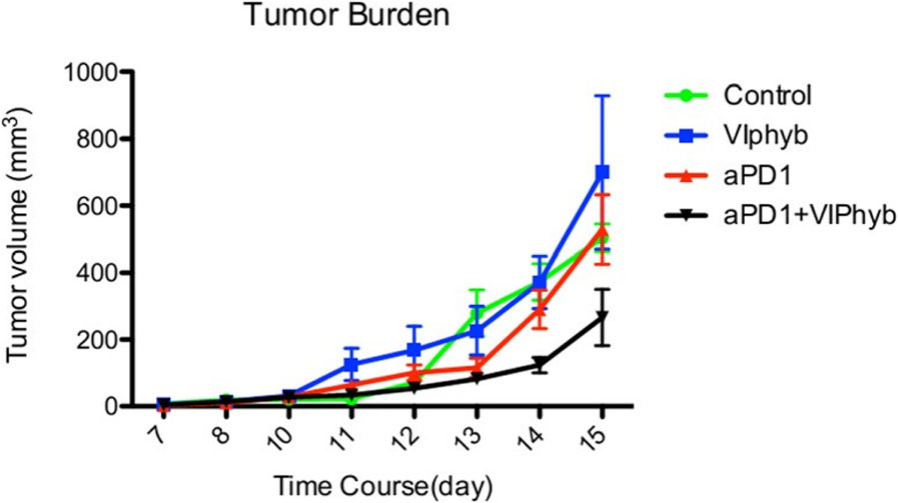
See text for description.

**Fig. 2 (abstract P390).**
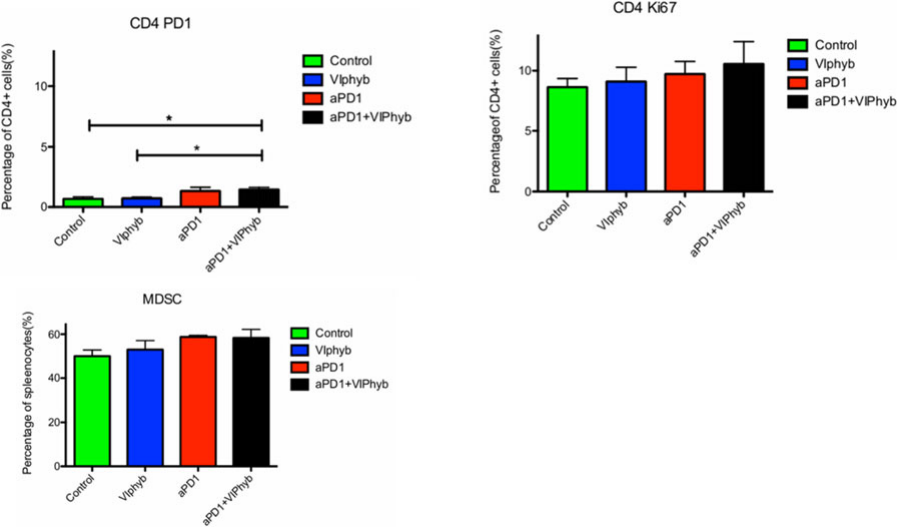
See text for description.

#### P391 A phase 1b/2 trial of lenvatinib in combination with pembrolizumab in patients with advanced melanoma

##### Matthew Taylor, MD^2^, Nicholas Vogelzang, MD^3^, Allen Cohn^3^, Daniel Stepan^4^, Robert Shumaker, PhD,^4^, Corina Dutcus^4^, Matthew Guo^4^, Emmett Schmidt, MD PhD^5^, Drew Rasco^6^

###### ^1^Oxford PharmaGenesis, Oxford, UK; ^2^Oregon Health and Science University, Portland, OR, USA; ^3^McKesson Specialty Health, Las Vegas, NV, USA; ^4^Eisai Inc., Woodcliff Lake, NJ, USA; ^5^Merck & Co., Inc., Kenilworth, NJ, USA; ^6^South Texas Accelerated Research Therape, San Antonio, TX, USA

####### **Correspondence:** Matthew Taylor (taylmatt@ohsu.edu)

**Background**

Lenvatinib is a multikinase inhibitor of VEGFR 1−3, FGFR 1−4, PDGFRα, RET, and KIT. Pembrolizumab, an anti-PD-1 antibody, is approved for the first-line treatment of patients with advanced melanoma, with objective response rates (ORR) of 21–34% [1,2]. Preclinical studies indicate that lenvatinib decreases the population of tumor- associated macrophages, increases CD8+ T cell infiltration, and augments the activity of PD-1 inhibitors; therefore, lenvatinib is a rational combination partner for pembrolizumab [3,4]. We report interim results of an ongoing phase 1b/2 trial evaluating lenvatinib in combination with pembrolizumab in patients with solid tumors, focusing on the advanced melanoma cohort.

**Methods**

In this multicenter, open-label study (NCT02501096), patients with measurable, confirmed, metastatic melanoma and ECOG performance status ≤1 received lenvatinib (20 mg/day orally) + pembrolizumab (200 mg Q3W, IV). Patients were not preselected based on PD-L1 status. Tumor assessments were performed by study investigators using immune-related RECIST (irRECIST). The phase 2 primary end point was ORR at 24 weeks (ORRWK24). Secondary end points included ORR, progression-free survival (PFS), and duration of response (DOR).

**Results**

At the data cutoff of March 1, 2018, 21 patients were enrolled: 14 (67%) patients were PD-L1(+), 4 (19%) were PD-L1(-), 3 (14%) were not tested; and 38% of patients had ≥1 prior anticancer therapy. The primary end point of ORRWK24 was 47.6% (95% CI, 25.7–70.2). Additional efficacy outcomes are summarized in the table (Table 1). All patients experienced ≥1 treatment-related adverse event (TRAE). Grade 3 and 4 TRAEs occurred in 13 (62%) and 1 (5%; adrenal insufficiency) patients respectively. There were no fatal TRAEs. The most common any-grade TRAEs were fatigue (52%), decreased appetite (48%), diarrhea (48%), hypertension (48%), dysphonia (43%), and nausea (43%). Dose reduction and interruption due to TRAEs occurred in 13 (62%) and 10 (48%) patients, respectively.

**Conclusions**

The lenvatinib and pembrolizumab combination regimen was well-tolerated and demonstrated encouraging clinical activity. The combination may potentially improve upon the antitumor activity of anti-PD-1 monotherapies, supporting further evaluation of this regimen in patients with advanced melanoma.

**Trial Registration**

NCT02501096

**References**

1. Ribas A, et al. Pembrolizumab versus investigator-choice chemotherapy for ipilimumab-refractory melanoma (KEYNOTE-002): a randomised, controlled, phase 2 trial. Lancet Oncology. 2015;16(8):908-18.

2. Robert C, et al. Pembrolizumab versus ipilimumab in advanced melanoma. N Engl J Med. 2015;372(26):2521- 2532.

3. Kato Y et al. Effects of lenvatinib on tumor-associated macrophages enhance antitumor activity of PD-1 signal inhibitors. Mol Cancer Ther. 2015;14 (12Suppl2):A92.

4. Kato Y. Upregulation of memory T cell population and enhancement of Th1 response by lenvatinib potentiate antitumor activity of PD-1 signaling blockade. Cancer Res. 2017;77 (13 Suppl):4614.

**Ethics Approval**

This study was approved by all relevant institutional review boards.

**Table 1 (abstract P391).**
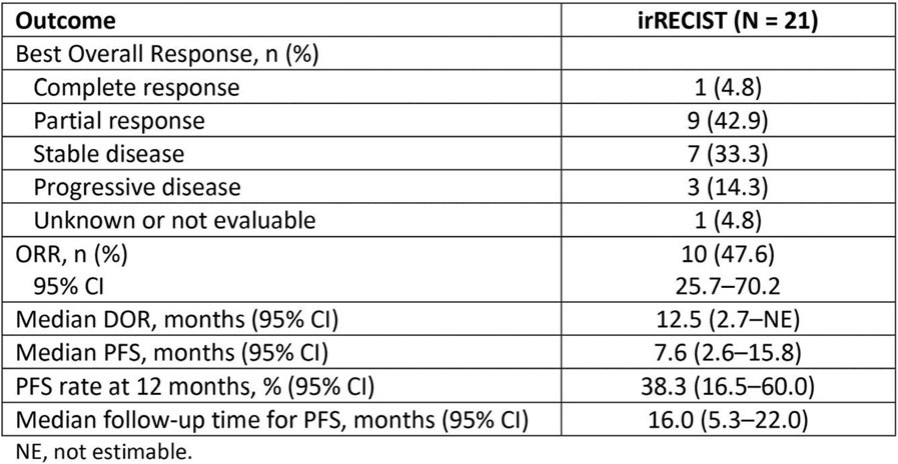
See text for description.

#### P392 A phase 1b/2 trial of lenvatinib in combination with pembrolizumab in patients with non-small cell lung cancer

##### Marcia Brose^2^, Nicholas Vogelzang, MD^3^, Christopher Di Simone^3^, Sharad Jain, MD^3^, Donald Richards^3^, Carlos Encarnacion^3^, Drew Rasco^4^, Robert Shumaker, PhD^5^, Corina Dutcus^5^, Daniel Stepan^5^, Matthew Guo^5^, Emmett Schmidt, MD PhD^6^, Matthew Taylor, MD^7^

###### ^1^Oxford PharmaGenesis, Oxford, UK; ^2^Abramson Cancer Center of the University, Philadelphia, PA, USA; ^3^McKesson Specialty Health, Las Vegas, NV,USA; ^4^South Texas Accelerated Research Therape, San Antonio, TX, USA; ^5^Eisai Inc., Woodcliff Lake, NJ, USA; ^6^Merck & Co., Inc., Kenilworth, NJ, USA; ^7^Oregon Health and Science University, Portland, OR, USA

####### **Correspondence:** Marcia Brose (Brosem@mail.med.upenn.edu)

**Background**

Lenvatinib is a multikinase inhibitor of VEGFR 1−3, FGFR 1−4, PDGFRα, RET, and KIT. Pembrolizumab, an anti-PD-1 antibody, is approved as a monotherapy for previously treated patients with metastatic PD-L1–positive (tumor proportion score [TPS] ≥1%) non-small cell lung cancer (NSCLC), with an objective response rate (ORR) of 18% [1]. We report interim results of an ongoing phase 1b/2 trial evaluating lenvatinib in combination with pembrolizumab in patient with solid tumors, focusing on the metastatic NSCLC cohort.

**Methods**

In this multicenter, open-label study (NCT02501096), patients with measurable, confirmed metastatic NSCLC and ECOG performance status ≤1 received lenvatinib (20 mg/day orally) and pembrolizumab (200 mg Q3W, IV). In the phase 2 portion, patients must have had ≤2 prior lines of systemic therapy; there was no limit for phase 1b. Patients were not preselected based on PD-L1 status. Tumor assessments were performed by study investigators using immune-related RECIST (irRECIST). The phase 2 primary end point was ORR at 24 weeks (ORRWK24). Secondary end points included ORR, progression-free survival (PFS), and duration of response (DOR).

**Results**

At the data cutoff of March 1, 2018, 21 patients were enrolled. 9 (43%) Patients were PD-L1(+) (TPS ≥1%); 5 (24%) were PD-L1(-); 7 (33%) were not tested. 3 (14%) Patients were treatment-naïve; 7 (33%), 10 (48%), and 1 (5%) patients had 1, 2, and ≥3 prior lines of systemic therapy, respectively. The primary end point of ORRWK24 was 33.3% (95% CI, 14.6–57.0). Additional efficacy outcomes are summarized in the table (Table 1). Grade 3 and 4 treatment-related adverse events (TRAEs) occurred in 10 (48%) and 1 (5%; increased aspartate aminotransferase) patients, respectively. There was 1 fatal TRAE (exsanguination; deemed “possibly related” to study treatment). The most common grade 3 TRAEs were hypertension (24%), fatigue (14%), diarrhea (14%), proteinuria (10%), and arthralgia (10%).

**Conclusions**

The combination of lenvatinib and pembrolizumab showed promising clinical activity with a manageable safety profile in previously treated patients with metastatic NSCLC who were not preselected for PD-L1 status. Further study is warranted.

**Trial Registration**

NCT02501096

**References**

1. Herbst RS et al. Pembrolizumab versus docetaxel for previously treated, PD-L1-positive, advanced non-small-cell lung cancer (KEYNOTE-010): a randomised controlled trial. Lancet. 2016;387(10027):1540-50.

**Ethics Approval**

This study was approved by all relevant institutional review boards.

**Table 1 (abstract P392).**
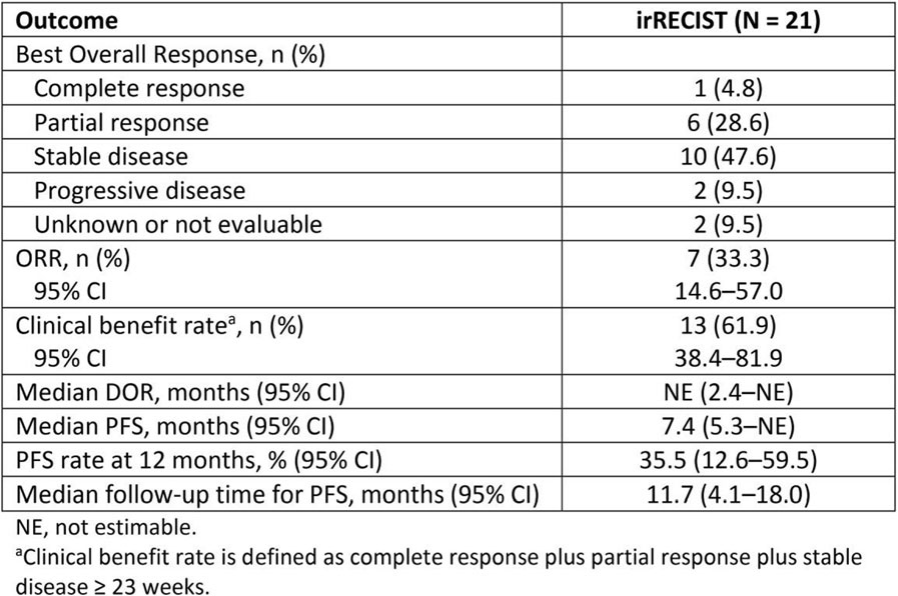
See text for description.

#### P393 A phase 1b/2 trial of lenvatinib in combination with pembrolizumab in patients with urothelial cancer

##### Nicholas Vogelzang, MD^2^, Carlos Encarnacion^2^, Allen Cohn^2^, Christopher Di Simone^2^, Drew Rasco^3^, Donald Richards^2^, Matthew Taylor, MD^4^, Corina Dutcus^5^, Daniel Stepan^5^, Robert Shumaker, PhD^5^, Matthew Guo^5^, Emmett Schmidt, MD PhD^6^, James Mier, MD^7^

###### ^1^Oxford PharmaGenesis, Oxford, UK; ^2^McKesson Specialty Health, Las Vegas, NV, USA; ^3^South Texas Accelerated Research Therape, San Antonio, TX, USA; ^4^Oregon Health and Science University, Portland, OR, USA; ^5^Eisai Inc., Woodcliff Lake, NJ, USA; ^6^Merck & Co., Inc., Kenilworth, NJ, USA; ^7^Beth Israel Deaconess Medical Center, Boston, MA, USA

####### **Correspondence:** Nicholas Vogelzang (nicholas.vogelzang@usoncology.com)

**Background**

Pembrolizumab, an anti-PD-1 antibody, is approved in the second-line setting for patients (objective response rate [ORR] 21%) with advanced/metastatic urothelial cancer and in the first-line setting for patients who are ineligible for cisplatin with combined positive score ≥10 or ineligible for platinum-based chemotherapy, with ORR (overall ORR 29%) [1–3]. However, there is still an unmet need for effective therapeutic options for advanced urothelial cancer. Lenvatinib is a multikinase inhibitor of VEGFR 1-3, FGFR 1-3, PDGFRα, RET and KIT. Tyrosine kinase inhibitors, such as lenvatinib, have demonstrated activity in urothelial cancer and may reverse the immunosuppressive environment that leads to immuno-oncology (IO) therapy failure. Here we present a phase 1b/2 trial to determine the safety and efficacy of lenvatinib in combination with pembrolizumab in patients with advanced urothelial cancer.

**Methods**

In this multicenter, open-label study (NCT02501096), patients with confirmed metastatic urothelial cancer and an ECOG PS of 0 or 1 received lenvatinib 20 mg orally once daily and 200 mg pembrolizumab intravenously every 3 weeks. Patients were not preselected based on PD-L1 status. The phase 2 primary end point was ORR at week 24 (ORRwk24), as assessed by study investigators using immune-related RECIST (irRECIST). Secondary end points included ORR, duration of response (DOR), and progression-free survival (PFS).

**Results**

At the time of data cutoff (March 1, 2018), 20 patients were enrolled. 9 (45%) Patients were PD-L1(+); 5 (25%) were PD-L1(-); 6 (30%) were not tested. 4 Patients (20%) were treatment-naïve, whereas 11 (55%) and 5 (25%) patients had had 1 and 2 lines of prior anticancer therapies, respectively. No patient had received prior IO therapy. The primary end point of ORRwk24 was 25% (95% CI: 8.7–49.1). Additional efficacy outcomes are summarized in the table (Table 1). 18 (90%) Patients experienced treatment-related adverse events (TRAEs). Grade 3 and 4 TRAEs occurred in 5 (25%) and 5 (25%) patients, respectively. There was 1 fatal TRAE (gastrointestinal hemorrhage). The most common any-grade TRAEs were proteinuria (45%), diarrhea (40%), fatigue (30%), hypertension (30%), and hypothyroidism (30%).

**Conclusions**

The tyrosine kinase inhibitor (lenvatinib) and immunotherapy (pembrolizumab) regimen demonstrated activity in this study, which included patients receiving later-line treatment. The combination of lenvatinib and pembrolizumab deserves further investigation in patients with metastatic urothelial cancer.

**Trial Registration**

NCT02501096

**References**

1. Bellmunt J, et al. Pembrolizumab as second-line therapy for advanced urothelial carcinoma. N Engl J Med. 2017;376(11):1015-26.

2. Vuky J et al. Updated efficacy and safety of KEYNOTE-052: A single arm phase 2 study investigating first-line pembrolizumab (pembro) in cisplatin-ineligible advanced urothelial cancer (UC). J Clin Oncol. 2018;36(15 Suppl):4524.

3. Keytruda® (pembrolizumab) [package insert]. Whitehouse Station, NJ: Merck & Co., Inc.; 2017.

**Ethics Approval**

This study was approved by all relevant institutional review boards.

**Table 1 (abstract P393).**
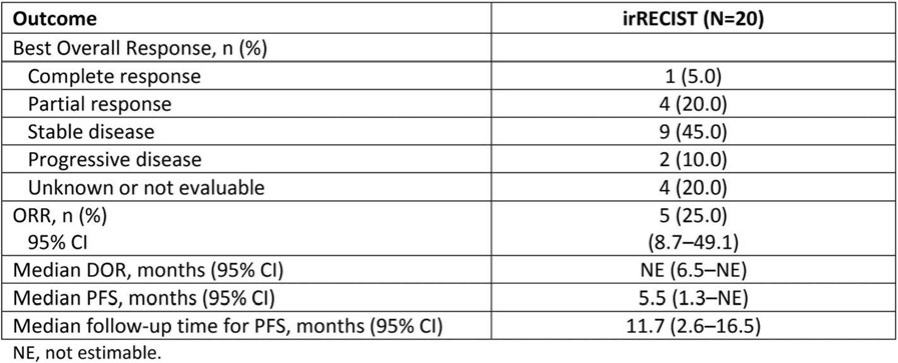
See text for description.

#### P394 CX3CR1+CD8+ T cells is responsible to the clinical benefit of chemoimmunotherapy in metastatic melanoma patients after disease progression on PD-1 blockade

##### Yiyi Yan, MD PhD, Roxana Dronca, MD, Svetomir Markovic, MD, PhD, Haidong Dong, MD, PhD

###### Mayo Clinic, Rochester, MN, USA

####### **Correspondence:** Yiyi Yan (yan.yiyi@mayo.edu)

**Background**

Clinical management of metastatic melanoma (MM) after PD-1 blockade failure remains a challenge and lacks a standard of care. Immunotherapy or chemotherapy alone provides limited benefit in this setting. Chemo-immunotherapy (CIT) combinations have demonstrated favorable efficacy and safety profiles in lung cancer patients. Our pre-clinical study in MM has shown that the addition of chemotherapy to PD-1 blockade can reshape a subset of therapy-responsive CD8+ T cells with resultant enhanced anti-tumor activities, suggesting the potential clinical benefits of CIT in MM patients whose disease have progressed after anti-PD-1 therapy. Further understanding of the clinical benefits and immunoregulatory mechanisms of CIT in this setting is crucial for the development of optimal combinatorial chemo-immunotherapeutic strategies to improve clinical outcomes in patients with advanced cancer.

**Methods**

MM patients (n=22) who have failed PD-1 blockade therapy were subsequently treated with CIT (paclitaxel and carboplatin in combination with pembrolizumab). The overall survival (OS), objective response rate (ORR), time-to- next therapy (TTNT), and toxicities were assessed. Using peripheral blood (PB) from MM patients, the phenotypic and functional changes induced by chemotherapy in therapy-responsive T cells, in the setting of anti-PD1 therapy, were examined. The immunoregulatory effects of CIT were also examined in melanoma mouse model.

**Results**

MM patients who have received subsequent CIT had a median OS of 5 years (95% CI: 2-NR) (median follow up of 3.9 years), with ORR of 61% (CR of 23%). The median TTNT was 8 months (95% CI: 6-15). No additional toxicities were identified. CX3CR1+CD8+ therapy-responsive T cells are low in MM patients who have failed to respond to anti-PD-1 monotherapy. However, in MM patients who responded to subsequent CIT, this subset of therapy-responsive T cells survived the chemotherapy with increased frequency and enhanced function. The clinical benefit of CIT is only observed in CX3CR1 wild type mice, not in KO mice, and ongoing PD-1 blockade is necessary to improve its anti-tumor activities.

**Conclusions**

In MM patients who have failed anti-PD-1 therapy, the chemo-immunotherapy combination showed favorable clinical outcomes and an acceptable toxicity profile. CX3CR1+ CD8+ effector T cells are responsible for the clinical benefit of CIT. This novel therapy-responsive population underlies the key cellular and molecular immunoregulatory mechanisms of chemotherapy. It serves as a meaningful marker to measure these collaborative effects and to develop the optimal chemo-immunotherapy strategy to improve clinical responses to current immune checkpoint blocking agents.

#### P395 PD-1 blockade synergies with RetroNectin-activated cytokine-induced killer cells in pretreated metastatic renal cell cancer

##### Lingdi Zhao^1^, Yonghao Yang^2^, Tiepeng Li^2^, Yong Zhang^2^, Wei Li^2^, Lu Han^2^, Hongwei Lin^2^, Quanli Gao^2^

###### ^1^Affiliated Cancer Hospital of Zhengzhou University & Henan Cancer Hospital, Zhengzhou, Peoples Republic of China; ^2^Affiliated Cancer Hospital of Zhengzhou, Zhengzhou, China

####### **Correspondence:** Quanli Gao (gaoquanli2015@126.com)

**Background**

Metastatic renal cell carcinoma (MRCC) has a poor prognosis after failure of multitargeted kinase inhibitors. New immunomodulators, such as anti-programmed death (PD)-1 antibody, have made progress in the treatment of MRCC, while the objective response rate is low. Therefore, it is urgent to improve the efficacy of anti-PD-1 therapy. We hypothesised that RetroNectin-activated cytokine-induced killer (R-CIK) cells could be combined safely with anti-PD-1 antibody and yield anti-tumor activity in patients with targeted therapy failed renal cell carcinoma.

**Methods**

Patients with MRCC were eligible if they were failed to at least one tyrosine kinase inhibitors, Eastern Cooperative Oncology Group performance status (ECOG PS) less than 3. Eligible patients received nivolumab (3mg/kg, q2w) or pembrolizumab (2mg/kg, q3w), and a dose of R-CIK cells about 5 to 10×109given intravenously twice a month. The primary objective was to determine the safety, secondary objectives included objective response rate (ORR), progression-free (PFS) survival and overall survival (OS).

**Results**

From May 2015 to May 2018, 15 patients were enrolled (median age 59 [45-79]), among which 12 patients were male. There were three patients received pembrolizumab and 12 patients received nivolumab therapy. No unexpected toxicities were observed associated with the therapy. Grade 1 fever occurred in one patient, grade 1 hypothyroidism occurred in 3 patients, grade 1/2 elevated transaminase occurred in 4 patients. For 12 patients evaluable for response, overall response rate was 75%, with complete remission rate 41.7 percent (5/12). The incidence of pseudo-progression was 25% (3/12). The PFS and OS were not reached.

**Conclusions**

PD-1 antibody combined R-CIK cells was found to be well tolerated and to have a manageable safety profile, and have synergistic effects in pretreated patients with metastatic renal cell cancer. Although only a pilot study, the ORR was encouraging in tyrosine kinase inhibitors therapy failed MRCC patients.

#### P396 Phase I trial of interferon-gamma (IFN-g) combined with nivolumab (nivo) in patients with advanced solid tumors

##### Matthew Zibelman, MD^1^, Alexander Macfarlane^1^, Kimberly Costello^1^, Thomas McGowan^1^, John O'Neill, BA^1^, Rutika Kokate^1^, Igor Astsaturov^1^, Hossein Borghaei, MS, DO^1^, Christina Chu, MD^1^, Crystal Denlinger^1^, Efrat Dotan, MD^1^, Daniel Geynisman^1^, Angela Jain, MD^1^, Lainie Martin, MD^1^, Elias Obeid, MD, MPH^1^, Joseph Treat, MD^1^, Namrata Vijayvergia, MD^1^, Rohit Walia^1^, Jennifer Winn, MD, MS^1^, Jeffery Nieves, PharmD^2^, Amy Grahn, MS^2^, Jeffrey Sherman, MD, FACP^2^, Karthik Devarajan^1^, Karen Ruth^1^, R Alpaugh, PhD^1^, Essel Al-Saleem^1^, Edna Cukierman, PhD^1^, Kerry Campbell, PhD^1^, Elizabeth Plimack, MD MS^1^

###### ^1^Fox Chase Cancer Center, Philadelphia, PA, USA; ^2^Horizon Pharma, Lake Forest, IL, USA

####### **Correspondence:** Matthew Zibelman (matthew.zibelman@fccc.edu)

**Background**

The presence of IFN-g is essential in the tumor microenvironment for a response to immune checkpoint blockade. We report a phase I trial evaluating the safety and preliminary efficacy of IFN-g/Nivo in patients with select solid tumors

**Methods**

Patients with advanced solid tumors (kidney, gastroesophageal, breast, ovarian, endometrial, small and non-small cell lung, anal, mesothelioma) who had progressed after > 1 prior therapy were recruited. Prior immunotherapy (ITx) was allowed. Eligible patients received IFN-g as part of four 6 patient cohorts, with dose levels ranging from 25-100 mcg/m2 (subq every other day), combined with nivo (3 mg/kg IV every 2 weeks). All patients had a baseline tumor biopsy, followed by one week of IFN-g induction alone, followed by an on-treatment biopsy and the addition of nivo. The primary endpoint was safety and to establish a recommended phase 2 dose (RP2D), with secondary efficacy endpoints.

**Results**

Twenty-six patients were accrued to four dose cohorts, with all evaluable for toxicity and 23 evaluable for efficacy. Median age was 60 years (33-76), 61.5% were female, and 26.9% had received prior ITx. Three dose limiting toxicities occurred, two at the highest dose level, thus 75 mcg/m2 was the max tolerated dose; however 50 mcg/m2 was selected as the RP2D based on study committee assessment of combined safety, efficacy, and correlative endpoints. The most common adverse events (AEs) irrespective of grade were predominantly known IFN-related side effects fatigue, fever, chills, and myalgias, and were predominantly < grade 3. Grade 3 AEs in three or more patients consisted of fatigue, hyponatremia, and leukopenia. No immune-related AE (irAE) was reported and no patient required steroids for an irAE. Nine patients had a new pleural effusion or ascites develop on study, two of whom came off study for a treatment-related AE. One patient with triple-negative breast cancer (TNBC) achieved a CR. Six patients had SD as best response, including 3/4 pts (75%) with renal cell carcinoma (RCC) evaluable for response, and 2/4 (50%) evaluable gastroesophageal cancer (GEC) patients. Two of seven patients (28.5%) who had received prior ITx achieved SD, including one patient each with RCC or GE cancer.

**Conclusions**

The combination of IFN-g and nivo was well tolerated and resulted in no irAEs. Further study in patients with RCC, GEC, and TNBC may be warranted, including in the post-immunotherapy setting.

**Acknowledgements**

Thank you to Horizon Pharma for providing funding support for this study

**Trial Registration**

Registered at ClinicalTrials.gov: NCT02614456

**Ethics Approval**

The study was approved by the Fox Chase Cancer Center Institutional Review Board, IRB number 15-1048

